# Global, Regional, and National Burden of Diabetes-Related Chronic Kidney Disease From 1990 to 2019

**DOI:** 10.3389/fendo.2021.672350

**Published:** 2021-07-01

**Authors:** Yujiao Deng, Na Li, Ying Wu, Meng Wang, Si Yang, Yi Zheng, Xinyue Deng, Dong Xiang, Yuyao Zhu, Peng Xu, Zhen Zhai, Dai Zhang, Zhijun Dai, Jie Gao

**Affiliations:** ^1^ Department of Nephrology, The Second Affiliated Hospital of Xi’an Jiaotong University, Xi’an, China; ^2^ Department of Oncology, The Second Affiliated Hospital of Xi’an Jiaotong University, Xi’an, China; ^3^ Department of Breast Surgery, The First Affiliated Hospital, College of Medicine, Zhejiang University, Hangzhou, China; ^4^ Celilo Cancer Center, Oregon Health Science Center Affiliated Mid-Columbia Medical Center, The Dalles, OR, United States

**Keywords:** diabetes-related chronic kidney disease, mortality, disability-adjusted life-years, incidence, prevalence

## Abstract

**Background:**

Chronic kidney disease (CKD) is a public health problem largely caused by diabetes. The epidemiology of diabetes mellitus–related CKD (CKD-DM) could provide specific support to lessen global, regional, and national CKD burden.

**Methods:**

Data were derived from the GBD 2019 study, including four measures and age-standardized rates (ASRs). Estimated annual percentage changes and 95% CIs were calculated to evaluate the variation trend of ASRs.

**Results:**

Diabetes caused the majority of new cases and patients with CKD in all regions. All ASRs for type 2 diabetes–related CKD increased over 30 years. Asia and Middle socio-demographic index (SDI) quintile always carried the heaviest burden of CKD-DM. Diabetes type 2 became the second leading cause of CKD and CKD-related death and the third leading cause of CKD-related DALYs in 2019. Type 2 diabetes–related CKD accounted for most of the CKD-DM disease burden. There were 2.62 million incident cases, 134.58 million patients, 405.99 thousand deaths, and 13.09 million disability-adjusted life-years (DALYs) of CKD-DM worldwide in 2019. Age-standardized incidence (ASIR) and prevalence rate (ASPR) of type 1 diabetes–related CKD increased, whereas age-standardized death rate (ASDR) and DALY rate decreased for females and increased for males. In high SDI quintile, ASIR and ASPR of type 1 diabetes–related CKD remained the highest, with the slowest increase, whereas the ASDR and age-standardized DALY rate remained the lowest there. In high SDI quintile, ASIR of type 2 diabetes–related CKD was the highest, with the lowest increasing rate. In addition, type 2 diabetes–related CKD occurred most in people aged 80-plus years worldwide. The main age of type 2 diabetes–related CKD patients was 55–64 years in Asia and Africa. The prevalence, mortality, and DALY rate of type 2 diabetes–related CKD increased with age. As for incidence, there was a peak at 80 years, and after age of 80, the incidence declined. CKD-DM-related anemia was mainly in mild to moderate grade.

**Conclusions:**

Increasing burden of CKD-DM varied among regions and countries. Prevention and treatment measures should be strengthened according to CKD-DM epidemiology, especially in middle SDI quintile and Asia.

## Introduction

Chronic kidney disease (CKD) remains a public health problem (1), which increases and affects over 75 million people worldwide (2, 3). At present, people suffer from CKD more than osteoarthritis, diabetes, or depression (4). CKD is ranked as the 12th leading cause of mortality (5) and was listed in 2013 as one of the top 10 causes of reduced life expectancy or disability-adjusted life-years (DALYs) (3). The burden of kidney disease varies greatly across the world, as does its testing and treatment (6, 7).

The most common causes of increased CKD burden are diabetes and hypertension. Diabetic nephropathy, the leading cause of end-stage renal disease (ESRD), is associated with the excess mortality in diabetic patients (8, 9). Moreover, diabetic CKD increased kidney disease–associated disability (10, 11) and triggered arterial disease and cardiovascular complications (12). Type 2 diabetes is gradually replacing infectious diseases as the main cause of CKD in less economically advanced countries, thereby causing competition for scarce medical resources (9). In patients with type 2 diabetes and mild/moderate CKD, use of metformin is associated with a significant reduction in all-cause mortality (13). In addition, the incidence of CKD caused by diabetes (CKD-DM) is determined by socioeconomic, cultural, and political factors, which have led to gaps in the current status of CKD prevention and management capabilities in countries around the world (14). Understanding the burden of CKD-DM in various countries and implementing early detection and management are important steps towards achieving equal kidney health.

Owing to broad array of data sources and scientific statistical modeling approaches (15, 16), GBD study can provide comprehensive estimates of CKD-DM burden to date. GBD 2019 study includes 369 diseases and injuries data in 204 countries and territories (4, 10). In this study, we aimed to investigate CKD-DM epidemiology and its variation trend at the global, regional, and national levels among different sex, age, and socio-demographic index (SDI). In this study, we provided a wide range of latest CKD-DM data, including incidence, prevalence, deaths, DALYs, and sequala among two sexes, four world regions, 21 regions, and 15 age-groups. These findings could provide specific guidance for decision-making and focus efforts toward the burden of inequities in CKD.

## Materials and Methods

### Study Population and Data Collection

We evaluated the CKD-DM burden (incidence, prevalence, deaths, and DALYs) and impairment (prevalence and YLDs) in 204 countries and territories within four world regions and 21 specific regions between 1990 and 2019 (**Appendix in Supplements**). All the data were retrieved using the Global Health Data Exchange (GHDx) query tool (http://ghdx.healthdata.org/gbd-results-tool). Four measures, age-standardized rates (ASRs), and impairment data of type 1 diabetes–related CKD (CKD-T1DM) and type 2 diabetes–related CKD (CKD-T2DM) were collected among different age-groups and gender. The age range included in this study was >10 years old and was segmented into 15 age-groups. Anemia (17), an impairment related to CKD-DM, was classified into three grades: mild, moderate, and severe.

SDI, ranging from 0 to 1, is a comprehensive measure of development and is an indicator of the overall fertility rate of women under 25 years of age, educational attainment, and lagging per capita income distribution in a country. Based on SDI values in 2019, countries and territories were classified into five categories: high, high-middle, middle, low-middle, and low.

### Statistical Analysis

All measures (counts, rates, and ASRs) were listed with a 95% uncertainty interval (UI). All rates in this study were reported per 100,000 individuals. We calculated estimated annual percentage changes (EAPCs) and their 95% CI to estimate the trend of ASRs, with the methods having been previously described (18, 19). When the EAPC and lower CI limit are positive, ASR increased. In contrast, when the EAPC and upper CI limit are negative, ASR decreased. The DisMod-MR 2.1 model, a Bayesian meta-regression method, was used for each measure. This study was approved by the Ethics Committee of the Second Affiliated Hospital, College of Medicine, Xi’an Jiaotong University. The access to and use of GBD study data did not require informed patient consent. This study followed the Guidelines for Accurate and Transparent Health Estimates (GATHER) Reporting guideline.

## Results

### Global Findings

In 2019, diabetes and CKD have become the seventh largest non-communicable diseases, the fourth leading cause of death, and the sixth leading cause of disability worldwide (**Figure S1**). CKD-T1DM was responsible for 12.9 thousand incident cases, 5.02 million patients, 8.20 thousand deaths, and 3.22 million DALYs in 2019, which increased by 75.09, 88.41, 89.73, and 72.63%, respectively, over 30 years worldwide (**Table S1**). Additionally, CKD-T2DM was associated with 2.5 million incident cases, 129.56 million patients, 405.99 thousand deaths, and 9.87 million DALYs, which increased by 156.49, 94.78, 172.39, and 141.73%, respectively (**Table 1**). Type 2 diabetes has become the second leading cause of CKD and CKD–related deaths and the third leading cause of CKD related DALYs in 2019 (**Figure S2**).

**Table 1:** The global and regional burden of chronic kidney disease caused by diabetes mellitus type 2.

Location	Sex	Incident cases (No. ×1000) (95%UI)		Prevalent cases (No. ×1000) (95%UI)		Deaths (No. ×1000) (95%UI)		DALYs (No. ×1000) (95%UI)	
		1990	2019	1990	2019	1990	2019	1990	2019
Global	Both	975.17(881.61-1077.11)	2501.25(2279.95-2740.78)	66515.19(60763.91-72748.74)	129560.07(119058.25-140047.7)	149.05(119.54-179.27)	405.99(328.43-484.98)	4083.28(3296.98-4859.14)	9870.47(8114.78-11736.44)
	Female	501.35(453.54-553.86)	1231.07(1124.19-1345.36)	34280.02(31266.23-37584.92)	65757(60532.52-71003.17)	73.33(59.22-88.53)	200.52(161.86-242.49)	1972.76(1601.93-2330.19)	4699.38(3859.13-5533.42)
	Male	473.82(427.82-524.2)	1270.18(1153.23-1393.48)	32235.17(29380.16-35247.29)	63803.08(58520.06-69067.59)	75.72(60.07-92.34)	205.47(165.23-247.34)	2110.52(1667.64-2545.21)	5171.09(4183.01-6233.02)
**Socio-demographic index**									
High SDI	Both	359.06(324.69-397.2)	710.94(648.66-775.19)	12128.36(11230.29-13033.34)	20966.26(19592.28-22376.84)	25.1(19.96-30.87)	79.74(61.62-99.49)	607.35(503.48-719.08)	1525(1252.92-1803.41)
	Female	194.22(175.53-214.66)	348.39(317.68-380.09)	6506.58(6034.87-6980.88)	10702.68(9990.61-11414.09)	14.2(11.18-17.66)	43.18(32.43-54.08)	324.78(269.52-386.02)	761.65(621.46-901.87)
	Male	164.84(148.36-182.57)	362.55(329.16-396.61)	5621.78(5194.52-6056.07)	10263.58(9569.04-10980.83)	10.9(8.69-13.39)	36.56(28.49-45.57)	282.56(231.46-335.16)	763.34(622.06-909.67)
High-middle SDI	Both	228.81(205.59-253.75)	557.41(505.53-612.61)	17088.34(15547.74-18733.64)	29513.21(27085.29-31891.68)	28.45(22.86-34.44)	62.02(49.89-75.24)	773.61(619.61-915.55)	1495.23(1229.3-1766.94)
	Female	122.3(109.98-135.67)	283.26(257.03-311.13)	9205.8(8393.29-10109.68)	15592.18(14302.68-16860.54)	14.26(11.44-17.38)	31.8(25.29-38.86)	380(308.84-450.27)	735.4(607.6-869.75)
	Male	106.51(95.43-118.11)	274.15(247.81-301.35)	7882.54(7184.33-8645.36)	13921.02(12746.11-15075.49)	14.19(11.24-17.36)	30.22(24.38-36.78)	393.61(311.77-472.32)	759.83(624.02-909.52)
Low SDI	Both	37.22(33.4-41.59)	100.53(90.57-111.68)	3786.14(3421.47-4195.79)	8498.37(7718.03-9331.47)	13.3(9.98-16.47)	28.93(22.62-35.57)	350.6(268.47-435.29)	754.94(587.97-928.13)
	Female	16.72(15.02-18.73)	49.8(44.83-55.2)	1820.41(1640.21-2026.23)	4111.38(3749.58-4498.82)	5.57(4.11-7.1)	13.12(10.15-16.25)	151.49(113.16-190.22)	345.36(269.94-429.53)
	Male	20.5(18.34-22.92)	50.73(45.55-56.35)	1965.73(1774.44-2177.77)	4386.99(3976.13-4834.14)	7.74(5.72-9.77)	15.8(12-19.79)	199.11(148.38-248.67)	409.58(313.34-509.5)
Low-middle SDI	Both	117.55(105.34-131.11)	347.55(313.72-384.69)	11700.33(10543.17-12982.04)	24407.41(22284.9-26589.21)	28.95(22.06-35.96)	79.2(61.95-96.51)	835.98(648.63-1026.23)	2104.04(1648.65-2571.91)
	Female	52.31(46.88-58.48)	165.09(148.68-182.69)	5566.86(5009.5-6203.64)	11825.13(10815.17-12817.21)	12.53(9.5-15.68)	36.18(27.75-44.19)	365.91(285.2-455.46)	948.6(752.73-1152.68)
	Male	65.24(58.21-73.09)	182.46(164.27-202.96)	6133.47(5552.86-6802.53)	12582.28(11477.75-13754.45)	16.42(12.31-21.05)	43.02(32.9-53.8)	470.08(355.91-596.68)	1155.44(887.1-1446.44)
Middle SDI	Both	232.05(207.35-259.87)	748.73(680.43-820.32)	21779.21(19643.7-24126.38)	46105(42148.52-50013.89)	53.15(43.11-63.02)	155.84(126.59-185.47)	1512.98(1213.59-1800.8)	3984.29(3268.66-4743.62)
	Female	115.54(103.17-129.15)	383.81(349.73-420.78)	11163.8(10055.93-12438.78)	23491.01(21509.14-25462.71)	26.73(21.71-31.88)	76.11(61.78-91.3)	749.27(603.7-894.02)	1905.01(1553.57-2255.29)
	Male	116.51(104.14-130.7)	364.91(330.24-402.1)	10615.41(9579.45-11733.26)	22614(20661.74-24521.16)	26.42(21.17-31.92)	79.73(64.38-95.83)	763.71(602.78-921.29)	2079.27(1676.95-2498.64)
**Region**									
Africa	Both	56.42(62.89-50.79)	194.97(216.65-176.19)	4066.44(4453.6-3693.69)	9929.63(10751.36-9071.61)	18.91(23.3-14.36)	42.68(53.14-32.82)	465.17(572.22-357.18)	1044.81(1285.39-810.78)
	Female	29.02(32.44-26)	95.19(105.79-85.93)	2080.58(2288.36-1889.57)	5005.81(5416.61-4583.23)	8.95(11.12-6.78)	20.93(26.03-15.98)	223.58(274.12-172.88)	510.66(634.56-393.77)
	Male	27.4(30.51-24.66)	99.78(110.83-89.85)	1985.86(2171.63-1800.35)	4923.82(5358.23-4506.43)	9.97(12.53-7.49)	21.75(27.48-16.58)	241.59(300.3-184.07)	534.15(671.31-409.28)
America	Both	215.37(239.37-194.09)	537.18(587.97-490.17)	8636.25(9262.04-7977.4)	18428.64(19681.15-17173)	20.09(24.52-15.97)	89.55(107.9-71.74)	533.44(631.65-430.33)	2026.44(2412.62-1647.08)
	Female	113.31(125.13-102.45)	270.9(296.04-247.8)	4605.25(4936.99-4255.85)	9533.55(10187.78-8881.42)	10.58(13.03-8.38)	45.44(54.93-36.5)	272.73(322.28-220.86)	993.07(1186.56-814.61)
	Male	102.06(114.12-91.23)	266.28(292.35-242.09)	4030.99(4323.97-3721.44)	8895.09(9515.83-8278.45)	9.51(11.59-7.48)	44.1(53.44-34.85)	260.72(309.71-208.79)	1033.37(1241.41-833.43)
Asia	Both	449.36(499.4-404.34)	1292.03(1418.95-1169.83)	41362.54(45796.5-37448.97)	83892.12(91100.56-76589.75)	91.82(109.7-74.3)	235.33(279.84-191.47)	2644.54(3142.28-2122.98)	6075.01(7221.28-4960.95)
	Female	210.93(234.32-189.26)	615.98(676.64-558.96)	20783.2(23149.92-18742.64)	42104.75(45602.97-38433.06)	43.69(52.08-35.33)	112.05(134.34-90.19)	1240.87(1481.96-992.52)	2811.43(3333.34-2302.49)
	Male	238.43(265.49-214.95)	676.05(744.83-611.18)	20579.34(22730.62-18644.22)	41787.38(45542.12-38207.77)	48.12(58.86-37.99)	123.27(148.76-98.87)	1403.68(1694.39-1100.63)	3263.59(4002.04-2634.77)
Europe	Both	252.41(278.24-227.39)	472.57(515.61-431.49)	12356(13362.41-11404.76)	17125.45(18338.75-15943.6)	17.82(22.72-13.55)	37.46(48.89-27.46)	430.21(528.12-339.33)	702.63(861.49-558.14)
	Female	147.28(162.58-132.79)	246.77(269.16-224.91)	6763.59(7308.31-6262.98)	9020.59(9624.32-8412.57)	9.91(12.71-7.51)	21.62(28.78-15.62)	231.07(282.47-182.43)	374.06(458.96-298)
	Male	105.13(116.72-94.17)	225.8(245.8-205.7)	5592.41(6063.73-5139.49)	8104.85(8708.2-7520.43)	7.9(10.2-6)	15.85(20.84-11.93)	199.15(246.65-155.04)	328.57(406.66-258.02)
Andean Latin America	Both	4.04(3.65-4.52)	21.14(19.05-23.33)	334.11(281.14-402.51)	962.88(823.71-1131.26)	1.2(0.94-1.49)	5.28(3.98-6.77)	28.31(21.98-34.68)	110.23(84.08-140.91)
Australasia	Both	8.34(7.74-9.04)	19.49(17.62-21.48)	227.65(211.32-243.83)	467.45(433.92-503.36)	0.12(0.09-0.16)	0.57(0.39-0.83)	4.01(3.2-5.02)	12.75(9.62-16.43)
Caribbean	Both	5.45(4.89-6.08)	17.09(15.54-18.81)	391.01(354.87-432.69)	779.97(719.12-843.52)	1.21(0.97-1.46)	3.38(2.59-4.22)	31.69(25.28-38.22)	85.23(66.85-105.5)
Central Asia	Both	6.32(5.48-7.28)	17.52(15.27-20.08)	841.44(758.01-938.02)	1434.75(1308.45-1573.23)	1.22(0.91-1.58)	3.02(2.33-3.72)	39(30.09-48.79)	92.94(72.42-114.09)
Central Europe	Both	27.97(24.83-31.68)	61.6(55.09-68.62)	1808.73(1659.88-1970.37)	2488.48(2307.31-2677.07)	2.65(1.97-3.36)	4.12(3.02-5.46)	72.43(54.82-90.3)	100.97(76.78-127.36)
Central Latin America	Both	31.71(28.36-35.62)	128.82(117.94-139.96)	1994.18(1802.07-2211.15)	5292.85(4897.66-5697.45)	5.07(4.11-6.05)	30.39(24-37.22)	138.58(112.85-163.25)	763.21(606.4-925.52)
Central Sub-Saharan Africa	Both	2.64(2.35-2.98)	8.13(7.27-9.09)	330.93(293.01-379.47)	794.64(710.14-894.94)	1.34(0.99-1.74)	2.76(1.92-3.71)	34.97(25.59-45.31)	70.9(49.8-94.95)
East Asia	Both	172.07(152.47-193.06)	458.41(413.64-506.95)	17631.4(15827-19724.77)	32313.15(29293.79-35236.46)	29.39(23.59-35.42)	67.92(53.83-81.92)	904.82(712.05-1092.64)	1760.71(1419.11-2118.73)
Eastern Europe	Both	37.73(33.33-42.63)	65.22(57.77-73.96)	4750.71(4335.35-5188.57)	5467.98(5017.55-5927.62)	1.24(0.86-1.67)	2.15(1.51-2.93)	50.43(38.26-64.67)	73.48(55.97-94.26)
Eastern Sub-Saharan Africa	Both	8.78(7.89-9.76)	23.72(21.23-26.37)	1045.6(944.12-1149.85)	2383.87(2162.64-2613.37)	4.69(3.57-5.9)	8.91(7.04-11.04)	117.8(90.32-145.99)	213.36(167.74-261.06)
High-income Asia Pacific	Both	73.77(67.09-81.64)	169.23(153.07-185.9)	3097.73(2849.74-3359.26)	5565.4(5189.09-5942.61)	8.66(7.17-10.18)	20.33(15.45-24.95)	194.22(164.37-222.92)	339.78(282.43-396.59)
High-income North America	Both	139.29(123.77-155.14)	262.82(236.67-291.2)	4205.65(3887.71-4527.02)	7459.82(6908.89-8008.41)	6.85(5.06-9.04)	34.02(26.61-42.21)	187.26(145.44-230.87)	706.43(566.96-843.06)
North Africa and Middle East	Both	60.32(54.04-67.27)	265.94(241.66-294.37)	2800.34(2558.83-3051.41)	8178.36(7522.26-8778.6)	15.14(11.85-19.22)	34.94(26.93-43.88)	370.21(293.31-453.59)	863.7(678.14-1069.67)
Oceania	Both	0.48(0.42-0.53)	1.44(1.26-1.61)	76.08(66.76-87.25)	173.89(154.87-196.4)	0.18(0.14-0.23)	0.49(0.38-0.63)	6.11(4.69-7.63)	16.12(12.34-20.44)
South Asia	Both	113.51(101-127.35)	336.49(300.75-375.01)	12131.69(10921.7-13489.19)	26710.17(24346.73-29149.49)	25.02(17.91-32.35)	75.5(56.32-94.73)	734.25(546.15-940.75)	2053.21(1534.68-2589.37)
Southeast Asia	Both	49.85(44.64-55.49)	181.86(164.12-200.65)	6441.62(5765.08-7213.32)	13931.04(12695.85-15228.08)	21.69(17.7-25.72)	54.67(44.56-65.66)	625.79(499.38-750.46)	1475.06(1195.58-1779.95)
Southern Latin America	Both	13.17(11.7-14.64)	32.55(29.53-35.83)	497.25(453.91-538.82)	988.22(914.99-1066.04)	2.56(2.01-3.14)	6.33(4.94-7.94)	57.06(45.28-68.25)	119.6(94.7-145.44)
Southern Sub-Saharan Africa	Both	5.89(5.25-6.66)	16.92(15.3-18.83)	418.34(380-460.58)	875.76(802.24-952.59)	1.13(0.83-1.47)	3.7(2.84-4.63)	29.62(22.02-38.25)	89.63(68.71-111.86)
Tropical Latin America	Both	23.01(20.67-25.52)	78.31(71.21-86.59)	1285.14(1174.08-1403.04)	3080.38(2849.35-3306.98)	3.56(2.84-4.28)	10.92(8.76-13.2)	98.49(79.2-117.44)	258.3(210.29-307.04)
Western Europe	Both	176.26(158.42-195.24)	293.23(266.22-320.3)	5080.3(4713.54-5452.88)	7474.3(6975.82-7958.19)	10.39(7.55-13.9)	24.91(17.37-34.13)	222.1(171.72-278.1)	380.27(294.05-489.27)
Western Sub-Saharan Africa	Both	14.57(13.14-16.09)	41.31(37.22-45.69)	1125.29(1026.97-1235.53)	2736.7(2489.79-2988.72)	5.75(4.24-7.41)	11.67(8.91-14.67)	136.13(99.13-173.95)	284.6(216.36-359.54)

DALY, disability adjusted life-year; UI, uncertainty interval; SDI, socio-demographic index.

**Figure 1:**
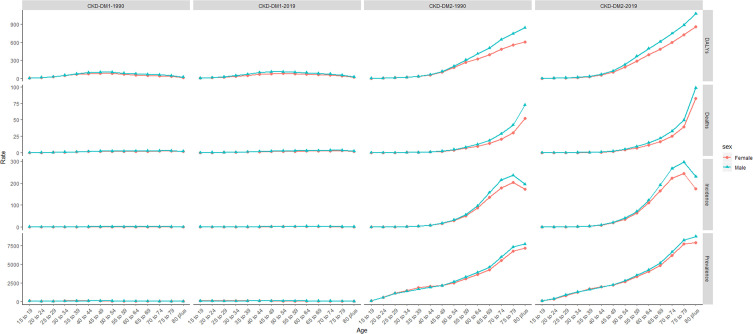
The incidence, prevalence, death, and DALY rate of CKD-DM burden from 1990 to 2019. CKD-DM1-1990 represents the incidence, prevalence, death, and DALY rate of type 1 diabetes–related CKD in 1990. CKD-DM1-2019 represents the incidence, prevalence, death, and DALY rate of type 1 diabetes–related CKD in 2019. CKD-DM2-1990 represents the incidence, prevalence, death, and DALY rate of type 2 diabetes–related CKD in 1990. CKD-DM2-2019 represents the incidence, prevalence, death, and DALY rate of type 2 diabetes-related CKD in 2019. CKD-DM, chronic kidney disease caused by diabetes; DALY, disability adjusted life-year. The vertical axis is the incidence, prevalence, death, and DALY rate (per 100,000 people), and the horizontal axis is the different age-groups (years).

**Figure 2:**
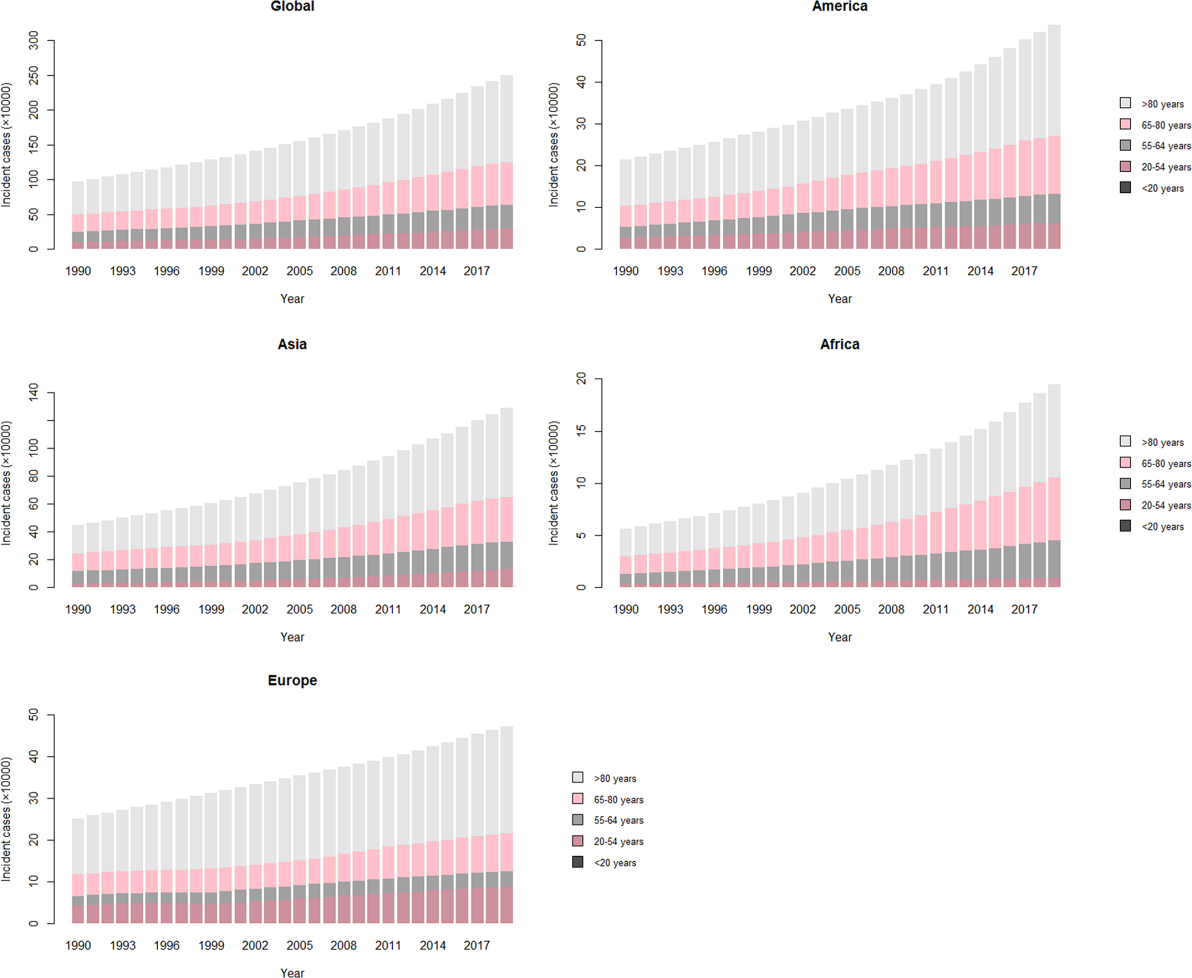
The number of type 2 diabetes–related CKD incident cases over 30 years. The vertical axis is the incident cases of type 2 diabetes–related CKD in four world regions (America, Asia, Africa, and Europe). The horizontal axis represents 30 years (1990–2019). Each column is the total number of incident cases among five age-groups (>80 years, 65–80 years, 55–64 years, 20–54 years, and <20 years) that year. CKD, chronic kidney disease.

As for the variation of ASRs, both age-standardized incidence rate (ASIR) and prevalence rate (ASPR) of CKD-T1DM exhibited upward trends in both genders globally (ASIR: EAPC = 1.21, 95% CI: 1.08–1.35; ASPR: EAPC = 1.15, 95% CI: 0.10–1.31). Interestingly, age-standardized death rate (ASDR) and DALY rate remained stable over 30 years (ASDR: EAPC = 0.08, 95% CI: −0.02–0.19; DALY: EAPC = −0.08, 95% CI: −0.18–0.02), falling for women but rising for men (**Table S2**). All ASRs of CKD-T2DM increased among women and men worldwide (**Table 2**). Further analysis indicated that incidence, prevalence, mortality of CKD-T1DM remained stable in all age-groups and gender. However, DALY rate showed a peak at 40–59 years (**Figure 1**). As for CKD-T2DM, the prevalence, mortality, and DALY rate increased with age. In four world regions, CKD-T2DM occurred mostly in people aged 80-plus years (**Figure 2**). The main age at which people develop CKD-T2DM, deaths, and DALYs is presented in **Figures S3**-**5**.

**Table 2:** The age-standardized rates and variation trends of diabetes mellitus type 2–related chronic kidney disease.

Location	Sex	ASIR (95%UI)		EAPC (95%CI)	ASPR (95%UI)		EAPC (95%CI)	ASDR (95%UI)		EAPC (95%CI)	Age-standardized DALY rate (95%UI)		EAPC (95%CI)
		1990	2019		1990	2019		1990	2019		1990	2019	
Global	Both	24.88(22.6-27.39)	30.29(27.65-33.05)	0.65(0.63-0.66)	1526.04(1396.82-1658.25)	1576.35(1448.28-1700.21)	0.06(0.04-0.09)	4.14(3.35-4.98)	5.16(4.2-6.17)	0.92(0.79-1.06)	101.71(82.95-120.08)	120.2(99.16-142.85)	0.75(0.63-0.87)
	Female	23.53(21.35-25.89)	28.11(25.69-30.74)	0.6(0.58-0.61)	1511.42(1383.69-1647.5)	1539.3(1415.97-1662.32)	0.05(0.02-0.08)	3.63(2.95-4.39)	4.57(3.7-5.53)	0.94(0.82-1.06)	91.99(74.8-108.81)	107.8(88.51-126.96)	0.69(0.58-0.79)
	Male	26.58(24.14-29.28)	32.86(29.96-35.88)	0.68(0.66-0.7)	1548.06(1420.38-1675.76)	1619.23(1487.83-1749.47)	0.07(0.04-0.11)	4.89(3.93-5.94)	5.91(4.79-7.14)	0.83(0.68-0.97)	114.29(92-136.53)	134.5(109.41-160.69)	0.76(0.62-0.9)
**Socio-demographic index**													
High SDI	Both	33.61(30.48-36.95)	37.74(34.43-41.16)	0.25(0.2-0.31)	1205.47(1113.38-1293.97)	1250.65(1162.65-1341.5)	0.04(0.01-0.07)	2.38(1.91-2.91)	3.69(2.9-4.51)	1.72(1.5-1.93)	58.49(48.34-68.99)	80.85(66.99-95.28)	1.28(1.09-1.47)
	Female	31.3(28.34-34.32)	34.35(31.4-37.47)	0.21(0.16-0.26)	1164.38(1074.72-1249.21)	1209.99(1121.13-1299.26)	0.06(0.02-0.09)	2.18(1.74-2.68)	3.31(2.58-4.06)	1.64(1.41-1.86)	54.02(44.85-63.6)	72.67(60.43-85.6)	1.16(0.96-1.35)
	Male	36.85(33.42-40.58)	41.67(38-45.49)	0.23(0.17-0.3)	1267(1173-1359.65)	1303.08(1214.18-1396.37)	-0.01(-0.05-0.03)	2.73(2.19-3.37)	4.17(3.27-5.17)	1.68(1.49-1.88)	64.8(53.12-76.69)	90.37(74.26-107.3)	1.33(1.14-1.52)
High-middle SDI	Both	21.41(19.36-23.65)	27.03(24.56-29.74)	0.88(0.86-0.9)	1524.61(1394.09-1662.33)	1528.13(1398.48-1655.86)	0.02(-0.01-0.06)	2.99(2.43-3.63)	3.13(2.54-3.79)	0.34(0.17-0.51)	72.84(59.13-86.62)	73.88(61.08-87.11)	0.27(0.12-0.42)
	Female	20.04(18.12-22.2)	25.15(22.84-27.63)	0.84(0.81-0.87)	1534.9(1399.23-1680.73)	1526.26(1396.3-1655.27)	0.03(-0.01-0.07)	2.51(2.02-3.04)	2.73(2.17-3.33)	0.43(0.27-0.58)	63.7(52.01-75.22)	65.99(54.35-78.06)	0.3(0.16-0.44)
	Male	23.59(21.24-26.05)	29.54(26.84-32.46)	0.87(0.84-0.9)	1523.6(1396.24-1654.39)	1535.61(1410.33-1663.21)	0.01(-0.03-0.04)	3.84(3.06-4.73)	3.75(3.02-4.61)	0.13(-0.06-0.32)	86.51(69.61-103.39)	84.11(69.8-100.32)	0.16(0-0.32)
Low SDI	Both	15.65(14.2-17.3)	19.61(17.7-21.68)	0.93(0.87-0.99)	1275.18(1167.68-1387.65)	1325.6(1218.77-1433.87)	0.01(-0.04-0.06)	6.9(5.27-8.63)	6.69(5.26-8.16)	-0.12(-0.19–0.05)	149.58(115.74-182.93)	146.36(115.93-178)	-0.07(-0.15-0)
	Female	14.26(12.87-15.86)	19.01(17.09-21.04)	0.99(0.96-1.01)	1234.51(1131.55-1347.02)	1273.82(1173.66-1373.46)	0.01(-0.04-0.06)	5.8(4.35-7.58)	5.88(4.62-7.26)	0.04(0-0.08)	129.35(98.11-162.2)	130.76(103.2-160.77)	0.03(-0.01-0.07)
	Male	16.98(15.38-18.83)	20.24(18.29-22.36)	0.9(0.8-1)	1314.47(1202.12-1433.01)	1378.76(1264.97-1494.27)	0.02(-0.03-0.07)	8.02(6.01-10.34)	7.58(5.82-9.47)	-0.19(-0.3–0.08)	169.42(128.6-211.12)	162.94(126.37-201.68)	-0.12(-0.23–0.01)
Low-middle SDI	Both	19.17(17.32-21.27)	25.18(22.81-27.8)	0.83(0.78-0.88)	1589.04(1455.16-1735.41)	1626.8(1495.56-1764.1)	-0.08(-0.14–0.01)	5.64(4.43-6.94)	6.41(5.07-7.8)	0.45(0.28-0.62)	135.5(106.29-165.68)	152.04(119.82-184.33)	0.46(0.29-0.62)
	Female	17.28(15.57-19.24)	23.06(20.85-25.45)	0.91(0.87-0.95)	1530.34(1396.91-1678.25)	1542.18(1413.95-1666.62)	-0.11(-0.17–0.04)	4.9(3.77-6.01)	5.57(4.35-6.79)	0.45(0.33-0.56)	119.3(93.1-147.31)	132.46(105.6-159.37)	0.38(0.28-0.48)
	Male	21.02(18.95-23.32)	27.49(24.86-30.31)	0.8(0.73-0.87)	1645.58(1503.21-1793.96)	1715.16(1573.13-1860.8)	-0.04(-0.1-0.03)	6.41(4.87-8.26)	7.36(5.76-9.1)	0.5(0.27-0.73)	151.6(116.86-190.7)	173.3(134.47-214.64)	0.55(0.33-0.77)
Middle SDI	Both	22.36(20.07-24.89)	29.39(26.77-32.19)	1.14(1.09-1.19)	1730.5(1580.15-1892.77)	1784.84(1637.95-1928.44)	0.12(0.09-0.15)	6.21(5.13-7.38)	7.03(5.79-8.35)	0.65(0.56-0.74)	143.21(117.35-167.24)	159.63(132.14-188.19)	0.63(0.53-0.73)
	Female	21.63(19.39-24.09)	28.95(26.45-31.67)	1.07(1.03-1.12)	1761.37(1603.45-1932.91)	1769.3(1623.71-1914.5)	0.07(0.03-0.1)	5.85(4.83-7.05)	6.38(5.15-7.65)	0.49(0.4-0.58)	138.25(112.44-162.95)	146.37(120.26-173.15)	0.42(0.33-0.51)
	Male	23.28(20.9-25.89)	29.92(27.21-32.9)	1.2(1.11-1.29)	1702.54(1555.65-1852.27)	1802.96(1651.97-1949.35)	0.17(0.13-0.21)	6.71(5.47-8.04)	7.85(6.37-9.41)	0.79(0.69-0.88)	149.59(121.97-177.17)	174.55(143.29-208.71)	0.82(0.71-0.92)
**Region**													
Africa	Both	19.93(18.02-22.14)	31.53(28.61-34.82)	1.62(1.59-1.66)	1166.31(1075.35-1260.2)	1331.27(1229.8-1430.13)	0.42(0.4-0.44)	8.1(6.23-10.07)	8.54(6.68-10.62)	0.25(0.21-0.29)	167.24(130.39-204.11)	173.75(136.33-213.95)	0.2(0.16-0.24)
	Female	20.1(18.14-22.46)	29.44(26.75-32.6)	1.42(1.32-1.51)	1181.96(1086.14-1280.44)	1301.53(1202.73-1395.88)	0.34(0.33-0.35)	7.37(5.66-9.23)	7.96(6.14-9.99)	0.36(0.32-0.39)	156.62(121.91-192.55)	163(127.42-200.92)	0.23(0.19-0.26)
	Male	19.81(17.93-21.88)	33.84(30.62-37.4)	1.83(1.78-1.89)	1151.24(1061.29-1242.84)	1365.31(1261.02-1467.95)	0.51(0.47-0.55)	8.96(6.79-11.32)	9.2(7.02-11.64)	0.14(0.09-0.2)	179.04(136.55-222.11)	185.65(143.4-233.64)	0.18(0.13-0.23)
America	Both	35.15(31.84-38.92)	42.08(38.52-45.93)	0.51(0.45-0.56)	1365.5(1265.06-1464.74)	1507.84(1401.23-1611.33)	0.27(0.24-0.31)	3.36(2.67-4.11)	6.88(5.53-8.28)	2.65(2.31-3)	87.55(70.61-103.98)	159.55(130.3-189.7)	2.19(1.89-2.48)
	Female	33.46(30.34-36.9)	39.41(36.14-42.93)	0.45(0.39-0.51)	1343.57(1241.32-1442.57)	1461.49(1356.91-1562.6)	0.22(0.18-0.25)	3.07(2.44-3.76)	6.18(4.96-7.44)	2.55(2.16-2.94)	81.29(65.76-96.19)	144.25(118.24-171.56)	2.05(1.71-2.38)
	Male	37.32(33.59-41.43)	45.22(41.18-49.58)	0.55(0.49-0.61)	1396.83(1293.07-1499.89)	1562.78(1454.16-1672.99)	0.32(0.28-0.36)	3.77(2.99-4.6)	7.76(6.15-9.41)	2.72(2.42-3.02)	95.49(76.92-113.48)	177.54(143.75-212.34)	2.3(2.05-2.56)
Asia	Both	22.3(20.15-24.62)	26.7(24.26-29.28)	0.61(0.6-0.62)	1709.44(1559.63-1868.1)	1705.51(1560.86-1849.21)	-0.04(-0.07–0.01)	5.42(4.48-6.47)	5.34(4.38-6.35)	0.04(-0.07-0.15)	127.59(104.29-149.71)	125.92(103.65-148.98)	0.13(0.02-0.25)
	Female	20.46(18.4-22.65)	24.53(22.3-26.9)	0.66(0.65-0.67)	1715.71(1562.7-1885.73)	1683.3(1540.06-1822.49)	-0.05(-0.08–0.02)	4.89(4.02-5.82)	4.71(3.78-5.65)	-0.07(-0.16-0.01)	117.49(95.42-139.45)	112.5(91.92-132.92)	-0.01(-0.09-0.08)
	Male	24.33(22.02-26.89)	29.13(26.43-31.95)	0.58(0.55-0.6)	1707.16(1561.63-1856.81)	1731.3(1589.18-1877.52)	-0.02(-0.06-0.01)	6.13(4.97-7.44)	6.13(5-7.35)	0.13(-0.01-0.27)	139.28(112.14-166.1)	140.8(115.64-169.88)	0.25(0.1-0.39)
Europe	Both	23.82(21.51-26.15)	30.57(27.96-33.37)	0.82(0.79-0.84)	1269.7(1166.51-1373.35)	1269.95(1175.27-1366.01)	-0.1(-0.15–0.05)	1.77(1.36-2.25)	2.13(1.6-2.75)	1.09(0.96-1.22)	41.77(33.14-51.04)	44.42(35.31-54.08)	0.46(0.39-0.53)
	Female	22.95(20.75-25.17)	28.31(25.75-30.97)	0.68(0.65-0.71)	1228.51(1129.87-1329.52)	1209.49(1119.17-1301.51)	-0.13(-0.17–0.09)	1.53(1.16-1.94)	1.94(1.44-2.52)	1.29(1.14-1.44)	37.25(29.75-45.45)	40.2(32.33-48.64)	0.47(0.41-0.53)
	Male	25.47(22.92-28.04)	33.68(30.73-36.64)	0.92(0.89-0.94)	1327.69(1225.96-1433.46)	1343.9(1241.99-1447.52)	-0.08(-0.13–0.03)	2.26(1.71-2.92)	2.43(1.83-3.18)	0.65(0.53-0.77)	49.32(38.7-61.37)	50.09(39.51-61.86)	0.33(0.25-0.42)
Andean Latin America	Both	19.96(18.06-22.26)	37.98(34.1-41.96)	2.23(2.11-2.35)	1401.81(1181.45-1679.85)	1660.07(1426-1945.63)	0.53(0.51-0.56)	6.51(5.11-8.06)	9.85(7.38-12.66)	1.66(1.36-1.96)	138.91(108.25-169.77)	198.82(151.5-253.93)	1.43(1.16-1.7)
Australasia	Both	33.96(31.6-36.64)	38.4(34.75-42.31)	0.34(0.28-0.4)	978.34(907.27-1049.67)	1012.3(937-1096.36)	0.07(0.03-0.12)	0.58(0.43-0.78)	1.02(0.7-1.46)	2.58(2.18-2.98)	17.34(13.81-21.58)	25.2(19.08-32.44)	1.53(1.27-1.78)
Caribbean	Both	20.72(18.6-23.15)	32.91(29.94-36.13)	1.55(1.43-1.66)	1367.33(1247.76-1495.93)	1524.92(1405.22-1646.62)	0.33(0.31-0.35)	4.94(3.99-5.93)	6.52(5-8.15)	1.43(1.29-1.58)	121.07(97.08-146.39)	164.33(129.45-202.86)	1.44(1.33-1.54)
Central Asia	Both	13.01(11.43-14.88)	21.75(19.19-24.65)	1.89(1.71-2.07)	1574.2(1430.38-1732.96)	1693.4(1553.87-1845.1)	0.22(0.17-0.26)	2.66(1.99-3.46)	4.48(3.5-5.54)	1.73(1.3-2.15)	78.61(60.41-98.13)	117.66(93.25-142.98)	1.17(0.77-1.58)
Central Europe	Both	18.61(16.6-20.87)	28.65(25.85-31.68)	1.24(1.12-1.35)	1280.91(1173.03-1395.65)	1338.32(1232.46-1445.35)	0.05(0.01-0.08)	1.87(1.41-2.35)	1.83(1.35-2.4)	0.2(-0.05-0.44)	48.91(37.72-60.54)	47.48(36.46-59.44)	0.09(-0.07-0.24)
Central Latin America	Both	36.83(33.01-41.52)	53.46(49.17-58.15)	1.18(1.1-1.27)	1949.19(1784.98-2120.56)	2174.88(2015.47-2342.85)	0.3(0.27-0.33)	6.99(5.68-8.33)	13.25(10.48-16.16)	2.44(2.1-2.79)	164.34(135.96-192)	320.2(255.6-388.56)	2.5(2.15-2.85)
Central Sub-Saharan Africa	Both	11.78(10.63-12.97)	15.86(14.22-17.42)	1.04(0.95-1.14)	1094.58(991.23-1217.6)	1114.36(1016.32-1221.23)	0(-0.04-0.03)	7.81(5.94-9.87)	6.86(4.81-9.13)	-0.54(-0.59–0.5)	158.7(119.27-200.54)	137.93(98.29-181.44)	-0.58(-0.63–0.53)
East Asia	Both	19.76(17.64-22.04)	21.7(19.63-24.02)	0.53(0.44-0.61)	1683.22(1529.89-1856.17)	1614.67(1465.8-1768.41)	0.02(-0.08-0.12)	4.04(3.32-4.8)	3.71(2.96-4.44)	0.04(-0.1-0.19)	100.28(80.75-119.3)	86.06(69.83-102.66)	-0.08(-0.24-0.08)
Eastern Europe	Both	13.54(12.05-15.21)	19.38(17.33-21.82)	1.33(1.14-1.51)	1791.58(1632.18-1961.24)	1781.78(1633.91-1931.68)	-0.07(-0.11–0.03)	0.48(0.35-0.64)	0.61(0.44-0.84)	0.81(0.62-1.01)	18.28(13.99-23.18)	21.53(16.5-27.48)	0.46(0.33-0.6)
Eastern Sub-Saharan Africa	Both	12.01(10.9-13.23)	15.19(13.64-16.83)	0.8(0.73-0.87)	1063.77(974.74-1159.28)	1097.74(1012.52-1186.64)	0.05(0.02-0.08)	7.71(5.91-9.86)	7.12(5.64-8.87)	-0.35(-0.41–0.28)	160.33(123.68-199.27)	139.28(110.65-169.71)	-0.58(-0.65–0.5)
High-income Asia Pacific	Both	36.22(33-39.95)	38.16(34.72-41.98)	0.03(-0.01-0.08)	1536.74(1416.33-1664.2)	1486.78(1379.2-1599.84)	-0.2(-0.22–0.17)	4.86(4.02-5.73)	3.49(2.76-4.19)	-1.16(-1.3–1.02)	98.87(83.9-113.3)	73.24(61.75-85.24)	-0.87(-1.06–0.68)
High-income North America	Both	38.8(34.84-43.15)	41.61(37.62-45.89)	0.09(0-0.18)	1232.98(1136.28-1328.65)	1320.68(1220.86-1417.1)	0.18(0.13-0.22)	1.88(1.39-2.46)	5.05(3.93-6.21)	3.58(3.15-4.01)	53.76(41.87-66.08)	113.61(91.76-135.29)	2.73(2.38-3.08)
North Africa and Middle East	Both	35.05(31.66-38.83)	61.33(55.98-67.44)	2.03(1.92-2.14)	1413.38(1306.46-1525.98)	1762.51(1636.48-1893.37)	0.79(0.76-0.82)	10.67(8.3-14.01)	9.58(7.47-11.95)	-0.21(-0.3–0.13)	222.15(175.54-274.43)	205.94(162.38-253.6)	-0.13(-0.18–0.07)
Oceania	Both	15.17(13.44-16.89)	19.77(17.47-22.07)	0.77(0.71-0.83)	1839.48(1661.26-2051.21)	1870.4(1692.5-2060.51)	-0.03(-0.06-0)	7.12(5.7-8.71)	8.23(6.58-10.14)	0.34(0.14-0.53)	182.83(144.01-224.89)	206.91(164.32-256.32)	0.27(0.07-0.47)
South Asia	Both	19.13(17.16-21.38)	23.58(21.1-26.18)	0.53(0.43-0.63)	1691.3(1547.37-1846.62)	1714.22(1569.95-1864.4)	-0.2(-0.31–0.09)	5.34(3.92-6.91)	5.88(4.47-7.41)	0.23(-0.06-0.52)	127.52(95.75-161.41)	143.36(108.63-179.93)	0.45(0.19-0.71)
Southeast Asia	Both	19.14(17.27-21.2)	29.11(26.35-31.92)	1.41(1.39-1.44)	1982.97(1801.55-2179.38)	2094.35(1918.32-2276.54)	0.12(0.08-0.15)	9.57(8.03-11.28)	10.06(8.22-12.04)	0.24(0.17-0.31)	226.44(184.91-267.62)	235.7(194.17-281.24)	0.23(0.17-0.3)
Southern Latin America	Both	28.35(25.28-31.4)	38.54(34.99-42.37)	1.04(0.95-1.13)	1074.34(981.95-1160.8)	1226.92(1133.37-1327.77)	0.44(0.41-0.47)	5.86(4.61-7.2)	7.37(5.76-9.24)	0.81(0.46-1.16)	124.21(99.31-148.26)	142.69(113.09-173.69)	0.47(0.18-0.77)
Southern Sub-Saharan Africa	Both	21.47(19.23-24.08)	29.93(27.01-32.99)	1.05(0.85-1.25)	1260.86(1159.11-1368.86)	1382.43(1276.29-1490.27)	0.24(0.18-0.31)	4.74(3.53-6.23)	7.86(6.14-9.87)	2.32(1.97-2.66)	106.17(79.5-135.34)	162.32(125.88-201.31)	2.01(1.7-2.33)
Tropical Latin America	Both	24.63(22.18-27.37)	32.01(29.15-35.36)	0.83(0.76-0.9)	1202.69(1107.92-1299.73)	1255.66(1161.7-1346.01)	0.08(0.05-0.11)	4.59(3.7-5.49)	4.71(3.78-5.67)	0.17(0.08-0.26)	106.48(86.54-126.13)	106.63(87.46-126.49)	-0.1(-0.22-0.02)
Western Europe	Both	29.12(26.21-31.99)	31.65(28.77-34.47)	0.19(0.12-0.25)	946.84(875.58-1018)	938.14(871.53-1003.36)	-0.19(-0.25–0.13)	1.73(1.27-2.31)	2.13(1.52-2.87)	1.17(1.04-1.31)	37.65(29.23-46.55)	37.99(29.94-47.8)	0.2(0.14-0.25)
Western Sub-Saharan Africa	Both	16.69(15.1-18.31)	22.47(20.24-24.77)	1.13(1.03-1.22)	1056.66(971.5-1142.21)	1156.15(1065.21-1241.99)	0.29(0.26-0.31)	8.01(6-10.38)	7.8(6.06-9.8)	-0.15(-0.22–0.08)	159.76(118.81-202.51)	155.3(119.85-194.88)	-0.13(-0.2–0.06)

ASIR, age-standardized incidence rate; ASPR, age-standardized prevalence rate; ASDR, age-standardized death rate; DALY, disability adjusted life-year; EAPC, estimated annual percentage change; SDI, socio-demographic index; UI, uncertainty interval.

### SDI Findings

**Figure 3:**
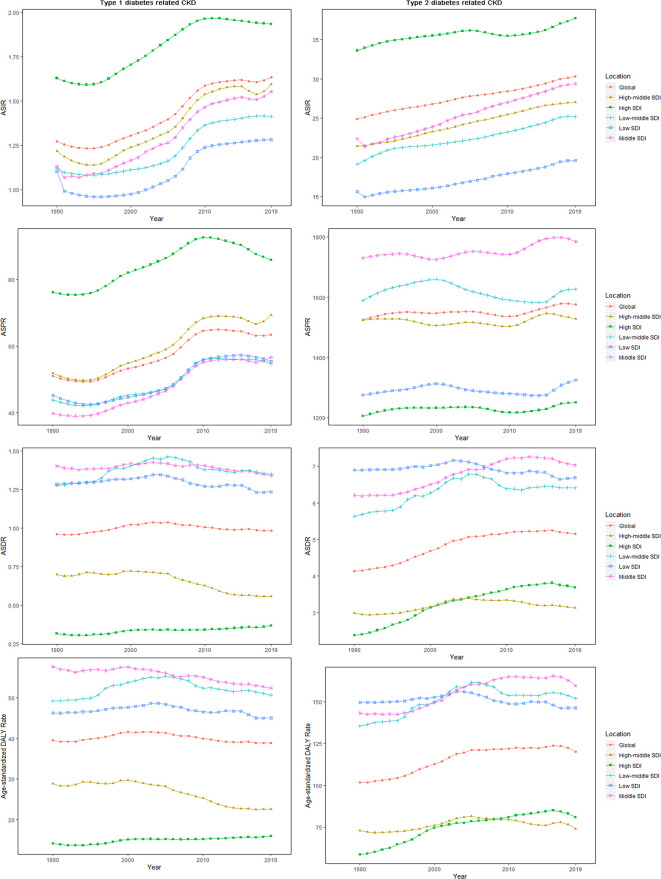
The age-standardized rates for CKD-DM among SDI quintiles over 30 years. The vertical axis is the age-standardized incidence, prevalence, death, and DALY rate (per 100,000 person-years), and the horizontal axis is the 30 years (1990–2019). Each point represents the age-standardized incidence, prevalence, death, and DALY rate (per 100,000 person-years) that year. Each color and shape represents an SDI quintile (Global, High SDI, High-middle SDI, Middle SDI, Low-middle SDI, and Low SDI). CKD-DM, type 1 diabetes–related chronic kidney disease; DALY, disability adjusted life-year; ASIR, age-standardized incidence rate; ASPR, age-standardized prevalence rate; ASDR, age-standardized death rate; SDI, socio-demographic index.

From 1990 to 2019, middle SDI quintile carried the heaviest burden of CKD-DM (**Tables 1** and **S1**). **Figure 3** shows the drift of CKD-DM among five SDI quintiles over 30 years. ASIR and ASPR of CKD-T1DM remained the highest in high SDI quintile, with the slowest increase (ASIR: EAPC = 0.90, 95% CI: 0.77–1.03; ASPR: EAPC = 0.74, 95% CI: 0.58–0.90), whereas they increased the fastest in middle SDI quintile (ASIR: EAPC = 1.53, 95% CI: 1.39–1.68; ASPR: EAPC = 1.62, 95% CI: 1.45–1.78), where they carried the highest age-standardized DALY rate (**Table S2**).

The ASIR of CKD-T2DM remained the highest in high SDI quintile, with the slowest increasing rate (EAPC = 0.25, 95% CI: 0.20–0.31). Among five SDI categories, ASIR (EAPC = 1.14, 95% CI: 1.09–1.19) and ASPR (EAPC = 0.12, 95% CI: 0.09–0.15) increased the fastest in middle SDI quintile. Only in low-middle SDI quintile did ASPR show a downward trend (EAPC = −0.08, 95% CI: −0.14–−0.01). ASDR and age-standardized DALY rate of CKD-T2DM increased the fastest in high SDI quintile (ASDR: EAPC = 1.72, 95% CI: 1.50–1.93; DALY: EAPC = 1.28, 95% CI: 1.09–1.47), especially for males (**Table 2**).

**Figure 4:**
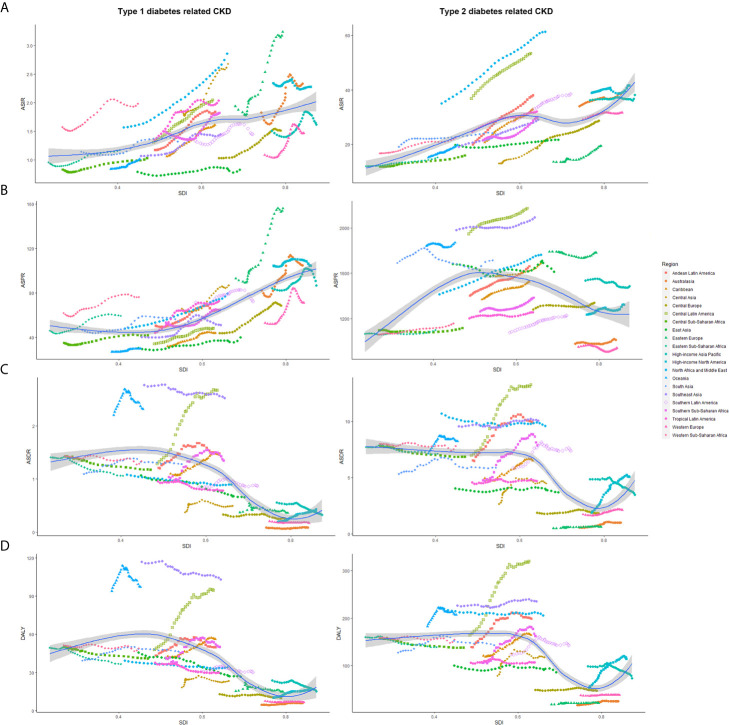
The age-standardized rates of CKD-DM among 21 regions based on SDI in 2019. The vertical axis is the age-standardized incidence, prevalence, death, and DALY rate (per 100,000 person-years), and the horizontal axis is the SDI value in 2019. Each combination of colors and shapes represents a region, 21 in total. Each point represents the age-standardized incidence, prevalence, death, and DALY rate (per 100,000 person-years) that year in this region. Each combination of the same color and shape, from front to back, is the data for each year from 1990 to 2019. **(A)** ASIR (per 100,000 population); **(B)** ASDR (per 100,000 population); **(C)** ASPR (per 100,000 population); **(D)** Age-standardized DALY rate (per 100,000 population). ASIR, age-standardized incidence rate; ASPR, age-standardized prevalence rate; ASDR, age-standardized death rate; CKD-DM, diabetes-related chronic kidney disease; DALY, disability adjusted life-year; SDI, socio-demographic index.

**Figure 4** showed the variation of ASRs with the increase of SDI value among 21 regions. ASIR increased with the SDI value. As opposed to CKD-T1DM, ASPR of CKD-T2DM rose before SDI value of 0.5 and then began to decline again. As for ASDR and DALY, they had two turning points with SDI value of 0.6 and 0.8.

### Regional Findings

Asia carried the heaviest burden of CKD-DM, especially in South and East (**Tables 1** and **S1**). The region with the highest ASIR of CKD-T1DM changed from High-income North America in 1990 (ASIR: 2.34, 95% UI:1.99–2.73; ASPR: 104.38, 95% UI: 92.23–117.03) to Eastern Europe in 2019 (ASIR: 3.24, 95% UI: 2.70–3.87; ASPR: 156.02, 95% UI: 135.02–181.61, **Table S2**). Similarly, that with the highest ASIR of CKD-T2DM changed from High-income North America in 1990 (38.80, 95% UI: 34.84–43.15) to North Africa and Middle East in 2019 (61.33, 95% UI: 56.00–67.44).

ASIR and ASPR of CKD-T1DM increased in most regions, and the fastest in Eastern Europe (ASIR: EAPC = 2.46, 95% CI: 2.17–2.75; ASPR: EAPC = 2.41, 95% CI: 2.14–2.69). Only in High-income North America, the ASIR of CKD-T1DM decreased (EAPC = −0.11, 95% CI: −0.20–−0.02), with the slowest decrease of ASPR (EAPC = 0.11, 95% CI: 0.01–0.21, **Table S2**).

ASIR of CKD-T2DM increased in all regions, except for High-income Asia Pacific (EAPC = 0.03, 95% CI: −0.01–0.08) and High-income North America (EAPC = 0.09, 95% CI: −0.01–0.18). ASPR of CKD-T2DM decreased the fastest in South Asia (EAPC = −0.20, 95% CI: −0.31–−0.09). Alarmingly, both ASDR and age-standardized DALY rate of CKD-T2DM increased most rapidly in High-income North America (ASDR: EAPC = 3.58, 95% CI: 3.15–4.01; DALY: EAPC = 2.73, 95% CI: 2.38–3.08), but decreased largely in High-income Asia Pacific (ASDR: EAPC = −1.16, 95% CI: −1.30–−1.02; DALY: EAPC = −0.87, 95% CI: −1.06–−0.68, **Table 2**).

### National Findings

The detailed data of CKD-T1DM and CKD-T2DM among 204 countries and territories are presented in **Tables S3**-**6**. China carried the highest burden of CKD-DM, followed by the United States and India. From 1990 to 2019, incident cases of CKD-T1DM increased the most in France (130.28%, 95% UI: 76.26–202.92, **Table S3**).

In 2019, people in China had the lowest ASIR of CKD-T1DM (0.81, 95% UI: 0.64–1.03). In addition, it increased faster in France than other countries (ASIR: EAPC = 3.00, 95% CI: 2.58–3.42). ASDR decreased most rapidly in Greece (EAPC = −4.57, 95% CI: −5.95–−3.17, **Table S4**).

Incident cases of CKD-T2DM increased in most countries and territories. The number of patients with CKD-T2DM increased most in Greenland (128.68%, 95% UI: 109.28–149.71), but decreased most in Afghanistan (−23.93%, 95% UI: −27.49–−19.84). Only in Solomon Islands, deaths and DALYs of CKD-T2DM decreased, and they grew largely in Armenia and El Salvador (**Table S5**). ASIR of CKD-T2DM increased faster in Morocco (EAPC = 2.73, 95% CI: 2.64–2.81) and Turkey (EAPC = 2.58, 95% CI: 2.37–2.79, **Table S6**).

### Impairment Associated With CKD-DM

CKD-T1DM resulted in 655,237 cases of anemia in 2019 (mild: 56.62%; moderate: 40.27%; severe: 3.11%), increased by 82.32% over 30 years (**Table 3**). In addition, CKD-T2DM contributed to 566,181 cases of anemia in 2019 (mild: 62.19%; moderate: 34.51%; severe: 3.30%), increased by 138.55%. Years lived with disability (YLDs) of CKD-T1DM- and CKD-T2DM-related anemia grew by 56.61 and 102.88%, respectively (**Table S7**).

**Table 3:** The prevalent cases and ASPR of impairment caused by diabetes mellitus–related chronic kidney disease.

Location	Impairment	Sex	Diabetes mellitus type 1–related chronic kidney disease					Diabetes mellitus type 2–related chronic kidney disease				
			Patient cases (No. ×1000) (95% UI)		ASPR (95% UI)		EAPC (95% CI)	Patient cases (No. ×1000) (95% UI)		ASPR (95% UI)		EAPC (95% CI)
			1990	2019	1990	2019		1990	2019	1990	2019	
Global	Anemia	Both	359.39(408.79-314.74)	655.24(741.99-577.15)	6.58(5.81-7.43)	8.48(7.46-9.57)	1.3(1.15-1.46)	3455.45(3817.46-3116.84)	8243.06(9070.84-7466.37)	89.15(80.39-98.24)	101.69(92.25-111.69)	0.54(0.51-0.57)
		Female	188.46(214.93-164.87)	358.02(410.74-311.18)	6.86(6.03-7.82)	9.29(8.08-10.6)	1.44(1.29-1.58)	1753.15(1936.67-1580.67)	4003.13(4419.19-3603.71)	81.47(73.5-89.79)	92.01(82.98-101.48)	0.54(0.51-0.58)
		Male	170.92(195.9-149.12)	297.22(337.86-261.36)	6.33(5.56-7.23)	7.72(6.77-8.77)	1.16(1-1.32)	1702.3(1893.46-1520.71)	4239.93(4682.53-3824.64)	101.94(91.43-112.61)	115.61(104.46-127.43)	0.48(0.45-0.5)
	Mild anemia	Both	180.23(202.72-159.05)	371.01(422.29-327.67)	3.39(3.01-3.83)	4.71(4.16-5.34)	1.63(1.46-1.8)	1934.53(2134.57-1741.24)	5126.35(5645.34-4643.76)	49.94(45.05-55.02)	63.21(57.37-69.6)	0.93(0.89-0.98)
		Female	90.03(102.7-78.83)	189.47(217.77-164.64)	3.29(2.89-3.74)	4.87(4.24-5.6)	1.75(1.6-1.89)	802.38(883.91-722.93)	2037.82(2249.03-1835.13)	37.23(33.59-41.05)	46.88(42.23-51.66)	0.98(0.93-1.04)
		Male	90.2(102.9-79.29)	181.54(206.66-159.04)	3.51(3.08-3.98)	4.6(4.04-5.22)	1.52(1.32-1.71)	1132.15(1257.05-1011.71)	3088.53(3415.83-2793.3)	67.56(60.59-74.75)	83.92(76.1-92.78)	0.82(0.79-0.85)
	Moderate anemia	Both	162.83(187-140.85)	263.86(301.92-230.18)	2.9(2.53-3.31)	3.5(3.04-4.01)	1(0.87-1.14)	1356.68(1501.94-1224.79)	2844.45(3148.81-2563.94)	34.97(31.56-38.55)	35.12(31.7-38.81)	0.07(0.04-0.1)
		Female	89.88(103.44-77.85)	157.39(181.2-136.39)	3.26(2.84-3.74)	4.12(3.58-4.74)	1.2(1.05-1.35)	854.03(944.94-770.7)	1804.56(2004.47-1622.38)	39.75(35.91-43.92)	41.45(37.28-45.99)	0.22(0.19-0.25)
		Male	72.95(85.55-62.29)	106.47(123.43-91.78)	2.55(2.2-2.96)	2.87(2.47-3.34)	0.73(0.61-0.86)	502.65(558.24-447.13)	1039.89(1150.01-934.35)	30.25(27.06-33.53)	28.61(25.83-31.54)	-0.18(-0.22–0.13)
	Severe anemia	Both	16.33(18.98-13.93)	20.36(23.64-17.62)	0.29(0.25-0.34)	0.27(0.23-0.32)	0.17(0.03-0.31)	164.24(182.67-147.46)	272.27(304.6-244.69)	4.24(3.81-4.71)	3.36(3.02-3.75)	-0.83(-0.92–0.75)
		Female	8.56(10.09-7.25)	11.15(12.97-9.57)	0.31(0.27-0.36)	0.29(0.25-0.34)	0.24(0.07-0.41)	96.74(108.01-86.41)	160.75(180.23-144.72)	4.49(4.02-5.02)	3.68(3.32-4.13)	-0.66(-0.72–0.59)
		Male	7.77(9.17-6.51)	9.21(11.11-7.74)	0.27(0.23-0.32)	0.25(0.21-0.3)	0.08(-0.05-0.2)	67.5(75.48-59.96)	111.51(125.42-99.63)	4.13(3.69-4.62)	3.08(2.76-3.43)	-1.13(-1.24–1.02)
**Socio-demographic index**												
High SDI	Anemia	Both	52.24(61.6-44.76)	72.01(82.87-62.09)	6.07(5.19-7.2)	6.29(5.41-7.31)	0.49(0.37-0.62)	851.24(949.21-766.96)	1652.9(1853.2-1484.69)	79.86(72.07-88.83)	83.3(74.85-93.14)	0.2(0.14-0.26)
		Female	31.56(37.76-26.76)	40.54(47.37-34.79)	7.25(6.11-8.71)	7.62(6.47-9.06)	0.44(0.29-0.58)	431.97(483.64-385.73)	741.18(837.2-650.87)	69.28(61.88-77.22)	69.47(61.05-78.43)	0.11(0.03-0.19)
		Male	20.68(24.32-17.61)	31.47(37.24-26.62)	5.05(4.27-5.96)	5.16(4.34-6.11)	0.61(0.39-0.84)	419.28(472.27-372.61)	911.72(1039.8-800.91)	99.43(89.08-111.35)	102.84(90.54-116.58)	0.11(0.07-0.15)
	Mild anemia	Both	39.08(45.95-33.45)	56.71(65.28-48.98)	4.49(3.85-5.31)	4.87(4.19-5.66)	0.66(0.53-0.78)	627.3(699.34-562.94)	1274.6(1432.5-1144.21)	58.8(53.02-65.44)	64.66(58.21-72.31)	0.36(0.32-0.4)
		Female	21.94(26.32-18.51)	29.27(34.3-25.13)	5.04(4.23-6.06)	5.53(4.68-6.58)	0.57(0.41-0.73)	273.95(306.07-243.12)	495.43(556.02-438.86)	44.14(39.4-49.37)	47.11(41.91-53.1)	0.31(0.25-0.36)
		Male	17.14(20.15-14.65)	27.44(32.62-23.08)	4.08(3.48-4.81)	4.36(3.68-5.14)	0.78(0.57-1)	353.36(398.56-313.89)	779.16(887.15-686.78)	83.14(74.16-93.35)	88.04(77.67-99.83)	0.17(0.14-0.21)
	Moderate anemia	Both	12.72(15.3-10.72)	14.83(17.24-12.76)	1.53(1.28-1.84)	1.38(1.18-1.62)	-0.01(-0.13-0.11)	214.27(240.57-190.65)	364.08(414.81-317.87)	20.15(17.99-22.52)	17.95(15.75-20.43)	-0.28(-0.39–0.17)
		Female	9.31(11.27-7.82)	10.94(12.92-9.31)	2.14(1.79-2.61)	2.03(1.71-2.38)	0.11(-0.01-0.23)	151.97(172.13-132.96)	238.07(276.2-203.54)	24.18(21.36-27.29)	21.68(18.53-25)	-0.24(-0.35–0.13)
		Male	3.41(4.29-2.75)	3.89(4.73-3.23)	0.93(0.73-1.18)	0.78(0.61-1)	-0.19(-0.46-0.08)	62.3(72-53.59)	126(152.96-105.08)	15.37(13.32-17.56)	14.07(11.77-16.98)	-0.21(-0.32–0.1)
	Severe anemia	Both	0.44(0.56-0.36)	0.47(0.56-0.4)	0.05(0.04-0.07)	0.04(0.03-0.05)	-0.4(-0.56–0.24)	9.67(10.93-8.5)	14.23(16.64-12.15)	0.91(0.81-1.02)	0.69(0.59-0.8)	-0.79(-0.97–0.6)
		Female	0.31(0.39-0.25)	0.33(0.4-0.27)	0.07(0.06-0.09)	0.06(0.05-0.07)	-0.2(-0.33–0.07)	6.05(6.99-5.19)	7.67(9.39-6.17)	0.95(0.82-1.09)	0.68(0.56-0.83)	-0.92(-1.11–0.73)
		Male	0.13(0.17-0.1)	0.14(0.17-0.12)	0.03(0.03-0.04)	0.02(0.02-0.03)	-0.77(-1.07–0.47)	3.62(4.21-3.09)	6.56(8.06-5.38)	0.91(0.78-1.06)	0.73(0.6-0.9)	-0.64(-0.8–0.48)
High-middle SDI	Anemia	Both	67.76(79.87-57.64)	104.99(124.02-87.91)	5.83(4.96-6.88)	7.07(5.93-8.49)	1.1(0.94-1.25)	785.88(873.46-707.68)	1610.42(1787.07-1451.96)	74.87(67.54-82.85)	79.26(71.58-87.83)	0.34(0.3-0.38)
		Female	35.62(42.52-29.95)	59.93(73.15-49.26)	6(5.06-7.16)	8.33(6.8-10.33)	1.58(1.43-1.74)	405.53(451.8-363.44)	800.41(888.37-719.69)	67.38(60.53-74.78)	71.98(65.04-79.97)	0.43(0.38-0.49)
		Male	32.14(38.39-27.09)	45.06(53.98-37.48)	5.74(4.86-6.83)	5.98(5.01-7.2)	0.56(0.4-0.73)	380.35(424.82-340.71)	810.01(900.84-727.65)	89.34(80.12-98.78)	91.61(82.52-101.44)	0.18(0.15-0.21)
	Mild anemia	Both	40.41(46.95-34.45)	72.14(85.25-60.58)	3.45(2.95-4.02)	4.71(3.94-5.62)	1.6(1.42-1.78)	475.87(529.72-426.95)	1114.64(1234.89-1004.58)	45.03(40.57-49.95)	54.8(49.5-60.57)	0.89(0.83-0.95)
		Female	19.47(22.88-16.45)	37.2(45.26-30.63)	3.25(2.74-3.82)	5.17(4.18-6.37)	2.15(1.97-2.33)	200.98(224.64-179.72)	461.96(513.39-415.6)	33.2(29.79-37.07)	41.65(37.52-46.24)	1.11(1.02-1.2)
		Male	20.94(24.6-17.8)	34.94(41.35-29.16)	3.71(3.17-4.35)	4.37(3.68-5.19)	1.09(0.89-1.29)	274.89(306.41-245.12)	652.68(731.46-584.46)	63.73(57-70.83)	73.45(65.87-81.59)	0.63(0.59-0.66)
	Moderate anemia	Both	25.74(31.24-21.31)	31.36(37.81-26.03)	2.25(1.86-2.72)	2.26(1.84-2.78)	0.32(0.21-0.42)	287.09(318.07-258.16)	468.73(520.85-422.78)	27.62(25-30.52)	23.12(20.9-25.6)	-0.58(-0.62–0.53)
		Female	15.2(18.59-12.53)	21.75(26.85-17.79)	2.59(2.12-3.17)	3.03(2.45-3.77)	0.88(0.76-1.01)	190.14(211.84-170.5)	321.73(360.13-288.54)	31.76(28.55-35.31)	28.84(25.91-32.23)	-0.25(-0.3–0.2)
		Male	10.54(13.04-8.46)	9.61(11.99-7.76)	1.91(1.53-2.37)	1.53(1.2-1.97)	-0.53(-0.62–0.44)	96.95(108.64-85.83)	147(167.22-129.97)	23.48(20.94-26.18)	16.96(15.07-19.19)	-1.17(-1.26–1.07)
	Severe anemia	Both	1.62(2.04-1.31)	1.49(1.86-1.21)	0.14(0.11-0.18)	0.1(0.08-0.13)	-0.88(-0.97–0.79)	22.92(25.95-20.31)	27.04(30.62-23.94)	2.23(1.99-2.51)	1.34(1.18-1.51)	-1.85(-1.95–1.75)
		Female	0.96(1.22-0.76)	0.98(1.24-0.77)	0.16(0.13-0.21)	0.13(0.1-0.17)	-0.51(-0.61–0.41)	14.41(16.47-12.58)	16.72(19.15-14.55)	2.42(2.11-2.75)	1.5(1.31-1.72)	-1.71(-1.8–1.61)
		Male	0.66(0.85-0.53)	0.52(0.68-0.41)	0.12(0.1-0.15)	0.08(0.06-0.1)	-1.39(-1.5–1.28)	8.51(9.83-7.41)	10.33(11.91-8.96)	2.12(1.85-2.42)	1.2(1.04-1.39)	-2.07(-2.19–1.96)
Low SDI	Anemia	Both	41.48(50.15-34.05)	121.24(146.13-99.29)	7.72(6.46-9.16)	10.34(8.65-12.22)	1.54(1.33-1.75)	180.83(200.62-161.61)	506.59(560.59-452.61)	75.84(67.96-84.34)	99.27(88.72-110.21)	1.01(0.97-1.05)
		Female	20.24(24.42-16.41)	63.21(76.7-51.03)	7.84(6.44-9.36)	10.95(8.97-13.11)	1.64(1.42-1.86)	94.43(104.96-84.39)	261.85(289.29-235.6)	75.78(67.75-84.07)	95.88(85.94-107.04)	0.86(0.83-0.9)
		Male	21.24(26.14-17.2)	58.03(70.97-47.35)	7.54(6.33-8.99)	9.67(8.11-11.48)	1.44(1.23-1.65)	86.4(96.98-76.08)	244.74(273-217.45)	75.79(67.19-84.96)	103.29(92.17-114.82)	1.18(1.14-1.23)
	Mild anemia	Both	13.61(16.09-11.3)	48.02(56.96-39.68)	2.86(2.4-3.37)	4.45(3.72-5.24)	2.2(1.94-2.45)	66.01(73.69-59.15)	214.2(237.87-192.17)	26.44(23.62-29.6)	40.13(36.07-44.57)	1.58(1.49-1.66)
		Female	6.24(7.5-5.09)	24.04(29.12-19.34)	2.54(2.09-3.01)	4.27(3.48-5.13)	2.34(2.1-2.58)	26.72(29.87-23.92)	84.87(94.07-76.01)	20.08(17.98-22.4)	29.23(26.12-32.45)	1.38(1.3-1.46)
		Male	7.37(8.76-6.13)	23.98(28.8-19.83)	3.15(2.65-3.75)	4.62(3.88-5.46)	2.09(1.82-2.36)	39.29(44.41-34.65)	129.33(144.5-115.04)	32.57(28.78-36.72)	51.7(46.01-57.85)	1.77(1.68-1.86)
	Moderate anemia	Both	24.03(29.49-19.53)	65.78(80.58-52.96)	4.21(3.51-5.02)	5.29(4.4-6.33)	1.25(1.07-1.44)	93.81(104.14-83.73)	250.83(279.06-223.82)	39.95(35.63-44.64)	50.3(44.88-56.04)	0.89(0.86-0.92)
		Female	12.13(14.76-9.79)	35.41(43.16-28.39)	4.6(3.8-5.51)	6.04(4.93-7.24)	1.41(1.2-1.62)	55.9(62.03-49.97)	152.89(170.29-137.03)	45.6(40.71-50.71)	57.16(51.08-63.85)	0.84(0.81-0.87)
		Male	11.9(14.98-9.32)	30.37(38.12-24.02)	3.78(3.09-4.59)	4.5(3.69-5.48)	1.05(0.88-1.22)	37.91(42.81-33.28)	97.93(109.86-85.98)	34.34(30.43-38.58)	43.35(38.24-48.56)	0.94(0.89-0.98)
	Severe anemia	Both	3.84(4.79-3.03)	7.44(9.22-5.99)	0.66(0.54-0.79)	0.6(0.49-0.72)	0.11(-0.05-0.27)	21.01(23.69-18.46)	41.56(46.98-36.62)	9.45(8.34-10.69)	8.83(7.81-10)	-0.26(-0.38–0.14)
		Female	1.87(2.34-1.47)	3.76(4.7-3.04)	0.7(0.57-0.86)	0.64(0.52-0.78)	0.17(-0.02-0.36)	11.81(13.33-10.33)	24.08(27.54-21.22)	10.1(8.84-11.4)	9.49(8.33-10.82)	-0.2(-0.28–0.12)
		Male	1.97(2.51-1.51)	3.68(4.66-2.83)	0.61(0.49-0.75)	0.54(0.44-0.67)	0.05(-0.09-0.19)	9.2(10.72-7.89)	17.48(20.2-14.95)	8.89(7.7-10.21)	8.25(7.14-9.48)	-0.31(-0.47–0.15)
Low-middle SDI	Anemia	Both	85.66(106.02-69.86)	170.49(206.95-140.93)	7.27(6.07-8.76)	9.39(7.82-11.27)	1.35(1.18-1.52)	589.68(656.07-526.87)	1612.87(1788.61-1449.51)	98.06(87.77-108.82)	121.02(108.75-134.17)	0.64(0.56-0.72)
		Female	41.29(52.23-33.33)	88.1(108.29-71.78)	7.09(5.8-8.75)	9.54(7.83-11.63)	1.4(1.25-1.55)	283.62(314.22-254.9)	773.3(860.3-691.54)	90.9(81.46-101.31)	109.12(97.86-121.5)	0.53(0.47-0.6)
		Male	44.37(54.62-35.76)	82.38(100.12-67.7)	7.41(6.19-8.94)	9.23(7.67-11.06)	1.33(1.14-1.52)	306.06(342.55-271.96)	839.57(928.54-754.58)	105.57(94.16-117.75)	135.39(121.77-150.12)	0.78(0.69-0.87)
	Mild anemia	Both	31.45(37.98-26.13)	80.76(96.5-67.27)	2.82(2.38-3.34)	4.43(3.73-5.26)	2.08(1.9-2.27)	223.92(249.74-200.37)	744.36(823.74-668.95)	35.81(32.03-39.93)	54.88(49.48-60.59)	1.48(1.43-1.53)
		Female	14.15(17.56-11.55)	38.69(47.55-31.62)	2.44(2.02-2.97)	4.14(3.4-5.05)	2.15(2.01-2.3)	82.16(91.6-73.39)	273.27(304.01-243.73)	25.1(22.43-27.94)	38.01(33.88-42.4)	1.45(1.42-1.47)
		Male	17.3(20.81-14.33)	42.07(49.99-35.21)	3.18(2.69-3.81)	4.74(3.99-5.61)	2.07(1.84-2.3)	141.75(159.65-125.73)	471.09(522.64-421.42)	46.49(41.38-52.08)	73.95(66.13-82.2)	1.61(1.55-1.67)
	Moderate anemia	Both	47.78(59.73-38.38)	82.83(102.07-67.53)	3.91(3.22-4.76)	4.57(3.76-5.57)	0.97(0.82-1.12)	305.58(340.62-272.43)	763(851.64-682.72)	51.52(46.02-57.39)	57.9(51.92-64.4)	0.29(0.19-0.39)
		Female	23.91(30.25-19.14)	45.74(56.47-37.07)	4.08(3.32-5.06)	4.99(4.08-6.11)	1.11(0.95-1.26)	168.38(187.15-150.77)	439.46(491.64-391.48)	54.62(48.99-60.88)	62.32(55.67-69.85)	0.34(0.26-0.42)
		Male	23.87(30.15-18.76)	37.1(46.67-29.69)	3.72(3.03-4.57)	4.13(3.34-5.12)	0.82(0.67-0.97)	137.2(154.2-121.02)	323.55(361.82-288.66)	48.69(43.21-54.76)	53.71(48.01-59.73)	0.24(0.11-0.37)
	Severe anemia	Both	6.43(8.35-5.06)	6.89(8.79-5.57)	0.54(0.44-0.68)	0.39(0.32-0.49)	-0.61(-0.79–0.42)	60.19(67.41-53.54)	105.52(118.78-94.13)	10.73(9.57-12.02)	8.24(7.37-9.24)	-1.11(-1.3–0.93)
		Female	3.22(4.29-2.48)	3.68(4.71-2.96)	0.57(0.45-0.74)	0.41(0.33-0.52)	-0.62(-0.82–0.42)	33.09(37.1-29.54)	60.58(68.19-54.07)	11.19(9.99-12.54)	8.8(7.87-9.89)	-0.98(-1.14–0.81)
		Male	3.2(4.2-2.48)	3.21(4.27-2.52)	0.51(0.42-0.64)	0.37(0.29-0.48)	-0.59(-0.77–0.41)	27.1(30.75-23.57)	44.94(51.19-39.45)	10.39(9.11-11.75)	7.73(6.83-8.75)	-1.29(-1.5–1.07)
Middle SDI	Anemia	Both	112.05(133.07-94.68)	186.11(221.96-159.74)	6.1(5.23-7.11)	7.83(6.68-9.32)	1.29(1.13-1.45)	1045.71(1168.11-928.1)	2854.16(3171.57-2557.21)	103.37(91.89-114.95)	118.4(106.73-131.35)	0.6(0.55-0.66)
		Female	59.66(71.52-50.2)	106.02(129.11-90.14)	6.38(5.41-7.58)	8.91(7.56-10.88)	1.57(1.4-1.74)	536.54(597.26-479.14)	1423.44(1585.78-1276.81)	98.16(87.22-108.99)	111.03(99.92-123.15)	0.61(0.53-0.69)
		Male	52.4(62.64-44.35)	80.1(93.84-69.01)	5.82(4.96-6.79)	6.77(5.8-7.96)	0.97(0.82-1.13)	509.17(573.16-447.99)	1430.73(1588.35-1279.98)	111.07(97.74-123.87)	128.71(115.71-142.57)	0.59(0.56-0.63)
	Mild anemia	Both	55.58(65.71-47.25)	113.15(134.33-97.1)	3.07(2.64-3.61)	4.65(4-5.56)	1.9(1.73-2.07)	540.13(604.03-477.72)	1774.42(1970.45-1592.8)	51.68(45.73-57.78)	72.82(65.65-80.75)	1.35(1.27-1.44)
		Female	28.18(34.07-23.76)	60.15(73.89-50.45)	2.97(2.53-3.54)	5.03(4.23-6.25)	2.21(2.05-2.37)	218.06(245.02-193.9)	720.7(803.21-644.88)	38.23(33.89-42.81)	55.89(50.28-62.12)	1.57(1.44-1.7)
		Male	27.41(32.25-23.36)	53(62.3-45.7)	3.17(2.74-3.69)	4.31(3.72-5.03)	1.61(1.42-1.8)	322.07(363.55-282.85)	1053.72(1175.68-942.72)	67.58(59.2-76.04)	93.12(83.9-103.53)	1.22(1.16-1.27)
	Moderate anemia	Both	52.48(62.77-44.06)	68.91(82.14-58.34)	2.81(2.39-3.32)	3(2.53-3.61)	0.62(0.47-0.76)	455.18(509.84-405.02)	995.93(1108.27-889.76)	46.24(41.23-51.52)	41.96(37.69-46.59)	-0.23(-0.27–0.19)
		Female	29.28(34.94-24.52)	43.46(52.48-36.85)	3.16(2.68-3.75)	3.68(3.09-4.47)	0.96(0.78-1.14)	287.13(320.03-255.76)	651.11(730.3-582.52)	53.78(47.9-59.98)	51.03(45.82-57.1)	-0.05(-0.11-0.02)
		Male	23.19(27.96-19.27)	25.44(30.85-21.28)	2.45(2.05-2.91)	2.31(1.91-2.83)	0.1(-0.01-0.21)	168.05(189.96-148.34)	344.83(385.88-305.9)	38.73(34.4-43.37)	32.44(28.94-36.2)	-0.56(-0.63–0.49)
	Severe anemia	Both	3.99(4.74-3.31)	4.06(4.86-3.44)	0.22(0.19-0.26)	0.18(0.15-0.21)	-0.21(-0.4–0.01)	50.4(56.79-44.7)	83.81(94.71-74.55)	5.45(4.84-6.14)	3.62(3.22-4.08)	-1.33(-1.4–1.27)
		Female	2.19(2.64-1.83)	2.41(2.87-2.01)	0.24(0.21-0.29)	0.2(0.17-0.24)	-0.05(-0.28-0.19)	31.35(35.45-27.6)	51.63(58.59-45.63)	6.16(5.41-7.03)	4.11(3.64-4.67)	-1.26(-1.31–1.21)
		Male	1.8(2.19-1.47)	1.65(2.03-1.35)	0.2(0.16-0.23)	0.15(0.12-0.18)	-0.44(-0.59–0.28)	19.05(21.76-16.64)	32.18(36.57-28.24)	4.76(4.2-5.42)	3.15(2.78-3.56)	-1.45(-1.55–1.35)
**Region**												
Andean Latin America	Anemia	Both	3.08(5.24-1.73)	6.99(11.53-4.16)	7.6(4.72-12.1)	10.74(6.48-17.5)	1.56(1.4-1.73)	14.18(15.93-12.59)	56.7(63.99-49.88)	67.56(59.85-75.89)	102.22(89.82-115.7)	1.38(1.26-1.49)
	Mild anemia	Both	1.4(2.35-0.83)	4.38(7.1-2.62)	3.62(2.34-5.6)	6.71(4.06-10.76)	2.62(2.4-2.83)	7.38(8.34-6.5)	36.91(41.8-32.65)	34.94(30.82-39.48)	66.33(58.59-75.15)	2.15(1.97-2.34)
	Moderate anemia	Both	1.55(2.69-0.85)	2.5(4.2-1.42)	3.68(2.17-6.04)	3.86(2.21-6.43)	0.37(0.24-0.51)	6.11(6.93-5.32)	18.31(20.92-15.8)	29.18(25.49-32.97)	33.18(28.48-37.9)	0.38(0.32-0.44)
	Severe anemia	Both	0.12(0.22-0.07)	0.11(0.18-0.07)	0.31(0.18-0.5)	0.17(0.11-0.27)	-1.67(-1.91–1.43)	0.7(0.82-0.59)	1.48(1.73-1.25)	3.44(2.92-4.04)	2.72(2.3-3.16)	-0.79(-0.88–0.69)
Australasia	Anemia	Both	1.43(2.17-0.98)	2.56(3.83-1.77)	6.68(4.5-10.31)	7.96(5.17-12.43)	0.99(0.75-1.24)	15.77(18.41-13.23)	32.13(39.53-25.81)	66.61(56.58-77.13)	61.01(49.15-74.53)	-0.37(-0.46–0.29)
	Mild anemia	Both	1.08(1.6-0.74)	2.03(3.03-1.39)	5.02(3.4-7.62)	6.24(4.03-9.68)	1.15(0.9-1.41)	11.76(13.83-9.82)	25.44(31-20.4)	49.33(41.76-57.41)	48.44(38.94-59)	-0.14(-0.21–0.07)
	Moderate anemia	Both	0.34(0.53-0.22)	0.51(0.79-0.34)	1.61(1.03-2.59)	1.68(1.02-2.71)	0.46(0.27-0.66)	3.84(4.84-2.96)	6.43(8.33-4.79)	16.53(13.01-20.67)	12.08(9.11-15.57)	-1.14(-1.31–0.98)
	Severe anemia	Both	0.01(0.02-0.01)	0.01(0.02-0.01)	0.05(0.03-0.07)	0.04(0.03-0.07)	0.12(-0.09-0.32)	0.17(0.23-0.13)	0.27(0.37-0.19)	0.74(0.56-0.97)	0.49(0.36-0.68)	-1.44(-1.65–1.22)
Caribbean	Anemia	Both	2.47(3.76-1.65)	4.71(6.98-3.31)	6.66(4.63-9.77)	10.09(6.96-15.15)	1.62(1.52-1.72)	32.35(36.38-28.61)	91.04(102.18-80.41)	123(108.99-138.76)	176.23(155.77-197.55)	1.15(1.06-1.25)
	Mild anemia	Both	1.29(1.91-0.89)	2.69(3.75-1.98)	3.55(2.57-5.06)	5.63(4.06-7.95)	1.84(1.69-1.99)	20.82(23.54-18.36)	63.5(71.42-56.12)	79.09(69.83-89.63)	122.92(108.81-138.42)	1.46(1.34-1.57)
	Moderate anemia	Both	1.12(1.73-0.72)	1.93(3.18-1.23)	2.95(1.94-4.46)	4.27(2.66-7.17)	1.38(1.33-1.44)	10.88(12.39-9.58)	26.24(29.8-22.95)	41.46(36.55-47.15)	50.78(44.48-57.61)	0.57(0.51-0.63)
	Severe anemia	Both	0.06(0.1-0.04)	0.09(0.16-0.05)	0.16(0.11-0.27)	0.2(0.11-0.37)	0.61(0.51-0.71)	0.65(0.75-0.56)	1.31(1.53-1.11)	2.45(2.12-2.83)	2.53(2.16-2.94)	-0.03(-0.1-0.03)
Central Asia	Anemia	Both	7.77(11.72-5.2)	14.32(21.26-10.05)	11(7.66-16.02)	14.93(10.51-22.18)	1.42(1.13-1.72)	38.91(44.34-34.29)	93.11(105.42-82.4)	78.5(69.28-88.83)	123.17(109.52-138.69)	1.76(1.58-1.93)
	Mild anemia	Both	3.6(5.2-2.47)	7.88(11.6-5.6)	5.22(3.68-7.27)	8.25(5.89-12.17)	2.15(1.82-2.48)	18.31(20.81-16.04)	50.56(57.42-44.81)	37.04(32.6-42.03)	67.28(59.75-75.71)	2.34(2.11-2.57)
	Moderate anemia	Both	3.9(6.17-2.49)	6.09(9.31-4.12)	5.39(3.62-8.25)	6.31(4.3-9.63)	0.74(0.47-1)	18.89(21.76-16.57)	39.75(45.21-34.84)	37.98(33.45-43)	52.2(45.99-58.8)	1.25(1.13-1.37)
	Severe anemia	Both	0.27(0.41-0.18)	0.35(0.52-0.25)	0.39(0.28-0.57)	0.36(0.26-0.54)	-0.03(-0.2-0.14)	1.71(2-1.47)	2.8(3.29-2.38)	3.48(2.99-4.02)	3.69(3.17-4.28)	0.19(0.16-0.22)
Central Europe	Anemia	Both	6.66(8.51-5.42)	8.42(10.35-6.77)	5.52(4.41-7.16)	7.56(5.98-9.63)	1.55(1.4-1.7)	59.62(67.45-52.15)	94.52(107.77-82.98)	41.1(36.2-46.18)	48.81(42.97-55.64)	0.52(0.48-0.56)
	Mild anemia	Both	4.37(5.51-3.56)	6.27(7.68-5.05)	3.56(2.86-4.57)	5.51(4.37-7)	2.03(1.84-2.21)	42.3(47.57-37.05)	72.54(82.32-63.88)	29.14(25.73-32.67)	37.22(32.9-42.34)	0.77(0.72-0.82)
	Moderate anemia	Both	2.18(2.83-1.73)	2.08(2.59-1.65)	1.87(1.46-2.47)	1.98(1.55-2.57)	0.52(0.42-0.62)	16.27(18.89-13.94)	20.91(24.31-17.9)	11.22(9.72-12.97)	11.05(9.45-12.83)	-0.13(-0.18–0.08)
	Severe anemia	Both	0.1(0.13-0.08)	0.08(0.1-0.06)	0.09(0.07-0.11)	0.07(0.05-0.09)	-0.44(-0.57–0.32)	1.06(1.27-0.89)	1.07(1.27-0.91)	0.73(0.62-0.87)	0.54(0.46-0.65)	-1.17(-1.25–1.08)
Central Latin America	Anemia	Both	10.34(14.3-7.81)	17.68(22.71-14.04)	5.48(4.23-7.29)	7.05(5.6-9.06)	0.96(0.89-1.04)	111.58(124.76-98.46)	368.87(408.67-333.47)	131.87(116.72-148.16)	158.16(143.14-175.29)	0.57(0.52-0.62)
	Mild anemia	Both	5.9(8.01-4.45)	11.72(15.06-9.38)	3.22(2.49-4.23)	4.63(3.7-5.95)	1.33(1.22-1.45)	68.2(76.28-60.17)	251.86(277.82-227.31)	78.85(69.85-88.57)	107.32(97.14-118.73)	0.96(0.87-1.05)
	Moderate anemia	Both	4.21(5.82-3.16)	5.71(7.47-4.48)	2.14(1.64-2.89)	2.33(1.82-3.05)	0.38(0.34-0.42)	39.77(44.55-35.01)	108.71(121.2-97.58)	48.35(42.67-54.31)	47.17(42.41-52.66)	-0.09(-0.11–0.07)
	Severe anemia	Both	0.23(0.3-0.17)	0.24(0.31-0.19)	0.12(0.09-0.16)	0.1(0.08-0.12)	-0.49(-0.62–0.37)	3.61(4.06-3.18)	8.3(9.24-7.43)	4.67(4.1-5.28)	3.66(3.27-4.08)	-0.72(-0.82–0.63)
Central Sub-Saharan Africa	Anemia	Both	3.35(6.1-1.79)	10.77(19.13-5.99)	5.62(3.43-9.48)	7.58(4.75-12.46)	1.35(1.14-1.56)	12.49(14.45-10.91)	38.48(44.01-33.75)	53.55(47.69-60.52)	73.31(65.06-82.41)	1.07(0.94-1.2)
	Mild anemia	Both	1.03(1.79-0.61)	4.41(7.51-2.63)	1.96(1.29-3.15)	3.42(2.25-5.4)	2.33(2.05-2.61)	4.86(5.64-4.19)	18.92(21.74-16.39)	20.16(17.48-23.23)	33.74(29.43-38.3)	1.86(1.63-2.09)
	Moderate anemia	Both	2.05(3.87-1.05)	5.89(10.83-3.05)	3.24(1.9-5.61)	3.86(2.29-6.71)	0.9(0.74-1.07)	6.77(7.91-5.87)	18(20.7-15.74)	29.58(26.05-33.73)	36.22(31.9-41.16)	0.66(0.59-0.73)
	Severe anemia	Both	0.28(0.56-0.13)	0.46(0.89-0.23)	0.42(0.22-0.79)	0.3(0.17-0.54)	-1.03(-1.23–0.84)	0.87(1.06-0.7)	1.56(1.9-1.29)	3.81(3.14-4.59)	3.35(2.76-4.08)	-0.56(-0.64–0.48)
East Asia	Anemia	Both	39.23(48.48-31.64)	32.54(39.02-26.89)	3.09(2.51-3.76)	2.14(1.76-2.59)	-0.92(-1.06–0.77)	686.62(772.27-609.6)	1257.38(1406.13-1119.81)	81.62(72.84-91.4)	62.83(56.2-70.14)	-0.54(-0.66–0.42)
	Mild anemia	Both	21.26(26.2-17.24)	23.96(28.69-19.82)	1.64(1.35-2)	1.52(1.26-1.83)	0.2(0.01-0.38)	374.3(425.13-330.18)	924.21(1043.45-821.69)	42.75(37.93-48.53)	45.77(41-51.33)	0.72(0.56-0.87)
	Moderate anemia	Both	16.85(20.97-13.48)	8.17(9.96-6.6)	1.36(1.09-1.68)	0.59(0.47-0.74)	-2.7(-2.8–2.6)	285.26(319.83-252.61)	316.67(364.56-276.5)	35.32(31.45-39.49)	16.21(14.2-18.66)	-2.51(-2.64–2.37)
	Severe anemia	Both	1.13(1.44-0.88)	0.41(0.52-0.32)	0.09(0.07-0.11)	0.03(0.02-0.04)	-3.92(-4.09–3.75)	27.07(31.75-22.98)	16.5(20.15-13.47)	3.55(3.03-4.16)	0.85(0.7-1.03)	-4.92(-5.2–4.63)
Eastern Europe	Anemia	Both	21.53(25.62-17.99)	35.15(42.17-29.21)	9.29(7.64-11.09)	15.29(12.49-18.54)	2.51(2.16-2.85)	176.09(200.15-154.05)	314.93(361.82-272.23)	64.08(56.4-72.34)	91.4(79.46-104.98)	1.34(1.26-1.42)
	Mild anemia	Both	14.24(16.8-11.93)	25.49(30.7-21.19)	6.14(5.08-7.29)	11.11(9.05-13.45)	2.96(2.58-3.36)	108.46(123.69-95.21)	212.8(245.15-183.55)	39.16(34.64-44.39)	62.1(54.06-71.24)	1.89(1.77-2.02)
	Moderate anemia	Both	6.92(8.4-5.69)	9.25(11.34-7.49)	2.99(2.42-3.68)	4.02(3.21-5)	1.54(1.29-1.8)	63.7(74.69-53.93)	97.04(115.11-80.94)	23.45(20.01-27.34)	27.84(23.29-32.79)	0.44(0.32-0.57)
	Severe anemia	Both	0.37(0.47-0.29)	0.41(0.54-0.31)	0.15(0.12-0.19)	0.17(0.12-0.22)	0.59(0.41-0.76)	3.93(4.91-3.15)	5.09(6.39-4.08)	1.46(1.19-1.78)	1.46(1.17-1.8)	-0.47(-0.72–0.23)
Eastern Sub-Saharan Africa	Anemia	Both	12.68(16.24-9.91)	37.49(48.39-29.62)	6.87(5.61-8.47)	9.36(7.62-11.6)	1.59(1.36-1.82)	42.53(47.74-37.7)	119.88(133.82-106.48)	56.4(49.93-63.44)	76.11(67.34-85.82)	1.1(1.01-1.2)
	Mild anemia	Both	4.43(5.47-3.58)	16.89(21.25-13.64)	2.79(2.31-3.37)	4.6(3.76-5.56)	2.47(2.17-2.76)	18.04(20.26-15.98)	59.93(67.05-52.88)	22.66(20-25.52)	36.21(31.94-40.66)	1.78(1.63-1.93)
	Moderate anemia	Both	7.22(9.46-5.52)	18.97(25.21-14.56)	3.6(2.88-4.52)	4.38(3.51-5.53)	1.08(0.89-1.27)	21.74(24.43-19.16)	54.94(61.96-48.59)	29.78(26.27-33.59)	36.51(32.21-41.45)	0.75(0.68-0.82)
	Severe anemia	Both	1.04(1.42-0.76)	1.62(2.17-1.23)	0.48(0.38-0.62)	0.38(0.3-0.48)	-0.72(-0.83–0.61)	2.75(3.11-2.43)	5.01(5.66-4.41)	3.96(3.51-4.48)	3.4(3-3.87)	-0.71(-0.77–0.64)
High-income Asia Pacific	Anemia	Both	12.02(15.93-9.13)	14.12(18.27-10.95)	6.5(4.96-8.57)	6.01(4.59-7.84)	0.33(0.11-0.55)	263.62(298.32-235.14)	590.06(686.75-515.42)	134.49(120.37-151.76)	118.84(104.32-137.74)	-0.57(-0.6–0.53)
	Mild anemia	Both	8.13(10.44-6.26)	10.9(14-8.49)	4.35(3.34-5.63)	4.55(3.51-5.9)	0.75(0.51-0.99)	178.86(201.34-157.14)	442.69(514.35-387.27)	90.29(79.79-101.29)	90.68(79.39-105.48)	-0.13(-0.19–0.07)
	Moderate anemia	Both	3.7(5.09-2.75)	3.12(4.08-2.32)	2.06(1.52-2.9)	1.42(1.05-1.89)	-0.7(-0.91–0.49)	81.1(94.01-70.02)	142.44(171.08-117.59)	42.28(36.32-48.74)	27.23(22.67-32.8)	-1.66(-1.75–1.56)
	Severe anemia	Both	0.18(0.28-0.12)	0.11(0.14-0.08)	0.1(0.07-0.16)	0.05(0.03-0.07)	-2.02(-2.34–1.69)	3.66(4.36-3.04)	4.93(6.3-3.84)	1.92(1.6-2.28)	0.93(0.73-1.18)	-2.6(-2.84–2.35)
High-income North America	Anemia	Both	25.01(29.66-21.11)	31.99(37.54-27.06)	8.45(7.1-10.11)	7.91(6.61-9.43)	0.07(-0.05-0.19)	307.85(354.29-268.44)	608.11(706.68-522.74)	84.1(73.52-96.43)	94.81(81.57-109.87)	0.9(0.68-1.12)
	Mild anemia	Both	19.41(23.02-16.37)	25.1(29.31-21.21)	6.52(5.47-7.8)	6.13(5.16-7.25)	0.08(-0.03-0.2)	237.57(273.91-208.13)	465.65(541.18-402.02)	64.94(57.01-74.35)	72.71(62.96-84.32)	0.79(0.59-0.98)
	Moderate anemia	Both	5.45(6.6-4.48)	6.67(8.09-5.52)	1.88(1.54-2.31)	1.72(1.4-2.14)	0(-0.14-0.13)	67.43(80.45-56.61)	136.46(167.31-110.29)	18.39(15.48-21.85)	21.18(17.12-26.04)	1.27(0.94-1.6)
	Severe anemia	Both	0.14(0.18-0.12)	0.22(0.28-0.17)	0.05(0.04-0.06)	0.05(0.04-0.07)	0.75(0.59-0.9)	2.85(3.46-2.31)	5.99(7.66-4.67)	0.77(0.62-0.93)	0.92(0.72-1.17)	1.52(1.18-1.87)
North Africa and Middle East	Anemia	Both	34.25(44.69-26.45)	88.8(113.55-70.17)	9.63(7.76-12.09)	14.52(11.63-18.4)	1.54(1.49-1.59)	248.86(278.29-221.45)	842.75(936.77-757.51)	150.19(134.17-167.77)	207.14(186.1-229.56)	1.16(1.05-1.28)
	Mild anemia	Both	17.37(22.08-13.65)	57.2(72.79-45.49)	5.23(4.26-6.44)	9.42(7.59-11.86)	2.15(2.1-2.21)	144.25(161.66-128.59)	589.9(660.19-526.16)	85.91(76.8-96.28)	144(128.3-160.75)	1.81(1.68-1.93)
	Moderate anemia	Both	15.94(21.31-11.89)	30.11(39.81-23.05)	4.14(3.25-5.41)	4.86(3.75-6.38)	0.66(0.61-0.72)	96.36(107.72-85.68)	239.76(267.5-213.92)	58.69(52.15-65.54)	59.74(53.36-66.55)	0.18(0.04-0.31)
	Severe anemia	Both	0.93(1.25-0.7)	1.49(2.1-1.06)	0.25(0.19-0.33)	0.24(0.17-0.34)	0.07(-0.06-0.19)	8.26(9.37-7.27)	13.09(14.9-11.52)	5.59(4.89-6.33)	3.4(2.98-3.9)	-1.59(-1.65–1.52)
Oceania	Anemia	Both	0.35(0.6-0.19)	0.81(1.57-0.42)	4.43(2.63-7.34)	5.37(2.96-10.05)	0.71(0.64-0.77)	3.31(3.78-2.89)	10.17(11.54-8.91)	108.84(96.11-123.19)	147.62(130.09-166.4)	0.94(0.89-0.98)
	Mild anemia	Both	0.13(0.21-0.08)	0.33(0.61-0.19)	1.74(1.15-2.67)	2.33(1.39-4.08)	1.07(1.02-1.12)	1.91(2.18-1.66)	6.12(6.98-5.32)	63.79(55.95-72.45)	90.27(78.76-103)	1.04(0.98-1.1)
	Moderate anemia	Both	0.2(0.37-0.11)	0.44(0.88-0.22)	2.46(1.36-4.35)	2.82(1.45-5.5)	0.48(0.4-0.57)	1.27(1.47-1.09)	3.72(4.25-3.21)	40.89(35.9-46.6)	52.61(46.06-59.93)	0.81(0.78-0.85)
	Severe anemia	Both	0.02(0.04-0.01)	0.03(0.07-0.02)	0.22(0.12-0.44)	0.22(0.1-0.46)	0.02(-0.13-0.17)	0.13(0.16-0.11)	0.33(0.41-0.27)	4.15(3.47-4.93)	4.74(3.9-5.7)	0.47(0.44-0.5)
South Asia	Anemia	Both	85.54(104.8-69.68)	160.99(196.32-132.81)	7.65(6.35-9.23)	8.72(7.27-10.52)	1.18(0.93-1.43)	618.71(692.62-549.79)	1755.26(1957.9-1565.23)	108.21(96.41-121.2)	128.22(114.72-142.85)	0.56(0.43-0.69)
	Mild anemia	Both	28.06(34.41-23.03)	67.82(82.98-56.06)	2.67(2.22-3.21)	3.62(3.03-4.4)	1.96(1.65-2.26)	190.75(214.9-169.96)	624.48(698.97-554.97)	30.51(27.12-34.25)	44.13(39.46-49.44)	1.42(1.32-1.52)
	Moderate anemia	Both	49.62(61.6-39.93)	84.58(102.94-69.27)	4.28(3.52-5.2)	4.62(3.82-5.58)	0.88(0.66-1.1)	343.53(383.28-304.63)	964.21(1076.78-857.67)	61.18(54.39-68.49)	71.36(63.63-79.38)	0.48(0.33-0.62)
	Severe anemia	Both	7.86(9.92-6.26)	8.59(10.74-6.98)	0.7(0.57-0.86)	0.48(0.4-0.6)	-0.6(-0.84–0.36)	84.43(94.84-74.77)	166.56(188.29-148.13)	16.52(14.6-18.57)	12.73(11.37-14.35)	-1.12(-1.34–0.89)
Southeast Asia	Anemia	Both	34.25(44.58-26.35)	48.61(61.13-39.71)	6.53(5.12-8.33)	7.22(5.87-9.16)	0.74(0.47-1.02)	280.13(313.79-250.14)	945.88(1058.95-843.75)	108.92(96.9-121.75)	163(145.19-181.59)	1.36(1.33-1.39)
	Mild anemia	Both	16.1(20.88-12.49)	29.59(36.24-24.42)	3.16(2.5-4.01)	4.32(3.57-5.29)	1.6(1.27-1.94)	136.91(153.48-121.63)	571.36(640-508.97)	50.74(44.98-56.91)	96.36(85.95-107.68)	2.25(2.19-2.31)
	Moderate anemia	Both	17.13(22.65-12.89)	18.27(24.24-14.41)	3.17(2.43-4.16)	2.8(2.17-3.75)	-0.21(-0.43-0.01)	131.62(147.87-116.83)	354.64(401.59-312.79)	53.24(47.19-59.71)	63.05(55.68-71.41)	0.49(0.45-0.52)
	Severe anemia	Both	1.01(1.36-0.75)	0.75(0.98-0.6)	0.19(0.15-0.25)	0.11(0.09-0.15)	-1.56(-1.76–1.37)	11.6(13.19-10.13)	19.87(23.12-17.14)	4.94(4.34-5.65)	3.6(3.12-4.19)	-1.19(-1.25–1.12)
Southern Latin America	Anemia	Both	3.89(6.31-2.43)	5.03(8.27-3.19)	7.88(5-12.61)	7.38(4.56-12.61)	0.18(-0.04-0.4)	35.13(40.08-30.44)	71.71(82.84-62.6)	77.93(67.89-88.37)	84.5(73.89-97.63)	0.24(0.18-0.3)
	Mild anemia	Both	2.6(4.16-1.68)	3.75(5.99-2.43)	5.29(3.46-8.38)	5.44(3.42-9.02)	0.5(0.25-0.76)	23.53(27.1-20.4)	52.75(60.71-46.16)	51.44(44.92-58.86)	62.31(54.58-71.75)	0.62(0.53-0.7)
	Moderate anemia	Both	1.24(2.19-0.71)	1.24(2.17-0.74)	2.48(1.46-4.3)	1.89(1.08-3.38)	-0.59(-0.75–0.44)	10.88(12.74-9.08)	17.9(21.71-14.71)	24.77(20.78-28.74)	20.95(17.32-25.36)	-0.61(-0.63–0.59)
	Severe anemia	Both	0.05(0.09-0.03)	0.04(0.07-0.03)	0.11(0.07-0.17)	0.06(0.04-0.1)	-1.42(-1.61–1.22)	0.73(0.9-0.58)	1.07(1.35-0.84)	1.72(1.37-2.1)	1.24(0.98-1.57)	-1.12(-1.15–1.09)
Southern Sub-Saharan Africa	Anemia	Both	4.82(6.8-3.55)	8.81(12.73-6.6)	9.06(7.04-12.22)	10.94(8.3-15.75)	0.85(0.61-1.1)	24.22(27.27-21.52)	62.02(69.34-55.51)	87.59(77.47-98.98)	114.57(102.61-128.12)	0.82(0.63-1.02)
	Mild anemia	Both	2.46(3.41-1.83)	4.99(6.91-3.79)	4.83(3.75-6.39)	6.24(4.79-8.52)	1.14(0.83-1.44)	13.47(15.25-11.85)	37.25(41.55-33.22)	48.75(42.5-55.13)	68.51(60.93-76.32)	1.05(0.76-1.35)
	Moderate anemia	Both	2.2(3.28-1.57)	3.58(5.51-2.58)	3.92(2.93-5.53)	4.4(3.21-6.7)	0.54(0.36-0.72)	9.81(11.12-8.6)	22.82(25.99-20.16)	35.44(31.27-40.34)	42.43(37.5-48.45)	0.54(0.46-0.62)
	Severe anemia	Both	0.17(0.23-0.12)	0.24(0.34-0.18)	0.32(0.24-0.43)	0.3(0.23-0.41)	0.03(-0.04-0.1)	0.95(1.13-0.79)	1.96(2.3-1.67)	3.4(2.85-4.04)	3.63(3.09-4.27)	0.2(0.06-0.35)
Tropical Latin America	Anemia	Both	13.06(17.28-10.05)	30.1(37.98-23.76)	8.37(6.59-10.75)	12.97(10.16-16.46)	1.31(1.08-1.54)	102.39(116.5-89.53)	275.79(317.87-237.81)	111.44(97.57-126.73)	114.35(99.01-131.62)	-0.09(-0.16–0.02)
	Mild anemia	Both	6.38(8.28-4.93)	17.53(21.86-14.02)	4.23(3.34-5.33)	7.44(5.91-9.32)	1.66(1.35-1.98)	57.4(65.82-49.66)	172.75(199.99-148.61)	61.82(53.39-70.76)	71.44(61.69-82.5)	0.3(0.23-0.38)
	Moderate anemia	Both	6.18(8.56-4.61)	11.88(15.31-9.09)	3.82(2.94-5.13)	5.23(3.96-6.81)	0.97(0.82-1.12)	41.22(47.65-35.27)	96.05(113.21-80.69)	45.36(38.86-52.49)	39.96(33.6-47.2)	-0.59(-0.66–0.53)
	Severe anemia	Both	0.5(0.73-0.34)	0.68(0.94-0.5)	0.31(0.22-0.44)	0.29(0.21-0.41)	-0.29(-0.42–0.17)	3.78(4.76-3)	6.99(9.07-5.36)	4.26(3.4-5.37)	2.95(2.25-3.8)	-1.39(-1.45–1.33)
Western Europe	Anemia	Both	15.21(18.51-12.63)	18.85(22.88-15.66)	3.72(3.09-4.62)	3.76(3.08-4.62)	0.59(0.32-0.88)	308.38(351.28-272.95)	398.07(453.93-352.58)	51.23(45.64-57.99)	39.79(35.23-45.5)	-1.01(-1.08–0.94)
	Mild anemia	Both	12.17(14.81-10.11)	15.68(19.08-12.99)	2.93(2.43-3.62)	3.07(2.53-3.77)	0.75(0.45-1.04)	238.27(271.48-210.57)	327.08(375.01-289.28)	39.65(35.14-45.01)	32.89(29.03-37.57)	-0.78(-0.84–0.73)
	Moderate anemia	Both	2.96(3.66-2.43)	3.1(3.8-2.55)	0.77(0.62-0.99)	0.67(0.53-0.84)	-0.02(-0.25-0.2)	67.2(77.71-58.76)	68.49(79.17-59.07)	11.1(9.79-12.7)	6.67(5.81-7.64)	-1.91(-2.04–1.78)
	Severe anemia	Both	0.07(0.09-0.06)	0.07(0.09-0.06)	0.02(0.01-0.02)	0.01(0.01-0.02)	-0.36(-0.62–0.1)	2.91(3.44-2.45)	2.5(3.01-2.09)	0.48(0.41-0.56)	0.23(0.2-0.28)	-2.59(-2.75–2.42)
Western Sub-Saharan Africa	Anemia	Both	22.45(27.17-18.15)	76.51(92.44-62.5)	11.65(9.62-13.77)	16.62(13.8-19.68)	1.67(1.51-1.83)	72.69(80.91-65.08)	216.2(240.15-193.53)	83.47(74.68-93.19)	117.37(104.51-130.71)	1.3(1.21-1.4)
	Mild anemia	Both	8.81(10.5-7.21)	32.4(39.08-26.55)	5.26(4.37-6.21)	7.88(6.57-9.36)	1.91(1.73-2.1)	37.2(41.44-33.01)	119.65(132.89-107.1)	41.88(37.25-47.09)	64.8(57.73-72.44)	1.66(1.52-1.79)
	Moderate anemia	Both	11.87(14.63-9.43)	39.77(48.64-32.02)	5.67(4.61-6.81)	7.97(6.56-9.57)	1.59(1.44-1.73)	33.06(37.22-29.32)	90.96(101.59-80.79)	38.74(34.32-43.59)	49.56(43.7-55.38)	0.97(0.89-1.04)
	Severe anemia	Both	1.77(2.24-1.39)	4.34(5.56-3.39)	0.72(0.58-0.9)	0.77(0.61-0.96)	0.48(0.34-0.63)	2.44(2.77-2.14)	5.58(6.39-4.87)	2.85(2.49-3.27)	3.01(2.61-3.49)	0.26(0.16-0.36)

ASPR, age-standardized prevalence rate; EAPC, estimated annual percentage change; UI, uncertainty interval.

ASPR of CKD-T1DM-related anemia decreased only in East Asia (EAPC = −0.92, 95% CI: −1.06–−0.77), but increased the fastest in Eastern Europe (EAPC = 2.51, 95% CI: 2.16–2.85, **Table 3**). As for CKD-T2DM, it increased most rapidly in Central Asia (EAPC = 1.76, 95% CI: 1.58–1.93). YLDs rate of CKD-T2DM-related anemia increased only in low SDI quintile (EAPC = 0.50, 95% CI: 0.46–0.54), and it increased the fastest in High-income North America (EAPC = 1.20, 95% CI: 0.90–1.50, **Table S7**).

**Figure 5:**
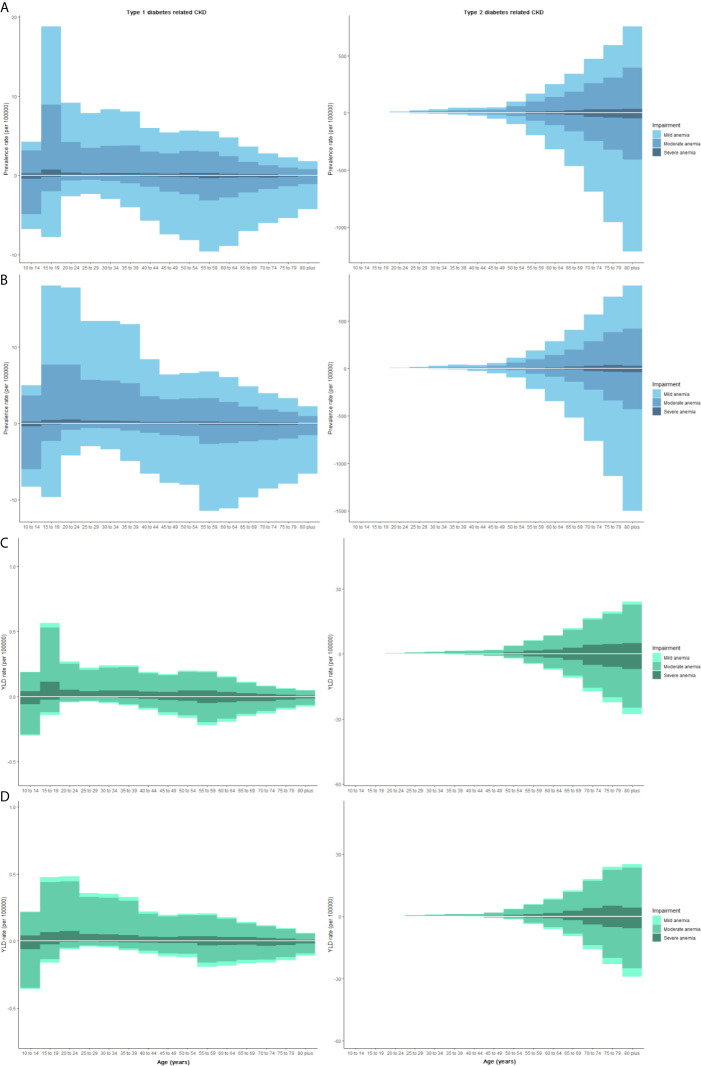
The prevalence and YLD rate of CKD-DM-related anemia at various age subgroups by gender. Each column represents the prevalence or YLD rate (per 100,000 people) of CKD-DM-related anemia (Three grades: mild, moderate, and severe). The upper column in each age-group is data for females, and the below column is for males. **(A)** Prevalence in 1990; **(B)** Prevalence in 2019; **(C)** YLD rate in 1990; **(D)** YLD rate in 2019. CKD, chronic kidney disease; YLD, years lived with disability.

The prevalence and YLD rate of CKD-T2DM and CKD-T1DM was different among sex and age (**Figure 5**). The main onset age of CKD-T1DM-related anemia changed from 15–19 years in 1990 to 15–39 years for females. But that for males was stable, with two peaks at 15–19 and 55–59 years. The YLD rate of CKD-T1DM-related anemia was higher in females aged 15–24 years and in males aged 10–14 years. As for CKD-T2DM-related anemia, the prevalence and YLD rate increased with age.

## Discussion

This study investigated the global, regional, and national disease burden of CKD-DM. Globally in 2019, there were 2.6 million incident cases, 135 million patients, 0.5 million deaths, and 13 million DALYs of CKD-DM, with a large increment as the global population grew. Further analysis showed that CKD-T2DM accounted for 95.32, 96.27, 83.20, and 75.38% of total CKD-DM incident cases, patients, deaths, and DALYs, respectively, reflecting the key role of type 2 diabetes in CKD development (20). All ASRs of CKD-T2DM increased from 1990 to 2019. All measured rates of CKD-T2DM increased with age, mirroring the cumulative risk effects of age. From the age of 50, all rates were higher in males than females. Interestingly, ASIR and ASPR of CKD-T1DM increased globally, whereas ASDR and age-standardized DALY rate decreased for women but increased for men. As for the sex difference, sex hormones had a vital role in the development of diabetes and renal complications (21). In a previous study, racial differences were observed between women and men in diabetes, and the relationship between life course and diabetes was peculiar to women (22).

In High-income North America, Eastern Europe, North Africa, and Middle East, ASIR of CKD-T1DM kept higher than other regions. One study reported that the incidence of ESRD in diabetic patients was 10 times higher than non-diabetic patients. In Australia, one of the high-income countries, diabetes had become the leading cause of ESRD over the past 20 years (23). The mortality in ESRD patients was 18.3 times higher than the general population (24). Nevertheless, not all patients with CKD-DM could receive renal replacement therapy, and 78% of patients lived in low- and middle-income countries, where resources, availability of dialysis, and kidney transplants were limited (25). However, the difference was not fully attributed to medical convenience. ASIR and ASPR of CKD-T1DM increased only in Eastern Europe, with the lowest ASDR of CKD-T2DM. ASIR of CKD-T1DM decreased only in High-income North America, but ASDR and DALY rate increased faster there. ASDR and DALY rate decreased faster in High-income Asia Pacific and East Asia. The difference of CKD-DM between regions might result from the gap in genetic, ethnic, and dietary risk factors.

Patients with CKD-T1DM- and CKD-T2DM-related anemia had doubled over the past 30 years. Anemia is a common complication of CKD. Among all causes of anemia, malaria, schistosomiasis, and CKD-related anemia have been on the rise (26). However, the severity and type of anemia were various among regions. The higher the SDI value, the lower the increasing rate of anemia-related ASPR, mirroring the gap in life and medical convenience among different SDI quintiles. Furthermore, there was 40% of the population with anemia in Ghana (27), a country in Western Sub-Saharan Africa, resulting from iron deficiency, hemoglobinopathies, micronutrient deficiency, and inflammation (26, 28). In addition, we should attach importance to the fast increase of ASPR in Central Asia. Conversely, in Western Europe, High-income Asia Pacific, and East Asia, ASPR of CKD-T2DM-related anemia decreased sharply, and the reasons should be further evaluated. In Austria, the incidence of type 1 diabetes is increasing in children aged 5 to 14 years (29). Alarmingly, for CKD-T1DM-related anemia, patients aged of 10–14 years mainly suffered moderate anemia, more severe than other age-groups, which we should pay attention to.

In many cases, the burden of CKD-DM is determined by various factors, which caused gaps in the CKD prevention and management capabilities worldwide (30). Our results reflected a shift of CKD-T1DM burden from high to low SDI quintile, but the ASDR and DALY rate of CKD-DM increased faster in high SDI quintile, which was not fully attributed to medical environment and renal replacement therapies (31). Global burden of CKD-DM was concentrated in middle SDI quintiles, especially in developing countries (20). Additionally, ASIR of CKD-DM increased with SDI value, revealing racial differences in disease susceptibility and medical disparities (32, 33). The variation in CKD-DM epidemiology reflects huge regional inequities in preventive care (34). White European individuals were reported to have a higher prevalence of CKD-T1DM (35). Race influenced mortality in patients with type 2 diabetes and multiple chronic conditions (36). Some studies explained it by economic inequality, socioeconomic status, and segregation (37–39).

Understanding the burden of CKD-DM in various countries benefited equal kidney health. China, India, and the USA carrying high disease burden for CKD-DM might partly be owing to their high populations. Notably, China had the lowest ASIR of CKD-T1DM. ASPR of CKD-T1DM was higher in Russia, Canada, and Mongolia. This was partially attributed to high prevalence of type 2 diabetes, improvements on CKD screening (40), and the relatively stagnant progress in addressing CKD-DM burden.

Although aging and population growth contributed to the increased burden of CKD-DM, risk factors such as diet and metabolism were involved. A study on children stated that type 1 diabetes was associated with younger age at ESRD onset, whereas type 2 diabetes was related to a higher mortality rate (41). The presence of diabetic nephropathy was associated with age, duration of diabetes, and poor glycemic control (42).

Almost one in five CKDs was caused by diabetes (10). Moreover, less than half of the patients were tested for urinary albumin, an early marker of kidney disease caused by diabetes (43). Many countries still lack a well-trained team of kidney experts and universal access to primary health care and renal replacement therapy. Screening for kidney function in diabetic patients as well as raising awareness are necessary for the early detection of CKD. Reducing the burden of CKD-DM should be reflected in the government’s health priorities and resource allocation measures, focusing on prevention, early control, and delayed progress.

Some inevitable limitations should be taken into consideration in the interpretation of our findings. The GBD study estimated the burden of CKD by relying on statistical methods and predicted covariant values. GBD data come from census, disease registration, household survey, health service usage, air pollution monitoring, disease notification civil registration and vital statistics, and other sources. High-quality results were based on well-established medical registration systems in some countries, such as China, USA, India, Australia, UK, Russia, and so on. GBD 2019 location hierarchy includes all WHO member states. Large, high-quality, population-based studies of CKD are scarce in some countries or territories, such as Cook Islands, Niue, Vatican City, Liechtenstein, Order of Malta, Palestine. There was inevitable information bias of primary data in those districts. Therefore, when specific data were applied to countries or territories that are not members of the World Health Organization, and areas with underdeveloped medical systems, the findings need to be interpreted with caution. Due to the limited data, we cannot further investigate the burden of CKD-DM at different stages. A greater investment is still needed to improve vital registration and data collection in developing countries. Despite these limitations, the findings from this analysis add novel knowledge on the global burden of CKD-DM.

## Conclusion

From 1990 to 2019, the increasing burden of CKD-DM varied among regions and countries. All ASRs of CKD-T2DM exhibited upward trends, and from the age of 50, all rates were higher in males than females. ASIR of CKD-DM increased with SDI value. Middle SDI quintile accounted for the majority burden of CKD-DM worldwide. Asia carried the heaviest burden of CKD-DM, especially in South and East. The three countries with the highest burden of CKD-DM were China, the United States, and India. CKD-T2DM patients with anemia were mainly in mild to moderate grade for females, and in mild grade for males. Anemia-related YLD was mainly in moderate grade. These findings could help guide the epidemiological monitoring of this disease and prioritize the most appropriate health interventions.

## Data Availability Statement

The original contributions presented in the study are included in the article/**Supplementary Material**. Further inquiries can be directed to the corresponding author.

## Ethics Statement

The studies involving human participants were reviewed and approved by Ethics Committee of the Second Affiliated Hospital, College of Medicine, Xi’an Jiaotong University. Written informed consent from the participants’ legal guardian/next of kin was not required to participate in this study in accordance with the national legislation and the institutional requirements.

## Author Contributions

All authors contributed to the article and approved the submitted version. ZD, JG, and YD designed the study. YD, YW, MW, and SY conducted the initial searches. YiZ and XD collected the data and verified the accuracy of the data. DX and YuZ contributed to data interpretation. YD, ZZ, and DZ performed the statistical analysis and visualization. YD wrote and revised the manuscript.

## Conflict of Interest

The authors declare that the research was conducted in the absence of any commercial or financial relationships that could be construed as a potential conflict of interest.

## Abbreviations

ASIR, age-standardized incidence rate; ASPR, age-standardized prevalence rate; ASDR, age-standardized death rate; CKD-DM, chronic kidney disease caused by diabetes; DALY, disability adjusted life-year; EAPC, estimated annual percentage change; SDI, socio-demographic index; UI, uncertainty interval; YLD, years lived with disability.
