# Supplementary material for: Global, Regional, and National Burden of Diabetes-Related Chronic Kidney Disease From 1990 to 2019
Source: Front Endocrinol (Lausanne). 2021 Jul 1;12:672350. doi: 10.3389/fendo.2021.672350 (PMC8281340; doi:10.3389/fendo.2021.672350)

Supplementary Material

Appendix 2
Table S1. The global and regional burden of diabetes mellitus type 1 related chronic kidney disease. 4
Table S2. The age standardized rates and their variation trends of diabetes mellitus type 1 related chronic kidney disease. 7
Table S3. The incidence, prevalence, deaths, DALYs and their change percent of type 1 diabetes related CKD in 204 countries and territories. 10
Table S4. The age-standardized rates and their EAPCs of type 1 diabetes related CKD in 204 countries and territories. 26
Table S5. The incidence, prevalence, deaths, DALYs and their change percent of type 2 diabetes related CKD in 204 countries and territories. 41
Table S6. The age-standardized rates and their EAPCs of type 2 diabetes related CKD in 204 countries and territories. 59
Table S7. The YLDs and their ASRs of impairment caused by diabetes mellitus related chronic kidney disease. 74
Figure S1. Ranking the prevalent cases, deaths and DALYs of non-communicable diseases per 100,000 worldwide. 83
Figure S2. Ranking the prevalent cases, deaths and DALYs of chronic kidney disease per 100,000 worldwide. 84
Figure S3. The number of patients with type 2 diabetes related CKD over 30 years. 85
Figure S4. The number of type 2 diabetes related CKD deaths over 30 years. 86
Figure S5. The number of type 2 diabetes related CKD DALYs over 30 years. 87

Appendix

GBD Overview

The Global Burden of Disease (GBD) is an approach to global descriptive epidemiology. It is a systematic, scientific effort to quantify the comparative magnitude of health loss due to diseases, injuries, and risk factors by age, sex, and geographies for specific points in time. IHME serves as the coordinating center for the GBD and affiliated projects.
Incidence data is obtained from individual cancer registries or aggregated databases of cancer registries, such as CI5 (cancer incidence of five continents), SEER, EUERG or NORDCAN. GBD study relies on a lot of data – over 90,000 data sources. GBD produces regular estimates of all-cause mortality, deaths by cause, years of life lost due to premature mortality (YLLs), years lived with disability (YLDs), and disability-adjusted life years (DALYs) for a cause list. The cause list is agreed upon annually by the Scientific Council. The critical milestones for ongoing estimation include regular updates to the GBD estimates, referred to as the "GBD round." For each round, the entire time series back to 1990 is re-estimated using all available data to ensure the most complete and highly comparable set of estimates possible. Previous results will be archived every time new results are released. The GBD provides cutting-edge and timely results through scientific papers, policy reports, web content, and interactive visualizations.
We estimated numbers and rates of incidence, prevalence, deaths, and disability-adjusted life-years (DALYs) for the years 1990–2019.
GBD 2019 provides an independent estimation of population, for each of 204 countries and territories and the globe, using a standardized, replicable approach, as well as a comprehensive update on fertility. GBD 2019 incorporates major data additions and improvements, and methodological refinements. Mortality and life expectancy estimates have been extended back to 1950, and new causes have been added to the fatal and non-fatal cause lists, for a total of 369 diseases and injuries (http://ghdx.healthdata.org/GBD-resultstool).
Results from GBD 2019 are available through an interactive data downloading tool on the Global Health Data Exchange (GHDx). The GHDx is the world's most comprehensive catalogue of surveys, censuses, vital statistics, and other health-related data. Results are measured in terabytes.

Definition of indicator

The GBD cause list is organized in a hierarchy. Levels 1 represent all garbage codes for which a Level 1 GBD cause cannot be directly assigned. Level 2 represent all garbage codes that can be assigned to Level 1 causes in the GBD cause list. Level 3 includes includes all garbage codes for which we know the Level 2 CoD and can redistribute onto Level 3 causes. Level 4 includes all garbage codes for underlying causes of death that can be redistributed within a Level 3 cause. In this publication, estimates for the GBD cancer groups, for both sexes, for the time from 1980 to 2019, and for the 5-year GBD age groups (0-5; 5-9; etc. until 95+) are presented for 204 countries or territories.
Chronic Kidney Disease Due to Type 1 was defined as diabetes mellitus cases of Type 1 Diabetes Mellitus that experience Chronic Kidney Disease. Chronic Kidney Disease Due to Type 2 was defined as diabetes mellitus cases of Type 1 Diabetes Mellitus that experience Chronic Kidney Disease. Diabetes mellitus (DM) was defined as fasting plasma glucose (FPG) > 126 mg/dL (7mmol/L) or being on treatment for diabetes. GBD 2019 study estimated deaths caused by five subtypes of chronic kidney disease: type 1 diabetes, type 2 diabetes, hypertension, glomerulonephritis, and other causes.

Data analysis

This study complies with GATHER recommendations. ASRs were calculated on the basis of the following formula:

$$ASR = \frac{\sum_{i=1}^{A} a_i w_i}{\sum_{i=1}^{A} w_i} \times 100{,}000$$

The ASR (per 100,000 population) is equal to the sum of the product of the specific age ratio (ai) in age group i and the number (or weight) (wi) of the selected reference standard population group i divided by the sum of number (or weight) of the standard population, i.e.

Meanwhile, EAPC, which is approximately equal to the annual change for a specified range, was calculated using the following regression model to assess the trends in ASR:
$Y=\alpha+\beta X+\varepsilon$, where y refers to ln(ASR), x represents calendar year, ε means error term, and β determines the positive or negative trends in ASR. The EAPC could be given by 100*(exp(β)-1), as well as its 95% confidence interval (CI). If both the EAPC estimates and their lower limits of 95% CIs are > 0, ASR is in an upward trend. By contrast, if both EAPC estimates and their upper limits of 95% CIs are < 0, ASR is in a downward trend. Otherwise, ASR is stable.

Data sources

Cancer incidence data sources

Cancer incidence was sought from individual cancer registries or aggregated databases of cancer registry data like "Cancer Incidence In Five Continents" (CI5)," EUREG, or NORDCAN. Data were excluded if they were not representative of the coverage population (e.g., hospital-based registries), if they did not cover all malignant neoplasms as defined in ICD8, ICD-9 or ICD10, if they did not include data for both sexes and all age groups, if the data were limited to years prior to 1980, or if the source did not provide details on the population covered. Preference was given to registries with national coverage over those with only local coverage, except those from countries where the GBD study provides subnational estimates. ICD3-5 included 2,525 country-years of detailed data from 1980 to 2018 by country, year, sex, and age groups. Additional metadata for each source is available in the online GBD citation tool, http://ghdx.healthdata.org/gbd-2019/data-input-sources.

Cancer mortality data sources

A detailed description of the data sources and processing steps for the cause of death database can be found in the appendix to the GBD 2019 paper "Global burden of 369 diseases and injuries in 204 countries and territories, 1990–2019: a systematic analysis for the Global Burden of Disease Study 2019."

Incidence estimation

GBD cancer incidence estimates were generated by dividing final mortality estimates (after CoDCorrect adjustment) by the MI ratio for the specific cancer. To propagate uncertainty from the MI ratios and the mortality estimates to incidence, this process was done at the 1,000-draw level. It was assumed that uncertainty in the MI ratio is independent of uncertainty in the estimated age-specific death rates.

Bias of categories of input data

Bias of the input data included for the COD database is described elsewhere. Cancer registry data can be biased in multiple ways. A high proportion of ill-defined cancer cases in the registry data requires redistribution of these cases to other cancers, which introduces a potential for bias. Changes between coding systems can lead to artificial differences in disease estimates; however, we adjust for this bias by mapping the different coding systems to the GBD causes. Since many cancer registries are located in urban areas, the representativeness of the registry for the general population can also be problematic. The accuracy of mortality data reported in cancer registries usually depends on the quality of the vital registration system. If the vital registration system is incomplete or of poor quality, the mortality-to-incidence ratio can be biased to lower ratios.

Input data

To estimate the prevalence of each of these categories for all locations, by age, year, and sex, the prevalence of these neoplasms from hospital data was used as input for a prevalence model in DisMod-MR 2.1. These inputs included MarketScan claims data from the United States in the years 2000, 2010, and 2012, as well as hospital and outpatient data from other health systems worldwide. Each of these data sources were crosswalked to the 2012 MarketScan data. To estimate inpatient admission rates for newborns, GBD input estimates of the in-facility delivery (IFD) rates for every subnational and national location at 5-year intervals starting at 1990 and including the most recent 2019 estimate. IFD was estimated by using an ST-GPR model based on populationrepresentative surveys and administrative data. GBD accepted data sources from 28,646 location-years (1413 from administrative records and 27,233 from population surveys).

Socio-demographic index (SDI)

SDI, ranging from 0 to 1, is a comprehensive measure of development and is an indicator of the overall fertility rate of women under 25 years of age, educational attainment and lagging per capita income distribution in a country. For GBD 2019, after calculating SDI, values were multiplied by 100 for a scale of 0 to 100. Based on SDI values in 2019, countries and territories were classified into five categories.

Estimated annual percentage change (EAPCs)

We calculated estimated annual percentage change (EAPCs) of age-standardized rates (ASRs). The EAPC describes the ASR trends within a specified time interval. The natural logarithm of ASR is assumed to be linear along with time; that is, $Y=\alpha+\beta X+\varepsilon$, where Y refers to ln (ASR); X, the calendar year; and ε, the error term. Based on this formula, β represents the positive or negative ASR trends. The EAPC was calculated as $EAPC=100\times(\exp(\beta)-1)$. Its 95% confidence intervals (CI) could be obtained from the linear model. When the EAPC and lower CI limit are positive, ASR shows an upward trend. Conversely, when the EAPC and upper CI limit are negative, ASR shows a descending trend. In addition, we evaluated the relationship between SDI and ASRs in the different regions to define the potential factors that affect ASRs.

Table S1. The global and regional burden of diabetes mellitus type 1 related chronic kidney disease.

Location	Sex	Incident cases (No. ×1000) (95%UI)		Prevalent cases (No. ×1000) (95%UI)		Deaths (No. ×1000) (95%UI)		DALYs (No. ×1000) (95%UI)	
		1990	2019	1990	2019	1990	2019	1990	2019
Global	Both	70.17(62.37-78.39)	122.87(110.25-136.58)	2666.92(2419.73-2967.45)	5024.74(4533.86-5628.36)	43.21(30.13-58.88)	81.98(54.69-114.97)	1866.95(1353.97-2463.9)	3222.95(2280.07-4373.19)
	Female	26.47(23.29-29.83)	45.67(40.69-51.41)	1340.56(1208.32-1505.32)	2642.11(2351.54-2988.69)	19.92(14.16-26.95)	35.95(24.37-50.06)	857.98(629.26-1132.82)	1392.13(987.83-1887.54)
	Male	43.7(38.85-48.96)	77.2(69.02-85.49)	1326.36(1200.91-1472.6)	2382.63(2160.79-2641.28)	23.29(15.85-31.66)	46.03(30.55-65.66)	1008.98(724.16-1340.25)	1830.81(1271.89-2509)
**Socio-demographic index**									
High SDI	Both	13.47(11.85-15.34)	22.66(20.28-25.43)	672.74(598.99-756.29)	977.09(875.76-1081.12)	3.16(2.12-4.56)	6.09(3.99-9.18)	133.82(94.3-178.71)	225.65(154.43-308.27)
	Female	5.05(4.37-5.8)	8.22(7.15-9.42)	359.36(315.27-409.74)	513.29(455.86-572.82)	1.47(0.98-2.17)	2.66(1.74-4.08)	59.02(41.87-79.51)	94.52(65.23-128.58)
	Male	8.41(7.33-9.67)	14.44(12.85-16.36)	313.37(279.6-352.35)	463.81(417.32-513.82)	1.69(1.13-2.41)	3.43(2.22-5.09)	74.8(52.28-100.18)	131.13(88.25-181.1)
High-middle SDI	Both	13.4(11.44-15.81)	21.87(19.05-25.36)	607.47(541.39-682.53)	1073.7(950.24-1214.6)	7.89(5.59-10.75)	10.78(7.31-15.32)	332.97(244.15-438.85)	416.19(295.35-555.13)
	Female	5.16(4.36-6.16)	8.48(7.29-9.93)	314.48(278.41-359.56)	595.51(520.24-691.74)	3.57(2.44-4.93)	4.75(3.14-6.82)	146.11(106.7-195.62)	175.5(124.25-237.59)
	Male	8.24(7.03-9.72)	13.39(11.61-15.62)	293(261.64-327.78)	478.2(426.39-539.43)	4.33(2.99-5.82)	6.03(4.05-8.62)	186.86(134.1-248.85)	240.69(168.81-326.78)
Low SDI	Both	7.4(6.02-8.99)	16.63(13.5-20.08)	212.6(183.85-243.06)	587.68(507.86-674.43)	3.78(2.44-5.49)	8.08(5.31-11.48)	152.8(104.26-219.43)	334.25(229.19-462.09)
	Female	2.74(2.16-3.38)	6.68(5.25-8.27)	103.59(89.61-119.54)	304.45(259.87-354.84)	1.72(1.11-2.53)	3.68(2.44-5.24)	72.3(48.57-103.86)	153.59(104.96-212.84)
	Male	4.66(3.78-5.79)	9.96(8.08-12.19)	109.01(93.6-126.3)	283.23(244.45-328.15)	2.05(1.28-3)	4.4(2.78-6.38)	80.51(52.72-114.71)	180.66(122.08-253.99)
Low-middle SDI	Both	14.76(11.92-18.2)	24.66(20.38-30.14)	475.37(412.75-552.12)	987.85(865.68-1142.48)	9.65(6.46-13.51)	20.96(13.55-30.5)	407.9(283.96-561.2)	825.23(559.37-1159.41)
	Female	5.31(4.21-6.78)	9.17(7.44-11.44)	222.37(191.76-259.82)	501.42(435.96-586.69)	4.34(2.96-6.01)	9.07(5.89-13.07)	187.91(132.29-257.2)	356.4(243.86-490.15)
	Male	9.45(7.64-11.57)	15.49(12.69-18.79)	253(217.12-296.93)	486.43(422.81-568.45)	5.31(3.43-7.71)	11.89(7.56-17.71)	219.99(150.22-312.58)	468.84(316.65-676.68)
Middle SDI	Both	21.11(17.7-25.24)	34.2(29.68-39.81)	697.65(615.63-791.2)	1396.06(1237.55-1580.76)	18.7(13.31-24.91)	36.01(24.46-49.97)	838.04(619.59-1078.59)	1418.83(1004.63-1908.01)
	Female	8.19(6.77-9.98)	13.09(11.15-15.56)	340.22(297.91-388.89)	726.27(637.16-834.02)	8.81(6.31-11.77)	15.77(10.66-22.22)	391.98(287.45-516.41)	610.88(429.15-816.65)
	Male	12.92(10.84-15.49)	21.1(18.34-24.61)	357.43(316.7-403.95)	669.79(595.31-750.86)	9.89(6.95-13.21)	20.24(13.46-28.35)	446.06(320.39-579.46)	807.95(560.66-1090.7)
**Region**									
Africa	Both	9.81(11.58-8.15)	24.66(29.1-20.68)	282.99(323.38-249.6)	811.22(931.66-710.2)	4.47(6.44-2.95)	9.22(13.2-6.09)	182.3(256.19-125.24)	384.45(531.58-264.84)
	Female	3.87(4.73-3.15)	9.48(11.35-7.73)	139.9(161.03-123.01)	429.72(500.45-372.45)	2.16(3.1-1.43)	4.3(6.19-2.82)	90.83(127.79-61.37)	180.59(251.54-124.1)
	Male	5.95(7.06-4.92)	15.18(17.88-12.63)	143.09(164.04-125.84)	381.51(437.71-332.12)	2.31(3.38-1.49)	4.92(7.15-3.19)	91.47(129.89-60.93)	203.86(285.92-137.55)
America	Both	12.79(14.73-11.13)	21.41(23.98-19.03)	503.79(557.94-453.09)	816.09(901.82-741.48)	3.98(5.49-2.69)	13.26(19.69-8.52)	169.92(226.77-121.84)	481.79(669.24-327.98)
	Female	4.78(5.49-4.11)	8.15(9.21-7.12)	263.66(295.83-235.77)	441.3(495.39-396.21)	1.9(2.59-1.29)	6.1(8.93-3.96)	80.42(106.7-57.59)	219.91(308.62-149.7)
	Male	8.01(9.29-6.93)	13.26(14.98-11.7)	240.13(267.87-214.9)	374.79(414.31-337.69)	2.08(2.91-1.39)	7.16(10.66-4.54)	89.49(120.21-64.09)	261.88(368.97-176.95)
Asia	Both	36.78(42.75-31.58)	57.26(65.48-49.58)	1344.38(1510.15-1195.27)	2513.48(2847.67-2244.02)	31.58(42.81-21.99)	55.59(77.62-37.87)	1394.81(1819.81-1017.51	2221.28(3025.58-1550.24)
	Female	13.59(16.06-11.47)	20.45(24-17.48)	655.15(747.41-574.45)	1284.13(1476.79-1129.88)	14.31(19.28-10.09)	23.62(33.05-15.84)	632.16(838.3-464.06)	930.47(1247.16-647.29)
	Male	23.19(26.82-19.81)	36.81(41.93-31.95)	689.23(772.17-611.84)	1229.35(1372.46-1099.96)	17.26(23.49-11.79)	31.97(45.75-21.27)	762.65(1012.62-544.77)	1290.8(1777.96-893.91)
Europe	Both	10.7(12.35-9.33)	19.39(22.14-16.69)	532.81(600.33-474.66)	879.2(992.03-774.55)	3.09(4.58-1.99)	3.75(5.76-2.37)	116.29(161.56-82.08)	129.53(176.49-90.51)
	Female	4.2(4.96-3.6)	7.54(8.92-6.36)	280.51(319.15-247.83)	484.71(556.68-422.12)	1.51(2.28-0.96)	1.86(2.89-1.13)	53.02(73.27-37.04)	58.63(80.97-41.04)
	Male	6.49(7.54-5.66)	11.86(13.64-10.31)	252.3(285.77-225.23)	394.49(443.74-351.2)	1.58(2.34-1.04)	1.89(2.87-1.2)	63.28(87.49-44.49)	70.9(97.06-49.29)
Andean Latin America	Both	0.5(0.27-0.88)	1.16(0.72-1.84)	18.26(12.59-25.64)	41.22(29.75-55.92)	0.29(0.19-0.41)	0.86(0.52-1.37)	11.51(7.93-15.8)	30.09(19.21-44.87)
	Female	0.19(0.09-0.36)	0.46(0.25-0.77)	9.21(6.14-13.46)	21.52(15-30)	0.14(0.1-0.2)	0.44(0.26-0.68)	5.78(4-7.94)	15.21(9.71-22.66)
	Male	0.31(0.17-0.54)	0.7(0.42-1.12)	9.06(5.88-13.3)	19.7(13.43-27.32)	0.15(0.09-0.21)	0.43(0.26-0.67)	5.74(3.91-8.01)	14.88(9.39-22)

	Both	0.39(0.28-0.53)	0.86(0.63-1.16)	17.92(13.51-23.69)	34.17(26.37-43.61)	0.02(0.02-0.02)	0.04(0.02-0.06)	1.08(0.85-1.4)	2.26(1.53-3.22)
Australasia	Female	0.17(0.11-0.26)	0.36(0.23-0.55)	10.24(7.28-14.59)	19.39(14.08-26.43)	0.01(0.01-0.01)	0.02(0.01-0.03)	0.53(0.41-0.71)	1.05(0.71-1.49)
	Male	0.22(0.14-0.32)	0.5(0.34-0.74)	7.68(5.51-10.54)	14.78(10.99-19.95)	0.01(0.01-0.01)	0.02(0.01-0.03)	0.55(0.43-0.71)	1.21(0.81-1.74)
	Both	0.41(0.26-0.65)	0.71(0.48-1.1)	11.31(8.68-14.78)	20.78(16.6-26.55)	0.31(0.21-0.43)	0.74(0.47-1.12)	12.65(8.77-17.2)	28.37(18.72-40.71)
Caribbean	Female	0.15(0.09-0.26)	0.27(0.16-0.47)	5.3(3.99-6.99)	9.93(7.72-13.01)	0.14(0.09-0.2)	0.35(0.22-0.53)	5.9(4.06-8.26)	13.63(9.09-19.82)
	Male	0.25(0.16-0.39)	0.44(0.3-0.66)	6.01(4.56-7.83)	10.85(8.59-13.86)	0.17(0.11-0.24)	0.39(0.24-0.6)	6.75(4.7-9.17)	14.74(9.57-21.51)
	Both	1.42(0.88-2.27)	2.51(1.63-3.95)	42.8(34.24-54.02)	83.17(66.15-106.36)	0.21(0.13-0.31)	0.45(0.28-0.68)	10.65(6.96-15.39)	22.61(14.83-33.2)
Central Asia	Female	0.6(0.34-1.02)	0.97(0.59-1.66)	20.78(16.31-26.52)	40.65(31.8-52.48)	0.09(0.06-0.14)	0.19(0.12-0.28)	4.92(3.16-7.15)	9.47(6.22-13.86)
	Male	0.82(0.52-1.3)	1.54(1.01-2.45)	22.02(17.27-28.47)	42.51(32.82-54.7)	0.11(0.07-0.17)	0.26(0.16-0.4)	5.73(3.71-8.24)	13.14(8.64-19.31)
	Both	1.18(0.96-1.48)	1.76(1.49-2.11)	55.27(47.66-64.47)	82.05(70.4-95.66)	0.47(0.29-0.71)	0.46(0.27-0.75)	18.26(11.8-26.18)	16.43(10.61-24.23)
Central Europe	Female	0.44(0.34-0.58)	0.7(0.56-0.88)	29.44(24.89-35.11)	47.3(39.78-56.82)	0.21(0.13-0.32)	0.22(0.13-0.36)	7.86(5.16-11.16)	7.31(4.79-10.87)
	Male	0.74(0.6-0.93)	1.06(0.89-1.27)	25.82(21.85-30.3)	34.74(29.78-40.58)	0.26(0.16-0.39)	0.24(0.14-0.39)	10.4(6.67-15.24)	9.12(5.77-13.68)
	Both	2.63(1.94-3.65)	4.82(3.8-6.36)	60.23(49.58-73.58)	121.7(103.17-144.83)	1.33(0.91-1.81)	6.66(4.22-9.84)	55.51(40.01-73.18)	240.85(156.13-344.86)
Central Latin America	Female	0.96(0.71-1.34)	1.63(1.27-2.14)	28.76(23.75-35.01)	57.49(48.63-69.32)	0.66(0.46-0.9)	3.05(1.9-4.65)	27.85(19.97-36.5)	108.71(70.38-159.48)
	Male	1.67(1.22-2.34)	3.19(2.46-4.24)	31.46(25.3-39.54)	64.21(53.09-78.33)	0.67(0.45-0.92)	3.61(2.25-5.38)	27.67(19.73-36.98)	132.15(85.86-189.3)
	Both	0.66(0.31-1.32)	1.65(0.82-3.17)	17.55(11.97-25.08)	51.76(36.03-74.18)	0.41(0.26-0.62)	0.82(0.5-1.24)	16.47(10.77-24)	33.83(21.35-49.81)
Central Sub-Saharan Africa	Female	0.24(0.11-0.53)	0.62(0.29-1.28)	9.31(6.37-13.49)	28.72(19.71-40.84)	0.18(0.11-0.28)	0.37(0.2-0.58)	7.69(4.77-11.34)	15.41(8.64-23.36)
	Male	0.41(0.19-0.83)	1.02(0.49-1.95)	8.24(5.16-12.96)	23.04(14.57-35.21)	0.22(0.14-0.34)	0.45(0.28-0.68)	8.78(5.75-12.74)	18.42(11.91-26.78)
	Both	9.55(7.38-12.33)	9.82(8.16-11.8)	384.08(327.16-446.32)	612.15(527.77-708.15)	11.77(8.29-15.73)	13.46(9-19.55)	534.1(383.81-706.31)	528.15(364.08-732.77)
East Asia	Female	3.54(2.63-4.75)	3.31(2.68-4.16)	193.48(162.04-227.72)	337.97(283.29-400.35)	5.63(3.92-7.8)	5.84(3.79-8.69)	250.49(176.87-345.64)	221.32(147.98-311.85)
	Male	6.01(4.68-7.67)	6.51(5.36-7.79)	190.6(161.19-223.99)	274.18(237.82-314.71)	6.14(4.16-8.31)	7.63(4.96-11.44)	283.61(193.85-379.78)	306.83(207.69-443.55)
	Both	4.06(3.44-4.81)	6.71(5.66-7.92)	221.88(197.69-253.22)	355.54(310.21-412.81)	1(0.68-1.45)	1.15(0.74-1.69)	40.33(29.31-54.95)	43(30.31-59.43)
Eastern Europe	Female	1.65(1.38-1.98)	2.71(2.26-3.25)	117.94(103.6-136.1)	204.07(175.78-239.38)	0.54(0.35-0.78)	0.62(0.38-0.93)	19.81(14.14-27.71)	20.61(14.39-29.43)
	Male	2.41(2.04-2.85)	4(3.38-4.7)	103.94(91.88-120.16)	151.47(132.97-175.51)	0.47(0.32-0.66)	0.53(0.34-0.79)	20.52(14.71-28.15)	22.4(15.64-30.98)
	Both	2.32(1.75-3.1)	5.38(4.08-7.22)	73.09(62.98-85.22)	216.06(184.84-254.2)	1.32(0.87-1.92)	2.19(1.44-3.11)	53.58(36.68-75.8)	91.07(63.5-124.45)
Eastern Sub-Saharan Africa	Female	0.78(0.57-1.07)	1.8(1.34-2.45)	36.34(30.74-42.78)	116.57(97.34-138.09)	0.62(0.41-0.94)	1(0.66-1.43)	26.28(17.76-38.36)	41.59(28.78-57.18)
	Male	1.54(1.15-2.05)	3.58(2.68-4.79)	36.75(31.02-43.88)	99.48(83.26-118.8)	0.7(0.45-1.03)	1.19(0.78-1.71)	27.3(18.11-39.04)	49.48(34.55-67.46)
	Both	2.67(2.11-3.35)	3.78(2.98-4.69)	159.99(131.32-194.56)	188.5(153.28-226.5)	1.14(0.77-1.63)	1.2(0.79-1.86)	48.58(32.78-66.34)	45.22(29.8-63.87)
High-income Asia Pacific	Female	0.94(0.73-1.22)	1.27(0.97-1.61)	88.43(71.56-109.62)	101.99(81.71-124.71)	0.49(0.33-0.71)	0.46(0.31-0.72)	20.59(14.09-27.86)	17.43(11.75-24.71)
	Male	1.73(1.36-2.17)	2.51(1.97-3.13)	71.55(58.92-86.85)	86.5(71.06-104.12)	0.65(0.43-0.91)	0.74(0.48-1.11)	27.99(18.73-38.66)	27.79(18.29-39.32)
	Both	6.58(5.68-7.58)	9.46(8.38-10.66)	314.57(278.1-351.98)	413.07(372.23-458.97)	0.64(0.41-0.96)	2.43(1.51-3.77)	32.36(22.18-44.39)	86.77(58.85-122.83)
High-income North America	Female	2.47(2.07-2.93)	3.48(2.97-4.06)	168.71(147-192.45)	218.67(193.27-248.65)	0.33(0.2-0.51)	1.1(0.68-1.73)	14.91(10.36-20.56)	38.11(25.73-53.84)
	Male	4.12(3.5-4.82)	5.98(5.18-6.86)	145.86(127.97-165.38)	194.4(173.44-217.51)	0.32(0.2-0.48)	1.33(0.81-2.02)	17.45(11.92-24.34)	48.66(31.61-69.69)
	Both	6.01(4.62-7.87)	16.68(13.47-20.67)	158.21(133.54-191.68)	488.3(414.39-587.87)	2.21(1.43-3.18)	4.66(2.92-7.07)	90.81(61.82-127.06)	196.52(130.99-285.11)
North Africa and Middle East	Female	2.53(1.86-3.4)	6.42(4.97-8.36)	77.66(64.86-95.84)	245.17(204.08-300.09)	1.01(0.65-1.47)	2.09(1.3-3.13)	43.14(29.32-60.58)	89.57(59.44-129.44)
	Male	3.48(2.7-4.62)	10.26(8.36-12.6)	80.55(67.07-98.47)	243.13(206.11-292.59)	1.2(0.77-1.75)	2.57(1.57-3.98)	47.66(31.9-67.55)	106.95(69.63-158.46)
	Both	0.08(0.04-0.15)	0.17(0.07-0.38)	1.86(1.31-2.65)	4.18(2.8-6.34)	0.1(0.07-0.13)	0.24(0.17-0.34)	4.68(3.45-6.2)	11.2(7.97-15.67)
Oceania	Female	0.03(0.01-0.05)	0.06(0.03-0.14)	0.85(0.6-1.24)	1.93(1.29-2.92)	0.04(0.03-0.06)	0.09(0.07-0.13)	1.98(1.41-2.68)	4.37(3.08-6.1)
	Male	0.05(0.02-0.1)	0.11(0.05-0.25)	1(0.68-1.52)	2.24(1.44-3.67)	0.06(0.04-0.08)	0.15(0.1-0.21)	2.69(1.95-3.6)	6.83(4.81-9.66)
South Asia	Both	14.08(11.33-17.61)	23.12(18.76-28.62)	483.26(420.01-563.38)	1010.08(885.5-1169.69)	7.69(4.68-11.62)	20.3(12.39-31.55)	305.34(194.59-454.44)	770.45(490.04-1150.91)

	Female	4.75(3.68-6.09)	7.89(6.25-10.2)	215.83(184.19-253.97)	491.24(421.73-578.12)	2.95(1.73-4.53)	8.2(4.87-12.61)	118.32(73.79-179.03)	307.15(192.69-456.76)
	Male	9.33(7.44-11.6)	15.23(12.38-18.75)	267.43(230.6-315.3)	518.83(452.93-606.6)	4.74(2.81-7.36)	12.1(7.28-19.12)	187.01(116.16-277.63)	463.29(291.11-704.37)
	Both	5.85(4.41-7.94)	8.9(7.16-11.35)	192.61(163-226.91)	355.49(307.31-410.19)	9.8(7.3-12.67)	17.81(12.6-23.9)	455.5(339.7-577.25)	749.99(546.43-984.09)
Southeast Asia	Female	2.4(1.75-3.37)	3.49(2.74-4.6)	96.06(79.61-114.33)	181(153.81-211.98)	4.72(3.48-6.16)	7.94(5.56-10.82)	218.75(160.96-283.01)	329.77(242.04-435.52)
	Male	3.44(2.58-4.68)	5.41(4.36-6.91)	96.55(80.48-114.94)	174.5(150.42-202.02)	5.09(3.73-6.61)	9.87(6.87-13.47)	236.75(175.65-299.38)	420.22(298.79-553.41)
	Both	0.66(0.42-1.09)	0.96(0.64-1.46)	34.92(25.3-46.97)	50.48(36.77-67.94)	0.4(0.25-0.58)	0.7(0.43-1.07)	14.59(9.55-20.53)	23.32(15.07-33.9)
Southern Latin America	Female	0.22(0.13-0.4)	0.36(0.22-0.6)	17.53(12.21-24.76)	27.43(19.3-37.96)	0.16(0.1-0.24)	0.3(0.18-0.45)	5.96(3.98-8.43)	9.76(6.44-13.84)
	Male	0.43(0.27-0.72)	0.6(0.39-0.94)	17.39(12.51-23.66)	23.05(16.8-31.23)	0.23(0.14-0.35)	0.4(0.25-0.62)	8.63(5.61-12.2)	13.56(8.65-19.61)
	Both	0.8(0.58-1.1)	1.42(1.06-1.91)	25.96(21.56-32.15)	51.75(43.02-63.03)	0.35(0.23-0.52)	0.89(0.57-1.3)	15.52(10.22-22.34)	35.8(23.42-51.97)
Southern Sub-Saharan Africa	Female	0.27(0.18-0.38)	0.47(0.33-0.66)	11.66(9.53-14.29)	24.58(20.18-30.14)	0.15(0.1-0.23)	0.36(0.23-0.53)	6.8(4.42-9.97)	13.98(9.22-20.27)
	Male	0.53(0.39-0.74)	0.95(0.71-1.27)	14.3(11.68-17.8)	27.17(21.94-33.69)	0.2(0.13-0.3)	0.53(0.33-0.79)	8.72(5.73-12.6)	21.81(14.02-32.45)
	Both	2.08(1.58-2.8)	4.39(3.59-5.38)	66.75(56.37-79.99)	171.9(149.11-199.52)	1.07(0.73-1.47)	1.97(1.27-2.87)	45.74(32.41-60.84)	76.05(52.56-104.72)
Tropical Latin America	Female	0.8(0.6-1.1)	1.98(1.58-2.45)	35.16(29.61-42.35)	107.65(91.72-126.81)	0.49(0.33-0.67)	0.91(0.59-1.31)	21.02(14.81-28.3)	36.05(25.09-49.16)
	Male	1.28(0.96-1.75)	2.42(1.96-3.01)	31.59(26.15-38.57)	64.25(55.6-75.63)	0.58(0.4-0.8)	1.06(0.68-1.56)	24.72(17.41-32.96)	40(27.18-55.61)
	Both	4.45(3.78-5.26)	8.4(7.24-9.78)	222.11(193.81-256.07)	355.36(310.04-402.96)	1.14(0.66-1.84)	1.59(0.88-2.69)	37.53(25.37-54.61)	46.44(31.59-66.81)
Western Europe	Female	1.72(1.4-2.1)	3.23(2.65-3.91)	116.68(100.03-136.6)	189.59(161.19-220.04)	0.57(0.33-0.96)	0.8(0.44-1.37)	17.2(11.62-25.25)	21.18(14.44-29.93)
	Male	2.73(2.3-3.3)	5.17(4.43-6.16)	105.42(90.6-123.99)	165.78(144.13-189.23)	0.57(0.33-0.89)	0.79(0.45-1.32)	20.34(13.59-28.95)	25.26(16.88-36.29)
	Both	3.79(3.02-4.67)	10.19(8.18-12.55)	104.3(91.4-119.99)	317.04(275.13-366.85)	1.53(0.97-2.27)	3.35(2.15-4.79)	62.16(40.21-90.37)	144.53(98.51-204.24)
Western Sub-Saharan Africa	Female	1.59(1.26-1.98)	4.18(3.34-5.2)	51.17(44.4-59.12)	169.23(145.27-197.75)	0.78(0.49-1.17)	1.62(1.02-2.36)	32.19(20.7-48.19)	70.45(47.07-101.76)
	Male	2.19(1.76-2.71)	6.01(4.82-7.49)	53.13(45.85-61.6)	147.82(127.28-171.43)	0.76(0.47-1.16)	1.73(1.09-2.56)	29.98(19.6-44.8)	74.08(48.86-106.93)

Abbreviation: DALY, disability adjusted life-year; UI, uncertainty interval; SDI, socio-demographic index.

Table S2. The age standardized rates and their variation trends of diabetes mellitus type 1 related chronic kidney disease.

Location	Sex	ASIR (95%UI)		EAPC (95%CI)	ASPR (95%UI)		EAPC (95%CI)	ASDR (95%UI)		EAPC (95%CI)	Age-standardized DALY rate (95%UI)		EAPC (95%CI)
		1990	2019		1990	2019		1990	2019		1990	2019	
	Both	1.27(1.14-1.41)	1.63(1.46-1.82)	1.21(1.08-1.35)	51.1(46.5-56.78)	63.43(57.27-70.93)	1.15(1-1.31)	0.96(0.67-1.31)	0.98(0.66-1.38)	0.08(-0.02-0.19)	39.48(28.4-52.75)	38.83(27.73-52.35)	-0.08(-0.18-0.02)
Global	Female	0.97(0.86-1.09)	1.23(1.09-1.39)	1.15(1.01-1.29)	51.77(46.72-58.04)	66.7(59.35-75.55)	1.23(1.09-1.38)	0.87(0.6-1.19)	0.84(0.58-1.17)	-0.16(-0.27--0.05)	36.14(26.21-48.17)	33.28(23.92-44.66)	-0.38(-0.51--0.26)
	Male	1.57(1.41-1.75)	2.03(1.82-2.26)	1.25(1.11-1.39)	50.44(45.86-55.84)	60.24(54.68-66.66)	1.06(0.9-1.23)	1.06(0.71-1.45)	1.13(0.75-1.6)	0.27(0.16-0.38)	42.92(30.43-57.82)	44.47(31.16-60.7)	0.16(0.06-0.26)
**Socio-demographic index**													
	Both	1.63(1.42-1.87)	1.93(1.71-2.2)	0.9(0.77-1.03)	76.2(67.84-85.4)	85.86(76.86-95.94)	0.74(0.58-0.9)	0.32(0.21-0.45)	0.37(0.24-0.53)	0.58(0.5-0.66)	14.15(9.97-18.78)	15.99(11.03-21.44)	0.49(0.41-0.58)
High SDI	Female	1.17(1-1.36)	1.37(1.19-1.6)	0.87(0.73-1)	80.17(70.37-91.53)	91.8(80.95-103.71)	0.78(0.62-0.95)	0.27(0.18-0.38)	0.3(0.2-0.43)	0.51(0.43-0.59)	11.97(8.52-15.92)	13.24(9.33-17.65)	0.43(0.34-0.53)
	Male	2.11(1.83-2.43)	2.5(2.19-2.85)	0.89(0.77-1.02)	72.55(64.79-81.38)	80.58(72.03-89.65)	0.7(0.54-0.85)	0.38(0.25-0.54)	0.44(0.29-0.63)	0.57(0.48-0.67)	16.46(11.55-21.99)	18.76(12.84-25.79)	0.51(0.42-0.59)
	Both	1.22(1.04-1.44)	1.59(1.36-1.88)	1.37(1.2-1.55)	51.95(46.47-58.3)	69.34(61.32-78.94)	1.39(1.22-1.56)	0.7(0.49-0.95)	0.56(0.38-0.78)	-0.95(-1.16--0.75)	28.81(20.95-38.02)	22.57(16.39-29.76)	-1.05(-1.25--0.85)
High-middle SDI	Female	0.94(0.79-1.13)	1.26(1.06-1.51)	1.4(1.23-1.57)	53.22(47.17-60.9)	77.15(66.89-90.2)	1.62(1.47-1.77)	0.6(0.41-0.83)	0.47(0.31-0.65)	-1.16(-1.4--0.92)	24.78(18.05-33.2)	18.69(13.55-24.8)	-1.3(-1.55--1.04)
	Male	1.5(1.28-1.76)	1.93(1.65-2.28)	1.36(1.18-1.54)	50.63(45.29-56.67)	62.03(54.97-70.02)	1.15(0.95-1.35)	0.81(0.56-1.1)	0.65(0.45-0.91)	-0.81(-1--0.62)	33.06(23.76-43.88)	26.48(19.02-35.4)	-0.88(-1.05--0.71)
	Both	1.1(0.93-1.31)	1.28(1.07-1.51)	1.2(0.96-1.44)	45.27(39.63-51.12)	55.51(48.49-62.97)	1.25(1.06-1.44)	1.29(0.83-1.88)	1.24(0.78-1.78)	-0.16(-0.24--0.07)	46.26(30.62-67.35)	45.02(30.03-63.58)	-0.08(-0.17-0.01)
Low SDI	Female	0.81(0.67-0.98)	1.03(0.84-1.25)	1.19(1-1.38)	45.03(39.14-51.53)	58.02(49.75-66.74)	1.35(1.17-1.54)	1.16(0.74-1.72)	1.11(0.71-1.58)	-0.2(-0.25--0.14)	43.22(28.04-62.75)	40.87(27.53-57.52)	-0.21(-0.27--0.16)
	Male	1.39(1.16-1.66)	1.53(1.28-1.82)	1.23(0.95-1.51)	45.41(39.48-51.98)	52.81(46.03-60.38)	1.13(0.93-1.34)	1.41(0.88-2.12)	1.37(0.85-2.01)	-0.09(-0.22-0.03)	49.07(31.33-70.39)	49.24(31.96-71.24)	0.05(-0.07-0.18)
	Both	1.12(0.93-1.36)	1.41(1.18-1.7)	1.18(1.01-1.35)	43.86(38.63-50.43)	54.76(48.22-63.03)	1.24(1.07-1.41)	1.28(0.85-1.81)	1.35(0.87-1.97)	0.19(0.03-0.35)	49.19(33.83-69.02)	50.69(33.91-71.85)	0.12(-0.04-0.28)
Low-middle SDI	Female	0.81(0.65-1.01)	1.06(0.86-1.31)	1.29(1.11-1.46)	42.07(36.84-48.49)	55.52(48.52-64.65)	1.38(1.22-1.53)	1.15(0.76-1.6)	1.15(0.75-1.66)	-0.11(-0.24-0.02)	45.33(31.45-62.84)	43.24(29.18-60.2)	-0.27(-0.41--0.14)
	Male	1.42(1.18-1.7)	1.76(1.45-2.11)	1.15(0.98-1.32)	45.51(39.72-52.88)	53.9(47.11-62.52)	1.12(0.93-1.3)	1.4(0.89-2.03)	1.56(0.99-2.35)	0.45(0.23-0.67)	52.84(35.09-76.05)	58.33(38.5-85.11)	0.45(0.24-0.66)
	Both	1.13(0.96-1.33)	1.55(1.34-1.82)	1.53(1.39-1.68)	39.85(35.38-44.76)	56.68(50.45-64.05)	1.62(1.45-1.78)	1.4(0.98-1.89)	1.34(0.92-1.86)	-0.08(-0.14--0.02)	57.63(41.61-75.64)	52.41(37.75-69.66)	-0.31(-0.36--0.25)
Middle SDI	Female	0.89(0.73-1.06)	1.23(1.04-1.47)	1.39(1.27-1.51)	39.32(34.7-44.64)	59.02(51.98-67.78)	1.74(1.58-1.89)	1.34(0.94-1.81)	1.16(0.79-1.61)	-0.47(-0.56--0.38)	54.82(39.59-73.04)	44.86(32.07-59.48)	-0.73(-0.83--0.63)
	Male	1.37(1.17-1.61)	1.87(1.61-2.2)	1.64(1.47-1.8)	40.32(35.87-45.32)	54.32(48.27-61.1)	1.49(1.31-1.67)	1.47(1.01-1.96)	1.53(1.03-2.15)	0.26(0.2-0.31)	60.37(43.01-79.79)	60.05(42.25-80)	0.05(0.01-0.09)
**Region**													
	Both	1.3(1.12-1.52)	1.79(1.53-2.08)	1.42(1.27-1.57)	50.6(45.05-57.26)	66.17(58.33-75.31)	1.32(1.18-1.46)	1.28(0.83-1.86)	1.17(0.75-1.72)	-0.26(-0.33--0.19)	45.99(30.79-65.72)	42.2(28.45-59.49)	-0.25(-0.32--0.17)
Africa	Female	1(0.83-1.19)	1.35(1.13-1.61)	1.4(1.22-1.59)	49.77(43.93-56.88)	68.9(60.21-79.28)	1.49(1.35-1.63)	1.2(0.78-1.76)	1.05(0.67-1.55)	-0.42(-0.47--0.38)	44.85(29.6-64.02)	38.45(26.01-54.45)	-0.51(-0.56--0.45)
	Male	1.61(1.38-1.88)	2.23(1.9-2.61)	1.44(1.31-1.58)	51.35(45.31-58.34)	63.19(55.38-72.1)	1.14(1-1.28)	1.36(0.86-2.03)	1.3(0.82-1.91)	-0.1(-0.2-0)	47.12(30.84-67.64)	46.14(30.4-65.21)	0(-0.11-0.1)
	Both	1.84(1.61-2.11)	2.17(1.9-2.46)	0.61(0.53-0.69)	71.45(64.45-79.07)	77.49(70.19-85.63)	0.4(0.3-0.51)	0.63(0.42-0.88)	1.1(0.71-1.59)	2.05(1.82-2.28)	26.14(18.46-35.16)	41.39(28.42-56.86)	1.66(1.49-1.84)
America	Female	1.36(1.17-1.56)	1.61(1.39-1.84)	0.63(0.56-0.69)	73.16(65.59-81.87)	81.67(73.24-91.69)	0.54(0.45-0.63)	0.57(0.38-0.78)	0.96(0.63-1.39)	1.96(1.7-2.23)	23.81(16.83-31.97)	36.47(25.11-50.5)	1.55(1.34-1.75)
	Male	2.35(2.04-2.69)	2.74(2.39-3.15)	0.59(0.49-0.68)	69.65(62.67-77.52)	73.07(65.71-81.03)	0.25(0.13-0.38)	0.7(0.46-0.99)	1.25(0.8-1.83)	2.11(1.9-2.31)	28.67(20.12-38.71)	46.72(31.63-64.83)	1.76(1.6-1.91)
	Both	1.07(0.93-1.24)	1.36(1.17-1.57)	1.21(1.04-1.39)	42.27(37.82-47.55)	53.51(47.83-60.37)	1.27(1.09-1.45)	1.24(0.86-1.68)	1.1(0.75-1.52)	-0.43(-0.54--0.33)	50.79(36.17-67.71)	43.68(30.83-59.29)	-0.55(-0.65--0.45)
Asia	Female	0.81(0.69-0.95)	1.01(0.85-1.18)	1.15(0.97-1.32)	42.23(37.32-48)	55.31(48.69-63.71)	1.32(1.15-1.49)	1.14(0.79-1.56)	0.93(0.63-1.29)	-0.8(-0.92--0.68)	46.94(33.69-62.94)	36.92(26.07-49.04)	-0.96(-1.1--0.83)
	Male	1.33(1.15-1.52)	1.7(1.46-1.94)	1.27(1.09-1.44)	42.27(37.69-47.12)	51.78(46.4-58.02)	1.23(1.04-1.43)	1.34(0.89-1.83)	1.26(0.85-1.81)	-0.14(-0.25--0.02)	54.49(38.48-73.2)	50.32(35.21-68.79)	-0.22(-0.32--0.12)
	Both	1.38(1.19-1.62)	2.1(1.79-2.44)	2.03(1.82-2.25)	62.93(55.85-71.01)	94.03(82.34-106.82)	1.95(1.73-2.17)	0.31(0.21-0.46)	0.26(0.17-0.39)	-0.87(-1--0.74)	12.55(8.95-17.07)	10.9(7.8-14.53)	-0.7(-0.81--0.59)
Europe	Female	1.05(0.88-1.26)	1.61(1.34-1.95)	2.02(1.82-2.22)	64.22(56.43-73.4)	103.62(89.34-120.88)	2.16(1.96-2.36)	0.27(0.17-0.39)	0.23(0.15-0.34)	-0.83(-0.94--0.71)	10.65(7.59-14.48)	9.28(6.69-12.24)	-0.69(-0.79--0.59)
	Male	1.74(1.5-2.05)	2.61(2.24-3.04)	2.04(1.81-2.28)	61.76(55.17-70.07)	84.99(75.01-97.14)	1.73(1.48-1.99)	0.37(0.24-0.55)	0.31(0.2-0.45)	-0.96(-1.12--0.81)	14.7(10.35-20.21)	12.65(8.89-17.15)	-0.73(-0.85--0.6)
	Both	1.17(0.71-1.91)	1.84(1.15-2.89)	1.94(1.79-2.09)	50.22(36.33-67.84)	64.04(46.67-86.35)	1.33(1.14-1.52)	1.21(0.79-1.75)	1.48(0.88-2.31)	0.82(0.51-1.13)	43.25(28.91-60.39)	49.76(31.45-75.49)	0.56(0.25-0.88)
Andean Latin America	Female	0.88(0.47-1.54)	1.46(0.8-2.45)	2.1(1.94-2.27)	50.09(35.11-70.54)	66.4(46.55-92.43)	1.42(1.23-1.62)	1.16(0.75-1.7)	1.45(0.87-2.28)	0.73(0.44-1.01)	42.21(28.45-59.24)	49.06(30.89-73.18)	0.46(0.18-0.75)
	Male	1.45(0.87-2.31)	2.21(1.34-3.49)	1.84(1.7-1.98)	50.23(34.44-71.23)	61.56(42.57-84.52)	1.23(1.05-1.42)	1.26(0.8-1.85)	1.5(0.91-2.38)	0.91(0.56-1.26)	44.33(29.43-63.22)	50.52(31.65-75.7)	0.67(0.31-1.03)
Australasia	Both	1.83(1.29-2.56)	2.33(1.64-3.28)	1.54(1.25-1.84)	82.24(61.41-108.1)	104.77(78.36-139.46)	1.44(1.18-1.7)	0.08(0.07-0.1)	0.09(0.05-0.14)	0.61(0.23-0.99)	4.86(3.79-6.31)	6.01(4.13-8.51)	1.21(1.05-1.37)

	Female	1.59(1.02-2.44)	1.89(1.18-2.96)	1.25(0.96-1.54)	92.81(65.39-132.45)	118.37(82.64-167.83)	1.31(1.06-1.56)	0.08(0.06-0.1)	0.08(0.04-0.12)	0.62(0.2-1.04)	4.67(3.57-6.3)	5.55(3.73-7.92)	1.08(0.9-1.26)
	Male	2.08(1.37-3.13)	2.8(1.82-4.31)	1.74(1.43-2.06)	71.58(50.96-98.53)	90.86(64.5-126.8)	1.6(1.3-1.9)	0.09(0.07-0.11)	0.1(0.06-0.16)	0.59(0.22-0.96)	5.05(3.95-6.6)	6.51(4.34-9.38)	1.33(1.18-1.48)
	Both	1.06(0.7-1.65)	1.61(1.05-2.54)	1.6(1.5-1.69)	31.78(25.27-40.65)	43.83(34.44-56.98)	1.4(1.27-1.52)	1.1(0.72-1.55)	1.45(0.91-2.18)	1.35(1.24-1.46)	42.74(29.16-59)	56.08(37.47-79.94)	1.29(1.19-1.39)
Caribbean	Female	0.79(0.46-1.34)	1.26(0.72-2.2)	1.71(1.64-1.79)	29.16(22.39-37.54)	41.55(31.93-55.49)	1.47(1.36-1.58)	0.96(0.63-1.38)	1.33(0.85-1.99)	1.57(1.42-1.72)	38.4(25.86-54.16)	52.76(35.34-75.84)	1.49(1.36-1.62)
	Male	1.34(0.88-2.01)	1.97(1.31-2.98)	1.53(1.42-1.63)	34.45(27.12-43.87)	46.19(36.45-59.65)	1.34(1.2-1.47)	1.24(0.81-1.79)	1.57(0.98-2.39)	1.18(1.07-1.28)	47.27(32.21-65.71)	59.67(39.02-86.46)	1.12(1.03-1.22)
	Both	1.86(1.22-2.88)	2.68(1.74-4.25)	1.63(1.39-1.88)	65.74(53.56-80.93)	86.79(69.4-110.56)	1.35(1.1-1.6)	0.37(0.23-0.56)	0.49(0.31-0.73)	0.3(-0.16-0.76)	18.27(11.8-26.49)	23.37(15.46-34.07)	0.22(-0.16-0.6)
Central Asia	Female	1.54(0.93-2.55)	2.11(1.25-3.63)	1.35(1.13-1.56)	61.64(49.11-77.64)	83.85(65.68-107.65)	1.27(1.05-1.5)	0.32(0.2-0.49)	0.39(0.25-0.59)	0.02(-0.42-0.45)	15.96(10.32-23.44)	19.06(12.61-27.64)	-0.06(-0.43-0.31)
	Male	2.22(1.47-3.33)	3.28(2.17-5.16)	1.83(1.56-2.1)	70.36(56.92-88.29)	90.5(70.48-115.38)	1.43(1.15-1.72)	0.43(0.27-0.65)	0.6(0.38-0.9)	0.53(0.06-1.01)	20.85(13.38-30.35)	28.12(18.59-40.77)	0.45(0.06-0.85)
	Both	1.03(0.83-1.32)	1.51(1.23-1.89)	1.73(1.59-1.86)	43.83(37.49-51.49)	68.43(57.42-81.78)	2.03(1.86-2.21)	0.33(0.2-0.49)	0.24(0.14-0.38)	-0.89(-1.24--0.54)	13.08(8.55-18.66)	10.01(6.63-14.47)	-0.64(-0.87--0.42)
Central Europe	Female	0.76(0.58-1.03)	1.22(0.94-1.6)	1.99(1.86-2.12)	46.17(38.69-55.62)	80.01(65.7-98.34)	2.32(2.15-2.48)	0.27(0.17-0.4)	0.2(0.12-0.32)	-0.95(-1.29--0.61)	10.74(7.14-14.98)	8.5(5.67-12.03)	-0.65(-0.84--0.45)
	Male	1.31(1.05-1.67)	1.82(1.47-2.26)	1.57(1.43-1.72)	41.61(34.97-48.94)	57.49(48.39-68.12)	1.71(1.51-1.9)	0.39(0.24-0.58)	0.28(0.16-0.45)	-0.86(-1.23--0.48)	15.6(10.08-22.53)	11.59(7.55-16.99)	-0.66(-0.91--0.4)
	Both	1.31(1-1.76)	2.05(1.61-2.7)	1.58(1.5-1.65)	34.25(28.7-40.65)	48.17(40.65-57.58)	1.32(1.23-1.41)	1.29(0.86-1.81)	2.67(1.7-3.95)	2.73(2.34-3.12)	47.9(32.78-64.66)	94.62(60.96-135.3)	2.57(2.24-2.89)
Central Latin America	Female	0.95(0.72-1.29)	1.41(1.09-1.87)	1.4(1.34-1.46)	32.48(27.08-38.7)	44.49(37.71-53.62)	1.24(1.15-1.33)	1.24(0.83-1.73)	2.32(1.45-3.55)	2.36(1.95-2.78)	46.28(32.19-62.3)	81.27(52.31-119.21)	2.16(1.82-2.49)
	Male	1.67(1.26-2.27)	2.69(2.08-3.57)	1.67(1.58-1.76)	35.97(29.58-43.76)	51.79(42.9-63.27)	1.39(1.3-1.49)	1.34(0.89-1.9)	3.08(1.93-4.6)	3.09(2.71-3.46)	49.6(33.6-68.03)	109.47(71.11-157.02)	2.96(2.64-3.27)
	Both	0.83(0.46-1.55)	1.03(0.59-1.83)	1.01(0.85-1.16)	34.8(25.35-47.02)	41.58(31.21-55.93)	1(0.82-1.18)	1.42(0.89-2.16)	1.17(0.71-1.83)	-0.77(-0.83--0.71)	49.66(31.62-74.28)	40.94(25.48-61.3)	-0.75(-0.8--0.7)
Central Sub-Saharan Africa	Female	0.61(0.31-1.18)	0.79(0.43-1.48)	1.13(0.94-1.32)	37.66(26.92-52.72)	47.12(34.12-64.78)	1.1(0.91-1.29)	1.18(0.7-1.85)	1(0.53-1.63)	-0.67(-0.77--0.58)	43.3(26.47-65.85)	36.06(19.57-56.11)	-0.73(-0.82--0.64)
	Male	1.05(0.55-1.95)	1.27(0.7-2.23)	0.93(0.79-1.07)	31.64(21.4-46.88)	35.75(24.51-51.63)	0.89(0.72-1.06)	1.69(1.03-2.6)	1.38(0.84-2.15)	-0.81(-0.88--0.74)	56.83(36.36-85.04)	46.55(29.25-69.64)	-0.76(-0.83--0.7)
	Both	0.79(0.61-1.01)	0.83(0.65-1.05)	0.5(0.32-0.68)	30.25(25.96-35.06)	40.06(34.58-46.59)	1.07(0.93-1.22)	1.07(0.74-1.42)	0.66(0.45-0.93)	-1.74(-1.91--1.57)	45.62(32.51-60.51)	26.85(19.1-36.2)	-2(-2.19--1.81)
East Asia	Female	0.61(0.46-0.81)	0.61(0.46-0.8)	0.29(0.1-0.49)	30.97(26.12-36.1)	45.01(38.04-53.6)	1.27(1.14-1.4)	1.05(0.71-1.49)	0.56(0.38-0.81)	-2.41(-2.65--2.17)	44.45(30.77-61.15)	22.45(15.51-30.81)	-2.72(-3.02--2.43)
	Male	0.96(0.75-1.21)	1.04(0.82-1.29)	0.61(0.44-0.79)	29.56(25.06-34.59)	35.3(30.28-40.65)	0.85(0.67-1.02)	1.09(0.74-1.45)	0.75(0.5-1.12)	-1.17(-1.31--1.03)	46.73(32.33-63)	31.15(21.41-44.01)	-1.39(-1.55--1.24)
	Both	1.93(1.62-2.3)	3.24(2.7-3.87)	2.46(2.17-2.75)	92.88(82.45-106)	156.02(135.02-181.61)	2.41(2.14-2.69)	0.37(0.26-0.53)	0.36(0.24-0.52)	-0.68(-1.05--0.32)	15.4(11.23-20.87)	14.91(10.74-20.26)	-0.71(-1.05--0.37)
Eastern Europe	Female	1.5(1.22-1.83)	2.54(2.06-3.09)	2.43(2.08-2.78)	92.4(80.88-107.39)	172.97(146.99-205.37)	2.77(2.42-3.12)	0.33(0.22-0.48)	0.31(0.2-0.46)	-0.68(-0.98--0.39)	13.52(9.82-18.48)	12.58(9.06-17.54)	-0.78(-1.05--0.5)
	Male	2.39(2.01-2.86)	4.01(3.36-4.77)	2.53(2.25-2.81)	93.27(82.57-108.11)	139.84(122.23-162.88)	2.05(1.79-2.3)	0.42(0.29-0.6)	0.41(0.27-0.59)	-0.72(-1.15--0.29)	17.6(12.81-23.93)	17.58(12.58-24.14)	-0.64(-1.04--0.24)
	Both	0.96(0.77-1.2)	1.17(0.94-1.49)	1.06(0.88-1.25)	46.01(39.71-53.08)	59.31(51.12-68.67)	1.41(1.21-1.61)	1.41(0.9-2.09)	1.06(0.68-1.56)	-1.13(-1.25--1.02)	49.4(32.62-71.59)	36.66(24.87-51.75)	-1.16(-1.28--1.05)
Eastern Sub-Saharan Africa	Female	0.63(0.49-0.8)	0.8(0.63-1.03)	1.19(0.97-1.41)	45.93(39.23-53.81)	64.1(54.15-75.22)	1.64(1.44-1.84)	1.27(0.81-1.93)	0.93(0.59-1.38)	-1.29(-1.41--1.16)	46.38(30.19-69.17)	32.63(21.77-46.46)	-1.44(-1.58--1.31)
	Male	1.28(1.03-1.63)	1.55(1.24-1.97)	1.01(0.84-1.18)	45.96(39.47-53.7)	54.05(46.14-62.85)	1.14(0.94-1.35)	1.55(0.98-2.35)	1.2(0.76-1.8)	-0.97(-1.1--0.84)	52.3(33.64-77.26)	40.95(27.44-57.78)	-0.9(-1.02--0.77)
	Both	1.5(1.19-1.92)	1.61(1.28-2.02)	0.93(0.67-1.19)	84(69.07-102.29)	86.56(70.16-104.8)	0.68(0.46-0.9)	0.56(0.38-0.79)	0.32(0.21-0.47)	-1.91(-2.11--1.71)	23.75(16.34-32.23)	15.29(10.25-21.2)	-1.36(-1.56--1.16)
High-income Asia Pacific	Female	1.03(0.79-1.35)	1.08(0.83-1.4)	0.98(0.67-1.29)	92.29(74.16-114.32)	97.51(77.57-120.64)	0.75(0.52-0.98)	0.45(0.3-0.64)	0.23(0.15-0.33)	-2.36(-2.51--2.22)	19.5(13.55-26.03)	11.73(8.01-16.17)	-1.5(-1.64--1.36)
	Male	1.99(1.55-2.56)	2.15(1.7-2.71)	0.88(0.64-1.12)	76.01(62.41-91.73)	76.46(62.58-92.23)	0.61(0.4-0.82)	0.69(0.46-0.98)	0.42(0.28-0.62)	-1.68(-1.92--1.44)	28.38(19.4-38.87)	18.91(12.5-26.31)	-1.3(-1.54--1.06)
	Both	2.34(1.99-2.73)	2.27(1.96-2.63)	-0.11(-0.2--0.02)	104.38(92.23-117.03)	104.29(93.24-117.35)	0.11(0.01-0.21)	0.19(0.12-0.28)	0.42(0.27-0.64)	2.92(2.7-3.15)	10.2(6.93-14.1)	17.31(11.53-23.88)	1.75(1.54-1.97)
High-income North America	Female	1.65(1.38-1.99)	1.56(1.31-1.86)	-0.21(-0.34--0.08)	108.34(94.43-123.92)	109.01(95.4-125.02)	0.2(0.08-0.33)	0.17(0.11-0.26)	0.36(0.23-0.55)	2.94(2.67-3.2)	8.89(6.12-12.22)	14.74(9.86-20.25)	1.76(1.47-2.06)
	Male	3.06(2.58-3.6)	3(2.55-3.52)	-0.08(-0.15-0)	100.48(87.98-114.13)	99.57(88.32-112.54)	0(-0.08-0.08)	0.21(0.13-0.32)	0.5(0.31-0.74)	2.9(2.7-3.11)	11.57(7.84-16.15)	20.04(13.24-27.9)	1.74(1.58-1.9)
	Both	1.57(1.26-1.96)	2.86(2.32-3.51)	2.19(2.12-2.25)	48(41.07-56.74)	79.32(67.83-94.6)	1.81(1.77-1.86)	1.08(0.68-1.59)	0.9(0.56-1.38)	-0.71(-0.76--0.65)	39.31(25.93-55.58)	34.01(22.3-49.5)	-0.55(-0.6--0.5)
North Africa and Middle East	Female	1.28(0.99-1.65)	2.25(1.75-2.9)	2.07(2-2.13)	47.84(40.86-57.88)	81.42(68.17-99.04)	1.84(1.79-1.89)	0.98(0.62-1.44)	0.83(0.51-1.25)	-0.67(-0.74--0.6)	37.1(24.49-53.05)	31.97(20.79-46.75)	-0.59(-0.66--0.53)
	Male	1.85(1.48-2.3)	3.42(2.8-4.17)	2.25(2.17-2.33)	48.16(41.12-57.32)	77.59(66.29-92.26)	1.8(1.73-1.86)	1.18(0.74-1.78)	0.97(0.59-1.51)	-0.74(-0.79--0.69)	41.46(26.98-59.68)	35.94(23.19-53.37)	-0.52(-0.56--0.47)
	Both	0.84(0.43-1.62)	1.01(0.46-2.14)	0.62(0.55-0.69)	26.86(20-36.48)	29.82(20.85-43.07)	0.46(0.41-0.51)	2.2(1.54-3.05)	2.32(1.57-3.29)	0.01(-0.23-0.25)	93.8(67.26-127.42)	97.19(68.36-135.86)	-0.05(-0.29-0.19)
Oceania	Female	0.65(0.32-1.19)	0.77(0.34-1.61)	0.52(0.43-0.61)	25.98(19-35.72)	28.85(19.98-42.11)	0.46(0.4-0.52)	1.92(1.28-2.73)	1.88(1.26-2.62)	-0.26(-0.56-0.04)	82.04(56.9-113.11)	78.09(54.19-108.1)	-0.36(-0.66--0.05)
	Male	1.01(0.51-2.03)	1.23(0.55-2.69)	0.67(0.61-0.73)	27.64(20.04-39.75)	30.65(20.78-48.16)	0.46(0.41-0.5)	2.47(1.68-3.44)	2.74(1.84-3.95)	0.2(0-0.4)	104.8(74.55-142.46)	115.68(80.7-163.28)	0.18(-0.01-0.37)
South Asia	Both	1.14(0.94-1.39)	1.32(1.08-1.62)	1(0.77-1.22)	46.4(40.54-53.36)	54.24(47.8-62.3)	1.17(0.94-1.4)	1.09(0.66-1.65)	1.28(0.78-1.98)	0.57(0.35-0.79)	39.37(24.52-59.2)	46.48(29.28-70.65)	0.68(0.46-0.9)
	Female	0.79(0.63-0.99)	0.91(0.72-1.16)	0.97(0.74-1.2)	43.56(37.59-50.96)	53.39(46.23-62.3)	1.25(1.03-1.47)	0.88(0.51-1.36)	1.04(0.61-1.6)	0.51(0.37-0.65)	31.77(19.35-47.85)	37.4(23.18-56.2)	0.55(0.42-0.67)

	Male	1.47(1.2-1.76)	1.71(1.39-2.08)	1.04(0.81-1.27)	48.95(42.68-57.13)	54.99(48.22-63.65)	1.11(0.87-1.36)	1.29(0.76-2.02)	1.52(0.91-2.4)	0.67(0.35-0.99)	46.18(27.93-69.96)	55.44(34.61-84.81)	0.82(0.5-1.13)
	Both	1.07(0.82-1.42)	1.45(1.15-1.86)	1.35(1.19-1.52)	40.28(34.73-46.77)	51.16(44.26-59.12)	1.48(1.13-1.83)	2.77(1.99-3.62)	2.52(1.77-3.39)	-0.29(-0.34--0.25)	116.81(87.43-150.17)	103.13(75.65-135.16)	-0.44(-0.49--0.39)
Southeast Asia	Female	0.88(0.65-1.2)	1.17(0.91-1.56)	1.23(1.09-1.38)	39.84(33.93-46.95)	52.19(44.34-61.31)	1.57(1.2-1.94)	2.59(1.89-3.42)	2.21(1.56-3.01)	-0.5(-0.55--0.46)	109.45(80.95-143.8)	90(66.62-118.57)	-0.64(-0.7--0.58)
	Male	1.26(0.97-1.67)	1.72(1.38-2.21)	1.43(1.24-1.61)	40.7(34.65-47.7)	50.18(43.16-58.29)	1.39(1.06-1.73)	2.96(2.1-3.94)	2.86(1.97-3.88)	-0.12(-0.23--0.01)	124.55(91.07-160.77)	116.53(84.1-154.4)	-0.27(-0.39--0.15)
	Both	1.34(0.86-2.19)	1.45(0.91-2.33)	0.81(0.59-1.02)	71.55(52.17-95.7)	72.35(52.13-98.57)	0.46(0.26-0.66)	0.85(0.53-1.24)	0.87(0.54-1.32)	-0.01(-0.28-0.26)	30.98(20.31-43.64)	30.25(19.79-43.15)	-0.12(-0.33-0.09)
Southern Latin America	Female	0.9(0.53-1.6)	1.06(0.62-1.86)	1.04(0.85-1.22)	70.39(49.21-99.06)	76.99(53.38-109.39)	0.59(0.43-0.76)	0.64(0.41-0.93)	0.68(0.42-1.02)	0.15(-0.14-0.44)	24.11(16.05-34.23)	24.1(15.97-33.86)	0.02(-0.21-0.24)
	Male	1.81(1.14-2.93)	1.86(1.15-2.99)	0.68(0.44-0.92)	72.93(52.67-98.99)	67.75(48.68-93.01)	0.31(0.07-0.56)	1.09(0.67-1.65)	1.1(0.67-1.68)	-0.12(-0.38-0.14)	38.72(24.97-54.96)	37.16(23.85-53.32)	-0.21(-0.42--0.01)
	Both	1.49(1.14-1.94)	1.82(1.39-2.42)	0.93(0.63-1.23)	53.06(45.03-62.9)	64.4(53.85-77.55)	1.04(0.82-1.27)	1.03(0.67-1.55)	1.34(0.85-1.98)	1.51(1.1-1.91)	41.4(27.58-60.72)	49.96(32.46-72.28)	1.2(0.78-1.62)
Southern Sub-Saharan Africa	Female	0.91(0.64-1.24)	1.17(0.83-1.62)	1.02(0.69-1.35)	45.24(37.71-54.55)	59.3(48.92-72.29)	1.24(1-1.48)	0.81(0.52-1.23)	0.99(0.63-1.48)	1.6(1.2-2)	33.36(21.93-49.81)	36.75(24.15-53.59)	1.14(0.68-1.61)
	Male	2.13(1.63-2.77)	2.53(1.94-3.29)	0.89(0.63-1.16)	61.52(50.98-73.69)	70.22(57.51-85.98)	0.89(0.68-1.1)	1.3(0.82-1.97)	1.77(1.1-2.64)	1.49(1-1.97)	50.61(32.98-74.02)	65.21(41.74-95.88)	1.27(0.8-1.73)
	Both	1.35(1.06-1.77)	2.04(1.63-2.55)	1.32(1.07-1.56)	44.02(38.02-51.78)	72.71(62.75-84.64)	1.63(1.36-1.91)	0.96(0.65-1.33)	0.78(0.51-1.13)	-0.85(-0.97--0.72)	37.59(26.18-50.64)	30.02(20.87-41.12)	-0.98(-1.11--0.86)
Tropical Latin America	Female	1.04(0.79-1.38)	1.76(1.39-2.2)	1.88(1.78-1.98)	45.91(39.41-54.29)	87.76(74.77-103.96)	2.25(2.08-2.43)	0.84(0.56-1.16)	0.68(0.44-0.98)	-0.91(-1.06--0.75)	33.32(22.91-45.07)	27.25(19.21-36.92)	-0.95(-1.1--0.8)
	Male	1.66(1.29-2.2)	2.31(1.85-2.92)	0.94(0.58-1.31)	41.93(35.35-50.07)	56.91(48.86-67.83)	0.84(0.39-1.29)	1.09(0.72-1.51)	0.9(0.58-1.32)	-0.77(-0.87--0.67)	42.16(29.2-56.89)	33.18(22.83-45.95)	-0.99(-1.09--0.88)
	Both	1.09(0.91-1.33)	1.47(1.25-1.74)	1.75(1.44-2.07)	52.91(45.96-61.2)	70.92(61.1-81.65)	1.72(1.33-2.11)	0.2(0.12-0.31)	0.17(0.1-0.27)	-0.12(-0.26-0.03)	7.54(5.17-10.63)	6.68(4.63-9.27)	0.01(-0.13-0.14)
Western Europe	Female	0.78(0.64-0.97)	1.1(0.89-1.37)	1.84(1.53-2.16)	54.63(46.35-64.29)	76.95(64.71-91.2)	1.79(1.38-2.2)	0.17(0.1-0.27)	0.15(0.09-0.24)	-0.17(-0.37-0.03)	6.34(4.34-8.99)	5.78(4.04-7.81)	0(-0.16-0.16)
	Male	1.4(1.16-1.74)	1.85(1.58-2.22)	1.69(1.37-2.01)	51.47(44.07-60.46)	65.23(56.31-75.65)	1.63(1.26-2.01)	0.24(0.15-0.37)	0.2(0.12-0.31)	-0.14(-0.28-0)	8.9(5.99-12.56)	7.64(5.2-10.67)	-0.02(-0.16-0.13)
	Both	1.57(1.32-1.86)	1.98(1.66-2.36)	1.23(1.02-1.44)	61.81(54.35-70.27)	76.28(66.7-87.09)	1.15(1.01-1.29)	1.43(0.91-2.16)	1.34(0.84-1.94)	-0.26(-0.35--0.17)	51.4(33.07-75.75)	49.08(32.4-69.78)	-0.16(-0.25--0.07)
Western Sub-Saharan Africa	Female	1.34(1.1-1.61)	1.64(1.34-1.98)	1.2(0.93-1.47)	61.12(53.3-69.95)	79.27(68.59-91.6)	1.34(1.19-1.48)	1.48(0.91-2.25)	1.22(0.76-1.8)	-0.74(-0.83--0.66)	54.87(34.05-82.81)	45.57(29.64-66.04)	-0.69(-0.78--0.6)
	Male	1.81(1.51-2.16)	2.34(1.96-2.81)	1.27(1.1-1.44)	62.41(54.31-72.09)	72.79(63.66-83.33)	0.96(0.82-1.1)	1.4(0.86-2.17)	1.46(0.9-2.2)	0.17(0.07-0.27)	48.39(31.32-73.1)	52.91(33.98-77.63)	0.34(0.24-0.45)

Abbreviation: ASIR, age-standardized incidence rate; ASPR, age-standardized prevalence rate; ASDR, age-standardized death rate; CI, confidence interval; DALY, disability adjusted life-year; EAPC, estimated annual percentage change; SDI, socio-demographic index; UI, uncertainty interval

Table S3. The incidence, prevalence, deaths, DALYs and their change percent of type 1 diabetes related CKD in 204 countries and territories.

Location	Sex	Incident cases (95%UI)		Change percent (%) (95%UI)	Prevalent cases (95%UI)		Change percent (%) (95%UI)	Deaths (95%UI)		Change percent (%) (95%UI)	DALYs (95%UI)		Change percent (%) (95%UI)
		1990	2019		1990	2019		1990	2019		1990	2019	
Afghanistan	Both	190.11(69.18-418.11)	915.62(305.17-2071.49)	37.21(22.55-48.91)	3900.2(2275.23-6352.4)	19620.89(10683.48-33249.57)	52.7(34.57-69.01)	173.77(99.19-283.8)	367.92(219.53-613.25)	53.13(7.8-107.03)	6737.45(3972.42-10707.07)	16330.28(10026.5-27080.5)	65.64(12.74-135.51)
Afghanistan	Female	91.47(26-222.23)	422.53(114.16-1045.79)	35.33(20.85-48.19)	1992.65(1078.3-3454.03)	9635.67(4693.33-17570.41)	51.63(34.09-69.34)	92.26(52.38-157.52)	202.87(111.22-359.37)	53.92(-1.29-115.91)	3873.42(2176.62-6583.1)	9250.69(5118.09-16392.06)	64.71(1.86-140.3)
Afghanistan	Male	98.65(36.93-212.9)	493.09(175.12-1061.83)	38.46(19.37-54.26)	1907.55(1069-3224.54)	9985.22(5233.63-17677.1)	53.77(31.3-74.35)	81.51(42.72-139.11)	165.06(92.03-278.41)	51.09(4.69-112.48)	2864.04(1585.51-4705.87)	7079.59(4168.54-11691.26)	67.4(12.94-146.8)
Albania	Both	47.61(20.29-103.55)	61.48(36.3-104.63)	70.75(26.96-158.44)	2035.09(1316.64-3102.74)	2646.09(1880.17-3891.84)	55.95(34.23-84.34)	6.32(3.82-9.68)	6.49(3.51-11.06)	-21.02(-38.4--0.29)	308.56(202.17-456.12)	303.81(195.31-471.03)	23.1(0.55-49.31)
Albania	Female	17.12(6.09-39.82)	23.62(11.14-45.05)	76.24(30.39-171.98)	1033.37(608.09-1689.77)	1450.93(934.68-2185.57)	63.44(40.75-93.87)	2.94(1.82-4.41)	3.05(1.63-5.1)	-21.83(-41.65-2.34)	143.31(91.91-209.81)	138.47(89.24-211.2)	19.09(-8.13-50.8)
Albania	Male	30.49(12.55-62.51)	37.86(20.85-64.74)	70.09(25.02-160.48)	1001.73(601.88-1618.72)	1195.17(795.65-1782.12)	47.42(23.95-78.97)	3.38(2-5.32)	3.45(1.82-5.99)	-20.46(-39.83-3.51)	165.25(105.24-245.26)	165.34(104.09-257.77)	26.6(0.23-54.31)
Algeria	Both	394.13(137.47-962.92)	1078.05(527.84-2135.81)	67.34(31.79-159.71)	10123.52(5672.18-18345.67)	29799.54(19259.54-47529.33)	76.03(46.75-118.81)	152.71(91.75-249.95)	306.3(182.77-476.9)	53.43(21.22-89.5)	6352.37(3893.73-10172.25)	12720.09(8003.38-19347.61)	92.39(48.61-146.67)
Algeria	Female	179.11(52.27-474.58)	440.48(173.35-974.83)	49.85(21.2-120.51)	5170.5(2744.81-9584.87)	15273.82(9339.83-24803.41)	77.2(45.7-119.14)	82.34(47.33-153.14)	167.03(94.68-270.33)	46.72(9.08-92.04)	3573.85(2031.19-6548.46)	7057.45(4374.51-11112.94)	77.87(29.3-143.21)
Algeria	Male	215.02(74.14-498.71)	637.57(309.51-1227.16)	82.15(39.19-191.24)	4953.02(2536.52-9012.52)	14525.71(8836.19-24049.97)	74.83(45.16-122.3)	70.38(41.6-111.03)	139.27(81.51-227.88)	58.66(18.59-118.5)	2778.52(1745.81-4195.48)	5662.64(3500-9012.29)	107.86(50.73-192.37)
American Samoa	Both	0.75(0.2-1.93)	0.85(0.24-2.15)	-6.61(-14.77-7.14)	15.54(7.96-27.99)	21.77(11.61-39.61)	20.31(13.34-28.66)	1.05(0.72-1.43)	2.63(1.65-3.92)	46.47(21.14-77.38)	45.73(32.68-61.07)	105.13(68.91-153.61)	74.78(42.04-118.37)
American Samoa	Female	0.29(0.07-0.81)	0.36(0.09-0.96)	2.45(-10.41-20.71)	6.95(3.64-13.17)	10.15(5.49-18.9)	22.96(13.34-35.14)	0.38(0.25-0.54)	1.17(0.73-1.75)	53.66(21.7-88.83)	16.54(11.23-23.14)	46.68(30.32-67.95)	96.53(51.97-150.64)
American Samoa	Male	0.46(0.12-1.16)	0.49(0.14-1.2)	-12.08(-20.98-2.26)	8.6(4.2-16.56)	11.62(5.85-22.59)	18.51(9.89-27.8)	0.67(0.44-0.93)	1.46(0.92-2.18)	42.53(9.87-83.7)	29.19(20.17-40.05)	58.46(37.54-85.92)	63.26(23.94-115.72)
Andorra	Both	0.73(0.48-1.06)	1.9(1.3-2.7)	75.01(46.36-105.48)	40.15(29.53-54.66)	83.25(62.96-108.03)	32.83(14.37-51.66)	0.12(0.07-0.22)	0.27(0.15-0.45)	-12.53(-29.36-9.1)	4.78(3.13-7.13)	9.71(6.32-14.06)	13.07(-3.33-33.15)
Andorra	Female	0.27(0.15-0.46)	0.71(0.41-1.16)	71.31(42.6-103.14)	19.8(13.14-29.19)	42.89(29.29-61.23)	34.49(15.08-55.98)	0.05(0.03-0.1)	0.14(0.07-0.24)	-20.69(-39.9-3.77)	2(1.32-3.09)	4.51(2.89-6.63)	14.84(-7.49-39.73)
Andorra	Male	0.46(0.27-0.71)	1.19(0.75-1.83)	79.42(44.73-118.33)	20.35(13.73-29.62)	40.36(28.88-55.59)	30.91(11.41-51.86)	0.07(0.04-0.12)	0.13(0.07-0.22)	-10.66(-30.5-20.52)	2.77(1.73-4.2)	5.2(3.36-7.53)	11.65(-8.14-37.36)
Angola	Both	117.82(36.04-297.44)	405.09(128.63-1010.67)	23.52(15.14-34.32)	3064.31(1726.67-5110.53)	11684.15(6622.2-19710.81)	32.94(21.98-44.63)	65.52(38.67-105.15)	162.65(90.3-254.57)	128.95(79.61-194.35)	2704.19(1597.81-4247.48)	6817.65(3964.26-10179.31)	154.56(97.37-240.21)
Angola	Female	40.94(10.55-109.05)	143.88(40.56-376.6)	23.67(12.13-42.44)	1552.46(876.71-2593.96)	6535.94(3811.68-10956.35)	40.55(27.2-56.1)	24.73(9.04-43.79)	67.53(25.2-113.49)	141.73(77.23-236.8)	1071.88(408.14-1864.52)	2892.38(1157.34-4709.55)	162.91(88.46-276.47)
Angola	Male	76.88(22.96-189.89)	261.21(77.84-647.3)	24.76(13.3-36.68)	1511.85(784.75-2765.29)	5148.21(2570.29-9594.26)	24.17(8.49-38.97)	40.79(23.91-64.23)	95.13(57.62-145.85)	122.41(64.92-205.9)	1632.31(972.39-2504.78)	3925.28(2432.16-5736)	150.52(81.42-243.75)
Antigua and Barbuda	Both	0.77(0.26-1.85)	1.46(0.68-3)	27.23(5.65-90.46)	26.34(14.18-49.43)	61.37(35.91-111.25)	58.74(42.54-83.56)	0.78(0.52-1.12)	2.03(1.25-3.13)	83.46(56.33-118.83)	29.03(20.49-39.72)	73.17(46.81-108.06)	71.45(43.68-108.61)
Antigua and Barbuda	Female	0.26(0.07-0.64)	0.48(0.19-1.05)	26.81(4.86-88.95)	11.34(5.96-21.12)	25.67(14.58-44.35)	56.07(38.2-82.9)	0.3(0.2-0.43)	0.97(0.58-1.5)	120.14(76.22-171.67)	11.53(8-15.79)	34.15(21.11-50.94)	92.78(50.86-143.43)
Antigua and Barbuda	Male	0.51(0.19-1.19)	0.98(0.46-2.06)	26.35(3.06-91.88)	15(7.55-29.36)	35.7(19.39-67.23)	60.08(42.1-85.83)	0.48(0.32-0.69)	1.06(0.67-1.65)	61.18(37.6-91.59)	17.5(12.03-24.53)	39.02(25.2-57.63)	58.06(33.25-90.35)
Argentina	Both	421.91(214.09-793.66)	621.5(357.66-1073.59)	8.27(-6.43-33.91)	22053.68(14358.29-32809.1)	32855.69(21947.84-47871.51)	10(-4-27.19)	322.01(201.59-472.27)	529.47(327.18-799.13)	18.71(5.91-33.13)	11708.22(7628.46-16574.68)	17770.68(11497.68-25191.35)	27.34(15.03-41.35)
Argentina	Female	139.83(63.41-301.35)	229.21(115.95-439.32)	19.87(-0.97-55.89)	10847.37(6614-16582.14)	17732.35(11197.31-27078.96)	19.79(0.72-42.53)	129.77(82.46-190.7)	220.45(137.1-335.88)	13.02(-2.35-29.5)	4737.9(3125.68-6732.51)	7329.77(4825.4-10412.27)	24.05(9.63-40.31)
Argentina	Male	282.07(139.05-539.18)	392.29(205.02-674.49)	2.75(-11.3-24.39)	11206.31(6910.61-17158.28)	15123.34(9851.2-22503.18)	0.46(-12.69-16.26)	192.24(117.26-287.77)	309.01(188.81-470.04)	24.51(9.91-41.56)	6970.32(4458.94-9979.44)	10440.9(6651.46-15056.68)	30.78(16.88-47.29)
Armenia	Both	44.55(17.77-98.47)	56.86(28.89-106.04)	40.95(14.37-98.83)	1962.55(1297.4-2937.9)	2539.76(1766.83-3625.58)	45.48(29.64-66.54)	1.58(0.98-2.37)	9.64(5.88-14.59)	417.76(345.66-502.62)	107.23(70.71-151.68)	406.74(269.3-582.89)	371.53(276.97-481.18)
Armenia	Female	18.89(5.77-45.51)	20.09(7.89-41.81)	14.94(-6.53-64.6)	971.69(576.44-1544)	1266.92(788.51-1958.75)	44.14(25.26-69.77)	0.58(0.36-0.89)	3.58(2.19-5.58)	399.56(322.59-495.67)	41.44(27.18-59.42)	144.78(95.61-203.33)	315.77(218.06-433.82)
Armenia	Male	25.66(10.57-55.61)	36.77(18.53-67.19)	62.05(31.17-127.23)	990.85(607.89-1588.68)	1272.83(830.51-1947.73)	47.08(28.22-74.11)	0.99(0.6-1.51)	6.07(3.65-9.23)	435.48(357.26-534.44)	65.79(43.13-93.38)	261.97(172-375.63)	414.09(314.7-532.38)
Australia	Both	295.26(195.26-433.45)	694.74(467.1-987.77)	62.04(35.83-94.33)	13864.21(9664.34-19372.66)	28371.38(20705.23-38334.7)	40.3(21.25-62.93)	14.8(12.31-18.57)	29.9(16.87-49.2)	39.15(-13.84-113.09)	812.11(636.02-1056.59)	1793.83(1195.82-2580.88)	65.44(26.93-114.94)
Australia	Female	133.63(74.03-222.72)	286.51(162.85-471.5)	47.08(21.38-78.77)	7954.19(5199.32-12054.12)	16082.31(11043.16-23159.82)	37.67(16.51-62.61)	7.41(5.77-9.92)	13.48(7.48-22.04)	20.07(-25.36-81.78)	396.24(298.55-537.23)	821.41(533.22-1198.86)	46.89(13.47-90.72)
Australia	Male	161.63(95.7-260.79)	408.23(257.05-630.48)	74.62(42.74-116.31)	5910.02(3806.8-8669.48)	12289.07(8631.06-17306.3)	43.59(21.33-71.13)	7.39(6.34-8.81)	16.43(9.27-27.5)	58.93(-3.11-151)	415.87(325.79-538.48)	972.42(645-1424.89)	84.14(37.42-147.28)
Austria	Both	97.04(64.25-143.86)	219.91(153.42-307.35)	100.94(62.68-156.09)	4843.89(3453.38-6788)	9286.77(6975.03-12172.76)	65.72(39.69-100.21)	14.09(8.07-23.44)	39.33(22.03-66.15)	176.68(124.68-248.96)	550.14(364.34-791.12)	1159.13(761.91-1664.44)	116.25(88.54-151.46)
Austria	Female	38.08(20.67-62.27)	86.84(50.45-138.07)	106.43(66.28-156.75)	2524.44(1626.22-3715.56)	5034.84(3375.87-7263.39)	77.37(47.35-115.06)	7.59(4.23-13.04)	20.66(11.1-36.22)	180.97(118.03-265.18)	252.67(167.14-370.39)	538.86(357.58-786.84)	120.37(90.3-158.75)
Austria	Male	58.96(34.28-94.72)	133.07(83.7-201.06)	95.55(53.17-155.43)	2319.45(1528.89-3376)	4251.93(3095.74-5696.24)	53.56(26.78-93.13)	6.49(3.71-10.35)	18.66(10.32-30.85)	172.25(113.84-250.18)	297.47(189.69-431.35)	620.27(392-889.38)	112.54(78.37-158.63)
Azerbaijan	Both	131.07(51.41-303.18)	241.89(116.52-493.33)	25.75(7.08-64.31)	4402.89(2879.15-7017.85)	9227.42(6235.25-13897.7)	49.49(33.02-69.07)	20.93(13.14-31.22)	52.43(32.09-81.72)	93.03(60.32-136.95)	1078.37(692.59-1565.24)	2497.77(1602.52-3733.62)	134.71(95.51-189.3)
Azerbaijan	Female	53.62(16.39-138.44)	90.55(34.57-210.47)	16.65(-0.41-52.96)	2150.23(1311.55-3716.14)	4479.46(2773.5-7241.55)	51.81(32.65-75.41)	8.82(5.51-13.21)	22.89(14-35.79)	102.75(52.74-161.54)	460.2(292.66-675.71)	1073.5(674.05-1636.58)	134.96(78.05-206.02)
Azerbaijan	Male	77.44(30.34-176.58)	151.34(70.9-299.71)	31.25(10.09-77.07)	2252.66(1383.03-3680.56)	4747.96(3011.61-7400.03)	46.96(28.37-68.64)	12.11(7.49-18.3)	29.54(17.34-46.62)	85.58(48.39-137.76)	618.16(392.12-921.42)	1424.27(900.63-2178.11)	134.36(87.01-201.86)

Bahamas	Both	3.09(1.08-7.49)	6.2(2.86-12.93)	32.17(12.28-85.31)	89.97(54.29-152.05)	191.82(129.47-302.6)	43.76(27.71-69.06)	3.12(2.12-4.37)	8.23(5.07-12.34)	37.36(17.93-57.95)	131.33(91.59-181.59)	328.93(213.65-486.88)	51.13(25.23-79.84)
Bahamas	Female	1.12(0.34-2.87)	2.2(0.84-5.07)	27.29(6.17-79.92)	41.12(24.03-70.08)	90.01(58.55-142.94)	45.69(27.35-72.17)	1.43(0.97-2)	3.68(2.29-5.57)	35.47(11.87-60.49)	60.73(42.1-83.63)	145.96(94.67-215.24)	45.94(15.82-78.31)
Bahamas	Male	1.96(0.68-4.5)	4(1.8-8.02)	35.8(13.58-93.55)	48.85(27.18-86.86)	101.81(63.71-166.74)	42.51(24.21-72.74)	1.69(1.13-2.42)	4.54(2.8-6.88)	39.02(20.29-61.37)	70.6(49.03-98.4)	182.97(118.13-273.78)	55.61(31.62-83.86)
Bahrain	Both	6.24(2.23-13.94)	30.58(17.08-51.51)	75.82(20.06-224.77)	192.75(119.18-314.96)	934.1(646.81-1357.34)	68.69(41.73-110.32)	2.08(1.3-3.18)	9(5.37-14.21)	98.62(63.35-130.93)	86.88(57.02-126.14)	391.06(249.14-588.01)	96.28(58.21-136.42)
Bahrain	Female	2.54(0.72-6.39)	8.72(3.61-18.6)	35.83(2.96-131.97)	86.39(48.98-156.05)	381.53(239.84-605.86)	71.54(44.44-113.11)	0.85(0.52-1.31)	2.86(1.71-4.54)	58.93(21.52-98.39)	34.63(22.41-51.3)	121.54(79.28-179.59)	64.33(23.29-110.95)
Bahrain	Male	3.7(1.37-8.01)	21.85(11.85-37.68)	97.42(32.94-256.76)	106.35(64.58-170.46)	552.57(375.16-804.51)	67.89(39.19-114.66)	1.24(0.76-2.01)	6.14(3.54-9.92)	124.93(80.77-173.92)	52.25(34.36-79.76)	269.53(170.17-421.7)	114.22(68.26-162.67)
Bangladesh	Both	1000.56(334.11-2397.32)	1743.16(800.48-3705.23)	29.86(7.16-90.69)	34653.34(20757.6-57366.18)	75365.1(49175.74-116109.26)	50.12(34.22-72.52)	358.05(201.02-559.74)	705.26(393.92-1110.28)	160.66(101.72-253.08)	14364.71(8449.64-21344.28)	26753.39(16231.72-40976.68)	233.98(151.84-356.34)
Bangladesh	Female	229.16(69.97-561.02)	484.08(191.02-1083.37)	53.13(22.3-120.87)	13371.84(7542.21-22546.45)	32631.6(19380.6-52823.05)	60.68(41-87.67)	102.22(54.7-166.8)	247.09(136.19-392.68)	228.64(144.44-393.15)	3867.88(2139.38-6218.2)	8844.8(5200.7-13793.68)	302.46(190.12-502.14)
Bangladesh	Male	771.4(254.08-1844.04)	1259.08(574.94-2637.32)	25.24(2.01-87.04)	21281.51(11997.75-36546.67)	42733.5(26620.6-67778.3)	45.3(29.24-70.68)	255.84(135.25-409.86)	458.17(251.9-732.98)	132.12(72.77-231.21)	10496.83(5861.06-15923.48)	17908.59(10889.84-27762.49)	211.47(130.48-348.96)
Barbados	Both	2.38(0.88-5.67)	3.82(1.89-7.82)	28.72(5.09-88.65)	72.75(44.4-118.42)	121.59(84.54-183.12)	41.43(23.8-69.15)	2.75(1.82-3.85)	5.97(3.66-9.41)	58.5(34.24-84.1)	100.75(69.87-133.11)	204.94(129.92-307.34)	60.13(34.05-89.43)
Barbados	Female	0.83(0.24-2.26)	1.31(0.51-3.22)	26.29(3.87-85.95)	33.04(20.01-54.27)	54.61(36.06-84.62)	40.34(21.78-65.49)	1.23(0.82-1.74)	2.98(1.78-4.75)	82.33(52.79-114.24)	44.17(30.48-60.41)	99.79(62.44-152.55)	75.71(44.35-112.47)
Barbados	Male	1.54(0.56-3.59)	2.51(1.22-5.08)	29.97(3.06-92.83)	39.72(22.85-69.01)	66.98(44.08-106.72)	42.21(21.74-75.46)	1.53(1.02-2.14)	2.99(1.82-4.66)	39.28(19.27-62.73)	56.58(39.06-75.25)	105.15(67.07-155.47)	48.15(24.33-74.96)
Belarus	Both	115.6(54.62-233.19)	207.71(119.83-350.53)	97.45(53.23-168.92)	7047.61(4827.7-10242.95)	12964.62(9331-18153.56)	102.14(74.28-135.64)	16.62(10.45-24.46)	31.61(18.89-49.36)	69.57(44.62-97.81)	545.36(377.86-754.06)	1166.86(767.55-1686.8)	124.17(97.24-154.99)
Belarus	Female	47.65(19.67-108.82)	78.62(38.35-150.76)	78.87(40.01-140.36)	3712.36(2327.36-5739.56)	7174.4(4567.28-11034.46)	110.86(80.77-147.58)	9.19(5.66-14.23)	15.68(9.13-24.89)	53.01(27.71-81.93)	262.21(180.28-371.08)	502.26(320.45-740.41)	103.15(73.05-137.93)
Belarus	Male	67.95(31.33-138.29)	129.09(70.68-224.99)	112.18(63.03-197.06)	3335.25(2109.51-5047.6)	5790.22(3972.35-8376.46)	92.36(65.07-131)	7.43(4.73-10.84)	15.93(9.58-25.18)	90.01(60.51-125.24)	283.15(193.88-393.73)	664.59(436.77-975.98)	143.44(113.62-179.95)
Belgium	Both	123.4(82.26-184.84)	224.98(157.41-317.02)	56.31(32.03-87.64)	5612.66(4109.34-7594.36)	9061.49(6835.87-12022.17)	40.29(22.97-61.36)	21.77(12.21-36.42)	25.61(14.04-44.27)	6.31(-13.4-34.49)	786.66(515.52-1153.26)	937.22(630.12-1343.44)	15.61(2.26-31.22)
Belgium	Female	47.08(26.15-79.61)	82.62(46.02-136.08)	50.75(26.85-82.27)	2852.24(1957.77-4234.41)	4647.69(3177.19-6615.55)	42.61(22.36-65.98)	11.84(6.49-20.92)	13.24(7.02-23.71)	-1.91(-22.75-27.19)	374.05(247.56-558.64)	421(273.22-615.6)	5.22(-9.23-22.16)
Belgium	Male	76.32(46.75-124.3)	142.36(91.1-213.12)	59.55(30.93-97.67)	2760.42(1878.38-3953.13)	4413.81(3184.15-6008.89)	37.86(16.3-63.79)	9.93(5.72-16.45)	12.37(6.81-20.99)	15.98(-5.4-46.73)	412.61(266.32-595.92)	516.21(340.04-740.32)	25.84(10.32-44.88)
Belize	Both	3.11(1.03-7.59)	8.77(3.51-19.75)	27.95(12.76-63.17)	72.09(40.29-135.14)	229.96(139.2-407.72)	44.35(31.28-64.01)	1.28(0.85-1.79)	8.82(5.73-12.81)	214.74(173.05-265.96)	52.32(36.81-70.64)	365.35(250.62-514.03)	261.21(206.23-324.36)
Belize	Female	1.14(0.29-3.09)	3.16(0.98-8.16)	24.82(9.41-59.61)	30.52(16.08-55.67)	100.25(57.57-172.83)	45.74(30.65-69)	0.64(0.42-0.9)	4.09(2.64-5.92)	251.24(199.54-314.26)	25.73(17.71-35.24)	170.33(115.83-239.59)	270.57(204.86-341.67)
Belize	Male	1.97(0.69-4.93)	5.6(2.26-12.83)	30.41(13.18-68.13)	41.57(22.44-79.56)	129.71(75.65-237.55)	44.02(29.52-65.57)	0.64(0.43-0.93)	4.73(3.07-6.9)	194.55(148.48-257.75)	26.59(18.62-36.67)	195.02(132.44-277.34)	256.36(196.23-336.76)
Benin	Both	91.79(27.56-216.39)	261.77(79.91-620.02)	12.79(5.36-21.88)	2187.74(1299.78-3622.78)	6507.46(3781.35-10885.4)	15.82(5.72-26.06)	39.86(25.77-58.05)	105.4(61.8-162)	89(47.07-140.35)	1603.27(1048.85-2287.25)	4503.14(2695.27-6892.58)	109.14(61.34-166.84)
Benin	Female	34.31(9.65-88.03)	99.41(28.47-251.2)	15.25(4.69-26.76)	1106.09(653.41-1782.42)	3320.34(1885.6-5420.32)	17.39(4.35-32.07)	19.12(12.09-28.32)	50.18(28.58-80.27)	90.28(37.7-155.51)	791.56(502.28-1167.03)	2164.18(1224.24-3474.06)	107.02(48.93-180.14)
Benin	Male	57.48(16.43-136.21)	162.37(48.4-380.8)	11.03(1.58-22.24)	1081.65(584.16-1948.78)	3187.12(1727.29-5724.59)	14.21(4.28-24.75)	20.74(12.9-31.11)	55.22(30.48-90.4)	88.02(39.58-153.21)	811.7(519.03-1183.66)	2338.96(1341.58-3756.69)	111.45(56.75-189.29)
Bermuda	Both	0.59(0.32-1.11)	1.31(0.85-2)	89.5(39.21-173.3)	23.07(16.42-32.59)	43.27(33.56-55.65)	72.9(48.62-103.16)	0.55(0.36-0.81)	0.71(0.42-1.12)	8.05(-7.44-24.84)	20.77(13.86-28.61)	25.32(16.28-37.73)	19.65(0.42-39.69)
Bermuda	Female	0.2(0.09-0.41)	0.4(0.22-0.71)	73.38(26.95-156.03)	11.06(7.45-16.59)	19.53(14.06-26.81)	61.93(37.76-92.76)	0.21(0.14-0.32)	0.26(0.15-0.42)	5.07(-11.79-25.77)	7.94(5.3-11.38)	9.28(5.96-13.83)	13.13(-7.48-37.03)
Bermuda	Male	0.39(0.19-0.76)	0.91(0.55-1.43)	98.4(44.89-185.79)	12.02(8.13-18.27)	23.75(17.59-31.95)	83.24(52.78-122.25)	0.34(0.21-0.5)	0.45(0.27-0.71)	9.7(-7.58-28.28)	12.83(8.4-17.9)	16.04(10.24-24.18)	24.14(4.05-45.66)
Bhutan	Both	7.98(2.72-18.4)	11.32(4.82-23.94)	29.7(9.77-79.56)	245.85(147.12-412.3)	419.22(268.2-665.99)	40.91(26.14-60.38)	3.91(2.06-6.76)	9.17(5.11-14.84)	212.31(136.82-347.48)	156.41(86.54-260.75)	336(193.61-547.06)	321.35(212.04-509.04)
Bhutan	Female	2.97(0.89-7.67)	4.06(1.49-9.43)	28.34(7.09-80.11)	103.89(57.69-170.85)	191.44(111.97-301.38)	48.91(29.29-72.79)	1.95(0.89-3.36)	4.4(2.1-7.53)	229(137.88-404.71)	76.97(36.84-130.56)	159.22(78.19-266.19)	320.49(200.27-575.76)
Bhutan	Male	5.01(1.7-12.14)	7.26(2.96-15.64)	29.12(8.31-78.12)	141.96(81.87-242.05)	227.78(139.99-375.74)	35.35(20.56-55.74)	1.96(0.93-3.87)	4.77(2.5-8.04)	198.69(103.41-343.04)	79.45(41.17-149.71)	176.78(97.19-300)	322.24(190.86-526.43)
Bolivia (Plurinational State of)	Both	94.68(32.43-236.24)	213.3(86.16-492.63)	19.67(7.65-47.84)	2367.05(1390.71-4252.87)	6244.55(3909.89-10646.47)	42.15(29.64-59.9)	76.21(48.06-116.36)	226.18(127.27-369.67)	138.87(94.59-187.45)	2911.33(1918.18-4195.34)	7580.48(4451.03-11940.6)	188.05(123.73-266.04)
Bolivia (Plurinational State of)	Female	32.04(9.07-88.72)	74.49(26.05-184.47)	25.07(9.51-62.41)	1108.49(626.26-1934.16)	3156.07(1847.24-5233.69)	55(37.53-79.32)	38.85(24.5-60.25)	116.2(65.8-192.03)	136.61(83.73-197.08)	1502.24(986.12-2267.14)	3904.09(2340.05-6244.43)	181.04(106.45-271.68)
Bolivia (Plurinational State of)	Male	62.65(21.66-149.87)	138.81(55.19-318.26)	16.13(3.32-40.68)	1258.56(687.3-2338.09)	3088.48(1745.85-5575.82)	30.83(19.8-46.61)	37.36(22.57-57.35)	109.98(60.16-185.02)	140.88(93-202.79)	1409.09(886.15-2092.54)	3676.4(2062.85-6044.93)	195.02(125.96-286.19)
Bosnia and Herzegovina	Both	38.09(18.25-75.97)	43.81(25.86-71.78)	72.4(33.83-145.47)	1957.51(1329.54-2967.89)	2084.66(1513.22-2939.46)	44.89(26.19-69.6)	16.14(9.56-25.86)	24.62(13.76-40.58)	17.33(-7.37-53.24)	643.37(404.56-987.92)	784.52(477.58-1229.56)	34.06(12.94-63.09)
Bosnia and Herzegovina	Female	14.61(5.82-32.93)	16.77(8.17-33.14)	66.85(29.41-134.91)	1026.97(659.45-1647)	1155.44(768.46-1715.53)	49.42(28.88-75.57)	8.76(5.21-14.42)	12.23(6.63-20.88)	0.19(-23.72-37.7)	326.22(204.84-505.55)	360.3(219.89-564.73)	14.81(-6.48-45.45)
Bosnia and Herzegovina	Male	23.48(11.27-46.51)	27.04(15.9-44.96)	78.37(34.86-152.75)	930.54(597.15-1427.21)	929.23(655.64-1310.91)	39.34(18.16-67)	7.38(4.29-11.94)	12.38(6.86-20.43)	37.56(5.37-83.1)	317.15(195.93-489.01)	424.23(254.17-673.63)	54.4(26.5-90.7)
Botswana	Both	17.44(6.59-38.49)	42.46(18.85-85.87)	37.99(19.61-71.79)	527.23(323.2-827.71)	1407.25(897.75-2068.91)	51.52(36.3-70.44)	8.21(4.48-13.95)	31.83(17.39-52.84)	89.57(35.42-160.39)	327.33(192.24-549)	1298.75(725.17-2127.6)	120.72(49.6-222.15)
Botswana	Female	5.01(1.55-12.37)	12.78(4.56-28.6)	47.34(26.47-83.57)	250.69(145.27-421.04)	653.49(394.28-1050.18)	51.14(34.52-74.97)	2.74(1.33-5.53)	12.34(6.21-22.39)	113.97(40.91-221.46)	110.69(58.14-213.35)	499.71(262.29-877.12)	149.86(53.03-290.58)

Botswana	Male	12.43(4.81-27.43)	29.68(12.93-60.01)	32.62(12.51-67.78)	276.54(161.38-446.12)	753.76(467.06-1159.82)	51.21(31.76-73.5)	5.48(2.92-9.29)	19.49(10.13-34.2)	78.72(23.84-149.86)	216.64(122.22-367.08)	799.05(423.03-1362.74)	106.35(36.28-202.36)
Brazil	Both	2026.73(1535.99-2749.11)	4260.98(3476.2-5216.07)	54.05(33.12-78.17)	65176.46(54946.66-78523.58)	166971.24(144455.73-194406.0	75.11(57.66-91.67)	1053.1(715.04-1443.19	1859.48(1204.1-2707.66)	23.12(7.28-41.41)	44952.4(31836.02-59742.91)	72083.34(49483.94-99557.74)	46.72(28.68-69.76)
Brazil	Female	781.27(580.15-1076.06)	1907.92(1522.72-2359.82)	75.65(49.13-107.63)	34185.66(28698.06-41370.6)	104184.7(89006.34-123929.04)	105.75(83.39-127.78)	478.75(323.38-653.24)	861.84(554.12-1237.85)	16.57(-0.25-36.01)	20637.55(14530.94-27797.24)	34235.16(23809.42-46667.09)	45.34(25.1-69.63)
Brazil	Male	1245.45(931.8-1705.82)	2353.06(1915.27-2926.36)	41.18(23.04-63.44)	30990.8(25511.04-37972.08)	62786.54(54000.25-73971.06)	40.31(27.47-53.33)	574.35(390.5-783.37)	997.65(639.89-1473.03)	27.98(12.12-47.04)	24314.84(17108.01-32415.83)	37848.18(25844.52-52639.17)	47.46(29.6-69.95)
Brunei Darussalam	Both	3.13(1.13-7.04)	4.75(2.16-9.66)	-5.99(-21.77-30.94)	119.42(75.13-188.67)	254.07(165.26-379.07)	24.97(8.91-43.75)	3.66(2.62-4.96)	8.49(5.65-12.16)	35.1(12.98-58.6)	158.85(116.55-210.79)	338.55(233.11-469.63)	32.49(7.05-58.41)
Brunei Darussalam	Female	0.95(0.29-2.31)	1.62(0.66-3.42)	3.11(-17.95-48.73)	55.43(33.21-88.04)	133.89(82.24-210.31)	39.56(19.67-61.43)	1.57(1.08-2.17)	3.68(2.41-5.4)	22.87(-7.25-54.46)	68.18(47.47-94.53)	143.64(97-204.01)	19.24(-12.08-53.58)
Brunei Darussalam	Male	2.18(0.72-5)	3.14(1.32-6.51)	-9.28(-24.05-25.29)	63.99(37.81-106.93)	120.18(75.45-188.94)	12.02(-1.62-30.35)	2.1(1.45-2.88)	4.82(3.15-6.91)	44.79(18.45-80.2)	90.68(63.76-123.05)	194.92(133.2-271.56)	43.78(14-80.77)
Bulgaria	Both	90.22(49.58-176.95)	146.28(92.88-238.85)	114.82(73.43-173.02)	4009.92(2883.37-5683.09)	5544.62(4198.51-7600.73)	72.52(50.05-99.78)	31.85(19.36-49.44)	62.51(34.96-101.91)	78.42(54.37-106.7)	1312.84(853.41-1934.45)	2263.96(1377.17-3569.28)	96.57(72.21-123.28)
Bulgaria	Female	31.53(14.42-69.17)	51.95(27.01-97.8)	112.97(70.41-172.09)	2013.5(1341.15-3033.23)	2868.47(1960.78-4194.47)	74.87(51.12-103.66)	14.03(8.57-21.76)	26.72(14.85-44.74)	54.86(28.45-84.51)	568.69(367.21-844.02)	935.47(565.95-1484.02)	75.79(48.34-106.38)
Bulgaria	Male	58.69(31.05-113.46)	94.33(57.81-156.99)	118.74(73.03-185.47)	1996.42(1315.19-3035.99)	2676.15(1918.65-3816.45)	70.2(44.32-105.48)	17.82(10.57-27.46)	35.79(20.08-58.41)	101.14(74.4-133.58)	744.15(478.25-1100.92)	1328.49(796.55-2090.29)	114.32(86.86-143.99)
Burkina Faso	Both	145.63(42.09-347.83)	413(119.5-978.04)	30.14(21.8-39.46)	3763.29(2260.27-6012.71)	10978.62(6484.91-17812.72)	24.4(13.7-34.2)	68.4(43.22-106.51)	172.19(106.02-258.99)	96.95(48.28-155.88)	2635.43(1705.91-3952.67)	7220.95(4625.58-10577.35)	120.54(60.28-193.3)
Burkina Faso	Female	55.68(16.21-135.08)	154.66(46.88-378.92)	29.36(18.1-43.28)	1895.55(1088.8-3037.15)	5701.93(3263.56-9327.99)	28.91(16.4-42.85)	32.64(20.14-50.95)	87.51(53.29-132.27)	115.51(53.26-189.18)	1316.94(834.37-2004.89)	3698.22(2284.08-5426.86)	129.66(57.58-215.42)
Burkina Faso	Male	89.95(25.47-215.45)	258.34(71.82-624.48)	29.68(18.41-40.39)	1867.74(975.7-3235.32)	5276.69(2686.31-9443.74)	19.83(6.3-31.61)	35.76(21.64-55.61)	84.68(51.54-128.94)	80.9(31.38-150.53)	1318.49(833.27-1995.5)	3522.74(2147.95-5255.69)	111.97(49.65-197.39)
Burundi	Both	67.53(23.52-167.12)	151.38(57.08-361.29)	12.37(4.02-26.24)	1923.87(1196-3018.81)	5541.96(3438.79-8569.11)	35.94(24.43-49.57)	44.38(27.41-68.64)	63.7(37.53-99.4)	64.29(30.85-106.18)	1740.15(1105.75-2611.02)	2632.68(1634.32-4016.61)	72.36(36.45-123.14)
Burundi	Female	22.69(6.84-57.54)	50.93(16.08-124.64)	16.42(5.38-30.99)	977.2(585.55-1570)	2941.12(1728.42-4783.43)	44.57(28.66-63.51)	20.44(12.27-32.81)	26.5(15.1-42.35)	63.23(20.43-117.05)	830.22(492.9-1338.34)	1121.08(665.28-1755.7)	63.61(18.21-123.23)
Burundi	Male	44.84(14.44-114.97)	100.45(34.8-244.66)	8.23(-1.75-24.05)	946.68(526.85-1626.48)	2600.84(1491.18-4399.65)	27.28(15.75-41.3)	23.94(14.26-37.94)	37.2(21.41-59.34)	64.38(25.77-119.54)	909.93(552.32-1413.54)	1511.6(913.07-2319.83)	79.3(34.99-144.64)
Cabo Verde	Both	5.43(1.69-12.68)	9.35(3.64-20.13)	10.1(-3.07-41.75)	154.57(90.34-256.64)	319.06(195.04-497.19)	30.78(16.77-49.8)	1.79(1.21-2.55)	5.21(3.32-7.59)	107.81(65.13-158.41)	72.05(49.79-99.3)	200.06(128.63-294.56)	172.52(113.06-244.36)
Cabo Verde	Female	2.02(0.57-5.13)	3.19(1.1-7.47)	6.18(-8.24-39.35)	78.16(44.53-126.26)	152.25(91.3-234.69)	29.99(14.95-49.41)	0.78(0.52-1.13)	1.78(1.15-2.62)	79.97(40.11-126.53)	31.91(21.38-45.06)	67.43(44.71-96.83)	130.99(76.85-195.51)
Cabo Verde	Male	3.4(1.05-8.22)	6.16(2.34-13.7)	9.18(-5.34-42.1)	76.41(40.11-142.07)	166.81(94.79-278.75)	30.82(14.76-56.53)	1.01(0.64-1.47)	3.43(2.12-5.11)	122.83(68.82-192.96)	40.14(27.03-56.45)	132.63(83.66-199.33)	196.18(122.69-300.03)
Cambodia	Both	132.36(41.37-334.73)	181.94(62.68-448.84)	-4.65(-13.65-10.62)	3724.57(2270.83-6217.3)	6710.55(4211.71-10846.7)	13.93(5.1-24.48)	193.87(138.65-264.21)	304.99(204.9-432.64)	62.42(30.52-104.95)	9304.37(6704.44-12439.3)	13554.64(9275.04-18782.27)	114.05(65.71-175.01)
Cambodia	Female	60.55(15.57-165.99)	77.69(22.43-206.86)	-8.65(-18.5-7.36)	1993.93(1195.82-3374.64)	3552.99(2192.82-5893.87)	14.99(3.61-28.3)	112.52(76.19-158.65)	153.99(101.89-221.46)	39.11(5.51-83.45)	5420.27(3684.74-7598.78)	6708.52(4574.43-9448.03)	80.32(32-143.49)
Cambodia	Male	71.81(20.81-181.87)	104.25(33.81-252.64)	-2(-13.28-14.28)	1730.64(963.17-2946.69)	3157.56(1842.11-5302.68)	12.86(3.89-25.04)	81.35(56.38-113.41)	151.01(100.26-213.06)	94.34(46.11-161.02)	3884.1(2721.01-5324.85)	6846.13(4607.57-9473.21)	160.99(94.21-255.42)
Cameroon	Both	262.52(86.28-625.84)	832.77(284.49-1974.86)	18.62(10.8-30.15)	6124.05(3552.91-10359.21)	21273.81(12443.04-36894.47)	27.11(17.6-36.24)	147.33(92.24-216.09)	418.29(245.58-657.25)	59.01(21.37-115.87)	6117.13(3866.96-8841.08)	18429.29(11098.44-28484.75)	85.99(35.27-157.75)
Cameroon	Female	109.13(35.75-272.05)	316.33(108.04-786.01)	10.02(1.42-22.77)	3120.61(1800.11-5305.91)	10672.52(6021.58-18795.63)	25.6(13.23-39.08)	85.32(49.81-132.39)	205.93(111.7-326.47)	36.45(-3.44-110.19)	3600.28(2116.99-5544.69)	9143.38(5135.26-14418.55)	55.55(6.42-143.96)
Cameroon	Male	153.39(47.41-368.01)	516.45(167.46-1215.06)	23.91(13.54-38.59)	3003.44(1663.68-5525.01)	10601.29(5773.99-19514.77)	28.67(16.13-41.12)	62.01(37.16-98.32)	212.36(122.99-342.35)	90.35(45.5-147.99)	2516.85(1567.92-4017.12)	9285.91(5512.75-14690.53)	129.47(69.98-206.99)
Canada	Both	941.09(627.47-1343.34)	1390.89(976.56-1896.74)	10.49(-10.67-38.71)	53432(37461.86-75575.3)	64318.27(47412.98-86548.11)	-11.49(-27.65-7.36)	39.45(23.74-63.4)	74.44(44.47-121.63)	24.64(8.13-43.65)	2483.81(1680.62-3458.48)	3775.83(2583.49-5254.52)	10.63(-7.4-31.24)
Canada	Female	381.44(219.12-616.95)	552.53(332.85-869.89)	8.9(-13.14-37.62)	30443.19(19232.99-46649.8)	37239.5(24937.63-54409.97)	-9.68(-27.15-10.56)	20.04(11.91-33.39)	38.15(22.53-62.24)	18.48(0.55-41.38)	1148.15(765.45-1658)	1756.59(1168.78-2497.85)	5.91(-11.95-26.53)
Canada	Male	559.65(357.1-840.09)	838.35(541.18-1214.03)	11.27(-11.55-43.13)	22988.81(15613.2-33653.73)	27078.77(20071.34-37311.79)	-13.77(-30.56-6.56)	19.42(11.66-30.66)	36.29(21.22-58.05)	30.27(10.98-53.46)	1335.66(858.32-1940.83)	2019.24(1338.53-2835.16)	15.26(-8.07-43.64)
Central African Republic	Both	30.89(9.69-80.75)	66.28(21.32-171.94)	13.09(4.73-23.49)	775.31(451.93-1299.16)	1796.77(1049.73-3004.45)	20.94(12.28-30.6)	24.9(14.02-40.49)	49.3(27.74-80.48)	37.8(12.96-69.56)	994.23(586.09-1547.71)	2023.77(1184.67-3263.39)	50.27(22.2-89.16)
Central African Republic	Female	10.97(3.07-29.77)	23.75(6.76-64.42)	15.23(4.29-28.58)	411.36(239.71-681.38)	991.95(573.38-1670.71)	25(12.85-37.72)	10.22(4.74-17.28)	18.96(8.12-33.81)	30.48(-0.29-65.13)	423.51(204.66-705.42)	797.18(362.89-1376.89)	42.01(8.37-83.66)
Central African Republic	Male	19.93(6.08-52.78)	42.53(13.21-116.58)	11.38(0.76-23.92)	363.95(193.61-675.93)	804.83(423.9-1508.83)	16.2(6.9-27.75)	14.67(8.01-24.39)	30.33(16.98-49.58)	42.62(7.41-93.16)	570.72(320.38-939.2)	1226.6(704.39-2021.38)	55.77(16.64-113.41)
Chad	Both	111.47(35.02-283.9)	360.34(108.34-936.45)	18.01(8.44-26.74)	2644.91(1580.13-4581.41)	8066.75(4485.67-14774.91)	13.54(1.87-23.72)	49.32(28.51-80.87)	116.85(69.4-182.89)	49.33(12.64-97.25)	1928.87(1145.94-3124.63)	4942.5(2978.35-7461.79)	59.49(16.19-113.85)
Chad	Female	41.12(11.96-107.23)	123.48(33.64-324.49)	12.26(0.82-22.87)	1316.5(768.75-2240.97)	3882.93(2115.32-6910.11)	11.88(-0.95-24.17)	24.66(13.58-47.61)	48.4(27.46-84.34)	30.61(-4.31-81)	997.56(550.74-1872.86)	2120.94(1227.54-3671.93)	35.68(-3.83-91.34)
Chad	Male	70.35(20.23-182.8)	236.86(65.67-627.34)	19.79(8.43-30.21)	1328.41(720.66-2479.55)	4183.82(2160.88-8519.27)	14.97(0.86-27.42)	24.67(14.39-41.7)	68.45(39.19-107.76)	67(18.3-126.84)	931.3(555.18-1515.82)	2821.56(1683.66-4395.98)	84.39(29.14-152.83)
Chile	Both	134.78(63.38-275.16)	261.21(150.53-441.57)	47.46(15.4-112.54)	7587.32(4835-11408.3)	13117.47(8826.59-18905.53)	24.38(6.95-46.77)	58.11(35.97-87.56)	142.98(86.26-227.45)	65.07(41.17-94.15)	2248.69(1471.09-3209.06)	4637.26(2934.31-6843.47)	64.95(40.44-92.78)
Chile	Female	49.62(19.99-110.75)	97.77(46.77-185.52)	48.37(13.67-114.55)	3913(2323.42-6280.64)	6979.22(4352.35-10671.09)	28.43(7.35-53.4)	25.51(15.91-39.08)	64.79(39.15-103.16)	57.81(36.06-87.12)	980.51(642.02-1412.58)	2051.5(1313.81-3041.91)	57.37(35.39-84.29)
Chile	Male	85.17(38.6-174.04)	163.44(90.55-286.65)	47.67(14.82-116.03)	3674.33(2179.67-5863.24)	6138.26(3936.3-9425.53)	20.08(2.22-44.99)	32.6(19.95-49.39)	78.2(46.19-124.15)	71.59(44.46-102.75)	1268.18(829.21-1829.09)	2585.76(1607.96-3807.79)	72.44(45.37-103.89)
China	Both	9148.36(7027.1-11901.97)	9248.57(7636.54-11248.47	-12.88(-23.61-0.13)	366455.06(311976.36-427566.7	573858.75(494819.02-664551.7	30.4(16.85-44.64)	11182.79(7847.12-149	12679.09(8458.11-1833	-11.23(-26.66-6.29)	508302.9(362794.67-673710.2	493680.72(334381.64-689359.4	4.84(-15.2-29.45)

China	Female	3395.99(2504.56-4592.3)	3094.11(2497.24-3892.04)	-22.14(-32.74--9.27)	184358.35(154305.97-217484.6	316969.6(265118.8-375403.05)	41.03(24.12-59.78)	5326.28(3686.94-7413.	5469.42(3504.55-8173.8	-12.49(-28.72-7.92)	237508.59(166982.64-327856.	205559.13(136503.59-293055.7	-2.84(-24.82-25.82)
China	Male	5752.37(4472.72-7408.4)	6154.46(5044.16-7386.61)	-7.05(-18.17-6.9)	182096.7(153374.1-214182.12)	256889.15(222749.28-294652.2)	19.21(7.38-31.72)	5856.52(3942.64-7937)	7209.66(4672.1-10851.3	-9.7(-27.28-11.9)	270794.32(183250.17-367471.	288121.58(192457.26-415103.4	11.17(-12.96-40.01)
Colombia	Both	448.37(154.09-1066.22)	706.87(326.71-1501.89)	16.12(-1.73-67.39)	11660.39(6576.11-20107.95)	21781.88(14103.68-33708.09)	28.68(13.06-53.69)	229.67(154.06-320.22)	387.77(225.36-619.27)	15.82(-2.81-37.84)	9519.75(6645.95-12985.23)	13618.59(8367.89-20894.13)	28.47(4.9-57.12)
Colombia	Female	145.61(43.76-377.84)	210.47(86.42-470.67)	4.9(-13.23-60.98)	5280.52(2971.24-9059.86)	9950.99(6203.93-15670.07)	27.71(9.22-54.91)	110.4(74.46-152.76)	184.96(108.6-298.94)	0.99(-16.91-21.25)	4577.93(3171.38-6204.2)	6474.85(4000.87-9960.64)	14(-9.73-42.16)
Colombia	Male	302.76(102.02-743.73)	496.4(213.94-1072.95)	22.61(3.4-73.08)	6379.87(3324.53-11746.86)	11830.89(7004.88-19902.32)	29.99(14-57.48)	119.27(79.09-167.86)	202.81(116.56-330.54)	28.5(8.2-55.4)	4941.82(3419.04-6756.02)	7143.74(4367.67-10946.16)	41.34(17.34-71.74)
Comoros	Both	6.65(2.34-16.62)	10.04(4.52-21.89)	4.34(-10.9-49.19)	196.17(121.96-309.54)	458.67(304.46-659.11)	53.79(33.2-78.18)	3.26(1.34-5.56)	6.07(3.53-9.35)	100.03(47.65-332.57)	122.6(46.18-197.69)	231.64(142.83-349.05)	161.36(84.06-523.4)
Comoros	Female	2.3(0.72-5.94)	3.79(1.5-8.64)	16.64(-4.25-73.48)	99.98(61.09-156.04)	257.05(160.51-392.92)	69.84(45.44-99.85)	1.55(0.62-2.59)	3.22(1.88-5)	109.22(46.54-361.07)	60.45(22.28-99.02)	122.47(73.17-184.49)	169.84(83.87-553.72)
Comoros	Male	4.36(1.45-11.02)	6.24(2.64-14.62)	-3.06(-17.43-36.45)	96.19(54.42-170.72)	201.62(120.17-324.84)	37.25(18.91-59.75)	1.71(0.7-2.96)	2.85(1.64-4.47)	90.05(35.88-323.78)	62.16(23.81-103.6)	109.16(63.62-165.82)	151.62(72.77-517.73)
Congo	Both	29.63(9.35-75.76)	67.76(26.38-155.99)	8.98(-1.96-35.83)	750.01(447.86-1273.75)	2253.28(1433.59-3590.88)	40.72(27.85-57.96)	25.78(14.3-40.92)	50.35(27.91-80.65)	65.92(29.84-120.52)	1025.54(580.69-1603.76)	2037.83(1171.84-3243.08)	83.52(38.14-155.39)
Congo	Female	11.21(3.5-28.18)	27.2(9.96-62.69)	18.99(5.24-47.49)	392.82(229.85-660.5)	1228.99(735.14-2016.87)	48.09(32.22-67.29)	11.96(4.72-20.59)	25.46(9.8-45.01)	75.11(33.85-131.86)	479.88(197.01-794.69)	1048.14(421.59-1811.64)	96.45(43.93-171.61)
Congo	Male	18.42(5.55-47.36)	40.56(14.4-95.01)	1.82(-9.87-27.96)	357.2(192.38-667.42)	1024.29(596.83-1774.05)	32.84(18.03-52.56)	13.82(8.04-22.28)	24.89(14.15-39.4)	57.6(11.39-155.56)	545.66(314.35-866.14)	989.69(575.96-1566.85)	71.78(16.17-188.62)
Cook Islands	Both	0.2(0.06-0.55)	0.19(0.07-0.49)	-9.71(-18.93-16.83)	6.08(3.64-10.53)	6.28(4.04-10.16)	7.34(-3.25-23.84)	0.3(0.2-0.43)	0.53(0.33-0.78)	41.93(6.71-77.24)	12.54(8.46-17.83)	19.57(12.26-28.75)	58.35(15.14-102.94)
Cook Islands	Female	0.08(0.02-0.22)	0.07(0.02-0.2)	-13.11(-23.36-11.16)	2.74(1.5-4.83)	3.03(1.89-4.88)	8.2(-3.35-26.91)	0.13(0.08-0.2)	0.2(0.12-0.31)	18.05(-15.66-55.88)	5.6(3.7-8.2)	7.44(4.36-11.31)	28.58(-15.91-74.76)
Cook Islands	Male	0.13(0.04-0.32)	0.12(0.04-0.28)	-6.22(-18.51-22.53)	3.34(1.91-5.8)	3.24(2.05-5.34)	7.5(-3.3-24.25)	0.17(0.11-0.24)	0.33(0.2-0.49)	61.19(20.87-109.84)	6.94(4.56-10.32)	12.13(7.52-17.98)	83.74(33.08-143.87)
Costa Rica	Both	58.86(20.56-141.38)	85.72(38.75-187.35)	0.11(-14.54-40.02)	1336.57(785.31-2402.48)	2336.26(1528.81-3786.18)	12.95(-2.05-33.47)	14.37(9.37-20.39)	66.77(38.43-107.29)	116.25(86.38-144.18)	568.5(392.08-778.23)	2352.25(1441.65-3697.9)	143.36(99.2-189.93)
Costa Rica	Female	19.45(6.39-51.1)	30.81(13.56-68.04)	5.66(-13.33-60.67)	611.94(359.16-1040.31)	1162.72(745.26-1790.32)	18.3(2.27-42.29)	6.68(4.37-9.41)	28.24(16.17-46.37)	92.9(62.86-122.12)	263.16(180.83-361.8)	981.89(598.27-1569.04)	118.38(73.1-167.19)
Costa Rica	Male	39.41(13.34-97.43)	54.91(23.21-122.8)	-1.22(-15.72-31.13)	724.63(374.11-1422.32)	1173.55(687.75-2153.35)	8.87(-4.87-30.45)	7.69(4.98-11.02)	38.53(21.99-63.44)	136.75(103.23-173.02)	305.34(209.85-421.87)	1370.36(832.79-2149.11)	165.22(117.72-214.74)
Croatia	Both	45.43(26.35-79.77)	78.63(52.41-121.43)	109.59(64.25-176.76)	2329.95(1645.85-3329.79)	3363.08(2481.8-4550.75)	65.43(42.34-95.63)	10.16(5.99-16.47)	12.64(6.69-21.82)	24.24(-3.43-69.28)	424.23(265.92-633.01)	450.51(287.01-677.52)	27.78(8.29-52.17)
Croatia	Female	17.63(9.2-34.21)	31.99(18.06-56.49)	115.59(70.39-184.84)	1294.85(833.8-1979.39)	1944.39(1308.08-2800.64)	71.76(45.63-104.9)	4.89(2.78-8.1)	6.61(3.4-11.63)	28.96(-4.03-80.09)	186.51(119.18-283.36)	207.75(130-314.39)	27.69(5.9-57.2)
Croatia	Male	27.81(15.37-49.2)	46.64(28.55-70.77)	107.49(57.17-183.99)	1035.1(681.18-1530.99)	1418.69(1002.49-1937.26)	57.43(31.64-91.43)	5.27(2.99-8.56)	6.03(3.25-10.52)	19.52(-7.59-57.31)	237.72(146.32-357.98)	242.76(150.97-370.15)	28.26(8.4-56.06)
Cuba	Both	85.04(30.74-213.21)	126.3(67.58-258.31)	33.51(1.44-127.62)	2713.5(1626.49-4588.84)	4124.62(2842.28-6119.55)	43.7(20-83.17)	53.84(36.54-75.04)	126.78(73.7-196.6)	56.74(28.95-86.03)	2226.94(1555.89-3051.49)	4413.81(2691.77-6577.34)	69.25(36.43-107.74)
Cuba	Female	32.29(10.52-86.05)	49.29(22.54-104.83)	36.24(1.69-142.57)	1345.12(783.19-2311.78)	2090.9(1398.9-3111.07)	45.44(20.88-83.08)	23.62(15.91-33.32)	60.61(35.14-94.98)	64.95(30.74-103.9)	988.52(680.74-1360.23)	2052.03(1237.18-3089.58)	78.05(36.75-125.95)
Cuba	Male	52.76(17.63-127.32)	77.01(36.85-154.44)	32.23(-0.83-122.98)	1368.38(742.27-2474.33)	2033.72(1348.99-3205.52)	42(16.91-87.38)	30.22(20.58-42.11)	66.17(38.5-101.41)	49.96(25.51-76.45)	1238.43(860.41-1701.93)	2361.79(1431.98-3525.91)	62.24(31.49-96.68)
Cyprus	Both	7.18(4.19-12.35)	25.06(16.74-36.2)	113(55.66-191.67)	373.21(252.4-535.31)	1190.6(876.65-1609.79)	85.98(55.32-126.7)	6.32(3.59-10.64)	8.3(4.55-13.7)	-13.71(-30.18-6.25)	152.45(97.72-234.83)	214.15(141.32-308.14)	-7.24(-22.43-10.77)
Cyprus	Female	2.68(1.37-4.85)	9.46(5.3-15.69)	110.93(58.05-178.4)	198.68(126.12-312.01)	671.76(443.55-1013.95)	94.4(60.96-142.74)	2.98(1.62-5.22)	3.55(1.9-6.12)	-17.95(-39.62-9.04)	65.03(41.76-102.36)	85.28(56.96-125.62)	-13.59(-31.99-7.52)
Cyprus	Male	4.5(2.27-8.26)	15.6(9.57-23.36)	116.37(54.08-213.17)	174.53(112.97-265.75)	518.84(364.31-733.04)	75.87(44.24-116.3)	3.34(1.9-5.76)	4.75(2.61-7.75)	-10.6(-29.48-16.63)	87.42(53.54-135.69)	128.87(83.85-188.67)	-2.46(-20.49-23.36)
Czechia	Both	85.72(44.92-158.85)	130.73(78.33-211.81)	51.42(24.07-88.92)	4626.88(3290.71-6654.68)	6316.99(4671.54-8807.81)	31.49(16.53-48.96)	40.35(23.78-64.19)	28.03(15.66-46.92)	-21.47(-37.98-0.36)	1515.64(930.52-2263.52)	1029.51(664.05-1503.97)	-21.43(-33.84--6.28)
Czechia	Female	33.56(15.9-69.48)	53.59(27.65-100)	59.12(33.96-95.12)	2572.95(1668.92-3976.65)	3617.9(2424.53-5324.35)	37.49(19.85-57.25)	16.9(9.96-27.52)	13.5(7.5-23.09)	-11.37(-31.6-14.72)	580.87(360.45-865.1)	452.06(291.1-673.31)	-13.19(-27.17-3.54)
Czechia	Male	52.16(26.22-99.51)	77.14(44.42-128.54)	46.24(15.78-90.86)	2053.94(1379.06-3078.29)	2699.1(1950.6-3773.85)	24.51(7.99-45.3)	23.44(13.67-36.5)	14.53(7.95-23.94)	-28.69(-44.66--7.71)	934.77(567.27-1430.37)	577.45(361.44-864.6)	-26.27(-39.33--9.77)
Ivory Coast	Both	256.85(79.95-640.63)	572.59(190.3-1384.55)	9.36(1.67-21.77)	6126.51(3523.52-10511.68)	15198.83(8813.41-25847.28)	17.64(9.46-27.51)	107.38(66.05-164.42)	251.68(145.35-386.4)	83.47(37.03-146.28)	4633.36(2900.19-6957.83)	10952.28(6440.42-16674.85)	107.22(51.06-182.16)
Ivory Coast	Female	94.4(26.77-244.83)	207.44(62.52-520.15)	9.32(-0.18-23.12)	2821.62(1600.69-4856.96)	7066.8(4026.91-11726.35)	19.45(8.26-32.73)	43.39(25.94-69.24)	101.98(57.47-160.33)	88.77(28.07-164.73)	1932.05(1171.31-3034.86)	4536.43(2597.08-7138.14)	109.34(40.28-196.03)
Ivory Coast	Male	162.45(47.47-420.49)	365.15(114.95-884.51)	8.75(-1.19-23.07)	3304.89(1708.92-6083.28)	8132.03(4405.54-14713.86)	16.04(5.56-27.55)	64(39.11-98.13)	149.7(85.25-233.71)	79.63(30.8-155.6)	2701.31(1673.22-4064.8)	6415.86(3708.2-9928.06)	105.36(45.26-196.42)
Democratic People's Republic of Korea	Both	248.13(80.12-594.7)	249.91(116.43-521.84)	-18.92(-32.62-21.68)	8160.93(5519.95-12451.54)	13303.62(9605.65-18267.58)	30.7(12.96-49.35)	308.74(195.28-469.39)	432.23(263.12-642.81)	-0.23(-23.42-30.22)	14075.85(9212.39-21062.46)	18388.83(11427.71-27182.95)	39.85(3.38-90.51)
Democratic People's Republic of Korea	Female	93.97(28.11-246.68)	93.01(36.95-225)	-16.99(-30.87-22.81)	4384.97(2895.9-6625.53)	7195.92(4971.12-10437.37)	37.9(19.4-58.64)	170.59(104.67-271.02)	214.75(128.6-326.57)	-10.38(-32.19-22.39)	7542(4731.7-11702.53)	8794.24(5386-13003.27)	27.05(-9.08-78.73)
Democratic People's Republic of Korea	Male	154.16(51.05-373.94)	156.91(70.62-320.09)	-21.57(-35.7-17.27)	3775.96(2405.64-6029.69)	6107.7(4331.7-8682.82)	23.21(4.25-44.79)	138.15(84.53-215.48)	217.48(126.19-332.86)	12.31(-18.87-59.26)	6533.85(4039.3-10112.53)	9594.59(5872.78-14557.16)	54.55(8.53-124.75)
Democratic Republic of the Congo	Both	459.46(141.95-1104.46)	1062.67(355.48-2533.98)	4.4(-3.64-16.76)	12497.49(7379.47-20100.54)	34580.1(20444.01-55664.9)	22.61(13.13-32.58)	278.08(174.1-421.57)	525.72(320.88-790.06)	76.87(39.73-118.16)	11176.1(7169.79-16428.52)	21701.14(13847.67-32042.45)	103.03(55.12-159.48)
Democratic Republic of the Congo	Female	174.62(49.11-449.11)	411.2(126.29-1035.69)	8.93(-1.36-25.5)	6711.09(3976.18-10913.78)	19179.44(11457.32-31025.08)	28.24(16.05-41.98)	131.8(80.49-200.51)	245.57(140.98-385.7)	73.19(26.88-129.43)	5483.47(3455.93-8099.47)	10102.92(6135.98-15095.17)	96.25(38.13-169.21)
Democratic Republic of the Congo	Male	284.85(80.85-695.94)	651.46(193.67-1551.11)	0.68(-8.88-13.91)	5786.4(3025.72-10333.39)	15400.66(7999.92-27329.91)	16.44(6.49-27.48)	146.28(91.64-228.09)	280.15(166.93-436.19)	80.15(37.91-131.92)	5692.63(3654.78-8519.1)	11598.22(7237.73-17402.47)	109.54(55.14-174.51)

Denmark	Both	89.54(61.07-128.81)	150.11(105.79-207.74)	48.14(25.71-76.49)	3906.47(2943.65-5108.84)	5774.48(4529.28-7394.41)	31(16.07-49.28)	14.8(8.89-23.82)	32.36(18.54-54.5)	133.94(96.45-178.74)	569.3(386.68-799.34)	959.34(636.88-1379.09)	81.25(59.2-108.89)
Denmark	Female	31.35(17.96-50.57)	48.87(28.53-79.65)	39.19(17.58-64.65)	1853.56(1290.46-2577.49)	2712.81(1919.42-3774.25)	31.26(14.36-52.44)	7.26(4.41-11.68)	14.11(8.2-23.67)	107.27(69.5-155.37)	258.75(174.56-361.04)	391.82(265.19-567.83)	59.96(40.58-85.19)
Denmark	Male	58.2(36.81-90.26)	101.23(65.54-147.61)	51.99(24.39-89.37)	2052.91(1450.51-2892.5)	3061.68(2243.73-4161.4)	30.5(12.39-53.87)	7.54(4.44-12.02)	18.24(10.42-30.96)	159.81(114.46-212.52)	310.55(210.79-440.08)	567.52(374.45-832.27)	100.05(69.89-133.47)
Djibouti	Both	6.32(2.1-15.79)	17.13(7.2-39.85)	13.19(0.04-44.06)	191.58(116.96-310.64)	685.28(455.88-1048.87)	46.33(29.02-68.9)	2.01(1.17-3.29)	9.4(5.27-15.88)	154(88.52-228.96)	86.57(51.87-136.12)	384.84(223.29-637.65)	203.33(123.89-306.02)
Djibouti	Female	1.79(0.55-4.79)	5.22(1.94-12.42)	21.73(2.75-62.16)	84.89(48.36-138.61)	346.76(207.85-542.44)	63.56(41.78-90.79)	0.83(0.47-1.46)	3.85(2.05-6.86)	158.04(81.97-255.2)	35.24(20.78-59.93)	159.43(86.57-277.93)	203.91(109.44-332.78)
Djibouti	Male	4.53(1.47-11.43)	11.91(4.66-27.08)	9.73(-2.25-38.59)	106.69(61.14-183.09)	338.52(209.66-551.95)	32.3(16.84-52.86)	1.19(0.67-1.92)	5.56(3.14-9.54)	151.02(81.58-235.21)	51.33(30.43-78.86)	225.41(132.52-375.68)	203.42(120.58-309.98)
Dominica	Both	1.17(0.42-2.84)	1.19(0.53-2.69)	7.76(-6.48-39.51)	27.65(16.16-49.36)	34.08(21.96-56.68)	31.53(18.68-51.66)	1.32(0.88-1.93)	2.25(1.36-3.45)	60.22(38.57-89.28)	46.97(32.81-65.77)	80.73(51.28-122.18)	68.4(41.5-98.9)
Dominica	Female	0.45(0.13-1.16)	0.45(0.16-1.11)	9.66(-4.5-40.91)	12.35(7.19-21.4)	16.03(9.93-26.64)	42.24(26.22-64.93)	0.63(0.42-0.92)	0.99(0.61-1.49)	66.06(39.73-99.95)	22.22(15.31-31.05)	35.89(23.3-52.55)	73.21(42.32-111.39)
Dominica	Male	0.72(0.27-1.8)	0.74(0.32-1.67)	5.55(-9.47-39.09)	15.3(8.53-29.14)	18.05(11.15-30.99)	22.59(9.1-42.77)	0.69(0.45-1.02)	1.26(0.75-1.99)	53.72(25.31-92.78)	24.75(16.8-35.22)	44.84(27.63-68.63)	63.98(30.28-105.2)
Dominican Republic	Both	92.92(33.3-210.18)	194.24(86.01-400.92)	33.76(16.12-73.51)	2714.87(1684.8-4362.98)	5662.94(3740.01-8662.72)	38.71(25.71-56.86)	37.94(25.25-53.65)	141.43(82.22-239.3)	104.58(59.11-156.83)	1696.9(1168.35-2363.05)	5714.1(3524.91-9191.58)	177.68(114.03-256.39)
Dominican Republic	Female	33.76(9.56-84.72)	69.18(24.71-160.72)	34.2(16.99-73.46)	1290.19(758.5-2140.07)	2670.14(1653.34-4373.26)	40.74(25.68-61.34)	17.15(11.46-24.45)	62.84(36.03-102.83)	112.03(57.22-169.46)	784.85(538.54-1092.91)	2573.29(1560.7-4012.65)	188.34(110.26-277.02)
Dominican Republic	Male	59.16(21.88-135.37)	125.06(57.4-255.95)	32.07(13.72-72.56)	1424.68(831.73-2425.39)	2992.8(1888.11-4889.11)	36.5(21.59-57.33)	20.79(13.58-30.42)	78.58(41.91-137.91)	99.5(39.06-179.31)	912.05(617.11-1290.5)	3140.81(1781.9-5249.67)	169.69(90.51-280.27)
Ecuador	Both	134.49(49.4-298.46)	342.89(157.43-695.23)	45.54(25.52-90.11)	3850.07(2283.41-6319.57)	9595.46(6030.21-15517.26)	43.82(30.8-60.7)	64.3(42.74-89.89)	319.39(184.96-509.01)	175.65(130.55-225.33)	2632.37(1822.49-3542.75)	10942.28(6708.9-16820.06)	207.46(141.52-277.83)
Ecuador	Female	51.89(16.55-121.52)	131.59(53.4-290.74)	43.07(20.72-94.21)	1931.29(1090.24-3185.69)	4897.69(2907.28-7849.62)	45.84(29.57-68.56)	32.53(21.79-45.88)	157.41(90.84-249.63)	165.22(114.95-223.52)	1346.39(939.08-1818.32)	5368.74(3262.43-8317.78)	192.91(122.55-276.21)
Ecuador	Male	82.6(29.88-184.44)	211.3(92.83-426.24)	47.83(26.83-90.99)	1918.79(1068.85-3342.51)	4697.77(2752.96-8015.5)	41.78(25.75-61.69)	31.77(20.76-44.94)	161.98(93.25-259.07)	185.71(137.93-237.83)	1285.98(889.26-1766.18)	5573.55(3379.79-8668.72)	222.51(154.94-293.88)
Egypt	Both	1039.64(504.47-2174.87)	2764(1472.73-5306.74)	59.08(40.04-90.99)	30052.54(19661.3-45972.83)	80651.29(52102.88-125228.1)	53.32(36.46-71.69)	372.03(217.75-581.05)	891.41(443.35-1601.74)	97.89(50.21-144.24)	15045.85(9441.79-22323.47)	35444.39(19254.74-59096.42)	155.42(100.28-215.1)
Egypt	Female	439.64(166.99-1037.79)	1100.96(455.09-2383.21)	50.98(32.45-84.37)	14871.41(9010.84-23863.9)	42403.72(25610.53-67430.54)	63.98(45.6-87.42)	192.63(92.13-305.04)	417.67(158.31-765.85)	102.25(38.98-156.09)	7902.45(4111.86-12165.31)	16749.73(7533.02-28378.61)	146.86(82.05-215.28)
Egypt	Male	600(300.77-1226.26)	1663.04(891.68-3118.76)	64.65(42-98.28)	15181.13(9463.81-23900.33)	38247.58(23914.12-61912.93)	43.01(23.07-65.19)	179.4(103.29-291.42)	473.75(231.47-894.6)	97.72(36.87-158.75)	7143.4(4393.77-11048.47)	18694.67(10252.36-33355.75)	166.46(92.42-249.63)
El Salvador	Both	79.77(26.21-183.98)	133.41(47.65-285)	53.63(36.29-80.36)	1899.77(1099.13-3230.23)	3070.75(1832.61-5154.94)	38.58(25.59-53.91)	46.38(30.61-64.72)	270.59(163.09-430.69)	374(308.8-444.92)	1887.35(1299.52-2565.83)	9980.91(6214.5-15644.75)	538.44(423.08-665.23)
El Salvador	Female	25.56(7.88-63.62)	38.94(13.49-96.19)	33.87(15-62.43)	916.71(535.21-1542.68)	1458.46(869.68-2410.52)	30.17(16.33-48.17)	18.65(12.19-26.16)	88.56(51.88-142.48)	255.3(201.36-315.84)	753.57(528.09-1032.25)	3153.65(1915.09-4990.87)	381.76(280.04-501.92)
El Salvador	Male	54.21(17.59-126.54)	94.47(33.43-207.96)	67.92(45.84-96.46)	983.05(506.11-1804.72)	1612.29(852.72-2910.54)	48.22(30.52-67.59)	27.73(17.9-39.29)	182.04(110.21-286.71)	466.97(376.99-572.13)	1133.79(761.67-1572.01)	6827.27(4239.12-10612.72)	654.35(508.67-821.07)
Equatorial Guinea	Both	5.09(1.55-12.98)	18.79(6.01-45.93)	26.91(14.11-44.99)	129.05(77.09-219.06)	631.93(361.85-1103.57)	51.34(35.94-65.73)	3.69(2.13-5.94)	8.93(4.92-15.05)	128.36(56.84-232.55)	144.57(87.09-223.28)	377.15(209-642.93)	159.03(76.47-285.63)
Equatorial Guinea	Female	1.79(0.51-4.93)	6.39(1.97-16.32)	38.1(21.61-64.62)	70.92(42.69-117.87)	314.9(183.25-524.81)	53.93(37.68-72.29)	1.57(0.76-2.67)	4.36(2.06-8.06)	138.95(49.47-276.39)	64.16(31.54-104.89)	179.45(85.64-326.83)	165.18(61.88-332.94)
Equatorial Guinea	Male	3.31(0.96-8.51)	12.4(3.71-31.18)	15.12(1.21-31.68)	58.13(30.74-110.67)	317.02(157.63-608.75)	50.71(34.98-65.71)	2.12(1.18-3.53)	4.57(2.51-7.64)	121.25(34.76-268.6)	80.41(46.36-129.1)	197.7(111.92-327.92)	154.77(55.04-333.95)
Eritrea	Both	34.73(11.1-82.75)	86.71(30.6-204.61)	24.82(13.2-43.32)	1024.02(645.15-1624.1)	3159.79(2034.41-4858.82)	40.09(28.36-53.16)	18.57(10.54-31.69)	48.77(26.76-81.53)	209.92(138.39-313.61)	775.07(462.46-1283.76)	2041.68(1166.34-3386.28)	259.55(177.29-377.66)
Eritrea	Female	9.9(2.98-26.78)	25.84(8.51-65.06)	33.5(19.15-61.53)	500.15(307.15-797.25)	1617.7(988.18-2594.82)	47.01(31.64-65.44)	7.95(3.84-15.96)	21.65(11.63-40.25)	202.44(111.05-367.47)	332.06(172.29-651.86)	872.37(491.91-1596.57)	244.74(142.52-424.73)
Eritrea	Male	24.83(7.71-60.65)	60.87(20.67-145.61)	19.84(7.8-37.82)	523.87(296.25-909.67)	1542.08(905.58-2601.73)	33.48(20.85-46.31)	10.62(5.76-18.06)	27.11(13.04-47.55)	216.16(126.47-390.19)	443.01(254.73-731.46)	1169.31(610.82-2014.49)	271.67(173.58-455.73)
Estonia	Both	22.47(11.7-41)	33.77(20.58-52.6)	79.31(40.22-138.08)	1376.47(968.75-1906.19)	1760.27(1325.75-2306.3)	52.25(29.27-81.65)	4.24(2.66-6.21)	21.77(12.61-34.56)	518.1(394.94-680.24)	142.31(96.78-196.04)	475.59(303.64-693.36)	380.08(293.18-495.09)
Estonia	Female	9.59(4.5-18.93)	12.34(6.58-21.62)	50.42(21.7-95.43)	756.71(514.36-1091.76)	922.14(639.5-1314.7)	46(21.89-76.99)	2.28(1.38-3.49)	13.59(7.4-22.61)	572.39(416.77-771.05)	67.32(45.4-93.11)	250.67(154.83-379.51)	397.11(291-540.05)
Estonia	Male	12.88(6.46-24.73)	21.43(12.19-35.26)	103.42(54.47-181.07)	619.76(401.35-949.93)	838.13(576.52-1176.16)	59.83(33.29-98.01)	1.96(1.26-2.87)	8.19(4.81-12.95)	441.15(345.11-559.27)	74.99(51.58-104.81)	224.92(145.23-323.99)	360.69(285.19-457.37)
Eswatini	Both	13.91(4.79-33.95)	24.02(9.14-56.75)	23.71(12.08-43.15)	349.73(203.35-601.61)	678.6(403.04-1133.14)	39.74(28.43-53.14)	6.82(3.98-10.64)	21.6(11.67-35.45)	71.94(26.93-128.6)	274.49(168.03-416.58)	879.13(477.39-1443.47)	108.4(46.01-193.57)
Eswatini	Female	4.73(1.45-12.43)	7.48(2.56-18.65)	16.33(3.24-37.84)	167.27(90.72-299.72)	329.81(188.76-573.51)	44.81(30-63.33)	2.52(1.4-4)	6.14(2.96-10.9)	33.6(-12.35-114.18)	104.91(60.63-164.24)	243.92(123.28-429.45)	53.71(-6.1-149.49)
Eswatini	Male	9.18(3.18-23.12)	16.55(6.2-40.5)	25.52(12.66-44.39)	182.46(100.04-327.5)	348.79(193.39-617.71)	34.73(22.15-48.69)	4.29(2.41-6.82)	15.45(8.42-25.55)	93.45(35.27-166.82)	169.57(99.54-265.36)	635.21(346.88-1041.9)	140.58(61.65-242.58)
Ethiopia	Both	641.21(384.05-1078.5)	1413.43(888.79-2340.2)	20.5(8.72-33.15)	17591(13712.15-22979.97)	62247.08(49043.1-79917.21)	71.5(56.44-89.63)	576.13(372.05-837.68)	551.04(374.98-769.38)	54.96(20.48-101.99)	23592.97(15563.19-33537.13)	23074.09(16425.55-31290.38)	64.57(23.36-120.89)
Ethiopia	Female	230.04(128.09-404)	494.21(289.51-834.88)	18.61(3.21-34.34)	8468.92(6448.75-11196.54)	33085.5(25226.92-43244.51)	89.39(68.58-114.12)	279.09(175.99-420.66)	245.88(162.8-362.33)	44.73(2.54-97.4)	12063.52(7836.8-17768.8)	10523.65(7290.34-15120.04)	45.6(1.88-105.44)
Ethiopia	Male	411.17(248.15-689.45)	919.22(574.95-1527.2)	20.99(9.22-33.88)	9122.08(6863.51-12185.25)	29161.58(22040.29-38115.27)	54.9(42.24-69.99)	297.04(168.85-469.27)	305.16(200.27-434.27)	64.41(18.61-140.22)	11529.45(6772.21-17876.02)	12550.45(8561.17-17126.13)	84.22(28.97-178.84)
Fiji	Both	10.18(2.73-25.85)	12.33(3.69-30.41)	-4.06(-11.82-8.22)	262.92(142.97-454.53)	340.33(197.86-576.14)	7.65(0.18-17.66)	17.7(11.92-25.34)	34.78(22.39-51.59)	21.31(-1.08-46.25)	804.07(556.16-1113.54)	1441.63(972.44-2067.69)	33.73(8.93-63.77)
Fiji	Female	3.99(0.93-10.66)	4.75(1.18-12.33)	-4.82(-13.07-8.5)	118.88(64.74-203.25)	154.85(88.99-257.79)	8.02(-0.91-19.94)	7.87(4.97-11.92)	15.15(9.63-22.75)	10.21(-14.12-43)	365.02(239.07-529.8)	633.53(417.08-924.68)	25.39(-4.22-66.76)
Fiji	Male	6.18(1.75-15.73)	7.58(2.31-18.85)	-3.91(-14.05-11.63)	144.04(74.76-267.71)	185.48(102.86-334.23)	7.38(-1.48-18.52)	9.83(6.64-14.6)	19.63(12.69-29.11)	30.68(1.32-65.6)	439.05(308.35-629.16)	808.09(545.54-1165.66)	41.01(8.79-78.83)
Finland	Both	69.1(45.15-98.29)	102.48(72.97-143.62)	37.58(9.67-73.33)	4291.01(3002.89-6067.88)	4586.31(3621.41-5731.51)	-4.21(-22.74-20.02)	9.26(5.88-14.53)	16.87(9.7-27.35)	60.12(29.88-104.14)	403.82(284.89-555.87)	525.89(362.06-739.53)	30.27(13.08-52.79)

Finland	Female	30.25(16.28-49.47)	41.23(23.84-66.83)	28.83(2.82-63.47)	2388.41(1563.58-3533.65)	2357.43(1705.36-3268.06)	-9.75(-27.9-13.26)	4.71(2.81-7.52)	8.91(4.92-15.18)	67.61(32.57-117.55)	182.97(127.85-253.75)	243.72(165.07-345.58)	30.12(11.42-54.82)
Finland	Male	38.85(23.23-61.1)	61.25(38.23-92.09)	43.29(11.42-87.38)	1902.59(1260.81-2860.21)	2228.88(1626.13-3005.35)	2.72(-18.13-30.21)	4.56(2.91-6.9)	7.96(4.66-12.9)	52.52(19.3-102.85)	220.86(152.2-303.72)	282.17(191.82-402.1)	30.65(10.68-54.44)
France	Both	675.9(428.16-1069.59)	1774.84(1245.45-2508.76)	130.28(76.26-202.92)	35876.84(25194.82-51556.65)	72893.22(54696.57-94842.84)	77.73(45.87-118.87)	128.98(73.35-213.69)	159.89(87.68-269.46)	5.64(-9.75-24.13)	4260.25(2912.14-6186.07)	6093.23(4196.42-8531.59)	37.13(20.68-57.75)
France	Female	275.08(145.81-488.93)	694.8(385.73-1154.61)	120.74(70.17-186.58)	19144.43(12187.87-28692.36)	38474.28(26361.03-54612.38)	74.8(41.05-117.27)	62.91(33.78-109.46)	74.85(40.17-130.18)	-3.19(-20.86-18.39)	1913.17(1295.72-2829.69)	2775.45(1815.67-3954.05)	30.26(12.66-52.06)
France	Male	400.82(231.4-639.61)	1080.04(696.31-1626.65)	137.18(74.5-223.88)	16732.41(11216.51-25518.73)	34418.95(25147.25-47601.87)	81.07(44.96-127.79)	66.07(38.59-105.67)	85.04(47.59-142.64)	14.69(-3.38-37.45)	2347.07(1571.43-3402.58)	3317.78(2227.8-4810.83)	44.17(22.76-71.04)
Gabon	Both	12.8(4.06-32.35)	25.02(9.15-58.97)	16.34(4.61-40.85)	331.33(204.58-549.4)	813.69(505.56-1300.48)	40.11(27.74-55.59)	11.19(6.66-17.54)	22.71(11.87-38.06)	80.54(34.91-136.65)	421.7(256.81-641.63)	875.64(476.42-1418.72)	105.72(49.37-177.88)
Gabon	Female	4.34(1.25-12.05)	9.41(3.13-24.29)	28.47(12.07-60.73)	172.66(104.53-279.04)	467.63(287.22-740.17)	50.49(35.64-68.44)	4.31(1.78-7.2)	10.12(3.15-17.94)	103.99(25.1-183.62)	162.21(70.82-262.45)	393.75(133.31-687.28)	129.25(41.49-231.69)
Gabon	Male	8.47(2.49-21.97)	15.61(5.34-38.03)	10.4(-1.62-32.06)	158.66(86.38-295.63)	346.07(198.28-613.53)	27.95(15.23-42.53)	6.89(3.96-11)	12.59(7.37-20.88)	65.84(19.73-147.17)	259.49(153.9-394.62)	481.89(284.71-777.79)	91.36(33.22-187.92)
Gambia	Both	17.84(5.6-43.42)	44.73(15.31-107.71)	15.33(6.21-27.81)	487.06(291.17-807.6)	1330.47(799.17-2213.01)	22.78(13.06-32.61)	6.49(3.87-10.33)	18.7(11.56-28.74)	107.56(48.44-197.34)	271.35(168.56-418.69)	775.4(483.25-1171.84)	159(76.36-284.99)
Gambia	Female	6.87(2.03-18.06)	16.38(5.3-41.06)	8.87(-1.94-26.17)	235.89(136.03-391.98)	671.86(390.01-1090.5)	25.3(13.54-39.04)	2.77(1.58-4.54)	8.43(5.02-12.93)	106.77(36.78-201.34)	118.86(70.1-191.9)	351.84(211.55-535.55)	152.87(65.72-281.04)
Gambia	Male	10.97(3.44-26.89)	28.35(9.02-70.88)	19.8(8.69-32.75)	251.17(142.95-440.93)	658.62(370.04-1178.61)	20.49(7.88-32.67)	3.72(2.21-5.95)	10.27(6.13-16.54)	108.46(42.68-215.05)	152.49(94.3-237.1)	423.55(256.71-668.21)	165.03(75.27-314.44)
Georgia	Both	75.72(34.69-155.71)	66.04(33.39-126.81)	24.48(10.08-47.9)	3338.66(2315.98-4792.33)	2693.92(1884.83-3799.96)	20.98(8.32-35.44)	12.01(7.31-18.65)	17.68(10.7-27.52)	72.46(45.41-106.03)	592.47(383.03-873.8)	737.04(470.22-1097.68)	86.94(60.42-119.54)
Georgia	Female	30.56(11.61-70.53)	25.49(10.75-56.59)	20.06(4.2-45.15)	1694.5(1073.74-2626.97)	1418.02(909.9-2183.24)	26.45(11.09-44.5)	4.53(2.82-7)	6.49(3.94-10.35)	70.72(39.5-109.56)	222.41(143.85-331.26)	259.09(165.04-392.92)	78.59(47.15-118.07)
Georgia	Male	45.16(19.95-88.61)	40.55(19.55-74.17)	26.97(11.1-50.85)	1644.16(1057.56-2494.61)	1275.9(815.77-1895.86)	15.33(0.55-32.18)	7.48(4.39-11.69)	11.19(6.67-17.23)	72.37(43.75-109.26)	370.06(231.77-551.56)	477.95(300.68-722.99)	90.88(60.73-127.8)
Germany	Both	693.06(411.32-1111.43)	1382.84(947.13-1957.87)	83.87(41.76-145.59)	28504.2(19973.08-39124.71)	47562.66(36901.18-60380.56)	56.41(29.18-90.51)	241.6(142.39-389.71)	478.54(260.15-833.24)	86.23(36.01-157.4)	7863.61(5042.27-11616.08)	10981.84(7119.42-16796.42)	42.46(17.55-83.02)
Germany	Female	259.42(136.45-446.27)	511.05(289.06-828.69)	86.2(44.42-143.88)	14278.77(9482.62-20842.32)	23591.54(16513.25-33248.54)	59.7(30.46-98.34)	128.81(71.57-221.47)	252.46(133.45-453.37)	94.31(39.49-171.73)	3639.35(2322.36-5638.26)	5052.65(3208.42-7690.56)	43.94(17.24-84.84)
Germany	Male	433.64(244.03-745.93)	871.79(555.36-1306.52)	80.06(35.55-148.78)	14225.43(9571.47-20973.62)	23971.12(17463.46-32847.28)	52.8(24.82-91.08)	112.79(66.63-179.31)	226.08(124.54-391.74)	77.83(29.29-153.28)	4224.26(2670.47-6239.41)	5929.19(3843.43-8888.82)	40.86(14.96-84.28)
Ghana	Both	226.01(78.98-538.83)	556.48(214.48-1290.81)	26.36(16.5-42.98)	6733.6(4185.15-11424.92)	17946.6(11154.47-30033.76)	29.09(18.25-40.06)	126.47(76.63-200.06)	372.72(220.73-557.52)	113.02(44.12-181.38)	5348.48(3260.45-8155.22)	15718.99(9656.5-23280.57)	153.57(67.32-247.85)
Ghana	Female	102.06(30.8-273.67)	243.76(83.11-617.45)	20.72(7.54-46.35)	3362.5(1982.64-5902.25)	9877.12(6004.33-16737.97)	38.66(25.4-55.67)	79.97(45.29-134.73)	204.36(121.98-310.87)	87.4(16.29-166.04)	3452.77(1976.19-5663.86)	8739.41(5237.93-13289.61)	118.94(31.73-222.17)
Ghana	Male	123.95(41.51-307.51)	312.72(107.47-776.46)	31.63(19.66-45.66)	3371.1(1927.64-5859.09)	8069.48(4610.44-14210.93)	19.11(5.83-32.36)	46.51(27.93-73.39)	168.36(90.01-263.25)	158.41(81.83-247.55)	1895.71(1188.42-2880.31)	6979.58(3895.03-10610.41)	216.93(118.6-330.77)
Greece	Both	132.13(85.72-201.48)	233.09(164.56-319.24)	78.68(43.08-124.15)	6597.71(4418.13-9809.75)	10073.16(7275.31-13928.76)	53.03(24.91-88.85)	52.66(29.55-86.37)	57.88(31.59-100.63)	-21.83(-34.17--4.79)	1517.58(991.05-2239.68)	1618.38(1067.84-2363.75)	-3.9(-13.69-8.02)
Greece	Female	48.75(25.77-82.86)	91.84(51.01-143.85)	88.56(50.98-142.16)	3322.76(2056.64-5377.77)	5369.63(3441.59-8463.24)	60.15(28.75-101.96)	27.52(15.27-46.52)	28.07(15.03-50.7)	-30.77(-44.76--10.47)	714.67(459.65-1077.54)	710.81(461.44-1046.52)	-12.33(-24-3.09)
Greece	Male	83.38(49.82-135.23)	141.25(89.88-213.31)	73.85(36.01-121.28)	3274.95(2082.26-4930.6)	4703.53(3193.62-6545.75)	45.73(18.08-79.73)	25.13(14.13-40.29)	29.8(16.61-51.01)	-11.87(-24.12-3.58)	802.9(510.76-1174.09)	907.57(585.54-1342.16)	4.01(-7.27-17.32)
Greenland	Both	1.16(0.72-1.78)	1.32(0.9-1.85)	17.43(0.42-42.13)	95.85(67.25-138.66)	74.99(54.71-103.46)	-23.46(-34.69--10.29)	0.09(0.05-0.13)	0.12(0.07-0.21)	29.31(2.04-59.83)	5.37(3.6-7.62)	5.77(3.73-8.62)	24.5(5.45-49.98)
Greenland	Female	0.51(0.28-0.86)	0.53(0.31-0.84)	3.68(-12.15-24.47)	56.54(35.72-86.81)	44.47(29.7-66.41)	-25.24(-37.27--9.48)	0.03(0.02-0.05)	0.05(0.03-0.08)	35.14(2.82-69.68)	2.17(1.44-3.09)	2.29(1.47-3.32)	23.51(2.92-48.96)
Greenland	Male	0.65(0.37-1.07)	0.79(0.51-1.17)	29.28(6.84-64.45)	39.31(25.79-58.59)	30.52(21.29-43.65)	-22.05(-33.67--8.13)	0.05(0.03-0.08)	0.07(0.04-0.13)	25.22(-6.12-67.52)	3.2(2.07-4.64)	3.48(2.2-5.37)	25.08(2.6-57.18)
Grenada	Both	1.14(0.38-2.74)	1.8(0.83-3.82)	28.83(7.96-84.88)	27.29(16.11-48.8)	48.91(32.58-77.66)	48.03(28.99-76.1)	1.41(0.94-2)	3.31(2.12-4.85)	135.99(99.85-175.77)	52.36(36.46-72.3)	120.89(79.58-169.91)	109.09(72.48-149)
Grenada	Female	0.42(0.12-1.06)	0.62(0.22-1.43)	23.54(4.95-69.22)	11.85(6.84-20.57)	20.86(13.34-32.82)	51.16(30.52-81.17)	0.6(0.4-0.86)	1.37(0.89-2.02)	143.02(104.95-185.8)	22.41(15.77-31.9)	50.42(33.45-71.66)	115.46(75.55-158.55)
Grenada	Male	0.71(0.24-1.69)	1.19(0.54-2.47)	29.2(7.03-87.16)	15.44(8.44-28.44)	28.05(17.53-46.38)	44.27(23.22-76.55)	0.81(0.53-1.16)	1.94(1.22-2.88)	128.6(90.64-170.4)	29.95(20.58-41.12)	70.47(45.65-99.33)	102.79(66.63-145.79)
Guam	Both	1.55(0.48-3.87)	2.5(0.88-5.96)	17.03(7.51-38.66)	46.03(28.1-75.13)	73.25(46.87-119.47)	26.07(15.65-37.21)	1.7(1.18-2.32)	5.29(3.5-7.68)	54.62(34.29-78.29)	74.61(53.51-98.33)	214.14(148.21-299.11)	84.74(56.94-119.12)
Guam	Female	0.63(0.16-1.71)	1(0.29-2.55)	14.03(2.44-38.58)	21.06(12.53-34.1)	34.83(21.74-55.1)	26.52(15.07-40.91)	0.87(0.58-1.21)	2.49(1.65-3.68)	30.37(8.86-55.64)	36.94(25.93-49.93)	98.36(67.09-139.5)	63.73(32.58-102.68)
Guam	Male	0.92(0.29-2.36)	1.5(0.54-3.6)	19.38(7.12-41.9)	24.97(15.14-42.68)	38.42(23.36-66.9)	25.94(13.37-39.28)	0.83(0.57-1.17)	2.8(1.85-4.04)	77.97(45.84-118.11)	37.67(26.54-50.95)	115.78(80.3-164.11)	104.9(64.99-153.83)
Guatemala	Both	148.54(46.35-391.83)	363.67(119.38-951.86)	31.65(20.64-46.8)	2866.9(1590.85-5329.23)	8150.22(4449.77-15404.07)	30.58(20.47-41.21)	79.45(52.25-111.43)	422.06(259.44-645.36)	307.42(261.3-358.13)	3291.63(2258.8-4467.04)	16281.88(10410.8-24290.58)	367.31(297.75-452.74)
Guatemala	Female	49.38(13.22-130.07)	116.29(33.85-296.21)	23.8(11.12-43.45)	1315.14(723.49-2284.34)	3675.99(2002.22-6333.29)	24.58(13.11-37.9)	40.9(26.53-56.91)	215.66(130.76-332.08)	302.05(239.93-369.07)	1725.65(1173.14-2378.27)	8184.68(5229.3-12463.66)	350.29(256.54-454.33)
Guatemala	Male	99.16(30.99-254.99)	247.38(77.15-650.64)	37.26(22.99-55.06)	1551.76(773.66-3197.2)	4474.23(2189.96-9454.09)	36.75(22.63-49.76)	38.54(24.96-56.27)	206.41(128.5-315.79)	313(261.92-375.8)	1565.98(1044.89-2178.6)	8097.2(5269.44-11864.35)	387.17(314.71-481.46)
Guinea	Both	120.64(42.66-290.73)	268.29(92.3-659.05)	15.74(6.7-23.45)	3140.98(1959.69-5002.86)	7002.13(4148.1-11799.45)	10.5(-0.74-20.92)	65.72(40.48-102.6)	118.81(71.94-177.65)	71.94(40.66-112.34)	2581.97(1651.64-4013.25)	4959.9(3066.25-7394.12)	100.63(61.75-149.42)
Guinea	Female	49.39(15.85-124.21)	102.8(31.18-258.01)	7.8(-2.08-17.21)	1594.98(941.85-2586.81)	3673.89(2073.46-6125.45)	12.85(0.16-26.11)	38.14(23.01-65.66)	58.74(34.49-92.29)	53.88(19.45-100.61)	1536.97(935.46-2562.86)	2529.41(1522.86-3943.33)	75.67(33.25-130.1)
Guinea	Male	71.25(24.21-172.73)	165.49(52.44-406.43)	21.55(9.62-31.72)	1546(883.79-2634.9)	3328.24(1781.58-5929.19)	8.05(-5.73-21.09)	27.58(15.86-45.46)	60.06(35.39-92.62)	98.05(54.39-162.08)	1045(630.74-1694.33)	2430.49(1510.98-3727.91)	138.07(84.15-211.31)
Guinea-Bissau	Both	20.28(6.2-48.31)	40.13(12.93-95.4)	13(5.36-22.34)	454.46(271.32-783.48)	1030.84(614.65-1791.31)	22.43(13.39-32.38)	15.9(9.78-24.83)	25.85(15.93-38.61)	88.52(51.26-136.74)	657.5(415.9-1022.63)	1126.07(706.91-1662.25)	119.82(70.85-181.78)
Guinea-Bissau	Female	7.72(2.25-19.24)	14.88(4.52-37.09)	10.65(1.84-23.25)	223.07(132.54-375.86)	517.03(305.87-873.47)	24.97(13.81-38.48)	7.89(4.55-12.74)	12.55(7.29-19.44)	78.49(35.58-135.93)	343.08(195.66-554.09)	557.58(327.14-863.13)	103.05(49.94-174.12)

Guinea-Bissau	Male	12.57(3.81-29.91)	25.25(7.81-59.92)	14.15(4.39-24.82)	231.39(123.4-415.69)	513.81(277.7-903.8)	19.99(9.36-31.45)	8.01(4.69-12.73)	13.3(8.31-19.94)	97.63(50.26-169.65)	314.43(192.03-499.06)	568.49(364.31-838.63)	137.33(77.22-226.43)
Guyana	Both	10.1(3.1-26.01)	12.71(4.8-29.17)	26.05(11.8-60.29)	232.65(129.36-433.9)	315.61(190.95-549.42)	36.32(23.33-53.75)	9.09(6-12.93)	23.5(13.99-37.36)	150.74(107.65-200.1)	382(261.47-532.37)	920.36(572.8-1424.4)	175.82(122.09-239.68)
Guyana	Female	3.8(1.01-10.35)	4.78(1.51-12.3)	26.27(10.32-60.09)	104.24(57.78-191.63)	144.82(85.7-248.42)	39.32(24.28-59.09)	4.03(2.6-5.86)	11.19(6.61-17.74)	164.41(118.01-219.78)	169.8(113.86-240.24)	440.7(273.52-685.49)	195.42(130.9-274.82)
Guyana	Male	6.3(1.95-15.67)	7.93(3.09-18.07)	25.7(9.74-63.2)	128.41(67.03-247.98)	170.79(98.41-314.41)	33.93(20.05-54.32)	5.06(3.36-7.29)	12.31(7.37-19.6)	139.38(96.95-186.3)	212.2(142.68-295.79)	479.66(295.72-732.46)	160.14(108.2-217.89)
Haiti	Both	85.53(25.68-219.07)	190.58(65.95-471.76)	19.75(9.86-41.92)	1778.69(1013.55-3205.06)	4726.11(2827.71-8224.45)	36.7(26.42-49.42)	94.74(57.85-148.32)	190.48(106.52-332.42)	68.6(38-102.67)	3947.77(2532.92-6124.94)	7894.96(4690.79-13375.29)	86.53(47.56-133.04)
Haiti	Female	38.18(9.35-104.45)	84.79(22.97-226.53)	19.6(9.13-40.31)	856.5(458.9-1595.32)	2340.98(1303.23-4120)	40.01(27.75-56.89)	47.73(28.07-82.82)	101.7(54.25-192.16)	75.99(36.87-123.03)	2091.95(1258.61-3667.79)	4332.14(2429.3-8078.06)	89.05(41.76-149.51)
Haiti	Male	47.36(14.97-114.61)	105.79(37.37-242.63)	19.81(5.91-43.24)	922.19(519.33-1720.07)	2385.13(1380.78-4220.62)	33.68(22.04-47.1)	47(27.09-75.59)	88.78(47.55-155.29)	60.83(25.4-103.29)	1855.82(1146.35-2845.79)	3562.82(2051.55-6008.8)	82.95(40.07-141.24)
Honduras	Both	74.74(22.04-177.16)	158.93(52.29-358.14)	15.78(5.22-35.23)	1526.8(787.48-2760.33)	3837.2(2115.71-6690.77)	22.44(12.13-35.42)	42.27(27.46-63.57)	210.66(117.92-343.67)	161.1(100.88-222.12)	1763.28(1185.12-2535.14)	7362.92(4170.26-11824.16)	211.57(133.71-297.94)
Honduras	Female	28.56(7.47-74.09)	59.37(17.76-152.67)	10.65(-1.8-32.97)	745.39(399.05-1312.42)	1917.67(1072.07-3190.86)	21.92(8.66-38.4)	22.49(14.66-32.05)	123.1(68.26-197.7)	165.12(97.61-246.88)	972.18(647.56-1388.9)	4415.21(2514.94-7053.65)	215.18(125.41-321.37)
Honduras	Male	46.18(13.9-112.95)	99.57(32.78-233.89)	20.13(6.9-41.26)	781.4(364.7-1551.29)	1919.53(952.27-3671.9)	23.24(11.92-38.82)	19.78(11.63-33.98)	87.55(48.48-144.8)	148.35(86.26-230.45)	791.1(490.27-1269.31)	2947.71(1667.26-4793.57)	197.97(119.6-305.4)
Hungary	Both	80.08(42.87-147.28)	152.82(96.29-245.2)	110.74(61.48-183.87)	4525.14(3237.66-6288.47)	7238.92(5375.54-9851.72)	72.01(50.14-98.14)	45.67(29.14-67.99)	59.39(34.47-94.79)	44.39(15.94-80.01)	1714.22(1141.72-2400.92)	1828.81(1196.58-2715.16)	34.54(16.36-58.46)
Hungary	Female	30.71(13.94-63.9)	65(34.3-116.58)	129.86(73.98-211.71)	2443.29(1625.38-3627.35)	4232.13(2920.1-6127.69)	84.49(59.63-113.51)	22.8(14.45-34.37)	33.66(19.02-55.12)	48.19(15.29-91.77)	785.85(527.75-1112.23)	914.09(602.35-1330.45)	34.48(14.8-61.61)
Hungary	Male	49.37(24.96-93.85)	87.82(51.79-148.35)	100.27(53.43-175.79)	2081.86(1387.93-3106.4)	3006.79(2143.83-4214.89)	56.96(32.31-88.35)	22.87(14.45-34.1)	25.74(14.95-42.57)	37.84(12.73-69.98)	928.37(611.46-1316.61)	914.71(581.22-1359.16)	34.02(14.58-59.25)
Iceland	Both	2.69(1.67-4.18)	4.41(2.9-6.44)	23.61(3.85-48.89)	134.13(94.99-183.56)	215.07(157.67-283.34)	16.79(1.63-33.41)	0.23(0.13-0.38)	0.46(0.26-0.79)	62.13(37.67-89.36)	9.95(6.84-14.05)	17.71(12.07-24.98)	45.4(29.47-61.65)
Iceland	Female	0.99(0.53-1.75)	1.56(0.9-2.66)	19.86(0.35-48.55)	67.75(43.81-100.61)	109.69(74.56-161.48)	19.11(1.32-40.32)	0.1(0.06-0.17)	0.18(0.1-0.31)	53.16(23.91-88.07)	4.05(2.73-5.86)	6.99(4.66-10.06)	42.34(25.3-62.29)
Iceland	Male	1.7(0.99-2.78)	2.84(1.72-4.3)	25.42(3.55-52.56)	66.39(43.99-100.3)	105.38(73.2-149.12)	14.43(-1.42-32.79)	0.13(0.08-0.21)	0.28(0.16-0.49)	67.4(40.08-97.87)	5.91(4.01-8.24)	10.72(7.12-15.32)	47.12(29.14-66.67)
India	Both	11160.12(8962.35-14103.	16757.19(13555.94-20842.	3.53(-3.55-11.38)	398070.35(343759.68-467208.9	801779.61(697812.53-936835.6	24.85(17.08-33.86)	6304.45(3797.62-9616.	15788.66(9391.57-2474	120.51(89.83-163.84)	250672.92(160050.46-374384.	588074.47(364103.15-888584.7	169.1(126.68-226.19)
India	Female	3780.3(2873.26-4888.78)	5622.93(4439.62-7190.53)	2.37(-5.97-12.13)	178103(151879.4-212239.22)	392163.59(333685.43-467553.9	33.66(23.02-45.86)	2387.39(1418.14-3659.	6181.91(3555.37-9712.4)	128.8(84.79-207.83)	96236.19(60917.46-144014.13)	225937.75(135499.36-345738.5	172.01(114.18-269.03)
India	Male	7379.82(5977.23-9255.47)	11134.25(9017.66-13758.0	4.22(-3.08-12.08)	219967.35(188177.51-259952.7	409616.02(353372.44-482888.2	17.74(10.15-25.74)	3917.06(2290.15-6058.	9606.75(5641.72-14869.	115.02(81.52-158.37)	154436.73(95453.68-231168.3	362136.72(221066.02-549375.8)	165.94(119.51-225.24)
Indonesia	Both	2327.73(1672.35-3265.26)	3307.65(2479.73-4479.27)	6.11(-3.27-16.57)	80628.71(67193.88-96328.98)	144820.36(121669.24-170861.6	30.18(21.12-40.71)	3523.6(2558.2-4654.96	5784.2(4031.83-7943.29)	32.11(12.48-53.62)	168814.55(125814.21-217177.	253438.27(182585.24-339671.6	65.99(39.51-97.42)
Indonesia	Female	938.33(656.04-1374.47)	1260.29(926.57-1756.47)	1.07(-9.07-11.75)	40375.47(32755.58-48872.65)	74470.51(61843.62-89313.19)	34.61(24.11-47.9)	1684.26(1192.43-2285.	2386.5(1559.16-3411.24)	16.34(-6.74-41.57)	81022.24(56895.08-108031.87)	104742.14(72060.65-146355.05)	45.15(13.19-85.74)
Indonesia	Male	1389.39(999.21-1931.69)	2047.36(1532.53-2802)	9.23(-0.39-19.87)	40253.24(32773.03-48475.28)	70349.85(58263.87-83267.92)	25.79(17.36-35.39)	1839.34(1313.98-2414.	3397.7(2254.18-4878.09)	45.87(25.71-70.44)	87792.3(64531.39-112859.66)	148696.13(101092.9-212935.77)	84.28(55.16-122.75)
Iran (Islamic Republic of)	Both	1345.59(1041.39-1715.41)	2658.24(2141.34-3185.3)	36.53(16.75-60.38)	34025.89(28762.74-40829.65)	82804.22(70764.08-97931.58)	64.55(46.79-82.78)	227.81(149.09-327.16)	442.17(293.1-625.96)	77.07(56.86-101.89)	9925.76(7019.62-13560.7)	19010.91(13512.74-25669.41)	141.87(111.45-179.88)
Iran (Islamic Republic of)	Female	550.78(409.54-736.15)	1014.47(793.43-1270.43)	25.69(7.57-47.43)	16209.29(13662.89-19653.61)	42356.51(35509.94-50900.38)	75.67(55.63-96.53)	93.51(62.68-134.54)	180.96(121.34-255.16)	69.93(46.21-98.37)	4317.99(3082.28-5841.74)	7938.29(5733.38-10586.36)	118.84(87.83-160.61)
Iran (Islamic Republic of)	Male	794.82(619-1013.19)	1643.77(1339.15-2001.63)	44.76(23.38-69.44)	17816.6(14910.22-21574.25)	40447.71(34600.3-48094.27)	54.38(37.94-71.27)	134.3(84.12-201.91)	261.21(169.56-369.66)	82.39(58.84-114.6)	5607.77(3860.33-7912.43)	11072.61(7759.59-15087.46)	161.88(126.04-206.45)
Iraq	Both	360.35(120.96-906.7)	1132.52(520.98-2471.06)	35.3(12.1-100.4)	6979.59(3675.07-13275.73)	29647.26(17640.36-50830.28)	76.88(50.87-113.96)	159.18(99.87-246.5)	385.23(221.87-628.97)	59.54(27.69-97.9)	6514.84(4156.2-9725.65)	16110.57(9718.05-25584.16)	87.91(41.93-143.19)
Iraq	Female	160.61(44.63-438.86)	450.44(168.74-1145.26)	20.39(1.47-69.32)	3366.92(1705.81-6631.29)	14321.66(8097.99-25782.34)	77.37(47.36-117.7)	75.96(44.34-128.72)	162.08(88.72-268.73)	45.03(4.72-94.91)	3218.56(1926.06-5356.47)	7051.63(4061.76-11442.61)	61.84(10.32-132.56)
Iraq	Male	199.74(70.39-464.94)	682.08(313.09-1402.14)	47.45(19.22-121.23)	3612.67(1806.53-6953.89)	15325.6(8786.35-26723.25)	76.41(52.26-113.08)	83.21(49.41-130.29)	223.15(125.66-371.57)	73.05(35.44-119)	3296.27(2002.65-5001.77)	9058.94(5283.21-14551.19)	114(63.28-179.09)
Ireland	Both	89.54(59.64-129.93)	144.76(99.63-204.86)	20.86(0.72-46.02)	3353.2(2533.12-4569.48)	5613.41(4277.56-7381.14)	21.45(5.6-40.03)	10.79(6.15-18.26)	13.16(7.19-22.09)	16.02(-2.1-40.94)	381.46(261.19-536.62)	501.22(347.59-694.85)	15.22(0.1-35.21)
Ireland	Female	34.01(18.91-56.56)	49.24(28.4-82.46)	6.65(-14.53-37.64)	1647.85(1140.05-2334.46)	2819.86(1956.38-4032.03)	23.66(3.42-45)	5.17(2.84-8.94)	6.04(3.31-10.42)	6.74(-13.1-30.77)	173.05(115.67-249.04)	213.13(143.2-299.57)	2.3(-10.97-19.52)
Ireland	Male	55.53(34.4-84.68)	95.52(60.25-143.16)	30.63(5.72-58.68)	1705.34(1170.48-2471.18)	2793.55(1987.32-3838.55)	19.35(2-40.1)	5.61(3.17-9.42)	7.12(3.9-12.1)	24.99(4.17-55.73)	208.4(138.33-296.31)	288.09(190.26-408.95)	27.67(5.07-58.23)
Israel	Both	63.41(38.82-103.44)	147.25(94.74-225.82)	22.91(4.39-49.59)	2750.34(1958.84-3876.45)	5558.14(4064.84-7434.97)	7.68(-8.91-28.06)	31.96(18.84-52.06)	75.39(44.53-120.64)	43.41(25.2-65.14)	874.35(586.36-1276.14)	1960.63(1317.51-2863.81)	42.81(28.22-58.4)
Israel	Female	21.64(11.71-38.6)	47.89(26.83-81.94)	17.54(-1.09-44.27)	1342.53(873.96-1973.68)	2682.91(1761.59-3961.78)	7.16(-10.97-30.8)	15.76(9.35-25.92)	32.36(18.93-53.26)	18.39(-0.8-42.64)	411.88(275.39-608.04)	774.01(524.58-1145.79)	17.23(3.1-33.42)
Israel	Male	41.77(23.99-72.87)	99.37(59.06-161.4)	25.47(4.43-56.28)	1407.81(933.37-2081.36)	2875.23(2014.08-4115.13)	8.1(-9.27-29.43)	16.2(9.49-26.39)	43.03(25.99-69.02)	69.84(47.85-95.67)	462.47(309.12-686.86)	1186.62(786.21-1737.38)	66.93(48.73-87.31)
Italy	Both	799.69(678.04-943.05)	1179.01(992.54-1387.84)	45.53(34.09-59.2)	44242.46(38540.96-50459.75)	59850.92(51426.1-69079.36)	26.83(16.81-37.55)	184.18(105.75-296.61)	179.93(100.89-300.12)	-19.09(-32.89-1.34)	6479.11(4307.1-9361.99)	6116.01(4123.62-8549.71)	-5(-13.96-6.65)
Italy	Female	305.1(251.76-370.08)	486.05(391.24-596.75)	60.32(44.86-77.95)	24537.84(20753.41-28656.71)	35123.93(29574.07-41972.14)	34.86(22.09-49.19)	89.8(50.54-150.85)	87.77(49.09-149.73)	-25.42(-40.09--4)	2957.02(1976.01-4279.8)	2809.71(1905.98-3994.35)	-9.71(-19.78-3.18)
Italy	Male	494.58(407.16-594.66)	692.96(571.5-828.83)	35.39(24.23-48.22)	19704.62(16997.56-23064.4)	24726.99(21117.52-28717.87)	17.03(8.01-26.44)	94.38(53.88-150.21)	92.16(51.92-152.13)	-12.26(-26.92-9.23)	3522.09(2332.7-5067.27)	3306.3(2205.01-4660.44)	-0.06(-9.11-11.2)

Jamaica	Both	31.13(10.68-75.84)	41.33(19.07-88.66)	11.45(-4.87-57.3)	832.62(493.11-1495.73)	1298.44(871.12-2092.42)	30.44(13.99-55.86)	19.85(13.05-28.28)	60.04(36.52-91.68)	96.78(68.04-131.82)	725.39(502.61-987.07)	2313.25(1469.89-3460.33)	148.94(107.15-195.95)
Jamaica	Female	11.06(3.2-28.96)	14.47(5.26-34.05)	10.99(-4.8-55.95)	363.97(199.29-651.63)	584.28(363.65-955.38)	35.65(17.58-62.38)	8.7(5.88-12.13)	28.6(17.61-43.21)	119.79(85.31-161.89)	343.07(237.72-469.98)	1149.25(727.49-1702.63)	165.73(115.96-224.6)
Jamaica	Male	20.07(7.1-48.39)	26.87(12.33-58.32)	11.07(-7.51-59.68)	468.64(258.24-861.24)	714.16(452.95-1143.44)	26.11(8.76-53.57)	11.15(7.17-16.58)	31.43(18.44-48.83)	78.67(49.59-113.5)	382.32(260.69-528.79)	1164(725.42-1771.18)	133.97(96.17-178.89)
Japan	Both	2133(1703.98-2596.98)	2972.49(2346.13-3672.63)	42.64(27.65-59.01)	126625.25(104495.76-151545.6	141867.52(114985.55-170140.3)	9.5(0.61-19.52)	769.02(496.05-1155.67	757.05(476.55-1197.16)	-42.96(-52.11--32.52)	33038.97(21504.14-46875.3)	29775.45(19030.84-42950.29)	-24.09(-31.67--15.12)
Japan	Female	754.18(591.88-942.38)	982.73(750.53-1244.22)	33.54(19.11-50.68)	70074.63(56957.45-85500.53)	76856.68(61442.18-93898.99)	6.57(-3.47-19.26)	339.53(217.75-519.68)	282.41(175.96-462.81)	-55.1(-63.56--44.97)	14224.51(9399.87-19829.69)	11427.12(7476.31-16543.11)	-33.73(-40.31--25.72)
Japan	Male	1378.82(1096.78-1715.79)	1989.76(1567.86-2486.27)	47.51(30.8-66.31)	56550.62(46493.04-67579.16)	65010.84(53257.34-78006.62)	13.07(2.9-22.58)	429.49(279.1-639.05)	474.64(298.72-739.37)	-31.73(-43.07--18.53)	18814.46(12019.95-27150.6)	18348.33(11599.38-26664.27)	-16.26(-25.53--4.44)
Jordan	Both	62.84(19.79-165.48)	285.53(129.05-622.23)	47.79(20.13-131.04)	1489.87(794.94-2853.06)	8047.71(4941.72-13688.36)	73.54(48.18-115.09)	21.96(13.92-32.72)	83.11(51.77-123.64)	75.68(46.31-111.78)	910.95(595.73-1294.26)	3434.9(2275.62-4949.57)	81.36(46.82-123.33)
Jordan	Female	28.93(7.6-78.43)	122.6(46.07-291.09)	39.96(13.46-112.79)	751.07(386.41-1464.5)	4240.28(2431.6-7410.96)	85.99(53.61-133.86)	10.61(6.58-15.95)	32.57(20.16-51.38)	61.95(15.42-102.82)	445.51(285.66-639.6)	1366.73(880.02-2029.32)	52.91(8.26-100.45)
Jordan	Male	33.91(10.63-86.08)	162.93(71.88-346.51)	54.02(22.28-146.9)	738.8(362.43-1507.1)	3807.43(2182.38-6856.46)	61.73(38.87-102.69)	11.35(7.04-17.05)	50.54(30.49-76.3)	85.4(51.23-135.47)	465.43(297.59-681.7)	2068.17(1288.04-3032.01)	106.52(60.24-173.59)
Kazakhstan	Both	273.7(106.69-628.28)	414.14(194.57-852.42)	31.29(14.95-68.76)	8965.74(5833.18-14059.14)	14139(9554.21-21420.3)	41.73(27.55-60.31)	43.94(27.05-67.08)	59.6(35.38-90.88)	26.5(1.51-56.95)	2284.65(1446.02-3359.58)	2841.72(1786.21-4165.98)	30.93(7.98-58.95)
Kazakhstan	Female	105.09(32.29-276.43)	143.9(51.29-345.04)	17.74(4.21-50.2)	3940.34(2367.48-6360.14)	6282.92(3929.87-10099.26)	42.79(27.74-64.14)	19.68(11.9-30.44)	24.11(14.23-37.13)	17.06(-6.68-46.8)	996.72(623.57-1499.79)	1113.64(705.25-1659.96)	16.83(-5.36-43.1)
Kazakhstan	Male	168.61(64.05-380.41)	270.24(125.29-549.89)	40.39(20.94-82.39)	5025.4(3089.65-8206.95)	7856.07(5044.76-12117.1)	41.07(24.68-62.96)	24.26(14.65-37.24)	35.48(20.87-54.16)	33.6(5.75-69.73)	1287.93(817.31-1901.61)	1728.08(1090.95-2516.35)	42.02(16.09-74.47)
Kenya	Both	268.36(217.06-330.16)	631.36(518.01-768.42)	15.54(2.92-31.49)	10174.24(8890.89-11710.77)	30463.82(26391.09-35227.1)	39.55(29.83-50.23)	77.72(49.41-117.66)	272.34(171.02-410.71)	129.83(90.59-168.68)	3195.56(2157.28-4540.6)	11377.22(7587.31-16654.57)	178.67(130.64-231.53)
Kenya	Female	89.27(70.48-111.53)	211.56(172.13-262.5)	18.18(4.32-36.42)	5133.4(4447-5978.64)	17083.02(14388.26-20077.84)	55.65(42.91-70.27)	35.77(22.16-55.51)	129.84(77.25-204.88)	144.5(84.94-208.67)	1481.41(979.9-2224.08)	5480.38(3445.61-8507.76)	193.95(120.14-280.8)
Kenya	Male	179.1(145.2-220.85)	419.8(342-508.8)	13.35(1.1-29.84)	5040.84(4371.6-5836.82)	13380.81(11598.58-15435.97)	23.27(15.06-32.34)	41.95(25.45-66.48)	142.5(85.07-224.87)	117.59(79.27-161.33)	1714.15(1132.6-2526.01)	5896.83(3778.01-8781.98)	165.33(116.04-226.96)
Kiribati	Both	1.11(0.3-2.67)	1.95(0.54-4.74)	4.88(-3.11-14.06)	22.28(12.59-38.82)	42.15(24.07-72.81)	18.8(12.56-26.31)	2.82(1.96-3.99)	6.36(4.08-9.4)	82.64(39.64-124.61)	128.94(90.25-176.75)	288.71(189.58-412.63)	101.03(50.59-158.18)
Kiribati	Female	0.49(0.12-1.28)	0.85(0.22-2.24)	4.09(-5.87-16.58)	10.91(6.03-19.14)	21.27(12.1-36.84)	20.65(12.86-31.07)	1.65(1.1-2.39)	3.16(1.96-4.96)	53.77(17.35-98.51)	74.71(50.3-105.53)	139.58(89.3-217.6)	69(24.15-123.53)
Kiribati	Male	0.62(0.17-1.54)	1.1(0.3-2.64)	5.5(-4.47-17.33)	11.37(6.12-20.57)	20.88(10.98-38.23)	17.12(8.57-25.99)	1.18(0.8-1.71)	3.2(1.85-4.76)	122.87(28.93-205.28)	54.22(38.07-76.67)	149.12(86.32-218.06)	145.59(43.86-245.63)
Kuwait	Both	28.6(12.86-62.53)	109.14(62.23-197.54)	53.68(16.71-129.76)	847.59(521.43-1361.4)	3323.1(2355.82-4664.06)	52.61(26.83-91.57)	8.1(5.29-11.47)	13.93(8.51-21.02)	-8.34(-23.31-9.11)	368.26(251.29-504.02)	702.73(467.05-974.81)	2.13(-14.89-22.25)
Kuwait	Female	11.54(3.86-29)	41.35(19.03-87.16)	33.1(-0.08-120.12)	392.17(222.48-685.56)	1719.3(1107.02-2573.89)	56.31(24.26-102.83)	3.22(2.13-4.65)	5.74(3.4-8.95)	12.03(-11.31-46.12)	150.35(102.99-210.11)	305.28(200.61-445.64)	-3.35(-24.97-24.12)
Kuwait	Male	17.06(8.11-35.3)	67.79(38.8-118.29)	71.59(30.54-148.76)	455.42(278.54-726.7)	1603.8(1088.37-2337.17)	47.32(22.89-83.37)	4.87(3.11-7.05)	8.18(4.97-12.35)	-17.4(-30.29--3.61)	217.91(145.23-302.69)	397.45(259.03-566.26)	5.83(-11.64-26.41)
Kyrgyzstan	Both	107.02(36.85-254.32)	190.18(79.77-421.53)	17.46(2.22-50.13)	3127.3(2041.87-4937.81)	6690.97(4489.56-10264.95)	47.07(29.08-67.05)	20.23(12.39-31.4)	22.36(13.1-35.23)	10.38(-10.07-33.16)	1060.26(667.35-1627.29)	1232.66(789.64-1838.93)	27.86(4.94-56.23)
Kyrgyzstan	Female	43.69(12.15-114.03)	72.53(24.74-177.18)	9.8(-3.79-39.04)	1500.01(939.01-2454.58)	3267.81(2029.48-5276.26)	50.9(31.16-76.17)	8.98(5.5-13.95)	8.96(5.17-14.27)	5.1(-16.61-32.32)	474.03(297.64-723.19)	492.89(314.73-731.93)	14.56(-9.19-46.2)
Kyrgyzstan	Male	63.33(22.51-152.23)	117.65(52.28-252.65)	22.73(4.47-56.84)	1627.29(970.63-2692.81)	3423.15(2190.13-5454.56)	43.41(24.04-64.74)	11.26(6.76-17.45)	13.4(7.81-21.06)	13.72(-8.14-37.51)	586.23(363.9-907.97)	739.78(473.58-1097.95)	38.6(13.03-69.52)
Lao People's Democratic Republic	Both	62.75(17.34-158.54)	103.14(32.78-253.65)	4.67(-2.75-17.79)	1449.42(832.28-2610.38)	3111.14(1850.52-5323.57)	25.85(16.34-38.02)	159.6(103.72-234.98)	241.8(153.07-354.63)	100.87(59.56-154.95)	6970.78(4702.08-10022.47)	10406.83(6794.71-14771.81)	146.86(86.94-227.74)
Lao People's Democratic Republic	Female	27.18(6.53-74.8)	43.24(11.25-117.82)	4.22(-4.26-18.09)	721.04(411.04-1309.83)	1558.95(923.62-2700.63)	29.24(17.8-44.48)	87.02(54.05-127.57)	117.46(73.04-175.86)	86.68(45.02-151.1)	3859.17(2436.43-5699.18)	5086.55(3233.37-7304.15)	123.22(65.38-215.18)
Lao People's Democratic Republic	Male	35.57(10.79-88.11)	59.9(20.17-146.19)	4.27(-5.42-19.22)	728.38(392.42-1340.22)	1552.19(876.04-2704.75)	22.48(11.86-34.2)	72.58(46.56-110.96)	124.34(75.69-188.65)	119.4(56.78-193.29)	3111.61(2066.91-4580.84)	5320.29(3356.14-7825.52)	177.01(95.91-283.19)
Latvia	Both	27.76(13.26-55.83)	36.08(20.8-61.12)	86.08(46.87-156.43)	1617.49(1115.54-2344.51)	2061.67(1497.34-2864.63)	75.83(49.23-107.2)	4.1(2.49-6.16)	13.94(8.1-21.97)	313.58(261.67-376.72)	140.59(96.38-201.66)	376.7(245.03-548.94)	300.84(245.14-362.66)
Latvia	Female	11.63(4.68-26.09)	13.8(6.37-27.09)	67.42(33.42-128.68)	848.7(565.35-1248.73)	1101.08(746.72-1613.39)	77.49(48.46-111.15)	2.21(1.28-3.46)	8.57(4.63-14.19)	335.07(273.45-416.53)	66.44(44.53-95.86)	197.15(118.44-302.02)	309.81(241.36-387.82)
Latvia	Male	16.13(7.13-31.71)	22.28(12.57-37.81)	101.15(55.8-186.23)	768.8(483.88-1190.67)	960.58(658.45-1398.05)	74.09(45.5-110.18)	1.89(1.16-2.83)	5.37(3.24-8.36)	278.5(227.32-335.72)	74.15(50.12-104.54)	179.56(116.9-259.18)	289.1(234.27-347.45)
Lebanon	Both	54.11(22.64-118.92)	138.07(73.73-253.42)	58.21(27.53-121.43)	1354.45(843.7-2231.82)	3811.05(2566.54-5766.22)	77.26(55.82-106.72)	23.84(14.63-37.51)	31.61(18.8-51.56)	-11.81(-35.53-17.35)	909.02(575.38-1383.24)	1225.5(767.03-1864.26)	19.92(-10.84-55.87)
Lebanon	Female	23.86(8.22-59.44)	58.48(24.59-124.27)	47.31(17.47-113.82)	696.89(414.55-1186.52)	2059.18(1310.95-3205.71)	81.19(57.32-114.22)	11.52(7-18.12)	15.73(9.04-24)	-14.5(-42.82-13.08)	457.49(281.84-705.56)	621.84(384.9-929.13)	10.04(-24.37-49.45)
Lebanon	Male	30.25(12.12-64.92)	79.59(39.7-144.7)	68.46(36.11-132.92)	657.56(375.58-1121.89)	1751.87(1105.26-2805.47)	72.66(50.75-102.35)	12.32(7.3-20.35)	15.89(8.83-28.89)	-10.23(-42.38-39.13)	451.53(281.92-712.5)	603.65(354.9-1024.27)	29.15(-8.73-89.61)
Lesotho	Both	25.07(9.72-54.28)	35.85(15-75.78)	23.09(12.38-37.07)	804.94(524.6-1211.96)	1165.58(763.35-1723.51)	27.91(16.5-40.13)	11.62(6.52-19.2)	39.76(21.88-64.97)	87.08(28.69-160.31)	442.27(265.02-699.31)	1559.23(845.58-2518.81)	127.13(49.91-218.32)
Lesotho	Female	7.67(2.63-18.47)	11.06(4.03-25.63)	25.6(10.36-43.95)	378.63(228.28-597.2)	560.12(338.39-890.9)	31.05(16.54-47.89)	3.51(1.96-6.04)	14.31(7.13-24.02)	109.34(33.54-209.5)	135.76(80.76-223.66)	545.78(283.71-891.26)	149.87(51.4-276.71)
Lesotho	Male	17.39(6.99-38.78)	24.8(10.35-53.67)	21.13(9.51-35.6)	426.32(253.92-684.7)	605.46(367.9-963.8)	25.07(12.78-38.78)	8.11(4.32-13.74)	25.45(13.18-43.57)	80.23(10.46-164.16)	306.51(173.62-501.42)	1013.45(524.83-1718.44)	119.32(29.26-230.27)
Liberia	Both	31.75(10.55-75.7)	81.54(28.47-197.45)	9.83(1.89-21.42)	818.72(495.13-1386.54)	2462.29(1483.13-4259.9)	23.95(14.66-35.47)	25.23(15.51-37.74)	48.34(28.27-78.74)	147.85(75.49-245.62)	965.33(606.74-1428.42)	2074.43(1243.69-3323.3)	202.06(101.64-336.53)
Liberia	Female	13.51(4.05-34.68)	33.05(10.21-83)	6.52(-2.52-23.09)	416.18(241.74-768.39)	1273.13(728.57-2353.86)	28.74(15.84-44.07)	13.06(7.99-19.35)	25.36(14.38-39.78)	140.59(62.48-239.81)	521.78(325.64-768.46)	1097.54(639.88-1695.25)	182.53(84.07-316.77)
Liberia	Male	18.23(6.09-45.93)	48.49(16.95-123.11)	11.55(1.76-23.34)	402.55(223.65-707.93)	1189.16(664.66-2168.22)	19.2(8.67-32.56)	12.17(6.97-20.17)	22.98(12.17-42.11)	153.38(68.2-275.4)	443.54(263.47-714.49)	976.89(530.44-1806.25)	222.37(111.77-398.23)

Libya	Both	70.47(25.37-173.19)	161.98(89.98-290.66)	44.98(1.34-158.89)	1697.69(967.98-3150.37)	5214.68(3534.46-8222.16)	89.17(50.61-142.8)	21.14(12.98-32.31)	61.93(34.39-102.11)	82.28(42.78-128.73)	878.42(546.32-1309.64)	2626.69(1544.69-4157.91)	135.9(80.79-204.05)
Libya	Female	31.55(9.63-87.21)	65.32(29.69-132.77)	29.07(-8.93-134.83)	850.24(463.48-1613.06)	2736.64(1718.75-4565.99)	96.67(50.56-155.18)	9.95(5.81-15.64)	31.55(16.38-52.08)	96.42(41.82-150.45)	423.18(259.96-635.77)	1352.46(747.18-2180.71)	144.98(76.43-225.67)
Libya	Male	38.91(14.16-93.46)	96.65(51.3-172.92)	58.41(7.19-177.94)	847.45(455.99-1635.81)	2478.04(1575.28-4141.65)	81.45(45.81-135.7)	11.19(6.44-18.05)	30.38(16.13-55.57)	69.52(23.17-138.93)	455.24(272.22-724.11)	1274.23(724.46-2194.89)	126.37(64.47-214.14)
Lithuania	Both	44.05(21.21-93.72)	56.19(33.93-94.51)	67.7(28.28-135.69)	2542.24(1690.79-3811.48)	3542.68(2485.83-5011.06)	81.79(51.65-125.2)	6.81(4.28-9.99)	14.7(8.64-22.55)	117.51(89.33-149.07)	235.96(161.25-330.32)	436.16(291.73-622.5)	135.33(107.29-165.63)
Lithuania	Female	18.3(7.61-41.38)	22.21(11.23-44.31)	53.11(16.67-116.31)	1360.04(832.86-2161.95)	2005.53(1295.33-2996.57)	88.21(53.43-135.5)	3.46(2.1-5.34)	8.28(4.77-13.19)	115.39(81.67-150.82)	108.76(73.01-152.05)	214.97(141.71-313.77)	132.04(99.66-168.48)
Lithuania	Male	25.76(12.1-53.59)	33.98(19.65-58.09)	82.24(39.17-160.72)	1182.2(729.18-1844.27)	1537.15(1042.05-2250.76)	74.05(42.33-120.59)	3.35(2.14-5.01)	6.42(3.86-9.83)	116.55(88.34-148.49)	127.2(86.75-180.22)	221.19(147.26-316.95)	137.62(107.24-169.13)
Luxembourg	Both	6.3(4.32-9.24)	9.39(6.3-13.75)	-7.5(-19.18-5.12)	283.35(209.21-385.35)	456.3(334.72-613.17)	-0.83(-13.18-13.34)	1.38(0.8-2.3)	2.26(1.25-3.85)	51.39(29.54-76.89)	45.1(30.14-63.94)	68.4(46.07-96.83)	27.78(14.55-43.55)
Luxembourg	Female	2.28(1.29-3.7)	3.43(1.91-5.52)	-5.24(-21.37-12.17)	135.91(91.85-197.7)	236.35(158.33-351)	10.01(-6.38-28.42)	0.73(0.41-1.26)	1.18(0.65-2)	43.54(20.77-73.71)	20.69(13.84-29.44)	32.38(21.47-47.24)	25.18(10.33-42.2)
Luxembourg	Male	4.01(2.5-6.26)	5.97(3.71-9.27)	-9.67(-22.63-4.97)	147.44(100.53-214.35)	219.95(151.22-316.8)	-10.72(-23.23-4.12)	0.64(0.38-1.03)	1.08(0.61-1.8)	59.94(32.76-96.38)	24.4(16.2-34.64)	36.01(23.87-51.83)	30.67(14.69-49.95)
Madagascar	Both	154.8(47.83-372.06)	339.74(118.79-803.3)	3.56(-4.81-19.63)	4613.69(2834.12-7391.27)	13366.25(8433.37-20651.84)	32.6(20.39-47.19)	67.7(42.2-104.23)	130.93(76.73-210.21)	76.38(42.88-116.49)	2850.08(1874.26-4226.53)	5592.49(3438.83-8619.8)	94.87(56.32-143.05)
Madagascar	Female	50.11(14.4-134.67)	114.01(35.57-302.83)	8.21(-3.18-30.98)	2215.05(1311.88-3655.99)	7037.07(4225.68-11337)	44.79(28.32-64.61)	32.05(19.34-53.53)	65.06(35.46-110.02)	78.78(39.16-130.03)	1403.26(876.27-2312.82)	2791.01(1662.75-4553.27)	93.26(44.42-155.29)
Madagascar	Male	104.69(31.86-247.88)	225.73(76.39-521.41)	0.94(-7.77-16.14)	2398.64(1316.97-4084.43)	6329.17(3522.31-10573.56)	21.3(10.42-33.23)	35.66(21.59-55.4)	65.87(37.69-108.14)	73.71(34.06-123.94)	1446.82(932.51-2128.68)	2801.48(1728.25-4399.95)	96.13(51.86-151.75)
Malawi	Both	109.19(34.64-270.9)	221.55(73.12-557.14)	15.94(5.62-27.81)	4761.78(2777.59-8327.15)	11209.45(6520.65-19400.19)	24.52(14.24-36.92)	57.41(36.25-86.69)	100.79(63.43-149.42)	140.46(90.25-201.21)	2313.81(1526.7-3334.43)	4117.89(2654.74-5996.86)	180.11(115.11-267.66)
Malawi	Female	36.83(10.86-99.63)	67.55(21.7-177.64)	5.57(-5.92-25.26)	2479.23(1325.33-4370.18)	6253.53(3398-10975.39)	31.49(18.11-48.85)	28.58(17.04-43.63)	42(25.61-63.58)	114.83(53.96-216.88)	1176.1(695.8-1759.6)	1690.8(1078.99-2490.23)	135.99(66.14-271.22)
Malawi	Male	72.36(21.75-180.31)	154(45.02-387.42)	20.67(8.36-34.53)	2282.55(1239.02-4019.8)	4955.92(2673.4-8851.93)	16.65(3.85-29.58)	28.83(17.49-43.86)	58.79(35.96-88.84)	164.05(104.74-240.42)	1137.7(733.62-1666.54)	2427.09(1554.66-3571.82)	223.32(147.23-324.65)
Malaysia	Both	167.64(52.75-447.91)	335.71(130.74-815.27)	13.34(-0.48-50.89)	5677.75(3494.38-9269.06)	13067.27(8619.21-20275.2)	32.53(21.28-48.01)	282.34(200.13-378.82)	603(378.59-904.26)	-0.21(-14.93-16.04)	12104.23(9015.7-15649.53)	23287.42(15780.95-33334.72)	10.16(-8.88-30.84)
Malaysia	Female	70.6(19.13-198.17)	141.6(45.7-354.71)	17.37(2.08-54.99)	2804.52(1656.28-4663.01)	6559.34(4178.18-10402.82)	37.77(24.39-55.63)	121.09(84.41-164.2)	275.24(170.69-422.34)	9.82(-10.59-32.67)	5114.02(3699.26-6638.84)	10196.7(6627.82-14840.67)	18.34(-6.32-47.01)
Malaysia	Male	97.04(29.84-251.12)	194.11(73.9-463.05)	9.72(-6.22-49.44)	2873.23(1693.89-4954.96)	6507.93(4141.67-10479)	27.56(15.36-45.05)	161.25(113.59-217.65)	327.76(206.89-487.52)	-7.3(-24.26-12.95)	6990.21(5112.56-9093.32)	13090.72(8808.96-18961.14)	4.24(-15.73-26.99)
Maldives	Both	3.15(0.97-7.78)	5.64(2.37-12.5)	-13.65(-27.04-26.64)	83.65(48.13-145.08)	235.61(156.95-351.57)	27.01(6.04-58.19)	5.93(3.96-8.36)	8.67(6.05-11.85)	40.24(9.27-78.07)	250.52(174.11-339.98)	368.97(264.79-490.83)	60.9(22.35-110.52)
Maldives	Female	1.31(0.35-3.57)	2.07(0.7-5.23)	-6.64(-20.12-30.96)	40.17(22.06-68.76)	97.22(59.74-151.15)	33.47(11.8-65.84)	3.27(1.96-5.11)	3.52(2.38-5.05)	16.85(-24.57-79.95)	140.34(85.37-216.88)	138.66(96.92-187.7)	27.3(-19.28-100.89)
Maldives	Male	1.83(0.57-4.45)	3.57(1.46-7.92)	-20.13(-34.13-19.66)	43.48(24.28-77.87)	138.39(89.62-220.06)	22.05(-0.58-55.8)	2.66(1.75-3.79)	5.15(3.58-7.18)	69.85(33.6-116.87)	110.17(77.68-150.21)	230.31(163.82-312.91)	100.6(54.1-171.4)
Mali	Both	246.22(78.23-631.3)	706.72(216.22-1815.78)	23.2(12.69-32.95)	3970.2(2428.08-6546.06)	11371.73(6546.93-19709.69)	15.26(3.35-25.11)	95.58(57.96-146.26)	171.38(101.46-274.81)	43.14(15.89-77.15)	3875.69(2427.94-5948.32)	7248.42(4436.11-11435.92)	51.29(20.48-89.03)
Mali	Female	110.93(30.11-296.21)	310.55(81.11-842.7)	23.02(10.07-36.09)	1907.14(1148.56-3299.72)	5553.59(3141.7-10097.06)	17.92(4.51-30.7)	54.46(30.36-88.6)	86.65(47.75-143.45)	33.49(0.21-73.44)	2271.28(1284.47-3660.33)	3708.23(2091.05-6023.26)	36.67(0.83-77.82)
Mali	Male	135.29(42.6-342.11)	396.16(119.31-1028.14)	22.67(8.5-37.39)	2063.05(1164.38-3583.23)	5818.15(3100.64-10458.49)	12.76(-1.09-24.72)	41.12(25.28-65.59)	84.73(49.15-137.77)	57.19(21.34-103.93)	1604.41(995.49-2403.84)	3540.19(2088.17-5618.85)	73(33.44-124.52)
Malta	Both	4.23(2.75-6.47)	9.84(6.93-13.56)	101.93(53.73-156.95)	198.52(145.87-268.48)	365.72(280.24-466.61)	52.2(33.82-72.87)	1.5(0.85-2.5)	2.34(1.28-3.96)	11.44(-3.33-29.9)	44.44(28.98-64.9)	68.52(44.27-100.69)	24.07(10.8-38.14)
Malta	Female	1.62(0.92-2.76)	3.55(2.11-5.76)	91.5(51.43-144.03)	100.15(67.44-142.82)	175.45(122-244.51)	47.48(28.51-71.61)	0.74(0.42-1.29)	1.15(0.62-1.98)	8.83(-7.96-28.8)	20.82(13.84-30.52)	31.03(19.86-45.81)	20.36(5.64-36.69)
Malta	Male	2.61(1.56-4.27)	6.29(4.17-9.46)	107.57(51.61-174.09)	98.37(67.91-138.02)	190.27(137.43-256.16)	56.64(33.21-83.19)	0.76(0.42-1.25)	1.18(0.65-1.98)	13.91(-2.96-35.28)	23.62(15.04-34.56)	37.49(24.04-55.15)	27.28(12.01-45.27)
Marshall Islands	Both	0.66(0.17-1.76)	0.77(0.21-1.99)	-7.94(-15.4-3.69)	13.7(6.96-27.24)	18.24(10.25-33.97)	6.76(-1.26-19.75)	0.95(0.66-1.36)	2.73(1.66-4.22)	99.1(60.03-138.69)	43.89(30.73-61.23)	121.2(75.33-182.94)	114.77(66.32-159.76)
Marshall Islands	Female	0.28(0.07-0.77)	0.33(0.08-0.91)	-5.69(-14.41-6.11)	6.37(3.28-12.44)	8.58(4.81-16.15)	7.92(-1-21.43)	0.44(0.28-0.69)	1.35(0.77-2.3)	99.18(52.83-149.49)	20.1(13.26-31.13)	59.26(34.44-100.09)	115.06(59.66-176.61)
Marshall Islands	Male	0.38(0.1-1.07)	0.44(0.13-1.19)	-9.8(-19.89-4.09)	7.33(3.48-15.5)	9.66(5.17-19.1)	5.76(-3.9-21.37)	0.51(0.35-0.73)	1.38(0.87-2.04)	96.31(52.02-146.76)	23.79(16.49-33.6)	61.94(39.4-89.54)	112.69(56.8-172.99)
Mauritania	Both	43.99(16.7-105.81)	94.88(40.23-214.5)	6.96(-5.01-25.66)	1267.28(807.88-2014.19)	3231.9(2070.56-5127.21)	32.46(22.89-44.26)	28.72(18.12-43.63)	34.69(19.25-53.75)	30.92(-2.49-65.76)	1121.1(731.95-1638.12)	1418.94(825.72-2203.77)	50.1(11.69-96.04)
Mauritania	Female	18.57(6.6-47.59)	39.07(15.05-95.15)	4.96(-6.26-22.92)	617.49(365.84-1018.69)	1611.95(957.51-2632.9)	34.14(22.2-48.24)	16.78(10.05-25.69)	20.53(11.3-32.65)	33.55(-2.52-74.44)	657.99(395.43-1001.24)	835.41(467.22-1335.61)	46.43(5.82-95.14)
Mauritania	Male	25.42(9.21-63.35)	55.82(23.57-127.67)	8.2(-5.7-29.62)	649.79(381.21-1084.99)	1619.96(972.6-2703.94)	30.95(18.52-44.81)	11.94(7.3-18.65)	14.16(7.29-23.94)	27.67(-13.93-79.59)	463.11(296.53-701.13)	583.53(308.87-958.13)	53.45(5.34-116.73)
Mauritius	Both	12.44(3.86-32.57)	16.41(6.6-39.65)	15.25(-2.44-58.91)	425.23(268.88-736.96)	605.75(415.6-969.22)	22.43(10.02-39.4)	40.32(28.06-54.87)	130.32(81.36-194.77)	106.74(78.49-136.86)	1663.11(1207.45-2172.83)	4743.56(3090.08-7018.11)	120.24(82.96-158.29)
Mauritius	Female	5.31(1.39-14.58)	6.5(2.18-16.43)	6.84(-7.81-44.93)	211.36(125.55-365.96)	294.63(189.31-473.2)	18.53(5.68-36.94)	17.95(12.42-24.36)	54.41(33.73-83.43)	78.04(49.72-109.27)	738.91(533.51-955.74)	1943.51(1252.25-2897.51)	93.95(55.44-137.69)
Mauritius	Male	7.14(2.19-18.52)	9.91(3.93-22.82)	21.58(-2.37-69.31)	213.87(130.86-379.09)	311.12(205.24-507.02)	26.42(11.11-46.51)	22.37(15.42-30.67)	75.91(47.17-114.18)	132.01(98.99-166.7)	924.2(665.86-1215.19)	2800.05(1790.79-4131.87)	142.79(101.43-186.86)
Mexico	Both	1428.77(1054.62-1925.34)	2661.52(2090.59-3542.87)	41.16(27.29-58.52)	31524.43(25939.27-38924.78)	64545.44(54823.29-78064.94)	41.37(30.82-53.88)	743.41(520.65-1012.72	4370.24(2717.73-6462.9	250.1(206.02-288.56)	31391.77(22764.09-41269.96)	158543.6(101648.42-226772.18)	307.5(240.62-374.97)
Mexico	Female	576.17(417.52-795.49)	976.41(756.98-1294.06)	26.04(11.82-42.28)	15704.85(13039.38-19304.53)	31483.03(26723.95-37797.17)	37.01(25.9-50.59)	389.46(271.48-522.51)	2034.57(1220.67-3190.7	205.04(159.26-246.39)	16458.08(12010.53-21338.73)	72338.4(45245.28-109287.51)	247.93(180.28-320.88)
Mexico	Male	852.6(624.29-1161.85)	1685.11(1286.61-2276.85)	52.76(37.69-71.72)	15819.58(12730.44-19863.1)	33062.41(27184.2-40491.72)	45.9(34.21-58.39)	353.94(244.08-490.78)	2335.68(1442.11-3461.4	298.35(250.62-346.99)	14933.69(10734.41-19849.06)	86205.2(55074.2-124560.57)	372.23(293.93-452.65)

Micronesia (Federated States of)	Both	1.77(0.49-4.68)	1.73(0.51-4.56)	-2.55(-11.1-10.15)	37.31(19.88-70.1)	41.71(23.41-75.19)	15.34(7.99-25.31)	3.37(2.26-4.87)	7.05(3.82-10.78)	86.74(16.46-155.9)	152.46(103.94-217.11)	302.17(156.7-452.51)	113.62(20.05-202.9)
Micronesia (Federated States of)	Female	0.76(0.18-2.14)	0.74(0.19-2.02)	-3.11(-13.3-11)	17.52(9.43-32.19)	19.68(11-35.03)	14.24(5.96-25.83)	1.66(1.06-2.44)	3.22(1.65-5.2)	67(-4.48-150.81)	74.11(48.01-108.61)	135.19(64.9-217.7)	94.31(-0.32-199.83)
Micronesia (Federated States of)	Male	1.01(0.29-2.67)	1(0.29-2.53)	-2.2(-12.82-13.7)	19.79(9.98-38.18)	22.02(11.95-41.61)	16.48(7.57-27.79)	1.7(1.14-2.5)	3.83(1.94-6.36)	105.58(18.96-190.88)	78.35(53.04-113.3)	166.98(84.21-273.22)	131.8(26.69-232.79)
Monaco	Both	0.59(0.4-0.83)	0.97(0.68-1.37)	33.3(18.29-52.01)	25.25(19.33-32.42)	35.25(27.4-44.66)	13.45(1.36-26.3)	0.07(0.04-0.13)	0.13(0.07-0.23)	47.28(23.64-72.98)	2.77(1.89-4.01)	4.23(2.79-6.31)	29.23(14.87-44.16)
Monaco	Female	0.24(0.13-0.4)	0.38(0.22-0.64)	32.38(13.98-54.15)	13.08(9.33-18.42)	18.16(12.83-24.85)	15.45(1.14-31.67)	0.03(0.02-0.06)	0.06(0.03-0.11)	59.07(27-98.11)	1.18(0.78-1.77)	1.87(1.23-2.81)	34.39(14.88-56.28)
Monaco	Male	0.35(0.23-0.54)	0.59(0.38-0.88)	32.39(13.57-55.67)	12.17(8.82-16.53)	17.1(12.49-22.52)	11.21(-2.22-27.43)	0.04(0.02-0.07)	0.07(0.04-0.12)	37.57(9.93-71.15)	1.59(1.04-2.29)	2.36(1.51-3.49)	25.32(8.4-44.66)
Mongolia	Both	61.19(24.4-138.91)	127.69(65.65-254.15)	34.39(8.29-92)	1844.8(1201.08-2932.5)	4958.57(3428.92-7128.87)	72.82(46.56-103.05)	13.42(8.12-20.5)	18.67(10.26-31.6)	15.39(-5.02-37.97)	654.14(412.36-994.08)	1006.49(605.04-1600.77)	57.22(27.14-92.23)
Mongolia	Female	25.12(8.09-63.29)	50.13(20.91-109.31)	26.77(2.31-86.18)	879.89(531.51-1444.23)	2562.03(1625.34-3836.73)	84(53.64-120.88)	7.37(4.32-11.72)	7.41(3.99-12.99)	-5.06(-24.3-20.88)	358.5(212.85-567.62)	403.27(243.62-654.29)	27.52(-2.01-66.16)
Mongolia	Male	36.07(14.14-81.85)	77.56(38.33-151.84)	40.47(13.57-99.49)	964.9(591.06-1572.75)	2396.54(1559.26-3637.85)	62.59(38.72-92.74)	6.05(3.53-9.33)	11.26(6.3-18.87)	40.45(7.75-78.41)	295.64(183.17-451.53)	603.22(358.44-969.81)	92.91(48.01-143.64)
Montenegro	Both	8.34(4.2-16.41)	12.51(7.67-20.63)	59.36(25.92-116.25)	317.93(220.97-472.08)	468.18(339.6-655.8)	46.98(27.9-69.84)	3.13(1.96-4.76)	4.47(2.59-7.31)	-9.57(-27.15-9.92)	120.85(78.67-177.83)	153.63(97.14-236.14)	3.89(-14.36-25.31)
Montenegro	Female	2.87(1.24-5.99)	4.55(2.27-8.42)	65.35(32.06-120.04)	157.32(96.93-251.91)	244.46(160.62-370.92)	53.97(35.96-76.76)	1.22(0.75-1.91)	1.91(1.12-3.16)	-3.81(-25.59-19.33)	45.12(28.55-67.2)	63.06(40.11-97.02)	11.2(-11.5-35.32)
Montenegro	Male	5.47(2.56-11.17)	7.96(4.59-13.27)	57.79(22.82-122.33)	160.61(102.54-252.08)	223.72(153.82-324.77)	40.1(18.83-68.42)	1.91(1.19-2.94)	2.57(1.44-4.26)	-12.99(-32.93-13.17)	75.72(49.36-113.14)	90.57(56.17-140.95)	-0.06(-20.65-26.03)
Morocco	Both	334.51(128.88-758.38)	904.6(473.52-1708.46)	93.72(54.96-182.76)	9947.63(6004.17-15955.39)	26394.91(17019.75-39922.48)	86.68(64.8-116.82)	136.05(83.13-207.15)	326.13(180.74-535.36)	86.2(43.55-131.45)	5465.1(3475.73-7960.06)	12633.19(7405.02-20087.65)	155.86(92.21-222.72)
Morocco	Female	149.76(51.26-359.61)	356.46(157.04-764.96)	71.77(41.27-143.64)	5258.31(3058.25-8639.39)	13385.19(8163.3-20950.53)	80.29(57.52-110.27)	64.17(37.7-98.63)	168.01(93.16-285.09)	106.85(42.08-177.72)	2693.95(1636.91-4066.75)	6717.05(3870.24-10736.96)	170.79(84-272.48)
Morocco	Male	184.74(71.83-410.08)	548.14(282.97-1024.07)	110.88(63.38-213.76)	4689.32(2723.97-8266.64)	13009.72(8161.5-21491.86)	93.85(67.86-132.56)	71.88(42.13-113.37)	158.13(82.06-270.38)	68.56(28.3-118.7)	2771.16(1701.65-4167.09)	5916.14(3372.55-9624.41)	140.77(84-213.09)
Mozambique	Both	149.77(51.05-375.41)	429.25(134.56-1094.68)	37.34(24.27-47.89)	4854.47(3053.8-7936.36)	12753.31(7412.44-22154.07)	18.64(4.54-31.56)	69.39(41.27-107.74)	160.62(95.97-246.03)	84.06(31.47-145.97)	2755.28(1711.75-4170.04)	6792.72(4251.93-10015.51)	122.99(57.46-197.08)
Mozambique	Female	44.89(14.68-124.87)	125.46(39.58-346.28)	36.49(23.14-50.88)	2539.35(1507.95-4105.81)	6869.87(4011.18-11430.17)	22.38(6.83-38.43)	31.94(19.24-49.05)	65.18(37.22-104.02)	68.46(20.82-132.04)	1285.91(793.78-1938.87)	2694.36(1609.12-4147.71)	93.95(36.11-164.8)
Mozambique	Male	104.87(32.47-274.7)	303.79(83.81-818.59)	35.94(18.8-49.72)	2315.13(1290.25-4114.8)	5883.44(3012.68-11580.82)	14.55(-4.03-31.77)	37.45(20.78-63.03)	95.44(57.52-145.75)	96.43(25.48-186.68)	1469.37(889.73-2328.98)	4098.36(2585.62-6153.63)	147.23(58.57-258.65)
Myanmar	Both	651.68(190.71-1676.01)	858.19(283.6-2084.12)	7.2(-1.54-26.28)	15270.47(8746.76-27371.69)	25932.25(15950-43132.36)	29.16(18.98-43.98)	1293.95(858.93-1847.3	1624.25(1070.89-2284.9	53.95(20.78-97.66)	62022.99(41529.73-87591.76)	72971.23(48629.11-102141.24)	93.9(46.67-154.86)
Myanmar	Female	317.71(77.51-915.31)	384.59(108.61-1071.15)	-3.42(-12.8-14.77)	7622.44(4168.07-14057.15)	13528.47(8015.21-22789.28)	30.57(15.6-50.65)	752.17(493.45-1088.49	811.84(537.32-1152.96)	27.93(-7.64-74.28)	36947.16(23894.6-54343.99)	36326.32(23926.93-51781.03)	57.51(7.6-124.87)
Myanmar	Male	333.97(92.81-875.26)	473.6(148.64-1179.69)	17.8(6.24-40.34)	7648.03(4121.81-13378.46)	12403.79(7097.07-21335.72)	27.79(17.63-41.67)	541.78(351.06-803.49)	812.41(526.94-1158.76)	89.09(41.7-149.58)	25075.83(16383.18-36521.5)	36644.91(24502.6-51054.56)	146.66(80.6-233.34)
Namibia	Both	18.19(7.11-42.03)	39.49(17.28-85.72)	35.42(20.05-63.21)	572.52(349.9-892.37)	1369.56(872.35-2065.13)	42.66(29.8-57.56)	8.83(4.75-15.57)	16.94(8.94-30.24)	23.32(-7.01-70.81)	327.59(179.8-554.71)	661.06(364.79-1139.39)	45.46(8.52-108.68)
Namibia	Female	5.58(1.72-14.12)	12.28(4.38-29.37)	36.79(17.91-71.23)	259.83(152.6-417.19)	655.33(391.93-1025.68)	48.21(32.09-68.83)	2.99(1.54-5.77)	5.45(2.56-11.71)	13.43(-19.09-63.78)	114.1(60.74-210.42)	208.86(104.19-424.05)	31.11(-10.41-96.37)
Namibia	Male	12.61(5.2-28.35)	27.21(12.42-57.31)	35.02(18.58-63.86)	312.68(182.21-525.4)	714.23(427.82-1192.53)	38.39(25.33-54.69)	5.84(2.97-10.54)	11.49(5.96-20.77)	29.58(-10.39-85.82)	213.49(110.31-375.85)	452.2(242.84-777.5)	53.4(6.14-122.8)
Nauru	Both	0.19(0.05-0.5)	0.22(0.06-0.57)	7.05(-1.21-16.35)	3.62(1.96-6.6)	4.59(2.45-8.63)	22.68(15.13-30.22)	0.35(0.22-0.51)	0.53(0.33-0.77)	67.04(33.91-102.51)	16.22(10.5-22.86)	24.93(15.55-35.61)	69(33.61-107.92)
Nauru	Female	0.08(0.02-0.22)	0.09(0.02-0.25)	3.99(-5.66-15.1)	1.7(0.94-3.2)	2.17(1.21-4.05)	21.19(12.75-30.35)	0.15(0.09-0.24)	0.23(0.12-0.39)	51.72(12.82-99.75)	7.14(4.31-10.89)	10.5(5.86-17.98)	55.68(11.4-114.7)
Nauru	Male	0.11(0.03-0.29)	0.13(0.04-0.34)	9.41(-1.08-21.89)	1.92(0.99-3.65)	2.42(1.18-4.74)	24.28(12.85-35.35)	0.19(0.1-0.29)	0.3(0.15-0.48)	78.21(26.56-129.49)	9.08(4.81-13.69)	14.43(7.07-22.61)	79.12(25.98-134.84)
Nepal	Both	270.17(94.7-625.73)	521.06(207.46-1154.05)	36.91(22.04-63.26)	6949.38(4307.37-11693.18)	14647.49(9227.49-24282.88)	38.43(27.08-51.51)	116.05(64.2-198.41)	329.7(184.51-556.08)	226.33(150.72-311.16)	4587.09(2684.83-7484.01)	11965.98(7007.01-20005.79)	334.92(220.02-461.81)
Nepal	Female	94.33(28.5-244.63)	207.77(70.1-503.09)	51.45(32.68-87.21)	3191.21(1876.63-5461.36)	7332.85(4459.89-12307.92)	42.61(28.29-60.65)	57.32(29.55-97.49)	175.52(88.23-290.56)	268.72(167.97-393.64)	2304.55(1228.09-3833.23)	6473.78(3395.27-10609.01)	367.61(229.77-546.32)
Nepal	Male	175.84(60.52-413.68)	313.29(121.44-729.54)	31.02(16.28-55.27)	3758.17(2089.09-6556.54)	7314.64(4037.96-13011.29)	35.73(22.38-50.09)	58.73(30-106.42)	154.19(79.12-285.17)	189.45(109.31-282.2)	2282.54(1276.56-3989.37)	5492.19(3004.73-9953.85)	301.85(181.73-437.9)
Netherlands	Both	160.76(105.9-242.3)	414.36(289.6-574.3)	124.89(73.38-192.22)	10490.82(7465.66-15288.21)	19421.59(14677.15-25479)	59.35(33.89-90.25)	29.23(16.63-47.62)	50.74(27.26-86.4)	40.46(20.7-64.46)	1009.95(681.79-1485.18)	1673.13(1118.82-2432.32)	46.06(29.13-66.78)
Netherlands	Female	59.05(32.23-98.34)	154.47(89.63-240.9)	129.94(77.33-196.79)	5659.8(3701.04-8825.14)	10518.71(7036.76-15309.06)	61.48(32.27-96.26)	16.05(8.84-28)	26.1(13.83-46.88)	25.13(5.7-49.12)	480.38(322.29-724.19)	774.14(520.17-1135.88)	35.38(19.46-55.24)
Netherlands	Male	101.7(59.93-159.99)	259.89(168.99-395.61)	121.11(65.33-201.54)	4831.01(3159.17-7214.84)	8902.88(6320.73-12507.79)	57.04(28.86-96.01)	13.17(7.57-21.49)	24.64(13.24-42.09)	58.89(30.1-91.79)	529.57(355.16-763.5)	898.99(599.55-1305.71)	56.99(33.17-84.97)
New Zealand	Both	95.72(66.3-135.68)	166.78(119.6-226.04)	34.97(17.69-54.22)	4057.41(3077.7-5394.41)	5797.55(4524.67-7502.17)	9.12(-2.82-23.36)	4.57(2.58-7.61)	8.22(4.59-13.7)	36.85(16.71-58.56)	269.48(180.37-380.86)	466.04(311.87-663.79)	48.03(30.14-67.18)
New Zealand	Female	40.53(23.97-63.8)	70.75(43.44-106.56)	34.17(14.22-60.35)	2286.68(1571.73-3308.51)	3302.72(2395.34-4604.7)	8.13(-6.09-26.4)	2.31(1.34-3.74)	3.96(2.33-6.52)	26.68(4.42-53.4)	134.72(89.24-191.27)	230.06(153.53-326.49)	38.94(21.38-59.95)
New Zealand	Male	55.19(36.34-81.07)	96.03(63.69-140.5)	35.93(15.42-60.86)	1770.73(1280.54-2485.96)	2494.84(1879.44-3320.06)	9.9(-4.15-25.98)	2.26(1.22-3.9)	4.26(2.3-7.42)	47.29(22.48-77.39)	134.76(88.37-197)	235.98(153.41-357.65)	57.26(29.73-86.92)
Nicaragua	Both	91.68(27.9-252.5)	174.32(63.89-436.23)	28.08(14.18-62.28)	1651.47(816.04-3497.43)	3615.3(1991.33-7304.77)	33.25(20.72-51.69)	44.06(28.83-62.02)	236.48(139.07-367.32)	239.68(184.38-291.38)	1759.69(1198.57-2441.74)	8402.75(5170.67-12723.53)	372.34(279.43-466.46)
Nicaragua	Female	21.55(5.41-61.46)	34.48(10.67-88.9)	7.91(-7.16-47.91)	623.19(322.5-1206.88)	1273.17(709.08-2260.24)	23.77(8.39-47.75)	15.55(10.21-22.56)	77.92(46.71-122.08)	207.46(139.9-281.82)	644.63(438.9-909.42)	2657.56(1620.87-4042.87)	298.65(199.75-411.99)
Nicaragua	Male	70.14(20.48-189.44)	139.84(48.1-347.56)	34.14(18.02-68.23)	1028.28(454.87-2364.78)	2342.13(1140.73-5160.39)	39.35(25.49-61.01)	28.52(18.34-40.83)	158.56(93.71-245.19)	260.47(206.29-315.75)	1115.06(734.94-1552.37)	5745.2(3452.04-8703.21)	419.83(324.35-521.91)

Niger	Both	167.09(60-372.84)	486.1(156.93-1137.49)	2.64(-8.35-10.88)	6541.84(3837.75-11041.16)	17573(9444.04-32369.86)	-6.37(-19.55-5.64)	63.67(39.38-97.6)	138.68(80.69-223.97)	74.3(41.58-119.71)	2753.85(1766.33-4075.31)	6051.1(3666.37-9587.86)	83.68(49.89-128.51)
Niger	Female	64.12(20.94-165.48)	178.65(54.56-475.68)	-0.82(-11-8.75)	3177.16(1769.7-5474.93)	8747.53(4560.17-16197.81)	-4.31(-17.47-9.08)	30.21(18.09-48.72)	63.75(35.98-104.74)	72.35(28.17-132.42)	1360.15(838.4-2093.93)	2821.14(1654.16-4507.73)	76.36(31.59-138.27)
Niger	Male	102.98(34.91-233.46)	307.45(96.94-721.02)	4.37(-8.64-14.77)	3364.68(1837.59-5959.46)	8825.47(4321.37-17013.36)	-8.3(-23.2-5.89)	33.46(19.89-53.43)	74.93(41.8-123.42)	75.94(32.95-141.3)	1393.71(877.13-2138.77)	3229.96(1882.72-5234.14)	90.69(45.78-156.54)
Nigeria	Both	1756.3(1424.31-2170.01)	4849.24(3953.21-5947.28)	24.59(12.5-38.48)	52121.59(45239.98-59997.52)	173645.46(149631.48-201604.5	41.43(30.95-53.28)	550.92(332.66-876.62)	1040.88(622.81-1613.1)	58.68(19.57-111.25)	21908.23(13804.35-32810.67)	46184.1(29938.51-70058.26)	78.89(32.1-144.87)
Nigeria	Female	773.53(614.7-964.36)	2105.61(1696.15-2634.15)	19.85(6.29-35.63)	25370.96(21732.61-29556.81)	96838.74(82960.96-113862.05)	55.42(42.26-71.59)	256.72(142.4-414)	494.03(281.62-806.28)	59.05(5.5-125.68)	10274.27(6050.86-16213.96)	21985.05(13460.01-35260.04)	75.12(13.56-157.61)
Nigeria	Male	982.77(797.93-1202.65)	2743.63(2213.91-3380.34)	29.4(16.6-44)	26750.63(23175.89-30966.34)	76806.72(66182.78-89402.13)	27.31(17.29-37.8)	294.2(165.91-503.46)	546.85(305.64-953.57)	58.04(8.43-125.3)	11633.96(6859.49-19350.25)	24199.05(14632.32-40448.68)	82.16(24.03-166.43)
Niue	Both	0.03(0.01-0.08)	0.02(0.01-0.06)	-3.67(-12.85-13.71)	0.74(0.44-1.34)	0.61(0.38-1.04)	13.89(6.53-24.23)	0.07(0.04-0.1)	0.08(0.05-0.13)	51.53(16.96-88.1)	2.78(1.79-4.08)	3.05(1.77-4.86)	42.89(4.62-85.37)
Niue	Female	0.01(0-0.03)	0.01(0-0.02)	-2.87(-11.88-13.47)	0.34(0.2-0.61)	0.28(0.18-0.49)	13.01(4.74-24.38)	0.03(0.02-0.05)	0.04(0.02-0.06)	56.68(15.96-98.83)	1.24(0.77-1.92)	1.32(0.75-2.19)	40.49(-0.92-89.59)
Niue	Male	0.02(0-0.04)	0.01(0-0.03)	-4.72(-15.78-15.58)	0.39(0.22-0.74)	0.33(0.2-0.59)	14.67(5.61-27.45)	0.04(0.02-0.06)	0.05(0.02-0.08)	45.73(2.35-93.27)	1.54(0.92-2.45)	1.73(0.94-2.82)	44.88(0-98.29)
North Macedonia	Both	20.47(10.41-41.59)	35.63(21.97-61.31)	72.82(37.57-133.95)	911.06(610.53-1376.19)	1479.05(1047.73-2104.84)	50.62(32.6-71.29)	4.93(2.88-7.75)	7.74(4.13-13.21)	8.51(-11.73-32.31)	217.12(135.51-331.72)	315.85(194.61-506.18)	29.56(10.75-51.59)
North Macedonia	Female	7.38(2.88-16.9)	13.33(6.28-26.57)	78.86(39.78-146.36)	461.69(290.82-714.64)	797.32(506.24-1213.79)	61.44(41.12-87.3)	2.1(1.22-3.38)	3.46(1.87-5.85)	11.39(-13.45-43.64)	93.4(58.64-142.02)	137.2(84.65-217.51)	30.87(6.14-58.33)
North Macedonia	Male	13.09(6.41-26.27)	22.3(13.14-37.55)	69.57(33.06-131.57)	449.37(290.21-720.83)	681.73(468.58-1037)	39.7(20.12-63.43)	2.82(1.58-4.56)	4.28(2.27-7.29)	6.52(-15.08-31.74)	123.72(75.94-190.22)	178.65(107.55-283.44)	28.58(7.5-53.38)
Northern Mariana Islands	Both	0.56(0.18-1.39)	0.5(0.22-1.15)	-14.92(-27.55-22.13)	15.32(9.64-25.12)	17.54(11.84-27.7)	20.01(8.96-36.25)	1.39(0.91-2.03)	2.48(1.47-3.81)	-6.21(-33.13-26.13)	65.74(43.03-95.83)	92.17(56.61-141.01)	8.07(-23.39-49.53)
Northern Mariana Islands	Female	0.24(0.06-0.64)	0.19(0.06-0.47)	-23.73(-36.99-7.16)	7.21(4.21-12.07)	8.11(5.16-12.83)	16.15(1.9-37.32)	0.66(0.43-0.98)	1.17(0.68-1.83)	-10.62(-37.86-22.22)	31.27(20.4-46.1)	43.44(25.64-66.64)	4.67(-29.9-51.67)
Northern Mariana Islands	Male	0.31(0.11-0.75)	0.31(0.14-0.68)	-8.42(-24.11-29.07)	8.11(5.04-13.45)	9.43(6.12-15.3)	23.63(12.01-39.17)	0.74(0.46-1.11)	1.31(0.77-2.03)	-3.52(-33.23-34)	34.46(21.08-51.17)	48.74(30.28-75.29)	10.71(-23.07-58.05)
Norway	Both	49.36(41.83-57.21)	96.68(83.01-112.91)	56.89(44.28-71.45)	2599.56(2302.89-2934.54)	4715.23(4140.54-5324.19)	43.25(32.49-55.51)	7.62(4.29-12.83)	12.05(6.98-19.79)	72.74(54.16-98.4)	297.05(205.03-414.66)	441.58(305.5-612.91)	51.85(41.85-63.68)
Norway	Female	18.24(14.8-21.97)	36.94(30.17-44.75)	64.6(48.51-84.11)	1320.71(1135.83-1526.78)	2524.24(2146.04-2948.84)	54.55(40.72-71.88)	3.24(1.83-5.54)	5.32(3.1-8.86)	69.42(48.26-98.53)	123.49(86.26-172.08)	193.37(134.79-264.49)	50.95(39.49-64.08)
Norway	Male	31.12(26.34-36.53)	59.74(50.87-70.36)	51.4(38.57-65.99)	1278.85(1118.89-1447.38)	2190.99(1923-2489.05)	32(22.67-42.42)	4.38(2.48-7.27)	6.73(3.86-11.07)	77.51(55.91-107.95)	173.56(117.01-243.36)	248.22(170.95-347.38)	54.4(42.29-68.35)
Oman	Both	24.77(8.98-56.69)	102.79(53.89-189.16)	75.99(33.85-179.18)	706.6(425.28-1181.34)	3567.57(2349.73-5431.81)	116.08(81.52-157.75)	3.81(2.25-6.27)	9.57(6.08-14.88)	99.23(53.77-154.82)	177.62(111.57-270.1)	558.96(375.53-820.64)	129.58(75.99-187.97)
Oman	Female	10.17(2.8-25.08)	32.29(12.65-69.62)	55.12(23.94-141.6)	291.56(159.01-505.87)	1382.57(828.56-2181.72)	136.92(94.06-193.02)	1.54(0.88-2.51)	3.87(2.47-5.68)	115.92(61.81-179.28)	71.14(44.79-108.51)	211.84(144.4-299.54)	140.51(75.91-215.68)
Oman	Male	14.6(5.49-31.63)	70.5(36.84-129.39)	85.86(37.75-189.69)	415.04(236.68-713.54)	2185.01(1366.66-3524.77)	104.12(71.34-144.27)	2.28(1.31-3.95)	5.71(3.31-10.12)	89.56(34.21-172.77)	106.48(66.18-170.58)	347.12(220.9-547.21)	123.25(64.29-196.73)
Pakistan	Both	1644.61(893.22-2966.09)	4088.08(2297.71-7059.88)	34.7(23.87-49.96)	43338.08(32130-59971.35)	117863.74(87241.77-164756.04)	40.57(32.09-50.13)	908.36(536.76-1492.27	3470.55(2054.07-5375.9	203.71(139.51-279.59)	35556.24(21591.08-56179.82)	143315.55(89195.78-220097.04)	238.15(163.15-327.69)
Pakistan	Female	646.43(314.1-1266.2)	1569.25(803.03-2883.4)	30.52(18.63-46.57)	21056.68(15019.2-29980.9)	58922.77(41703.75-83680.41)	40.39(30.02-53.38)	397.12(193.77-650.35)	1590.35(809.01-2594.63)	212.61(135.23-337.68)	15837.9(8054.7-25351.61)	65737.71(34893.27-106464.28)	245.58(150.58-392.12)
Pakistan	Male	998.18(535.77-1748.79)	2518.83(1393.96-4246.38)	37.79(25.19-54.51)	22281.4(16135.17-31910.12)	58940.97(42320.87-85666.09)	40.61(30.6-51.82)	511.24(273.98-924.33)	1880.2(1081.06-3189.18)	195.62(115.78-298.64)	19718.34(11173.74-34207.06)	77577.84(46597.52-126658.16)	231.12(138.48-356.48)
Palau	Both	0.22(0.06-0.56)	0.22(0.08-0.52)	-22.04(-31.07-5.5)	5.55(3.18-9.76)	6.91(4.62-11.07)	4.62(-7.44-25.06)	0.69(0.44-1.02)	1.65(1.01-2.45)	49.61(19.04-81.42)	29.92(19.64-43.32)	66.66(42.12-97.8)	60.96(24.86-102.57)
Palau	Female	0.08(0.02-0.22)	0.08(0.03-0.22)	-13.48(-24.72-14.54)	2.47(1.44-4.08)	2.99(1.95-4.61)	9.21(-3.42-29.14)	0.24(0.15-0.36)	0.49(0.29-0.75)	33.85(3.77-67.33)	9.95(6.3-15.05)	18.79(11.47-28.23)	44.1(7.36-88.01)
Palau	Male	0.14(0.04-0.36)	0.14(0.05-0.33)	-28.44(-37.91-0.66)	3.08(1.64-5.89)	3.91(2.5-6.63)	0.27(-13.4-23.2)	0.45(0.29-0.66)	1.15(0.72-1.72)	57(18.91-98.91)	19.96(12.86-29.18)	47.87(30.44-69.78)	66.9(23.56-116.49)
Palestine	Both	51.7(17.41-129.81)	137.74(58.41-291.83)	14.29(-3.11-59.25)	1001.45(538.22-1910.8)	3908.15(2283.99-6791.98)	63.52(41.46-94.96)	13.92(8.4-21.77)	29.25(18.59-42.98)	54.89(27.21-87.57)	555.06(348.22-839.37)	1278.67(862.74-1818.14)	77.07(35.27-131.69)
Palestine	Female	21.87(6.07-60.77)	55.19(18.81-137.99)	8.4(-6.29-45.43)	490.96(261.97-970.79)	1998.49(1128.56-3561.51)	71.94(45.3-106.42)	6.73(4.03-10.52)	12.27(7.99-18.1)	34.22(6.88-69.59)	277.53(174.34-427.48)	554.77(377.2-791.15)	53.87(14.36-108.74)
Palestine	Male	29.83(9.24-72.19)	82.55(35.09-172.98)	18.53(-1.77-69.19)	510.49(245.41-991.61)	1909.67(1028.17-3411.14)	55.48(34.79-86.82)	7.19(4.19-11.34)	16.98(10.79-25.75)	74.26(38.78-122.86)	277.53(169.12-421.28)	723.9(478.13-1055.37)	100.23(53.8-166.55)
Panama	Both	34.6(12.64-80.4)	75.73(30.28-162.09)	30.6(17.93-53.11)	920.3(552.48-1584.43)	1971.94(1210.21-3325.38)	25.12(14.12-38.21)	13.47(9-18.77)	60.03(35.63-95.81)	123.55(96.07-152.63)	545.35(384.18-737.67)	2181.3(1348.91-3455.7)	142.95(98.47-188.86)
Panama	Female	10.47(3.4-26.53)	22.01(7.87-52.54)	24.15(9.85-50.11)	416.48(241.79-700.33)	863.23(521.07-1416.41)	19.51(7.86-35.74)	5.57(3.7-7.83)	24.21(14.14-39.19)	111.44(77.55-147.46)	228.48(159.86-313.8)	868.59(532.05-1371.88)	128.58(79.91-180.53)
Panama	Male	24.14(8.3-54.74)	53.72(20.67-116.2)	34.22(19.72-59.09)	503.82(271.93-913.61)	1108.7(618.54-1974.75)	30.16(16.44-46.41)	7.91(5.28-11.25)	35.82(21.08-56.61)	132.72(102.91-166.86)	316.87(219.63-429.86)	1312.71(816.03-2069.09)	153.9(107.76-202.85)
Papua New Guinea	Both	43.06(11.5-108.58)	123.31(33.54-314.96)	17.06(6.9-28.9)	1074.61(626.07-1806.93)	2943.41(1706.03-5015.04)	14.54(8-22.39)	41.64(29.27-57.4)	126.89(85.05-187.35)	41.68(15.92-73.12)	2053.46(1450.04-2754.14)	6175.76(4287.18-8894.35)	51.3(22.76-87.2)
Papua New Guinea	Female	15.05(3.41-38.24)	43.52(10.04-112.39)	18.83(5.86-35.48)	491.5(275.95-831.73)	1351.01(758.8-2254.72)	13.99(6.03-24.11)	14.03(9.15-20.36)	42.11(27.57-62.85)	38.45(2.81-89.95)	702.16(467.66-988.35)	2079.35(1416.07-3020.3)	47.25(9.61-103.95)
Papua New Guinea	Male	28.01(7.16-73.13)	79.79(20.72-208.92)	15.88(2.98-29.85)	583.11(313.22-1076.99)	1592.39(854.46-2987.67)	15.1(6.38-24.69)	27.6(18.12-39.25)	84.78(55.93-127.46)	43.74(12.56-82.03)	1351.3(896.11-1859.68)	4096.41(2794.58-6006.34)	54.07(21.63-96.93)
Paraguay	Both	54.02(20.94-130.26)	131.35(62.42-267.22)	44.17(18.82-98.82)	1574.77(967.15-2558.35)	4932.56(3206.34-7478.36)	82.88(61-112.83)	19.69(13.02-28.14)	109.88(63.51-176.75)	172.57(129.66-217.29)	787.55(543.51-1084.87)	3964.35(2429.84-6225.58)	221.88(159.64-294.34)
Paraguay	Female	23.45(8.84-53.11)	68.18(30.34-132.88)	71.99(35.03-140.23)	971.38(599.99-1547.96)	3469.78(2178.8-5298.83)	108.93(82.01-142.86)	9.33(6.19-13.54)	49.04(27.62-80.07)	177.53(126.13-235.35)	386.62(268.48-536.57)	1816.8(1121.4-2846.8)	215.48(146.21-302.8)

Paraguay	Male	30.57(10.28-75.09)	63.17(25.29-141.87)	22.79(4.24-62.05)	603.39(315.59-1130.55)	1462.78(821.49-2648.39)	41.28(26.01-62.1)	10.35(6.57-15.18)	60.84(34.91-98.93)	169.65(113.35-223.45)	400.93(265.38-569.8)	2147.55(1293.88-3375.53)	228.4(149.7-311.58)
Peru	Both	273.01(106.17-604.59)	600.13(288.63-1161.85)	39.38(16.5-90.66)	12047.8(7119.31-18628.88)	25383.17(16171.22-38228.37)	35.04(18.9-55.17)	149.59(97.12-216.5)	317.05(185.31-513.92)	94.69(62.76-130.77)	5970.22(4021.87-8346.53)	11567.59(7161.59-17842.32)	138.64(89.64-197.93)
Peru	Female	108.16(37.4-262.29)	252.95(108.93-544.08)	49.17(21.86-114.37)	6169.79(3457.48-10628.1)	13470.41(8115.72-21446.03)	40.76(19.39-68.27)	72.12(46.92-105.35)	162.97(95.81-265.91)	98.91(57.46-142.11)	2928.15(1978.98-4126.87)	5937.96(3662.56-9171.1)	142.97(83.14-212.41)
Peru	Male	164.85(63.27-369.49)	347.18(161.87-674.21)	32.72(11.06-81.16)	5878.02(3171.15-9793.56)	11912.76(7011.14-18906.97)	29.11(14.42-48.89)	77.47(49.78-112.07)	154.09(86.65-253.11)	89.35(57.1-128.94)	3042.07(2074.85-4269.22)	5629.63(3468.64-8775.11)	133.87(84.47-194.63)
Philippines	Both	874.7(701.04-1087.82)	2126.79(1739-2602.94)	47.41(38.11-58.16)	24553.23(21584-28067.17)	66055.25(58096.45-75681.19)	53.83(44.86-63.89)	1334.81(969.56-1736.0	4344.46(2906.78-6003.9	88.42(65.66-119.98)	60032.21(45107.3-76323.46)	185069.09(127749.93-251565)	133.87(97.57-178.63)
Philippines	Female	353.92(277.99-448.39)	811.52(646.66-1015.33)	39.81(30.76-50)	11998.62(10518.01-13854.67)	31606.28(27612.89-36759.61)	51.29(41.95-63.45)	598.65(433.92-776.85)	1849.41(1194.73-2708.4	70.77(45.65-100.08)	26873.56(19959.83-34214.65)	78152.56(51943.74-112127.41)	114.56(68.33-169.85)
Philippines	Male	520.79(415.28-654.03)	1315.27(1068.28-1610.18)	52.28(42.16-63.86)	12554.61(11018.22-14500.84)	34448.97(30253.89-39658.12)	56.2(47-66.33)	736.16(520.27-980.45)	2495.05(1564.43-3627.4	102.36(75.1-149.68)	33158.65(24217.68-43498.78)	106916.53(69855.64-149552.99)	149.06(103.41-210.13)
Poland	Both	398.86(312.8-511.06)	489.66(405.73-596.99)	32.59(14.68-53.97)	16726.61(14252.44-19612.93)	25225.8(21565.33-29984.07)	48.97(35.07-64.91)	177.97(112.11-264.99)	105.85(62.12-170.69)	-43.77(-53.08--31.57)	6924.51(4574.83-9839.03)	3930.66(2522.91-5775.35)	-37.71(-45.93--27.12)
Poland	Female	149.49(111.61-199.87)	204.37(159.19-257.51)	45.7(24.87-71.41)	8860.52(7425.98-10544.84)	15309.03(12745.04-18609.77)	69.64(52.8-89.16)	79.36(50.57-119.07)	48.61(27.92-82.03)	-44.83(-56.11--28.89)	2963.3(1966.32-4170.98)	1715.94(1140.01-2535.78)	-38.93(-49.36--25.68)
Poland	Male	249.37(194.42-320.79)	285.29(233.55-347.41)	25.38(8.71-45.91)	7866.09(6598.25-9388.11)	9916.77(8462.97-11642.73)	25.36(12.93-38.2)	98.61(61.25-149.31)	57.24(31.25-96.52)	-42.76(-51.51--31.2)	3961.21(2572-5730.71)	2214.72(1356.15-3331.9)	-36.76(-44.87--26.95)
Portugal	Both	85.41(53.69-135.34)	175.35(120.59-251.15)	102.15(53.1-165.44)	4383.99(3169.26-6124.23)	7846.26(6065.17-10081.64)	67.7(41.47-100.38)	50.5(29.66-79.63)	72.97(39.31-124.14)	23.33(-4.48-62.02)	1580.44(1041.29-2258.81)	1824.36(1206.15-2693.19)	19.93(1.44-43.51)
Portugal	Female	31.25(17.19-53.41)	66.51(38.2-106.71)	105.3(59.8-169.08)	2241.37(1522.71-3302.72)	4033.88(2868.8-5561.68)	67.1(39.95-102.61)	22.38(13.09-36.23)	37.65(19.97-65.24)	36.81(2.1-83.65)	661.82(439.02-972.48)	851.66(556.59-1290.33)	25.46(4.96-54)
Portugal	Male	54.16(30.24-90.14)	108.84(68.2-166.36)	102.58(48.74-172.64)	2142.62(1449.99-3150.95)	3812.38(2772.05-5139.47)	68.48(38.78-107.04)	28.11(16.57-43.78)	35.32(19.73-59.53)	12.26(-12.81-45.55)	918.61(601.42-1322.83)	972.7(638.47-1409.51)	16.86(-1.59-39.33)
Puerto Rico	Both	47.89(21.38-103.16)	56.99(33.67-99.33)	13.54(-12.45-65.69)	1702.73(1165.47-2544.41)	2173.97(1642.06-2977.99)	29.06(11.19-52.48)	51.71(33.66-73.15)	76.66(43.92-121.09)	14.66(0.09-30.72)	1953.74(1322.97-2639.72)	2588.25(1567.25-4011.55)	30.07(9.68-53.06)
Puerto Rico	Female	15.05(5.49-37.39)	17.89(8.66-36.86)	11.1(-15.99-75.03)	739.99(497.03-1158.06)	970.02(694.8-1385.5)	30.07(12.89-51.85)	20.67(13.63-30.17)	32.1(18.39-51.73)	2.53(-11.88-18.6)	768.51(515.32-1053.33)	1054.71(631.87-1635.92)	19.84(-0.68-42.34)
Puerto Rico	Male	32.84(14.56-68.75)	39.09(21.79-66.78)	16.31(-10.88-69.25)	962.74(623.54-1534.76)	1203.95(863.22-1753.05)	29.03(8.69-59.02)	31.05(20.02-43.65)	44.56(25.45-71.19)	25.94(9.17-44.42)	1185.23(802.37-1613.22)	1533.54(928.56-2371.32)	39.85(18.58-63.52)
Qatar	Both	5.68(2.1-13.28)	52.83(25.91-103.97)	52.83(18.76-123.78)	167.52(105.14-277.29)	1826.19(1209.46-2869.74)	67.8(47.73-94.41)	1.65(0.97-2.63)	7.83(4.65-12.12)	41.44(10.82-73.74)	74.23(47.34-110.73)	408.89(266.01-607.75)	25.95(-2.83-59.17)
Qatar	Female	2.11(0.55-5.64)	13.45(4.55-33.61)	35.99(13.57-95.21)	59.19(31.81-108.05)	513.06(301.41-874.86)	74.35(49.29-109.21)	0.6(0.35-0.98)	2.13(1.26-3.28)	44.23(1.88-93.46)	25.96(16.67-39.61)	104.25(67.55-154.78)	18.16(-16.24-61.82)
Qatar	Male	3.57(1.39-7.95)	39.38(19.02-72.53)	60.47(22.84-129.75)	108.33(66.91-173.63)	1313.14(845.5-2053.65)	66.89(45.19-92.41)	1.05(0.6-1.73)	5.7(3.34-8.94)	42.79(10.63-82.28)	48.26(29.63-74.24)	304.64(195.37-457.15)	29.26(-3.23-70.54)
Republic of Korea	Both	500.54(256.14-952.16)	716.13(465.86-1067.82)	24.49(-14.7-92.15)	30752.6(19518.88-47048.97)	41020.28(29821.75-55030.31)	9.97(-11.03-37.68)	337.53(233.98-452.13)	398.7(261.84-586.09)	-12.04(-29-9.73)	14206.45(10241.1-18617.66)	13546.76(8907.95-18648.58)	-10.21(-24.68-7.17)
Republic of Korea	Female	173.87(79.17-341.08)	256.44(138.46-426.82)	29.13(-11.32-95.34)	17016.28(10083.69-28007.18)	22046.53(14996.98-31718.24)	7.52(-12.74-34.8)	136.03(94.45-186.47)	158.23(105.85-241.87)	-19.75(-40.05-6.41)	5713.74(4140.53-7496.71)	5132.06(3492.67-7232.66)	-20.87(-35.48--2.9)
Republic of Korea	Male	326.66(157.56-631.5)	459.69(282.18-716.2)	21.68(-18.79-94.52)	13736.32(8306.72-21861.7)	18973.75(13364.67-26145.49)	13.12(-10.59-49.65)	201.5(136.9-271.97)	240.47(153.46-348.58)	-5.48(-24.15-18.52)	8492.72(6052.92-11108.51)	8414.7(5455.05-11623.18)	-1.45(-19.71-20.71)
Republic of Moldova	Both	36.27(15.96-75.86)	50.88(28.83-87.41)	70.27(32.55-146.42)	2276.97(1567.44-3423.59)	3491.68(2467.33-5047.97)	82.78(61.46-111.61)	8.33(5.53-11.94)	19.54(12.36-28.73)	144.05(112.06-183.29)	307(215.71-424.7)	674.81(457.33-962.33)	182.47(146.55-225.07)
Republic of Moldova	Female	13.98(5.18-31.38)	19.17(9.02-36.05)	64.44(27.14-139.27)	1192.72(739.47-1876.78)	1906.19(1213.69-2966.58)	90.48(66.42-123.58)	3.76(2.42-5.37)	9.73(6.01-14.56)	173.41(131.58-228)	127.67(88.22-177.62)	304.57(201.02-438.69)	210.56(159.97-270.2)
Republic of Moldova	Male	22.29(9.63-47.65)	31.7(17.38-58.03)	75.16(33.62-155.78)	1084.24(699.49-1702.2)	1585.48(1044.07-2409.21)	74.3(49.74-106.74)	4.57(3.07-6.62)	9.8(6.27-14.59)	120.09(88.15-159.24)	179.32(125.55-249.8)	370.25(248.79-533.87)	162.37(128.45-203.29)
Romania	Both	219.38(109.89-444.24)	337.63(210.92-532.09)	97.74(43.82-185.92)	10119.01(6872.94-14843.09)	15146.51(11046.9-20935.11)	82.22(54.06-118.06)	43.95(22.8-71.46)	54.98(31.07-90.39)	16.97(-8.43-86.04)	1819.13(1070.11-2741.26)	2372.87(1490.64-3508.4)	58.7(30.9-129.27)
Romania	Female	85.64(41.12-179.9)	132.98(71.17-233.86)	95.19(45.81-178.15)	5655.54(3670.39-8693.54)	8789.72(5820.94-13260.69)	86.86(57.98-123.76)	22.2(11.19-36.25)	26.51(14.78-44.4)	6.56(-18.52-77.82)	865.78(483.82-1318.31)	1066.27(677.91-1592.64)	46.44(18.9-124.95)
Romania	Male	133.74(62.92-270.23)	204.65(121.52-336.97)	101.33(43.5-200.38)	4463.47(2783.7-7052.17)	6356.79(4432.92-9099.12)	75.71(44.2-118.29)	21.75(11.44-35.61)	28.47(15.55-48.03)	27.65(-1.14-96.98)	953.35(564.96-1467.19)	1306.6(806.4-1967.79)	69.95(37.66-138.38)
Russian Federation	Both	3317.21(2826.75-3872.98)	5613.78(4766.89-6555.65)	75.25(59.83-91.65)	173289.19(154467.97-197438.7	281568.04(247351.16-324909.1	67.29(54.25-81.89)	877.75(595.57-1262.42	867.4(552.25-1301.59)	-10.46(-23.59-6.01)	36030.7(26176.15-49490.2)	33057.4(23384.84-45944.73)	-5.42(-15.79-7.73)
Russian Federation	Female	1336.24(1120.34-1574.05)	2281.2(1908.45-2700.05)	72.89(56.64-91.91)	91583.65(80956.1-105491.39)	161160.39(138745.29-187845.5)	80.03(63.99-100.43)	467.84(306.87-688.26)	477.66(291.75-741.6)	-6.69(-22.6-11.74)	17724.01(12596.06-24943.7)	16266.49(11212.14-23588.53)	-6.3(-19.2-8.47)
Russian Federation	Male	1980.97(1671.45-2321.83)	3332.58(2834.16-3933.55)	78.82(62.07-96.86)	81705.54(72316.19-93494.56)	120407.65(105314.37-138941.5	52.87(40.81-64.93)	409.91(274.93-587.65)	389.74(240.93-592.8)	-14.76(-26.56-0.29)	18306.69(13068.78-25217.29)	16790.92(11631.33-23579.63)	-4.77(-14.89-8.34)
Rwanda	Both	89.97(29.96-210.12)	155.8(62.38-340.02)	8.99(-3.68-36.53)	2570.01(1586.28-4125.79)	7020.24(4487.46-10751.16)	57.87(40.15-78.82)	60.02(36.97-88)	75.1(45.37-115.27)	102.87(60.35-157.82)	2400.02(1558.53-3462.36)	3049.52(1924.42-4430.81)	125.03(72.38-198.75)
Rwanda	Female	31.06(9.23-79.61)	54.19(19.88-124.62)	11.31(-6.1-49.8)	1287.72(756.58-2088.24)	3879.52(2308.6-6066.9)	72.62(48.64-101.25)	30.8(19.2-46.13)	38.1(21.53-59.82)	95.29(32.71-168.54)	1270.12(807.29-1856.48)	1516.83(872.34-2284.8)	107.91(38.99-192.68)
Rwanda	Male	58.92(19.44-137.47)	101.61(38.7-227.96)	6.97(-5.74-30.63)	1282.29(735.58-2230.89)	3140.72(1881.37-5142.97)	42.87(28.4-60.05)	29.22(17.14-46.25)	37(22.16-56.82)	110.43(55.18-193.18)	1129.91(686.26-1754.25)	1532.7(945.94-2262.44)	144.12(75.39-247.42)
Saint Kitts and Nevis	Both	0.93(0.29-2.34)	1.58(0.71-3.23)	17.37(-5.99-90.34)	13.78(8.05-24.47)	30.48(21.41-46.3)	51.66(26.04-92.9)	0.99(0.66-1.46)	2.06(1.08-3.35)	101.83(33.55-168.17)	35.69(25.17-49.04)	72.34(36.82-117.21)	71.85(2.35-138.42)
Saint Kitts and Nevis	Female	0.34(0.09-0.94)	0.42(0.15-1)	-11.44(-25.38-34.89)	5.91(3.37-10.5)	13.09(8.6-20.01)	55.26(28.21-92.97)	0.48(0.32-0.72)	0.79(0.41-1.31)	98.45(30.65-165.98)	17.57(12.28-24.59)	27.31(13.46-43.77)	58.88(-9.37-122.68)
Saint Kitts and Nevis	Male	0.59(0.18-1.46)	1.16(0.51-2.37)	31.58(3.73-113.32)	7.87(4.35-14.42)	17.39(11.71-27.18)	48.08(21.98-92.87)	0.51(0.33-0.73)	1.27(0.66-2.08)	100.59(30.21-168.61)	18.13(12.31-25.31)	45.03(22.51-74.42)	79.38(7.98-145.87)
Saint Lucia	Both	1.76(0.54-4.47)	2.4(1.13-5.08)	3.6(-16.7-68.94)	40.29(22.35-72.74)	70.66(47.66-109.68)	36.12(12.57-73.96)	1.76(1.2-2.47)	4.42(2.73-6.57)	61.96(37.77-89.34)	68.32(47.6-93.37)	166.72(106.26-242.23)	83.8(52.03-118.68)
Saint Lucia	Female	0.63(0.17-1.79)	0.75(0.28-1.77)	-7.17(-24.31-45.55)	17.51(9.48-32.41)	29.56(18.51-47.92)	33.42(11.54-67.86)	0.78(0.52-1.1)	1.84(1.12-2.79)	67.92(33.36-103.34)	30.43(21.26-41.91)	69.14(42.91-101.28)	81.8(41.91-126.19)

Saint Lucia	Male	1.13(0.36-2.84)	1.65(0.8-3.44)	8.17(-15.54-78.74)	22.78(11.72-43.2)	41.1(26.11-65.7)	37.4(12.7-80.06)	0.98(0.66-1.37)	2.58(1.6-3.87)	56.1(33.32-83.35)	37.89(26.58-51.54)	97.59(62.22-143.14)	84.06(54.91-117.47)
Saint Vincent and the Grenadines	Both	1.33(0.42-3.2)	1.62(0.72-3.48)	13.48(-4.31-68.63)	34.03(18.84-60.58)	46.85(30.71-74.63)	33.94(14.7-64.68)	1.16(0.79-1.62)	3.09(1.93-4.51)	89.04(60.48-123.63)	46.04(32.26-61.68)	116.44(75.46-166.37)	122.65(83.9-168.11)
Saint Vincent and the Grenadines	Female	0.54(0.14-1.47)	0.6(0.2-1.47)	7.19(-7.55-47.58)	15.49(8.13-28.28)	21.05(12.78-34.52)	35.53(16.63-66.13)	0.56(0.38-0.8)	1.38(0.87-2.05)	105.93(73.48-145.25)	21.83(15.44-29.56)	52.6(34.29-76.49)	132.28(91.04-180.69)
Saint Vincent and the Grenadines	Male	0.79(0.25-1.87)	1.02(0.45-2.08)	16.28(-5.07-76.81)	18.53(9.75-34.21)	25.8(16.19-41.86)	32.08(11.51-67.07)	0.6(0.41-0.84)	1.71(1.07-2.49)	74.83(44.6-111.45)	24.21(16.79-32.17)	63.84(40.93-91.86)	114.65(73.07-163.13)
Samoa	Both	2.11(0.53-5.62)	3.13(0.87-8.35)	4.88(-3.75-18.29)	57.67(31.54-106.48)	84.76(48.36-151.01)	13.52(7.01-21.73)	3.58(2.23-5.5)	7(4.3-10.66)	49.57(12-95.69)	152.87(97.51-234.59)	295.83(181.99-453.33)	65.85(16.86-121.88)
Samoa	Female	0.9(0.21-2.56)	1.33(0.32-3.75)	3.96(-7.11-18.25)	26.73(14.47-49.13)	39.79(22.18-70.26)	12.58(5.26-22.03)	1.78(1.07-2.77)	3.44(2.07-5.25)	32.62(-7.02-92.24)	75.59(46.98-118.69)	144.04(86.08-221.21)	49.48(-0.64-120)
Samoa	Male	1.21(0.3-3.25)	1.79(0.5-4.7)	5.75(-5.43-23.73)	30.94(15.16-59.67)	44.97(23.27-85.78)	14.53(6.04-25.01)	1.8(1.09-2.77)	3.55(2.14-5.52)	65.32(17.62-117.99)	77.28(48.69-116.82)	151.79(92.04-239.7)	80.81(25.26-144.94)
San Marino	Both	0.28(0.19-0.41)	0.57(0.39-0.81)	44.91(24.66-66.03)	15.63(11.57-20.32)	27.47(20.97-35.16)	23.81(9.08-37.91)	0.03(0.02-0.05)	0.07(0.03-0.13)	22.67(0.05-50.75)	1.37(0.93-1.94)	2.58(1.67-3.86)	22.83(10.93-37.96)
San Marino	Female	0.11(0.06-0.18)	0.23(0.13-0.36)	45.29(24.15-70.16)	7.97(5.58-11.26)	15.04(10.63-21.2)	29.63(13.34-47.04)	0.01(0.01-0.02)	0.04(0.02-0.07)	28.53(-2.13-66.51)	0.59(0.4-0.87)	1.27(0.81-1.95)	31.57(14.27-53.17)
San Marino	Male	0.17(0.11-0.27)	0.35(0.22-0.52)	46.47(22.11-73.18)	7.66(5.26-10.94)	12.43(8.78-17.31)	17.52(2.69-33.48)	0.02(0.01-0.03)	0.03(0.02-0.06)	16.03(-9.8-49.95)	0.77(0.52-1.1)	1.31(0.83-1.95)	16.43(1.75-33.71)
Sao Tome and Principe	Both	2.84(0.91-7.05)	5.24(1.98-12.33)	14.96(2.6-41.99)	65(38.08-115.59)	148.64(91.27-252.75)	38.06(25.91-53.92)	1.39(0.81-2.15)	3.22(1.97-5.08)	151.53(99.42-251.2)	53.12(31.54-81.11)	131.53(81.89-199.15)	226.14(156.1-371.54)
Sao Tome and Principe	Female	1.2(0.39-2.99)	2.22(0.8-5.15)	17.19(2.85-50)	31.6(18.22-54.33)	74.99(44.25-123.23)	44.28(30.73-64.16)	0.77(0.45-1.21)	1.88(1.12-2.99)	171.05(108.13-284.82)	29.72(17.15-46.18)	77.17(47.19-121.08)	241.2(155.71-411.71)
Sao Tome and Principe	Male	1.64(0.49-4.1)	3.02(0.99-7.19)	12.63(0.24-37.27)	33.41(17.67-63.12)	73.65(40.9-135.76)	32.18(19.58-49.2)	0.62(0.37-0.99)	1.35(0.8-2.18)	130.57(70.9-229.59)	23.4(14.01-36)	54.36(33.64-84.64)	206.76(127.07-341.64)
Saudi Arabia	Both	281.98(108.94-627.18)	1328.73(755-2293.28)	122.67(55.31-277.28)	7097.55(4460.32-11732.84)	35682.52(25311.65-51053.53)	123.41(83.91-175.77)	128.29(74.08-204.81)	474.59(274.47-758.76)	138.26(76.64-219.12)	5352.56(3199.61-8316.25)	21548.33(13287.6-33767.51)	161.28(87.14-262.92)
Saudi Arabia	Female	119.18(38.68-285.19)	472.47(217.49-942.03)	97.74(41.81-240.02)	3327.35(1921.44-5562.15)	16243.33(10725.68-24753.68)	128.65(87.74-184.59)	49.76(28.38-80.64)	201.13(113.53-321.05)	221.03(116.17-344.99)	2200.83(1283.51-3500.42)	9420.02(5632.97-14666.3)	213.87(100.34-358.91)
Saudi Arabia	Male	162.79(65.35-351.61)	856.26(461.2-1431.22)	138.05(61.06-298.86)	3770.21(2250.74-6436.43)	19439.19(13469.31-28522.73)	120.08(78.07-178.73)	78.53(43.24-130.81)	273.46(153.87-454.15)	98.07(36.81-194.17)	3151.73(1836.09-5001.12)	12128.31(7268.8-19434.67)	129.79(56.41-239.31)
Senegal	Both	135.5(43.12-337.42)	249.71(88.67-612.66)	-5.32(-14.16-9.19)	3006.18(1708.32-5370.7)	6902.09(4023.99-12061.69)	16.8(7.53-28)	79.37(48.62-123.59)	158.38(94.54-243.2)	91.37(40.77-155.73)	3180.44(2025.86-4761.09)	6366.21(3795.11-9770.06)	124.9(57.89-201.08)
Senegal	Female	52.62(15.1-141.94)	90.29(29.14-234.08)	-8.48(-18.09-7.71)	1498.86(830.51-2702.75)	3435.08(1963-5889.9)	19.51(7.26-35.31)	40.58(23.47-68.83)	73.8(42.56-118.86)	70.31(20.57-136.85)	1682.61(957.16-2819.29)	2979.14(1737.45-4755.32)	95.25(34.6-177.73)
Senegal	Male	82.88(26.06-204.83)	159.42(54.63-385.78)	-4.93(-14.96-10.28)	1507.32(785.41-2834.56)	3467.02(1835.34-6336.49)	14.07(4.29-25.46)	38.79(23.67-60.34)	84.58(48.4-140.12)	113.49(51.61-198.22)	1497.82(947.03-2220.86)	3387.08(1971.1-5596.45)	158.52(79.19-270.15)
Serbia	Both	79.24(46.16-139.1)	136.52(88.67-208.72)	92.26(56.38-145.03)	4277.58(3053.98-6164.71)	6305.86(4607.71-8935.51)	58.24(40.31-79.63)	55.46(32.1-89.96)	67.08(36.87-115.77)	-0.39(-26.01-36.66)	1999.9(1196.65-3159.87)	2001.89(1202.63-3175.07)	6.14(-14.51-31.22)
Serbia	Female	26.81(12.47-52.3)	52.21(27.52-91.68)	119.24(77.78-179.76)	2108.81(1379.85-3302.94)	3449.79(2300.72-5316.95)	76.9(55.48-103.08)	23.1(13.16-37.29)	31.67(17.07-56.12)	7.63(-23.8-51.16)	810.11(486.47-1257.61)	876.86(522.48-1390.95)	9.1(-16.29-40.79)
Serbia	Male	52.42(28.59-90.39)	84.31(51.38-131.26)	77.76(42.42-130.63)	2168.77(1500.69-3221.58)	2856.07(2008.51-4110.56)	40.27(22.41-61.89)	32.36(18.16-54.99)	35.41(18.53-61.71)	-5.77(-30.92-28.32)	1189.8(694.59-1922.78)	1125.02(680.14-1800.68)	4.78(-17.5-32.55)
Seychelles	Both	0.72(0.25-1.72)	1.09(0.46-2.45)	7.52(-5.37-32.49)	26.39(16.73-42.87)	39.88(27.35-60.64)	7.98(-2.91-21.59)	1.58(1.09-2.16)	4.31(2.94-6.03)	88.75(64.71-115.09)	62.61(46.16-83)	166.02(118.9-227.69)	84.88(60.29-113.83)
Seychelles	Female	0.3(0.09-0.81)	0.44(0.15-1.12)	11.62(-1.17-35.3)	13.31(8.05-22)	19.17(12.48-30.24)	9.07(-3.01-23.18)	0.68(0.47-0.93)	1.77(1.17-2.59)	80.39(50.14-112.15)	26.99(19.61-35.47)	65.33(45.13-92.87)	75.7(41.06-112.7)
Seychelles	Male	0.42(0.15-1.01)	0.64(0.27-1.43)	3.28(-11.74-32.46)	13.08(7.92-21.63)	20.71(13.85-31.61)	6.91(-4.78-23.09)	0.9(0.62-1.25)	2.54(1.67-3.62)	94.93(64.6-134.8)	35.61(25.7-47.85)	100.69(69.64-139.46)	91.31(58.27-132.79)
Sierra Leone	Both	71.96(28.96-166.46)	178.18(67.13-411.65)	12.96(2.3-21.55)	2571.15(1596.9-3941.67)	6143.16(3595.05-9847.37)	6.68(-6.11-17.62)	31.41(19.06-49.31)	67.15(41.76-103.09)	95.55(53.81-152.09)	1240.8(780.6-1907.27)	2933.07(1871.69-4356.17)	128.59(74.88-199.43)
Sierra Leone	Female	28.72(9.6-69.03)	69.08(21.89-169.04)	11.87(-1.38-22.74)	1309.73(776.29-2055.07)	3030.79(1730.25-5049.17)	4.28(-10.09-19.09)	14.56(8.54-23.43)	33.74(19.74-52.14)	105.1(46.96-182.53)	601.17(361.01-943.62)	1488.11(887.44-2248.01)	137.34(67.13-232.23)
Sierra Leone	Male	43.24(16.82-100.41)	109.1(40.77-256.61)	12.8(1.75-23.66)	1261.43(728.5-2127.92)	3112.37(1761.64-5315.01)	9.11(-3.96-22.13)	16.85(10.09-26.17)	33.41(19.66-52.73)	86.55(41.64-150.28)	639.63(405.87-966.16)	1444.95(889.7-2169.6)	120.14(64.02-199.03)
Singapore	Both	34.43(16.59-72.28)	85.24(53.96-132.12)	37.74(-5.61-111.8)	2490.29(1629.19-3675.94)	5354.26(3847.78-7160.34)	14.54(-7-41.81)	28.35(18.98-39.17)	39.41(24.98-60.64)	-15.79(-29.37-1.66)	1172.11(822.94-1599.38)	1560.67(1010.19-2266.12)	-16.08(-29.68--0.12)
Singapore	Female	10.6(4.6-23.31)	30.23(16.16-53.1)	60.71(9.93-144.31)	1287.32(775.3-1972.78)	2957.87(1954.25-4269.27)	24.65(0.4-54.17)	14.32(9.65-20.02)	19.78(12.76-31.08)	-20.63(-35.16--3.32)	580.74(408.19-784.98)	730.91(483.59-1054.5)	-23.02(-36.58--8.17)
Singapore	Male	23.83(10.63-50.53)	55.01(32.02-89.8)	26.79(-13.36-97.34)	1202.97(746.08-1907.66)	2396.39(1659.47-3381.5)	4.2(-17.63-33.46)	14.04(9.22-19.74)	19.63(12.09-30.11)	-11.47(-27.17-7.19)	591.38(408.66-806.13)	829.76(521.52-1220.99)	-9.13(-26.12-9.69)
Slovakia	Both	53.68(27.5-109.12)	104.39(66.89-166.48)	101.46(46.7-187.38)	2532.87(1692.17-3855.99)	4605.5(3328.63-6403.46)	75.07(46.18-111.53)	30.94(18.81-47.35)	22.62(12.44-37.72)	-26.73(-39.76--11.72)	1157.4(721.49-1731.85)	862.11(541.25-1301.74)	-19.98(-32.68--6.1)
Slovakia	Female	18.99(8.55-39.49)	38.81(20.79-69.87)	108.74(55.46-196.33)	1320.18(801.45-2081.23)	2544.27(1641.18-3744.59)	85.47(52.45-125.17)	12.97(7.65-20.61)	11.23(6.09-19.2)	-21.69(-39.11--2.51)	447.96(277.47-673.07)	383.62(238.31-577.54)	-15.06(-30.33-2.78)
Slovakia	Male	34.69(16.51-69.49)	65.59(39.04-108.46)	98.78(44.46-191.96)	1212.68(751.4-1986.58)	2061.24(1402.97-2949.7)	63.74(33.09-105.08)	17.98(10.76-27.79)	11.39(6.17-18.88)	-30.38(-45.13--12.48)	709.45(431.48-1095.71)	478.48(294.28-734.64)	-22.69(-37.81--4.01)
Slovenia	Both	15.1(7.9-28.2)	34.3(22.19-52.95)	127.24(68.95-211.21)	895.67(613.96-1299.81)	1621.08(1206.71-2175.36)	71.35(43.35-105.45)	2.41(1.37-3.96)	2.56(1.37-4.52)	-5.64(-29.8-28.2)	99.76(64.93-144.7)	127.03(82.26-186.86)	31.44(6.22-62.54)
Slovenia	Female	5.86(2.76-11.92)	13(6.82-22.51)	123.31(68.74-204.21)	495.47(308.04-778.21)	900.17(597.11-1314.58)	75.67(45.92-109.4)	1.25(0.69-2.1)	1.35(0.71-2.45)	-3.74(-32.3-35.51)	45.25(28.83-65.99)	58.04(37.79-85.29)	31.69(4.12-66.34)
Slovenia	Male	9.24(4.51-17.4)	21.31(13.16-33.46)	128.99(66.88-233.4)	400.2(260.1-611.71)	720.9(510.1-1037.35)	66.76(34.82-109.36)	1.16(0.66-1.87)	1.21(0.63-2.11)	-7.67(-34.1-26.02)	54.51(35.37-79.74)	68.99(43.99-102.53)	31.26(3.35-66.51)
Solomon Islands	Both	6.36(1.66-16.91)	11.66(3.16-31.05)	-5.32(-13.06-4.11)	110.85(60.17-208.26)	230.95(127.46-427.47)	9.05(3.27-16.29)	13.5(8.77-19.7)	19.55(13.21-26.7)	-17.16(-34.76-5.39)	647.82(421.33-946.8)	949.65(650.45-1293.65)	-6.37(-27.26-21.65)
Solomon Islands	Female	2.9(0.67-8.51)	5.27(1.24-14.9)	-7.85(-17.27-4.62)	53.07(28.51-99.25)	112.83(61.97-206.96)	9.18(1.42-18.91)	7.28(4.4-11.18)	9.79(6.42-13.9)	-28.38(-47.33-6.76)	354.46(219.82-539.42)	478.48(316.36-681.09)	-16.7(-40.74-23.4)

Solomon Islands	Male	3.46(0.92-9.21)	6.39(1.73-16.69)	-2.97(-13.19-9.15)	57.78(29.63-113.75)	118.12(61.98-234.73)	8.98(1.56-18.08)	6.22(4.02-9.67)	9.76(6.64-13.75)	-5.45(-35.81-24.74)	293.36(192.51-453.96)	471.17(322.24-645.29)	5.39(-27.98-40.79)
Somalia	Both	88.88(27.76-225.15)	283.29(86.12-728.88)	15.3(5.01-23.67)	2612.09(1598.5-4364.52)	8624.96(5194.66-14567.84)	17.56(6.59-29.17)	55.36(32.51-86.13)	133.67(78.45-210.46)	53.05(24.45-92.58)	2284.09(1408.52-3513.52)	5651.55(3497.62-8878.52)	61.39(28.83-107.61)
Somalia	Female	28.3(8.11-75.57)	93.6(26.72-245.87)	19.33(7.79-30.61)	1235.56(753.86-2055.19)	4416.18(2611.78-7663.94)	25.27(11.36-40.77)	26.13(14.72-42.39)	63.62(36.13-105.88)	51.91(15.66-102.3)	1107.28(649.83-1772.18)	2648.32(1547.05-4252.18)	56.16(16.26-112.37)
Somalia	Male	60.58(17.87-153.22)	189.69(52.83-485.93)	13.56(1.45-24.76)	1376.54(769-2412.21)	4208.78(2301.34-7477.11)	10.51(-1.32-21.78)	29.23(16.82-46.87)	70.05(40.13-111.58)	53.9(20.68-104.26)	1176.81(707.65-1803.19)	3003.24(1821.95-4708.81)	66.33(28.02-120.3)
South Africa	Both	590.14(410.7-847.79)	1055.87(771.13-1442.53)	22.66(9.16-39.06)	18809.17(15439.29-22955.51)	38091.54(31611.29-46104.71)	35.53(24.65-47.39)	276.06(179.86-407.93)	639.09(402.92-948.81)	30.39(7.09-58.1)	12436.41(8126.89-18051.66)	25662.8(16890.75-37174.08)	43(17-74.13)
South Africa	Female	201.55(132.14-302.63)	347.96(237.81-508.5)	19.85(3.99-41.52)	8384.82(6692.44-10244.97)	18081.43(14614.8-22305.03)	45.75(32.12-62.81)	123.8(81.03-180.86)	260.46(166.43-392.85)	11.23(-16.15-42.28)	5653.36(3664.26-8220.38)	10133.75(6693.21-14873.76)	19.09(-11.49-56.32)
South Africa	Male	388.6(275.18-547.4)	707.91(516.45-984.72)	23.2(9.59-38.86)	10424.36(8416.16-13087.02)	20010.12(16390.34-25262.71)	27.1(16.77-38.95)	152.26(98.06-223.51)	378.63(236.34-564.25)	47.78(22.08-76.85)	6783.05(4426.08-9945.6)	15529.06(10003.3-22780.73)	64.55(34.74-98.55)
South Sudan	Both	64.84(22.55-151.88)	121.26(43.93-282.71)	20.25(11.82-29.88)	2175.06(1308.17-3581.37)	4230.42(2611.77-6879.25)	24.13(12.61-36.38)	33.35(18.87-54.29)	52.33(28.85-86.28)	69.35(23.98-135.64)	1276.32(754.63-1951.8)	2088.91(1189.09-3420.54)	82.68(25.44-168.1)
South Sudan	Female	19.86(6.35-50.6)	39.42(13.62-98.94)	23.14(12-42.36)	993.81(574.6-1639.69)	2241.59(1347.87-3699.04)	35.65(22.46-52.5)	12.86(7.29-21.8)	23.66(12.87-39.46)	98.39(29.9-203.47)	518.77(302.38-850.17)	979.07(549.56-1585.42)	106.2(30.6-233.39)
South Sudan	Male	44.98(15.96-108.19)	81.83(28.99-195.48)	21.14(11.02-31.47)	1181.25(652.8-1963.18)	1988.83(1096.24-3354.48)	13.69(1.01-25.88)	20.48(11.2-34.37)	28.67(14.96-50.58)	51.15(8.68-108.52)	757.55(445.73-1203.39)	1109.84(608.62-1905.16)	66.5(14.79-138.74)
Spain	Both	387.62(244.22-603.8)	805.09(576.98-1117.51)	74.39(29.55-136.2)	18765.08(13174.5-26737.56)	34620.53(26265.84-44833.07)	53.88(24.38-91.87)	174.01(100.14-284.98)	166.38(88.83-296.45)	-27.04(-44.06--2.81)	4831.38(3202.17-7172.82)	4269.05(2852.67-6113.88)	-20.28(-32.42--4.66)
Spain	Female	157.16(86.14-264.81)	333.6(195.66-525.15)	76.19(33.65-131.69)	10543.95(6843.93-15884.97)	19672.93(13607.87-27562.32)	55.86(24.8-95.72)	87.88(49.34-150.87)	87.62(44.83-160.36)	-29.08(-47.84--3.06)	2236(1458.05-3376.77)	2034.95(1360.6-3000.08)	-24.3(-38.42--7.83)
Spain	Male	230.46(137.37-380.88)	471.49(303.17-707.55)	74(24.14-145.44)	8221.14(5420.91-12165.9)	14947.6(10874.22-20434.22)	51.39(19.47-94.68)	86.12(50.52-136.29)	78.75(44.37-135.7)	-25.48(-42.18-0.43)	2595.38(1703.91-3799.86)	2234.1(1443.99-3279.99)	-16.18(-28.9-1.63)
Sri Lanka	Both	173.73(55.93-444.4)	230.25(91.17-563.3)	7.64(-3.42-33.09)	6430.56(4088.29-10549.82)	8832.23(5854.32-14138.38)	9.34(0.99-19.64)	327.57(234.87-432)	519.02(322.46-803.26)	22.68(-0.93-51.3)	14473.31(10681.17-18819.01)	19720.97(12902.54-29112.31)	32.17(3.42-68.7)
Sri Lanka	Female	64.12(17.09-170.7)	86.89(29.01-218.61)	6.11(-5.89-33.1)	3071.22(1894.68-5057.83)	4374.92(2867.32-7023.82)	8.98(-0.99-21.46)	115.25(80.49-155.06)	195.33(120.05-303.35)	5.3(-20.26-41.77)	5227.2(3705-6858.15)	7148.73(4528.07-10817.57)	16.79(-14.37-62.68)
Sri Lanka	Male	109.61(36.31-278.22)	143.36(55.96-352.31)	10.12(-3.38-36.08)	3359.34(2045.99-5777.87)	4457.3(2820.81-7692.52)	10.12(0.86-21.78)	212.32(149.25-289.57)	323.69(198.31-506.23)	37.53(7.63-71.72)	9246.1(6614.1-12286.97)	12572.24(8145.91-18860.87)	45.5(11.21-87.92)
Sudan	Both	284.75(98.58-719.93)	834.42(347.92-1938.55)	46.48(31.02-81.59)	6691.96(3888.68-11742.28)	23990.02(14064.49-41421.32)	79.15(63.78-95.98)	94.58(55.6-147.14)	187.83(99.69-328.44)	148.5(62.41-273.13)	3870.16(2357.1-5928.64)	8181.53(4656.24-13944.55)	204.55(101.01-359.76)
Sudan	Female	120.03(35.93-307.01)	336(115.74-831.23)	40.14(24.26-69.8)	3398.82(1849.16-6026.27)	12310.48(6605.54-21600.47)	80.62(62.3-103.87)	44.11(24.73-73)	87.01(44.52-153.73)	160.35(44.83-285.43)	1916.31(1115.74-3090.75)	4001.16(2114.13-6881.02)	201.59(68.59-361.28)
Sudan	Male	164.72(57.77-396.56)	498.43(211.27-1114.11)	50.99(31.69-92.91)	3293.14(1774.15-5815.11)	11679.55(6433.56-20509.11)	77.62(58.4-98.11)	50.47(29.4-79.59)	100.82(49.33-196.02)	140.12(57.54-319.55)	1953.85(1185.42-3027.98)	4180.37(2263.15-7728.1)	207.64(100.91-423.31)
Suriname	Both	7.92(2.3-22.12)	15.63(5.28-39.31)	29.6(15.62-60.43)	126.04(72.22-231.11)	248.04(154.3-434.34)	32.07(19.59-48.29)	5.16(3.41-7.32)	17.34(10.71-26.35)	107.21(79.28-151.09)	207.61(138.86-288.74)	648.13(415.75-967.08)	130.38(95.66-183.33)
Suriname	Female	2.98(0.68-8.6)	5.85(1.63-16.34)	26.66(12.22-55.19)	55.81(31.82-104.43)	112.06(67.36-196.07)	32.12(18.46-48.4)	2.23(1.46-3.22)	7.91(4.98-11.5)	120.83(85.93-170.84)	89.55(59.4-125.02)	298.69(196.64-436.54)	142.01(97.85-211.04)
Suriname	Male	4.94(1.4-13.11)	9.77(3.29-23.99)	32.39(14.91-68.3)	70.23(37.02-136.96)	135.99(78.03-251.41)	32.57(18.48-52.3)	2.93(1.91-4.16)	9.43(5.7-14.71)	96.93(64.36-146.35)	118.06(78.2-163.31)	349.44(217.68-531.89)	121.66(81.51-184.68)
Sweden	Both	134.05(98.17-178.13)	172.92(128.5-229.28)	6.36(-5.49-20.68)	8869.48(6873.46-11419.6)	11234.34(8909.92-14101.51)	5.89(-4.29-17.23)	20.92(12.61-32.68)	40.3(23.75-65.69)	91.6(66.39-124.23)	805.3(567.16-1125.59)	1188.26(826.87-1674.86)	48.2(32.49-65.73)
Sweden	Female	47.75(30.63-74.34)	58.42(37.85-89.38)	3.03(-10.6-18.42)	3513.24(2597.31-4672.56)	4888.03(3611.13-6562.92)	18.65(5.62-33.96)	8.65(5.26-13.8)	17.22(10.22-29.21)	91.58(62.47-130.36)	324.24(230.83-446.45)	479.3(337.04-674.53)	43.47(27.07-62.41)
Sweden	Male	86.3(58.7-119.6)	114.5(79.28-159.25)	6.96(-6.78-23.96)	5356.24(3785.05-7477.49)	6346.32(4678.53-8816.4)	-3.01(-13.44-9.49)	12.27(7.34-19.17)	23.08(13.33-37.57)	93.16(64.68-126.4)	481.06(333.64-676.98)	708.97(480.89-1015.45)	53.07(35.13-72.56)
Switzerland	Both	95.63(63.39-141.71)	203.45(141.68-282.96)	68.45(41.03-102.85)	5006.5(3662.55-6799.08)	8848.87(6654.07-11387.33)	37.56(20.91-54.88)	11.63(6.63-19.11)	23.12(12.64-40.36)	75.95(43.72-121.37)	473.54(318.14-654.78)	757.22(522.32-1054.54)	51.6(34.79-74.03)
Switzerland	Female	40.83(23.34-69.59)	82.39(48.69-133.96)	60.17(30.98-95.98)	2731.59(1896.98-4023.9)	4860.57(3388.2-6870.65)	40.32(19.64-61.19)	5.23(2.89-8.99)	11.7(6.25-21.18)	87.2(48.04-138.42)	212.22(142.54-299.56)	358.22(242.63-499.01)	50.06(31.02-73.14)
Switzerland	Male	54.81(34.61-84.69)	121.06(77.46-182.96)	74.45(42.73-114.52)	2274.91(1545.98-3308.73)	3988.31(2820.76-5516.95)	34.55(16.18-56.04)	6.4(3.7-10.62)	11.42(6.32-19.34)	67.13(35.66-108.77)	261.32(172.96-367.85)	399(263.32-571.49)	54.25(34.49-78.52)
Syrian Arab Republic	Both	290.68(98.29-743.92)	389.01(216.06-712.92)	23.44(-14.2-138.79)	5792.54(3153.5-11473.32)	11508.35(7723.32-17904.74)	74.21(34.89-133.85)	85.88(53.75-129.77)	101.7(56.8-169.05)	2.74(-18.98-26.9)	3815.17(2460.83-5567.03)	4197.05(2532.69-6751.8)	33.62(0.16-72.77)
Syrian Arab Republic	Female	125.09(36.81-325.18)	153.45(71.24-313.43)	8.03(-22.12-96.92)	2787.04(1514.93-5299.01)	6031.49(3787.13-9634.57)	80.8(36.26-138.87)	39.81(24.14-61.19)	46.17(25.8-75.39)	2.93(-23.2-38.06)	1857.28(1136.74-2786.75)	1975.58(1186.06-3118.05)	23.58(-14.15-73.31)
Syrian Arab Republic	Male	165.6(57.21-420.16)	235.56(130.66-437.24)	37.76(-7-177.78)	3005.5(1529.16-6046.59)	5476.85(3528.89-9093.66)	68.1(33.19-131.97)	46.07(27.84-71.38)	55.52(30.37-95.59)	2.87(-20.78-32.01)	1957.89(1213.23-2883.5)	2221.47(1335-3628.76)	43.31(9.31-89.96)
Taiwan (Province of China)	Both	156.4(70.54-310.36)	324.53(205.85-502.24)	73.84(21.91-183.94)	9461.34(7063.96-13162.92)	24987.33(19631.06-31561.29)	124.47(85.47-171.23)	274.29(191.63-379.05)	351.32(207.22-547.84)	-28.65(-39.16--17.47)	11719.94(8408.24-15291.79)	16077.17(10070.03-23100.13)	2.28(-14.05-18.42)
Taiwan (Province of China)	Female	49.34(17.05-116.43)	125.04(63.04-223.54)	105.84(35.64-280.43)	4737.48(3347.03-6621.4)	13806.47(10349.45-18542.56)	137.93(93.97-190.63)	128.5(88.06-177.12)	153.44(91.09-246.17)	-38.33(-48.54--25.61)	5442.77(3931.15-7156.36)	6963.44(4385.47-10078.93)	-10.22(-25.75-8.01)
Taiwan (Province of China)	Male	107.06(49.37-214.75)	199.49(117.42-311.68)	60.99(15.96-150.71)	4723.86(3342.78-6857.29)	11180.86(8596.05-14397.26)	109.51(70.01-157.27)	145.78(101.18-202.53)	197.88(116.12-317.07)	-20.46(-32.3--7.4)	6277.16(4468.78-8273.64)	9113.73(5661.98-13341.32)	13.47(-5.28-33.19)
Tajikistan	Both	84.1(26.93-214.79)	192.91(67.7-471.39)	32.25(17.74-71.14)	2654.92(1652.36-4412.51)	6719.35(4120.64-11014.74)	44.96(31.09-60.53)	4.49(2.79-6.79)	19.21(11.53-30.67)	260.69(204.89-329.53)	281(182.26-409.42)	1071.61(674.22-1676.8)	274.04(210.61-355.12)
Tajikistan	Female	37.67(9.65-105.12)	79.74(22.84-213.41)	22.4(8.89-54.14)	1329.74(766.59-2207.04)	3425.16(1946.76-5674.7)	49.3(31.84-72)	2.69(1.61-4.19)	9.4(5.44-15.42)	211.15(147.68-288.15)	164.51(103.29-244.69)	527.61(324.3-822.73)	222.4(156.98-305.37)
Tajikistan	Male	46.43(15.34-112.77)	113.17(41.13-267.7)	40.12(22.27-79.52)	1325.19(767.83-2249.4)	3294.19(1925.95-5481.58)	40.66(26.15-57.84)	1.8(1.11-2.72)	9.82(5.79-15.98)	338.98(258.27-442.41)	116.49(76.57-167.83)	544(336.1-840.12)	348.75(262.9-472.47)
Thailand	Both	818.67(242.93-2069.19)	823.17(416.69-1611.12)	-15.1(-35.48-59.9)	28699.26(17550.78-47418.43)	38161.25(27251.95-53129.24)	8.13(-11.62-34.71)	1480.4(1065.35-1967.1	2164.58(1296.58-3387.2	-11.01(-33.25-15.01)	69983.14(50756.61-92478.59)	81319.13(50790.12-124640.34)	-4.02(-30.85-27.73)
Thailand	Female	277.33(70.44-796.35)	290.74(120-666.64)	-11.48(-31.84-64.49)	13598.44(7923.86-22879.53)	19176.27(12990.58-28384.43)	12.68(-7.18-39.73)	496.36(342.08-674.43)	991.53(583.06-1567.69)	15.65(-11.05-48.61)	22279.92(15899.01-30042.57)	35213.87(21496.71-54445.74)	26.47(-9.56-71.78)

Thailand	Male	541.35(154.21-1309.23)	532.43(260.98-1031.5)	-16.93(-38-59.06)	15100.81(8844.2-26196.29)	18984.98(13045.39-27552.32)	4.14(-17.27-34.1)	984.04(695.05-1345.15	1173.05(713.09-1831.73)	-24.68(-45.76-3.16)	47703.22(33915.42-65665.24)	46105.27(29846.4-68479.72)	-18.14(-42.43-14.48)
Timor-Leste	Both	10.39(3.21-24.31)	18.84(6.04-43.87)	21.92(9.55-48.42)	294(179.85-479.24)	619.54(370.33-1045.21)	26(15.58-36.55)	14.66(9.04-21.84)	25.08(13.83-38.78)	72.17(5.9-141.18)	673.34(423.17-981.59)	1011.86(513.49-1495.58)	124.11(25.19-235.45)
Timor-Leste	Female	4.43(1.23-11.07)	7.48(2.24-18.55)	13.13(-0.15-38.58)	144.63(83.98-237.59)	315.75(176.95-531.43)	27.33(15.37-40.38)	8.08(5-12.07)	12.19(6.63-18.32)	64.58(-4.83-140.62)	369.52(229.88-541.87)	490.86(238.2-720.17)	106.06(-0.4-218.3)
Timor-Leste	Male	5.97(1.88-14.17)	11.37(3.59-27.15)	28.54(13.16-58.27)	149.37(85.17-264.6)	303.79(168.51-557.25)	24.57(11.22-38.29)	6.58(3.8-11.19)	12.89(6.57-21.36)	84.15(4.86-170.18)	303.82(179.76-484.25)	521(253.35-835.02)	147.16(26.24-284.59)
Togo	Both	74.75(25.6-176.66)	176.34(71-387.26)	13.39(2.84-34.97)	2121.93(1299.71-3423.31)	5903.92(3757.31-9101.37)	29.89(18.78-43.54)	28.21(17.43-44.67)	76.56(46.27-115.12)	98.45(49.92-154.44)	1214.38(771.94-1861.57)	3290.17(2051.83-4845.57)	134(69.87-205.39)
Togo	Female	28.52(9.34-71.25)	65.78(24.9-153.94)	11.55(-1.88-35.74)	1084.43(659.58-1741.41)	3120.66(1945.96-4982.4)	34.32(19.64-52.31)	15.17(8.89-25.71)	35.9(20.8-55.02)	74.98(27.02-135.11)	664.89(399.38-1098.83)	1547.63(906.74-2352.12)	102.79(41.01-182.24)
Togo	Male	46.23(14.72-109.62)	110.55(41.05-250.92)	14.2(2.21-36.23)	1037.49(581.63-1779.76)	2783.26(1619.07-4675.51)	25.26(13.78-40.02)	13.04(8.11-20.09)	40.66(24.23-62.43)	125.81(66.44-197.78)	549.5(352.53-819.96)	1742.55(1078.93-2582.72)	171.81(95.67-267.72)
Tokelau	Both	0.02(0.01-0.05)	0.02(0.01-0.05)	9.08(0.53-24.09)	0.49(0.29-0.83)	0.5(0.3-0.84)	20.89(13.89-29.7)	0.04(0.03-0.06)	0.05(0.03-0.08)	73.05(34.92-115.54)	1.68(1.06-2.53)	2.06(1.25-3.28)	84.02(34.31-138.98)
Tokelau	Female	0.01(0-0.02)	0.01(0-0.02)	9.62(-0.77-25.23)	0.23(0.14-0.39)	0.23(0.13-0.37)	21.31(13.62-31.24)	0.03(0.02-0.04)	0.03(0.02-0.05)	56.23(18.68-101.97)	1.11(0.68-1.72)	1.2(0.7-1.97)	66.56(18.36-123.33)
Tokelau	Male	0.01(0-0.03)	0.01(0-0.03)	7.21(-4.48-26.26)	0.26(0.14-0.47)	0.27(0.16-0.47)	19.74(10.73-31.37)	0.01(0.01-0.02)	0.02(0.01-0.03)	108.42(40.4-174.2)	0.56(0.35-0.86)	0.86(0.47-1.38)	120.03(43.27-199.94)
Tonga	Both	1.25(0.36-3.54)	1.61(0.49-4.51)	14.44(6.16-27.65)	34.07(19.69-66.65)	42.56(24.81-81.18)	18.84(11.15-27.51)	1.65(1.05-2.55)	3.06(1.85-4.91)	43.88(16.71-72.35)	68.47(45.08-104.37)	124.08(77.39-195.03)	73.92(37.86-117.1)
Tonga	Female	0.53(0.13-1.59)	0.63(0.16-1.85)	5.36(-3.2-18)	16.39(9.17-30.34)	20.31(11.9-36.78)	16.03(7.04-27.19)	0.93(0.58-1.48)	1.32(0.78-2.14)	7.08(-13.3-37.65)	38.72(24.84-60.43)	53.41(32.36-87.83)	32.79(1.7-75.65)
Tonga	Male	0.72(0.21-1.96)	0.98(0.29-2.67)	21.18(9.49-36.96)	17.69(9.67-34.64)	22.25(12.21-44.66)	21.69(11.38-32.65)	0.72(0.44-1.16)	1.74(1-2.83)	91.43(46.61-139.88)	29.75(18.78-47.03)	70.67(42.68-113.55)	127.46(72.55-187.14)
Trinidad and Tobago	Both	13.23(4.36-31.26)	19.36(8.95-40.91)	24.03(1.25-93.59)	362.19(202.92-630.5)	587.28(388.63-923.38)	39.36(19.1-69.94)	11.37(7.64-15.96)	35.67(19.55-59.93)	120.75(83.75-160.6)	452.9(318.2-614.91)	1274.12(734.42-2104.12)	128.52(80.59-180.32)
Trinidad and Tobago	Female	4.74(1.32-11.99)	6.55(2.52-15.07)	18.01(-3.86-85.76)	157.44(91-273.17)	264.14(171.06-405.73)	44.52(21.96-78.58)	4.53(3.02-6.41)	14.93(8.13-24.78)	136.63(92.26-185.41)	185.65(127.78-254.9)	538.67(308.11-875.83)	139.78(83.76-202.92)
Trinidad and Tobago	Male	8.49(2.7-19.94)	12.81(5.86-26.58)	26.87(2.62-96.72)	204.75(106.9-377.36)	323.15(197.81-526.43)	35.33(15.2-69.38)	6.84(4.57-9.62)	20.74(11.36-34.91)	109.93(74.66-146.85)	267.25(184.47-368.14)	735.45(429.04-1200.84)	120.44(75.95-168.3)
Tunisia	Both	108.98(44.67-247.43)	270.62(156.74-463.15)	81.49(31.28-207.75)	3189.94(1878.47-5480.54)	8092.65(5602.5-11771.22)	82.81(50.27-130.46)	31.48(19.3-48.26)	69.38(38.63-117.28)	39.95(10.63-71.49)	1275.51(826.88-1918.73)	2777.73(1730.5-4372.06)	102.53(61.95-152.78)
Tunisia	Female	48.33(16.12-114.75)	107.55(51.08-208.25)	59.52(17-172.74)	1671.71(928.59-2838.01)	4287.3(2758.14-6492.39)	81.79(46.9-130.62)	14.6(8.97-22.61)	31.86(17.64-52.81)	33.33(-3.33-70.58)	627.67(401.99-924.47)	1304.49(795.3-2030.4)	88.04(40.94-146.12)
Tunisia	Male	60.64(23.96-129.98)	163.07(91.46-274.36)	100.51(44.22-239.27)	1518.23(837.05-2665.95)	3805.35(2488.41-5780.39)	83.69(50.34-135.67)	16.88(9.87-27.19)	37.52(20.2-64.58)	45.29(8.37-90.62)	647.83(398.14-983.62)	1473.24(873.92-2344.95)	116.76(68.32-178.72)
Turkey	Both	814.19(325.19-1852.26)	2304.08(1422.84-3703.86)	107.22(33.48-276.77)	26687.94(16748.68-44201.24)	77584.12(55732.98-109759.57)	112.5(73.84-163.34)	479.91(291.28-726.85)	548.81(340.76-831.48)	-1.84(-26.39-21.91)	19292.2(12613.35-27903.46)	22019.21(14499.18-31567.03)	36.81(4.46-72.15)
Turkey	Female	311.19(97.86-778.29)	804.78(412.42-1503.29)	88.25(24.26-253.74)	13002.29(7852.67-21890.21)	39388.96(26064.55-57858.31)	120.48(80.96-171.74)	189.42(114.52-295.93)	224.31(139.14-340.65)	-4.21(-35.62-26.61)	7841.85(5030.19-11923.98)	8846.76(5819.8-12875.39)	27.93(-9.81-67.81)
Turkey	Male	503.01(209.41-1154.07)	1499.3(896.3-2468.81)	119.62(40.68-290.24)	13685.65(7929.66-23400.1)	38195.15(25308.52-56429.78)	104.91(65.23-161.5)	290.49(173.54-455.46)	324.5(199.23-497.59)	0.6(-24.45-33.07)	11450.34(7189.67-16981.97)	13172.45(8463.2-18916.37)	44.96(11.22-91.49)
Turkmenistan	Both	83.86(27.71-211.65)	150.75(59.24-358.14)	32.42(17.39-69.29)	2297.18(1417.15-3750.32)	4430.73(2889.52-6896.23)	41.77(27.04-61.2)	13.56(8.5-20.38)	35.84(21.3-58.77)	115.49(82.42-153.8)	700.22(454.28-1030.25)	1763.51(1104.52-2786.01)	166.17(120.82-222.46)
Turkmenistan	Female	34.98(9.33-94.8)	55.88(17.28-142.44)	20.42(7.53-52.87)	1100.7(634.43-1885.08)	1992.51(1214.32-3250.31)	37.19(21.47-58.58)	6.73(4.19-10.02)	12.95(7.62-21.24)	65.74(34.83-100.91)	349.21(223.36-518.48)	641.5(399.93-1024.89)	102.75(62.15-151.62)
Turkmenistan	Male	48.88(17.21-119.8)	94.87(38.12-222.59)	39.68(21.78-77.92)	1196.48(710.12-2040.39)	2438.22(1511.53-3952.07)	45.17(27.74-68.73)	6.83(4.21-10.25)	22.89(13.63-37.07)	161.14(121.49-206.27)	351.01(224.1-519.48)	1122.01(695.17-1743.45)	226.25(173.25-291.74)
Tuvalu	Both	0.11(0.04-0.27)	0.15(0.05-0.35)	-0.2(-9.53-12.68)	2.78(1.71-4.62)	4.26(2.57-7)	21.65(15.01-28.37)	0.33(0.21-0.5)	0.54(0.32-0.85)	65.17(33.45-100.14)	13.87(9.18-20.34)	22.64(14.19-34.41)	101.44(55.48-152.5)
Tuvalu	Female	0.05(0.01-0.12)	0.06(0.02-0.15)	2.39(-7.97-15.77)	1.38(0.84-2.3)	1.95(1.16-3.3)	21.48(12.93-30.53)	0.19(0.12-0.3)	0.24(0.13-0.42)	30.1(-2.1-64.54)	8.14(5.22-12.38)	9.66(5.54-16.63)	55.63(13.68-106.78)
Tuvalu	Male	0.07(0.02-0.16)	0.09(0.03-0.22)	-4.56(-16.18-11.66)	1.4(0.82-2.45)	2.31(1.32-4.09)	20.71(13.2-30.27)	0.14(0.09-0.2)	0.3(0.17-0.47)	113.38(55.21-176.44)	5.72(3.72-8.54)	12.99(7.77-19.87)	164.55(92.48-250)
Uganda	Both	176.82(57.57-460.73)	454.14(148.71-1140.84)	18.87(9.53-28.83)	5430.62(3350.94-9036.17)	16040.33(9765.85-26728.52)	26.41(16.38-36.9)	83.8(50.5-133.72)	198.56(121.6-300.94)	183.78(117.66-279.22)	3186.65(1961.34-4859.72)	8128.5(5085.47-12084.99)	212.31(129.2-323.1)
Uganda	Female	54.49(15.38-154.31)	134.68(41-360.53)	15.02(3.04-33.16)	2704.84(1617.07-4538.35)	8587.16(5178.75-14738.74)	35.2(23.22-51.02)	30.33(17.06-52.65)	81.2(47.44-127.4)	234.41(136.34-383.63)	1189.51(688.95-2031.93)	3290.69(1980.61-5095.55)	251.59(139.51-426.69)
Uganda	Male	122.33(38.6-311.52)	319.46(98.14-824.9)	20.17(8.74-32.78)	2725.78(1549.31-4797.49)	7453.18(4058.31-13555.83)	17.63(4.93-31.07)	53.46(31.05-85.28)	117.36(70.58-179.03)	153.99(86.76-246.62)	1997.13(1232.45-3040.19)	4837.81(2969.03-7208.3)	187.22(108.17-297.52)
Ukraine	Both	500.67(275.64-938.8)	713.64(439.29-1175.98)	69.1(41.86-109.11)	33734.51(24722.07-45178.03)	50152.6(37120.43-66857.06)	76.65(57.78-99.1)	85.12(53.43-126.81)	177.79(114.85-260.82)	85.15(53.12-125.95)	2928.23(2071.94-3988.47)	6815.13(4687.95-9677.39)	132.53(99.51-169.96)
Ukraine	Female	212.47(102.44-404.72)	283.79(148.14-491.4)	57(30.59-95.85)	18487.57(12543.93-26972.68)	29799.81(19856.47-43598.61)	90.68(67.52-118.33)	47.39(28.83-72.76)	82.35(50.28-125.55)	66.16(37.22-102.73)	1457.19(1026.97-2025.41)	2870.27(1921.74-4159.2)	114.85(79.79-153.38)
Ukraine	Male	288.2(149.52-557.96)	429.85(248.88-733.37)	78.85(48.17-123.99)	15246.94(10012.54-22564.52)	20352.79(13873.02-29266.63)	59.47(40.16-83.19)	37.73(24.6-54.72)	95.43(60.95-143.79)	107.14(70.8-153.1)	1471.04(1032-2007.38)	3944.86(2633.63-5864.45)	148.47(112.73-192.65)
United Arab Emirates	Both	32.94(12.39-74.53)	349.74(192.57-576.48)	123.73(38.48-302.25)	878.15(539.36-1411.66)	9187.38(6556.68-12907.83)	107.83(58.54-169.2)	20.32(12.11-31.39)	186.03(93.84-353.22)	80.85(26.77-164.38)	954.57(589.57-1460.48)	8896.79(4762.35-16391.95)	89.18(30.44-183.87)
United Arab Emirates	Female	10.25(2.7-27.43)	60.17(25.86-118.56)	56.89(7.13-211.34)	290.04(158.26-533.96)	2485.19(1621.68-3777.92)	119.78(69.68-190.89)	4.67(2.74-7.43)	36.47(18.59-61.66)	166.65(51.24-284.61)	218.97(130.4-347.79)	1761.08(950.55-2880.28)	134.02(28.3-261.59)
United Arab Emirates	Male	22.69(8.67-49.93)	289.57(158.09-479.86)	138.26(49.86-291.9)	588.11(360.78-949.42)	6702.19(4622.58-9479.34)	101.43(56-159.2)	15.65(8.87-24.9)	149.56(71.83-310.21)	60.01(8.75-150.62)	735.6(429.47-1157.68)	7135.71(3607.07-14117.62)	71.51(13.49-175.66)
United Kingdom	Both	679.97(594.51-773.77)	914.49(797.34-1040.5)	17.16(7.21-27.32)	30759.91(27561.82-34353.96)	35732.9(31652.79-39955.75)	-0.55(-9.57-8.18)	124.55(72.64-202.77)	132.51(77.78-210.6)	9.11(0.01-21.85)	4558.5(3081.68-6479.17)	5008.73(3400.15-6977.23)	11.25(5.11-17.63)
United Kingdom	Female	265(228.92-303.9)	337.79(287.59-395.94)	13.59(2.09-24.77)	16141.18(14260.07-18095.6)	18839.72(16402.78-21249.87)	2(-8.44-13.09)	64.06(36.17-106.04)	66.88(38.81-108.49)	9.25(-0.48-21.91)	2154.83(1476.97-3121.78)	2379.71(1631.15-3353.78)	11.32(4.15-18.79)
United Kingdom	Male	414.97(361.11-477.34)	576.69(500.69-662.43)	18.09(7.71-28.56)	14618.74(13055.97-16580.83)	16893.17(14855.06-19068.34)	-3.26(-12.43-4.72)	60.49(35.26-96.1)	65.63(38.7-103.62)	9(-1.09-23.48)	2403.67(1618.44-3378.73)	2629.02(1769.39-3691.02)	11.24(5.13-18.35)

United Republic of Tanzania	Both	346.53(123.5-799.04)	779.96(279.31-1743.76)	11.56(2.13-22.09)	11999.12(7591.54-17986.65)	30641.62(19143.92-47023.77)	19.63(9.45-31.74)	118.8(72.16-183.82)	249.17(157.56-369.61)	100.03(47.71-169.25)	4653.5(2959.8-6834.19)	10041.24(6774.69-14031.63)	128.18(65.44-210.54)
United Republic of Tanzania	Female	120.58(38.79-291.21)	282.48(94.94-669.59)	16.96(4.29-31.27)	6162.63(3621.16-9508.21)	16809.53(10014.66-26260.49)	26.9(13.46-42.38)	55.3(32.65-87.96)	123.91(77.11-187.96)	116.06(51.12-201.11)	2206.63(1368.86-3415.89)	4970.96(3259.61-7100.33)	139.84(69.25-236.62)
United Republic of Tanzania	Male	225.95(75.82-511.37)	497.48(164.81-1124.58)	8.3(-2.26-19.8)	5836.49(3318.22-9451.73)	13832.09(7907.87-22580.35)	11.84(0.62-23.48)	63.5(37.55-100.53)	125.27(77.15-190.87)	86.29(35.59-160.02)	2446.87(1531.8-3621.63)	5070.28(3379.3-7229.99)	117.75(55.62-205.06)
United States of America	Both	5639.54(4865.74-6539.99)	8064.02(7150.6-9094.98)	13.27(5.51-20.61)	261031.21(233219.87-292011.1	348669.04(314549.55-384199.3	2.94(-1-7.47)	603.3(380.01-902.29)	2353.39(1472.32-3651.6)	182.32(146.57-222.27)	29872.28(20418.13-41109.3)	82991.34(55516.69-118468.54)	109.1(78.69-143.17)
United States of America	Female	2083.87(1754.24-2466.37)	2922.84(2509.38-3380.24)	11.73(4.36-20.25)	138202.2(121118.64-156322.99)	181381.67(160780.54-203179.6	1.42(-2.29-5.75)	305.16(188.22-478.7)	1059.35(661.07-1667.53)	150.58(119.79-189.39)	13758.06(9576.05-19049.49)	36350.48(24353.58-51617.01)	94.55(69.98-121.74)
United States of America	Male	3555.67(3026.39-4195.83)	5141.17(4498.8-5920.4)	13.8(5.84-21.81)	122829.01(108681.7-138038.68)	167287.37(149444.4-186405.56)	4.67(-0.32-10.63)	298.14(187.69-452.31)	1294.03(790.89-1954.42)	214.89(164.06-276.82)	16114.22(10892.04-22813.02)	46640.87(30343.37-67046.05)	122.3(84.28-169.53)
United States Virgin Islands	Both	1.52(0.72-3.29)	1.88(1.09-3.47)	17.54(-2.89-61.65)	45.17(30.56-67.97)	56.09(42.12-77.93)	25.05(10.53-46.91)	1.27(0.81-1.85)	2.64(1.57-4.15)	4.91(-19.35-33.4)	51.55(34.25-73.03)	85.91(53.8-132.22)	25.23(-2.87-60.8)
United States Virgin Islands	Female	0.47(0.18-1.13)	0.64(0.3-1.29)	27.9(0.78-87.43)	19.45(13.02-28.9)	26.29(18.67-37.39)	33.61(16.89-58.16)	0.55(0.34-0.84)	1.02(0.6-1.63)	2.48(-23.66-33.4)	21.36(13.7-31.41)	32.47(19.82-50.5)	20.13(-10.94-57.35)
United States Virgin Islands	Male	1.06(0.48-2.26)	1.25(0.68-2.35)	14.23(-5.15-55.34)	25.71(16.37-41.32)	29.8(20.73-43.97)	19.1(4.16-42)	0.72(0.46-1.06)	1.62(0.95-2.59)	6.19(-21.01-40.6)	30.19(19.62-42.75)	53.44(32.52-82.96)	28(-4.12-71.67)
Uruguay	Both	98.43(66.57-140.82)	78.89(52.26-121.68)	-26.45(-39.27--13.92)	5280.74(3887.92-7051.3)	4508.84(3243.18-6213.27)	-22.29(-33.08--10.86)	16.12(10.5-23.78)	26.52(17.45-39.65)	43.95(22.24-67.71)	633.9(458.64-838.49)	913.82(647.99-1255.04)	44.41(25.14-64.47)
Uruguay	Female	34.31(19.18-55.15)	33.51(18.63-55.52)	-11.8(-27.66-4.46)	2773.02(1826.02-4030.24)	2721.41(1807.06-3958.77)	-11.86(-24.89-3.04)	6.08(4.07-9.05)	10.96(7.19-16.56)	42.71(19.06-68.66)	243.56(177.55-322.99)	379.79(273.47-519.45)	46.92(27.79-67.68)
Uruguay	Male	64.12(40.63-97.37)	45.38(27.42-74.27)	-33.89(-46.21--20.57)	2507.71(1748.45-3649.36)	1787.43(1225.21-2663.44)	-34.15(-44.71--22.85)	10.04(6.51-14.88)	15.56(10.01-23.14)	48.5(25.61-74.4)	390.33(278.62-530.95)	534.03(369.32-734.14)	44.74(23.82-66.78)
Uzbekistan	Both	554.07(187.16-1295.57)	1072.77(415.04-2335.15)	18.96(7.8-37.17)	14208.17(9046.14-22106.5)	31767.31(20352.12-49525.24)	40.11(26.69-55.28)	75.81(46.44-119.09)	215.33(130.36-328.1)	82.52(39.73-119.65)	3896.31(2509.44-5837.85)	11052.66(7004.25-16257.72)	117.72(69.31-163.3)
Uzbekistan	Female	246.51(73.72-595.22)	435.97(140.65-1014.04)	8.68(-1.35-23.19)	7213.93(4424.67-11847.19)	15960.01(9876.36-26093.7)	39.16(22.54-58.45)	35.5(20.42-60.43)	92.24(56.19-141.25)	74.15(14.88-124.07)	1855.35(1139.52-3011.25)	4809.13(3066.16-7175.99)	100.01(37.34-155.8)
Uzbekistan	Male	307.56(107.54-709.55)	636.81(246.45-1404.65)	27.18(14.16-48.93)	6994.24(4019.26-11583.78)	15807.3(9424.15-25730.27)	41.07(26.03-59)	40.3(24.81-61)	123.08(73.69-191.97)	89.39(52.54-126.47)	2040.97(1305.58-3043.1)	6243.53(3895.4-9298.85)	133.77(90.36-176.07)
Vanuatu	Both	1.86(0.52-5.02)	3.99(1.18-10.95)	20.23(9.96-32.7)	44.16(25-80.01)	101.45(58.35-186.62)	19.46(12.7-27.64)	2.27(1.34-3.53)	9.53(5.8-14.59)	107.54(55.5-188.22)	103.32(61.42-159.58)	419.85(257.92-622.82)	133.16(65.77-231.13)
Vanuatu	Female	0.82(0.19-2.24)	1.72(0.42-4.69)	18.22(6.57-32.69)	21.79(11.88-38.39)	50.66(28.51-89.08)	18.41(10.36-28.06)	1.12(0.62-1.92)	4.6(2.55-7.61)	94.99(33.14-201.09)	50.8(29.34-83.81)	201.95(110.46-331.45)	123.42(43.21-263.19)
Vanuatu	Male	1.04(0.3-2.73)	2.26(0.67-5.76)	21.81(8.07-38.87)	22.37(12.01-41.86)	50.78(27.77-98.36)	20.47(11.54-31.11)	1.15(0.66-1.85)	4.93(3.06-7.4)	117.38(55.55-194.48)	52.52(30.88-81.54)	217.9(136.93-320.87)	141.74(68.95-234.5)
Venezuela (Bolivarian Republic of)	Both	268.81(93.04-658.63)	459.49(204.54-975.53)	17.85(-0.06-62.78)	6839.09(3870.04-11888.03)	12390.65(7720.97-20025.39)	21.6(8.22-43.5)	116.11(77.76-162.05)	640.04(361.03-1057.86)	161.03(116.77-211.19)	4786.39(3331.59-6413.12)	22128.88(12964.14-35980.83)	198.69(138.22-274.28)
Venezuela (Bolivarian Republic of)	Female	84.97(24.79-214.01)	145.42(53.28-336.16)	16.43(-2.68-65.65)	3147.48(1773.67-5419.95)	5706.63(3538.2-9208.11)	19.62(4.7-41)	52.86(35.83-72.95)	274.95(154.68-456.27)	150.04(98.92-204.78)	2221.44(1557.96-3001.05)	9630.38(5743.32-15640.86)	183.67(115.85-263.56)
Venezuela (Bolivarian Republic of)	Male	183.84(64.32-436.21)	314.07(136.19-656.87)	19.41(0.04-68.08)	3691.61(1915.92-6960.16)	6684.02(3971.48-11456.68)	23.72(8.09-49.51)	63.25(41.4-89.67)	365.09(202.82-608.25)	170.34(124.77-225.4)	2564.96(1756.31-3512.68)	12498.5(7184.41-20537.32)	211.77(152.82-287.32)
Viet Nam	Both	603.97(183.78-1498.27)	879.55(373.22-1974.59)	8.72(-7.08-58.44)	25089.79(14973.55-40845.9)	46836.14(31553.18-69329.97)	34.27(17.39-58.73)	1132.12(740.23-1664.0	2033.21(1334.71-2978.8	23.05(-8.04-55.77)	48535.83(31782.84-69264.1)	82949.88(55818.62-118690.1)	48.4(10.67-94.2)
Viet Nam	Female	280.56(76.16-724.84)	373.23(135.38-861.5)	2.3(-13.98-52.48)	13335.24(7771.09-22083.79)	25203.6(15909.87-38731.94)	37.87(18.07-64.31)	715.15(457.55-1058.64	1073.82(693.26-1575.27)	9.96(-16.66-44.26)	30438.53(19650.26-45076.17)	43127.06(29089.45-61077.55)	28.8(-5.6-74.87)
Viet Nam	Male	323.41(93.45-813.31)	506.32(207.04-1119.85)	13.42(-4.76-64.88)	11754.55(6645.6-21004.12)	21632.54(13889.49-34169.33)	30.43(13.72-55.11)	416.98(267.27-633.72)	959.39(600.4-1406.77)	49.46(-3.33-110.74)	18097.3(11806.92-26653.96)	39822.82(25936.58-57635.49)	84.41(23.33-161.87)
Yemen	Both	221.93(77.66-513.84)	714.77(316.12-1523.42)	47.57(27.28-92.41)	5276.9(3177.33-9249.24)	22204.91(13957.71-37207.98)	84.89(66.84-107.64)	51.02(27.72-86.47)	124.39(69.02-204.47)	105.17(52.71-182.81)	2183.52(1263.63-3490.43)	5822.45(3560.28-8834.35)	156(86.59-258.15)
Yemen	Female	90.94(27.72-241.35)	293.03(102.17-720.04)	47.08(22.56-95.95)	2645.32(1518.73-4607.39)	11762.28(6768.94-20202.14)	92.65(70.35-122.73)	22.09(11.49-39.83)	56.15(30.75-97.01)	129.75(54.18-237.54)	986.31(541.94-1712.59)	2766.59(1631.99-4522.68)	174.19(87.05-310.13)
Yemen	Male	131(45.78-301.52)	421.74(189.23-866.6)	48.1(24.74-97.66)	2631.58(1463.8-4711.8)	10442.63(6007.38-17690.4)	76.81(58.04-100.13)	28.93(15.75-49.34)	68.25(38.42-112.43)	87.03(33.11-165.32)	1197.21(693.98-1956.33)	3055.86(1864.31-4776.22)	141.11(70.41-244.4)
Zambia	Both	113.72(38.53-273.49)	285.25(105.94-665.22)	24.48(14.54-40.68)	2915.66(1746.52-4629.24)	9441.61(5881.72-14608.77)	43.13(31.69-58.42)	55.86(34.88-81.93)	134.26(81.61-200.57)	121.7(69.01-189.85)	2306.02(1526.61-3352.23)	5792.27(3677.84-8469.27)	159.43(93.31-249.82)
Zambia	Female	40.92(11.91-109.1)	97.02(31.84-239.22)	20.41(6.81-45.55)	1432.26(824.32-2316.19)	5055.39(2956.73-8089.83)	56.57(37.68-79.18)	30.03(18.88-45.56)	62.11(36.46-93.9)	106.93(48.65-186.78)	1298.09(822.5-1964.71)	2691.96(1658.12-3999.37)	128.4(57.78-229.87)
Zambia	Male	72.8(24.2-174.85)	188.23(65.88-443.92)	25.2(13.81-41.05)	1483.4(827.81-2512.49)	4386.21(2452.84-7388.55)	30.2(18.89-43.4)	25.82(15.41-39.89)	72.16(43.49-108.63)	141.17(69.03-239.65)	1007.93(628.46-1478.82)	3100.32(1945.35-4635.12)	200.37(114.46-323.46)
Zimbabwe	Both	139.31(53.85-333.38)	222.54(88.59-533.47)	8.82(-0.18-19.5)	4892.17(2812.55-8947.22)	9039.79(5216.99-16683.89)	30.63(20.37-42.3)	42.84(24.02-80.02)	141.55(76.85-235.98)	104.74(56.34-173.79)	1712.26(1008.3-3061.61)	5737.17(3207.28-9677.58)	137.48(76.93-224.57)
Zimbabwe	Female	45.76(13.27-118.01)	78.62(23.25-202.63)	15.22(3.48-30.43)	2217.97(1257.26-3920.85)	4303.31(2479.62-7637.91)	33.25(20.78-47.87)	16.37(8.29-36.89)	57.87(28.55-124.59)	107.55(54-184.63)	676.96(359.79-1476.8)	2352.76(1207.08-4840.84)	140.04(70.99-234.09)
Zimbabwe	Male	93.55(35.68-218.34)	143.92(57.31-336.56)	6.56(-3.01-18.25)	2674.2(1454.39-4986.59)	4736.48(2545.75-9226.65)	29.24(16.8-43.73)	26.47(14.59-47.74)	83.67(45.07-135.55)	105.17(42.35-197.2)	1035.3(606.29-1819.23)	3384.41(1843.7-5486.52)	137.18(63.23-243.22)

Abbreviation: DALY, disability adjusted life-year; UI, uncertainty interval.

Table S4. The age-standardized rates and their EAPCs of type 1 diabetes related CKD in 204 countries and territories.

Location	Sex	ASIR (95% UI)		EAPC (95% CI)	ASPR (95% UI)		EAPC (95% CI)	ASDR (95% UI)		EAPC (95% CI)	Age-standardized DALYs Rate (95% UI)		EAPC (95% CI)
		1990	2019		1990	2019		1990	2019		1990	2019	
Afghanistan	Both	1.27(0.58-2.49)	2.01(0.93-3.92)	1.9(1.65-2.15)	34.56(22.43-51.84)	51.38(32.51-79.65)	1.74(1.51-1.97)	2.3(1.35-3.68)	1.88(1.11-3.07)	-0.59(-0.64--0.53)	86.79(51.28-138.44)	70.89(43.06-116.23)	-0.59(-0.65--0.53)
Afghanistan	Female	1.14(0.4-2.59)	1.71(0.59-3.9)	1.61(1.43-1.79)	33.69(20.17-53.53)	50(28.63-82.39)	1.64(1.42-1.86)	2.34(1.37-3.9)	1.98(1.11-3.53)	-0.43(-0.49--0.36)	94.2(53.44-158.46)	78.46(44.99-139.43)	-0.49(-0.56--0.43)
Afghanistan	Male	1.42(0.67-2.69)	2.32(1.12-4.37)	2.09(1.8-2.39)	35.66(22.3-54.59)	53.05(32.62-83.16)	1.82(1.59-2.06)	2.18(1.18-3.63)	1.81(1.01-2.95)	-0.58(-0.67--0.5)	75.84(42.81-120.75)	64.34(36.16-104.18)	-0.51(-0.59--0.42)
Albania	Both	1.42(0.7-2.81)	2.18(1.14-4.19)	1.96(1.77-2.15)	63.45(43.32-93.1)	91.01(62.14-140.48)	1.75(1.55-1.94)	0.26(0.16-0.4)	0.18(0.1-0.3)	-3.78(-4.76--2.8)	11.3(7.25-16.56)	9.38(6.1-14.27)	-1.94(-2.49--1.4)
Albania	Female	1.05(0.43-2.26)	1.72(0.73-3.65)	2.07(1.89-2.24)	66.07(41.28-102.65)	101.36(62.8-158.12)	1.86(1.68-2.04)	0.24(0.14-0.36)	0.16(0.09-0.27)	-4.38(-5.51--3.24)	10.69(6.93-15.81)	8.41(5.47-12.91)	-2.45(-3.09--1.8)
Albania	Male	1.76(0.85-3.41)	2.65(1.31-5.06)	1.95(1.76-2.15)	60.84(37.92-93.14)	81.24(51-127.9)	1.63(1.42-1.85)	0.28(0.17-0.44)	0.2(0.11-0.33)	-3.29(-4.14--2.43)	11.87(7.53-17.53)	10.41(6.57-16.27)	-1.49(-1.96--1.02)
Algeria	Both	1.38(0.62-2.99)	2.57(1.29-5.05)	2.38(2.29-2.47)	39.71(25.2-65.41)	70.52(45.27-112.24)	2.28(2.15-2.42)	1.08(0.65-1.81)	0.79(0.47-1.23)	-0.93(-1.13--0.72)	39.33(23.57-64.24)	29.9(19-45.35)	-0.78(-0.99--0.57)
Algeria	Female	1.22(0.46-2.88)	2.12(0.86-4.66)	2.12(2.03-2.2)	41.1(24.37-67.74)	72.08(43.45-117.86)	2.19(2.04-2.35)	1.13(0.64-2.09)	0.86(0.5-1.4)	-0.71(-0.99--0.43)	42.92(24.51-77.99)	32.87(20.26-52.07)	-0.7(-0.98--0.42)
Algeria	Male	1.53(0.68-3.13)	3.01(1.49-5.67)	2.57(2.46-2.68)	38.39(22.61-64.46)	68.91(42.03-113.96)	2.37(2.24-2.5)	1.04(0.61-1.68)	0.72(0.42-1.2)	-1.17(-1.3--1.03)	35.93(21.76-55.59)	26.94(16.74-42.54)	-0.9(-1.04--0.75)
American Samoa	Both	1.1(0.33-2.75)	1.51(0.44-3.77)	1.14(1.1-1.18)	29.78(17.26-49.4)	35.72(19.85-64.08)	0.72(0.67-0.76)	3.28(2.2-4.65)	5.06(3.22-7.45)	1.57(1.45-1.7)	127(87.93-173.51)	199.29(131.91-288.16)	1.63(1.52-1.75)
American Samoa	Female	0.87(0.22-2.36)	1.3(0.32-3.54)	1.42(1.39-1.46)	27.81(16.25-49.55)	33.83(18.75-62.41)	0.77(0.71-0.82)	2.48(1.58-3.64)	4.41(2.79-6.49)	1.94(1.63-2.24)	95.6(62.99-136.18)	173.78(113.61-249.96)	2(1.7-2.31)
American Samoa	Male	1.31(0.37-3.16)	1.71(0.5-4.15)	0.96(0.91-1)	31.5(17.32-56.08)	37.48(19.76-70.94)	0.69(0.65-0.73)	4.03(2.65-5.77)	5.73(3.65-8.39)	1.36(1.29-1.43)	156.27(105-215.59)	225.62(146.51-330.43)	1.42(1.35-1.5)
Andorra	Both	1.37(0.88-2.11)	1.69(1.08-2.5)	1.32(1.1-1.53)	64.68(47.84-87.98)	79.91(57.62-110.51)	1.28(1.1-1.46)	0.24(0.12-0.42)	0.2(0.11-0.33)	-0.6(-0.79--0.4)	8.06(5.25-12.28)	7.46(4.98-10.69)	-0.13(-0.24--0.03)
Andorra	Female	1.05(0.58-1.82)	1.29(0.73-2.17)	1.39(1.15-1.63)	67.61(45.45-99.08)	86.39(57.83-128.17)	1.36(1.19-1.54)	0.21(0.11-0.39)	0.2(0.11-0.33)	-0.23(-0.54-0.09)	7.16(4.64-11.2)	7.12(4.69-10.34)	0.13(-0.05-0.3)
Andorra	Male	1.66(0.96-2.67)	2.05(1.25-3.25)	1.31(1.1-1.53)	62.37(42.05-90.12)	73.71(50.52-106.62)	1.16(0.96-1.35)	0.26(0.14-0.46)	0.2(0.11-0.33)	-0.91(-1--0.81)	8.89(5.61-13.55)	7.76(5.11-11.27)	-0.33(-0.4--0.26)
Angola	Both	0.8(0.32-1.83)	1.05(0.43-2.34)	1.2(1.05-1.35)	32.01(20.46-49.42)	41.58(27.13-64.16)	1.26(1.14-1.37)	1.25(0.74-1.99)	1.08(0.61-1.73)	-0.58(-0.63--0.52)	43.74(26.09-69.14)	37.64(21.4-57.78)	-0.56(-0.62--0.5)
Angola	Female	0.55(0.18-1.34)	0.75(0.28-1.78)	1.38(1.19-1.56)	34.05(21.14-53.6)	46.63(29.95-72.87)	1.39(1.27-1.51)	0.9(0.33-1.64)	0.81(0.31-1.39)	-0.5(-0.62--0.37)	33.7(12.86-59.47)	29.54(11.64-48.93)	-0.57(-0.69--0.45)
Angola	Male	1.06(0.41-2.38)	1.35(0.54-3.09)	1.15(1.03-1.28)	30.04(17.25-50.14)	35.68(20.44-59.46)	1(0.88-1.12)	1.59(0.9-2.58)	1.39(0.83-2.21)	-0.49(-0.56--0.42)	53.61(31.62-84.14)	47.11(28.9-71.55)	-0.44(-0.53--0.35)
Antigua	Both	1.24(0.47-2.87)	1.98(0.79-4.54)	1.79(1.67-1.91)	42.44(24.23-76.81)	71.46(37.89-135.69)	2.14(1.98-2.29)	1.54(1.02-2.2)	1.9(1.18-2.91)	1.05(0.87-1.24)	56.58(38.55-79.61)	67.85(44.31-99.26)	0.97(0.81-1.14)
Antigua	Female	0.82(0.25-1.99)	1.35(0.43-3.23)	1.89(1.79-1.99)	35.63(19.59-64.93)	58.53(31.34-109.57)	2.06(1.92-2.19)	1.07(0.71-1.57)	1.73(1.06-2.66)	1.91(1.77-2.06)	41.56(28.27-58.34)	60.66(38.78-89.87)	1.62(1.47-1.78)
Antigua	Male	1.69(0.67-3.75)	2.61(1.07-6)	1.72(1.57-1.86)	49.57(26.95-91.29)	84.39(41.58-168.71)	2.16(1.98-2.34)	2.08(1.35-3.01)	2.09(1.33-3.21)	0.36(0.12-0.6)	73.72(49.04-104.93)	75.73(49.63-109.71)	0.45(0.28-0.63)
Argentina	Both	1.28(0.66-2.41)	1.4(0.74-2.54)	0.81(0.59-1.04)	68.33(44.5-101.57)	70.06(46.19-102.85)	0.4(0.18-0.62)	0.99(0.62-1.46)	1.02(0.63-1.53)	-0.11(-0.38-0.16)	36.38(23.7-51.44)	35.38(22.99-50.31)	-0.22(-0.44-0)
Argentina	Female	0.83(0.38-1.8)	1(0.47-2.05)	1.06(0.85-1.28)	66.28(40.21-101.68)	73.9(45.6-114.44)	0.53(0.31-0.75)	0.75(0.48-1.09)	0.78(0.48-1.17)	0.04(-0.25-0.33)	28.22(18.77-39.74)	27.74(18.24-39.56)	-0.11(-0.35-0.13)
Argentina	Male	1.74(0.88-3.27)	1.81(0.92-3.26)	0.69(0.45-0.93)	70.62(44.1-108.01)	66.25(42.9-99.66)	0.27(0.04-0.51)	1.29(0.79-1.95)	1.3(0.8-1.98)	-0.2(-0.47-0.07)	45.56(29.04-64.95)	43.99(28.06-63.29)	-0.29(-0.51--0.07)
Armenia	Both	1.27(0.53-2.75)	2.05(0.91-4.21)	2.04(1.73-2.35)	57.85(39-84.88)	80.43(53.38-120.68)	1.56(1.27-1.85)	0.06(0.03-0.08)	0.25(0.15-0.38)	4.89(4.15-5.64)	3.41(2.24-4.72)	11.37(7.55-16.16)	4.07(3.51-4.62)
Armenia	Female	1.08(0.34-2.58)	1.54(0.51-3.54)	1.49(1.18-1.79)	55.52(33.31-86.53)	78.88(46.27-123.33)	1.53(1.25-1.81)	0.04(0.02-0.06)	0.17(0.1-0.26)	4.93(3.99-5.87)	2.48(1.62-3.55)	7.64(5.06-10.9)	3.92(3.25-4.59)
Armenia	Male	1.49(0.65-3.09)	2.6(1.19-5.2)	2.43(2.12-2.75)	60.69(38.2-95.28)	82.86(52.52-130.91)	1.6(1.3-1.9)	0.08(0.05-0.12)	0.36(0.22-0.54)	4.88(4.25-5.51)	4.47(2.92-6.25)	15.71(10.36-22.2)	4.19(3.68-4.7)
Australia	Both	1.66(1.08-2.53)	2.23(1.46-3.35)	1.59(1.35-1.85)	76.39(52.87-106.51)	103.09(73.3-142.46)	1.59(1.36-1.81)	0.08(0.06-0.09)	0.08(0.05-0.13)	0.63(0.16-1.11)	4.34(3.38-5.73)	5.64(3.79-8.11)	1.33(1.19-1.46)
Australia	Female	1.47(0.8-2.5)	1.8(0.98-3.01)	1.24(0.96-1.52)	86.72(56.46-132.04)	116.65(77.73-174.93)	1.44(1.24-1.65)	0.07(0.06-0.09)	0.07(0.04-0.11)	0.59(0.04-1.13)	4.15(3.09-5.75)	5.13(3.34-7.51)	1.14(1-1.28)
Australia	Male	1.87(1.09-3.12)	2.68(1.61-4.42)	1.83(1.58-2.08)	66.02(42.11-97.27)	89.33(59.7-130.29)	1.78(1.5-2.05)	0.08(0.07-0.1)	0.09(0.05-0.15)	0.66(0.23-1.09)	4.55(3.56-5.93)	6.18(4.1-9.03)	1.49(1.35-1.63)
Austria	Both	1.15(0.7-1.84)	1.83(1.21-2.68)	2.24(2-2.48)	56.54(39.68-80.11)	89.73(65.2-123.35)	2.22(2.02-2.41)	0.12(0.07-0.2)	0.21(0.13-0.35)	2.16(1.81-2.5)	5.6(3.68-7.96)	8.3(5.53-11.38)	1.54(1.3-1.79)
Austria	Female	0.83(0.43-1.5)	1.4(0.77-2.36)	2.48(2.25-2.72)	57.66(36.39-87.15)	98.99(63.57-147.84)	2.44(2.25-2.63)	0.1(0.06-0.17)	0.19(0.11-0.32)	2.47(2.14-2.8)	4.6(3.05-6.56)	7.29(5.01-10.05)	1.85(1.62-2.09)
Austria	Male	1.51(0.84-2.52)	2.27(1.35-3.55)	2.05(1.77-2.33)	55.99(36.4-81.77)	81.07(57.01-114.9)	1.94(1.68-2.19)	0.14(0.08-0.23)	0.24(0.14-0.39)	1.82(1.47-2.17)	6.7(4.29-9.65)	9.36(6.06-13.24)	1.28(1.02-1.54)
Azerbaijan	Both	1.66(0.72-3.67)	2.55(1.12-5.48)	1.82(1.58-2.07)	62.17(42.58-93.66)	84.63(56.95-132.11)	1.48(1.24-1.73)	0.35(0.21-0.51)	0.47(0.3-0.71)	1.18(0.78-1.58)	16.72(10.73-24.37)	21.4(14.02-31.77)	0.79(0.44-1.14)
Azerbaijan	Female	1.35(0.45-3.4)	2.03(0.69-5.02)	1.68(1.43-1.92)	58.28(36.92-97.73)	82.25(50.18-140.95)	1.5(1.24-1.76)	0.27(0.17-0.4)	0.4(0.25-0.59)	1.45(1.09-1.81)	13.25(8.44-19.7)	18.11(11.57-26.56)	1.01(0.71-1.3)
Azerbaijan	Male	2(0.88-4.18)	3.08(1.36-6.25)	1.91(1.65-2.16)	66.53(42.96-102.06)	87.62(55.63-140.22)	1.48(1.23-1.72)	0.44(0.27-0.66)	0.56(0.34-0.87)	0.97(0.52-1.41)	20.61(13.05-30.39)	24.99(16.05-37.58)	0.63(0.21-1.05)

Bahamas	Both	1.22(0.48-2.86)	1.95(0.79-4.46)	1.77(1.61-1.92)	34.28(22.19-55.18)	49.47(31.69-81.23)	1.56(1.42-1.7)	1.63(1.06-2.34)	1.91(1.2-2.83)	0.82(0.72-0.91)	62.7(42.59-88.17)	74.91(49.02-109.73)	0.84(0.76-0.92)
Bahamas	Female	0.87(0.28-2.17)	1.41(0.47-3.41)	1.78(1.64-1.91)	30.41(18.67-50.19)	45.05(27.83-75.31)	1.64(1.51-1.77)	1.38(0.9-1.98)	1.63(1.03-2.47)	0.92(0.79-1.06)	54.18(36.84-75.2)	64(41.83-94.18)	0.85(0.74-0.96)
Bahamas	Male	1.58(0.63-3.45)	2.5(0.99-5.31)	1.77(1.61-1.94)	38.36(23.41-64.68)	54.23(32.39-91.89)	1.5(1.35-1.65)	1.92(1.23-2.82)	2.22(1.39-3.33)	0.71(0.62-0.79)	72.36(48.16-103.92)	86.96(56.95-128.07)	0.82(0.74-0.9)
Bahrain	Both	1.17(0.51-2.41)	2.26(1.1-4.33)	2.54(2.33-2.76)	36.31(23.5-57.77)	60.47(39.13-96.91)	1.96(1.77-2.15)	0.9(0.54-1.42)	0.71(0.44-1.12)	-0.91(-1.38--0.43)	27.64(17.32-41.87)	22.79(15.05-33.56)	-0.84(-1.21--0.47)
Bahrain	Female	1.02(0.36-2.35)	1.78(0.67-4.07)	2.2(2.02-2.37)	38.8(23.5-65.6)	64.1(39.27-107.79)	1.9(1.71-2.1)	0.86(0.51-1.36)	0.63(0.39-1.01)	-1.31(-1.67--0.96)	27.5(16.89-42.53)	20.7(13.47-30.5)	-1.29(-1.58--1.01)
Bahrain	Male	1.28(0.56-2.63)	2.55(1.23-4.88)	2.67(2.46-2.89)	34.89(21.37-55.41)	58.38(35.82-94.27)	2.06(1.87-2.25)	0.94(0.56-1.59)	0.76(0.47-1.23)	-0.7(-1.29--0.11)	28.05(17.27-45.68)	24.14(15.71-36.24)	-0.61(-1.07--0.14)
Bangladesh	Both	0.77(0.35-1.64)	1.17(0.55-2.45)	1.62(1.43-1.81)	34.81(23-52.18)	46.17(31.09-69.91)	1.44(1.27-1.6)	0.64(0.35-1.04)	0.5(0.28-0.78)	-0.48(-0.8--0.17)	22.63(12.91-34.8)	18.2(10.88-28.13)	-0.24(-0.53-0.06)
Bangladesh	Female	0.37(0.15-0.82)	0.63(0.25-1.39)	2.06(1.79-2.34)	28.36(17.85-44.99)	38.53(23.56-61.11)	1.52(1.32-1.72)	0.42(0.22-0.69)	0.36(0.2-0.58)	-0.09(-0.36-0.17)	13.86(7.47-22.64)	12.19(7.08-19.21)	0.14(-0.14-0.42)
Bangladesh	Male	1.14(0.5-2.44)	1.7(0.78-3.56)	1.57(1.39-1.76)	40.74(25.76-63.03)	53.79(34.38-84.18)	1.44(1.28-1.6)	0.83(0.43-1.37)	0.64(0.35-1.02)	-0.53(-0.85--0.2)	30.11(16.54-46.98)	24.31(14.68-37.95)	-0.23(-0.52-0.07)
Barbados	Both	1.07(0.39-2.58)	1.66(0.64-3.93)	1.69(1.55-1.83)	29.17(17.88-47.96)	42.31(26.08-70.65)	1.58(1.44-1.72)	1.07(0.71-1.53)	1.39(0.88-2.1)	0.9(0.72-1.08)	40.73(27.74-54.94)	52.07(34.18-74.97)	0.86(0.69-1.03)
Barbados	Female	0.76(0.21-2.08)	1.23(0.36-3.42)	1.78(1.67-1.88)	25.69(15.45-43.14)	38.03(22.48-64.87)	1.6(1.49-1.72)	0.87(0.57-1.23)	1.29(0.81-1.98)	1.7(1.44-1.96)	33.23(22.33-46.14)	47.8(31.4-70.05)	1.51(1.29-1.73)
Barbados	Male	1.4(0.52-3.19)	2.11(0.84-4.82)	1.64(1.48-1.8)	32.93(19.5-57.02)	46.84(27.72-83.07)	1.55(1.39-1.71)	1.32(0.87-1.89)	1.5(0.94-2.24)	0.16(-0.05-0.37)	49.51(33.73-67.55)	56.79(37.02-81.83)	0.29(0.08-0.5)
Barbuda	Both	1.24(0.47-2.87)	1.98(0.79-4.54)	1.79(1.67-1.91)	42.44(24.23-76.81)	71.46(37.89-135.69)	2.14(1.98-2.29)	1.54(1.02-2.2)	1.9(1.18-2.91)	1.05(0.87-1.24)	56.58(38.55-79.61)	67.85(44.31-99.26)	0.97(0.81-1.14)
Barbuda	Female	0.82(0.25-1.99)	1.35(0.43-3.23)	1.89(1.79-1.99)	35.63(19.59-64.93)	58.53(31.34-109.57)	2.06(1.92-2.19)	1.07(0.71-1.57)	1.73(1.06-2.66)	1.91(1.77-2.06)	41.56(28.27-58.34)	60.66(38.78-89.87)	1.62(1.47-1.78)
Barbuda	Male	1.69(0.67-3.75)	2.61(1.07-6)	1.72(1.57-1.86)	49.57(26.95-91.29)	84.39(41.58-168.71)	2.16(1.98-2.34)	2.08(1.35-3.01)	2.09(1.33-3.21)	0.36(0.12-0.6)	73.72(49.04-104.93)	75.73(49.63-109.71)	0.45(0.28-0.63)
Belarus	Both	1.2(0.5-2.58)	2.24(1.09-4.4)	2.76(2.23-3.29)	65.09(43.63-95.92)	127.17(86.23-189.92)	2.99(2.57-3.4)	0.13(0.08-0.19)	0.21(0.13-0.33)	2.33(1.36-3.32)	4.49(3.13-6.19)	8.89(5.88-13.03)	3.21(2.27-4.16)
Belarus	Female	0.96(0.35-2.35)	1.69(0.67-3.84)	2.37(1.81-2.94)	65.02(39.47-105.71)	137.06(83.11-225.59)	3.17(2.75-3.6)	0.11(0.07-0.17)	0.17(0.1-0.26)	2.1(1.28-2.92)	3.68(2.56-5.14)	6.8(4.37-10.16)	3.03(2.25-3.83)
Belarus	Male	1.45(0.63-3.09)	2.85(1.39-5.61)	3.05(2.54-3.56)	65.36(41.12-99.27)	118.35(76.76-179.97)	2.81(2.41-3.21)	0.16(0.1-0.24)	0.27(0.17-0.43)	2.41(1.35-3.48)	5.57(3.84-7.68)	11.39(7.48-16.59)	3.29(2.26-4.33)
Belgium	Both	1.14(0.68-1.94)	1.53(0.99-2.45)	1.61(1.39-1.83)	51.35(36.19-72.74)	70.6(50.68-99.8)	1.64(1.42-1.85)	0.14(0.08-0.23)	0.11(0.06-0.19)	-2.01(-2.4--1.63)	6.07(4.04-8.71)	5.56(3.81-7.71)	-0.81(-1.07--0.56)
Belgium	Female	0.82(0.42-1.53)	1.12(0.6-1.99)	1.7(1.47-1.93)	51.74(34.26-78.67)	73.93(47.8-112.09)	1.77(1.57-1.98)	0.13(0.07-0.21)	0.1(0.06-0.17)	-2.03(-2.38--1.67)	5.36(3.56-7.69)	4.8(3.18-6.75)	-0.84(-1.1--0.58)
Belgium	Male	1.46(0.83-2.63)	1.97(1.18-3.34)	1.55(1.32-1.78)	51.29(33.81-76.27)	67.52(45.73-99.86)	1.48(1.25-1.71)	0.16(0.09-0.26)	0.13(0.07-0.21)	-2.05(-2.47--1.63)	6.84(4.57-9.84)	6.35(4.23-9.04)	-0.81(-1.07--0.56)
Belize	Both	1.4(0.62-3.14)	2.2(0.93-4.88)	1.79(1.69-1.9)	39.45(25.68-62.72)	54.07(34.26-91.75)	1.43(1.29-1.56)	1.24(0.79-1.8)	2.59(1.67-3.84)	2.62(2.09-3.15)	46.01(31.1-63.86)	99.56(66.96-141.48)	2.64(2.13-3.15)
Belize	Female	0.95(0.3-2.38)	1.56(0.5-3.94)	1.83(1.76-1.89)	32.48(19.61-51.99)	45.81(27.42-77.56)	1.43(1.3-1.57)	1.26(0.8-1.82)	2.4(1.52-3.55)	2.57(2.08-3.06)	46.52(30.81-65.22)	91.75(61.12-130.88)	2.55(2.06-3.05)
Belize	Male	1.83(0.79-3.99)	2.83(1.19-6.26)	1.82(1.69-1.95)	46.03(28.9-75.62)	62.28(38.44-108.31)	1.45(1.3-1.6)	1.21(0.78-1.8)	2.79(1.79-4.14)	2.68(2.08-3.28)	45.52(30.81-64.06)	107.57(72.1-153.04)	2.74(2.19-3.29)
Benin	Both	1.42(0.59-3.01)	1.66(0.68-3.5)	0.76(0.62-0.89)	51.87(35.35-76.84)	54.45(35.64-83.1)	0.44(0.21-0.68)	1.66(1.05-2.49)	1.6(0.92-2.5)	0.02(-0.08-0.11)	59.12(37.85-86.74)	58.22(34.34-89.29)	0.1(0.01-0.2)
Benin	Female	1.07(0.4-2.41)	1.25(0.46-2.84)	0.74(0.56-0.93)	51.07(32.53-78.49)	54.78(33.64-83.6)	0.43(0.15-0.71)	1.49(0.93-2.25)	1.43(0.8-2.28)	0.05(-0.08-0.17)	54.29(34.27-81.21)	53.24(30.32-84.84)	0.11(-0.01-0.24)
Benin	Male	1.78(0.69-3.82)	2.07(0.82-4.44)	0.77(0.66-0.89)	52.46(32.13-85.3)	53.93(33.28-88.29)	0.48(0.28-0.68)	1.85(1.13-2.87)	1.79(0.98-2.92)	0.02(-0.09-0.13)	64.37(40.51-97.33)	63.75(36.07-103.21)	0.09(-0.01-0.2)
Bermuda	Both	1.1(0.54-2.24)	2.01(1.11-3.79)	2.47(2.29-2.65)	37.57(25.82-56.44)	60.62(42.71-88.78)	2.13(1.95-2.32)	0.84(0.54-1.24)	0.65(0.4-1)	-0.61(-0.84--0.38)	30.88(20.55-42.73)	26.33(17.62-38.03)	-0.22(-0.46-0.01)
Bermuda	Female	0.76(0.31-1.7)	1.3(0.58-2.71)	2.22(2.05-2.38)	34.91(22.8-53.58)	54.98(36.48-82.06)	2.03(1.84-2.23)	0.6(0.38-0.9)	0.46(0.28-0.71)	-1.07(-1.25--0.9)	22.4(14.98-32.38)	19.19(12.81-27.57)	-0.56(-0.72--0.4)
Bermuda	Male	1.46(0.69-2.99)	2.72(1.43-5.09)	2.59(2.39-2.79)	40.33(26.64-63.87)	66.43(44.72-100.3)	2.22(2.04-2.4)	1.13(0.7-1.66)	0.86(0.53-1.32)	-0.35(-0.67--0.04)	40.33(26.67-56.87)	33.84(22.33-48.82)	-0.04(-0.35-0.26)
Bhutan	Both	1.13(0.5-2.31)	1.58(0.7-3.28)	1.56(1.4-1.71)	42.6(28.17-64.57)	53.11(35.23-82.68)	1.15(1-1.31)	1.25(0.65-2.12)	1.46(0.8-2.37)	0.6(0.53-0.68)	44.23(23.76-75.68)	50.03(28.4-82.24)	0.48(0.4-0.56)
Bhutan	Female	0.84(0.31-1.94)	1.14(0.43-2.62)	1.44(1.29-1.6)	38.01(23.38-58.18)	49.78(30.47-77.2)	1.29(1.14-1.44)	1.27(0.58-2.22)	1.46(0.69-2.53)	0.4(0.34-0.46)	45.23(21.37-77.92)	49.73(24.21-85.16)	0.23(0.16-0.3)
Bhutan	Male	1.4(0.61-2.93)	1.99(0.85-4.3)	1.63(1.46-1.8)	46.76(29.79-72.8)	56.45(35.39-92.4)	1.07(0.9-1.24)	1.23(0.59-2.41)	1.46(0.77-2.5)	0.79(0.67-0.92)	43.43(21.78-83.03)	50.37(27.14-85.88)	0.72(0.59-0.84)
Bolivia	Both	1.17(0.52-2.61)	1.63(0.74-3.59)	1.41(1.31-1.51)	37.63(24.61-61.3)	51.6(33.56-85.63)	1.47(1.35-1.6)	2(1.23-3.09)	2.37(1.34-3.93)	0.59(0.51-0.66)	68.99(44.33-103.55)	74.96(43.65-119.15)	0.28(0.2-0.35)
Bolivia	Female	0.77(0.27-1.91)	1.15(0.44-2.7)	1.61(1.5-1.71)	35.18(20.99-58.08)	52.22(31.53-84.92)	1.7(1.59-1.81)	1.93(1.18-3.04)	2.35(1.32-3.9)	0.63(0.57-0.69)	67.83(43.44-105.07)	75.34(45.07-122.76)	0.28(0.22-0.35)
Bolivia	Male	1.58(0.71-3.33)	2.11(0.95-4.53)	1.29(1.18-1.4)	40.08(24.8-67.68)	50.94(30.27-87.97)	1.27(1.12-1.41)	2.09(1.24-3.31)	2.39(1.31-4.06)	0.54(0.42-0.65)	70.36(43.6-106.61)	74.69(42.63-124.11)	0.27(0.15-0.39)
Bosnia and Herzegovina	Both	0.87(0.4-1.77)	1.35(0.66-2.67)	2.01(1.84-2.18)	40.72(27.49-61.83)	60.84(40.92-90.82)	1.92(1.71-2.13)	0.37(0.22-0.58)	0.44(0.25-0.7)	2.01(0.83-3.21)	13.7(8.67-20.71)	15.7(9.98-23.74)	1.46(0.56-2.36)
Bosnia and Herzegovina	Female	0.67(0.25-1.54)	1.08(0.42-2.46)	2.15(1.96-2.33)	42.91(27.58-69.16)	69.18(42.96-110.27)	2.12(1.91-2.33)	0.37(0.22-0.59)	0.39(0.22-0.63)	1.14(-0.18-2.48)	13.34(8.46-20.21)	13.67(8.73-20.93)	0.59(-0.46-1.64)
Bosnia and Herzegovina	Male	1.08(0.51-2.17)	1.63(0.82-3.12)	1.92(1.76-2.09)	38.98(25.25-59.26)	52.92(33.98-79.71)	1.69(1.47-1.91)	0.36(0.21-0.55)	0.49(0.28-0.78)	2.94(1.85-4.05)	13.98(8.87-21)	17.83(11.2-27.2)	2.25(1.44-3.08)
Botswana	Both	1.29(0.64-2.55)	1.82(0.88-3.59)	1.46(1.2-1.72)	45.63(30.9-65.71)	57.66(38.25-83.09)	1.16(0.97-1.34)	1.19(0.65-2.04)	1.76(0.97-2.91)	0.75(0.17-1.34)	42.97(24.15-73.31)	63.46(35.69-103.88)	0.75(0.16-1.34)

Botswana	Female	0.67(0.25-1.46)	1.08(0.4-2.36)	1.78(1.56-1.99)	40.6(25.11-63.27)	51.84(31.92-81.71)	1.04(0.94-1.15)	0.73(0.35-1.47)	1.28(0.65-2.33)	2.09(1.84-2.34)	26.51(13.5-52.71)	46.31(24.16-82.91)	2.12(1.87-2.38)
Botswana	Male	1.98(0.95-3.76)	2.6(1.23-5)	1.28(1.01-1.56)	51.15(33.58-73.83)	63.96(41.22-95.43)	1.26(1.01-1.52)	1.75(0.93-3)	2.34(1.23-4.04)	0.15(-0.66-0.97)	62.13(34.32-104.55)	83.04(44.48-142.42)	0.12(-0.7-0.95)
Brazil	Both	1.36(1.05-1.78)	2.04(1.63-2.54)	1.3(1.05-1.55)	44.09(38.1-52.22)	72.77(62.68-84.75)	1.62(1.34-1.91)	0.96(0.65-1.34)	0.76(0.49-1.1)	-1(-1.14--0.87)	37.81(26.4-50.86)	29.19(20.26-40)	-1.12(-1.26--0.99)
Brazil	Female	1.04(0.79-1.4)	1.75(1.38-2.21)	1.86(1.76-1.96)	45.73(38.99-54.43)	87.43(74.46-104.31)	2.25(2.07-2.43)	0.84(0.56-1.16)	0.66(0.43-0.95)	-1.06(-1.22--0.89)	33.45(22.98-45.26)	26.52(18.58-35.96)	-1.08(-1.25--0.92)
Brazil	Male	1.68(1.3-2.22)	2.33(1.85-2.95)	0.92(0.56-1.29)	42.27(35.65-50.56)	57.35(48.97-68.31)	0.83(0.37-1.29)	1.1(0.72-1.52)	0.87(0.56-1.29)	-0.93(-1.04--0.82)	42.47(29.42-57.23)	32.24(22-44.74)	-1.14(-1.25--1.03)
Brunei	Both	1.13(0.5-2.34)	1.19(0.54-2.46)	0.49(0.3-0.68)	46.14(31.12-68.06)	52.59(34.04-79.69)	0.85(0.68-1.01)	2.49(1.69-3.56)	2.19(1.45-3.19)	0.1(-0.29-0.49)	85.42(59.29-117.79)	73.75(50.57-103.13)	-0.04(-0.46-0.37)
Brunei	Female	0.74(0.28-1.62)	0.82(0.33-1.79)	0.75(0.46-1.04)	45.85(29.44-69.78)	56.97(35.07-89.99)	1.13(0.95-1.31)	2.18(1.42-3.19)	1.9(1.25-2.77)	0.11(-0.25-0.46)	78.28(52.45-111.12)	65(44.54-93.27)	-0.05(-0.48-0.38)
Brunei	Male	1.49(0.59-3.18)	1.54(0.64-3.27)	0.37(0.21-0.52)	46.68(29.47-75.37)	49(30.59-79.45)	0.57(0.43-0.71)	2.91(1.94-4.24)	2.58(1.66-3.8)	0.16(-0.31-0.63)	93.65(64.48-130.7)	83.19(56.08-117.17)	-0.01(-0.43-0.41)
Bulgaria	Both	1.1(0.51-2.43)	2.14(1.09-4.41)	2.87(2.66-3.08)	44.15(30.18-66.69)	76.62(53.38-115.68)	2.67(2.4-2.94)	0.28(0.18-0.42)	0.56(0.32-0.91)	2.29(1.92-2.67)	12.4(8.23-18.05)	23.26(14.27-36.33)	2.18(1.87-2.49)
Bulgaria	Female	0.78(0.31-1.94)	1.61(0.68-3.7)	3.06(2.82-3.3)	44.35(28.04-69.84)	82.02(51.89-128.34)	2.91(2.61-3.21)	0.24(0.15-0.36)	0.45(0.26-0.74)	2.04(1.65-2.44)	10.58(7.03-15.66)	19.07(11.65-29.62)	1.99(1.66-2.33)
Bulgaria	Male	1.41(0.66-3.05)	2.68(1.36-5.41)	2.78(2.58-2.97)	44.03(27.71-70.66)	72.12(47.13-112.4)	2.46(2.21-2.7)	0.33(0.2-0.5)	0.68(0.39-1.1)	2.51(2.12-2.9)	14.33(9.35-21.06)	27.64(16.95-43.34)	2.31(1.99-2.63)
Burkina Faso	Both	1.13(0.47-2.33)	1.43(0.58-2.96)	1.17(1.02-1.31)	45.31(29.8-64.5)	51.79(33.99-76.83)	0.92(0.77-1.08)	1.35(0.85-2.11)	1.44(0.89-2.17)	0.14(-0.01-0.3)	46.49(30.17-71.48)	51.38(32.26-76.53)	0.28(0.12-0.43)
Burkina Faso	Female	0.85(0.32-1.86)	1.07(0.41-2.33)	1.16(1-1.31)	43.52(27.02-65.88)	52.4(32.81-79.42)	1.06(0.91-1.22)	1.17(0.73-1.84)	1.33(0.8-2.04)	0.34(0.2-0.49)	42.15(26.54-65.68)	48.39(30-72.63)	0.42(0.28-0.56)
Burkina Faso	Male	1.42(0.57-3.01)	1.8(0.7-3.83)	1.18(1.04-1.33)	47.22(28.41-75.47)	50.75(30.31-81.9)	0.77(0.6-0.93)	1.53(0.92-2.4)	1.57(0.95-2.43)	0.01(-0.18-0.19)	51.18(31.9-79.3)	54.91(33.91-82.35)	0.17(-0.02-0.35)
Burundi	Both	0.92(0.43-2.02)	1.06(0.52-2.2)	0.91(0.74-1.08)	40.66(27.8-57.54)	52.8(36.1-75.65)	1.42(1.25-1.6)	1.58(0.96-2.49)	1.09(0.63-1.74)	-1.71(-1.88--1.53)	54.95(33.87-84.67)	37.58(22.64-58)	-1.73(-1.91--1.55)
Burundi	Female	0.59(0.24-1.29)	0.72(0.31-1.51)	1.08(0.89-1.28)	40.79(26.24-61.66)	57.12(36.56-87.78)	1.64(1.46-1.83)	1.32(0.77-2.15)	0.93(0.52-1.51)	-1.71(-1.92--1.5)	47.95(28.51-78.04)	32.82(18.93-52.24)	-1.82(-2.04--1.61)
Burundi	Male	1.28(0.53-2.87)	1.4(0.62-3.02)	0.74(0.58-0.89)	40.36(25.17-61.69)	48.46(30.98-73.97)	1.2(1.03-1.38)	1.9(1.09-3.09)	1.24(0.7-2.01)	-1.85(-2.01--1.68)	63.39(37.65-100.52)	41.96(25.05-65.07)	-1.77(-1.94--1.61)
Cambodia	Both	0.87(0.33-2.05)	1.05(0.37-2.55)	0.86(0.76-0.96)	37.57(25.43-57.04)	39.05(25.12-61.83)	0.43(0.31-0.56)	2.93(2.02-4.12)	2.04(1.35-2.91)	-1.39(-1.47--1.31)	126.55(89.03-172.89)	85.27(58.28-119.07)	-1.5(-1.58--1.43)
Cambodia	Female	0.77(0.24-1.99)	0.9(0.27-2.39)	0.76(0.66-0.87)	37.91(24.47-58.94)	40.88(25.5-67.27)	0.56(0.42-0.7)	3(2.01-4.32)	1.93(1.27-2.78)	-1.79(-1.92--1.67)	131.11(88.98-184.59)	80.79(54.71-113.9)	-1.95(-2.06--1.83)
Cambodia	Male	0.98(0.38-2.26)	1.2(0.43-2.85)	0.89(0.79-1)	37.25(23.86-56.3)	37.28(22.6-60.69)	0.3(0.19-0.41)	2.84(1.91-4.1)	2.18(1.44-3.07)	-0.91(-0.95--0.86)	121.11(83.71-169.52)	90.4(60.95-125.37)	-0.99(-1.02--0.96)
Cameroon	Both	2.04(0.89-4.37)	2.58(1.15-5.44)	0.96(0.74-1.18)	66.46(44.39-101.37)	75.84(49.25-118.45)	0.51(0.02-1.01)	2.59(1.61-3.83)	2.47(1.46-3.9)	-0.23(-0.46-0)	95.08(60.13-138.04)	92.7(55.48-143.86)	-0.18(-0.48-0.11)
Cameroon	Female	1.73(0.74-3.89)	2.01(0.84-4.56)	0.6(0.31-0.88)	67.25(41.92-106.34)	76.02(46.39-122.2)	0.38(-0.17-0.93)	2.88(1.67-4.5)	2.35(1.28-3.79)	-0.8(-0.99--0.61)	107.96(63.54-167.37)	89.9(49.35-141.24)	-0.76(-1.02--0.5)
Cameroon	Male	2.35(0.98-5.1)	3.15(1.35-6.76)	1.22(1.04-1.4)	65.47(40.88-105.63)	75.43(46.55-124.86)	0.66(0.23-1.1)	2.3(1.36-3.64)	2.59(1.5-4.23)	0.38(0.08-0.68)	81.37(50.23-127.93)	95.62(57.52-151.99)	0.47(0.12-0.83)
Canada	Both	3.21(2.13-4.72)	2.81(1.86-4.13)	0.6(0.16-1.04)	174.26(122.73-243.94)	155.17(110.19-217.49)	0.47(-0.02-0.96)	0.12(0.07-0.19)	0.11(0.07-0.18)	-0.59(-0.8--0.39)	7.94(5.38-11.05)	7.4(4.93-10.27)	0.18(-0.04-0.4)
Canada	Female	2.49(1.4-4.12)	2.14(1.25-3.53)	0.73(0.18-1.29)	194.54(122.45-296.65)	179.56(114.06-270.94)	0.64(0.09-1.19)	0.11(0.06-0.18)	0.11(0.06-0.17)	-0.33(-0.5--0.16)	7.05(4.6-10.25)	6.7(4.45-9.37)	0.37(0.09-0.66)
Canada	Male	3.97(2.48-6.17)	3.5(2.15-5.31)	0.5(0.12-0.88)	153.73(105.06-224.7)	130.39(91.02-187.98)	0.22(-0.18-0.62)	0.14(0.08-0.22)	0.12(0.07-0.19)	-0.89(-1.14--0.63)	8.89(5.69-12.89)	8.13(5.22-11.43)	0(-0.18-0.18)
Central African Republic	Both	0.82(0.32-1.93)	0.98(0.38-2.31)	0.88(0.75-1.02)	30.11(19.84-45.03)	34.37(22.45-53.07)	0.82(0.64-0.99)	1.66(0.94-2.69)	1.65(0.93-2.67)	0.01(-0.14-0.16)	58.47(33.11-93.36)	58.49(33.42-94.78)	0.03(-0.11-0.17)
Central African Republic	Female	0.56(0.19-1.38)	0.7(0.24-1.75)	0.99(0.8-1.18)	32.28(20.65-49.91)	38.07(23.99-59.78)	0.88(0.69-1.07)	1.25(0.59-2.11)	1.19(0.51-2.09)	-0.12(-0.27-0.04)	46.32(21.56-77.58)	43.83(19.68-77.23)	-0.13(-0.27-0.02)
Central African Republic	Male	1.07(0.41-2.65)	1.26(0.49-3.2)	0.84(0.72-0.96)	27.61(16.85-47.29)	30.16(17.94-52.9)	0.73(0.57-0.88)	2.14(1.19-3.5)	2.21(1.27-3.53)	0.12(-0.03-0.27)	72.28(40.39-118.99)	74.97(43.54-120.57)	0.12(-0.02-0.27)
Chad	Both	1.35(0.57-3.04)	1.61(0.67-3.66)	0.87(0.67-1.08)	50.41(33.47-77.33)	54.2(34.97-85.75)	0.66(0.46-0.86)	1.52(0.86-2.56)	1.58(0.93-2.53)	0.28(0.14-0.42)	54.17(31.58-88.92)	56.62(33.59-88.2)	0.33(0.2-0.47)
Chad	Female	1(0.38-2.28)	1.11(0.41-2.58)	0.61(0.37-0.85)	49.11(30.5-78.21)	52.46(32.56-84.09)	0.57(0.37-0.76)	1.43(0.77-2.73)	1.31(0.71-2.28)	-0.17(-0.25--0.08)	52.47(28.82-100.31)	48.19(27.43-83.87)	-0.13(-0.23--0.03)
Chad	Male	1.72(0.69-3.99)	2.1(0.81-5.09)	0.97(0.79-1.14)	51.68(32.02-84.74)	55.6(33.79-95.57)	0.74(0.53-0.94)	1.61(0.93-2.74)	1.81(1.03-2.91)	0.55(0.33-0.77)	55.7(32.33-92.3)	64.42(37.66-101.16)	0.66(0.45-0.88)
Chile	Both	1.03(0.52-1.99)	1.46(0.76-2.72)	1.85(1.63-2.07)	55.44(36.08-81.07)	68.79(44.62-101.79)	1.43(1.19-1.67)	0.54(0.33-0.84)	0.61(0.37-0.95)	0.53(0.17-0.9)	19.68(12.73-28.55)	20.45(13.19-29.7)	0.37(0.1-0.64)
Chile	Female	0.75(0.32-1.6)	1.07(0.48-2.24)	1.78(1.57-1.98)	55.76(33.96-87.56)	72.22(43.06-111.65)	1.46(1.24-1.68)	0.44(0.27-0.68)	0.51(0.31-0.79)	0.56(0.18-0.95)	16.25(10.54-23.76)	17.26(11.28-25.1)	0.39(0.12-0.67)
Chile	Male	1.32(0.65-2.52)	1.87(0.96-3.55)	1.9(1.66-2.14)	55.12(34.13-85.09)	65.53(40.02-102.28)	1.41(1.13-1.69)	0.66(0.4-1.01)	0.72(0.43-1.15)	0.5(0.15-0.85)	23.51(15.17-34.38)	23.98(15.21-34.79)	0.34(0.07-0.62)
China	Both	0.79(0.61-1.02)	0.81(0.64-1.03)	0.44(0.26-0.63)	29.79(25.59-34.58)	39.07(33.62-45.5)	1.02(0.87-1.17)	1.05(0.73-1.41)	0.64(0.44-0.91)	-1.77(-1.95--1.6)	44.9(31.77-59.83)	25.97(18.3-35.12)	-2.07(-2.28--1.86)
China	Female	0.61(0.45-0.81)	0.59(0.45-0.78)	0.21(0.01-0.41)	30.49(25.64-35.76)	43.92(36.86-52.44)	1.21(1.08-1.35)	1.03(0.7-1.46)	0.54(0.36-0.79)	-2.46(-2.71--2.21)	43.68(30.25-60.43)	21.59(14.73-29.81)	-2.82(-3.13--2.51)
China	Male	0.95(0.74-1.21)	1.01(0.8-1.27)	0.57(0.39-0.75)	29.12(24.6-34.23)	34.38(29.42-39.89)	0.8(0.61-0.98)	1.07(0.72-1.43)	0.74(0.49-1.09)	-1.19(-1.34--1.04)	46.06(31.51-62.41)	30.25(20.71-43.12)	-1.45(-1.61--1.28)
Colombia	Both	1.19(0.49-2.69)	1.63(0.69-3.62)	1.2(1.13-1.27)	34.24(21.21-54.95)	45.7(28.54-72.84)	1.3(1.18-1.42)	1.05(0.68-1.5)	0.74(0.43-1.19)	-1.52(-1.77--1.26)	38.74(26.5-53.38)	26.22(16.29-40.01)	-1.65(-1.92--1.37)
Colombia	Female	0.77(0.27-1.87)	0.98(0.36-2.33)	0.94(0.87-1.02)	30.46(18.09-50.26)	40.61(24.69-66.13)	1.31(1.17-1.46)	0.98(0.64-1.39)	0.66(0.39-1.06)	-1.67(-1.97--1.38)	36.2(24.43-49.77)	23.58(14.74-36.16)	-1.77(-2.08--1.46)

Colombia	Male	1.61(0.63-3.65)	2.28(0.94-5.08)	1.33(1.26-1.41)	37.98(22.02-66.04)	50.91(29.22-88.02)	1.31(1.2-1.42)	1.12(0.73-1.62)	0.84(0.48-1.36)	-1.32(-1.57--1.07)	41.35(28.15-57.29)	29.25(17.98-44.43)	-1.5(-1.76--1.24)
Comoros	Both	1.14(0.53-2.48)	1.4(0.69-2.9)	1.1(0.93-1.26)	49.63(34.16-70.23)	65.65(45.04-90.75)	1.54(1.34-1.74)	1.3(0.57-2.25)	1.11(0.66-1.73)	-0.86(-1.12--0.61)	44.37(17.47-73.25)	38.8(23.57-57.84)	-0.79(-1.09--0.49)
Comoros	Female	0.79(0.31-1.81)	1.07(0.45-2.32)	1.5(1.29-1.71)	51.02(33.48-75.65)	73.89(47.63-110.79)	1.84(1.62-2.05)	1.18(0.51-2)	1.12(0.65-1.76)	-0.48(-0.72--0.24)	42.03(15.85-70.59)	39.93(23.76-60.12)	-0.47(-0.76--0.18)
Comoros	Male	1.5(0.67-3.36)	1.73(0.82-3.79)	0.87(0.73-1.02)	48.02(30.18-73.98)	57.32(35.7-89.48)	1.22(1.02-1.41)	1.43(0.62-2.51)	1.11(0.64-1.79)	-1.21(-1.49--0.93)	46.75(18.24-79.81)	37.89(22.37-57.89)	-1.09(-1.41--0.77)
Costa Rica	Both	1.78(0.77-3.88)	2.07(0.83-4.79)	0.74(0.66-0.81)	43.51(28.2-70.82)	49.95(31.45-85.41)	0.82(0.71-0.93)	0.73(0.46-1.07)	1.28(0.75-2.06)	1.7(0.91-2.5)	26.34(17.63-37.04)	44.93(27.63-70.96)	1.67(0.95-2.4)
Costa Rica	Female	1.21(0.49-2.84)	1.45(0.56-3.5)	0.9(0.78-1.03)	40.22(25.13-64.44)	47.45(29.53-76.27)	0.96(0.82-1.09)	0.66(0.42-0.96)	1.02(0.59-1.68)	1.34(0.6-2.08)	23.93(15.78-33.73)	35.57(21.84-56.33)	1.3(0.66-1.95)
Costa Rica	Male	2.33(0.97-5.22)	2.69(1.03-6.35)	0.68(0.62-0.74)	46.65(27.45-84.32)	52.26(29.47-99.76)	0.7(0.61-0.79)	0.8(0.51-1.19)	1.57(0.9-2.56)	2.04(1.2-2.88)	28.81(19.26-40.9)	55.4(33.74-86.49)	2(1.22-2.79)
Croatia	Both	0.96(0.49-1.88)	1.66(0.96-2.93)	3(2.66-3.34)	44.83(30.28-66.73)	73.47(49.94-106.17)	2.84(2.51-3.17)	0.16(0.1-0.25)	0.16(0.09-0.26)	-1.93(-2.61--1.24)	7.05(4.47-10.35)	7.08(4.62-10.34)	-0.87(-1.25--0.49)
Croatia	Female	0.72(0.32-1.59)	1.36(0.68-2.81)	3.26(2.92-3.61)	49.18(30.25-77.15)	86.53(53.93-135.67)	3.06(2.72-3.39)	0.14(0.08-0.22)	0.14(0.07-0.23)	-1.65(-2.27--1.01)	5.78(3.78-8.63)	6.1(3.92-8.77)	-0.56(-0.87--0.24)
Croatia	Male	1.2(0.6-2.31)	1.97(1.12-3.46)	2.84(2.5-3.18)	40.68(25.97-62.16)	61.04(40.1-90.62)	2.57(2.24-2.9)	0.19(0.11-0.3)	0.18(0.1-0.29)	-2.23(-2.97--1.48)	8.37(5.22-12.22)	8.08(5.18-12.07)	-1.11(-1.55--0.68)
Cuba	Both	0.87(0.31-2.23)	1.4(0.57-3.29)	1.86(1.72-2.01)	24.65(14.75-42.37)	37.49(23.54-62.16)	1.86(1.66-2.06)	0.51(0.34-0.71)	0.75(0.45-1.15)	1.61(1.4-1.81)	20.77(14.38-28.79)	28.3(18.21-41.37)	1.23(1.07-1.39)
Cuba	Female	0.68(0.22-1.83)	1.11(0.4-2.82)	1.89(1.78-2.01)	24.36(14.22-42.2)	37.79(22.6-63.8)	1.91(1.72-2.1)	0.44(0.3-0.63)	0.7(0.42-1.07)	2.06(1.66-2.47)	18.3(12.51-25.49)	25.89(16.75-38.28)	1.58(1.26-1.89)
Cuba	Male	1.06(0.35-2.58)	1.68(0.66-3.82)	1.85(1.69-2.02)	24.88(13.63-45.75)	37.22(21.4-66.04)	1.82(1.61-2.03)	0.58(0.39-0.81)	0.81(0.49-1.22)	1.21(1.11-1.32)	23.28(16.06-31.91)	30.76(19.61-45.16)	0.93(0.84-1.03)
Cyprus	Both	0.94(0.53-1.68)	1.62(1.03-2.55)	2.66(2.36-2.96)	46.43(31.45-66.29)	77.38(55.86-107.93)	2.39(2.16-2.62)	0.84(0.48-1.39)	0.43(0.25-0.69)	-2.67(-2.79--2.55)	18.98(12.16-28.63)	11.64(7.8-16.53)	-1.92(-2--1.84)
Cyprus	Female	0.69(0.34-1.3)	1.17(0.63-2.08)	2.57(2.27-2.87)	49.57(31.36-77.86)	84.99(55.33-131.04)	2.41(2.22-2.61)	0.77(0.41-1.34)	0.33(0.19-0.56)	-3.17(-3.35--2.98)	15.71(10.03-24.22)	8.75(5.89-12.65)	-2.26(-2.38--2.14)
Cyprus	Male	1.19(0.59-2.23)	2.09(1.21-3.42)	2.75(2.44-3.06)	43.34(27.96-65.76)	69.73(47.32-102.06)	2.35(2.06-2.63)	0.91(0.53-1.55)	0.53(0.3-0.85)	-2.28(-2.46--2.09)	22.55(14.08-34.37)	14.74(9.69-21.42)	-1.69(-1.78--1.61)
Czech Republic	Both	0.9(0.41-1.84)	1.23(0.58-2.46)	1.48(1.35-1.62)	43.04(29.26-64.38)	57.8(38.8-87.21)	1.51(1.35-1.67)	0.31(0.18-0.48)	0.15(0.08-0.23)	-2.65(-2.8--2.5)	12.14(7.52-18.18)	6.49(4.18-9.33)	-2.03(-2.17--1.89)
Czech Republic	Female	0.68(0.28-1.54)	1.04(0.45-2.37)	1.82(1.68-1.97)	47.26(29.65-75.65)	68.13(40.94-107.6)	1.69(1.54-1.85)	0.22(0.13-0.35)	0.12(0.07-0.2)	-2.38(-2.52--2.25)	8.61(5.43-12.67)	5.49(3.58-7.96)	-1.62(-1.72--1.51)
Czech Republic	Male	1.12(0.5-2.27)	1.43(0.69-2.82)	1.26(1.12-1.4)	38.92(25.28-59.98)	48.07(31.93-73.65)	1.3(1.13-1.48)	0.4(0.23-0.62)	0.17(0.1-0.27)	-2.87(-3.07--2.67)	15.88(9.67-24.07)	7.5(4.74-11.01)	-2.34(-2.52--2.15)
Democratic Republic of the Congo	Both	0.83(0.34-1.8)	1.01(0.43-2.17)	0.91(0.75-1.07)	36.05(23.24-52.98)	41.75(27.42-62.19)	0.9(0.7-1.1)	1.4(0.86-2.13)	1.13(0.68-1.77)	-0.85(-0.91--0.79)	48.8(31.03-73.28)	39.53(25.02-58.73)	-0.82(-0.88--0.76)
Democratic Republic of the Congo	Female	0.62(0.23-1.45)	0.8(0.31-1.81)	1.05(0.86-1.23)	39.19(25.34-60.37)	47.71(30.72-72.64)	1.01(0.79-1.22)	1.21(0.74-1.85)	1(0.57-1.59)	-0.75(-0.83--0.66)	44.15(27.09-66.36)	36.09(21.37-55.28)	-0.81(-0.89--0.73)
Democratic Republic of the Congo	Male	1.04(0.4-2.3)	1.22(0.49-2.62)	0.82(0.68-0.97)	32.49(18.92-53.62)	35.67(21.46-58.01)	0.8(0.61-0.99)	1.62(0.98-2.56)	1.28(0.76-2.02)	-0.9(-0.98--0.83)	54.15(33.65-83.46)	43.56(26.56-67.5)	-0.83(-0.91--0.76)
Denmark	Both	1.51(0.94-2.31)	1.9(1.26-2.78)	1.54(1.29-1.8)	66.05(48.66-89.55)	84.87(63.23-113.64)	1.47(1.25-1.69)	0.19(0.12-0.29)	0.29(0.17-0.46)	1.5(0.59-2.42)	8.41(5.75-11.79)	10.62(7.29-14.61)	0.7(0.07-1.34)
Denmark	Female	1.02(0.54-1.75)	1.25(0.69-2.07)	1.57(1.26-1.88)	62.31(42.44-89.26)	83.1(55.76-122.03)	1.6(1.37-1.84)	0.17(0.1-0.25)	0.23(0.14-0.37)	1.07(0.37-1.77)	7.31(4.94-10.23)	8.52(5.87-11.8)	0.29(-0.16-0.73)
Denmark	Male	2.04(1.22-3.21)	2.56(1.62-3.82)	1.5(1.26-1.73)	70.32(48.75-101.01)	87.12(61.5-122.64)	1.33(1.13-1.54)	0.22(0.13-0.35)	0.35(0.21-0.58)	1.78(0.68-2.89)	9.66(6.47-13.72)	12.82(8.71-18.21)	0.97(0.19-1.75)
Djibouti	Both	1.03(0.48-2.18)	1.33(0.64-2.82)	1.32(1.1-1.53)	45.12(31.42-65.49)	57.4(39.6-84.57)	1.32(1.12-1.52)	1.02(0.57-1.71)	1.2(0.68-1.98)	0.53(0.39-0.68)	34.45(20.55-55.77)	40.83(23.77-67.24)	0.56(0.42-0.71)
Djibouti	Female	0.61(0.26-1.35)	0.85(0.37-1.86)	1.59(1.31-1.87)	44.99(28.64-68.02)	61.26(38.5-92.35)	1.46(1.27-1.65)	0.86(0.48-1.59)	1.05(0.57-1.86)	0.72(0.62-0.82)	29.78(17.27-52.66)	35.97(20.05-62.06)	0.68(0.58-0.78)
Djibouti	Male	1.39(0.63-3.01)	1.73(0.79-3.62)	1.15(0.96-1.34)	45.25(29.51-70.03)	53.44(34.69-83.38)	1.13(0.93-1.33)	1.17(0.64-1.96)	1.33(0.76-2.2)	0.36(0.18-0.53)	38.74(22.55-62.39)	45(26.8-74.61)	0.46(0.28-0.64)
Dominica	Both	1.48(0.58-3.45)	2.15(0.83-5.05)	1.41(1.28-1.54)	36.88(23.54-62.6)	50.54(30.64-89.05)	1.35(1.24-1.46)	2.08(1.37-3)	2.73(1.69-4.13)	1.09(0.99-1.2)	77.42(53.14-108.96)	102.76(66.57-150.5)	1.1(0.97-1.22)
Dominica	Female	1.12(0.34-2.85)	1.72(0.54-4.44)	1.61(1.49-1.73)	33.01(20.07-55.24)	48.84(28.83-85.5)	1.68(1.57-1.8)	1.83(1.21-2.64)	2.47(1.53-3.68)	1.3(1.14-1.46)	69.63(47.52-98.28)	95.36(62.31-138.65)	1.24(1.07-1.41)
Dominica	Male	1.85(0.75-4.36)	2.55(0.98-6.08)	1.23(1.1-1.37)	41(25.28-70.58)	52.12(29.94-95.92)	1.03(0.91-1.14)	2.43(1.56-3.61)	2.97(1.8-4.53)	0.75(0.67-0.83)	87.92(57.81-126.26)	109.6(69.65-162.24)	0.83(0.75-0.92)
Dominican Republic	Both	1.13(0.52-2.32)	1.79(0.81-3.66)	1.62(1.49-1.75)	39.56(27.04-58.23)	51.69(34.59-78.58)	1.08(0.96-1.21)	0.81(0.52-1.17)	1.39(0.81-2.36)	2.75(2.43-3.08)	32.97(22.09-46.7)	54.06(33.16-87.5)	2.52(2.24-2.81)
Dominican Republic	Female	0.77(0.27-1.79)	1.29(0.47-2.97)	1.76(1.65-1.87)	35.97(22.83-55.83)	48.58(30.34-79.76)	1.17(1.06-1.28)	0.7(0.45-1.02)	1.22(0.7-2.02)	2.69(2.38-3)	28.93(19.39-41.26)	48.44(29.04-75.87)	2.5(2.21-2.79)
Dominican Republic	Male	1.5(0.69-3)	2.29(1.08-4.63)	1.5(1.36-1.63)	43.2(28.31-67.87)	54.87(35.13-88.05)	1.01(0.88-1.14)	0.92(0.59-1.36)	1.56(0.84-2.78)	2.79(2.42-3.16)	37.11(24.46-53.04)	59.8(33.97-100.57)	2.53(2.21-2.85)
Ecuador	Both	1.2(0.55-2.41)	1.98(0.93-3.96)	2.02(1.91-2.14)	38.77(25.39-58.45)	53.56(34.41-85.1)	1.62(1.41-1.82)	1.01(0.65-1.42)	2.01(1.16-3.23)	2.43(1.66-3.21)	37.14(25.16-51.43)	66.7(40.93-102.78)	1.93(1.14-2.72)
Ecuador	Female	0.91(0.35-2)	1.52(0.63-3.36)	2.11(1.97-2.26)	38.55(23.43-59.94)	54.16(32.68-85.74)	1.73(1.48-1.98)	1(0.65-1.45)	1.92(1.1-3.06)	2.28(1.55-3.01)	37.08(25.04-51.89)	63.83(38.79-99.2)	1.75(1.01-2.48)
Ecuador	Male	1.48(0.65-3.03)	2.44(1.13-4.81)	1.97(1.87-2.08)	38.89(23.41-61.97)	52.9(31.79-88.47)	1.5(1.34-1.67)	1.02(0.65-1.46)	2.1(1.2-3.37)	2.59(1.77-3.41)	37.18(24.95-51.79)	69.74(42.24-108.62)	2.11(1.26-2.97)
Egypt	Both	1.85(1.07-3.27)	2.95(1.69-5.34)	1.74(1.63-1.84)	59.27(41.53-84.59)	82.78(56.49-123.1)	1.26(1.17-1.36)	1.08(0.62-1.71)	1.22(0.61-2.13)	0.61(0.53-0.69)	38.4(23.64-57.59)	42.76(23.04-72.62)	0.55(0.48-0.62)
Egypt	Female	1.47(0.67-3.03)	2.35(1.06-4.74)	1.71(1.63-1.79)	58.15(37.79-88.27)	87.65(55.14-134.88)	1.44(1.38-1.49)	1.11(0.54-1.79)	1.25(0.49-2.29)	0.55(0.46-0.64)	39.88(20.28-61.87)	42.5(18.67-73.71)	0.32(0.23-0.41)
Egypt	Male	2.23(1.26-3.85)	3.49(1.95-6.26)	1.7(1.56-1.84)	60.49(41.03-88.93)	77.54(50.82-120.73)	1.05(0.91-1.19)	1.05(0.62-1.75)	1.2(0.6-2.28)	0.73(0.63-0.83)	36.96(22.36-58.27)	43.12(23.28-77.52)	0.78(0.69-0.87)

El Salvador	Both	1.29(0.53-2.7)	2.2(0.8-4.69)	2.01(1.96-2.06)	35.57(23.25-54.87)	48.36(29.33-79.91)	1.41(1.31-1.52)	1.38(0.88-1.98)	4.62(2.76-7.4)	4.47(3.73-5.2)	52.53(35.63-72.89)	169(104.46-266.46)	4.39(3.7-5.08)
El Salvador	Female	0.81(0.31-1.94)	1.29(0.44-3.19)	2(1.87-2.13)	33.94(21.36-52.67)	43.3(25.77-71.35)	1.32(1.14-1.51)	1.05(0.66-1.52)	2.65(1.54-4.27)	3.37(2.84-3.91)	39.63(26.6-55.42)	93.9(56.67-148.58)	3.19(2.66-3.73)
El Salvador	Male	1.78(0.73-3.71)	3.17(1.17-6.82)	2.05(1.97-2.13)	37.1(21.77-60.91)	53.67(29.89-93.34)	1.5(1.43-1.58)	1.75(1.11-2.53)	7.27(4.37-11.61)	5.28(4.43-6.14)	66.95(43.87-94.33)	268.83(165.27-419)	5.24(4.45-6.03)
Equatorial Guinea	Both	0.79(0.31-1.79)	1.2(0.51-2.61)	1.98(1.76-2.19)	33.54(22.54-51.01)	45.12(29.71-69.75)	1.48(1.32-1.65)	1.54(0.87-2.49)	1.35(0.75-2.32)	-0.32(-0.52--0.11)	54.35(31.84-85.77)	45.95(25.57-77.32)	-0.46(-0.67--0.25)
Equatorial Guinea	Female	0.54(0.19-1.32)	0.9(0.34-2.08)	2.24(2.04-2.44)	35.96(22.75-56.97)	49.95(31.31-78.6)	1.52(1.38-1.66)	1.15(0.55-2)	1.21(0.59-2.21)	0.49(0.27-0.7)	42.85(21.06-70.95)	41.96(20.54-77.28)	0.25(0.02-0.48)
Equatorial Guinea	Male	1.05(0.4-2.44)	1.47(0.63-3.29)	1.7(1.48-1.93)	30.22(18.04-50.14)	40.22(23.95-68.07)	1.56(1.34-1.78)	2.02(1.12-3.37)	1.56(0.84-2.59)	-1.02(-1.24--0.8)	68.47(38.85-111.4)	51.06(28.02-85.77)	-1.13(-1.35--0.9)
Eritrea	Both	0.92(0.4-1.94)	1.18(0.54-2.45)	1.11(0.99-1.23)	39.52(28.24-56.01)	49.89(35.37-72.59)	1.24(1.08-1.41)	1.34(0.74-2.28)	1.34(0.73-2.16)	-0.09(-0.24-0.05)	47.24(27.34-78.98)	46.8(26.45-76.84)	-0.09(-0.21-0.04)
Eritrea	Female	0.52(0.2-1.22)	0.73(0.29-1.64)	1.41(1.24-1.58)	39.25(25.85-58.57)	52.15(34.01-79.8)	1.34(1.19-1.49)	1.03(0.49-2.09)	1.13(0.6-2.08)	0.47(0.35-0.58)	37.37(18.54-73.27)	39.49(21.97-71.73)	0.37(0.26-0.47)
Eritrea	Male	1.32(0.57-2.83)	1.63(0.73-3.45)	0.99(0.89-1.1)	39.57(25.53-60.63)	47.59(30.48-72.85)	1.15(0.98-1.33)	1.78(0.89-3.15)	1.6(0.77-2.72)	-0.6(-0.81--0.38)	59.94(32.86-99.94)	55.34(28-93.69)	-0.49(-0.7--0.29)
Estonia	Both	1.48(0.72-2.94)	2.54(1.35-4.64)	2.46(2.05-2.88)	83.02(57.19-119.49)	122.89(86.87-172.64)	1.92(1.57-2.28)	0.21(0.13-0.3)	0.8(0.48-1.25)	5.01(4.19-5.84)	7.38(5.1-10.21)	21.43(14.15-30.46)	3.6(2.88-4.32)
Estonia	Female	1.22(0.51-2.65)	1.89(0.85-3.93)	2.02(1.59-2.46)	86.92(57.06-130.39)	127.7(83.64-191.36)	1.95(1.57-2.32)	0.17(0.1-0.25)	0.73(0.42-1.14)	5.55(4.83-6.27)	5.85(4.04-8.19)	17.95(11.68-25.88)	3.94(3.38-4.5)
Estonia	Male	1.77(0.84-3.55)	3.24(1.68-6.05)	2.75(2.36-3.15)	79.6(50.89-122.79)	119.79(79.71-175.76)	1.93(1.59-2.27)	0.27(0.17-0.39)	0.86(0.51-1.33)	4.32(3.36-5.29)	9.39(6.5-12.88)	25.09(16.67-35.79)	3.26(2.39-4.13)
Ethiopia	Both	0.93(0.62-1.42)	1.24(0.88-1.84)	1.53(1.31-1.75)	39.65(32.31-49.21)	66.59(54.02-81.88)	2.57(2.33-2.82)	2.2(1.39-3.33)	1.05(0.69-1.53)	-2.63(-2.84--2.42)	78.33(50.9-114.58)	36.24(25.03-49.6)	-2.73(-2.96--2.49)
Ethiopia	Female	0.65(0.4-1.04)	0.88(0.58-1.35)	1.54(1.3-1.77)	38.74(30.35-49.42)	71.4(56.05-90.76)	2.87(2.61-3.12)	2.08(1.28-3.21)	0.94(0.6-1.4)	-3.03(-3.33--2.73)	77.72(48.94-117.18)	33.08(22.36-48.14)	-3.24(-3.56--2.91)
Ethiopia	Male	1.2(0.81-1.85)	1.58(1.11-2.36)	1.53(1.31-1.75)	40.51(31.78-51.74)	61.53(48.42-76.92)	2.26(2.01-2.51)	2.29(1.29-3.69)	1.16(0.73-1.75)	-2.25(-2.39--2.1)	77.85(44.82-122.12)	39.28(26.27-55.26)	-2.21(-2.39--2.04)
Fiji	Both	1.11(0.32-2.75)	1.37(0.41-3.38)	0.8(0.74-0.85)	31.89(18.91-52.22)	36.62(21.21-61.88)	0.61(0.54-0.67)	3.25(2.09-4.76)	3.94(2.55-5.8)	0.18(-0.18-0.55)	130.34(87.39-186.41)	156.22(105.13-225.1)	0.15(-0.2-0.5)
Fiji	Female	0.88(0.22-2.31)	1.08(0.27-2.8)	0.75(0.69-0.81)	29.31(17.38-47.48)	34.02(19.43-56.71)	0.62(0.55-0.7)	2.83(1.73-4.36)	3.4(2.17-5.08)	0.23(-0.12-0.59)	116.56(73.81-175.13)	138.49(92.08-200.23)	0.16(-0.2-0.52)
Fiji	Male	1.33(0.41-3.29)	1.65(0.5-4.08)	0.83(0.77-0.89)	34.38(20.03-58.83)	39.11(21.8-70.04)	0.59(0.53-0.65)	3.67(2.39-5.53)	4.53(2.92-6.57)	0.17(-0.23-0.56)	144.08(97.29-211.53)	174.42(117.25-251.73)	0.14(-0.22-0.5)
Finland	Both	1.19(0.74-1.78)	1.3(0.86-1.91)	0.7(0.25-1.14)	74.49(51.35-106.75)	69.11(51.41-91.58)	0.39(-0.15-0.94)	0.13(0.09-0.2)	0.14(0.09-0.22)	3.45(2.08-4.85)	6.3(4.46-8.57)	6.1(4.32-8.29)	1.65(0.72-2.6)
Finland	Female	0.96(0.51-1.66)	1.05(0.56-1.8)	0.22(0.03-0.41)	81.5(52.37-123.87)	73.55(50.09-104.7)	-0.08(-0.43-0.28)	0.11(0.07-0.16)	0.13(0.08-0.2)	3.79(2.51-5.08)	5.23(3.71-7.15)	5.41(3.8-7.38)	1.55(0.83-2.28)
Finland	Male	1.46(0.86-2.36)	1.56(0.95-2.5)	0.96(0.32-1.6)	68.21(44.45-102.54)	65.34(45.81-92.67)	0.9(0.14-1.66)	0.16(0.1-0.25)	0.16(0.1-0.24)	3.17(1.65-4.72)	7.5(5.2-10.28)	6.82(4.72-9.46)	1.71(0.6-2.83)
France	Both	1.11(0.65-1.88)	2(1.33-3.02)	3(2.58-3.42)	57.78(39.76-83.55)	98.54(70.63-135.82)	2.88(2.39-3.38)	0.15(0.09-0.24)	0.11(0.07-0.19)	-0.86(-0.98--0.74)	5.95(4.11-8.52)	6.3(4.25-8.88)	0.61(0.43-0.8)
France	Female	0.86(0.42-1.62)	1.56(0.8-2.75)	2.83(2.5-3.17)	60.61(37.62-93.49)	104.91(67.83-159.36)	2.74(2.31-3.17)	0.12(0.07-0.19)	0.09(0.05-0.15)	-1(-1.28--0.73)	4.9(3.35-7.01)	5.55(3.62-8.12)	0.66(0.39-0.93)
France	Male	1.37(0.76-2.33)	2.49(1.54-3.83)	3.13(2.65-3.62)	55.09(36.44-85.12)	92.35(64.64-134.26)	3.04(2.47-3.62)	0.2(0.12-0.31)	0.14(0.08-0.23)	-0.85(-0.94--0.76)	7.18(4.88-10.36)	7.15(4.85-10.44)	0.54(0.37-0.71)
Gabon	Both	0.99(0.39-2.29)	1.35(0.56-3.03)	1.35(1.2-1.5)	35.51(23.72-53.99)	45.96(30.4-69.5)	1.3(1.12-1.48)	1.8(1.07-2.88)	1.81(0.94-3.06)	-0.06(-0.34-0.22)	63.36(38.5-97.39)	61.9(33.12-100.77)	-0.15(-0.44-0.14)
Gabon	Female	0.67(0.24-1.7)	1.02(0.38-2.46)	1.72(1.51-1.94)	38.41(24.65-59.31)	51.35(33.06-78.31)	1.38(1.17-1.59)	1.33(0.55-2.27)	1.53(0.47-2.74)	0.43(-0.05-0.91)	47.91(20.56-79.28)	53.5(18.13-94.45)	0.31(-0.18-0.8)
Gabon	Male	1.31(0.48-3.1)	1.69(0.65-3.91)	1.15(1.03-1.27)	32.57(19.47-54.99)	39.98(24.04-68.68)	1.17(1.01-1.32)	2.37(1.35-3.77)	2.11(1.25-3.5)	-0.47(-0.63--0.32)	80.45(46.45-123.51)	70.91(42.45-113.05)	-0.52(-0.67--0.37)
Gambia	Both	1.43(0.65-2.99)	1.78(0.78-3.81)	0.99(0.87-1.1)	56.45(37.91-83.37)	62.11(41.61-93.99)	0.71(0.57-0.85)	1.4(0.82-2.27)	1.53(0.91-2.41)	0.25(0.05-0.45)	49.24(29.54-78.23)	54.55(33.38-84.8)	0.33(0.12-0.54)
Gambia	Female	1.12(0.46-2.48)	1.33(0.54-2.97)	0.82(0.66-0.97)	55.44(34.84-84.19)	61.37(38.1-95.46)	0.65(0.5-0.8)	1.24(0.69-2.13)	1.34(0.79-2.14)	0.13(-0.09-0.36)	44.97(25.58-74.93)	48.85(29.03-74.83)	0.19(-0.04-0.43)
Gambia	Male	1.74(0.72-3.79)	2.24(0.91-4.99)	1.12(1.01-1.22)	57.44(35.89-91.23)	62.62(38.92-101.03)	0.76(0.61-0.91)	1.54(0.91-2.45)	1.72(1.02-2.8)	0.4(0.22-0.58)	53.05(32.35-85.06)	60.52(36.43-97.22)	0.48(0.29-0.68)
Germany	Both	0.88(0.46-1.67)	1.28(0.8-2.11)	2.13(1.73-2.53)	33.44(22.41-48.31)	49.23(35.11-67.19)	2.22(1.83-2.62)	0.2(0.12-0.3)	0.23(0.13-0.38)	1.55(0.84-2.27)	7.25(4.8-10.47)	7.29(4.91-10.32)	0.73(0.22-1.25)
Germany	Female	0.6(0.28-1.19)	0.92(0.47-1.62)	2.21(1.82-2.61)	32.65(21.12-48.6)	50.25(33.39-74.09)	2.28(1.89-2.67)	0.17(0.1-0.27)	0.2(0.11-0.34)	1.85(1.14-2.56)	5.96(3.95-8.66)	6.11(4.12-8.68)	0.94(0.43-1.45)
Germany	Male	1.16(0.58-2.28)	1.65(0.96-2.9)	2.06(1.66-2.47)	34.48(22.23-54.09)	48.6(33.75-71.68)	2.15(1.75-2.56)	0.23(0.14-0.35)	0.26(0.15-0.43)	1.26(0.53-2)	8.59(5.5-12.47)	8.49(5.63-12.08)	0.58(0.05-1.11)
Ghana	Both	1.24(0.57-2.67)	1.63(0.74-3.52)	1.09(0.99-1.19)	50.18(33.99-75.52)	56.93(37.94-89.17)	0.8(0.62-0.97)	1.51(0.91-2.45)	1.74(1.02-2.66)	0.79(0.6-0.98)	55.94(34.01-87.58)	64.07(38.46-94.91)	0.79(0.6-0.98)
Ghana	Female	1.09(0.41-2.65)	1.41(0.55-3.41)	1(0.86-1.13)	48.78(31.13-77.33)	60.19(38.42-96.76)	1.04(0.83-1.25)	1.81(1.03-3.08)	1.72(1.02-2.59)	-0.13(-0.25--0.02)	69.2(39.37-115.69)	65.33(39.4-98.95)	-0.12(-0.25-0.01)
Ghana	Male	1.38(0.61-3.04)	1.85(0.79-4.2)	1.18(1.09-1.28)	51.61(31.6-82.17)	53.16(32.6-88.28)	0.53(0.37-0.69)	1.2(0.71-1.94)	1.77(0.93-2.79)	1.97(1.66-2.28)	42.11(25.73-65.86)	62.92(34.18-95.98)	2.01(1.72-2.31)
Greece	Both	1.18(0.7-2.04)	1.64(1.07-2.58)	2.08(1.71-2.45)	58.87(37.98-90.84)	81.38(55.47-119.38)	1.95(1.64-2.26)	0.35(0.2-0.56)	0.24(0.14-0.4)	-4.57(-5.95--3.17)	11.15(7.39-16)	9.29(6.23-13.26)	-2.09(-2.86--1.31)
Greece	Female	0.82(0.42-1.54)	1.23(0.65-2.08)	2.31(1.94-2.69)	58.86(35.36-97.38)	87.1(52.77-142.78)	2.15(1.85-2.44)	0.33(0.19-0.54)	0.2(0.12-0.34)	-5.08(-6.51--3.64)	9.79(6.44-14.5)	7.58(5.08-10.92)	-2.17(-3.02--1.31)
Greece	Male	1.53(0.87-2.71)	2.07(1.26-3.28)	1.97(1.59-2.35)	58.98(36.9-91.47)	75.81(49.08-111.49)	1.74(1.42-2.07)	0.37(0.22-0.59)	0.29(0.17-0.47)	-4.15(-5.51--2.78)	12.54(8.19-17.99)	11.12(7.32-16.13)	-2(-2.73--1.27)
Greenland	Both	2.39(1.59-3.5)	2.02(1.32-3.08)	-0.1(-0.27-0.07)	158.3(115.82-220.79)	122.28(87.63-172.95)	-0.45(-0.63--0.27)	0.24(0.14-0.38)	0.18(0.1-0.31)	-1.01(-1.17--0.85)	11.07(7.4-15.32)	8.3(5.47-12.1)	-0.85(-0.97--0.73)

Greenland	Female	2.36(1.35-3.83)	1.69(0.96-2.77)	-0.54(-0.78--0.3)	213.09(140.48-315.02)	153.07(100.23-231.76)	-0.64(-0.86--0.42)	0.2(0.12-0.33)	0.15(0.08-0.25)	-1.62(-1.92--1.31)	10.16(6.72-14.28)	7.26(4.73-10.5)	-1.2(-1.3--1.09)
Greenland	Male	2.47(1.49-3.91)	2.32(1.41-3.75)	0.14(0.01-0.28)	116.73(80.04-167.24)	94.53(64.07-140.12)	-0.35(-0.5--0.2)	0.27(0.15-0.43)	0.21(0.11-0.35)	-0.46(-0.74--0.17)	11.8(7.68-16.89)	9.17(5.93-13.75)	-0.56(-0.75--0.37)
Grenada	Both	1.2(0.47-2.75)	2.01(0.82-4.5)	1.84(1.73-1.95)	31.33(20.37-51.2)	47.16(30.04-79.25)	1.64(1.54-1.74)	2.2(1.47-3.16)	2.84(1.87-4.1)	1.4(1.19-1.6)	82.72(56.87-115.28)	104.09(69.19-145.56)	1.33(1.12-1.55)
Grenada	Female	0.85(0.27-2.11)	1.45(0.47-3.53)	1.82(1.71-1.93)	26.76(16.75-43.94)	41.59(25.95-68.02)	1.73(1.63-1.83)	1.71(1.13-2.46)	2.39(1.56-3.51)	1.77(1.44-2.09)	65.9(45.58-93.77)	89.51(60.07-126.08)	1.63(1.33-1.93)
Grenada	Male	1.57(0.62-3.43)	2.53(1.02-5.62)	1.78(1.67-1.88)	36.16(21.85-60.89)	52.45(31.66-90.91)	1.52(1.41-1.62)	2.8(1.83-4.06)	3.3(2.16-4.82)	1.03(0.81-1.25)	102.05(68.25-141.65)	118.36(78.11-164.93)	1.02(0.82-1.22)
Grenadines	Both	1.08(0.4-2.43)	1.67(0.64-3.81)	1.65(1.55-1.76)	29.17(18.29-47.8)	41.53(25.8-69.34)	1.47(1.37-1.57)	1.57(1.04-2.2)	2.31(1.48-3.33)	1.53(1.39-1.67)	59.69(40.77-80.73)	89.21(59.86-125)	1.54(1.43-1.64)
Grenadines	Female	0.84(0.25-2.24)	1.31(0.4-3.38)	1.63(1.53-1.72)	26.05(15.33-44)	38.59(22.67-66.03)	1.59(1.49-1.69)	1.42(0.95-2.03)	2.15(1.37-3.15)	1.57(1.4-1.75)	54.38(37.45-74.97)	83.66(55.2-120.38)	1.53(1.41-1.65)
Grenadines	Male	1.32(0.51-2.9)	2(0.79-4.4)	1.64(1.53-1.74)	32.39(19.46-54.24)	44.26(26.5-75.67)	1.34(1.24-1.45)	1.72(1.14-2.41)	2.47(1.58-3.52)	1.43(1.29-1.57)	65.34(44.14-88.7)	94.33(62.19-132.65)	1.5(1.38-1.62)
Guam	Both	1.02(0.35-2.46)	1.52(0.51-3.7)	1.36(1.25-1.47)	34.18(21.99-53.53)	43.26(26.92-72.22)	0.96(0.86-1.06)	1.62(1.09-2.28)	2.9(1.98-4.12)	2.39(2.25-2.54)	62.39(43.66-84.34)	120.54(86.12-165.64)	2.65(2.53-2.76)
Guam	Female	0.85(0.23-2.25)	1.27(0.34-3.28)	1.41(1.3-1.52)	32.99(20.63-51.23)	42.81(26.19-68.99)	1.06(0.95-1.17)	1.78(1.17-2.56)	2.77(1.85-4.05)	1.73(1.57-1.89)	67.4(45.86-93.01)	113.3(78.74-158.99)	2(1.88-2.11)
Guam	Male	1.19(0.42-2.93)	1.76(0.6-4.29)	1.33(1.21-1.45)	35.34(22.34-58.08)	43.77(25.96-78.24)	0.87(0.77-0.96)	1.48(0.98-2.13)	3.02(2.04-4.26)	3.04(2.87-3.21)	58.03(40.32-80.61)	127.22(89.87-175.52)	3.26(3.1-3.42)
Guatemala	Both	1.33(0.54-3.16)	1.9(0.69-4.73)	1.28(1.24-1.32)	34.88(22.19-58.07)	42.59(24.83-76.83)	0.85(0.8-0.91)	1.72(1.11-2.48)	3.32(1.99-5.13)	3.18(2.64-3.72)	63.62(42.94-87.77)	118.4(73.16-180.51)	2.97(2.47-3.47)
Guatemala	Female	0.85(0.28-2.05)	1.22(0.38-3)	1.31(1.23-1.4)	32.09(19.81-52.89)	37.62(21.76-63.52)	0.73(0.67-0.8)	1.72(1.11-2.45)	3.13(1.84-4.92)	3.08(2.43-3.74)	64.32(42.19-89.75)	110.37(67.13-170.2)	2.79(2.17-3.41)
Guatemala	Male	1.82(0.72-4.3)	2.6(0.93-6.44)	1.29(1.26-1.32)	37.6(21.83-67.32)	47.66(25.64-93.76)	0.96(0.9-1.03)	1.73(1.09-2.58)	3.55(2.12-5.57)	3.32(2.86-3.79)	62.78(41.46-88.69)	128.28(80.05-193.67)	3.21(2.78-3.64)
Guinea	Both	1.51(0.68-3.13)	1.76(0.76-3.8)	0.82(0.67-0.96)	58.19(39.25-86.66)	59.75(39.55-90.07)	0.55(0.37-0.73)	1.74(1.07-2.76)	1.72(1.03-2.63)	0.02(-0.1-0.14)	62.91(39.39-98.74)	62.99(38.7-94.93)	0.13(0-0.25)
Guinea	Female	1.23(0.49-2.72)	1.35(0.52-3.01)	0.59(0.44-0.74)	57.15(35.28-85.22)	60.02(36.31-92.81)	0.57(0.39-0.74)	1.95(1.15-3.31)	1.61(0.94-2.57)	-0.48(-0.69--0.28)	72.01(43.04-119.98)	60.4(35.35-95.07)	-0.35(-0.56--0.14)
Guinea	Male	1.79(0.76-3.93)	2.17(0.91-4.82)	0.97(0.83-1.11)	59.21(36.48-93.81)	58.84(35.47-95.67)	0.51(0.31-0.7)	1.51(0.86-2.53)	1.82(1.06-2.91)	0.54(0.45-0.63)	52.9(31.24-87.28)	65.33(39.55-100.21)	0.67(0.59-0.74)
Guinea-Bissau	Both	1.57(0.68-3.35)	1.81(0.77-3.89)	0.76(0.62-0.9)	49.86(33.07-76.58)	55.63(36.85-87.12)	0.79(0.63-0.96)	3.03(1.84-4.83)	2.45(1.47-3.74)	-0.6(-0.69--0.52)	111.35(68.33-174.27)	91.38(56.81-136.45)	-0.52(-0.62--0.42)
Guinea-Bissau	Female	1.18(0.45-2.64)	1.32(0.5-3.02)	0.64(0.48-0.8)	47.64(30.93-73.73)	53.86(34.71-84.75)	0.76(0.59-0.93)	2.7(1.58-4.44)	2.14(1.23-3.37)	-0.71(-0.82--0.6)	104.77(60.61-170.51)	82.67(48.44-128.14)	-0.68(-0.8--0.55)
Guinea-Bissau	Male	1.98(0.8-4.22)	2.31(0.92-5.01)	0.85(0.72-0.98)	52.24(31.77-83.04)	57.39(34.68-91.36)	0.82(0.66-0.99)	3.34(1.93-5.4)	2.82(1.69-4.43)	-0.45(-0.53--0.37)	117.31(69.42-187.77)	101.31(63.27-152.37)	-0.34(-0.43--0.26)
Guyana	Both	1.09(0.4-2.67)	1.7(0.65-3.89)	1.57(1.53-1.61)	28.43(17.65-48.69)	40.04(24.52-69.62)	1.38(1.31-1.46)	1.86(1.18-2.72)	3.23(1.95-5.06)	2.67(2.4-2.95)	70.08(46.97-99.42)	122.07(75.43-188.36)	2.6(2.32-2.87)
Guyana	Female	0.8(0.24-2.11)	1.29(0.4-3.34)	1.66(1.62-1.7)	24.83(15.05-42.22)	36.33(21.61-62.88)	1.52(1.43-1.6)	1.61(1.01-2.41)	2.97(1.75-4.72)	2.87(2.58-3.16)	60.88(39.44-88.82)	113.64(70.37-176.46)	2.81(2.52-3.1)
Guyana	Male	1.39(0.53-3.22)	2.12(0.83-4.79)	1.52(1.48-1.56)	32.09(19.05-56.52)	43.8(25.83-79.65)	1.27(1.2-1.34)	2.13(1.35-3.13)	3.51(2.12-5.44)	2.52(2.24-2.79)	79.57(52.78-114.45)	131.07(81.31-199.83)	2.42(2.15-2.7)
Haiti	Both	0.99(0.37-2.39)	1.38(0.55-3.26)	1.26(1.22-1.3)	27.15(17.56-45.2)	36.9(23.48-61.03)	1.34(1.26-1.43)	2.3(1.38-3.69)	2.1(1.13-3.7)	0.04(-0.15-0.24)	87.86(55.05-137.73)	78.4(45.22-132.82)	-0.01(-0.21-0.19)
Haiti	Female	0.83(0.23-2.17)	1.18(0.35-3.08)	1.26(1.23-1.29)	24.68(14.73-42.56)	34.78(20.4-58.82)	1.42(1.34-1.49)	2.18(1.28-3.81)	2.05(1.06-3.9)	0.18(0-0.35)	87.42(51.72-152.41)	79.03(43.16-146.5)	0.04(-0.14-0.22)
Haiti	Male	1.16(0.46-2.57)	1.61(0.67-3.41)	1.31(1.25-1.37)	29.73(18.84-51.05)	39.37(24.69-65.28)	1.31(1.2-1.41)	2.43(1.36-3.97)	2.14(1.1-3.9)	-0.09(-0.35-0.18)	87.73(52.72-138.98)	77.65(43.19-134.52)	-0.06(-0.33-0.22)
Honduras	Both	1.13(0.42-2.45)	1.49(0.55-3.26)	1.03(0.98-1.08)	30.02(18.24-48.54)	36.6(21.86-60.94)	0.9(0.82-0.99)	1.64(1.01-2.56)	3.1(1.73-5.03)	2.52(2.22-2.82)	61.59(40.28-90.09)	101.35(56.71-165.16)	1.94(1.63-2.26)
Honduras	Female	0.87(0.28-2.1)	1.12(0.37-2.78)	1.01(0.93-1.08)	30.19(18.16-47.42)	35.91(20.98-57.3)	0.87(0.75-0.99)	1.66(1.04-2.45)	3.37(1.83-5.5)	2.7(2.28-3.13)	64.48(41.89-92.41)	113.01(62.69-179.58)	2.12(1.71-2.53)
Honduras	Male	1.39(0.52-3.1)	1.87(0.7-4.16)	1.06(1-1.12)	29.68(16.54-52.08)	36.94(20-67.62)	0.92(0.85-0.98)	1.62(0.92-2.86)	2.79(1.55-4.62)	2.28(2.08-2.47)	58.47(34.64-96.59)	88.1(48.67-144.07)	1.69(1.48-1.91)
Hungary	Both	0.84(0.38-1.7)	1.51(0.8-2.85)	2.47(2.3-2.65)	41.57(28.53-60.24)	70.11(48.31-102.54)	2.33(2.18-2.48)	0.33(0.22-0.48)	0.33(0.2-0.51)	0.11(-1.2-1.43)	13.37(9.01-18.62)	12.29(8.16-17.59)	-0.17(-1.06-0.73)
Hungary	Female	0.63(0.24-1.45)	1.26(0.56-2.75)	2.81(2.63-2.98)	43.8(28.02-68.13)	82.01(51.62-127.51)	2.6(2.45-2.75)	0.29(0.19-0.43)	0.31(0.19-0.48)	0.49(-0.71-1.7)	11.29(7.73-15.64)	11.14(7.53-15.72)	0.22(-0.52-0.97)
Hungary	Male	1.07(0.47-2.24)	1.77(0.9-3.48)	2.28(2.1-2.46)	39.44(25.54-61.34)	58.73(38.84-88.19)	2.01(1.85-2.17)	0.38(0.24-0.56)	0.35(0.21-0.56)	-0.3(-1.75-1.17)	15.61(10.38-21.98)	13.49(8.73-19.77)	-0.5(-1.53-0.54)
Iceland	Both	1.08(0.66-1.72)	1.1(0.68-1.8)	0.52(0.34-0.71)	51.4(36.55-69.77)	56.91(40.04-78.2)	0.79(0.64-0.94)	0.08(0.05-0.13)	0.08(0.05-0.14)	1.66(0.55-2.78)	3.79(2.62-5.35)	3.9(2.67-5.56)	0.9(0.26-1.54)
Iceland	Female	0.79(0.41-1.41)	0.79(0.42-1.46)	0.45(0.22-0.68)	52.05(33.71-76.69)	60.02(39.43-89.94)	0.93(0.78-1.08)	0.06(0.04-0.11)	0.06(0.04-0.1)	1.25(0.15-2.37)	2.98(1.99-4.3)	3.11(2.05-4.48)	0.85(0.24-1.46)
Iceland	Male	1.37(0.79-2.29)	1.42(0.8-2.4)	0.55(0.35-0.74)	50.95(33.93-76.45)	54.27(36.52-78.92)	0.65(0.47-0.84)	0.1(0.06-0.16)	0.11(0.06-0.19)	1.97(0.86-3.09)	4.64(3.16-6.48)	4.71(3.16-6.72)	0.94(0.26-1.62)
India	Both	1.18(0.97-1.45)	1.27(1.03-1.57)	0.81(0.57-1.06)	48.42(42.28-56.21)	55.36(48.44-64.21)	1.13(0.88-1.37)	1.11(0.67-1.66)	1.24(0.73-1.94)	0.41(0.19-0.63)	39.92(25.04-59.91)	44.56(27.47-67.69)	0.47(0.25-0.7)
India	Female	0.82(0.64-1.02)	0.86(0.68-1.1)	0.73(0.47-0.98)	45.51(39.23-53.57)	55.06(47.12-65.34)	1.22(0.99-1.46)	0.87(0.52-1.35)	0.98(0.55-1.53)	0.27(0.11-0.44)	31.76(19.63-47.73)	34.61(20.56-53.2)	0.24(0.08-0.4)
India	Male	1.51(1.25-1.86)	1.65(1.35-2.03)	0.89(0.64-1.13)	51.03(44.3-59.94)	55.61(48.34-65.21)	1.05(0.79-1.31)	1.32(0.79-2.05)	1.51(0.88-2.38)	0.55(0.21-0.89)	47.3(28.73-71.54)	54.35(32.95-83.17)	0.67(0.32-1.01)
Indonesia	Both	1.09(0.8-1.49)	1.44(1.07-1.98)	1.27(1.01-1.52)	42.32(35.86-49.59)	53.43(44.94-63.38)	1.57(0.95-2.19)	2.41(1.73-3.27)	2.12(1.47-2.87)	-0.43(-0.54--0.32)	105.63(77.4-138.28)	89.51(64.95-119.38)	-0.54(-0.65--0.44)
Indonesia	Female	0.89(0.64-1.28)	1.14(0.84-1.61)	1.12(0.88-1.35)	42.45(35.18-51.18)	55.41(45.92-66.58)	1.68(1.01-2.34)	2.26(1.6-3.11)	1.74(1.15-2.48)	-0.94(-1.16--0.72)	99.93(71.06-135.66)	74.19(51.78-103.99)	-1.04(-1.25--0.83)

Indonesia	Male	1.29(0.95-1.77)	1.72(1.27-2.38)	1.35(1.08-1.62)	42.11(35.28-49.93)	51.45(42.42-61.53)	1.47(0.9-2.04)	2.57(1.82-3.48)	2.5(1.68-3.62)	-0.02(-0.06-0.02)	111.6(81.07-145.22)	104.8(72.07-147.01)	-0.14(-0.18--0.11)
Iran	Both	2.13(1.73-2.61)	3.19(2.58-3.82)	1.47(1.31-1.63)	61.27(53.23-71.36)	94.15(80.86-110.77)	1.59(1.31-1.86)	0.72(0.47-1.06)	0.53(0.35-0.76)	-1.24(-1.43--1.05)	26.72(18.16-37.36)	20.78(14.69-27.84)	-0.99(-1.18--0.81)
Iran	Female	1.71(1.34-2.17)	2.52(1.96-3.19)	1.43(1.24-1.62)	59.75(51.79-69.94)	96.13(81.01-114.66)	1.71(1.4-2.03)	0.6(0.38-0.88)	0.44(0.29-0.63)	-1.43(-1.69--1.17)	23.04(15.88-32.18)	17.43(12.52-23.12)	-1.26(-1.5--1.01)
Iran	Male	2.54(2.07-3.09)	3.86(3.15-4.66)	1.53(1.38-1.67)	62.64(53.91-73.45)	92.39(78.56-109.81)	1.48(1.25-1.72)	0.83(0.53-1.23)	0.62(0.4-0.9)	-1.05(-1.21--0.89)	30.03(19.98-42.88)	24.11(16.61-32.86)	-0.75(-0.92--0.59)
Iraq	Both	1.53(0.72-3.35)	2.8(1.47-5.6)	2.46(2.24-2.68)	38.45(24.31-63.21)	69.96(45.3-112.37)	2.43(2.2-2.66)	1.69(1.05-2.63)	1.33(0.78-2.17)	-0.96(-1.04--0.88)	61.49(38.34-94.36)	48.34(28.64-76.81)	-0.96(-1.05--0.87)
Iraq	Female	1.31(0.48-3.26)	2.19(0.92-5.15)	2.1(1.91-2.3)	37.33(21.93-64.06)	67.56(41.07-116.04)	2.35(2.11-2.59)	1.57(0.89-2.66)	1.09(0.6-1.77)	-1.4(-1.49--1.3)	59.76(34.31-100.86)	41.55(23.61-67.14)	-1.39(-1.5--1.27)
Iraq	Male	1.74(0.81-3.53)	3.4(1.71-6.35)	2.71(2.47-2.95)	39.55(23.94-68.06)	72.53(44.59-119.47)	2.52(2.29-2.75)	1.81(1.05-2.9)	1.59(0.91-2.6)	-0.61(-0.69--0.54)	63.43(37.64-99.21)	55.24(32.01-91.24)	-0.61(-0.69--0.52)
Ireland	Both	2.46(1.62-3.68)	2.47(1.6-3.88)	0.53(0.36-0.7)	92.63(69.4-127.35)	102.25(75.7-138.53)	0.83(0.69-0.97)	0.26(0.15-0.43)	0.18(0.1-0.29)	-1.49(-1.57--1.42)	10.03(6.84-13.91)	7.65(5.34-10.45)	-1.08(-1.24--0.92)
Ireland	Female	1.83(0.99-3.09)	1.61(0.9-2.75)	0.31(0.08-0.54)	90.42(61.96-129.64)	102.32(67.63-150.16)	0.88(0.76-1)	0.22(0.13-0.36)	0.15(0.08-0.25)	-1.53(-1.62--1.45)	8.69(5.85-12.32)	6.31(4.31-8.91)	-1.24(-1.4--1.08)
Ireland	Male	3.12(1.9-4.89)	3.34(2.04-5.47)	0.64(0.45-0.83)	95.08(65.41-138.14)	101.9(70.09-148.49)	0.77(0.59-0.94)	0.31(0.18-0.51)	0.21(0.12-0.35)	-1.5(-1.6--1.4)	11.51(7.65-16.3)	9.06(6.06-12.6)	-0.98(-1.18--0.78)
Israel	Both	1.34(0.84-2.12)	1.49(0.93-2.33)	1.1(0.8-1.39)	57.58(41.37-79.68)	59.52(42.93-81.16)	1.08(0.57-1.6)	0.65(0.4-1.06)	0.66(0.4-1.04)	4.37(2.79-5.98)	18.59(12.57-27.43)	18.69(12.55-26.84)	3.36(1.95-4.79)
Israel	Female	0.89(0.48-1.59)	0.95(0.52-1.67)	0.71(0.4-1.01)	55.12(36.25-81.12)	57.74(36.87-87.19)	0.82(0.27-1.37)	0.59(0.36-0.96)	0.51(0.3-0.82)	2.91(1.67-4.16)	16.35(10.94-23.95)	13.82(9.44-19.77)	1.99(0.91-3.08)
Israel	Male	1.81(1.06-2.99)	2.04(1.21-3.36)	1.26(0.95-1.56)	60.21(40.77-87.12)	61.63(42.68-89.27)	1.34(0.83-1.85)	0.73(0.43-1.17)	0.84(0.51-1.33)	5.42(3.58-7.28)	21.11(14.04-31.64)	23.94(15.76-34.71)	4.21(2.59-5.87)
Italy	Both	1.26(1.05-1.52)	1.42(1.17-1.72)	1.48(0.88-2.08)	69.78(60.45-80.76)	87.1(73.74-103.67)	1.94(1.04-2.85)	0.21(0.12-0.33)	0.13(0.08-0.2)	-2.02(-2.51--1.52)	8.49(5.77-11.99)	6.06(4.16-8.29)	-0.88(-1.19--0.56)
Italy	Female	0.89(0.72-1.1)	1.1(0.88-1.39)	1.97(1.22-2.73)	75.77(63.51-89.54)	103.71(85.12-126.92)	2.28(1.26-3.32)	0.18(0.11-0.28)	0.11(0.06-0.18)	-2.14(-2.77--1.52)	7.2(4.92-10.09)	5.32(3.65-7.23)	-0.76(-1.12--0.41)
Italy	Male	1.66(1.35-2.01)	1.75(1.41-2.13)	1.15(0.64-1.67)	63.95(54.9-75.21)	70.86(58.76-84.52)	1.47(0.74-2.2)	0.25(0.15-0.4)	0.15(0.09-0.24)	-1.96(-2.35--1.57)	9.96(6.71-14.2)	6.87(4.68-9.49)	-0.99(-1.28--0.71)
Ivory Coast	Both	1.65(0.71-3.69)	1.88(0.81-4.2)	0.8(0.62-0.98)	55.16(35.66-84.64)	59.13(37.48-92)	0.7(0.5-0.9)	1.85(1.13-2.83)	1.64(0.95-2.52)	-0.59(-0.83--0.35)	66.26(41.17-100.4)	59.84(34.9-91.05)	-0.48(-0.72--0.24)
Ivory Coast	Female	1.24(0.49-2.81)	1.39(0.53-3.18)	0.71(0.5-0.92)	53.36(34.04-82.35)	56.83(35.07-89.03)	0.57(0.35-0.78)	1.55(0.93-2.46)	1.37(0.78-2.18)	-0.31(-0.56--0.07)	57.35(34.86-90.72)	51.41(29.53-80.42)	-0.23(-0.48-0.03)
Ivory Coast	Male	2.04(0.81-4.68)	2.34(0.94-5.28)	0.82(0.66-0.99)	56.95(33.16-94.54)	61.08(35.85-101.74)	0.8(0.59-1)	2.11(1.27-3.25)	1.88(1.08-2.95)	-0.72(-0.98--0.47)	73.9(46-111.48)	67.36(39.21-104.4)	-0.63(-0.88--0.37)
Jamaica	Both	1.25(0.5-2.83)	1.69(0.71-3.8)	1.41(1.17-1.66)	35.28(22.9-58.22)	45.4(29.69-75.06)	1.28(1.04-1.51)	1.14(0.74-1.63)	2.02(1.23-3.1)	1.44(0.8-2.07)	41.36(27.52-57.09)	77.41(49.25-115.65)	1.63(0.99-2.27)
Jamaica	Female	0.85(0.28-2.17)	1.23(0.42-3.01)	1.51(1.35-1.68)	29.76(18.17-49.25)	40.5(24.68-67.87)	1.36(1.17-1.56)	0.96(0.63-1.34)	1.89(1.16-2.85)	1.76(1.17-2.36)	37.59(25.25-52.39)	75.65(47.86-111.62)	1.93(1.38-2.49)
Jamaica	Male	1.66(0.69-3.76)	2.16(0.91-4.79)	1.35(1.07-1.63)	40.99(25.57-69.09)	50.32(31.17-83.25)	1.2(0.94-1.47)	1.35(0.86-2)	2.15(1.29-3.34)	1.11(0.4-1.83)	45.59(30.21-64.31)	79.1(49.47-119.39)	1.31(0.57-2.06)
Japan	Both	1.58(1.27-1.92)	1.83(1.47-2.24)	1.21(0.92-1.51)	90.54(74.64-107.63)	97.03(79.53-117.68)	0.9(0.62-1.18)	0.46(0.3-0.67)	0.26(0.17-0.4)	-1.99(-2.3--1.68)	20.24(13.47-28.43)	14.52(9.44-20.69)	-1.03(-1.32--0.74)
Japan	Female	1.07(0.85-1.34)	1.21(0.93-1.52)	1.28(0.94-1.63)	100.01(81.2-121.64)	109.65(87.94-135.24)	0.99(0.69-1.29)	0.37(0.24-0.55)	0.17(0.11-0.26)	-2.57(-2.78--2.35)	16.78(11.16-23.11)	11.09(7.34-15.79)	-1.13(-1.33--0.93)
Japan	Male	2.1(1.68-2.58)	2.45(1.97-3.06)	1.16(0.89-1.43)	81.34(66.81-96.81)	85.02(69.94-102.03)	0.79(0.53-1.05)	0.56(0.37-0.83)	0.36(0.23-0.54)	-1.69(-2.06--1.32)	24.03(15.77-34.17)	18.05(11.56-25.79)	-0.98(-1.34--0.62)
Jordan	Both	1.29(0.53-2.96)	2.56(1.27-5.19)	2.82(2.6-3.04)	36.83(22.64-62.82)	67.54(44.51-109.65)	2.54(2.35-2.74)	1.27(0.79-1.89)	1.01(0.63-1.51)	-0.76(-1--0.51)	43.76(28.11-63.96)	35.53(23.14-51.27)	-0.73(-0.99--0.46)
Jordan	Female	1.21(0.43-2.96)	2.29(0.94-5.07)	2.74(2.52-2.97)	39.71(23.51-68.13)	76.43(46.86-128.53)	2.77(2.56-2.99)	1.26(0.78-1.95)	0.88(0.53-1.39)	-1.44(-1.95--0.93)	43.78(27.46-65.35)	30.9(19.93-45.97)	-1.39(-1.89--0.88)
Jordan	Male	1.37(0.55-3.03)	2.79(1.34-5.6)	2.86(2.64-3.09)	34.3(19.31-61.3)	60.49(36.89-103.45)	2.33(2.12-2.53)	1.27(0.78-1.93)	1.13(0.68-1.75)	-0.21(-0.28--0.13)	43.81(27.34-65)	39.44(24.53-58.42)	-0.2(-0.27--0.14)
Kazakhstan	Both	1.6(0.66-3.61)	2.29(1.03-4.79)	1.82(1.48-2.16)	55.29(36.83-85.42)	75.73(50.33-115.33)	1.51(1.24-1.77)	0.29(0.18-0.45)	0.31(0.19-0.47)	-1.03(-1.65--0.41)	14.73(9.27-21.94)	14.4(9.23-20.98)	-1.32(-1.93--0.71)
Kazakhstan	Female	1.21(0.39-3.15)	1.62(0.55-4.03)	1.32(1.06-1.59)	46.98(28.6-74.4)	66.19(40.04-108.3)	1.11(0.91-1.31)	0.25(0.15-0.38)	0.23(0.14-0.35)	-1.63(-2.26--1)	12.33(7.69-18.56)	10.77(6.92-15.9)	-1.94(-2.56--1.31)
Kazakhstan	Male	2.02(0.85-4.35)	3.02(1.39-6.18)	2.13(1.75-2.51)	64.85(41.2-101.37)	86.8(55.61-135.68)	1.8(1.45-2.16)	0.35(0.21-0.54)	0.41(0.25-0.62)	-0.51(-1.13-0.12)	17.47(11.15-26.28)	18.65(11.96-27.09)	-0.84(-1.46--0.22)
Kenya	Both	1(0.85-1.19)	1.25(1.06-1.48)	1.05(0.86-1.24)	56.28(49.29-64.18)	66.26(58.17-76.04)	1.02(0.83-1.21)	0.77(0.47-1.21)	0.96(0.59-1.48)	0.91(0.7-1.11)	26.73(17.47-39.32)	33.96(21.92-50)	1(0.78-1.22)
Kenya	Female	0.66(0.55-0.79)	0.88(0.73-1.06)	1.33(1.1-1.55)	57.44(49.55-66.7)	74.86(64.1-87.31)	1.36(1.17-1.55)	0.69(0.42-1.08)	0.88(0.51-1.4)	0.98(0.9-1.07)	24.41(15.39-37.34)	31.84(19.38-49.13)	1.08(0.99-1.16)
Kenya	Male	1.35(1.14-1.6)	1.62(1.36-1.91)	0.88(0.71-1.05)	54.98(47.87-63.38)	57.13(49.66-65.74)	0.59(0.4-0.78)	0.86(0.5-1.42)	1.06(0.63-1.71)	0.86(0.49-1.22)	29.15(18.55-45.47)	36.23(22.61-55.48)	0.93(0.56-1.3)
Kiribati	Both	1.09(0.32-2.57)	1.39(0.41-3.35)	0.89(0.82-0.95)	28.78(18.12-46.68)	33.5(20.3-55.23)	0.62(0.55-0.69)	5.34(3.6-7.79)	6.47(4.18-9.57)	0.37(-0.06-0.81)	222.51(155.97-313.36)	272.24(177.48-391.11)	0.4(-0.04-0.85)
Kiribati	Female	0.98(0.26-2.51)	1.24(0.34-3.22)	0.82(0.78-0.87)	28.32(17.1-46.91)	33.62(20.06-56.81)	0.69(0.62-0.76)	5.96(3.85-8.83)	6.14(3.81-9.64)	-0.19(-0.63-0.24)	248.42(165.22-365.6)	252.9(159.29-397.24)	-0.24(-0.67-0.2)
Kiribati	Male	1.2(0.36-2.92)	1.54(0.46-3.65)	0.94(0.86-1.03)	29.21(17.81-48.72)	33.21(19.23-57.38)	0.55(0.47-0.62)	4.66(3.06-6.94)	6.83(3.97-10.03)	1.02(0.57-1.47)	194.82(134.01-282.95)	293.1(171.97-431.16)	1.11(0.65-1.58)
Kuwait	Both	1.75(0.94-3.37)	2.76(1.59-5.07)	2.78(2.25-3.32)	49.07(32.68-74.57)	72.51(50.46-109.1)	2.51(1.97-3.06)	0.81(0.51-1.22)	0.37(0.23-0.59)	-3.25(-4.1--2.4)	28.67(18.72-40.77)	14.66(10-20.85)	-2.68(-3.3--2.04)
Kuwait	Female	1.54(0.68-3.44)	2.29(1.07-4.97)	1.71(1.44-1.99)	52.9(32.4-85.79)	76.91(48.78-118.8)	1.54(1.2-1.88)	0.85(0.53-1.31)	0.35(0.2-0.57)	-3.36(-4.1--2.61)	30.7(20.03-44.63)	13.92(9.07-20.69)	-3.14(-3.76--2.51)
Kuwait	Male	1.86(0.99-3.49)	3.1(1.79-5.57)	3.39(2.71-4.06)	46.28(29.86-70.78)	67.78(45.15-104.64)	3.15(2.45-3.87)	0.79(0.49-1.18)	0.39(0.24-0.61)	-3.21(-4.14--2.28)	27.45(17.48-39.71)	15.08(9.97-21.55)	-2.4(-3.05--1.74)

Kyrgyzstan	Both	2.18(0.87-4.87)	2.84(1.26-6.08)	1.37(1.13-1.61)	76.61(53.43-115.28)	103.44(70.91-154.28)	1.56(1.33-1.8)	0.58(0.35-0.9)	0.37(0.22-0.58)	-2.33(-2.9--1.76)	28.91(18.18-44.29)	19.55(12.53-28.76)	-2.04(-2.53--1.55)
Kyrgyzstan	Female	1.73(0.55-4.32)	2.15(0.75-5.15)	1.08(0.88-1.28)	70.83(46.71-111.71)	98.46(61.99-156.48)	1.53(1.32-1.74)	0.48(0.29-0.76)	0.29(0.17-0.45)	-2.54(-3.02--2.06)	24.56(15.43-37.69)	15.15(9.61-22.7)	-2.33(-2.74--1.92)
Kyrgyzstan	Male	2.66(1.14-5.89)	3.58(1.72-7.44)	1.57(1.3-1.84)	83(53.96-128.7)	109.56(72.48-168.57)	1.61(1.34-1.87)	0.69(0.41-1.07)	0.46(0.27-0.72)	-2.2(-2.82--1.57)	33.65(20.9-51.7)	24.29(15.47-35.38)	-1.83(-2.38--1.29)
Laos	Both	1.05(0.36-2.54)	1.32(0.45-3.17)	0.96(0.85-1.08)	33.54(21.78-54.04)	41.09(25.62-67.86)	0.99(0.88-1.1)	5.88(3.73-8.78)	4.11(2.57-6.15)	-1.39(-1.48--1.29)	236.68(155.05-345.38)	161.23(103.52-232.41)	-1.45(-1.55--1.35)
Laos	Female	0.89(0.25-2.41)	1.12(0.31-2.99)	0.88(0.77-1)	32.58(20.69-54.12)	41.11(25.33-69.58)	1.06(0.94-1.17)	6.1(3.78-9.17)	3.94(2.45-5.93)	-1.75(-1.87--1.63)	249.94(153.81-370.24)	156.13(97.68-228.22)	-1.85(-1.97--1.72)
Laos	Male	1.22(0.45-2.9)	1.53(0.54-3.6)	1(0.88-1.12)	34.53(21.38-57.41)	41.1(24.39-69.53)	0.93(0.81-1.05)	5.66(3.6-8.83)	4.3(2.61-6.6)	-1(-1.08--0.92)	222.52(144.19-334.46)	166.58(103.32-249.37)	-1.02(-1.11--0.93)
Latvia	Both	1.14(0.47-2.46)	2.06(0.95-4.11)	2.47(2.15-2.79)	58.73(38.84-87.61)	105.8(70.39-160.01)	2.52(2.26-2.79)	0.11(0.07-0.17)	0.37(0.23-0.56)	5.03(4.41-5.66)	4.26(2.94-6.06)	12.59(8.55-18.05)	4.46(3.94-4.97)
Latvia	Female	0.95(0.33-2.3)	1.61(0.62-3.7)	2.03(1.7-2.37)	58.86(37.62-91.14)	112.29(69.93-176.86)	2.6(2.35-2.85)	0.09(0.06-0.14)	0.34(0.19-0.56)	5.88(5.21-6.55)	3.37(2.3-4.86)	10.78(6.93-16.19)	5.05(4.53-5.58)
Latvia	Male	1.36(0.56-2.84)	2.56(1.19-4.92)	2.79(2.47-3.11)	59.24(36.67-91.73)	100.87(64.04-155.38)	2.47(2.19-2.75)	0.15(0.09-0.22)	0.4(0.25-0.6)	4.02(3.29-4.77)	5.42(3.7-7.59)	14.48(9.71-20.83)	3.89(3.24-4.54)
Lebanon	Both	1.38(0.68-2.74)	2.67(1.42-4.9)	2.65(2.53-2.77)	41.97(28.11-64.93)	73.6(48.87-114.16)	2.3(2.17-2.44)	0.97(0.61-1.53)	0.59(0.35-0.97)	-1.67(-1.97--1.38)	34.73(21.96-52.67)	22.78(14.19-35.04)	-1.41(-1.75--1.07)
Lebanon	Female	1.21(0.47-2.82)	2.23(0.93-4.82)	2.51(2.37-2.65)	43.67(27.36-71.16)	77.64(48.14-124.8)	2.33(2.19-2.48)	0.91(0.55-1.44)	0.54(0.31-0.83)	-2.03(-2.34--1.73)	33.78(20.78-52.12)	21.52(13.32-32.13)	-1.74(-2.09--1.4)
Lebanon	Male	1.56(0.71-3.08)	3.18(1.62-5.68)	2.82(2.7-2.94)	40.16(24.82-63.91)	69.98(44.45-111.86)	2.3(2.17-2.42)	1.03(0.62-1.63)	0.66(0.36-1.2)	-1.26(-1.6--0.93)	35.52(22.11-56.18)	24.45(14.23-41.87)	-1(-1.37--0.63)
Lesotho	Both	1.35(0.67-2.52)	1.71(0.8-3.4)	0.84(0.55-1.13)	50.67(35.86-71.29)	54.93(37.28-78.58)	0.39(0.22-0.57)	1.06(0.59-1.76)	2.6(1.43-4.27)	3.94(3.5-4.39)	37.66(21.97-60.01)	93.66(50.93-153.46)	4.06(3.6-4.51)
Lesotho	Female	0.76(0.32-1.63)	1.03(0.41-2.29)	1(0.71-1.28)	46.34(29.09-70.07)	51.7(32.36-78.73)	0.44(0.29-0.59)	0.6(0.33-1.02)	1.73(0.88-2.91)	5.31(4.63-6)	21.75(12.66-36.05)	62.06(31.99-103.59)	5.3(4.6-6)
Lesotho	Male	1.97(0.96-3.81)	2.43(1.12-4.88)	0.79(0.52-1.07)	55.03(35.7-80.58)	58.81(37.71-88.29)	0.4(0.21-0.6)	1.61(0.88-2.71)	3.7(1.97-6.27)	3.4(2.95-3.85)	55.28(31.12-90.95)	130.55(68.62-218.77)	3.58(3.12-4.04)
Liberia	Both	1.24(0.53-2.69)	1.52(0.66-3.3)	0.93(0.8-1.06)	45.15(29.81-70.86)	51.09(33.43-81.36)	0.75(0.64-0.87)	2.01(1.23-3.04)	1.64(0.95-2.7)	-0.46(-0.83--0.1)	71.83(44.24-107.18)	59.8(35.48-96.43)	-0.37(-0.76-0.01)
Liberia	Female	1.07(0.39-2.5)	1.26(0.47-2.93)	0.8(0.66-0.94)	45.29(28.13-78.05)	53.18(32.61-90.43)	0.83(0.71-0.96)	2.16(1.32-3.19)	1.73(0.96-2.69)	-0.52(-0.94--0.09)	79.11(49.74-117.14)	64.05(37.12-100.15)	-0.47(-0.9--0.04)
Liberia	Male	1.42(0.58-3.18)	1.77(0.73-4.12)	1.01(0.88-1.14)	45.03(26.55-74.11)	48.82(29.69-80.69)	0.67(0.54-0.8)	1.87(1.06-3.09)	1.56(0.82-2.81)	-0.37(-0.68--0.05)	64.7(37.83-105.58)	55.88(30.22-100.84)	-0.22(-0.56-0.11)
Libya	Both	1.41(0.69-2.89)	2.68(1.45-5.12)	2.55(2.38-2.72)	40.67(26.35-65.88)	73.53(49.79-118.18)	2.33(2.15-2.5)	0.93(0.56-1.47)	0.94(0.53-1.56)	0.15(-0.02-0.32)	34.02(21.09-51.67)	35.52(20.58-56.7)	0.3(0.11-0.49)
Libya	Female	1.25(0.51-2.92)	2.24(0.97-4.88)	2.34(2.18-2.5)	43.61(27.47-74.15)	77.95(49.6-131.36)	2.25(2.05-2.44)	0.96(0.56-1.53)	0.97(0.51-1.6)	0.31(-0.05-0.66)	36.23(21.72-55.64)	37.45(20.67-59.54)	0.35(-0.03-0.73)
Libya	Male	1.54(0.72-3.17)	3.1(1.6-5.92)	2.75(2.56-2.94)	38.34(23.29-65.41)	69.5(43.9-119.34)	2.4(2.22-2.58)	0.92(0.53-1.51)	0.91(0.48-1.67)	-0.02(-0.14-0.11)	32.44(18.99-51.72)	33.77(19.31-58.09)	0.22(0.12-0.32)
Lithuania	Both	1.28(0.56-2.85)	2.12(1.02-4.2)	2.15(1.86-2.45)	66.81(43.29-101.95)	119.89(79.86-182.55)	2.39(2.17-2.61)	0.15(0.1-0.22)	0.28(0.17-0.42)	1.29(0.43-2.17)	5.53(3.81-7.7)	10.27(7.01-14.21)	1.39(0.6-2.18)
Lithuania	Female	1.04(0.38-2.52)	1.67(0.67-3.83)	1.84(1.5-2.18)	68.46(41.07-112.26)	133.49(80.68-213.22)	2.51(2.28-2.73)	0.12(0.08-0.19)	0.24(0.15-0.37)	1.53(0.73-2.35)	4.4(3.03-6.21)	8.68(5.89-12.11)	1.59(0.87-2.3)
Lithuania	Male	1.54(0.69-3.3)	2.61(1.26-5.08)	2.4(2.13-2.68)	65.6(39.98-102.59)	107.52(68.41-165.47)	2.28(2.06-2.5)	0.19(0.12-0.28)	0.32(0.2-0.48)	1.04(0.14-1.96)	6.94(4.77-9.76)	12.15(8.23-17.02)	1.22(0.38-2.06)
Luxembourg	Both	1.43(0.93-2.22)	1.29(0.8-2.1)	0.04(-0.16-0.25)	64.27(46.26-89.02)	65.1(45.57-91.37)	0.39(0.22-0.56)	0.25(0.15-0.4)	0.22(0.13-0.38)	-0.19(-0.38-0)	9(6.08-12.72)	7.7(5.23-10.79)	-0.27(-0.47--0.08)
Luxembourg	Female	0.98(0.52-1.68)	0.95(0.49-1.68)	0.36(0.16-0.56)	60.9(40.59-90.02)	68.78(44.64-103.83)	0.74(0.57-0.9)	0.22(0.13-0.36)	0.21(0.12-0.35)	0.18(-0.03-0.39)	7.52(5.09-10.52)	7.14(4.8-10.1)	0.19(-0.02-0.4)
Luxembourg	Male	1.93(1.16-3.14)	1.63(0.94-2.74)	-0.22(-0.44-0.01)	68.27(46.53-100.84)	61.65(40.22-91.97)	0.01(-0.18-0.21)	0.29(0.17-0.48)	0.24(0.14-0.4)	-0.56(-0.74--0.37)	10.63(7.11-14.9)	8.27(5.56-11.79)	-0.68(-0.87--0.5)
Macedonia	Both	1.04(0.52-2.13)	1.67(0.88-3.36)	2.01(1.86-2.16)	43.83(29.44-66.52)	64.87(43.01-98.76)	1.77(1.59-1.95)	0.25(0.14-0.39)	0.25(0.14-0.42)	-2(-3.58--0.4)	10.49(6.57-15.94)	10.99(6.88-17.17)	-1.16(-2.29--0.02)
Macedonia	Female	0.76(0.29-1.76)	1.32(0.53-2.95)	2.22(2.08-2.37)	44.71(28.03-69.26)	72.71(45.03-113.39)	2.02(1.85-2.2)	0.21(0.12-0.32)	0.22(0.12-0.37)	-1.93(-3.68--0.15)	8.92(5.59-13.44)	9.7(6.08-14.93)	-1.1(-2.38-0.19)
Macedonia	Male	1.32(0.64-2.71)	2(1.03-3.95)	1.87(1.72-2.01)	43.1(27.97-69.32)	57.85(36.91-92.62)	1.51(1.33-1.7)	0.29(0.17-0.46)	0.29(0.16-0.48)	-2.07(-3.52--0.6)	12.14(7.48-18.6)	12.28(7.52-19.4)	-1.24(-2.27--0.2)
Madagascar	Both	1(0.44-2.12)	1.14(0.53-2.42)	0.7(0.59-0.81)	45.11(30.83-64.5)	54.67(37.48-78.58)	1.13(0.98-1.29)	1.06(0.65-1.68)	0.9(0.53-1.47)	-0.62(-0.69--0.55)	38.98(25.08-59.57)	32.07(19.61-50.45)	-0.65(-0.73--0.56)
Madagascar	Female	0.63(0.24-1.51)	0.78(0.3-1.82)	0.91(0.78-1.03)	43.94(28.32-65.81)	57.91(37.26-86.92)	1.38(1.22-1.53)	0.97(0.58-1.63)	0.85(0.46-1.5)	-0.51(-0.61--0.4)	37.27(22.57-60.79)	31.14(17.9-52.01)	-0.63(-0.75--0.5)
Madagascar	Male	1.37(0.57-2.88)	1.51(0.67-3.14)	0.59(0.48-0.7)	46.3(28.32-71.54)	51.24(31.88-79.4)	0.88(0.71-1.04)	1.15(0.67-1.86)	0.95(0.54-1.62)	-0.66(-0.73--0.6)	40.54(25.36-61.64)	33.07(20.04-52.93)	-0.65(-0.69--0.6)
Malawi	Both	0.87(0.39-1.86)	1.07(0.48-2.31)	1.08(0.9-1.27)	60.49(37.19-95.65)	69.98(43.13-109)	1.01(0.78-1.24)	1.2(0.74-1.86)	1.11(0.67-1.72)	-0.5(-0.73--0.27)	41.49(26.46-62.17)	37.88(24.21-56.09)	-0.54(-0.77--0.3)
Malawi	Female	0.56(0.22-1.31)	0.69(0.29-1.56)	1.13(0.91-1.35)	62.24(34.89-105.17)	78.09(45.03-131.15)	1.3(1.05-1.54)	1.12(0.66-1.78)	0.87(0.52-1.35)	-1.14(-1.31--0.96)	39.96(24.07-60.45)	29.88(18.26-44.5)	-1.32(-1.51--1.13)
Malawi	Male	1.19(0.5-2.61)	1.47(0.6-3.25)	1.07(0.9-1.24)	58.47(34.19-97.73)	60.78(35.8-99.21)	0.63(0.41-0.84)	1.3(0.77-2.04)	1.39(0.84-2.14)	0.02(-0.33-0.37)	43.37(27.35-64.64)	46.89(29.19-69.92)	0.09(-0.26-0.44)
Malaysia	Both	0.79(0.29-1.99)	1.18(0.45-2.93)	1.56(1.45-1.67)	31.29(20.72-47.41)	40.78(26.58-64.67)	1.18(1.05-1.31)	2.31(1.57-3.17)	1.99(1.24-3)	-0.91(-1.11--0.71)	88.01(62.35-117.42)	72.88(48.77-104.86)	-0.97(-1.16--0.78)
Malaysia	Female	0.67(0.2-1.79)	1.04(0.33-2.69)	1.65(1.52-1.77)	30.95(19.87-49.14)	42.11(26.62-67.4)	1.28(1.16-1.41)	2(1.34-2.81)	1.87(1.16-2.87)	-0.73(-0.96--0.5)	75.07(52.38-100.7)	66.35(43.07-97.16)	-0.83(-1.02--0.63)
Malaysia	Male	0.91(0.32-2.23)	1.32(0.49-3.2)	1.48(1.37-1.6)	31.61(20.19-50.86)	39.65(25.09-65.09)	1.09(0.96-1.23)	2.62(1.78-3.7)	2.09(1.3-3.14)	-1.08(-1.28--0.88)	100.86(71.36-135.83)	78.76(51.93-114.88)	-1.11(-1.31--0.91)
Maldives	Both	0.97(0.38-2.2)	1.31(0.54-2.93)	1.35(1.2-1.49)	38.21(25.09-57.94)	46.47(30.28-73.71)	0.97(0.83-1.11)	4.88(3.24-7.11)	2.02(1.33-2.91)	-3.46(-3.84--3.08)	183.27(124.64-253.71)	72.03(50.37-99.43)	-3.56(-3.95--3.17)

Maldives	Female	0.79(0.26-2.04)	1.07(0.37-2.68)	1.25(1.12-1.39)	37.13(23.3-57.56)	47.52(29.53-73.56)	1.1(0.97-1.24)	5.88(3.45-9.25)	2.1(1.37-3.07)	-3.98(-4.4--3.56)	218.95(133.19-336.32)	72.62(49.98-101.76)	-4.17(-4.6--3.72)
Maldives	Male	1.13(0.44-2.58)	1.52(0.61-3.41)	1.36(1.2-1.51)	39.03(25.16-62.96)	46.58(28.95-77.26)	0.95(0.81-1.09)	4.08(2.68-5.79)	1.94(1.28-2.85)	-2.96(-3.3--2.63)	152.91(105.18-210.33)	70.89(48.81-99.78)	-2.96(-3.29--2.63)
Mali	Both	1.99(0.82-4.53)	2.34(0.94-5.54)	0.76(0.6-0.93)	52.11(34.77-77.79)	56.64(37.65-87.39)	0.72(0.58-0.86)	1.91(1.14-2.95)	1.55(0.91-2.48)	-0.56(-0.78--0.34)	69.98(42.8-108)	56.65(34.3-90.36)	-0.59(-0.81--0.36)
Mali	Female	1.8(0.63-4.29)	2.06(0.7-5.08)	0.63(0.45-0.8)	48.73(31.17-76.93)	54.34(33.81-88.89)	0.77(0.63-0.92)	2.09(1.17-3.46)	1.56(0.86-2.6)	-0.92(-1.13--0.72)	79.15(44.7-128.75)	57.65(31.7-95.19)	-1(-1.21--0.78)
Mali	Male	2.18(0.88-4.99)	2.61(1.03-5.94)	0.86(0.7-1.02)	55.74(34.03-86.41)	58.68(36.54-92.33)	0.65(0.49-0.8)	1.71(1.04-2.73)	1.54(0.87-2.5)	-0.13(-0.36-0.1)	60.01(37.02-92.03)	55.68(32.15-89.33)	-0.05(-0.29-0.19)
Malta	Both	1.11(0.68-1.78)	1.65(1.09-2.47)	1.98(1.74-2.22)	49.75(36.08-68.64)	70.86(50.81-97.46)	1.89(1.64-2.15)	0.35(0.2-0.58)	0.27(0.16-0.43)	-0.8(-1.04--0.57)	10.4(6.8-15.22)	9.82(6.61-13.78)	0.12(-0.04-0.29)
Malta	Female	0.8(0.44-1.43)	1.18(0.65-2.01)	2.17(1.8-2.54)	49.09(32.53-72.51)	69.7(46.18-103.02)	2.04(1.67-2.41)	0.31(0.18-0.52)	0.24(0.14-0.39)	-0.86(-1.09--0.64)	9.02(6.07-13.11)	8.6(5.76-12.22)	0.17(0-0.34)
Malta	Male	1.43(0.84-2.41)	2.13(1.32-3.38)	1.81(1.61-2.03)	50.63(34.6-71.44)	72.36(49.36-104.81)	1.74(1.52-1.95)	0.41(0.23-0.67)	0.3(0.17-0.48)	-0.79(-1.07--0.52)	12.06(7.7-17.61)	11.07(7.26-15.7)	0.03(-0.14-0.2)
Marshall Islands	Both	0.95(0.28-2.45)	1.25(0.36-3.19)	0.99(0.87-1.11)	25.8(15.72-45.02)	29.8(17.51-53.88)	0.59(0.49-0.69)	3.89(2.5-5.8)	5.45(3.3-8.39)	1.06(0.74-1.39)	156.55(106.23-223.99)	224.07(139.67-339.76)	1.11(0.77-1.45)
Marshall Islands	Female	0.83(0.21-2.25)	1.09(0.28-2.94)	1(0.88-1.11)	24.78(14.95-43)	28.51(16.59-52.49)	0.57(0.46-0.67)	3.72(2.26-5.99)	5.53(3.17-9.5)	1.12(0.66-1.59)	150.58(95.86-239.02)	225.45(131.09-378.04)	1.14(0.66-1.61)
Marshall Islands	Male	1.07(0.32-2.88)	1.39(0.42-3.7)	0.99(0.87-1.11)	26.71(15.48-49.4)	31.05(17.57-59.14)	0.63(0.54-0.72)	4.08(2.66-6.04)	5.36(3.37-7.91)	0.98(0.78-1.18)	163.05(110.4-235.49)	222.31(142.91-325.75)	1.06(0.84-1.28)
Mauritania	Both	1.82(0.89-3.79)	2.33(1.21-4.69)	1.23(1.08-1.38)	71.61(49.87-105.08)	87.97(61.69-128.37)	1.23(1.05-1.41)	2.45(1.51-3.74)	1.41(0.78-2.17)	-1.73(-1.88--1.59)	87.52(55.47-131.31)	51.18(29.58-79)	-1.62(-1.77--1.46)
Mauritania	Female	1.54(0.66-3.49)	1.91(0.84-4.18)	1.04(0.89-1.2)	68.36(43.19-103.53)	84.11(52.88-129.75)	1.09(0.92-1.26)	2.76(1.61-4.34)	1.63(0.89-2.61)	-1.72(-1.84--1.6)	100.12(59.48-154.5)	58.8(33.17-93.06)	-1.69(-1.81--1.56)
Mauritania	Male	2.11(0.99-4.56)	2.76(1.38-5.59)	1.36(1.2-1.52)	74.99(48.31-116.31)	91.89(59.51-140.68)	1.36(1.14-1.59)	2.13(1.26-3.33)	1.17(0.62-1.97)	-1.76(-1.96--1.55)	74.35(46.44-115.78)	43.05(22.8-71.37)	-1.54(-1.76--1.32)
Mauritius	Both	1.13(0.38-2.93)	1.82(0.6-4.8)	1.5(1.43-1.57)	37.52(24.43-63.22)	49.75(31.33-88.63)	0.83(0.74-0.92)	4.49(3-6.3)	7.65(4.92-11.22)	2.26(1.83-2.7)	171.5(119.94-229.81)	290.45(194.92-418.72)	2.24(1.82-2.67)
Mauritius	Female	0.98(0.27-2.67)	1.52(0.41-4.21)	1.41(1.34-1.47)	37.22(22.75-63.05)	48.59(28.4-87.85)	0.77(0.67-0.87)	3.85(2.59-5.34)	6.21(3.99-9.27)	1.84(1.43-2.25)	148.5(104.03-197.92)	235.63(157.22-340.99)	1.78(1.39-2.17)
Mauritius	Male	1.29(0.43-3.28)	2.12(0.68-5.37)	1.57(1.48-1.65)	37.88(24.07-64.58)	50.94(30.56-93.05)	0.89(0.81-0.97)	5.19(3.43-7.28)	9.16(5.78-13.55)	2.56(2.1-3.03)	195.84(135.14-264.11)	346.35(229.63-496.86)	2.58(2.11-3.05)
Mexico	Both	1.33(1.01-1.78)	2.3(1.79-3.08)	1.82(1.71-1.92)	33.32(28.43-39.89)	51.14(43.14-62.17)	1.51(1.4-1.62)	1.4(0.95-1.96)	3.47(2.15-5.18)	3.45(2.89-4.01)	52.29(36.55-70.63)	122.87(79.15-175.55)	3.28(2.8-3.76)
Mexico	Female	1.08(0.8-1.45)	1.71(1.3-2.3)	1.55(1.48-1.62)	32.95(27.96-39.35)	48.87(41.31-58.71)	1.4(1.28-1.51)	1.41(0.97-1.96)	3.08(1.86-4.86)	2.96(2.33-3.59)	52.84(37.57-70.75)	107.22(67.24-160.98)	2.74(2.21-3.28)
Mexico	Male	1.59(1.2-2.13)	2.89(2.2-3.91)	1.98(1.85-2.11)	33.62(27.88-41.05)	53.28(43.76-65.32)	1.61(1.48-1.74)	1.38(0.93-1.95)	3.91(2.4-5.81)	3.95(3.45-4.45)	51.7(35.74-70.87)	140.08(88.96-203.06)	3.81(3.37-4.25)
Micronesia	Both	1.2(0.39-3.05)	1.62(0.5-4.24)	1.12(1.04-1.21)	33.04(20.79-54.74)	37.52(21.96-65.98)	0.58(0.42-0.74)	5.27(3.44-7.67)	7.76(4.26-11.71)	1.26(0.84-1.68)	216.46(144.31-313.35)	314.85(163.35-470.82)	1.21(0.78-1.64)
Micronesia	Female	1.04(0.28-2.79)	1.4(0.36-3.82)	1.07(0.99-1.14)	31.93(19.63-53.14)	36.1(21.06-62.69)	0.55(0.38-0.73)	5.42(3.36-8.07)	7.11(3.75-11.29)	0.71(0.18-1.24)	220.43(139.46-322.71)	284.33(137.92-460.47)	0.64(0.11-1.18)
Micronesia	Male	1.35(0.45-3.35)	1.84(0.57-4.66)	1.18(1.07-1.28)	34.02(21.12-56.94)	38.95(22.41-71.17)	0.62(0.48-0.76)	5.11(3.32-7.6)	8.41(4.35-13.7)	1.78(1.47-2.1)	212.51(141.14-315.08)	344.31(174.24-555.94)	1.74(1.41-2.07)
Moldova	Both	0.84(0.36-1.77)	1.55(0.72-3.09)	2.28(2.01-2.55)	50.71(34.94-77.36)	88.12(58.66-137.02)	2.24(1.98-2.5)	0.19(0.13-0.27)	0.36(0.23-0.52)	2.01(1.52-2.51)	6.79(4.8-9.26)	13.21(9.1-18.9)	1.98(1.48-2.49)
Moldova	Female	0.64(0.22-1.46)	1.17(0.45-2.6)	2.06(1.77-2.36)	50.74(31.14-82.24)	95.05(56.19-155.7)	2.42(2.16-2.69)	0.15(0.1-0.21)	0.3(0.19-0.45)	2.74(2.22-3.25)	5.05(3.54-7)	10.73(7.31-15.28)	2.64(2.15-3.13)
Moldova	Male	1.05(0.46-2.23)	1.94(0.91-3.97)	2.43(2.16-2.69)	50.9(33.11-79.38)	81.96(52.62-128.34)	2.07(1.81-2.33)	0.25(0.17-0.36)	0.42(0.27-0.61)	1.37(0.85-1.89)	8.91(6.3-12.33)	15.89(10.77-22.53)	1.48(0.93-2.03)
Mongolia	Both	2.72(1.38-5.43)	3.72(1.94-7.29)	1.51(1.27-1.75)	102.39(72.46-145.3)	142.44(99.56-203.91)	1.55(1.32-1.78)	0.99(0.59-1.53)	0.54(0.31-0.9)	-3.41(-3.9--2.92)	44.11(26.95-67.09)	27.32(17.02-42.57)	-2.82(-3.26--2.38)
Mongolia	Female	2.13(0.86-4.87)	2.9(1.17-6.42)	1.37(1.15-1.6)	95.56(62.5-144.29)	143.26(91.1-214.13)	1.74(1.53-1.94)	1.05(0.61-1.71)	0.41(0.23-0.71)	-4.66(-5.24--4.09)	47.49(27.7-75.92)	21.34(13.06-34.55)	-4.05(-4.59--3.52)
Mongolia	Male	3.31(1.66-6.59)	4.63(2.37-8.87)	1.66(1.39-1.94)	109.21(73.47-159.78)	142.91(94.75-211.3)	1.41(1.15-1.67)	0.94(0.55-1.49)	0.68(0.39-1.12)	-2.32(-2.77--1.87)	40.97(25.04-62.88)	33.85(20.92-53.47)	-1.72(-2.12--1.32)
Montenegro	Both	1.38(0.67-2.81)	1.95(1.06-3.69)	1.79(1.57-2.01)	49.5(34.03-74.19)	71.03(48.37-106.56)	1.87(1.65-2.08)	0.49(0.31-0.74)	0.49(0.3-0.78)	0.88(0.31-1.46)	18.64(12.14-27.19)	18.52(11.99-27.26)	0.66(0.2-1.12)
Montenegro	Female	0.95(0.39-2.1)	1.47(0.63-3.05)	2.04(1.81-2.27)	48.95(29.85-79.04)	75.89(46.93-121.49)	2.06(1.86-2.27)	0.36(0.23-0.56)	0.39(0.24-0.62)	0.98(0.44-1.52)	13.49(8.56-19.86)	14.86(9.6-21.97)	0.77(0.35-1.2)
Montenegro	Male	1.81(0.83-3.76)	2.45(1.25-4.71)	1.66(1.44-1.88)	50.38(32.29-79.29)	66.77(43.61-102.36)	1.66(1.43-1.9)	0.64(0.41-0.98)	0.61(0.36-0.99)	0.8(0.18-1.43)	24.2(15.83-35.93)	22.46(14.38-33.85)	0.58(0.08-1.09)
Morocco	Both	1.22(0.6-2.45)	2.63(1.34-5.11)	2.9(2.71-3.09)	40.57(27.12-60.28)	71.76(45.86-109.46)	2.24(2.06-2.42)	0.86(0.51-1.32)	0.95(0.54-1.54)	0.7(0.56-0.84)	30.78(19.37-45.81)	34.31(20.35-53.89)	0.78(0.62-0.94)
Morocco	Female	1.05(0.43-2.3)	2.11(0.88-4.63)	2.66(2.53-2.79)	42.35(26.7-64.95)	72.46(43.63-115.1)	2.08(1.9-2.25)	0.77(0.45-1.2)	0.95(0.53-1.59)	1.25(1-1.49)	29.07(17.22-44.35)	35.89(20.79-57.09)	1.26(1.02-1.49)
Morocco	Male	1.39(0.67-2.71)	3.16(1.6-6.03)	3.07(2.84-3.31)	38.74(24.91-62.73)	71.03(44.85-117.47)	2.42(2.22-2.62)	0.94(0.55-1.5)	0.95(0.51-1.57)	0.17(0.04-0.31)	32.54(19.5-49.86)	32.7(18.77-52.83)	0.29(0.17-0.41)
Mozambique	Both	0.88(0.39-1.92)	1.14(0.48-2.57)	1.12(1.03-1.2)	42.79(28.7-63.31)	48.02(32-73.88)	0.77(0.65-0.9)	0.96(0.57-1.49)	1.13(0.65-1.75)	0.71(0.59-0.83)	33.16(20.3-51.54)	40.06(24.03-61.01)	0.87(0.76-0.98)
Mozambique	Female	0.51(0.21-1.23)	0.69(0.29-1.65)	1.29(1.15-1.42)	43.42(27.04-66.03)	51.56(32.07-79.54)	0.98(0.84-1.11)	0.82(0.48-1.25)	0.86(0.48-1.35)	0.21(0.09-0.32)	28.99(17.93-44.42)	30.4(17.54-48.21)	0.27(0.12-0.42)
Mozambique	Male	1.26(0.5-2.94)	1.61(0.59-3.9)	1.05(0.98-1.12)	41.74(25.96-66.51)	43.58(26.44-73.22)	0.53(0.41-0.65)	1.11(0.62-1.84)	1.45(0.86-2.27)	1.14(0.93-1.35)	37.8(22.26-60.85)	51.32(31.33-76.58)	1.34(1.15-1.52)
Myanmar	Both	1.23(0.4-3.07)	1.62(0.53-3.95)	1.06(0.95-1.17)	35.21(22.07-58.44)	46.03(28.23-77.08)	1.21(1.09-1.34)	4.07(2.67-5.95)	2.96(1.97-4.15)	-1.23(-1.4--1.06)	180.78(119.98-257.39)	128.98(86.05-179.38)	-1.3(-1.48--1.12)
Myanmar	Female	1.19(0.31-3.35)	1.46(0.41-4.12)	0.8(0.7-0.91)	34.28(20.21-58.78)	46.78(27.48-80.26)	1.35(1.23-1.48)	4.5(2.88-6.63)	2.76(1.83-3.89)	-1.95(-2.21--1.69)	206.76(133.39-302.3)	121.3(80.65-172.57)	-2.12(-2.38--1.85)

Myanmar	Male	1.28(0.4-3.22)	1.78(0.57-4.37)	1.31(1.19-1.43)	36.23(21.81-59)	45.37(26.29-77.57)	1.07(0.94-1.2)	3.64(2.29-5.44)	3.2(2.12-4.5)	-0.41(-0.51--0.32)	154.34(100.78-227.15)	137.91(92.28-191.98)	-0.34(-0.44--0.24)
Namibia	Both	1.25(0.64-2.5)	1.65(0.83-3.25)	1.45(1.09-1.82)	46.11(31.13-66.33)	58.63(39.39-85.7)	1.36(1.11-1.62)	1.11(0.59-1.95)	1.03(0.54-1.85)	-0.72(-1.27--0.16)	38.44(20.86-66.75)	36.55(20.15-63.71)	-0.64(-1.19--0.08)
Namibia	Female	0.68(0.27-1.56)	0.96(0.39-2.16)	1.6(1.22-1.99)	39.89(25.04-60.87)	53.46(33.29-81.39)	1.47(1.26-1.69)	0.7(0.36-1.33)	0.61(0.29-1.31)	-0.99(-1.46--0.53)	25(13.05-47.1)	21.36(10.46-44.08)	-1.02(-1.46--0.58)
Namibia	Male	1.84(0.93-3.61)	2.4(1.22-4.54)	1.43(1.08-1.79)	52.66(33.16-79.77)	64.52(41.23-100.25)	1.29(1.01-1.57)	1.57(0.81-2.87)	1.57(0.82-2.82)	-0.48(-1.09-0.14)	53.26(27.54-94.62)	54.58(29.48-95.7)	-0.37(-1.01-0.27)
Nepal	Both	1.13(0.51-2.35)	1.7(0.74-3.63)	1.51(1.4-1.61)	38.31(25.98-58.09)	46.85(31.19-73.62)	1.01(0.85-1.16)	1(0.54-1.72)	1.34(0.74-2.24)	1.22(0.85-1.59)	35.93(20.57-60.5)	46.42(26.83-78.06)	1.06(0.69-1.44)
Nepal	Female	0.81(0.31-1.96)	1.3(0.47-3.06)	1.64(1.53-1.75)	35.95(22.54-57.24)	44.12(27.66-71.69)	1.04(0.87-1.21)	0.99(0.51-1.71)	1.34(0.68-2.23)	1.23(0.73-1.73)	36.21(18.99-59.88)	46.84(24.54-77.2)	1.05(0.54-1.56)
Nepal	Male	1.43(0.64-3.04)	2.11(0.89-4.65)	1.46(1.34-1.57)	40.47(24.97-65.7)	48.92(28.83-82.29)	0.94(0.8-1.09)	1(0.51-1.81)	1.33(0.68-2.45)	1.21(0.96-1.46)	35.57(19.26-62.83)	45.77(24.79-83.33)	1.07(0.82-1.33)
Netherlands	Both	1.03(0.62-1.7)	1.71(1.13-2.49)	2.36(2.16-2.57)	63.2(44.38-92.31)	98.02(70.63-139.13)	1.67(1.41-1.93)	0.15(0.08-0.23)	0.15(0.08-0.25)	-1.43(-1.83--1.02)	5.51(3.76-8)	6.27(4.39-8.99)	-0.1(-0.29-0.09)
Netherlands	Female	0.71(0.37-1.27)	1.25(0.69-2.04)	2.57(2.36-2.78)	67.86(44-107.41)	109.16(69.7-166.66)	1.66(1.39-1.93)	0.13(0.08-0.22)	0.14(0.08-0.23)	-1.43(-1.86--1)	4.85(3.27-7.15)	5.64(3.79-8.01)	-0.06(-0.26-0.13)
Netherlands	Male	1.36(0.76-2.26)	2.17(1.36-3.36)	2.22(2.01-2.43)	59.03(38.46-89.88)	87.18(59.11-125.53)	1.65(1.4-1.91)	0.16(0.09-0.26)	0.16(0.09-0.27)	-1.49(-1.89--1.08)	6.23(4.19-8.99)	6.92(4.64-9.91)	-0.16(-0.37-0.05)
New Zealand	Both	2.67(1.83-3.8)	2.87(1.99-4.02)	1.39(0.8-1.99)	111.9(84.32-149.45)	113.51(85.09-151.45)	0.88(0.42-1.34)	0.12(0.07-0.21)	0.13(0.07-0.21)	0.54(0.28-0.81)	7.48(5.01-10.62)	8.06(5.51-11.51)	0.88(0.6-1.17)
New Zealand	Female	2.19(1.28-3.5)	2.36(1.41-3.71)	1.29(0.76-1.82)	123.45(84.79-179.81)	127.01(89.06-184.82)	0.81(0.38-1.24)	0.12(0.07-0.2)	0.12(0.07-0.2)	0.71(0.32-1.11)	7.33(4.86-10.5)	7.81(5.35-11.01)	0.93(0.58-1.29)
New Zealand	Male	3.17(2.09-4.66)	3.42(2.22-5.08)	1.47(0.84-2.11)	100.01(72.05-139.32)	98.96(71.87-136.89)	0.95(0.45-1.45)	0.13(0.07-0.22)	0.14(0.08-0.24)	0.38(0.13-0.62)	7.64(4.95-11.11)	8.34(5.53-12.49)	0.84(0.61-1.06)
Nicaragua	Both	1.72(0.66-4.24)	2.62(1.01-6.38)	1.57(1.52-1.62)	38.55(22.78-70.4)	53.12(30.46-102.68)	1.32(1.25-1.38)	2.32(1.47-3.4)	4.65(2.72-7.2)	2.99(2.65-3.33)	82.79(53.87-117.51)	154.1(92.3-235.92)	2.82(2.48-3.16)
Nicaragua	Female	0.79(0.25-2.07)	1.06(0.34-2.71)	1.3(1.19-1.41)	29.84(17.31-50.6)	37.04(21.06-64.83)	1.05(0.94-1.16)	1.52(0.95-2.26)	2.89(1.7-4.55)	2.65(2.39-2.92)	56.2(36.38-82.17)	92.77(56.34-142.68)	2.25(2-2.51)
Nicaragua	Male	2.66(1-6.38)	4.18(1.54-10.11)	1.63(1.57-1.68)	47.34(25.65-93.16)	69.36(36.2-144.38)	1.48(1.42-1.53)	3.19(1.99-4.71)	6.69(3.9-10.47)	3.24(2.85-3.63)	111.37(71.5-159.66)	223.99(133.22-344.04)	3.17(2.77-3.58)
Niger	Both	1.76(0.86-3.36)	1.69(0.78-3.3)	0.38(0.1-0.65)	97.44(65.23-144.67)	84.51(53.33-132.81)	-0.01(-0.27-0.25)	1.69(1.02-2.71)	1.36(0.79-2.19)	-0.7(-0.84--0.56)	61.23(38.01-93.55)	49.65(29.83-79.5)	-0.66(-0.8--0.52)
Niger	Female	1.35(0.6-2.97)	1.24(0.53-2.86)	0.21(-0.06-0.49)	94.8(57.63-145.38)	84.31(51.29-136.44)	0.03(-0.21-0.28)	1.58(0.93-2.62)	1.19(0.67-1.95)	-1.12(-1.26--0.98)	59.92(36.21-95.41)	44.69(25.7-72.41)	-1.14(-1.28--0.99)
Niger	Male	2.16(1-4.13)	2.15(0.93-4.3)	0.51(0.23-0.79)	100.04(61.24-159.11)	84.67(49.84-140.75)	-0.05(-0.33-0.22)	1.77(1.04-2.96)	1.53(0.84-2.49)	-0.29(-0.44--0.13)	62.09(38-97.44)	54.8(31.29-89.76)	-0.19(-0.35--0.03)
Nigeria	Both	1.59(1.33-1.9)	2.13(1.8-2.51)	1.59(1.27-1.91)	65.75(57.58-74.9)	91.98(80.59-105.46)	1.64(1.46-1.81)	1.06(0.64-1.73)	0.92(0.54-1.43)	-0.76(-1.01--0.5)	37.43(23.12-58.18)	33.94(21.56-51.39)	-0.55(-0.8--0.29)
Nigeria	Female	1.44(1.19-1.74)	1.84(1.53-2.22)	1.58(1.16-2.01)	66.56(57.83-76.3)	97.98(84.42-114.37)	1.89(1.7-2.09)	1.08(0.59-1.74)	0.82(0.46-1.32)	-1.25(-1.48--1.01)	38.89(21.82-62.84)	30.54(18.59-47.36)	-1.09(-1.32--0.85)
Nigeria	Male	1.76(1.46-2.09)	2.44(2.03-2.88)	1.61(1.38-1.85)	64.97(56.01-75.1)	85.05(73.62-98.39)	1.35(1.18-1.51)	1.07(0.6-1.8)	1.03(0.56-1.79)	-0.33(-0.59--0.07)	36.58(21.36-60.89)	37.81(22.38-63.66)	-0.06(-0.31-0.19)
North Korea	Both	0.96(0.34-2.23)	1.16(0.44-2.66)	0.83(0.68-0.98)	39.07(26.76-58.6)	48.46(33.33-70.52)	1.02(0.9-1.15)	1.57(1.02-2.36)	1.33(0.81-1.94)	-0.61(-0.69--0.53)	69.15(45.47-101.98)	57.3(35.96-83.59)	-0.68(-0.75--0.62)
North Korea	Female	0.72(0.23-1.85)	0.89(0.3-2.34)	0.86(0.71-1.01)	39.66(26.41-59.41)	52.75(34.78-79.3)	1.26(1.13-1.38)	1.6(0.98-2.49)	1.27(0.77-1.92)	-0.81(-0.91--0.71)	69.85(43.5-107.34)	54.86(33.85-79.46)	-0.88(-0.96--0.79)
North Korea	Male	1.23(0.46-2.82)	1.44(0.58-3.2)	0.78(0.62-0.93)	38.38(25.05-59.74)	44.83(29.72-69.03)	0.85(0.72-0.98)	1.54(0.97-2.35)	1.39(0.83-2.09)	-0.37(-0.45--0.3)	68.25(43.46-104.77)	59.73(37.29-89.86)	-0.49(-0.54--0.43)
Northern Mariana Islands	Both	1.18(0.43-2.86)	1.49(0.53-3.69)	0.86(0.72-1.01)	34.46(22.53-55.5)	39.27(24.57-67.51)	0.66(0.53-0.78)	3.81(2.49-5.66)	4.39(2.85-6.38)	0.6(0.51-0.7)	149.93(99.61-217.84)	168.85(110.71-241.61)	0.48(0.4-0.57)
Northern Mariana Islands	Female	1.03(0.29-2.63)	1.23(0.33-3.19)	0.65(0.44-0.87)	33.13(20.59-53.63)	37.94(22.75-64.02)	0.68(0.53-0.82)	4.45(2.82-6.89)	4.24(2.64-6.29)	-0.15(-0.27--0.04)	172.95(111.26-263.23)	162.73(104.34-234.06)	-0.22(-0.33--0.11)
Northern Mariana Islands	Male	1.29(0.47-3.08)	1.73(0.6-4.27)	1.12(1.01-1.22)	35.1(21.84-59.57)	40.58(24.02-74.32)	0.72(0.61-0.83)	3.41(2.24-5.01)	4.54(2.98-6.65)	1.24(1.12-1.36)	134.92(88.31-196.37)	174.26(115.7-252.95)	1.08(0.98-1.19)
Norway	Both	1.01(0.84-1.18)	1.38(1.17-1.62)	2.44(2.07-2.81)	54.69(48.29-62.06)	75.58(65.99-86.12)	2.74(2.29-3.2)	0.11(0.07-0.18)	0.13(0.08-0.21)	-1.11(-1.78--0.43)	5.46(3.85-7.44)	5.71(4.04-7.74)	0.16(-0.11-0.43)
Norway	Female	0.71(0.57-0.87)	1.06(0.85-1.29)	3.01(2.58-3.44)	55.42(47.54-64.64)	84.09(70.66-99.5)	3.27(2.71-3.83)	0.08(0.05-0.13)	0.11(0.06-0.17)	-0.76(-1.47--0.04)	4.35(3.08-5.91)	5.05(3.58-6.8)	0.75(0.41-1.08)
Norway	Male	1.32(1.1-1.57)	1.71(1.43-2.03)	2.04(1.68-2.4)	54.33(47.5-61.88)	67.71(59.06-77.5)	2.1(1.71-2.49)	0.15(0.09-0.24)	0.15(0.09-0.25)	-1.51(-2.19--0.82)	6.68(4.66-9.25)	6.4(4.48-8.74)	-0.36(-0.61--0.1)
Oman	Both	1.13(0.57-2.2)	2.61(1.5-4.58)	3.35(3.13-3.56)	37.86(25.17-57.83)	73.46(50.29-110.09)	2.83(2.56-3.11)	0.44(0.25-0.72)	0.4(0.25-0.62)	-0.02(-0.28-0.25)	15.23(9.21-24.09)	14.84(10.02-21.32)	0.12(-0.2-0.45)
Oman	Female	0.93(0.37-1.97)	2.04(0.92-4.06)	3.1(2.89-3.31)	38.92(23.97-61.07)	80.45(50.5-123.61)	3.03(2.73-3.33)	0.42(0.24-0.69)	0.4(0.25-0.61)	0.11(-0.1-0.33)	15.46(9.38-24.15)	15.6(10.74-22.08)	0.29(0.03-0.56)
Oman	Male	1.25(0.63-2.42)	2.96(1.67-5.24)	3.42(3.18-3.66)	37.34(23.48-59.89)	71.53(45.92-110.94)	2.8(2.55-3.06)	0.47(0.26-0.82)	0.42(0.25-0.68)	-0.09(-0.35-0.17)	15.62(9.21-25.81)	14.93(9.62-22.54)	0.05(-0.27-0.38)
Pakistan	Both	1.19(0.73-1.96)	1.61(0.99-2.61)	1.41(1.26-1.55)	42.25(33.33-55.08)	53.02(41.11-70.9)	1.27(1.1-1.45)	1.38(0.79-2.29)	2.37(1.42-3.7)	1.87(1.54-2.19)	50.04(29.98-81.25)	88.47(54.21-136.17)	2(1.68-2.32)
Pakistan	Female	0.95(0.52-1.69)	1.24(0.69-2.18)	1.22(1.11-1.34)	43.59(32.68-58.88)	54.16(40.01-74.24)	1.17(1.01-1.33)	1.28(0.63-2.13)	2.23(1.13-3.67)	1.86(1.51-2.2)	46.75(23.16-76.27)	82.79(42.38-133.8)	1.96(1.61-2.3)
Pakistan	Male	1.4(0.87-2.22)	1.95(1.19-3.07)	1.53(1.37-1.7)	41.07(31.58-55.84)	51.69(39.15-71.13)	1.35(1.16-1.53)	1.46(0.77-2.65)	2.51(1.44-4.2)	1.9(1.59-2.21)	52.88(29.32-92.46)	93.91(55.57-157.54)	2.05(1.76-2.35)
Palestine	Both	1.8(0.83-3.79)	2.81(1.44-5.36)	1.67(1.58-1.76)	49.07(31.86-80.97)	78.3(50.31-125.03)	1.85(1.76-1.94)	1.41(0.84-2.22)	0.97(0.63-1.48)	-1.08(-1.32--0.84)	49.92(30.27-76.99)	35.8(24.15-51.42)	-1.02(-1.19--0.86)
Palestine	Female	1.48(0.54-3.59)	2.23(0.9-5.1)	1.48(1.43-1.54)	48.96(29.45-83.7)	80.97(49.67-135.1)	1.9(1.83-1.97)	1.23(0.73-1.96)	0.8(0.52-1.2)	-1.38(-1.57--1.19)	45.32(27.45-70.58)	30.39(20.39-43.49)	-1.35(-1.52--1.18)
Palestine	Male	2.15(0.98-4.38)	3.37(1.69-6.41)	1.76(1.63-1.89)	49.33(29.5-82.49)	75.82(45.87-125.09)	1.78(1.66-1.91)	1.65(0.94-2.66)	1.16(0.75-1.74)	-0.95(-1.32--0.57)	56.12(32.53-89.09)	41.22(27.02-60.17)	-0.89(-1.17--0.6)

Panama	Both	1.34(0.56-2.96)	1.88(0.74-4.05)	1.16(1.11-1.21)	37.5(24.28-60.72)	47.27(28.93-80.16)	0.98(0.91-1.05)	0.79(0.52-1.13)	1.43(0.85-2.29)	2.1(1.72-2.48)	29.5(20.33-40.44)	51.78(32.14-81.27)	1.98(1.64-2.33)
Panama	Female	0.82(0.3-2)	1.12(0.39-2.69)	1.15(1.11-1.2)	34.5(20.89-55.3)	41.75(25.04-68.66)	0.94(0.83-1.05)	0.66(0.42-0.95)	1.15(0.67-1.86)	2.01(1.54-2.48)	24.88(16.72-34.69)	41.2(25.29-64.99)	1.86(1.44-2.28)
Panama	Male	1.83(0.72-3.97)	2.62(1-5.71)	1.17(1.1-1.23)	40.34(23.53-68.84)	52.6(29.34-93.77)	1.02(0.96-1.07)	0.92(0.59-1.34)	1.73(1.02-2.73)	2.2(1.85-2.54)	33.96(23.16-46.48)	62.44(38.84-97.9)	2.1(1.77-2.43)
Papua New Guinea	Both	0.72(0.21-1.77)	0.92(0.27-2.3)	0.82(0.73-0.91)	24.63(15.74-38.77)	28.18(17.36-45.45)	0.56(0.51-0.62)	1.44(0.99-2.04)	1.63(1.06-2.45)	0.37(0.21-0.53)	64.08(45.07-88.28)	71.77(49.01-104.35)	0.33(0.17-0.48)
Papua New Guinea	Female	0.52(0.13-1.28)	0.67(0.16-1.71)	0.83(0.71-0.96)	23.99(15.05-38.27)	27.22(16.32-43.95)	0.54(0.48-0.61)	1.01(0.65-1.51)	1.12(0.72-1.67)	0.36(0.15-0.56)	45.44(29.72-65.34)	49.72(33.66-73.24)	0.32(0.11-0.52)
Papua New Guinea	Male	0.9(0.26-2.37)	1.15(0.32-2.96)	0.81(0.73-0.88)	25.18(15.36-42.31)	28.97(17.01-50.96)	0.58(0.53-0.63)	1.83(1.21-2.64)	2.1(1.36-3.22)	0.37(0.24-0.51)	81.36(54.48-114.48)	92.83(62.55-138.96)	0.35(0.22-0.48)
Paraguay	Both	1.18(0.56-2.51)	1.99(0.98-3.97)	2.04(1.89-2.18)	41.19(27.26-60.71)	69(45.97-101.7)	2.1(1.94-2.26)	0.78(0.51-1.16)	1.84(1.07-2.97)	3.55(3.29-3.82)	28.63(19.27-40.51)	63.24(38.61-99.52)	3.27(3.04-3.5)
Paraguay	Female	1.13(0.51-2.31)	2.1(0.95-4.01)	2.34(2.17-2.51)	53.81(35.44-82.14)	97.9(62.66-148.21)	2.35(2.18-2.52)	0.72(0.47-1.07)	1.62(0.91-2.65)	3.37(3.04-3.7)	27.69(18.66-39.4)	57.69(35.25-90.93)	3.04(2.77-3.32)
Paraguay	Male	1.22(0.49-2.7)	1.87(0.77-4.17)	1.75(1.62-1.88)	28.54(16.78-49.21)	40.59(23.35-71.85)	1.65(1.48-1.81)	0.85(0.53-1.29)	2.07(1.18-3.36)	3.7(3.49-3.91)	29.68(19.05-42.78)	68.94(41.2-108.61)	3.47(3.27-3.66)
Peru	Both	1.15(0.54-2.3)	1.82(0.88-3.56)	2.05(1.83-2.27)	58.9(37.94-88)	73.21(46.62-110.58)	1.26(1.03-1.49)	1.08(0.69-1.6)	0.96(0.55-1.57)	-0.3(-0.51--0.1)	38.83(25.59-55.47)	34.09(20.92-53)	-0.34(-0.54--0.14)
Peru	Female	0.91(0.38-2.03)	1.54(0.66-3.4)	2.25(2.03-2.47)	59.56(35.42-96.45)	77.13(46.6-122.32)	1.33(1.1-1.56)	1.01(0.64-1.5)	0.96(0.56-1.55)	-0.36(-0.61--0.11)	37.18(24.4-53.28)	34.14(20.91-53.24)	-0.39(-0.62--0.15)
Peru	Male	1.39(0.64-2.77)	2.1(0.98-4.08)	1.92(1.7-2.14)	58.11(34.52-91.07)	69.14(41.28-108.77)	1.19(0.95-1.42)	1.14(0.72-1.7)	0.96(0.54-1.59)	-0.24(-0.45--0.03)	40.51(26.61-58.13)	34.06(20.76-53.05)	-0.28(-0.48--0.09)
Philippines	Both	1.1(0.91-1.34)	1.78(1.47-2.16)	1.9(1.8-1.99)	38.61(34.32-43.41)	57.52(51-65.67)	1.97(1.77-2.17)	3.15(2.24-4.22)	4.42(2.93-6.16)	1.76(1.42-2.09)	126.25(91.1-163.82)	177.68(122.31-242.52)	1.84(1.49-2.19)
Philippines	Female	0.88(0.71-1.09)	1.38(1.11-1.71)	1.7(1.61-1.79)	37.87(33.49-42.94)	55.79(48.98-64.8)	1.92(1.68-2.15)	2.8(1.99-3.8)	3.75(2.4-5.51)	1.59(1.27-1.91)	111.82(81.41-145.05)	150.92(99.41-217.74)	1.69(1.37-2.02)
Philippines	Male	1.31(1.08-1.6)	2.17(1.79-2.63)	2.02(1.91-2.12)	39.33(34.97-44.33)	59.34(52.21-67.91)	2.03(1.84-2.23)	3.5(2.42-4.85)	5.11(3.25-7.55)	1.9(1.55-2.25)	140.76(99.88-186.62)	204.23(132.54-289.65)	1.95(1.58-2.31)
Poland	Both	1.16(0.89-1.54)	1.33(1.03-1.71)	0.63(0.41-0.85)	43.72(36.86-51.73)	63.51(52.96-76.3)	1.55(1.13-1.96)	0.42(0.26-0.62)	0.17(0.1-0.27)	-2.57(-3.05--2.08)	16.5(10.88-23.44)	7.32(4.8-10.41)	-2.17(-2.57--1.77)
Poland	Female	0.87(0.63-1.2)	1.1(0.81-1.47)	0.96(0.76-1.16)	45.6(37.87-54.47)	76.7(62.34-95.1)	1.94(1.5-2.39)	0.34(0.22-0.5)	0.14(0.08-0.22)	-2.7(-3.22--2.17)	13.37(8.88-18.53)	6.07(4.09-8.82)	-2.17(-2.58--1.75)
Poland	Male	1.47(1.13-1.9)	1.56(1.23-1.99)	0.42(0.18-0.67)	41.98(34.98-50.41)	50.75(42.16-60.59)	1.06(0.66-1.45)	0.51(0.32-0.76)	0.21(0.12-0.34)	-2.52(-2.99--2.05)	19.96(12.93-28.6)	8.65(5.44-12.69)	-2.21(-2.61--1.8)
Portugal	Both	0.84(0.48-1.46)	1.21(0.77-1.92)	1.78(1.62-1.95)	40.91(28.87-58.23)	62.29(45.82-85.15)	1.99(1.81-2.16)	0.38(0.23-0.59)	0.3(0.18-0.48)	-0.96(-1.44--0.48)	12.88(8.68-18.13)	9.86(6.75-14.03)	-0.97(-1.37--0.57)
Portugal	Female	0.57(0.29-1.07)	0.86(0.46-1.5)	1.99(1.79-2.2)	40.56(26.71-61.83)	63.65(43.26-93.85)	2.07(1.91-2.22)	0.29(0.18-0.46)	0.26(0.15-0.42)	-0.58(-1.1--0.05)	9.9(6.68-13.73)	8.27(5.72-11.64)	-0.64(-1.08--0.21)
Portugal	Male	1.12(0.59-2.04)	1.59(0.94-2.59)	1.7(1.53-1.87)	41.39(27.5-62.07)	61.19(42.85-86.25)	1.91(1.69-2.12)	0.49(0.29-0.76)	0.35(0.21-0.56)	-1.31(-1.75--0.86)	16.34(10.85-23.33)	11.66(7.95-16.54)	-1.22(-1.59--0.84)
Puerto Rico	Both	1.39(0.61-3.01)	2(0.91-4.35)	1.49(1.35-1.62)	46.74(32.22-69.81)	59.37(40.21-92.32)	1.11(0.96-1.27)	1.43(0.94-2.02)	1.38(0.81-2.15)	0.4(0.08-0.72)	54.46(36.7-74.08)	53.77(34.24-81.68)	0.43(0.14-0.73)
Puerto Rico	Female	0.87(0.31-2.2)	1.3(0.47-3.25)	1.55(1.45-1.64)	39.15(26.13-61.49)	51.12(33.34-82.1)	1.19(1.06-1.33)	1.07(0.7-1.54)	1.04(0.6-1.64)	0.41(0.1-0.72)	40.23(27.18-55.13)	40.54(25.5-60.66)	0.49(0.22-0.75)
Puerto Rico	Male	1.94(0.87-4.01)	2.73(1.24-5.81)	1.44(1.28-1.6)	54.96(35.99-85.99)	68.12(44.41-109.76)	1.04(0.86-1.22)	1.84(1.19-2.6)	1.77(1.03-2.79)	0.4(0.07-0.73)	70.64(47.23-96.81)	68.74(43.43-103.95)	0.39(0.07-0.71)
Qatar	Both	1.27(0.56-2.82)	2.38(1.17-4.85)	2.36(2.18-2.54)	36.06(23.03-60.54)	59.95(37.98-100.88)	1.73(1.57-1.9)	1.01(0.58-1.74)	0.71(0.43-1.12)	-1.14(-1.45--0.83)	28.61(17.05-46.45)	19.28(12.45-28.11)	-1.51(-1.78--1.24)
Qatar	Female	1.11(0.39-2.69)	1.91(0.69-4.7)	2.04(1.91-2.17)	37.99(22.2-65.24)	64.08(37.44-113.46)	1.67(1.5-1.83)	1.15(0.64-2.14)	0.96(0.57-1.55)	-0.43(-0.71--0.16)	34.26(20.05-59.65)	23.48(15.08-35.35)	-1.38(-1.56--1.2)
Qatar	Male	1.35(0.57-2.96)	2.58(1.2-5.29)	2.41(2.22-2.61)	35.41(21.41-59.61)	59.85(36.91-101.73)	1.87(1.69-2.05)	0.97(0.54-1.7)	0.63(0.37-0.99)	-1.51(-1.83--1.19)	26.69(15.48-43.79)	17.9(11.47-26.49)	-1.58(-1.88--1.29)
Republic of Congo	Both	0.9(0.38-2.05)	1.16(0.52-2.49)	1.3(1.1-1.5)	32.65(21.96-49.95)	43.41(29.48-66.82)	1.48(1.28-1.68)	1.96(1.11-3.16)	1.45(0.8-2.36)	-1.3(-1.47--1.13)	69.89(38.27-111.34)	50.42(29.1-79.6)	-1.37(-1.55--1.19)
Republic of Congo	Female	0.67(0.26-1.55)	0.93(0.39-2.03)	1.47(1.25-1.69)	34.47(21.61-53.49)	47.39(29.73-74.96)	1.5(1.28-1.71)	1.68(0.67-2.91)	1.41(0.56-2.47)	-0.7(-0.9--0.49)	61.61(24.87-105.13)	50.96(20.66-88.67)	-0.76(-1--0.53)
Republic of Congo	Male	1.14(0.44-2.66)	1.4(0.58-3.02)	1.15(0.96-1.34)	30.51(18.61-51.38)	39.19(24.04-63.45)	1.47(1.26-1.68)	2.32(1.34-3.74)	1.5(0.84-2.37)	-1.91(-2.13--1.69)	79.96(46.32-130.37)	50.25(29.14-78.78)	-1.99(-2.21--1.76)
Romania	Both	0.97(0.46-2.09)	1.66(0.9-3.11)	2.37(2.2-2.54)	41.85(27.94-62.41)	74.7(50.54-111.09)	2.71(2.48-2.93)	0.16(0.09-0.26)	0.17(0.09-0.28)	0.71(0.25-1.18)	6.94(4.17-10.41)	8.57(5.57-12.57)	1.53(1.1-1.96)
Romania	Female	0.74(0.33-1.65)	1.31(0.63-2.73)	2.45(2.27-2.63)	46.25(29.11-71.75)	88.31(54.8-143.84)	2.88(2.66-3.1)	0.15(0.08-0.25)	0.14(0.08-0.23)	-0.01(-0.52-0.5)	6.37(3.67-9.53)	7.44(4.83-10.87)	1.06(0.6-1.51)
Romania	Male	1.2(0.53-2.59)	2.03(1.05-3.82)	2.36(2.19-2.53)	37.37(22.93-60.15)	61.85(40.28-95.39)	2.53(2.3-2.76)	0.17(0.09-0.27)	0.19(0.11-0.32)	1.33(0.89-1.78)	7.52(4.52-11.48)	9.76(6.26-14.39)	1.91(1.49-2.33)
Russia	Both	2.36(1.99-2.78)	3.81(3.19-4.5)	2.31(2.03-2.59)	108.67(96.33-123.86)	177.31(154.29-207.07)	2.3(2.04-2.56)	0.5(0.34-0.71)	0.39(0.26-0.58)	-1.56(-1.98--1.14)	20.88(15.15-28.5)	16.47(11.89-22.44)	-1.59(-1.99--1.2)
Russia	Female	1.81(1.49-2.17)	3(2.47-3.64)	2.35(2.02-2.68)	107.47(94.31-124.01)	196.39(168.58-230.89)	2.68(2.34-3.03)	0.45(0.3-0.66)	0.35(0.22-0.54)	-1.48(-1.82--1.13)	18.48(13.28-25.63)	14.28(10.14-20.19)	-1.59(-1.92--1.26)
Russia	Male	2.94(2.48-3.46)	4.7(3.94-5.58)	2.34(2.06-2.62)	109.85(97.66-125.57)	159.13(138.82-184.24)	1.91(1.64-2.18)	0.55(0.38-0.79)	0.43(0.28-0.64)	-1.66(-2.15--1.17)	23.62(17.14-32.37)	18.94(13.17-26.13)	-1.57(-2.03--1.11)
Rwanda	Both	0.98(0.44-2)	1.19(0.59-2.31)	1.26(0.99-1.52)	42.18(28.8-61.06)	59.14(40.18-85.72)	1.92(1.65-2.19)	1.69(1.01-2.58)	1.02(0.61-1.59)	-2.76(-3.2--2.33)	59.46(37.02-87)	35.12(21.9-52.31)	-2.83(-3.27--2.39)
Rwanda	Female	0.65(0.25-1.45)	0.83(0.36-1.71)	1.45(1.17-1.73)	41.83(26.2-63.05)	63.67(40.63-96.92)	2.15(1.88-2.42)	1.55(0.94-2.37)	0.93(0.53-1.48)	-2.69(-3.14--2.24)	57.3(35.6-85.03)	32.34(18.92-49.05)	-2.94(-3.42--2.46)
Rwanda	Male	1.34(0.6-2.76)	1.57(0.76-3.12)	1.16(0.91-1.41)	42.44(27.65-65.56)	54.1(34.99-83.55)	1.65(1.38-1.93)	1.87(1.07-3.07)	1.14(0.67-1.79)	-2.82(-3.26--2.37)	62.55(36.52-98.36)	38.66(24.45-57.86)	-2.73(-3.16--2.3)
Saint Lucia	Both	1.07(0.39-2.56)	1.73(0.68-3.95)	1.76(1.64-1.88)	27.66(17.04-46.48)	40.82(25.46-69.31)	1.53(1.43-1.64)	1.9(1.26-2.7)	2.03(1.27-2.96)	0.47(0.24-0.71)	71.16(48.59-98.26)	77.32(50.15-110.42)	0.58(0.36-0.79)

Saint Lucia	Female	0.73(0.21-1.99)	1.17(0.37-3.09)	1.65(1.54-1.76)	23.2(13.74-40.72)	34.5(20.56-60.33)	1.48(1.39-1.57)	1.57(1.03-2.26)	1.66(1.02-2.51)	0.47(0.19-0.76)	59.57(40.07-82.74)	63.79(39.87-91.64)	0.62(0.33-0.9)
Saint Lucia	Male	1.42(0.55-3.33)	2.29(0.93-5.24)	1.78(1.65-1.91)	32.41(19.19-55.17)	47.14(27.66-82.42)	1.54(1.43-1.66)	2.3(1.51-3.26)	2.41(1.51-3.57)	0.41(0.2-0.61)	84.32(57.32-116.3)	91.19(58.77-132.23)	0.5(0.33-0.68)
Saint Vincent	Both	1.08(0.4-2.43)	1.67(0.64-3.81)	1.65(1.55-1.76)	29.17(18.29-47.8)	41.53(25.8-69.34)	1.47(1.37-1.57)	1.57(1.04-2.2)	2.31(1.48-3.33)	1.53(1.39-1.67)	59.69(40.77-80.73)	89.21(59.86-125)	1.54(1.43-1.64)
Saint Vincent	Female	0.84(0.25-2.24)	1.31(0.4-3.38)	1.63(1.53-1.72)	26.05(15.33-44)	38.59(22.67-66.03)	1.59(1.49-1.69)	1.42(0.95-2.03)	2.15(1.37-3.15)	1.57(1.4-1.75)	54.38(37.45-74.97)	83.66(55.2-120.38)	1.53(1.41-1.65)
Saint Vincent	Male	1.32(0.51-2.9)	2(0.79-4.4)	1.64(1.53-1.74)	32.39(19.46-54.24)	44.26(26.5-75.67)	1.34(1.24-1.45)	1.72(1.14-2.41)	2.47(1.58-3.52)	1.43(1.29-1.57)	65.34(44.14-88.7)	94.33(62.19-132.65)	1.5(1.38-1.62)
Samoa	Both	1.07(0.31-2.76)	1.38(0.41-3.67)	0.92(0.88-0.96)	31.72(20.13-52.49)	36.37(22.61-61.59)	0.61(0.55-0.67)	3.41(2.1-5.27)	4.09(2.53-6.26)	0.54(0.29-0.78)	134.84(85.73-205.41)	164.71(102.9-251.58)	0.61(0.35-0.86)
Samoa	Female	0.92(0.23-2.55)	1.18(0.3-3.27)	0.85(0.8-0.9)	31.04(19.06-50.96)	35.3(21.21-58.83)	0.55(0.49-0.61)	3.43(2.03-5.4)	4.12(2.5-6.27)	0.41(0.07-0.75)	136.55(83.92-215.66)	165.66(99.62-253.64)	0.47(0.13-0.82)
Samoa	Male	1.2(0.36-3.09)	1.56(0.47-3.95)	0.96(0.92-1)	32.41(19.25-56.29)	37.35(21.42-66.83)	0.65(0.59-0.72)	3.41(2.04-5.25)	4.08(2.47-6.21)	0.68(0.52-0.84)	133.35(82.54-206.18)	164.28(99.57-255.42)	0.76(0.58-0.93)
Sao Tome and Principe	Both	1.83(0.75-4.17)	2.41(1.05-5.29)	1.24(1.1-1.37)	58.58(38.83-89.69)	71.32(47.1-113.07)	1.08(0.92-1.25)	2.01(1.19-3.12)	2.36(1.43-3.74)	0.29(0-0.58)	73.13(42.68-112.97)	84.34(52.24-129.74)	0.21(-0.12-0.54)
Sao Tome and Principe	Female	1.6(0.61-3.61)	2.1(0.86-4.52)	1.23(1.06-1.39)	57.42(35.71-89.85)	73.04(45.35-113.92)	1.21(1.02-1.39)	2.15(1.25-3.35)	2.66(1.58-4.22)	0.3(-0.07-0.68)	79.38(45.18-124.11)	97.98(59.8-154.78)	0.26(-0.17-0.68)
Sao Tome and Principe	Male	2.05(0.77-4.68)	2.74(1.06-6.04)	1.27(1.15-1.39)	59.66(36.51-99.25)	69.66(41.56-119.25)	0.97(0.82-1.12)	1.86(1.12-2.97)	2.06(1.21-3.32)	0.37(0.21-0.53)	66.27(39.45-102.23)	71.22(43.6-113.6)	0.28(0.09-0.47)
Saudi Arabia	Both	1.7(0.89-3.17)	4.17(2.45-7.09)	3.29(2.92-3.65)	47.67(33.77-70.99)	97.29(70.43-142.35)	2.42(1.96-2.88)	1.58(0.9-2.63)	1.57(0.93-2.5)	0.12(-0.13-0.37)	55.26(32.39-87.84)	57.29(35.54-89.51)	0.22(-0.03-0.46)
Saudi Arabia	Female	1.52(0.68-3.09)	3.46(1.66-6.71)	2.95(2.63-3.27)	52.63(34.79-79.65)	102.61(67.88-154.23)	2.13(1.69-2.57)	1.44(0.83-2.37)	1.57(0.89-2.43)	0.17(-0.12-0.46)	54.6(31.08-88.13)	60.22(35.66-93.34)	0.21(-0.07-0.5)
Saudi Arabia	Male	1.82(0.88-3.42)	4.64(2.62-7.67)	3.46(3.07-3.85)	44.46(29.32-69.25)	93.59(64.95-140.24)	2.63(2.16-3.1)	1.69(0.91-2.9)	1.58(0.93-2.54)	0.04(-0.22-0.3)	56.1(31.35-93.58)	55.47(33.69-87.71)	0.18(-0.06-0.42)
Senegal	Both	1.28(0.53-2.96)	1.4(0.61-3.17)	0.46(0.35-0.57)	42.24(27.47-66.4)	45.88(29.22-73.68)	0.56(0.36-0.75)	2.01(1.2-3.21)	1.72(1.02-2.73)	-0.39(-0.61--0.16)	71.37(43.51-109.45)	61.21(36.4-94.31)	-0.35(-0.59--0.11)
Senegal	Female	0.99(0.37-2.4)	1.03(0.39-2.48)	0.29(0.12-0.46)	41.78(25.63-68.62)	45.97(28.16-76.02)	0.56(0.33-0.79)	1.99(1.13-3.37)	1.54(0.9-2.49)	-0.92(-1.15--0.69)	72.83(41.86-124.69)	55.5(32.52-89.76)	-0.96(-1.2--0.72)
Senegal	Male	1.58(0.62-3.63)	1.76(0.71-3.92)	0.51(0.44-0.58)	42.69(25.13-71.64)	45.56(26.74-76.26)	0.54(0.38-0.7)	2.02(1.21-3.28)	1.91(1.1-3.16)	0.14(-0.09-0.38)	69.31(42.78-105.86)	67.18(38.73-112.7)	0.27(0.01-0.53)
Serbia	Both	0.83(0.44-1.61)	1.4(0.79-2.47)	2.36(2.13-2.6)	43.22(29.93-64.7)	67.71(45.85-103.22)	2.02(1.85-2.2)	0.5(0.3-0.78)	0.44(0.25-0.74)	-1.23(-1.52--0.95)	17.83(11.19-27.23)	15.42(9.66-24.04)	-1.3(-1.56--1.05)
Serbia	Female	0.56(0.23-1.18)	1.12(0.5-2.22)	2.99(2.72-3.27)	42.8(27.21-70.43)	77.04(48.43-126.55)	2.48(2.29-2.67)	0.4(0.24-0.62)	0.38(0.21-0.63)	-1.25(-1.59--0.91)	14.15(8.82-21.15)	12.88(7.86-19.64)	-1.42(-1.76--1.08)
Serbia	Male	1.13(0.57-2.14)	1.69(0.94-2.97)	1.97(1.75-2.19)	43.98(29.54-67.84)	59.08(39.03-90.32)	1.51(1.34-1.68)	0.61(0.35-0.99)	0.52(0.28-0.87)	-1.22(-1.49--0.95)	21.76(13.09-34.11)	18.11(11.08-28.52)	-1.23(-1.45--1.01)
Seychelles	Both	0.93(0.37-2.17)	1.26(0.47-2.93)	1.23(1.13-1.33)	35.72(24.25-55.12)	39.94(25.58-64.04)	0.6(0.49-0.7)	2.72(1.85-3.77)	3.58(2.47-4.97)	0.43(0.1-0.77)	104.85(74.42-140.49)	135.65(98.43-182.76)	0.4(0.1-0.71)
Seychelles	Female	0.77(0.25-2.02)	1.09(0.32-2.84)	1.28(1.21-1.36)	36.06(22.77-56.8)	41.05(25.08-68.32)	0.6(0.51-0.7)	2.23(1.51-3.07)	3.08(2.11-4.45)	0.94(0.64-1.24)	87.96(62.25-117.54)	115.23(81.3-160.05)	0.87(0.63-1.1)
Seychelles	Male	1.09(0.43-2.5)	1.41(0.54-3.38)	1.16(1.04-1.29)	35.39(22.73-55.69)	39.22(24.5-64.07)	0.63(0.51-0.74)	3.24(2.16-4.6)	4(2.71-5.69)	-0.02(-0.4-0.37)	122.07(85.52-168.84)	153.03(107.62-209.89)	0.02(-0.37-0.41)
Sierra Leone	Both	1.73(0.87-3.46)	1.98(0.95-4.07)	0.71(0.56-0.86)	82.71(55.61-119.43)	79.49(52.15-118.24)	0.16(0.03-0.3)	1.42(0.84-2.22)	1.4(0.85-2.2)	0.23(0.09-0.37)	50.86(31.28-79.32)	52.46(33-80.55)	0.43(0.28-0.59)
Sierra Leone	Female	1.39(0.59-3.01)	1.52(0.61-3.44)	0.49(0.31-0.67)	82.29(51.53-123.56)	76.53(46.93-120.63)	-0.06(-0.22-0.1)	1.3(0.74-2.14)	1.38(0.81-2.18)	0.49(0.33-0.65)	48.17(28.7-76.55)	52.8(30.81-81.92)	0.65(0.47-0.83)
Sierra Leone	Male	2.08(1-4.32)	2.44(1.12-5.05)	0.83(0.71-0.96)	83.11(50.65-129.18)	82.21(50.92-129.15)	0.39(0.25-0.53)	1.53(0.9-2.4)	1.41(0.82-2.26)	0.03(-0.11-0.16)	53.32(33.06-81.76)	52.15(31.49-80.64)	0.24(0.09-0.39)
Singapore	Both	1.26(0.6-2.67)	1.4(0.78-2.63)	0.96(0.76-1.16)	72.29(48.77-104.38)	79.71(55.34-111.77)	0.87(0.71-1.04)	1.06(0.69-1.51)	0.5(0.32-0.76)	-0.75(-1.37--0.13)	39.12(26.69-54.04)	19.36(12.83-27.5)	-0.65(-1.21--0.1)
Singapore	Female	0.77(0.33-1.77)	1(0.48-2.02)	1.27(1.13-1.42)	73.36(45.5-109.65)	89.68(56.99-130.93)	1.03(0.9-1.16)	1.03(0.68-1.49)	0.5(0.33-0.78)	-0.57(-1.24-0.1)	37.63(25.73-52.06)	18.81(12.6-26.74)	-0.57(-1.14-0.01)
Singapore	Male	1.73(0.8-3.69)	1.78(0.9-3.33)	0.77(0.55-1)	71.37(45.75-110.93)	69.93(46.77-103.6)	0.7(0.47-0.93)	1.1(0.71-1.57)	0.49(0.31-0.74)	-0.97(-1.55--0.38)	40.78(27.68-56.84)	19.78(12.77-28.76)	-0.78(-1.32--0.24)
Slovakia	Both	1.09(0.52-2.32)	1.79(0.97-3.4)	2.16(1.99-2.33)	47.09(30.91-73.01)	78.59(52.97-117.7)	2.26(2.1-2.43)	0.53(0.32-0.82)	0.26(0.15-0.43)	-1.26(-1.82--0.7)	20.39(12.56-30.82)	11.15(7.08-16.77)	-1.02(-1.49--0.54)
Slovakia	Female	0.76(0.32-1.68)	1.36(0.63-2.86)	2.4(2.23-2.57)	48.28(29.21-77.95)	88.26(54.18-138.89)	2.45(2.3-2.6)	0.4(0.24-0.63)	0.23(0.13-0.38)	-0.91(-1.29--0.54)	14.6(9.02-21.6)	9.43(5.92-14.1)	-0.6(-0.91--0.3)
Slovakia	Male	1.43(0.66-2.95)	2.23(1.2-4.24)	2.04(1.86-2.21)	46.1(28.54-76.38)	69.82(44.55-108.59)	2.08(1.88-2.27)	0.69(0.41-1.06)	0.3(0.17-0.49)	-1.54(-2.25--0.82)	26.69(16.18-40.8)	12.91(8.08-19.54)	-1.31(-1.92--0.7)
Slovenia	Both	0.82(0.38-1.67)	1.5(0.81-2.75)	2.75(2.47-3.03)	43.06(28.84-63.13)	73.38(50.4-106.37)	2.41(2.21-2.62)	0.1(0.06-0.16)	0.06(0.03-0.11)	-1.29(-1.54--1.03)	4.31(2.84-6.13)	4.18(2.78-5.99)	0.06(-0.07-0.2)
Slovenia	Female	0.63(0.26-1.39)	1.2(0.54-2.44)	2.83(2.54-3.12)	47.1(28.57-75.23)	85.21(52.56-131.83)	2.5(2.31-2.69)	0.09(0.05-0.14)	0.06(0.03-0.1)	-1.05(-1.34--0.76)	3.58(2.32-5.21)	3.79(2.48-5.58)	0.3(0.13-0.47)
Slovenia	Male	1.03(0.46-2.1)	1.81(0.94-3.25)	2.67(2.4-2.95)	39.27(25.03-60.69)	62.59(40.51-94.98)	2.34(2.11-2.57)	0.12(0.07-0.19)	0.07(0.04-0.12)	-1.41(-1.67--1.14)	5.1(3.33-7.4)	4.58(2.95-6.71)	-0.14(-0.28-0.01)
Solomon Islands	Both	1.24(0.38-3.2)	1.34(0.39-3.51)	0.26(0.2-0.32)	30.13(19.22-50.28)	32.43(19.93-56.08)	0.37(0.3-0.44)	6.32(3.99-9.45)	3.74(2.53-5.21)	-1.93(-2.36--1.5)	280.76(182.87-409.78)	169.33(116.32-229.55)	-1.86(-2.28--1.43)
Solomon Islands	Female	1.16(0.3-3.34)	1.25(0.31-3.48)	0.2(0.15-0.24)	30.09(18.74-49.83)	32.75(19.62-56.86)	0.39(0.33-0.46)	7.07(4.31-10.98)	3.76(2.48-5.27)	-2.32(-2.81--1.82)	315.14(193.49-482.74)	170.13(112.62-240.88)	-2.25(-2.74--1.75)
Solomon Islands	Male	1.3(0.4-3.32)	1.42(0.42-3.56)	0.32(0.25-0.4)	30.13(18.63-52.62)	32.06(18.88-58.56)	0.34(0.27-0.42)	5.63(3.53-8.81)	3.73(2.52-5.31)	-1.52(-1.9--1.14)	248.73(162.46-382.83)	168.67(115.73-235.31)	-1.44(-1.81--1.06)
Somalia	Both	0.97(0.42-2.11)	1.09(0.46-2.38)	0.8(0.6-1)	41.99(28.78-63.07)	47.91(32.29-73.08)	1.03(0.79-1.27)	1.59(0.92-2.56)	1.46(0.83-2.31)	-0.2(-0.24--0.17)	55.14(33.24-84.73)	50.9(29.84-80.2)	-0.18(-0.22--0.14)
Somalia	Female	0.62(0.24-1.47)	0.73(0.28-1.69)	0.96(0.73-1.19)	41.52(26.56-64.08)	51.6(33.17-80.53)	1.26(1.02-1.5)	1.38(0.75-2.26)	1.3(0.72-2.22)	0.02(-0.06-0.1)	50.1(28.35-80.33)	46.11(26.35-75.09)	-0.07(-0.14-0)

Somalia	Male	1.31(0.55-2.92)	1.44(0.57-3.24)	0.78(0.6-0.97)	42.22(26.53-66.55)	44.2(27.81-69.27)	0.8(0.56-1.04)	1.84(1.03-3.04)	1.69(0.95-2.76)	-0.31(-0.37--0.26)	60.82(35.91-97.76)	57.04(33.12-90.02)	-0.23(-0.29--0.18)
South Africa	Both	1.59(1.17-2.18)	1.94(1.43-2.63)	0.97(0.69-1.25)	53.91(45.28-63.89)	65.67(54.59-78.89)	1.09(0.88-1.31)	1.05(0.68-1.53)	1.24(0.78-1.86)	1.31(0.91-1.72)	43.47(28.75-63.73)	46.64(30.56-67.69)	0.87(0.43-1.3)
South Africa	Female	0.99(0.68-1.42)	1.27(0.86-1.85)	1.05(0.73-1.36)	45.45(37.27-54.51)	60.92(49.25-74.41)	1.34(1.1-1.58)	0.86(0.56-1.25)	0.94(0.59-1.41)	1.16(0.76-1.57)	36.49(24.02-52.98)	34.93(23.21-51.38)	0.58(0.1-1.07)
South Africa	Male	2.25(1.69-3.03)	2.66(1.96-3.69)	0.91(0.66-1.15)	63.2(52.15-77.51)	71.19(58.68-87.98)	0.89(0.7-1.08)	1.28(0.8-1.9)	1.61(1-2.39)	1.43(0.97-1.9)	51.68(33.82-75)	59.96(38.44-87.11)	1.07(0.63-1.52)
South Korea	Both	1.23(0.66-2.28)	1.17(0.68-2.06)	0.43(0.25-0.62)	64.49(43.24-95.72)	65.79(46.04-93.69)	0.36(0.22-0.5)	0.9(0.61-1.24)	0.46(0.31-0.66)	-2.19(-2.28--2.09)	34.29(24.06-45.4)	16.58(11.51-22.3)	-2.34(-2.44--2.24)
South Korea	Female	0.85(0.4-1.66)	0.81(0.4-1.49)	0.4(0.14-0.65)	71.01(43.73-113.68)	73.48(47.18-111.34)	0.29(0.13-0.44)	0.68(0.47-0.95)	0.34(0.24-0.51)	-2.49(-2.69--2.29)	26.73(18.9-35.5)	12.57(8.84-17.23)	-2.59(-2.72--2.45)
South Korea	Male	1.61(0.84-2.98)	1.52(0.84-2.76)	0.42(0.25-0.59)	58.33(36.79-89.86)	59.08(39.3-85.71)	0.47(0.32-0.62)	1.16(0.77-1.61)	0.59(0.39-0.84)	-2.04(-2.14--1.95)	42.74(29.77-56.8)	20.62(13.94-27.84)	-2.23(-2.34--2.12)
South Sudan	Both	0.92(0.45-1.9)	1.07(0.52-2.19)	0.86(0.71-1.01)	42.9(28.55-63.9)	51.11(34.09-76.13)	1.07(0.91-1.23)	1.19(0.66-2)	1.11(0.61-1.83)	-0.29(-0.36--0.22)	39.32(22.85-62.53)	36.86(21.16-59.76)	-0.26(-0.36--0.17)
South Sudan	Female	0.57(0.24-1.28)	0.72(0.31-1.57)	1.12(0.94-1.3)	42.8(26.47-66.38)	54.61(33.89-86.39)	1.25(1.09-1.4)	0.99(0.55-1.73)	0.99(0.53-1.66)	0.04(-0.08-0.15)	34.33(18.84-58.23)	33.66(18.99-54.51)	-0.01(-0.15-0.13)
South Sudan	Male	1.23(0.57-2.55)	1.41(0.63-3.02)	0.82(0.69-0.96)	43.02(26.56-65.04)	46.66(28.16-71.21)	0.81(0.64-0.98)	1.33(0.72-2.31)	1.21(0.63-2.14)	-0.44(-0.51--0.37)	43(24.42-70.05)	39.76(21.86-68.06)	-0.36(-0.45--0.28)
Spain	Both	0.98(0.57-1.66)	1.23(0.84-1.85)	1.54(1.13-1.96)	46.06(31.51-66.71)	62.64(44.96-86.78)	1.32(1.09-1.56)	0.33(0.19-0.52)	0.16(0.09-0.27)	-2.75(-2.94--2.56)	10.05(6.74-14.57)	5.43(3.75-7.53)	-2.16(-2.41--1.91)
Spain	Female	0.75(0.38-1.35)	0.97(0.55-1.63)	1.26(0.96-1.56)	51.24(32.62-77.85)	72.14(47.44-106.54)	1.04(0.85-1.24)	0.28(0.16-0.46)	0.14(0.08-0.24)	-2.85(-3.16--2.53)	8.45(5.73-12.39)	4.81(3.28-6.79)	-2.19(-2.49--1.89)
Spain	Male	1.22(0.66-2.14)	1.51(0.94-2.38)	1.76(1.26-2.27)	40.92(26.67-60.75)	53.34(37.44-76.4)	1.78(1.36-2.21)	0.38(0.23-0.59)	0.18(0.1-0.3)	-2.69(-2.81--2.58)	11.79(7.9-17)	6.1(4.1-8.69)	-2.15(-2.37--1.93)
Sri Lanka	Both	0.97(0.34-2.4)	1.25(0.43-3.18)	1.04(0.91-1.18)	35.86(23.9-56.69)	40.65(25.97-67.33)	0.66(0.53-0.8)	2.31(1.62-3.11)	2.05(1.3-3.15)	-0.25(-0.5-0.01)	93.42(67.44-122.19)	80.09(53.35-117.87)	-0.43(-0.65--0.21)
Sri Lanka	Female	0.72(0.21-1.85)	0.97(0.27-2.58)	1.13(0.99-1.26)	34.29(21.83-54.78)	39.5(24.66-66.08)	0.66(0.51-0.8)	1.62(1.11-2.23)	1.44(0.9-2.21)	-0.23(-0.52-0.05)	66.7(47.33-89.18)	55.27(35.9-82.57)	-0.51(-0.71--0.31)
Sri Lanka	Male	1.22(0.44-2.95)	1.53(0.54-3.92)	1.02(0.88-1.16)	37.4(23.63-62.09)	41.8(25.65-74.38)	0.67(0.54-0.81)	2.99(2.06-4.16)	2.73(1.7-4.23)	-0.16(-0.42-0.11)	120(85.27-161.32)	107.27(70.17-161.25)	-0.3(-0.56--0.05)
Sudan	Both	1.07(0.53-2.23)	2.04(1.07-4.09)	2.37(2.16-2.57)	34.64(22.7-53.11)	59.52(38.42-93.55)	2.03(1.86-2.2)	0.85(0.49-1.35)	0.79(0.41-1.39)	-0.58(-0.87--0.28)	31(18.22-47.87)	29(16.36-48.97)	-0.5(-0.79--0.2)
Sudan	Female	0.87(0.34-1.99)	1.55(0.64-3.48)	2.13(1.94-2.32)	34.77(20.85-56.04)	59.48(35.22-98.26)	1.95(1.77-2.12)	0.77(0.42-1.32)	0.72(0.36-1.27)	-0.53(-0.84--0.22)	29.54(16.55-48.51)	27.3(14.7-47.11)	-0.52(-0.82--0.21)
Sudan	Male	1.27(0.61-2.59)	2.49(1.27-4.85)	2.49(2.27-2.71)	34.32(21.13-54.61)	58.76(36.17-93.82)	2.08(1.9-2.25)	0.92(0.52-1.47)	0.84(0.41-1.61)	-0.63(-0.92--0.34)	32.09(19-50.74)	30.25(16.27-56)	-0.5(-0.78--0.21)
Suriname	Both	1.83(0.57-5.02)	3.11(0.96-8.01)	1.9(1.84-1.97)	31.29(19.65-54.51)	43.15(25.81-77.66)	1.21(1.16-1.26)	1.76(1.16-2.52)	2.76(1.74-4.15)	1.45(1.16-1.73)	66.73(44.61-92.28)	102.93(66.98-150.16)	1.34(1.09-1.6)
Suriname	Female	1.37(0.33-3.95)	2.39(0.61-6.81)	2.03(1.98-2.08)	27.88(16.83-49.61)	39.18(22.78-70.92)	1.27(1.21-1.32)	1.49(0.97-2.16)	2.44(1.57-3.55)	1.63(1.33-1.92)	56.47(37.06-79.35)	93.29(61.97-133.89)	1.61(1.35-1.88)
Suriname	Male	2.3(0.71-5.89)	3.82(1.2-9.74)	1.81(1.73-1.9)	34.75(20.01-63.24)	47.09(26.35-88.66)	1.14(1.09-1.19)	2.05(1.35-2.94)	3.12(1.89-4.76)	1.33(1.04-1.62)	77.65(51.13-108.29)	113.32(72.06-169.56)	1.14(0.87-1.4)
Sweden	Both	1.24(0.88-1.76)	1.21(0.88-1.67)	0.48(0.21-0.75)	90(68.47-117.84)	96.22(72.84-126.08)	0.73(0.37-1.1)	0.14(0.09-0.22)	0.19(0.12-0.31)	1.22(0.41-2.04)	6.79(4.77-9.16)	7.43(5.29-10.16)	0.61(0.16-1.06)
Sweden	Female	0.87(0.53-1.4)	0.83(0.52-1.29)	0.54(0.28-0.8)	71.96(52.35-98)	87.87(62.64-123.3)	1.12(0.88-1.36)	0.11(0.07-0.16)	0.15(0.1-0.24)	1.62(0.7-2.55)	5.3(3.8-7.12)	5.95(4.26-8.01)	0.78(0.33-1.24)
Sweden	Male	1.64(1.08-2.38)	1.6(1.07-2.3)	0.4(0.07-0.73)	108.45(75.21-154.17)	104.76(74.89-148.49)	0.44(-0.02-0.9)	0.19(0.12-0.29)	0.24(0.14-0.38)	0.82(0.06-1.58)	8.43(5.93-11.65)	8.95(6.3-12.47)	0.44(-0.02-0.89)
Switzerland	Both	1.28(0.8-2.1)	1.74(1.13-2.63)	1.72(1.46-1.97)	64.5(46.01-89.32)	85.95(60.83-116.44)	1.57(1.27-1.87)	0.11(0.07-0.18)	0.13(0.07-0.21)	-2.77(-3.85--1.68)	5.32(3.62-7.35)	5.61(3.88-7.74)	-1.02(-1.43--0.6)
Switzerland	Female	1.07(0.57-1.93)	1.42(0.79-2.48)	1.68(1.46-1.9)	70.46(47.39-105.63)	97.02(64.76-142.36)	1.63(1.35-1.91)	0.08(0.05-0.14)	0.11(0.06-0.19)	-2.55(-3.76--1.31)	4.56(3.03-6.49)	5.24(3.55-7.27)	-0.61(-1.02--0.2)
Switzerland	Male	1.51(0.92-2.52)	2.07(1.29-3.27)	1.71(1.43-2)	59.18(39.3-86.81)	75.5(51-106.83)	1.47(1.15-1.8)	0.15(0.09-0.24)	0.14(0.08-0.24)	-3.03(-3.99--2.05)	6.22(4.13-8.73)	6.01(4.02-8.65)	-1.41(-1.83--0.99)
Syria	Both	1.72(0.79-3.82)	2.77(1.51-5.21)	2.13(1.92-2.34)	44.49(28.74-73.76)	77.39(52.93-117.89)	2.42(2.24-2.6)	1.28(0.78-1.95)	0.74(0.44-1.19)	-2.61(-3.03--2.18)	49.66(31.24-75.26)	28.88(17.8-45.29)	-2.53(-2.96--2.1)
Syria	Female	1.44(0.55-3.33)	2.19(0.99-4.57)	1.92(1.7-2.14)	43.67(27.46-72.86)	78.12(49.64-123.24)	2.5(2.32-2.67)	1.17(0.72-1.82)	0.68(0.4-1.1)	-2.57(-3.02--2.12)	47.4(29.14-72.92)	26.69(16.24-41.33)	-2.61(-3.07--2.15)
Syria	Male	1.98(0.91-4.41)	3.36(1.81-6.42)	2.29(2.1-2.49)	45.22(27.48-79.61)	74.66(48.84-120.74)	2.27(2.07-2.47)	1.37(0.82-2.13)	0.8(0.47-1.33)	-2.62(-3.03--2.22)	51.67(31.37-79.21)	30.74(19.11-49.03)	-2.49(-2.89--2.08)
Taiwan	Both	0.87(0.39-1.73)	1.54(0.76-2.93)	2.47(2.32-2.62)	46.15(34.52-63.37)	89.24(67.76-118.13)	2.74(2.61-2.87)	1.54(1.05-2.12)	0.95(0.57-1.45)	-1.66(-1.91--1.41)	60.64(42.8-80.44)	46.08(29.96-65.11)	-0.79(-1.01--0.58)
Taiwan	Female	0.57(0.2-1.35)	1.23(0.5-2.66)	3.17(3.02-3.31)	47.09(33.77-64.37)	99.21(71.58-139.16)	3.05(2.92-3.19)	1.53(1.03-2.15)	0.78(0.47-1.22)	-2.41(-2.67--2.16)	59.14(41.03-79.16)	38.87(25.38-55.2)	-1.35(-1.56--1.14)
Taiwan	Male	1.14(0.52-2.28)	1.85(0.89-3.52)	2.16(2.01-2.31)	45.16(32.35-64.76)	79.65(58.38-108.45)	2.4(2.28-2.52)	1.55(1.06-2.12)	1.12(0.67-1.76)	-1.02(-1.27--0.76)	62.24(43.6-82.87)	53.59(34.73-77.26)	-0.31(-0.55--0.08)
Tajikistan	Both	1.28(0.53-2.95)	1.87(0.75-4.35)	1.71(1.41-2.02)	56.97(38.87-86.02)	70.14(45.54-110.06)	1.11(0.92-1.31)	0.12(0.08-0.19)	0.25(0.15-0.39)	2.09(1.69-2.49)	7.14(4.66-10.5)	12.35(7.91-19.13)	1.5(1.15-1.86)
Tajikistan	Female	1.11(0.34-2.87)	1.54(0.48-4.01)	1.4(1.14-1.67)	55.44(34.36-86.43)	70.91(41.49-113.93)	1.12(0.95-1.29)	0.14(0.08-0.22)	0.24(0.14-0.38)	1.22(0.77-1.68)	8.01(4.96-12.09)	11.78(7.33-18.43)	0.77(0.35-1.18)
Tajikistan	Male	1.46(0.64-3.18)	2.2(0.95-4.89)	1.93(1.59-2.27)	58.56(36.84-90.79)	69.57(43.06-112.14)	1.12(0.9-1.34)	0.11(0.07-0.17)	0.27(0.17-0.43)	2.97(2.61-3.33)	6.3(4.17-9.15)	12.99(8.15-19.95)	2.3(2.01-2.6)
Tanzania	Both	1.13(0.57-2.16)	1.21(0.59-2.34)	0.35(0.12-0.59)	57.79(40.19-81)	61.55(41.75-87.69)	0.51(0.31-0.71)	0.94(0.55-1.47)	0.87(0.53-1.33)	-0.35(-0.43--0.27)	32.01(20.1-48.27)	29.45(19.34-42.23)	-0.39(-0.47--0.3)
Tanzania	Female	0.77(0.34-1.56)	0.88(0.38-1.8)	0.53(0.23-0.84)	57.8(36.7-86.06)	65.66(41.59-97.8)	0.64(0.45-0.83)	0.84(0.49-1.36)	0.83(0.5-1.26)	0(-0.13-0.14)	29.15(17.24-45.67)	28.09(17.97-42.06)	-0.12(-0.27-0.03)
Tanzania	Male	1.51(0.72-2.87)	1.56(0.71-3.05)	0.25(0.05-0.45)	57.54(36.2-85.41)	56.67(35.78-84.66)	0.35(0.14-0.57)	1.04(0.61-1.67)	0.91(0.55-1.41)	-0.65(-0.78--0.53)	35.02(21.18-52.9)	30.91(20.39-45.84)	-0.62(-0.74--0.51)

Thailand	Both	1.44(0.44-3.49)	1.56(0.57-3.55)	0.88(0.58-1.18)	47.86(30.75-76.95)	54.29(35.31-85.14)	1.45(1.07-1.84)	2.97(2.11-4)	2.24(1.39-3.41)	-1.44(-1.75--1.13)	128.07(93.05-167.69)	88.35(57.89-129.74)	-1.98(-2.4--1.56)
Thailand	Female	1(0.27-2.83)	1.16(0.34-3.11)	1.05(0.8-1.31)	44.81(27.38-73.81)	53.65(33.07-88.25)	1.66(1.26-2.06)	2.02(1.36-2.8)	1.93(1.17-3.03)	-0.09(-0.56-0.39)	82.84(57.8-111.83)	72.66(45.5-112.53)	-0.51(-1.06-0.05)
Thailand	Male	1.88(0.58-4.49)	1.97(0.72-4.4)	0.81(0.47-1.14)	50.87(31.36-84.42)	54.91(34.61-89.52)	1.27(0.88-1.66)	3.97(2.8-5.45)	2.57(1.64-3.94)	-2.33(-2.8--1.85)	175.38(124.65-240.19)	104.54(69.8-152.31)	-2.85(-3.44--2.27)
Timor-Leste	Both	0.92(0.36-1.99)	1.17(0.44-2.57)	1.19(1.04-1.34)	38.95(26.32-58.8)	44.7(29.43-70.01)	0.94(0.77-1.12)	3.1(1.87-4.74)	2.68(1.53-4.18)	-0.59(-0.92--0.26)	121.66(75.75-181.76)	101.58(52.79-153.19)	-0.73(-1.1--0.36)
Timor-Leste	Female	0.78(0.26-1.84)	0.95(0.32-2.27)	0.99(0.84-1.14)	39.68(24.8-61.84)	46.35(27.96-74.28)	0.99(0.8-1.17)	3.5(2.13-5.2)	2.62(1.42-3.95)	-1.26(-1.62--0.89)	137.18(84.74-204.19)	99.46(50.48-148.07)	-1.39(-1.8--0.98)
Timor-Leste	Male	1.05(0.41-2.34)	1.39(0.51-3.17)	1.33(1.17-1.48)	38.25(24.03-59.85)	42.82(26.13-71.03)	0.88(0.7-1.05)	2.71(1.53-4.88)	2.74(1.42-4.52)	0.13(-0.18-0.44)	107.02(62.13-182.74)	103.66(51.02-168.49)	-0.03(-0.36-0.3)
Tobago	Both	1.05(0.37-2.42)	1.74(0.65-3.94)	1.93(1.84-2.01)	28.75(17.29-48.55)	44.01(26.68-74.34)	1.73(1.64-1.82)	1.2(0.8-1.72)	1.97(1.1-3.23)	1.92(1.6-2.24)	45.27(31.04-63.12)	72.92(42.87-117.55)	1.8(1.49-2.11)
Tobago	Female	0.75(0.22-1.87)	1.23(0.4-3.06)	1.92(1.83-2)	25.07(15.12-42.06)	39.85(24.27-66.2)	1.84(1.75-1.93)	0.94(0.61-1.36)	1.64(0.91-2.72)	2.15(1.77-2.53)	36.51(24.64-51.65)	62.13(35.84-100.55)	1.97(1.6-2.34)
Tobago	Male	1.36(0.47-3.1)	2.22(0.87-5.07)	1.92(1.83-2)	32.4(17.97-56.46)	48.07(26.86-84.48)	1.64(1.55-1.72)	1.49(0.98-2.14)	2.31(1.31-3.79)	1.74(1.45-2.03)	54.32(37.27-76.47)	83.74(50.37-134.21)	1.66(1.37-1.96)
Togo	Both	1.72(0.82-3.42)	2.09(1.02-4.15)	1.11(0.9-1.32)	69.32(47.2-100)	78.99(53.04-114.48)	1.04(0.81-1.26)	1.65(1.01-2.65)	1.53(0.91-2.35)	-0.21(-0.28--0.14)	60.14(37.7-93.58)	56.72(35.46-83.7)	-0.08(-0.17-0)
Togo	Female	1.32(0.56-2.81)	1.56(0.68-3.34)	1.02(0.79-1.26)	68.96(44.22-103.67)	80.43(52.1-123.33)	1.08(0.86-1.3)	1.62(0.94-2.75)	1.29(0.74-2.03)	-0.7(-0.91--0.5)	60.44(35.51-102.03)	49.1(28.91-74.76)	-0.6(-0.83--0.37)
Togo	Male	2.13(0.96-4.43)	2.64(1.23-5.52)	1.18(0.99-1.37)	69.46(43.84-108.08)	76.87(48.7-120.47)	0.97(0.75-1.2)	1.69(1.04-2.7)	1.83(1.09-2.87)	0.35(0.17-0.54)	59.44(37.26-91.51)	65.99(41.07-99.27)	0.47(0.29-0.66)
Tonga	Both	1(0.32-2.74)	1.38(0.44-3.79)	1.16(1.12-1.21)	32.33(21.13-55.04)	39.16(24.48-69.97)	0.85(0.77-0.92)	2.55(1.63-3.99)	3.61(2.2-5.72)	1.12(0.76-1.48)	100.63(65.34-153.68)	141.6(87.66-223.06)	1.11(0.76-1.45)
Tonga	Female	0.86(0.22-2.48)	1.1(0.29-3.2)	0.93(0.87-0.98)	31.18(19.35-53.04)	37.82(23.11-65.02)	0.87(0.78-0.96)	2.75(1.72-4.43)	3(1.76-4.86)	-0.19(-0.62-0.23)	108.93(68.68-170.27)	117.91(71.12-193.12)	-0.23(-0.64-0.19)
Tonga	Male	1.14(0.38-2.98)	1.64(0.52-4.32)	1.31(1.27-1.36)	33.59(21.19-58.21)	40.3(24.1-75.39)	0.79(0.73-0.86)	2.34(1.44-3.77)	4.27(2.49-6.96)	2.45(2.06-2.83)	91.66(57.64-145.29)	167.46(100.75-269.64)	2.45(2.08-2.82)
Trinidad	Both	1.05(0.37-2.42)	1.74(0.65-3.94)	1.93(1.84-2.01)	28.75(17.29-48.55)	44.01(26.68-74.34)	1.73(1.64-1.82)	1.2(0.8-1.72)	1.97(1.1-3.23)	1.92(1.6-2.24)	45.27(31.04-63.12)	72.92(42.87-117.55)	1.8(1.49-2.11)
Trinidad	Female	0.75(0.22-1.87)	1.23(0.4-3.06)	1.92(1.83-2)	25.07(15.12-42.06)	39.85(24.27-66.2)	1.84(1.75-1.93)	0.94(0.61-1.36)	1.64(0.91-2.72)	2.15(1.77-2.53)	36.51(24.64-51.65)	62.13(35.84-100.55)	1.97(1.6-2.34)
Trinidad	Male	1.36(0.47-3.1)	2.22(0.87-5.07)	1.92(1.83-2)	32.4(17.97-56.46)	48.07(26.86-84.48)	1.64(1.55-1.72)	1.49(0.98-2.14)	2.31(1.31-3.79)	1.74(1.45-2.03)	54.32(37.27-76.47)	83.74(50.37-134.21)	1.66(1.37-1.96)
Tunisia	Both	1.18(0.57-2.43)	2.39(1.29-4.46)	2.76(2.62-2.9)	37.58(24.32-60.33)	67.97(45.26-103.28)	2.37(2.26-2.47)	0.57(0.35-0.87)	0.53(0.3-0.89)	-0.22(-0.33--0.11)	20.81(13.16-31.76)	20.8(13.11-32.41)	0.01(-0.1-0.11)
Tunisia	Female	1.04(0.4-2.29)	1.94(0.85-3.96)	2.58(2.45-2.71)	39.84(24.15-64.35)	70.99(43.54-111.11)	2.38(2.26-2.5)	0.52(0.33-0.81)	0.48(0.26-0.79)	-0.26(-0.42--0.11)	20.09(12.81-29.99)	19.21(11.88-29.77)	-0.08(-0.23-0.06)
Tunisia	Male	1.31(0.61-2.55)	2.84(1.49-5.09)	2.91(2.73-3.09)	35.31(21.8-57.33)	64.6(40.68-102.62)	2.33(2.2-2.46)	0.61(0.36-0.97)	0.59(0.32-1)	-0.15(-0.26--0.05)	21.47(12.92-33.07)	22.43(13.43-35.28)	0.1(0.01-0.19)
Turkey	Both	1.3(0.61-2.66)	2.87(1.62-5.05)	2.37(2.16-2.58)	45.19(30.49-69.29)	88.1(61.95-128.51)	1.83(1.59-2.07)	1.16(0.69-1.78)	0.6(0.37-0.9)	-2.75(-3.03--2.47)	42.24(26.84-61.95)	23.41(15.46-33.42)	-2.44(-2.67--2.2)
Turkey	Female	0.97(0.36-2.24)	2.08(0.97-4.35)	2.32(2.08-2.56)	44.34(28.51-70.45)	90.08(58.67-136.1)	1.81(1.53-2.1)	0.89(0.53-1.41)	0.48(0.3-0.72)	-2.68(-3.04--2.31)	33.56(20.99-51.33)	18.79(12.39-27.11)	-2.42(-2.67--2.17)
Turkey	Male	1.63(0.79-3.32)	3.67(2.09-6.39)	2.41(2.21-2.62)	46.12(28.8-73.15)	86.97(56.7-132.58)	1.86(1.66-2.07)	1.44(0.86-2.28)	0.73(0.45-1.1)	-2.8(-3.02--2.57)	51.17(32-77.51)	28.15(18.28-40.45)	-2.45(-2.66--2.23)
Turkmenistan	Both	1.93(0.8-4.44)	2.92(1.17-6.91)	1.81(1.6-2.02)	67.95(45.58-100.25)	86.33(56.95-134)	1.21(0.99-1.43)	0.53(0.32-0.82)	0.72(0.43-1.16)	0.62(0.34-0.9)	25.17(16.08-37.71)	33.92(21.28-53.66)	0.73(0.49-0.97)
Turkmenistan	Female	1.55(0.48-4.05)	2.23(0.69-5.73)	1.51(1.33-1.68)	62.99(39.34-100.51)	80.09(48.74-130)	1.03(0.84-1.23)	0.49(0.3-0.74)	0.52(0.31-0.85)	-0.11(-0.36-0.13)	23.44(14.82-35.14)	25.38(15.95-40.08)	0.01(-0.23-0.26)
Turkmenistan	Male	2.34(0.98-5.2)	3.59(1.52-8.2)	1.96(1.73-2.2)	73.41(46.13-113.14)	93.14(58.82-148.39)	1.37(1.13-1.62)	0.58(0.35-0.9)	0.92(0.54-1.48)	1.13(0.78-1.48)	27.12(17.21-40.69)	42.49(26.71-65.53)	1.24(0.96-1.53)
Uganda	Both	0.73(0.34-1.55)	0.89(0.4-1.94)	1.04(0.83-1.26)	37.62(26.45-55.26)	44.01(30.49-67.2)	1(0.8-1.2)	1.1(0.64-1.77)	1.1(0.68-1.7)	-0.44(-0.69--0.18)	36.02(22.04-56.4)	37.11(22.84-55.36)	-0.38(-0.66--0.1)
Uganda	Female	0.43(0.17-1.01)	0.54(0.23-1.23)	1.15(0.89-1.42)	37.61(24.44-59.77)	47.25(30.59-76.25)	1.18(0.98-1.38)	0.77(0.42-1.34)	0.83(0.47-1.39)	-0.15(-0.45-0.15)	25.82(14.61-44.18)	28.14(16.51-43.31)	-0.2(-0.54-0.14)
Uganda	Male	1.04(0.45-2.26)	1.25(0.53-2.82)	1.01(0.83-1.2)	37.54(23.94-59.27)	40.04(25.26-62.75)	0.75(0.55-0.96)	1.45(0.83-2.35)	1.44(0.85-2.29)	-0.48(-0.73--0.23)	46.57(27.72-73.56)	47.65(28.91-71.94)	-0.4(-0.67--0.13)
UK	Both	1.11(0.96-1.29)	1.19(1.02-1.37)	0.2(0.11-0.3)	49.49(44.25-55.54)	47.09(41.58-53.05)	-0.44(-0.58--0.3)	0.14(0.08-0.22)	0.11(0.07-0.17)	-0.3(-0.7-0.09)	6.12(4.23-8.44)	5.24(3.62-7.11)	-0.25(-0.41--0.08)
UK	Female	0.8(0.68-0.93)	0.85(0.72-1)	0.21(0.11-0.31)	50.29(44.42-56.65)	49.74(43.17-56.92)	-0.48(-0.69--0.28)	0.12(0.07-0.19)	0.1(0.06-0.16)	-0.06(-0.43-0.3)	5.37(3.74-7.42)	4.87(3.35-6.6)	-0.18(-0.31--0.05)
UK	Male	1.43(1.23-1.67)	1.53(1.3-1.77)	0.18(0.07-0.28)	48.68(43.19-55.46)	44.53(39.16-50.33)	-0.39(-0.47--0.3)	0.16(0.1-0.26)	0.12(0.07-0.18)	-0.62(-1.07--0.16)	6.95(4.81-9.61)	5.64(3.88-7.74)	-0.34(-0.57--0.11)
Ukraine	Both	1.04(0.5-2.11)	1.79(0.93-3.49)	2.5(2.15-2.86)	60.31(43.62-82.13)	103.36(73.02-145.2)	2.55(2.18-2.91)	0.12(0.08-0.18)	0.27(0.18-0.39)	3.63(3.02-4.25)	4.5(3.23-6.11)	11.44(7.94-16.24)	4.36(3.7-5.03)
Ukraine	Female	0.84(0.36-1.79)	1.37(0.61-2.74)	2.17(1.72-2.63)	61.53(40.52-92.51)	116.81(74.79-181.94)	2.86(2.46-3.27)	0.11(0.07-0.16)	0.2(0.13-0.3)	3.22(2.65-3.8)	3.81(2.76-5.18)	8.6(5.78-12.34)	3.97(3.31-4.64)
Ukraine	Male	1.25(0.61-2.51)	2.24(1.14-4.32)	2.74(2.43-3.05)	58.96(38.11-88.43)	89.95(58.51-131.52)	2.19(1.88-2.51)	0.15(0.1-0.22)	0.35(0.22-0.52)	3.75(3.12-4.39)	5.45(3.85-7.51)	14.77(10.08-22.16)	4.56(3.89-5.23)
United Arab Emirates	Both	1.71(0.76-3.58)	3.58(1.91-6.59)	2.54(2.34-2.74)	44.8(28.55-71.16)	85.99(57.72-133.05)	2.25(2.02-2.48)	1.95(1.16-3.04)	1.69(0.93-3.07)	-0.5(-0.75--0.25)	66.84(40.84-102.32)	63.3(35.26-113.6)	-0.2(-0.28--0.12)
United Arab Emirates	Female	1.27(0.46-3.04)	2.51(1.11-5.24)	2.35(2.19-2.5)	42.99(26.23-72.77)	84.96(54.7-135.6)	2.32(2.08-2.56)	1.73(0.95-2.91)	1.51(0.78-2.46)	-0.22(-0.68-0.24)	59.47(33.58-97.92)	56.07(30.33-88.58)	-0.14(-0.27-0)
United Arab Emirates	Male	1.94(0.86-4.1)	4.03(2.13-7.57)	2.5(2.3-2.7)	46.06(28.42-75.58)	86.94(56.23-139.03)	2.23(2.01-2.44)	2.07(1.18-3.33)	1.75(0.91-3.4)	-0.72(-0.9--0.55)	70.03(41.57-110.76)	65.3(35.59-127.03)	-0.31(-0.37--0.24)
Uruguay	Both	2.92(1.93-4.28)	2.09(1.27-3.6)	-0.65(-1.01--0.28)	165.59(119.51-224.14)	124.39(86.88-175.24)	-0.53(-0.85--0.21)	0.43(0.29-0.62)	0.56(0.37-0.8)	1.36(1.12-1.6)	18.42(13.4-24.23)	21.68(15.61-29.18)	1.06(0.82-1.3)

Uruguay	Female	1.95(1.05-3.28)	1.66(0.87-3.03)	-0.03(-0.28-0.21)	169.34(110.6-248.87)	145.2(92.73-215.72)	-0.13(-0.37-0.11)	0.3(0.2-0.43)	0.41(0.28-0.59)	1.64(1.37-1.9)	13.52(9.83-17.74)	16.99(12.36-22.65)	1.32(1.07-1.56)
Uruguay	Male	3.99(2.5-6.15)	2.56(1.47-4.53)	-1.02(-1.49--0.54)	162.31(111.88-237.98)	102.37(67.81-155.08)	-1.04(-1.49--0.59)	0.6(0.39-0.87)	0.74(0.48-1.07)	1.2(0.96-1.44)	24(17.26-32.34)	27.1(19.05-36.79)	0.9(0.65-1.14)
USA	Both	2.24(1.91-2.63)	2.21(1.91-2.55)	-0.24(-0.38--0.11)	96.53(85.79-108)	98.5(88.19-109.27)	0.03(-0.03-0.08)	0.2(0.12-0.3)	0.46(0.29-0.7)	3.11(2.87-3.35)	10.45(7.09-14.54)	18.48(12.24-25.58)	1.87(1.64-2.09)
USA	Female	1.56(1.3-1.87)	1.49(1.26-1.76)	-0.41(-0.59--0.24)	98.8(86.45-111.87)	101.04(88.62-114.42)	0.08(0.04-0.12)	0.18(0.11-0.27)	0.39(0.25-0.59)	3.11(2.82-3.4)	9.09(6.23-12.61)	15.68(10.51-21.68)	1.87(1.57-2.17)
USA	Male	2.95(2.49-3.55)	2.93(2.52-3.45)	-0.18(-0.3--0.07)	94.37(83.34-106.61)	95.98(84.89-108.12)	-0.04(-0.11-0.03)	0.22(0.14-0.33)	0.54(0.33-0.81)	3.09(2.88-3.31)	11.88(7.99-16.83)	21.47(14.03-30.08)	1.86(1.69-2.03)
Uzbekistan	Both	2.37(1.04-5.03)	3.08(1.24-6.54)	1.21(0.96-1.47)	77.02(53.04-111.99)	91.45(60.15-139.41)	0.95(0.63-1.26)	0.54(0.32-0.84)	0.69(0.43-1.05)	0.24(-0.41-0.9)	25.31(15.95-38.74)	32.55(21.04-47.69)	0.26(-0.25-0.77)
Uzbekistan	Female	2.06(0.73-4.68)	2.52(0.83-5.82)	0.91(0.67-1.16)	76.35(49.72-116.85)	90.72(57.04-146.26)	0.83(0.52-1.14)	0.47(0.26-0.8)	0.57(0.36-0.87)	0.11(-0.53-0.75)	22.56(13.7-36.21)	27.58(17.79-40.81)	0.09(-0.43-0.61)
Uzbekistan	Male	2.7(1.19-5.64)	3.66(1.52-7.81)	1.43(1.17-1.7)	77.74(49.76-117.76)	92.65(57.08-146.16)	1.08(0.76-1.4)	0.61(0.36-0.94)	0.82(0.5-1.26)	0.4(-0.27-1.08)	28.2(17.73-42.17)	37.98(24.12-55.89)	0.43(-0.09-0.95)
Vanuatu	Both	0.83(0.27-2.15)	1.12(0.36-3)	1.01(0.92-1.1)	28.12(18.41-45.05)	32.44(20.25-55.27)	0.6(0.54-0.66)	2.42(1.41-3.9)	4.25(2.57-6.6)	1.95(1.79-2.11)	98.9(59.07-153.19)	174.18(106.18-261.44)	1.93(1.76-2.1)
Vanuatu	Female	0.75(0.2-1.99)	0.98(0.25-2.62)	0.9(0.81-0.98)	28.66(17.83-45.44)	32.89(19.94-54.74)	0.56(0.5-0.63)	2.57(1.44-4.48)	4.22(2.38-6.92)	1.48(1.26-1.69)	102.81(57.49-174.09)	170.59(94.66-279.45)	1.48(1.25-1.7)
Vanuatu	Male	0.9(0.31-2.25)	1.24(0.41-3.06)	1.11(1.02-1.2)	27.51(17.48-45.71)	31.81(19.28-56.93)	0.63(0.57-0.7)	2.31(1.34-3.71)	4.3(2.65-6.62)	2.41(2.23-2.58)	96.13(55.91-151.34)	178.41(111.55-265.24)	2.37(2.2-2.54)
Venezuela	Both	1.24(0.51-2.8)	1.74(0.72-3.81)	1.35(1.19-1.51)	35.36(22.05-56.77)	43.72(26.72-72.69)	0.99(0.81-1.17)	0.98(0.63-1.4)	2.09(1.19-3.41)	2.01(1.47-2.55)	36.09(24.16-49.74)	71.28(42.46-115.01)	1.73(1.25-2.22)
Venezuela	Female	0.78(0.27-1.83)	1.12(0.38-2.7)	1.46(1.33-1.59)	32.48(19.55-52.39)	39.59(23.95-65.34)	0.9(0.71-1.09)	0.84(0.55-1.2)	1.74(0.99-2.85)	1.9(1.38-2.41)	31.88(21.66-43.53)	60.53(36.42-97.64)	1.66(1.21-2.12)
Venezuela	Male	1.71(0.68-3.73)	2.35(0.99-5.11)	1.29(1.12-1.47)	38.21(22.46-66.88)	47.74(27.59-83.95)	1.06(0.88-1.25)	1.12(0.72-1.64)	2.48(1.38-4.09)	2.09(1.49-2.7)	40.55(26.62-57.21)	82.68(48.07-134.78)	1.78(1.23-2.34)
Vietnam	Both	0.76(0.28-1.78)	1.06(0.4-2.47)	1.41(1.3-1.51)	37.58(24.66-56.9)	46.66(30.35-71.38)	1.04(0.89-1.2)	2.42(1.57-3.59)	1.94(1.29-2.82)	-0.75(-1.29--0.21)	95.33(61.96-139.3)	75.68(51.53-107.34)	-0.72(-1.23--0.2)
Vietnam	Female	0.69(0.22-1.71)	0.93(0.29-2.26)	1.2(1.11-1.29)	38.17(23.81-59.33)	50.06(30.78-79.64)	1.19(1.04-1.33)	2.76(1.75-4.12)	1.94(1.28-2.85)	-1.34(-1.98--0.7)	110.19(70.21-164.58)	76.71(52.43-107.79)	-1.3(-1.9--0.7)
Vietnam	Male	0.83(0.3-1.91)	1.19(0.45-2.79)	1.54(1.42-1.66)	37.06(23.47-59.55)	43.4(27.05-70.33)	0.89(0.72-1.07)	2(1.23-3.09)	1.93(1.25-2.85)	0.05(-0.34-0.44)	78.14(49.25-117.48)	74.79(48.59-107.72)	0.07(-0.31-0.46)
Virgin Islands	Both	2.44(1.43-4.28)	2.34(1.38-4.03)	-0.36(-0.5--0.22)	107.21(74.57-149.78)	107.42(77.06-147.95)	-0.11(-0.17--0.04)	0.22(0.14-0.34)	0.5(0.31-0.8)	2.8(2.46-3.15)	11.32(7.48-15.98)	19.74(12.85-28.59)	1.67(1.36-1.97)
Virgin Islands	Female	1.72(0.88-3.13)	1.58(0.82-2.81)	-0.61(-0.8--0.42)	110.52(70.78-164.58)	110.68(72.68-161.04)	-0.1(-0.16--0.03)	0.21(0.12-0.32)	0.42(0.25-0.68)	2.75(2.4-3.1)	10.01(6.71-13.94)	16.76(10.65-24.88)	1.62(1.26-1.98)
Virgin Islands	Male	3.2(1.77-5.64)	3.12(1.7-5.51)	-0.26(-0.38--0.13)	104.15(68.9-153.78)	104.16(71.5-154.73)	-0.13(-0.2--0.05)	0.24(0.15-0.37)	0.59(0.35-0.95)	2.85(2.48-3.22)	12.68(8.27-18.22)	22.95(14.53-34.12)	1.7(1.43-1.98)
Yemen	Both	1.3(0.68-2.45)	2.38(1.31-4.39)	2.61(2.37-2.86)	44.61(30.64-67.4)	75.47(51.47-115.95)	2.4(2.08-2.73)	0.82(0.46-1.4)	0.71(0.4-1.15)	-0.6(-0.7--0.49)	30.37(17.02-50.78)	27.68(16.39-43.36)	-0.36(-0.48--0.25)
Yemen	Female	1.02(0.43-2.26)	1.84(0.79-4.01)	2.49(2.24-2.74)	45.66(29.03-71.31)	78.54(49.62-126.55)	2.4(2.08-2.73)	0.69(0.35-1.26)	0.61(0.33-1.04)	-0.48(-0.67--0.3)	26.6(13.95-47.5)	25.1(14.55-41.84)	-0.23(-0.41--0.04)
Yemen	Male	1.56(0.8-2.97)	2.92(1.62-5.19)	2.72(2.47-2.97)	43.38(27.56-67.09)	72.39(46.22-111.37)	2.42(2.09-2.75)	0.97(0.54-1.64)	0.81(0.46-1.34)	-0.71(-0.78--0.64)	34.06(19.16-56.05)	30.33(17.91-47.79)	-0.45(-0.53--0.38)
Zambia	Both	1.09(0.51-2.24)	1.38(0.66-2.78)	1.21(1.02-1.41)	42.75(29.42-61.07)	56.81(39.15-81.69)	1.55(1.34-1.77)	1.53(0.94-2.31)	1.46(0.88-2.23)	-0.53(-0.81--0.25)	53.63(33.59-78.88)	52.03(32.2-76.99)	-0.47(-0.74--0.2)
Zambia	Female	0.75(0.29-1.73)	0.96(0.42-2.05)	1.28(1.06-1.49)	41.76(26.02-63.2)	61.36(39.22-93.7)	1.89(1.67-2.11)	1.58(0.99-2.39)	1.31(0.76-2.05)	-1.1(-1.3--0.89)	58.08(36.72-87.52)	47.72(28.68-72.21)	-1.13(-1.34--0.91)
Zambia	Male	1.44(0.65-3.02)	1.81(0.83-3.84)	1.18(1-1.37)	43.8(27.5-66.26)	51.99(32.38-79.42)	1.17(0.95-1.39)	1.45(0.85-2.28)	1.63(0.98-2.5)	0.11(-0.3-0.52)	48.53(29.19-72.74)	56.67(35.65-84.77)	0.26(-0.14-0.66)
Zimbabwe	Both	1.25(0.64-2.54)	1.39(0.68-2.9)	0.41(-0.04-0.87)	51.18(34.33-81.67)	59.3(38.42-98.85)	0.72(0.41-1.03)	0.88(0.49-1.63)	1.59(0.85-2.72)	2.46(1.99-2.93)	30.92(17.83-57.02)	56.86(31.45-94.83)	2.59(2.14-3.05)
Zimbabwe	Female	0.74(0.29-1.71)	0.91(0.34-2.17)	0.61(0.17-1.05)	45.08(28.08-70.97)	53.82(32.75-88.95)	0.82(0.52-1.11)	0.64(0.31-1.47)	1.17(0.56-2.57)	3.59(2.87-4.32)	23.08(12.12-50.08)	42.29(21.2-88)	3.72(2.96-4.48)
Zimbabwe	Male	1.78(0.89-3.57)	1.94(0.95-4.02)	0.43(-0.02-0.88)	57.39(35.97-92.13)	65.37(39.27-112.3)	0.67(0.35-1)	1.12(0.61-2.01)	2.13(1.16-3.49)	2.12(1.48-2.78)	38.85(22.26-68.44)	74.81(40.49-120.41)	2.23(1.6-2.87)

Abbreviation: ASIR, age-standardized incidence rate; ASPR, age-standardized prevalence rate; ASDR, age-standardized death rate; CI, confidence interval; DALY, disability adjusted life-year; EAPC, estimated annual percentage change; UI, uncertainty interval

Table S5. The incidence, prevalence, deaths, DALYs and their change percent of type 2 diabetes related CKD in 204 countries and territories.

Location	Sex	Incident cases (95%UI)		Change percent (%) (95%UI)	Prevalent cases (95%UI)		Change percent (%) (95%UI)	Deaths (95%UI)		Change percent (%) (95%UI)	DALYs (95%UI)		Change percent (%) (95%UI)
		1990	2019		1990	2019		1990	2019		1990	2019	
Afghanistan	Both	2330.62(2031.87-2670.26)	6083.88(5339.25-6989.98)	-25.67(-31.66--19.49)	99175.82(90079.13-109616.63)	248537.18(220694.02-278873.48)	-23.93(-27.49--19.84)	1122.32(754.26-1705.63)	1595.25(1113.36-2415.81)	3.03(-21.17-31.45)	28085.08(19306.45-40574.77)	43544.85(30559.33-61668.85)	6.08(-24.57-42.62)
Afghanistan	Female	1180.69(1008.95-1376.51)	3104.3(2685.97-3614.86)	-23.01(-29.5--15.34)	52877.81(47196.93-59131.92)	131718.66(116734.96-147717.75)	-21.89(-25.27--18.3)	524.69(348.4-861.18)	866.22(571.79-1372.68)	15.74(-21.41-58.27)	14067.83(9479.33-21494.74)	24588.16(16679.64-38083.77)	20.61(-21.36-72.42)
Afghanistan	Male	1149.93(1008.27-1323.01)	2979.58(2587.61-3401.1)	-28.26(-35.24--20.79)	46298.01(41900.12-51172.61)	116818.52(103358.47-133386.04)	-25.88(-30.69--20.65)	597.63(388.25-929.62)	729.03(472.36-1134.21)	-8.71(-29.98-18.86)	14017.25(9099.01-20931.1)	18956.7(12643.81-27568.13)	-8.23(-33.91-27.73)
Albania	Both	336.87(292.46-386.45)	1133.11(986.73-1281.18)	345.01(299.78-393.69)	28429.99(25128.65-31985.68)	48923.11(44582.38-53424.8)	106.39(91.27-121.99)	27.75(19.15-38.18)	47.21(28.77-70.73)	30.98(7.05-54.57)	822.09(600.57-1066.2)	1437.26(1045.24-1980.37)	118.99(85.64-154.77)
Albania	Female	156.78(134.94-181.84)	514.13(446.45-590.82)	319.03(263.61-382.76)	13910.04(12080.91-15759.52)	24287.16(21932.18-26763.64)	103.23(86.34-120.59)	13.23(9.1-18.23)	23.77(14.65-36.13)	35.84(6.07-67.43)	390.46(284.3-512.34)	713.1(522.74-978.95)	125.57(87.91-169.57)
Albania	Male	180.1(154.61-209.76)	618.98(530.57-710.79)	371.19(318.86-431.95)	14519.95(12784.07-16389.44)	24635.94(22394.21-26984.1)	109.64(94.57-127.39)	14.52(9.72-20.1)	23.44(13.69-36.28)	26(-2.33-56.13)	431.63(307.06-564.74)	724.16(508.85-1001.3)	112.6(72.94-153.77)
Algeria	Both	4624.77(4026.93-5284.79)	21233.54(18647.33-24141.23)	180.87(163.59-200.88)	204596.05(182446.08-229226.95)	630690.82(572815.11-692254.52)	84.35(74.02-95.31)	1019.77(705.13-1504.2)	2532.67(1771.82-3567.63)	89.99(53.38-138.51)	24802.85(17526.04-35989.51)	59538.04(43839.09-81257.01)	130.71(83.67-188.31)
Algeria	Female	2581.01(2222.07-3008.17)	10234.94(8908.26-11608.68)	141.62(124.43-162.51)	110274.88(97132.21-125216.03)	321393.7(289123.04-354118.33)	74.83(64.25-85.61)	524.47(335.5-892.59)	1388.83(935.08-2110.67)	91.57(45.33-153.71)	13319.35(8952.23-21863.87)	32628.87(23339.42-47651.64)	120.72(65.51-190.07)
Algeria	Male	2043.76(1754.41-2351.73)	10998.6(9527.11-12611.7)	230.57(201.88-259.93)	94321.17(83650.87-106263.61)	309297.12(280077.75-341121.54)	95.5(82.72-108.98)	495.3(350.68-706.97)	1143.84(786.15-1656.38)	85.11(42.18-147.31)	11483.5(8273.21-15943.56)	26909.17(19229.88-37517.38)	139.08(84-213.81)
American Samoa	Both	6.79(5.87-7.84)	19.84(17.38-22.46)	139.16(122.24-157.12)	666.34(574.11-773.55)	1135.57(1026.02-1259.76)	46.4(35.73-57.9)	2.68(2.14-3.34)	8.93(7.04-11.06)	94.86(66.43-129.84)	77.45(61-97.21)	233.38(182.31-291.01)	129.73(98.79-167.94)
American Samoa	Female	2.79(2.35-3.32)	8.82(7.63-10.2)	157.18(129.32-187.93)	333.07(285.84-392.46)	580.48(519.36-648.49)	46.67(33.35-60.39)	1.04(0.8-1.36)	4.12(3.13-5.41)	96.42(59.49-135.96)	30.52(23.69-39.19)	108.68(83.05-140.54)	148.22(105.1-195.78)
American Samoa	Male	4(3.45-4.65)	11.01(9.59-12.57)	126.85(107.67-147.55)	333.27(285.35-384.87)	555.09(497.24-617.09)	46.05(36.56-56.82)	1.64(1.25-2.1)	4.81(3.8-5.86)	92.47(55.89-142.58)	46.93(35.16-60.98)	124.7(97.37-155.05)	117.46(79.66-168.2)
Andorra	Both	16.57(14.39-18.89)	42.32(37.35-47.46)	70.94(58.26-84.13)	528.78(478.11-585.84)	1198.7(1091.56-1303.58)	45.21(38.26-52.83)	0.81(0.53-1.25)	2.96(1.89-4.43)	45.37(10.25-95.87)	19.13(13.74-26.63)	49.59(35.74-67.07)	44.15(20.03-71.73)
Andorra	Female	8.2(7.04-9.51)	21.07(18.52-23.58)	66.76(49.72-82.44)	241.16(215.9-269.56)	571.99(517.21-625.67)	47.23(38.8-56.43)	0.39(0.24-0.64)	1.69(1.03-2.66)	34.09(-5.21-86.4)	9.07(6.35-13.13)	26.53(18.49-36.99)	49.11(16.03-87.79)
Andorra	Male	8.37(7.11-9.7)	21.25(18.4-24.2)	75.18(58.61-94.24)	287.62(258-320.39)	626.72(565.9-688.24)	43.82(36.38-52.31)	0.42(0.27-0.64)	1.27(0.83-1.87)	40.95(3.42-101.2)	10.06(7.12-14.1)	23.07(16.84-30.83)	36.54(10.15-70.13)
Angola	Both	419.24(371.16-479.32)	1728.21(1532.21-1978.81)	48.12(39.53-56.76)	59746.01(51670.14-70072.45)	171607.12(149948.55-196488.96)	0.14(-4.18-5.21)	193.78(127.97-261.63)	523.14(339.1-735.25)	149.39(93.67-222.16)	5228.23(3488.09-7241.83)	13808.1(9209.87-19374.52)	167.02(110.68-245.61)
Angola	Female	174.12(150.56-202.07)	846.16(732.02-985.47)	70.99(59.37-83.99)	28747.65(24584.21-33696.88)	87474.4(76034.86-100624.17)	1.58(-4.53-8.4)	65.57(24.36-103.86)	216.63(87.21-331.63)	193.21(117.77-315.01)	1842.78(754.62-2908.53)	5773.55(2639.76-8774.63)	205.79(129.51-324.67)
Angola	Male	245.12(215.44-278.51)	882.05(768.68-1015.28)	32.16(21.74-43.42)	30998.37(26700.58-36205.06)	84132.72(72764.68-97521.48)	-1.03(-5.72-4.15)	128.2(84.1-175.78)	306.51(211.67-418.61)	128.21(66.73-226.84)	3385.45(2234.4-4677.05)	8034.55(5611.3-10992.47)	147.45(80.38-248.56)
Antigua and Barbuda	Both	13.96(12.35-15.63)	44.33(38.85-49.84)	112.34(97.77-128.43)	811.37(726.99-905.38)	1838.28(1652.13-2028.85)	54.35(45.4-64.11)	4.13(3.31-5)	10.46(8.08-13.16)	78.94(56.6-104.57)	92.78(74.6-110.75)	251.71(195.82-311.07)	84.7(63.02-111.03)
Antigua and Barbuda	Female	6.92(6.07-7.82)	21(18.23-23.74)	105.85(85.78-126.07)	416.33(369.34-469.77)	891.11(795.6-995.91)	47.58(37.54-58.76)	1.68(1.33-2.08)	5.21(3.99-6.49)	109.89(76.03-148.51)	37.18(30.08-45.45)	123.81(96.4-154.9)	116.94(84.94-152.3)
Antigua and Barbuda	Male	7.05(6.1-8.04)	23.34(20.34-26.56)	118.18(98.5-142.39)	395.04(353.02-444.67)	947.17(848.59-1053.93)	61.24(51.62-73.21)	2.45(1.92-3.03)	5.25(4-6.71)	57.42(35.62-83.07)	55.6(43.95-67.77)	127.9(97.85-161.11)	63.18(42.41-88.11)
Argentina	Both	9383.09(8226.4-10487.37)	20261.58(18181.95-22531.68)	58.7(45.52-73.99)	346824.36(314597.4-377782.89)	635258.67(583222.07-690114.82)	35.24(29.66-41.25)	2070.59(1610.98-2572.35)	4639.3(3586.9-5831.04)	61.76(41.45-84.42)	45877.91(35902.98-55324.05)	87630.06(68842.53-106326.31)	60.26(44.55-78.31)
Argentina	Female	4794.49(4178.95-5428.02)	10456.7(9196.07-11901.81)	59.51(41.66-81.55)	185191.79(166888.47-204170.62)	340810.23(309102.26-373650.36)	34.86(27.63-43.38)	942.33(725.76-1181.1)	2298.09(1749.81-2939.22)	62.25(38.89-90.51)	20143.11(15799.11-24404.66)	40696.52(32006.79-49997.29)	62.02(43.19-82.84)
Argentina	Male	4588.6(3986.15-5194.9)	9804.88(8663.73-11198.52)	57.88(43-75.04)	161632.57(145122.8-177480.31)	294448.45(268380.67-322375.18)	35.61(29.34-42.11)	1128.26(866.05-1400.79)	2341.21(1798.77-2950.32)	60.74(39.16-86.36)	25734.81(19807.24-31173.66)	46933.54(36415.9-57636.61)	59.23(42.35-80.3)
Armenia	Both	320.42(270.25-380.57)	927.24(806.38-1069.22)	219.59(186.96-257.78)	47775.99(41725.89-54271.1)	65815.83(59288.06-72112.6)	54.87(45.87-64.74)	21.02(16.44-26.28)	156.48(118.26-200.62)	530.72(453.18-621.79)	683.1(546.55-829.96)	3563.49(2776.19-4422.5)	548.66(460.51-653.79)
Armenia	Female	178.84(150.15-212.65)	465.7(400.95-536.79)	181.44(145.85-219.84)	24752.17(21415.5-28257.43)	34145.43(30595.1-37636.47)	52.5(42.1-62.77)	9.61(7.42-11.91)	73.25(55.41-94.05)	521.32(429.23-625.39)	324.25(261.09-399.91)	1550.32(1228.8-1937.09)	469.4(380.54-572.05)
Armenia	Male	141.58(118.02-170.44)	461.55(394.59-543.02)	268.61(225.25-316.56)	23023.82(19968.16-26418.55)	31670.4(28363.03-34963.54)	57.5(47.26-68.62)	11.42(8.8-14.4)	83.23(62.21-106.34)	540.16(457.37-645.84)	358.85(282.05-440.02)	2013.16(1534.78-2518.02)	624.27(520.3-749.06)
Australia	Both	7005.16(6491.86-7575.83)	16522.45(14834.23-18270.3)	62.4(50.57-75.36)	191703.55(177557.32-205790.34)	393979.24(364211-424105.26)	40.89(34.02-48.3)	86.97(63.96-117.58)	454.24(302.97-665.07)	259.7(173.22-363.33)	2990.5(2337.18-3815.57)	10069.59(7530.67-13085.36)	152.71(104.84-213.47)
Australia	Female	3848.03(3527.82-4205.92)	8274.25(7435.69-9203.09)	47.5(35.87-60.97)	107955.82(99343.14-117356.28)	207893.71(192040.4-224644.78)	31.12(23.77-38.5)	49.6(35.22-69.03)	231.64(148.32-350.19)	208.33(135.6-293.37)	1702.52(1306.41-2191.07)	4940.36(3706.54-6492.45)	106.07(68.8-155.23)
Australia	Male	3157.13(2823.51-3530.08)	8248.2(7122.67-9368.1)	80.62(60.11-104.14)	83747.74(76483.43-91812.04)	186085.54(169906.69-203883.7)	53.44(43.61-64.16)	37.36(28.89-48.96)	222.6(145.65-326.35)	325.86(207.54-473.9)	1287.98(1009.77-1661.54)	5129.22(3793.26-6769.9)	214.19(145.46-308.98)
Austria	Both	3585.17(3102.91-4090.62)	6584.33(5811.69-7449.61)	62.83(49.9-77.77)	101007.79(91887.74-109979.42)	157381.75(144191.9-170058.48)	34.68(28.01-41.01)	158.58(113.93-216.54)	761.1(525.66-1044.38)	375.65(280.18-482.55)	3615.28(2763.65-4584.92)	10712.08(8237.21-13885.92)	204.32(161.68-250.87)

Austria	Female	2273.25(1958.45-2603.16)	3540.39(3124.57-3990.29)	40.97(29.19-54.96)	58001.54(52559.71-63481.44)	81781.5(74468.08-89121.14)	25.39(19.4-32.09)	101.92(71.97-140.13)	474.97(314.49-668.06)	381.27(270.61-507.37)	2176.12(1667.96-2795.28)	6027.77(4514.76-7937.48)	186.54(137.15-240.47)
Austria	Male	1311.93(1126.31-1518.69)	3043.94(2634.68-3488)	101.03(79.06-126.91)	43006.25(38844.15-47367.48)	75600.25(68776.08-82378.73)	47.25(38.15-56.33)	56.66(40.26-77.79)	286.12(201.25-393)	378.19(290.01-479.27)	1439.16(1073-1822.61)	4684.31(3528.42-6086.92)	231.9(181.88-288.96)
Azerbaijan	Both	659.53(558.16-782.39)	2434.24(2077.94-2820.92)	151.59(129.26-179.28)	91738.41(80279.51-104368.63)	184071.91(163484.9-205256.45)	43.12(35.42-51.72)	128.55(97.58-163.59)	439.1(328.52-560.87)	163.36(114.87-216.61)	4027.8(3082.56-5008.14)	12682.8(9771-15908.02)	219.26(166.45-281.02)
Azerbaijan	Female	374.77(316.71-447.48)	1234.98(1054.08-1442.23)	127.69(104.25-156.46)	48523.55(42009.76-55693.88)	91981.92(80879.36-104058.47)	38.14(29.34-47.57)	63.07(47.03-83.74)	222.4(160.83-291.25)	175.67(102.94-244.48)	1891.59(1474.51-2416.47)	6178.63(4678.64-7996.33)	229.1(161.56-307.53)
Azerbaijan	Male	284.76(235.7-341.9)	1199.26(1010.9-1410.26)	183.03(150.52-220.92)	43214.86(36942.9-49556.74)	92089.99(81246.64-103257.03)	48.59(39.79-58.28)	65.49(46.32-83.93)	216.7(149.19-299.65)	152(96.47-220.23)	2136.21(1540.61-2759.93)	6504.16(4643.59-8752.84)	209.95(150.18-287.62)
Bahamas	Both	39(34.19-44.17)	143.39(126.82-162.13)	141.65(126.28-159.7)	2765.04(2451.27-3157)	6443.16(5866.74-7075.18)	57.13(46.83-67.91)	9.55(7.56-11.63)	29.9(22.28-38.18)	63.71(46.56-84.12)	270.37(213.55-325.56)	821.23(621.55-1039.1)	83.62(64.2-104.88)
Bahamas	Female	19.15(16.67-22.01)	70.77(61.62-81.04)	140.18(117.89-165.32)	1425.51(1250.18-1647.08)	3236.63(2907.6-3584.64)	51.12(40.07-62.67)	4.55(3.55-5.62)	14.67(11.05-19.01)	69.94(46.62-97.14)	127.16(100.81-154.6)	393.15(297.85-500.33)	88.14(63.54-115.89)
Bahamas	Male	19.84(17.04-22.87)	72.61(62.99-83.01)	143.4(121.86-167.16)	1339.53(1177.79-1525.15)	3206.53(2901.44-3529.91)	63.7(51.99-75.84)	5(3.92-6.14)	15.23(11.13-19.5)	58.29(39.53-80.77)	143.21(111.28-175.23)	428.08(317.86-543.53)	79.75(58.17-102.79)
Bahrain	Both	77.52(68.03-88.6)	826.32(702.73-966.58)	282.52(250.66-315.07)	4319.71(3729.06-5028.81)	23297.15(20962.3-25947.74)	87.73(71.36-105.32)	14.88(11.32-19.18)	61.67(45.48-80.38)	90.7(58.05-121.23)	381.85(295.73-474.71)	1775.78(1344.84-2239.09)	102.76(67.05-142.5)
Bahrain	Female	37.13(32.53-42.52)	276.66(239.23-318.92)	194.39(169.47-220.49)	1860.35(1616.47-2176.3)	8653.73(7737.85-9661.7)	80.69(64.19-96.16)	7.38(5.38-9.72)	26.53(19.07-34.89)	69.2(30.09-112.91)	187.7(140.73-239.94)	699.14(521.1-897.15)	74.37(33.03-118.13)
Bahrain	Male	40.39(34.74-46.69)	549.67(461.38-651.75)	355.3(309.7-403.92)	2459.37(2097.87-2873.39)	14643.42(13041.16-16404.21)	92.41(73.32-113.81)	7.5(5.46-10.38)	35.14(24.5-48.2)	112.48(73.18-156.49)	194.16(145.67-260.57)	1076.64(793.89-1401.85)	130.22(86-184.79)
Bangladesh	Both	5608.21(4800.05-6433.89)	24345.42(20957.33-27864.37)	223.67(200.82-250.3)	775595.85(676040.5-902643.52)	1865175.99(1669692.61-2076795.26)	65.99(55.59-77.92)	1371.98(970.04-1837.73)	3388.38(2354.27-4663.48)	226.89(164.41-321.67)	36630.28(26356.99-48402.09)	92073.25(67201.27-122065.87)	350.86(262.28-472.24)
Bangladesh	Female	2127.83(1847.29-2423.02)	10214.93(8872.55-11771.17)	247.99(218.5-282.82)	318039.77(272791.96-376117.47)	800646.77(711515.6-899900.29)	65.76(53.14-79.07)	498.21(293.55-704.33)	1504.07(1041.07-2068.73)	310.51(200.54-528.68)	12013.5(7693.47-16606.83)	35988.88(26488.25-47389.83)	427.34(292.09-655.54)
Bangladesh	Male	3480.38(2899.93-4043.73)	14130.49(11829.24-16506.31)	211.73(184.68-240.68)	457556.08(398304.41-534580.96)	1064529.22(947280.3-1196072.13)	68.35(57.86-79.94)	873.77(572.59-1202.56)	1884.31(1283.25-2645.44)	179.49(118.83-272.49)	24616.78(16911.02-33523.64)	56084.37(40075.77-75387.7)	316.02(223.23-457.18)
Barbados	Both	66.09(57.85-74.5)	178.38(158.59-203.75)	116.06(100.36-133.28)	4003.05(3619.75-4427.14)	7129.06(6555.54-7756.42)	50.72(41.8-59.23)	15.79(12.67-19.12)	36.62(28.03-46.06)	69.73(49.77-90.5)	355.55(293.28-418.83)	844.94(664.68-1044.92)	87.41(66.8-108.78)
Barbados	Female	34.89(30.32-39.94)	90.36(79.77-103.21)	107.44(86.69-126.91)	2128.83(1911.03-2367.04)	3618.73(3296.46-3948.42)	44.32(35.64-53.29)	7.69(6.16-9.4)	19.26(14.81-24.21)	88(62.3-113.63)	171.4(141.11-202.95)	437.29(342-541.34)	98.74(73.97-124.56)
Barbados	Male	31.2(26.67-35.96)	88.02(75.93-101.95)	125.69(101.62-151.59)	1874.22(1687.29-2107.44)	3510.33(3193.01-3869.42)	57.95(47.11-68.08)	8.11(6.47-9.79)	17.36(13.18-21.95)	52.72(33.89-72.88)	184.15(150.72-217.66)	407.65(315.81-510.2)	76.8(58.05-99.86)
Belarus	Both	1554.49(1350.1-1808.05)	2462.28(2156.98-2812.86)	74.17(58.13-93.23)	211476.9(187902.33-236054.13)	234641.77(211856.4-257294.25)	21.91(15.9-28.34)	36.39(25.59-51.77)	61.98(41.1-90.36)	52.31(30.91-76.47)	1436.56(1092.44-1846.24)	2307.65(1717.29-3047.21)	68.98(47.49-92.49)
Belarus	Female	980.81(848.6-1147.49)	1305.4(1125.4-1505.58)	44.32(27.03-64.8)	116289.45(103317.42-130621.72)	125666.44(113003.95-138897.8)	17.9(11.38-24.32)	21.39(14.75-30.84)	36.11(23.95-53.7)	51.83(26.22-81.87)	842.69(643.68-1092.86)	1221.58(904.41-1604.08)	54.37(33.61-77.89)
Belarus	Male	573.68(489.16-677.18)	1156.88(991.46-1332.18)	125.45(99.11-154.03)	95187.45(82637.4-108004.84)	108975.33(97365.65-121405.62)	26.84(19.93-34.27)	15(10.37-20.96)	25.86(16.76-37.53)	53.32(28.74-80.32)	593.87(442.55-769.23)	1086.06(792.38-1472.78)	90.4(59.85-122.94)
Belgium	Both	5038.49(4472.64-5624.22)	7641.54(6904.5-8429.76)	29.99(16.99-43.16)	137058.21(125651.97-148802.32)	192684.88(177933.36-207516.97)	22.16(15.56-28.66)	278.44(198.77-392.23)	518.09(348.71-771.03)	68.21(34.21-108.76)	5998.17(4575.48-7710.94)	8578.73(6428.66-11361.46)	38.9(21.14-58.97)
Belgium	Female	3019.6(2656.23-3388.41)	4152.16(3672.3-4663.28)	18.1(5.13-33.47)	75840.95(69226.81-82946.67)	101289.24(92148.11-110356.81)	16.89(8.97-24.64)	174.01(123.04-246.78)	315.75(203.39-474.86)	59.24(24.03-103.49)	3523.93(2680.56-4551.44)	4746.66(3522.33-6309.22)	26.05(7.62-49.12)
Belgium	Male	2018.89(1769.39-2330.6)	3489.38(3078.49-3909.68)	47.79(30.15-68.04)	61217.27(55681.37-67191.14)	91395.64(83688.98-99520.58)	28.72(21.78-36.41)	104.43(73.95-146.11)	202.34(139.95-299.52)	80.44(45.67-121.98)	2474.24(1832.69-3204.24)	3832.07(2862.5-5083.07)	55.88(34.45-80.54)
Belize	Both	22.23(19.16-25.56)	105.39(92.09-118.7)	115.4(94.95-137.88)	1542.31(1366.2-1756.01)	5075.88(4579.91-5599.3)	48.93(41.61-56.76)	5.59(4.47-6.84)	29.11(23.08-36.34)	138.26(110.62-173.6)	139.22(111.01-168.1)	793.78(632.13-979.5)	195.11(158.05-238.19)
Belize	Female	10.46(9.04-12.02)	49.11(42.68-56.54)	111.05(88.53-136.31)	741.83(649.03-848.35)	2414.1(2151.63-2689.82)	44.4(35.53-53.65)	2.89(2.26-3.6)	13.43(10.6-16.68)	155.15(119.17-202.33)	70.05(55.47-85.89)	371.23(293.93-456.84)	196.76(154.89-249.08)
Belize	Male	11.77(10.07-13.7)	56.29(48.71-64.49)	119.64(96.34-146.54)	800.48(708.35-914.37)	2661.78(2390.92-2966.72)	53.49(44.71-61.94)	2.7(2.13-3.32)	15.68(12.32-19.55)	132.37(99.99-171.63)	69.17(54.57-84.36)	422.56(330.48-528.17)	197.01(155.66-248.36)
Benin	Both	340.33(302.02-382.2)	1093.74(957.7-1243.27)	27.09(19.97-34.35)	26676.4(23276.46-30505.48)	73946.47(65055.74-84026.93)	7.93(4.07-11.96)	159.55(117.95-207.49)	352.07(252.09-484.55)	58.61(26.5-98.73)	3589.05(2645.46-4608.65)	8548.43(6036.99-11776.66)	78.12(42.74-118.48)
Benin	Female	170.28(148.21-193.94)	569.03(495.02-649.88)	32.99(23.23-43.76)	14010.17(12117.74-16153.64)	37705.24(33238.16-42805.64)	5.24(0.12-10.73)	69.53(50.04-91.54)	166.87(108.62-231.75)	75.07(29.58-129.97)	1626.65(1184.57-2156.22)	4044.69(2690.11-5757.45)	89.16(44.35-145.31)
Benin	Male	170.05(148.42-191.77)	524.71(455.98-602.73)	21.21(12.12-31.93)	12666.23(10993.5-14582.58)	36241.23(31528.92-41569.05)	10.9(6.72-15.52)	90.02(64.88-121.28)	185.2(124.87-264.44)	46.01(9.54-97.01)	1962.4(1406.14-2633.49)	4503.74(3020.75-6562.78)	69.04(26.13-124.73)
Bermuda	Both	15.24(13.38-17.25)	48.19(42.9-54.14)	170.83(151.45-193.66)	914.66(819.09-1019.87)	1738.14(1596.42-1879.12)	75.19(63.07-86.92)	2.76(2.15-3.4)	5.54(4.15-7.12)	69.57(46.45-96)	69.8(55.66-84.1)	128.46(103.11-158.78)	80.71(59.23-105.8)
Bermuda	Female	7.54(6.57-8.64)	23.51(20.57-26.67)	165.48(140.3-193.72)	467.65(415.3-523.42)	861.41(780.66-937.51)	68.93(57.08-81.44)	1.23(0.96-1.53)	2.48(1.83-3.25)	73.02(44.92-104.77)	30.05(23.84-36.33)	54.54(43.62-69.31)	75.59(52.31-103.42)
Bermuda	Male	7.71(6.7-8.82)	24.69(21.6-28.34)	176.34(148.59-207.61)	447.01(398.52-502)	876.73(796.03-955.55)	81.84(67.81-95.33)	1.53(1.18-1.89)	3.05(2.31-3.98)	66.97(41.99-95.78)	39.75(30.95-48.42)	73.92(58.66-91.95)	84.83(62.15-112.04)
Bhutan	Both	40.36(34.45-47.19)	143.79(125.39-164.54)	225.77(193.18-292.76)	4818.98(4150.47-5603.13)	9971.86(8830.62-11233.44)	71.01(60.69-81.85)	12.26(7.21-18.53)	41.45(27.83-57.15)	350.28(239.33-512.73)	350.55(213.86-515.2)	1013.34(700.06-1373.57)	467.49(329.06-669.46)
Bhutan	Female	19.01(16.15-22.11)	64.48(55.66-73.91)	218.44(184.19-283.54)	2159.27(1850.13-2535.26)	4346.7(3830.68-4897.94)	62.68(52.52-73.8)	6.28(3.25-9.74)	20.12(10.72-29.1)	365.97(240.45-570.59)	175.57(96.95-262.8)	484.15(282.06-702.87)	461.04(320.17-714.54)
Bhutan	Male	21.35(18.04-25.28)	79.31(69.31-92.85)	231.1(193.2-304.72)	2659.71(2263.33-3121.97)	5625.16(4949.68-6381.52)	78.42(66.25-90.77)	5.98(3.33-10.73)	21.34(13.48-31.34)	338.9(202.76-544.14)	174.98(102.15-303.47)	529.19(345.3-769.14)	474.29(305.21-726.24)

Bolivia (Plurinational State of)	Both	711.12(628.55-801.28)	3043.28(2696.28-3390.47)	127.42(112.39-146.61)	45248.37(40132.73-51314.71)	124054.15(112009.68-136952.92)	47.72(40.22-55.79)	301.27(225.24-394.58)	1258.66(904.93-1695.04)	237.33(184.2-297.12)	7291.14(5454.9-9565.61)	27113.05(19635.92-36235.58)	312.36(240.61-399.49)
Bolivia (Plurinational State of)	Female	305.02(268.04-342.73)	1402.52(1222.01-1595.81)	147.47(128.21-169.23)	21373.7(18664.43-24507.63)	57392.09(51724.01-63722.48)	46.18(36.23-56.44)	151.15(110.44-220.03)	654.12(464.83-908.18)	243.48(175.7-320.35)	3637.39(2679.26-5139.32)	13888.95(10028.3-19272.64)	313.9(228.05-409.81)
Bolivia (Plurinational State of)	Male	406.1(352.31-464.6)	1640.76(1446.38-1853.54)	111.87(93.69-132.29)	23874.67(21074.3-27129.32)	66662.06(59806.57-74193.48)	48.86(41.38-56.44)	150.12(105.69-199.01)	604.54(407.16-841.75)	230.42(169.28-308.8)	3653.75(2590.58-4810.99)	13224.1(9082.6-18282.93)	310.14(225.17-414.31)
Bosnia and Herzegovina	Both	702.84(619.15-800.72)	1807.03(1590.43-2043.49)	285.22(250.33-327.13)	54813.51(48820.37-61842.27)	72986.72(67364.74-79251.47)	81.16(69.32-94.6)	58.56(40.21-79.44)	143.17(94.61-204.13)	88.25(54.79-128.84)	1803.74(1305.52-2324.94)	3552.86(2558.99-4754.38)	116.83(84.62-155.28)
Bosnia and Herzegovina	Female	381.68(331.27-438.37)	895.65(777.65-1030.7)	241(205.32-279.33)	28717.47(25464.26-32621.28)	38179.25(34831.13-42005.29)	76.56(64.82-89.51)	36.37(25.05-49.07)	79.06(52.63-113.93)	56.14(25.3-94.99)	1059.05(770.39-1357.51)	1857.78(1367.47-2509.68)	82.62(52.07-120.07)
Bosnia and Herzegovina	Male	321.17(279.82-374.36)	911.38(791.74-1041.52)	339.38(282.4-401.03)	26096.04(23031.38-29747.19)	34807.47(31681.22-38236.8)	86.12(73.19-101.95)	22.19(14.92-30.59)	64.11(41.31-93.42)	136.97(87.97-198.92)	744.69(524-1009.33)	1695.08(1193.81-2299.47)	163.04(116.29-220.66)
Botswana	Both	115.69(99.94-131.64)	427.88(372.01-485.97)	109.64(97.81-122)	8700.67(7617.4-9964.47)	23525.33(20915.23-26452.29)	53.49(46.64-60.98)	28.66(18.23-42.07)	102.34(66.18-149.6)	74.91(37.17-117.34)	763.52(493.71-1108.34)	2763.67(1800.9-4011.32)	101.64(53.86-158.42)
Botswana	Female	47.18(40.26-54.44)	192.15(164.48-222.27)	135.36(119.32-152.42)	4486.47(3867.83-5235.46)	11874.16(10470.29-13396.93)	53.45(44.62-62.72)	10.2(5.88-18.64)	45.57(27.71-74.61)	112.11(46.91-197.67)	266.09(162.75-454.11)	1158.49(731.23-1852)	141.23(61.74-244.87)
Botswana	Male	68.52(58.85-78.42)	235.73(205.24-268.43)	91.07(77.31-107.46)	4214.2(3684.53-4817.96)	11651.17(10262.21-13083.85)	53.38(46.35-61.5)	18.46(11.39-26.73)	56.76(35.04-84.07)	54.78(11.9-106.59)	497.43(315.4-717.39)	1605.18(997.69-2381.14)	80.87(32.53-139.29)
Brazil	Both	22456.99(20170.71-24911.99)	76105.97(69139.55-84244.24)	148.31(135.93-162.66)	1254308.96(1146299.99-1369473.75)	2997735.08(2769956.34-3217604.15)	63.36(56.22-70.63)	3468.01(2768.51-4180.95)	10370.85(8237.11-12526.55)	108.55(91.21-126.45)	96296.93(77500.15-114873.29)	245480.05(199781.5-291253.94)	133.27(113.61-155.19)
Brazil	Female	11081.39(9927.57-12344.22)	37941.53(34415.06-42198.03)	146.26(133.6-162.19)	628576.27(571706.11-690865.71)	1514659.68(1397625.1-1626131)	62.68(53.7-71.64)	1594.91(1255.38-1945.98)	5089.13(4029.64-6202.99)	106.67(84.63-130)	44169.67(35397.95-53078.86)	117034.45(96162.31-139306.88)	132.21(109.2-158.81)
Brazil	Male	11375.59(10194.52-12627.32)	38164.43(34569.32-42272.46)	150.7(136.39-165.59)	625732.69(569528.19-683304.42)	1483075.4(1371655.63-1602734.85)	64.15(58.41-70.37)	1873.1(1508.02-2233.82)	5281.72(4153.97-6421.63)	107.79(89.95-126.69)	52127.26(41689.15-62814.29)	128445.6(103693.18-153756.32)	133.46(112.82-154.98)
Brunei Darussalam	Both	39.1(33.92-44.49)	132.17(113.15-153.9)	109.14(95.35-124.47)	2620.27(2279.05-3060.28)	6520.97(5814.6-7309.85)	46.18(37.25-56.52)	12.13(9.98-14.51)	33.82(27.71-40.07)	62.62(40.49-86.57)	312.68(254.69-376.15)	849.51(687.71-1009.44)	68.95(45.29-93.68)
Brunei Darussalam	Female	17.09(14.88-19.49)	59.71(51.05-69.79)	112.21(94.23-131.92)	1228.58(1056.97-1442.88)	3099.72(2754.63-3486.5)	45.78(35.07-57.56)	5.37(4.29-6.64)	16.88(13.73-20.37)	64.56(29.01-96.9)	139.13(110.15-173.92)	417.63(334.78-509.95)	70.02(34.29-103.66)
Brunei Darussalam	Male	22.01(18.78-25.39)	72.46(61.26-85.04)	107.23(91.48-124.78)	1391.69(1200.35-1633.22)	3421.25(3013.36-3894.35)	46.64(37.1-56.44)	6.75(5.31-8.34)	16.94(13.14-20.83)	58.33(29.92-98.06)	173.55(134.2-215.85)	431.88(336.59-530.72)	66.53(37.5-105.73)
Bulgaria	Both	2363.5(2043.1-2783.34)	4214.28(3709.69-4775.97)	136.29(109.19-165.64)	145399.85(131238.45-159767.02)	172038.93(157630.1-186866.79)	47.63(40.6-55.23)	165.12(113.05-219.35)	425.18(290.78-593.97)	134.46(103.05-172.19)	5184.93(3820.89-6602.11)	10709.13(7661.33-14496.83)	135.86(110.44-165.14)
Bulgaria	Female	1135.27(980.44-1331.63)	1972.09(1663.7-2295.77)	124.55(96.21-157.11)	73953.54(66074.09-81818.06)	90049.93(81562.56-98729.97)	49.46(40.54-58.77)	77.94(54.59-103.7)	200.62(135.63-280.75)	109.66(76.7-147.77)	2466.31(1825.86-3156.37)	5005.58(3622.21-6767.71)	117.38(89.67-147.98)
Bulgaria	Male	1228.23(1055.03-1448.82)	2242.2(1947.21-2547.69)	148.51(115.3-185.27)	71446.31(64358.39-78804.61)	81988.99(74759.45-89293.66)	45.71(37.77-54.55)	87.17(58.14-117.09)	224.56(149.19-320.98)	158.35(117.8-209.32)	2718.63(1974.45-3465.88)	5703.55(4011.9-7823.79)	152.22(121.06-190.18)
Burkina Faso	Both	671.61(590.74-753.37)	1865.57(1630.53-2098.5)	27.5(19.9-37.46)	53769.31(47355.48-60964.82)	130577.32(115628.24-147609.05)	3.55(0.11-7.5)	280.41(201.97-367.56)	607.71(451.52-810.93)	69.66(32.46-111.77)	6588.39(4747.28-8607.99)	14805.58(10958.86-19652.21)	80.96(38.68-130.69)
Burkina Faso	Female	356.48(308.49-406.61)	1047.87(909.25-1193.55)	36.97(26.2-49.11)	29012.35(25370.85-33461.33)	69260.3(61297.71-78633.88)	2.31(-1.99-7.1)	119.8(82.63-162.73)	304.78(218.77-406.28)	104.63(49.78-170.06)	2933.59(2071.43-3960.82)	7473.49(5369.68-9884.66)	108.44(52.56-174.64)
Burkina Faso	Male	315.13(273.33-358.5)	817.71(713.87-926.32)	17.15(7.07-29.49)	24756.96(21807.47-28138.5)	61317.01(53717.74-70187.7)	5.05(0.55-10.16)	160.6(114.59-211.6)	302.93(216.78-416.97)	44.17(5.65-94.21)	3654.81(2575.36-4853.35)	7332.09(5329.33-10207.88)	59.22(13.86-116.57)
Burundi	Both	272.59(242.64-304.9)	631.33(554.84-717.62)	16.11(8.07-23.94)	31616.66(27234.97-36971.85)	64486.41(55869.72-74669.77)	-3.74(-8.03-0.92)	171.85(121.25-234.26)	230.65(160.45-312.79)	54.08(23.98-93.78)	4122.05(2952.91-5606.27)	5893.13(4153.45-7918.18)	63.2(31.23-104.32)
Burundi	Female	122.88(106.78-139.11)	262.5(227.44-302.34)	10.79(2.28-19.82)	16336.9(13887.12-19202.35)	29835.99(25663.05-34842.54)	-12.27(-17.45--6.97)	71.68(47.39-110.54)	94.26(65.28-136.2)	66.1(25.08-123.97)	1779.67(1177.61-2682.37)	2417.26(1676.56-3437.63)	64.95(23.13-120.36)
Burundi	Male	149.71(131.34-168.86)	368.84(321.54-422.31)	19.06(9.36-29)	15279.76(13035.66-17859.35)	34650.42(30053.39-40258.11)	5.06(-0.17-11.06)	100.18(68.11-140.08)	136.38(90.75-194.28)	44.42(11.33-89.53)	2342.38(1616.48-3322.87)	3475.86(2356.88-4923.24)	60.44(24.34-111.1)
Cabo Verde	Both	32.66(28.61-36.62)	93.26(81.96-105.74)	82.59(69.49-97.67)	2510.59(2246.31-2803.29)	5885.59(5280.59-6542.01)	48.53(42.15-55.24)	9.1(6.89-12.12)	28.76(22.34-36.08)	126.84(68.27-180.42)	186.8(144.66-235.53)	573.85(446.27-711.6)	202.15(139-259.78)
Cabo Verde	Female	16.26(14.08-18.43)	49.73(43.51-56.56)	106.42(86.86-125)	1394.18(1240.1-1568.11)	2993.85(2697.27-3315.01)	43.3(36.17-50.89)	4.04(2.94-5.7)	12.39(9.23-16.13)	143.16(57.54-221.23)	83.12(63.36-108.37)	225.63(173.17-280.22)	197.14(118.16-272.24)
Cabo Verde	Male	16.4(14.21-18.58)	43.53(37.75-49.45)	60.06(44.34-78.57)	1116.4(991.79-1259.54)	2891.73(2550.63-3281.63)	55.22(46.78-64.19)	5.06(3.73-6.79)	16.37(12.53-20.79)	113.46(55.96-172.96)	103.68(77.94-133.56)	348.22(262.63-436.39)	201.89(130.54-275.86)
Cambodia	Both	647.29(557.7-750.11)	2694.36(2354.27-3071.14)	188.79(168.87-209.88)	105723.96(90307.39-122285.04)	256586.83(227078.38-289408.67)	53.47(45.59-61.77)	347.4(260.8-447.64)	780.84(600.25-992.91)	132.4(81.06-189.31)	10974.44(8129.29-14210.2)	22791.91(17323.7-29341.01)	205.56(141.25-287.7)
Cambodia	Female	380.64(326.03-442.92)	1609.33(1392.54-1851.33)	201.07(174.45-228.46)	58105.85(49429.67-67414.87)	134851.73(119298.03-151419.12)	49.77(39.84-60.29)	197.03(143.46-255.5)	436.07(327.93-558.35)	125.3(75.57-192.81)	6345.29(4691.84-8347.02)	12572.61(9598.07-16100.93)	188.97(126.34-275.75)
Cambodia	Male	266.65(227.97-313.03)	1085.03(930.02-1250.33)	174.74(151.76-199.2)	47618.11(40422.44-55437.45)	121735.1(105554.75-139954.69)	58.14(50.13-66.68)	150.37(109.38-201.97)	344.78(248.18-460.98)	140.38(71.18-226.4)	4629.15(3341.06-6187.09)	10219.3(7340.98-13715.99)	227.34(141.29-348.15)
Cameroon	Both	981.44(864.98-1116.02)	3710.36(3264.14-4226.1)	41.34(32.65-50.5)	61709.61(54576-69463.62)	199870.16(177867.76-224069.87)	18.51(14.71-22.69)	483.1(343.91-643.94)	1222.38(878.21-1668.41)	42.44(15.39-81.77)	11802.45(8308.89-15797.68)	31105.43(21793.88-43107.7)	63.3(29.69-109.94)
Cameroon	Female	525.03(455.77-605.57)	1870.24(1633.01-2155.94)	35.24(24.71-47)	31937.98(27963.24-36271.03)	99438.44(87493.52-111171.45)	14.34(9.57-19.63)	259.33(159.82-357.3)	597.41(408.7-824.26)	31.03(0.15-96.91)	6482.89(4121.05-8993.15)	15087.54(10100.78-21169.45)	43.15(7.45-113.85)
Cameroon	Male	456.41(399.53-521.29)	1840.12(1592.92-2128.81)	48.33(36.53-61.78)	29771.62(26156.26-33870.87)	100431.72(88846.46-114238.84)	22.96(18.05-28.48)	223.77(152.82-320.23)	624.97(432.8-893.42)	55.89(20.7-101.19)	5319.55(3589-7546.96)	16017.89(10895.62-23188.7)	87.83(44.72-142.86)
Canada	Both	9865.95(8486.7-11199.27)	21449.95(18872.3-24127.37)	62.53(42.56-82.1)	249154.56(225605.58-275629.33)	577060.11(527270.75-628340.94)	70.3(57.55-83)	598.54(440.42-783.34)	1433.78(1018.32-1951.35)	58.24(34.23-84.42)	13282.4(10181.15-16563.52)	28433.05(21818.34-36011.78)	55.77(40.43-74.63)

Canada	Female	4990.55(4267.05-5777.88)	10392.99(9167.41-11696.86)	56.58(36.12-80.48)	129169.17(115211.28-144380.53)	294944.87(265866.1-322619.19)	68.59(54.16-83.34)	349.32(260.03-459.33)	857.12(602.52-1184.61)	52.69(27.01-84.2)	7182.88(5597.34-8947.35)	15273.97(11776.58-19538.07)	47.15(28.25-67.78)
Canada	Male	4875.4(4187.91-5543.47)	11056.95(9483.17-12813.33)	68.47(45.35-92.28)	119985.39(107722.4-133020.56)	282115.24(254350.67-311954.98)	72.11(58.37-86.87)	249.22(178.23-332.17)	576.66(411-797.9)	61.26(33.49-91.77)	6099.52(4488.36-7732.81)	13159.08(9785.32-16927.42)	64.5(43.49-87.56)
Central African Republic	Both	128.65(111.22-148.03)	293.59(254.82-337.9)	20.31(13.38-27.06)	16868.14(14327.16-19601.03)	32153.45(27568.97-37012.16)	-0.53(-4.82-4.19)	74.68(50.72-101.09)	135.58(89.17-193.25)	26.67(3.83-55.98)	2047.12(1393.56-2789.63)	3834.76(2520.34-5480.99)	38.6(13.18-70.83)
Central African Republic	Female	58.45(49.42-68.68)	140.03(119.64-163.95)	27.48(18.68-37.11)	8662.97(7260.62-10120.12)	16185.26(13735.5-18640.02)	-3.15(-8.53-2.2)	28.61(14.47-42.65)	53.08(23.39-84.5)	30.72(2.11-63.13)	793.61(421.94-1181.55)	1474.27(712.72-2316.68)	40.31(10.51-77.61)
Central African Republic	Male	70.21(61.07-80.71)	153.56(132.94-177.33)	14.16(5.69-23.41)	8205.18(6964.62-9614.93)	15968.19(13568.34-18643.52)	2.26(-2.88-7.72)	46.07(30.66-63.39)	82.5(55.39-113.69)	23.94(-6.46-68.16)	1253.51(842.88-1738.93)	2360.49(1606.55-3291.47)	36.9(4.23-84.12)
Chad	Both	454.08(401.75-510.81)	1115.89(985.48-1274.91)	-10.28(-15.6--5.2)	34444.43(30461.83-39066)	80161.22(70781.91-91175.13)	-13.37(-16.38--10.01)	201.52(136.58-299.18)	390.81(269.19-555.88)	22.33(-4.78-55.28)	4591.08(3147.4-6700.72)	9655.22(6584.59-13637.65)	30.95(-0.33-69.56)
Chad	Female	223.05(192.11-255.13)	492.92(424.79-571)	-17.37(-23.34--11.41)	17914.59(15752.46-20481.69)	37911.85(33080.39-43770.97)	-19.73(-23.37--15.9)	90.15(56.57-154.09)	141.28(92.7-230.17)	4.39(-21.52-37.92)	2133.25(1362.85-3713.95)	3690.03(2392.96-5965.73)	10.45(-17.87-46.06)
Chad	Male	231.03(202.51-262.22)	622.97(548.87-714.78)	-4.08(-11.96-4.16)	16529.85(14601.3-18800.45)	42249.37(37138.4-48278.67)	-6.71(-10.55--2.71)	111.38(73.65-169.27)	249.53(169.7-357.66)	34.88(-0.12-81.09)	2457.83(1623.6-3642.92)	5965.19(4031.93-8559.2)	47.74(5.99-103)
Chile	Both	2858.94(2532.86-3239.05)	10633.09(9524.01-11898.32)	183.09(159.01-209.55)	120648.47(108573.22-133085.1)	301747.06(277220.04-328115.67)	79.93(70.92-89.94)	356.34(267.79-447.81)	1402.05(1072.34-1791.79)	163.93(128.37-205.82)	8457(6460.12-10279.49)	27007.06(21044.67-33286.12)	155.39(129.65-186.79)
Chile	Female	1562.94(1368.63-1800.93)	5570.64(4918.16-6263.73)	168.51(142.6-197.65)	65722.67(58926.99-73188.51)	160606.8(146342.76-175738.26)	75.95(65.27-87.68)	180.91(134.47-229.28)	748.1(562.8-974.63)	156.96(118.33-204.88)	4176.7(3162.41-5123.06)	13565.68(10604.01-16796.52)	144.27(116.34-177.55)
Chile	Male	1296(1122.79-1488.81)	5062.45(4451.19-5790.09)	200.69(165.82-236.82)	54925.8(49047.29-61138.28)	141140.26(128987.25-154723.35)	84.69(73.94-97.68)	175.44(131.15-220.1)	653.95(501.35-835.79)	166.62(131.13-208.04)	4280.3(3258.96-5205.68)	13441.38(10375.8-16691.62)	165.52(133.55-199.78)
China	Both	163054.32(144122.8-183482.05)	433951.66(390221.94-481040.83)	129.36(118.4-140.38)	16972362.27(15231399.25-18994828.03)	31075697.34(28151769.76-33908523.69)	52.47(44.64-61.11)	27412.94(21888.6-33263.31)	63354.4(49787.07-77280.21)	81.19(59.28-103.41)	849044.04(663737.18-1032537.09)	1640410.22(1312349.07-1979867.96)	108.68(82.51-138.75)
China	Female	78131.98(68873.05-88169.65)	209643.41(189326.72-232608.89)	129.3(117.18-142.57)	9207351.6(8254838.74-10371531.82)	16946030.66(15319817.39-18523044.9)	50.97(41.95-60.58)	14143.97(10908.72-17470.06)	31058.39(23720.36-38907.3)	87.13(60.73-112.32)	428996.76(335326.82-530607.92)	792926.87(617200.87-972754.94)	107.46(78.11-139.9)
China	Male	84922.34(75003.06-95376.38)	224308.25(199329.31-250281.35)	129.48(117.25-141.54)	7765010.67(6976423.9-8627363.39)	14129666.68(12864035.88-15411707.95)	53.77(46.69-61.43)	13268.97(9997.86-16929.57)	32296.01(24274.92-41392.71)	79.08(50.86-108.04)	420047.28(317211.61-528129.91)	847483.34(655066.52-1064265.62)	111.17(77.32-149.08)
Colombia	Both	5340.19(4673.35-6129.4)	21753.1(19386.12-24599.38)	199.88(170.98-230.09)	381167.09(333243.24-431844.1)	958366.05(871067.82-1049015.81)	73.19(61.94-86.15)	866.31(677.35-1055.28)	2634.27(1858.3-3630.09)	109.14(83.65-136.24)	22995.04(18306.68-27517.21)	61776.45(47133.59-80004.79)	141.82(112.52-174.58)
Colombia	Female	2543.71(2209.25-2931.24)	10340.05(8999.11-11833.36)	194.83(157.23-238.99)	187737.36(163414.78-216888.34)	467831.66(419175.01-517968.23)	68.88(55.28-84.54)	431.23(336.18-529.25)	1255.27(894.48-1724.9)	75.94(51.29-101.33)	11118.36(8895.52-13321.97)	29118.65(22036.65-37835.18)	111.54(82.67-142.61)
Colombia	Male	2796.48(2410.21-3257.54)	11413.05(9875.93-13154.82)	205.04(172.94-246.69)	193429.73(169212.23-219300.23)	490534.39(442300.14-541623.91)	77.75(66.03-90.45)	435.08(337.08-533.24)	1379(968.81-1923.53)	140.13(106.77-176.96)	11876.68(9338.25-14356.09)	32657.8(24619.8-43013.84)	169.52(135.41-209.42)
Comoros	Both	27.42(24.09-30.94)	76.26(67.23-85.63)	92.34(75.25-129.93)	2781.41(2457.26-3174.94)	5953.4(5304.67-6714.95)	40.78(34.08-47.77)	15.4(9.74-21.46)	31.02(23.08-41.06)	116.57(71.29-214.32)	345.64(204.19-474.42)	681.17(502.66-897.99)	172.9(109.27-326.67)
Comoros	Female	12.62(11.03-14.36)	40.08(34.87-45.8)	124.41(101.74-165.52)	1393.55(1212.43-1618.67)	3001.17(2662.55-3388.09)	42.27(32.78-51.37)	6.69(4.22-9.51)	16.73(12.21-22.26)	151.83(85.33-259.74)	155.09(92.69-218.47)	366.92(272.86-484.78)	215.35(132.37-400.63)
Comoros	Male	14.8(12.88-16.93)	36.18(31.55-41.13)	65.3(47.14-104.85)	1387.86(1218.24-1584.46)	2952.23(2592.49-3349.83)	39.29(32.6-46.88)	8.71(5.27-12.62)	14.29(9.87-19.8)	87.41(40.49-184.33)	190.54(108.54-274.67)	314.25(219.07-432.8)	136.54(72.82-280.9)
Congo	Both	135.73(117.63-156.57)	456.78(396.76-519.51)	60.39(50.54-70.18)	15648.61(13546.53-18176.88)	40264.06(35172.35-45647.1)	20.52(14.79-26.82)	84.45(54.91-117.94)	170.11(108.71-241.04)	71.59(42.33-109.72)	2174.12(1412.8-3093.6)	4313.57(2825.71-6098)	83.62(48.61-129.24)
Congo	Female	66.46(56.68-77.95)	223.26(191.41-259.04)	64.78(52.86-77.21)	8188.81(7029.29-9567.83)	19796.97(17241.2-22534.46)	14.44(8.14-21.25)	38.43(16.47-58.07)	83.84(36.8-130.32)	79.95(42.25-127.9)	1000.98(460.49-1519.39)	2135.48(1016.44-3314.7)	92.21(47.84-148.22)
Congo	Male	69.26(59.85-79.51)	233.52(200.76-268.55)	55.9(44.02-68.43)	7459.79(6422.16-8723.47)	20467.09(17752.16-23514.7)	27.1(20.17-34.68)	46.01(32.48-63.87)	86.27(59.12-120.62)	64.49(23.81-131)	1173.14(814.2-1616.13)	2178.1(1493.7-3042.6)	76.24(32.39-154.18)
Cook Islands	Both	3.13(2.72-3.56)	9.11(7.95-10.26)	177.18(157.73-198.62)	294.65(259.5-334.49)	499.5(453.84-544.83)	76.28(65.85-88.72)	0.96(0.76-1.18)	2.55(2.06-3.18)	114.51(79.86-158.01)	26.52(20.85-32.56)	61.91(50.45-76.7)	136.86(100.87-180.61)
Cook Islands	Female	1.25(1.08-1.46)	3.78(3.27-4.36)	175.64(149.63-202.56)	143.54(125.37-165.41)	251.37(225.67-278.9)	71.23(59.02-84.58)	0.41(0.3-0.53)	1.05(0.8-1.35)	98.23(56.98-150.12)	11.64(8.87-14.7)	25.87(20.45-32.78)	115.02(74.43-166.39)
Cook Islands	Male	1.88(1.61-2.16)	5.34(4.62-6.1)	181.38(157.02-208.34)	151.11(132.73-171.48)	248.13(225.31-271.54)	81.77(70.32-95.05)	0.55(0.41-0.69)	1.51(1.21-1.86)	126.53(81.58-188.43)	14.87(11.2-18.89)	36.04(28.51-44.54)	154.63(105.27-218.22)
Costa Rica	Both	1066.35(986.03-1156.06)	3200.86(2975.82-3458.82)	106.35(98.33-114.83)	46129.08(41429.71-51321.78)	117362.77(108819.5-127247.5)	64.4(56.56-73.29)	68.46(53.27-84.57)	383.37(275.25-529.68)	161.3(135.35-190.82)	2137.5(1720.9-2581.22)	9598.5(7240.63-12403.74)	164.86(137.68-199.73)
Costa Rica	Female	538.76(496.51-584.22)	1709.26(1577.08-1845.24)	111.72(102.58-122.46)	22966.04(20368.33-25797.99)	59970.03(55127.3-65382.09)	62.57(53.54-72.83)	32.45(25.09-40.58)	178.22(126.68-243.68)	151.29(119.2-187.79)	1022.09(824.56-1235.19)	4410.06(3374.21-5674.29)	153.2(121.3-189.62)
Costa Rica	Male	527.58(480.23-579.77)	1491.6(1371.98-1639.96)	100.43(90.35-110.57)	23163.04(20804.81-25620.52)	57392.74(52685.79-62563.35)	66.57(58.62-75.09)	36.01(27.97-44.4)	205.16(145.99-285.39)	169.83(139.71-205.13)	1115.41(894.9-1352.83)	5188.44(3838.93-6833.57)	175.65(144.79-213.45)
Croatia	Both	1451.39(1261.56-1675.07)	2980.81(2649.13-3354.25)	148.68(121.52-182.33)	79695.03(71759.38-88411.44)	104209.5(95881.64-112978.63)	49.86(41.66-59.64)	107.5(77.63-141.86)	218.83(147.77-310.1)	103.61(69.97-147.03)	2980.42(2234.73-3760.11)	4715.62(3498.66-6166.54)	90.56(67.94-119.41)
Croatia	Female	790.02(680.64-923.18)	1498.55(1295.27-1723.37)	125.31(96.65-155.15)	43011.21(38393.41-48375)	55471.31(50587.37-60732.78)	47.52(37.73-57.91)	62.3(45.09-82.7)	130.45(87.27-186.58)	99.94(60.63-145.97)	1633.34(1212.61-2063.9)	2563.08(1887.66-3405.26)	80.04(54.61-112.52)
Croatia	Male	661.37(570-769.65)	1482.26(1303.85-1713.29)	177.14(140.72-233.19)	36683.82(32856.65-40774.64)	48738.19(44420.56-53031.34)	52.6(43.04-64.61)	45.21(32.16-59.78)	88.38(59.33-126.25)	104.68(70.14-148.43)	1347.08(996.37-1722.63)	2152.54(1560.54-2815.56)	100.91(73.55-137.48)
Cuba	Both	1859.25(1635.58-2114.96)	5913.84(5205.65-6693.93)	185.76(161.55-209.81)	142284.38(126774.46-160114.11)	251692.96(230214.4-272897.7)	67.23(55.87-79.07)	201.31(160.34-243.21)	726.18(529.63-983.62)	140.13(114.44-167.95)	6119.69(4931.3-7335.19)	18735.29(14239.36-24126.31)	161.71(134.22-191.2)
Cuba	Female	895.54(769.84-1039.93)	2965.19(2576.83-3386.13)	195.24(160.78-231.12)	68766.91(60752.37-78177.95)	124215.36(112388.08-135852.99)	69.01(54.87-83.07)	86.36(68.03-104.33)	372.41(271.44-512.33)	177.15(141.31-218.57)	2772.26(2224.38-3321.7)	9492.72(7163.22-12439.23)	194.02(154.58-235.72)

Cuba	Male	963.72(836.49-1116.06)	2948.65(2577.65-3410.14)	177.01(150.94-206.74)	73517.46(65317.46-83655.25)	127477.6(115361.25-139853.24)	65.67(55.25-77.63)	114.95(90.55-139.11)	353.77(256.74-477.42)	110.82(85.71-139.32)	3347.43(2661.38-4007.19)	9242.57(6945.92-11983.22)	135.12(110.66-164.1)
Cyprus	Both	339.74(291.32-388.22)	823.72(717.53-936.81)	47.93(37.91-58.27)	8808.61(7984.6-9734.29)	19622.46(17938.49-21361.64)	29.86(24.39-35.89)	48.51(33.45-68.54)	79.26(55.9-110.73)	7.29(-14.14-31.4)	876.25(635.39-1177.68)	1331.19(1006.87-1777.98)	0.31(-17.08-18.24)
Cyprus	Female	176.82(150.76-203.48)	395.84(343.01-454.92)	33.77(23.15-45.25)	4337.35(3926.38-4815.3)	9492.58(8571.36-10541.14)	25.83(19.03-32.52)	25.61(17.25-36.98)	40.85(28.1-59.28)	9.59(-19.94-48.21)	434.9(312.36-590.69)	621.51(465.36-827.19)	-5.8(-27.39-18.35)
Cyprus	Male	162.93(138.36-188.41)	427.87(369.64-490.78)	63.82(47.5-81.04)	4471.26(4025.98-4961.27)	10129.88(9175.29-11087.85)	34.02(26.52-41.94)	22.89(15.27-32.71)	38.42(26.49-54.94)	5.54(-15.65-35.71)	441.35(310.72-596.88)	709.68(519.73-951.09)	6.37(-12.78-32.49)
Czechia	Both	2616.78(2274.73-2967.11)	6072.28(5278.81-6875.22)	130.41(112.48-150.48)	164872.27(149981-180683.7)	242755.02(222689.21-262804.39)	41.8(35.56-48.06)	259.74(190.02-334.82)	308.76(212.64-424.67)	34.51(13.28-59.21)	6887.23(5169.66-8683.78)	7794(5813.45-10010.43)	31.02(13.66-51.77)
Czechia	Female	1437.96(1240.51-1634.65)	3005.24(2577.98-3445.51)	108.29(88.04-133.65)	89237.48(80646.5-98555.24)	125899.88(114249.34-137390.48)	37.96(30.86-45.02)	141.34(105.3-180.49)	174.66(119.17-241.38)	37.27(11.81-67.48)	3527.24(2682.18-4407.58)	3995.05(3009.91-5097.6)	26.45(8.69-47.24)
Czechia	Male	1178.82(1007.23-1353.97)	3067.04(2615.48-3583.19)	157.27(129.85-190.43)	75634.79(67595.6-83548.66)	116855.15(106352.93-127548.38)	46.38(38.23-54.45)	118.4(83.51-156.57)	134.11(91.15-187.03)	30.45(8.51-56.42)	3359.99(2469.04-4295.02)	3798.95(2792.84-4941.62)	35.09(15.28-60.2)
Ivory Coast	Both	767.98(679.6-869.68)	2557.6(2260.47-2904.92)	63.34(53.04-73.19)	66940.47(57645.84-77110.32)	174463.35(153584.06-199709.1)	23.59(17.92-29.87)	295.29(207.47-394.64)	732.81(514.1-985.34)	94.56(55.67-149.39)	7972.54(5474.34-10737.85)	19254.9(13428.59-26056.74)	112(65.7-173.79)
Ivory Coast	Female	358.13(309.84-411.57)	1215.44(1046.71-1390.54)	68.81(55.51-83.5)	31065.1(26632.17-36159.18)	81044.5(71372.94-92241.96)	24.43(17-32.21)	109.66(76.43-152.25)	290.57(199.27-405.38)	113.22(53.25-187.09)	3060.15(2120.6-4313.24)	7719.34(5310.61-10902.92)	125.24(63.14-198.52)
Ivory Coast	Male	409.85(357.25-465.79)	1342.16(1168.12-1530.02)	58.38(46.29-72.52)	35875.37(30814.64-41584.81)	93418.86(80998.26-107808.46)	22.8(16.89-29.6)	185.63(129.71-253.15)	442.24(297.95-611.95)	83.17(40.81-144.01)	4912.39(3332.02-6841.01)	11535.56(7731.8-16045.49)	103.27(52.19-173.61)
Democratic People's Republic of Korea	Both	3026.9(2642.61-3457.77)	7543.47(6633.96-8521.01)	100.69(86.87-117.71)	307890.61(269983.26-351452.52)	525086.35(471077.1-584007.01)	36.73(29.58-44.23)	731.48(535.88-942.09)	1466.4(1132.69-1882.32)	42.83(20.36-72.95)	23972.52(17697.47-30921.93)	43649.12(33945.55-55201.14)	95.06(63.18-137.63)
Democratic People's Republic of Korea	Female	1707.22(1482.43-1968.22)	4266.86(3709.72-4853.6)	109.66(90.87-132.01)	186691.97(162020.32-213713.27)	307091.48(272395.89-342642.35)	38.23(29.64-47.48)	464.84(334.58-610.51)	862.91(640.62-1165.66)	32.01(7.33-69.2)	14513.33(10681.98-18860.27)	24468(18526.27-32106.77)	83.57(49.49-129.79)
Democratic People's Republic of Korea	Male	1319.68(1122.51-1529.5)	3276.61(2855.22-3796.56)	91.41(73.63-110)	121198.64(105666.96-139050.7)	217994.87(194177.51-245223.81)	37(29.8-44.4)	266.64(185.46-371.99)	603.49(444.63-798.88)	61.65(27.29-109.6)	9459.19(6647.71-13030.39)	19181.12(14396.03-24870.9)	113.96(69.2-170.79)
Democratic Republic of the Congo	Both	1853.15(1617.78-2112.3)	5321.5(4684.4-6010.13)	29.63(21.55-37.64)	228662.22(197917.08-265131.64)	526631.76(459732.96-602693.88)	2.06(-1.96-6.3)	931.37(676.12-1212.67)	1803.13(1255.42-2444.8)	81.11(40.96-124.83)	24105.67(17410.02-31742.44)	45926.04(32635.66-61997.8)	99.27(52.53-152.23)
Democratic Republic of the Congo	Female	901.28(769.67-1044.94)	2803.27(2428.54-3202.92)	43.88(31.69-55.71)	117832.13(101098.4-136806.39)	262001.22(229663.84-298388.71)	-0.22(-5.48-5.61)	389.27(272.17-522.68)	906.36(600.01-1267.87)	116.42(56.3-184.35)	10714.22(7471.5-14439.95)	22374.65(15341.2-31142.59)	122.52(61.63-199.13)
Democratic Republic of the Congo	Male	951.87(834.88-1087)	2518.23(2185.99-2884.08)	16.48(6.8-25.94)	110830.09(95502.3-129360.2)	264630.55(226692.2-309659.4)	4.46(-0.57-9.87)	542.1(384.39-729.47)	896.77(616.21-1231.35)	55.59(17.21-102.74)	13391.45(9481.54-18224.9)	23551.39(16177.41-32069.71)	80.91(35.32-136.6)
Denmark	Both	2167.16(1872.4-2466.05)	3824.25(3361.19-4296.07)	55.93(40.87-70.89)	67040.21(61077.98-73381.42)	96236.26(88552.18-104098.89)	27.22(21.44-33.35)	77.26(55.65-105.23)	240.66(171.73-334.89)	233.32(179.63-291.24)	2072.35(1574.5-2645.51)	4460.14(3406.14-5754.01)	131.56(103.56-163.77)
Denmark	Female	1204.19(1041.23-1378.62)	1924.42(1668.82-2202.19)	42.69(27.97-58.32)	35529.35(32210.67-39427.08)	47903.89(43674.84-52269.03)	20.92(14.62-27.42)	40.76(29.31-55.11)	119.85(84.22-168.21)	213.28(155.53-280.92)	1102.07(843.81-1392.53)	2112.56(1642.63-2693.44)	102.59(74.61-136)
Denmark	Male	962.98(825.31-1108.69)	1899.83(1632.5-2191.43)	72.34(48.93-96.35)	31510.86(28337.15-34813.26)	48332.36(44079.48-52949.56)	34.21(26.21-43.38)	36.5(25.48-50.52)	120.81(85.69-171.12)	255.66(198.67-319.74)	970.29(714.45-1254.01)	2347.58(1766.92-3121.5)	164.91(129.13-207.33)
Djibouti	Both	16.83(14.68-19.28)	99.64(87.12-113.4)	147.28(131.35-163.07)	2416.08(2044.89-2853.07)	9174.15(8041.91-10457.37)	55.34(46.2-66.26)	6.13(4.04-8.83)	33.6(23.51-46.26)	199.75(128.12-282.5)	168.81(111.78-242.01)	872.41(601.19-1197.18)	254.44(169.3-350.27)
Djibouti	Female	7.36(6.34-8.52)	41.71(36.33-48.05)	137.05(118.95-155.75)	1088.45(918.75-1295.52)	4115.18(3581.16-4770.03)	51.38(41.35-62.84)	2.54(1.67-3.97)	13.88(9.29-20.7)	205.35(125.39-301.4)	69.81(46.33-108.11)	357.31(238.42-525.26)	245.69(158.71-351.36)
Djibouti	Male	9.47(8.22-10.96)	57.92(50.05-67.18)	155.18(133.91-178.68)	1327.63(1119.2-1575.37)	5058.97(4428.45-5799.39)	58.88(48.51-70.92)	3.6(2.24-5.37)	19.72(13.28-27.49)	195.71(110.01-304.88)	99(63.1-148.56)	515.1(352.56-720.72)	261.3(163.06-377.9)
Dominica	Both	20.07(17.61-22.5)	35.04(31.01-39.3)	83.69(70.48-97.08)	1038.98(943.26-1151.03)	1424.32(1306.64-1547.76)	46.27(39.66-52.79)	7.42(5.95-9.06)	12.38(9.41-15.72)	56.8(36.15-81.78)	160.54(130.99-193.21)	270.14(210.45-341.62)	65.1(45.89-87.59)
Dominica	Female	10.57(9.17-12.06)	15.79(13.8-17.68)	61.25(46.29-76.97)	551.16(497.32-613.7)	662.95(601.73-726.58)	31.76(25.03-38.98)	3.86(3.02-4.78)	6(4.55-7.83)	64.47(36.87-99.09)	81.19(64.7-99.55)	122.88(95.26-155.74)	62.59(40.05-91.66)
Dominica	Male	9.5(8.21-10.84)	19.25(16.8-21.94)	107.68(87.02-130.35)	487.82(439.57-543.19)	761.37(690.95-834.12)	62.21(53.84-72.1)	3.56(2.79-4.43)	6.38(4.64-8.31)	50.58(25.5-84.01)	79.35(62.66-98.53)	147.27(110.33-191.49)	68.13(42.72-102.47)
Dominican Republic	Both	614.19(534.54-709.26)	2701.01(2386.45-3063.05)	181.49(160.82-203.12)	57867.8(50757.31-66495.78)	140725.09(127622.44-154999.25)	61.72(52-72.77)	119.72(91.62-149.2)	552.53(383.67-771.66)	154.21(105.15-206.03)	3393.29(2635.22-4163.44)	14476.72(10302.03-20145.19)	252.84(188.26-322.94)
Dominican Republic	Female	284.61(247.06-327.61)	1275.03(1114.62-1450.42)	193.34(167.41-220.21)	28449.88(24692.18-32783.6)	67004.68(60294.14-74135.16)	60.17(47.94-73.22)	53.21(39.94-67.21)	262.75(176.16-364.59)	186.88(121.71-247.22)	1526.26(1191.13-1900.61)	6608.23(4763.66-8987.65)	281.98(202.08-360.62)
Dominican Republic	Male	329.58(284.9-381.89)	1425.98(1236.52-1636.54)	170.47(143.87-196.49)	29417.92(25701.29-33614.31)	73720.41(66280.02-81529.39)	62.85(52.92-73.78)	66.5(49.09-84.02)	289.78(188.92-443.15)	130.65(75.99-211.93)	1867.03(1412.34-2330.13)	7868.49(5309.96-11963.96)	230.88(154.74-335.8)
Ecuador	Both	1181.19(1027.2-1346.01)	6924.77(6169.02-7738.62)	234.59(206.59-264.81)	76416.22(67406.51-86651.46)	237474.14(217386.44-259095.82)	79.33(68.05-91.28)	254.06(199.65-308.25)	1919.22(1407.41-2551.61)	320.51(275.95-365.53)	6197.42(4914.4-7433.07)	40338.59(29770.1-52921.08)	382.25(321.8-454.78)
Ecuador	Female	579.4(501.17-666.39)	3472.26(3071.32-3901.57)	238.12(206.17-271.92)	36144.46(31620.34-41422.36)	114827.63(104109.91-126009.23)	82.7(69.36-97.31)	130.01(101.48-157.24)	985.25(720.6-1291)	316.63(267.28-377.46)	3143.19(2483.21-3779.5)	20420.77(15160.76-26650.77)	378.05(311.16-467.97)
Ecuador	Male	601.79(520.61-690.01)	3452.51(3018.22-3919.23)	231.45(197.52-270.19)	40271.76(35288.35-45877.68)	122646.51(111267.07-135083.78)	76.36(65.81-87.58)	124.05(96.39-154.72)	933.97(675.78-1269.38)	323.18(267.75-385.68)	3054.23(2395.52-3727.3)	19917.81(14258.01-26546.4)	386.05(313.19-467.75)
Egypt	Both	10648.05(9371.5-12229.31)	43073.19(37762.33-49122.38)	142.15(128.82-154.97)	458669.34(409490.79-511113.83)	1233329(1120022.1-1354223.24)	53.62(46.61-60.34)	2831.03(1833.68-3780.22)	7306.04(4099.56-11030.29)	113.85(53.22-163.23)	68875.37(45839.04-90852.11)	184235.33(109808.7-273243.11)	190.61(118.56-256.46)
Egypt	Female	6153.24(5320.48-7161.67)	20013.82(17410.04-23009.23)	96.18(83.23-109.54)	250338.55(222819.02-281553.01)	592391.5(531258.17-655661.32)	36.09(29.72-42.9)	1608.44(842.68-2321.79)	3880.17(1694.1-5839.9)	125.84(46.23-187.41)	39085.8(21921.35-54968.81)	97255.08(47368.56-143899.79)	190.46(101.96-272.69)

Egypt	Male	4494.81(3922.66-5203.31)	23059.37(19926.78-26625.42)	204.83(181.03-228.28)	208330.79(185831-234743.41)	640937.51(583162.31-707068.14)	74.64(64.47-84.81)	1222.59(860.13-1721.95)	3425.87(2042.82-5788.07)	110.38(42.33-178.77)	29789.57(21358.69-41195.37)	86980.25(54842.72-142753.34)	197.76(108.23-285.25)
El Salvador	Both	894.75(771.27-1045.08)	2822.97(2510.41-3179.09)	189.75(166.81-214.88)	60041.16(53114.29-68059.38)	124069.81(113432.03-136023.53)	77.17(66.37-87.85)	155.51(122.15-190.13)	1172.46(831.76-1580.15)	514.37(441.05-591.64)	4508.9(3557.1-5460.62)	27509.57(19978.99-36916.52)	638.25(528.8-753.82)
El Salvador	Female	411.64(355.77-483.21)	1327.42(1160.56-1489.98)	183.37(153.49-219.29)	29906.95(26056.15-34159.29)	61651.71(56066.46-68666.19)	68.66(56.36-81.57)	68.05(52.37-83.1)	462.63(329.17-620.1)	410.77(341.67-505.32)	1954.22(1529.67-2382.33)	10310.93(7600.53-13823.9)	509.34(411.03-620.37)
El Salvador	Male	483.11(410.53-568.69)	1495.56(1289.03-1716.84)	198.17(172.88-224.4)	30134.22(26582.69-34254.79)	62418.09(56221.04-68976.1)	87.2(75.29-100.07)	87.46(68.32-109.73)	709.83(499.88-965.74)	602.56(495.79-711.1)	2554.67(1998.35-3169.96)	17198.64(12147.11-23332.35)	744.79(609.12-893.2)
Equatorial Guinea	Both	21.29(18.71-24.07)	93.37(81.83-105.74)	50.77(40.11-62.5)	2621.9(2274.9-3020.12)	8482.8(7288-9820.7)	-0.01(-4.13-4.46)	12.36(8.25-17.04)	33.92(22.83-49.18)	161.18(85.56-256.83)	319.55(210.51-440.44)	788.36(523.93-1166.43)	146.49(77.59-238.81)
Equatorial Guinea	Female	10.07(8.72-11.52)	51.4(44.73-59.11)	97.22(80.48-114.18)	1425.51(1227.9-1663.03)	4245.7(3676.1-4853.9)	3.24(-2.1-8.6)	4.88(2.42-7.59)	17.61(9.62-27.02)	214.14(94.91-399.48)	128.24(65.35-198.24)	401.85(230.84-634.16)	199.34(95.1-354.78)
Equatorial Guinea	Male	11.23(9.85-12.78)	41.97(36.49-48.15)	14.66(5.1-25.92)	1196.39(1036.64-1391.18)	4237.1(3574.08-5033.31)	-2.13(-8.09-4.9)	7.47(4.71-10.77)	16.31(10.67-22.96)	125.1(45.56-260.11)	191.31(120.71-271.47)	386.52(254.34-562.95)	110.42(37.86-222.14)
Eritrea	Both	101.46(88.06-117.6)	383.78(334.74-441.27)	89.06(77.45-102.53)	15058.44(12816.51-17681.04)	39664.55(34410.48-45660.82)	19.58(14.21-25.69)	50.29(30.71-74.51)	150.03(98.39-215.28)	254.24(158.14-373.65)	1462.54(940.61-2132.94)	4089.56(2710.09-5830.75)	283.84(194.86-399.93)
Eritrea	Female	50.41(43.54-58.49)	193.89(168.22-224.64)	96.65(82.38-112.78)	7801.04(6585.52-9176.06)	19851.02(17283.17-22824.01)	15.65(9.21-22.59)	22.09(11.46-41.93)	77.12(47.75-123.58)	289.29(155.13-529.62)	633.38(356.5-1171.31)	1998.97(1265.14-3119.78)	315.74(183.95-539.83)
Eritrea	Male	51.05(43.59-59.75)	189.89(162.08-221.48)	81.77(66.49-98.34)	7257.4(6111.45-8649.41)	19813.53(16910.16-23240.94)	23.79(16.83-31.14)	28.2(15.71-42.82)	72.91(40.38-107.84)	222.89(118.46-440.88)	829.16(489.14-1227.34)	2090.59(1235.05-3082.49)	257.8(152.37-475.33)
Estonia	Both	302.12(261.47-354.59)	592.95(509.96-686.73)	134.1(115.98-153.4)	33354(29927.82-37230.26)	38768.7(35561.99-42422.55)	38.38(31.82-45.69)	8.63(6-12.01)	65.33(44.29-96.76)	810.57(641.71-1013.02)	313.97(236.07-400.37)	1160.17(838.42-1574.77)	431.07(335.87-539.08)
Estonia	Female	192.78(164.8-226.85)	300.84(255.66-357.9)	82.34(64.06-100.28)	18569.27(16538.01-20836.78)	20721.64(18808.56-22820.17)	33.69(26.62-40.97)	5.13(3.53-7.19)	44.68(29.84-68.2)	879.87(677.93-1118.43)	184.23(138.26-234.51)	694.27(497.89-959.19)	403.28(301.86-531.35)
Estonia	Male	109.34(92.33-130.66)	292.11(249.9-337.77)	226.58(193.69-263.59)	14784.73(13024.34-16752.22)	18047.06(16310.15-19816.24)	44.26(36.26-52.53)	3.49(2.39-4.77)	20.66(13.67-30.37)	666.25(536.48-820.39)	129.74(95.78-168.43)	465.89(330.66-631.7)	451.99(358.71-561.51)
Eswatini	Both	69.05(60.07-79.19)	180.32(157.17-206.81)	87.05(76.74-98.76)	4977.35(4370.86-5714.98)	9952.21(8851.61-11244.13)	43.99(38-50.48)	22.76(15.84-30.46)	66.27(44.25-90.44)	58.71(31.37-95.77)	596.43(416.12-800.43)	1755.33(1161.64-2463.28)	92.12(52.21-144.84)
Eswatini	Female	30.48(26.28-35.16)	83.65(71.76-96.58)	101.75(87.99-116.11)	2656.25(2283.32-3077.1)	5203.3(4623.54-5866.3)	43.86(35.77-52.88)	8.98(5.57-12.65)	25(15.08-36.74)	53.62(11.64-134.8)	224.89(145.49-314.57)	603.87(369-904.08)	78.24(23.48-170.81)
Eswatini	Male	38.57(33.21-44.49)	96.67(83.54-111.89)	74.6(61.18-89.53)	2321.1(2025.78-2678.64)	4748.91(4139.41-5447.3)	44.2(37.98-50.67)	13.77(9.25-18.95)	41.27(27.44-56.63)	61.76(24.68-108.48)	371.53(253.74-509.71)	1151.46(751.99-1600.3)	99.77(53-161.78)
Ethiopia	Both	2313.1(2041.56-2592.04)	5877.62(5210.82-6569.03)	38.9(32.58-46.03)	268384.8(238288.18-301366.47)	572539.79(515848.21-634058.59)	3.39(-0.65-8.05)	1741.44(1288.25-2205.92)	2505.17(1941.16-3119.09)	133.31(82.2-195.28)	46149.28(34303.68-58649.07)	55828.59(44066.04-68961.1)	103.79(59.03-159.33)
Ethiopia	Female	974.81(854.74-1105.09)	2727.99(2416.44-3095.19)	54.51(47.07-63.02)	133185.07(117512.14-149444.46)	276691.88(247933.14-305978.73)	0.72(-4.72-6.67)	700.12(471.26-949.22)	1046.58(796.82-1312.3)	146.18(78.96-244.92)	19650.2(13500.28-26446.84)	24154.75(18646.69-30265.65)	105.49(50.21-179.54)
Ethiopia	Male	1338.3(1178.77-1505.24)	3149.64(2803.41-3526.2)	27.35(19.6-35.83)	135199.73(120698.26-151091.05)	295847.91(265550.57-329091.49)	6.03(2.38-10)	1041.32(717.71-1411.86)	1458.59(1065.5-1903.05)	124.39(65.8-203.31)	26499.08(18274.84-36107.81)	31673.84(23289.05-41056.1)	102.49(45.39-183.71)
Fiji	Both	98.07(83.74-112.36)	263.34(227.33-297.98)	112.7(94.91-130.63)	11017.29(9579.82-12857.72)	18530.06(16604.73-20696.22)	39.87(30.6-49.55)	38.26(28.83-50.24)	104.72(78.38-134.22)	69.17(38.18-100.14)	1201.86(898.89-1567.2)	2978.14(2260.08-3857.47)	85.03(55.46-118.47)
Fiji	Female	40.53(34.16-47.2)	112.02(96.17-127.3)	121.35(101.47-146.82)	5629.31(4854.37-6653.15)	9391.86(8376.66-10531.53)	38.36(27.98-49.39)	16.2(11.01-22.81)	45.55(33.7-60.44)	61.18(26.9-112.99)	535.19(377.96-739.91)	1332.18(1005.73-1728.1)	80.11(45.24-127.73)
Fiji	Male	57.54(48.97-66.05)	151.33(129.66-173.04)	106.12(85.75-128.02)	5387.97(4649.58-6267.85)	9138.2(8149.72-10314.91)	41.44(33.13-51.12)	22.06(16.59-29.18)	59.17(43.32-78.04)	75.7(35.06-115.69)	666.67(490.72-881.57)	1645.96(1222.29-2161.15)	89.28(46.33-130.98)
Finland	Both	1504.28(1298.96-1736.76)	3160.4(2777.1-3570.11)	94.88(76.15-115.03)	55287.42(49901.61-60348.78)	89975.25(82561.09-97200.63)	45.85(38.91-53.22)	61.07(45.69-78.91)	193.98(136.62-262.6)	179.34(129.98-235.07)	1513.97(1198.65-1870.83)	3276.93(2571.3-4099.98)	116.57(90.83-147.61)
Finland	Female	983.44(824-1146.61)	1834.8(1576.31-2100.2)	76.32(56.45-101.41)	30780.74(27666.72-33830.75)	48077.65(43810.23-52552.33)	42.81(35.1-51.29)	37.53(28.03-48.69)	117(79.84-160.73)	176(120.6-237.22)	885.32(700.08-1096.79)	1829(1414.21-2322.79)	101.81(73.26-136.08)
Finland	Male	520.84(446.36-601.44)	1325.6(1148.66-1530.47)	131.29(104.03-160.27)	24506.68(21870.84-27171.23)	41897.6(38237.6-45749.9)	49.9(39.92-59.71)	23.53(17.09-30.83)	76.98(54.6-106.15)	185.7(134.19-247.87)	628.65(487.91-783.85)	1447.93(1110.34-1810.91)	135.61(104.46-175.13)
France	Both	22876.21(20395.52-25855.24)	40874.62(36344.68-45861.31)	56.68(43.48-69.39)	658055.67(600637.16-716651.08)	1004174.37(919723.68-1089511.46)	33.48(25.58-41)	1268.32(886.19-1815.28)	2263.71(1535.79-3296.33)	52.09(23.1-86.92)	24089.51(18358.93-30857.17)	37487.65(28345.72-48687.26)	49.33(31.39-68.77)
France	Female	13198.68(11674.2-15020.08)	21740.63(19042.8-24468.82)	43.97(29.95-58.51)	352919.7(320105.2-387122.2)	526975.82(480155.45-577263.08)	29.88(21.07-37.97)	728.65(498.89-1041.23)	1242.75(801.06-1861.15)	38.77(6.58-78.19)	13117.62(9790.9-16869.09)	19558.82(14663.25-25310.5)	34.05(13.68-55.22)
France	Male	9677.53(8384.99-11225.73)	19133.99(16706.16-22141.03)	74.01(52.9-97.49)	305135.96(275098.55-336406.72)	477198.55(431876.6-525532.43)	37.66(27.77-47.55)	539.67(375.16-777.81)	1020.96(705.65-1462.53)	68.57(36.6-107.18)	10971.89(8197.41-14186.13)	17928.83(13561.97-23300.19)	66.75(43.91-93.31)
Gabon	Both	85.04(74.23-95.85)	236.5(206.27-267.98)	65.58(54.66-76.1)	7379.67(6510.95-8411.38)	15501.39(13804.65-17529.08)	19.84(15.27-25.26)	46.97(31.3-64.71)	98.29(55.82-138.51)	86.6(41.45-135.58)	1096.73(747.3-1488.54)	2233.87(1342.64-3140.25)	102.13(53.02-159.1)
Gabon	Female	40.59(35.13-46.89)	116.15(100.69-134.04)	69.4(58.24-81.96)	3741.46(3284.3-4276.7)	7846.38(6952.94-8919.75)	16.53(10.79-22.66)	20.08(9.36-29.54)	49.78(17.97-78.02)	115.89(38.32-180.36)	445.99(222.77-646.67)	1042.29(423.76-1598.43)	121.12(47.89-191.69)
Gabon	Male	44.45(38.49-50.91)	120.35(103.48-137.57)	62.17(47.64-76.91)	3638.21(3163.43-4194)	7655.01(6765.2-8736.69)	23.43(17.59-30.18)	26.89(17-37.1)	48.51(32.37-66.92)	63.84(20.18-139.43)	650.73(420.58-891.08)	1191.58(799.5-1665.21)	88.96(36.58-176.72)
Gambia	Both	58.57(51.91-65.81)	210.47(186.67-235.45)	65.3(55.71-76.28)	5156.55(4479.78-5871.1)	14118.6(12489.34-15877.71)	23.06(18.03-28.18)	22.47(15.57-30.79)	71.92(51.79-96.3)	130.84(74.94-206.62)	544.69(375.49-749.74)	1662.42(1193.03-2245.8)	176.93(95.59-281.91)
Gambia	Female	27.97(24.35-31.97)	107.26(92.64-121.74)	75.08(60.18-89.32)	2478.81(2125.26-2877.81)	7085.95(6251.65-7979.76)	25.76(18.77-33.38)	9.59(6.47-13.72)	33.01(23.33-45.65)	133.75(68.85-221.76)	228.21(156.16-327.29)	753.7(531.85-1026.52)	182.14(93.46-303.02)
Gambia	Male	30.6(26.63-34.81)	103.2(91.16-116.33)	56.38(43.25-69.76)	2677.74(2306.61-3086.77)	7032.65(6111.5-8049.04)	20.68(15.47-26.6)	12.88(8.65-18.2)	38.91(26.71-55.16)	128.68(65.9-226.52)	316.48(211.6-446.5)	908.72(626.28-1287.97)	174.52(88.49-300.95)
Georgia	Both	792.31(673.91-933.18)	1168.26(1028.67-1337.96)	110.47(88.38-134.07)	95304.61(85012.63-106943.87)	87367.03(79335.95-95804.4)	37.44(30.79-44.62)	102.64(76.28-130.22)	232.52(174.34-298.45)	165.58(127.67-209.19)	3172.86(2444.11-3946.89)	5648.74(4335.97-7086.1)	167.68(133.68-208.2)
Georgia	Female	444.08(380.35-525.7)	600.2(520.07-694.68)	94.57(71.61-122.09)	51280.31(45560.33-57677.45)	46250.95(41891.79-51058.22)	36.28(28.35-44.48)	50.63(37.5-64.6)	115.97(87.07-149.82)	173.25(130.93-221.75)	1512.37(1171.69-1878.79)	2596.24(2007.45-3246.49)	163.43(127.98-207.32)

Georgia	Male	348.24(291.2-413.76)	568.05(489.22-648.77)	130.68(104.31-162.11)	44024.3(38654.27-50242.26)	41116.08(36900.75-45453.81)	38.8(31.1-47.1)	52(37.83-66.8)	116.55(86.52-151.31)	158.32(117.86-205.73)	1660.49(1243.43-2113.55)	3052.5(2316.41-3851.66)	171.77(132.48-216.66)
Germany	Both	42200.38(37448.99-47298.6)	70549.67(64277.73-77316.59)	54.04(41.69-68.71)	1149767.79(1053697.06-1254995.69)	1643744.64(1532810.49-1762135.56)	34.01(27.96-40.23)	2551.1(1867.23-3383.42)	9486(6730.27-13068.63)	249.65(177.04-336.82)	58385.14(45599.64-73229.13)	131186.39(100603.97-171012.34)	129.34(94.08-168.3)
Germany	Female	26379.17(23260.89-29831.86)	37587.85(33951.11-41542.59)	34.68(23.25-48.12)	657697.33(600310.11-724195.59)	854290(788488.2-922516.88)	25.55(18.99-32.46)	1628.9(1195.07-2165.06)	5825.65(4029.05-8048.71)	254.62(174.44-355.2)	35194.22(27460.1-44159.25)	73518.85(55086.36-95808.04)	116.72(76.79-159.09)
Germany	Male	15821.21(13861.94-18002.56)	32961.82(29854.83-36666.59)	86.58(69.35-106.67)	492070.45(443193.2-543792.39)	789454.63(730179.69-850219.05)	45.47(38.01-54.35)	922.21(667.79-1239.52)	3660.35(2612.18-5147.17)	252.16(186.19-332.02)	23190.92(17525.17-29299.9)	57667.55(43823.04-75752.3)	149.66(109.43-197.82)
Ghana	Both	914.9(799.64-1041.76)	3439.83(3005.59-3885.05)	92.92(80.46-105.84)	86943.81(75337.9-101702.67)	236185.02(208372.33-268588.79)	31.57(26.29-37.25)	379.39(265.58-520.48)	1227.9(839.49-1698.98)	134.22(67.14-200.41)	9700.79(6659.83-13540.67)	30058.18(20494.11-41948.62)	167.73(87.2-249.84)
Ghana	Female	520.47(450.03-601.36)	1987.2(1710.92-2260.82)	92.98(77.55-109.87)	45429.99(39382.22-52756.23)	126419.93(112413.34-142988.72)	31.36(24.07-38.86)	220.38(142.41-340.04)	669.34(454.24-901.98)	123.08(48.18-205.4)	5742.71(3698.11-8679.58)	16158.89(11142.36-21970.59)	143.81(60.23-237.06)
Ghana	Male	394.43(343.78-452.7)	1452.63(1262.24-1658.64)	92.06(75.31-107.97)	41513.82(35417.47-48988.85)	109765.09(94569.3-127827.7)	31.56(25.66-37.41)	159.02(106.58-220.95)	558.56(328.12-818.58)	150.95(72.71-239.06)	3958.08(2673.71-5515.58)	13899.28(8289.09-20478.3)	202.64(107.8-313.35)
Greece	Both	6030.39(5274.54-6788.83)	8537.65(7595.1-9489.06)	43.39(33.97-54.22)	180264.17(146562.34-222474.38)	244053.02(203154.53-295212.96)	35.7(26.98-43.91)	675.49(493.88-928.28)	983.12(682.07-1388.52)	3.51(-12.98-20.69)	12540.21(9426.9-16210.48)	15590.29(11846.99-20338.07)	12.07(-1.3-25.96)
Greece	Female	3363.78(2915.58-3808.89)	4646.55(4091.51-5191.88)	38.27(25.26-51.76)	91881.94(75798.08-112510.37)	124818.55(104954.35-148965.65)	34.63(25.43-44.92)	388.88(282.65-534.54)	539.4(364.03-767.03)	-5.86(-24.41-16.2)	6997.25(5269.03-9077.41)	8081.21(6006.29-10605.4)	1.86(-13.92-19.47)
Greece	Male	2666.61(2295.8-3041.18)	3891.1(3405.83-4428.83)	49.71(37.43-62.53)	88382.23(69642.14-111923.28)	119234.47(96745.95-146155.41)	36.89(26-46.71)	286.61(207.77-400.68)	443.72(312.11-621.59)	15.08(-2.22-35.61)	5542.97(4158.94-7262.12)	7509.08(5690.55-9817.4)	24.69(10.16-40.24)
Greenland	Both	6.91(6.1-7.85)	20.06(17.71-22.62)	200.83(178.43-227.91)	290.79(252-339.01)	679.72(613.88-748.64)	128.68(109.28-149.71)	0.81(0.6-1.05)	1.51(1.04-2.08)	70.64(41.94-102.48)	21.9(16.67-27.8)	39.06(28.39-52.45)	106.71(76.72-138.24)
Greenland	Female	3.24(2.79-3.73)	8.5(7.45-9.69)	165.02(140.4-196.25)	118.11(99.21-140.39)	307.34(275.8-341.51)	147.34(121.72-178.89)	0.43(0.3-0.58)	0.7(0.48-0.98)	62.87(21.55-104.5)	10.47(7.82-13.6)	16.82(12.36-22.21)	88.05(48.89-128.96)
Greenland	Male	3.67(3.17-4.25)	11.57(10.09-13.27)	234.37(202.22-272.91)	172.68(148.99-201.26)	372.38(334.7-413.44)	116.53(97.19-137.61)	0.39(0.27-0.52)	0.82(0.54-1.16)	82.65(43.66-135.47)	11.42(8.33-15.08)	22.24(15.49-30.39)	123.86(83.62-175.17)
Grenada	Both	18.2(16.19-20.41)	51.37(44.88-57.62)	129.3(112.19-147.65)	1029.38(922.54-1141)	1957.26(1785.57-2142)	57.05(49.41-65.53)	7.39(5.96-9.04)	14.31(11.44-17.44)	94.77(69.16-121.5)	160.07(129.19-193.25)	364.86(290.95-447.45)	106.48(82.8-130.99)
Grenada	Female	8.76(7.63-9.99)	21.37(18.58-24.45)	107.15(84.61-129.39)	533.29(474.44-592.35)	902.31(816.58-996.02)	45.29(37.28-54.02)	3.35(2.69-4.11)	6.58(5.2-8.05)	108.13(77.88-140)	71.42(57.91-86.41)	157.55(126.67-194.75)	111.26(81.67-142.01)
Grenada	Male	9.44(8.26-10.73)	30(25.95-33.86)	146.78(118.74-174.48)	496.09(442.65-552.18)	1054.94(953.07-1161.44)	68.88(58.67-79.6)	4.04(3.19-4.97)	7.73(6.13-9.55)	83.37(57.42-111.61)	88.65(70.78-107.29)	207.31(163.16-255.01)	101.63(75.04-129.24)
Guam	Both	18.92(16.21-22.05)	59.87(52.1-68.24)	129.12(112.19-146.89)	2107.08(1823.51-2431.55)	3884.39(3523.47-4280.72)	46.03(34.32-58.61)	4.93(3.9-5.98)	19.32(15.1-24.12)	95.37(69.11-124.59)	147.74(118.24-177.46)	505.32(395.51-624.9)	120.38(94.28-149.26)
Guam	Female	8.84(7.41-10.39)	27.13(23.26-31.34)	119.51(99.18-143.61)	1027.28(882.64-1196.61)	1958.18(1761.24-2180.06)	45.8(33.3-59.21)	2.77(2.16-3.43)	10.13(7.74-12.73)	66.8(39.98-99.63)	81.05(64.69-99.18)	258.85(200.01-320.2)	96.58(68.08-129.35)
Guam	Male	10.08(8.59-11.8)	32.74(28.38-37.74)	137.76(115.16-162.77)	1079.8(921.98-1252.95)	1926.21(1723.36-2133.35)	46(34.8-59.02)	2.16(1.64-2.73)	9.18(6.99-11.84)	125.59(86.22-171.97)	66.69(51.85-83.11)	246.47(188.45-314.24)	146.59(108.29-189.09)
Guatemala	Both	1211.5(1060.12-1397.03)	5601.27(4972.92-6378.06)	148.62(131.83-167.01)	78074.84(68521.81-89114.48)	262691.24(236101.56-291324)	54.54(47.38-61.96)	259.98(200.68-324.98)	1731.7(1264.97-2274.13)	412.04(355.62-481.81)	7098.24(5539.71-8820.12)	42109.77(31666.34-54731.08)	461.51(393.32-544.03)
Guatemala	Female	538.25(467.69-620.85)	2733.05(2401.96-3106.22)	167.02(147.49-188.08)	37524.18(32488.37-43556.96)	131824.5(118104.82-147221.54)	56.58(47.51-66.27)	127.46(97.33-159.22)	894.94(656.16-1165.53)	436.58(369.64-515.88)	3470.11(2678.2-4331.35)	21909.23(16316.62-28611.74)	500.44(414.66-604.7)
Guatemala	Male	673.25(572.84-787.25)	2868.22(2487.3-3265.56)	134.32(112.96-156.99)	40550.66(35570.71-46287.61)	130866.74(116785.59-146899.95)	53.05(46.25-60.73)	132.52(98.5-169.98)	836.76(615.7-1122.8)	387.98(324.8-463.85)	3628.12(2740.08-4670.82)	20200.55(15086.95-26542.73)	425.43(354.13-511.56)
Guinea	Both	554.43(488.41-625.48)	1186.34(1046.05-1351.43)	11.35(4.59-18.4)	38811.98(34188.75-43351.25)	75601.86(66445.56-84180.1)	-3.44(-6.56--0.21)	259.84(176.93-362.56)	427.56(304.62-573.88)	56.62(27.87-93.85)	6001.6(4107.9-8212.71)	10125.34(7179.95-13808.66)	76.28(46.14-117)
Guinea	Female	284.27(247.13-327.16)	581.89(506.24-667.16)	5.97(-1.51-14.31)	20160.61(17655.03-22824.86)	38304.39(33547.49-43262.11)	-6.92(-10.8--3.09)	134.73(88.92-205.77)	191.95(133.94-273.28)	42.59(11.09-88.19)	3242.05(2140.95-4901.41)	4668.42(3239.31-6629.76)	53.9(19.45-98.73)
Guinea	Male	270.16(236.86-305.73)	604.45(528.75-694.5)	17.09(7.54-28.05)	18651.37(16368.48-20869.97)	37297.47(32519.76-41997.14)	0.37(-3.48-4.66)	125.11(83.41-187.59)	235.6(164.08-322.13)	71.23(29.52-130.91)	2759.55(1847.36-4163.21)	5456.92(3719.2-7482.29)	102.31(54.41-166.84)
Guinea-Bissau	Both	72.98(64.36-82.5)	155.45(135.84-178.52)	21.61(14.06-29.36)	5677.89(4930.17-6474.06)	11614.76(10118.46-13231.55)	10.42(6.57-14.81)	46.38(32.6-62.37)	66.33(47.27-90.53)	65.55(35.89-103.51)	1199.3(836.04-1639.56)	1796.81(1261.36-2439.76)	92.1(55.21-137.55)
Guinea-Bissau	Female	35.07(30.46-40.61)	80.06(68.22-92.87)	30.98(21.48-40.73)	2980.89(2575.36-3413.55)	6049.85(5277.89-6894.98)	9.44(4.38-15.13)	18.94(12.34-27.45)	30.14(20.12-43.51)	78.39(37.08-139.76)	518.63(338.1-759.71)	823.97(556.96-1212.19)	98.29(51.43-161.66)
Guinea-Bissau	Male	37.91(33.36-42.56)	75.38(65.91-86.56)	12.91(4.07-23.31)	2697(2338.24-3082.63)	5564.91(4803.27-6426.9)	11.5(6.54-16.93)	27.44(18.38-37.28)	36.19(25.64-48.5)	56.65(22.02-108.54)	680.67(458.88-953.8)	972.84(684.43-1318.59)	87.45(44.04-152.97)
Guyana	Both	89.32(77.89-101.94)	238.71(209.01-272.28)	167.77(148.92-187.67)	6974.07(6072.76-7955.59)	10942.09(9887.78-12033.18)	57.66(47.35-68.87)	27.63(21.33-34.54)	76.97(55.51-102.03)	170.9(134.72-213.64)	774.68(606.67-971.22)	2172.92(1578.16-2892.98)	221.98(178.97-273.43)
Guyana	Female	42.53(36.92-49.1)	114.3(99.28-131.33)	170.29(145.97-196.58)	3446.97(3002.95-4012.48)	5371.59(4815.24-5968.53)	56.28(43.97-69.16)	12.64(9.59-15.9)	37.52(26.76-49.64)	183.72(143.2-233.2)	355.6(277.09-448.09)	1048.11(762.69-1396.04)	236.52(189.09-297.93)
Guyana	Male	46.79(40.16-53.64)	124.41(107.29-144.71)	165.34(141.3-189.69)	3527.1(3048.39-4021.81)	5570.49(4970.68-6146.4)	59.04(48.71-70.54)	14.99(11.51-19.01)	39.45(28.05-53.08)	159.29(124.02-201.27)	419.08(321.72-530.28)	1124.81(803.34-1507.25)	209.52(166.5-259.06)
Haiti	Both	663.57(574.94-766.67)	2027.31(1764.87-2313.4)	64.21(54.68-73.75)	54432.76(47611.34-62213.68)	124830.43(110803.88-140010.05)	17.99(13.62-22.86)	264.35(180.02-410.54)	565.39(363.02-901.43)	79.76(49.04-117.91)	7610.44(5302.65-11202.89)	16383.89(10897.69-25611.26)	101.18(65.21-146.93)
Haiti	Female	315.76(268.82-367.56)	1012.35(866.79-1168.99)	72.69(60.52-85.72)	27462.57(23629.29-31720.42)	63727.95(56333.34-72253.75)	18.87(12.72-25.5)	116.2(75.48-198.07)	273.74(160.35-472.46)	95.05(44.68-153.37)	3509.71(2379.83-5484.1)	8347.57(5305.53-13848.02)	117.59(70.52-176)
Haiti	Male	347.81(298.01-404.21)	1014.96(882.9-1170.1)	56.49(45.76-68.71)	26970.19(23567.51-30646.27)	61102.48(53911.53-69002.01)	17.1(12.16-22.37)	148.15(93.04-246.24)	291.65(182.46-472.61)	67.99(33.91-114.2)	4100.73(2674.89-6412.19)	8036.32(5203.55-12571.52)	87.04(47.51-140.23)

Honduras	Both	634.89(547.71-730.42)	2663.99(2339.62-3027.82)	128.25(114.57-144.15)	45208.76(39517.41-51498.63)	134326.05(120669.71-149436.11)	44.74(38.02-51.91)	134.02(93.15-203.67)	931.93(677.94-1261.82)	266.07(188.5-344.12)	3731.11(2723.69-5247.74)	23159.05(17085.71-30684.85)	365.69(282.72-455.5)
Honduras	Female	311.84(269.25-361.06)	1341.15(1174.69-1538.63)	128.77(110.13-149.67)	22416.18(19409.67-26138)	67398.3(59796.41-75468.69)	42.49(33.39-51.54)	62.71(44.42-90.44)	506.91(360.78-691.63)	293.48(200.5-411.91)	1838.7(1349.31-2464.05)	12794.44(9347.59-17352.55)	385.31(286.02-513.8)
Honduras	Male	323.05(275.79-373.87)	1322.84(1139.37-1511.31)	127.93(110.79-146.09)	22792.58(19754.21-25982.58)	66927.75(59686.78-75054.86)	47.29(40.63-54.71)	71.31(45.34-123.64)	425.03(303.8-599.52)	235.78(136.91-351.76)	1892.41(1289.99-3039.44)	10364.61(7545.79-14317.27)	340.44(226.23-475.63)
Hungary	Both	2376.26(2074.67-2708.63)	5620.03(4923.71-6395.47)	161.19(141.59-181.81)	167457.03(151544.91-184351.25)	224771.75(206459.16-244932.81)	44.33(37.53-51.16)	190.77(139.19-243.38)	438.57(311.87-599.57)	155.49(115.23-201.42)	5235.27(3976.85-6461.84)	9260.24(7015.77-11950.33)	123.29(96.75-154.35)
Hungary	Female	1327.36(1145.51-1516.26)	3063.02(2666.45-3531.7)	150.64(126.28-177.91)	91278.48(81736.28-102028.72)	124839.24(113318-137602.61)	45.67(37.56-54.16)	115.36(84-147.1)	292.49(204.08-403.74)	154.65(106.74-207.91)	2944.54(2242.69-3601.67)	5466.75(4152.29-7076.05)	114.85(85.17-147.71)
Hungary	Male	1048.9(900.71-1203.96)	2557.01(2170.48-2957.76)	174.45(146.42-205.44)	76178.55(68062.48-85046.21)	99932.51(90540.1-109621.67)	42.57(35.22-49.9)	75.4(54.86-97.14)	146.08(102.36-201.84)	137.59(102.51-179.47)	2290.72(1721.04-2876.88)	3793.48(2860.93-4912.71)	125.48(96.58-158.75)
Iceland	Both	71.56(63.97-80.69)	146(129.3-163.52)	53.94(41.68-67.72)	2419.5(2199.14-2643.43)	4387.02(4029.47-4744.34)	32.07(26.77-38.21)	1.85(1.32-2.65)	4.83(3.27-7.08)	113.9(76.3-152.33)	49.35(38.02-63.37)	103.76(79.3-135.54)	71.84(52.25-92.88)
Iceland	Female	38.92(34.1-44.83)	73.12(63.85-83.12)	42.61(28.74-57.58)	1255.48(1129.43-1384.94)	2199.35(2004.33-2415.04)	28.87(22.28-36.07)	0.95(0.66-1.36)	2.27(1.45-3.37)	107.86(62.06-161.36)	24.7(18.81-31.96)	48.42(37.07-63.85)	61.6(38.44-88.23)
Iceland	Male	32.64(28.87-36.92)	72.88(63.41-82.79)	67.4(52.19-87.06)	1164.02(1057.07-1272.25)	2187.67(1997.41-2395)	35.49(29.09-42.81)	0.9(0.62-1.29)	2.56(1.76-3.75)	121.26(83.18-166.56)	24.64(18.67-32.49)	55.34(40.7-73.2)	82.02(56.84-109.55)
India	Both	95772.84(84971.11-107757.91)	278934.05(248867.2-310803.99)	100.83(91.9-110.91)	10180697.49(9152554.9-11349692.87)	22293223.32(20283593.51-24381117.29)	35.73(31.15-40.64)	19984.77(14287.85-25891.59)	60989.67(45051.06-77995.26)	168.85(132.5-223.68)	601244.55(446100.75-773108.07)	1648771.58(1227573.28-2117240.11)	214.69(170.4-276.55)
India	Female	38220.22(33788.6-43108.23)	124970.13(110839.15-140315.18)	125.06(113.78-137.63)	4499405.75(4049380.6-5033146.43)	10174149.44(9293424.27-11055782.11)	37.26(30.8-43.43)	7501.99(5127.04-9994.32)	25394.67(17795.21-33271.61)	199.32(144.94-305.84)	228397(164591.65-302661.61)	685851.19(497595.49-883274.93)	248.11(183.47-351.52)
India	Male	57552.62(51124.1-64669.85)	153963.92(137691.84-171802.59)	84.81(76.02-94.42)	5681291.73(5137680.43-6323846.1)	12119073.88(11024444.71-13330821.19)	34.88(31.08-39.09)	12482.78(8815.51-16606.92)	35595(25953.61-47728.25)	150.18(109.59-201.69)	372847.55(271602.65-488007.29)	962920.39(698414.51-1289117.9)	193.07(143.29-252.92)
Indonesia	Both	15208(13505.49-17145.56)	48064.6(42760.36-54217.1)	135.99(127.14-144.32)	2535141.07(2263853.79-2847498.95)	5129531.34(4645112-5652616.01)	46.65(40.37-53.46)	6265.74(4922.99-7665.56)	13934.73(10814.84-17434.3)	78.94(55.19-104.59)	203041.34(157198.49-249576.82)	428025.79(329027.16-539662.89)	133.08(102.04-168.56)
Indonesia	Female	8008.25(7097.37-9033.14)	25139.16(22361.18-28270.08)	136.19(126.11-146.33)	1247201.64(1107806.47-1409223.18)	2461988.21(2229487.48-2697513.92)	44.06(36.56-51.99)	2946.02(2243.77-3743.89)	5906.27(4310.41-7662.21)	64.69(35.54-96.96)	98222.37(74753.54-123550.3)	186903.5(140512.07-243592.09)	113.7(76.8-157.3)
Indonesia	Male	7199.75(6366.42-8159.75)	22925.44(20390.81-25780.16)	136.02(126.23-145.9)	1287939.43(1153207.26-1446260.25)	2667543.13(2403892.05-2967893.79)	49.08(43.36-55.21)	3319.72(2566.98-4131.15)	8028.47(5944.5-10590.04)	91.09(60.62-119.54)	104818.97(79045.94-132885.8)	241122.29(177357.27-319095.28)	150.46(111.14-194.68)
Iran (Islamic Republic of)	Both	11474.91(9991.79-13062.77)	40489.57(36549.61-44830.82)	143.92(129.33-160.5)	483270.17(440799.83-527770.54)	1371661.76(1268553.42-1475169.91)	91.92(84.39-100.33)	1409.07(1114.25-1704.82)	3891.35(3150.17-4647.09)	152(112.34-191.19)	37969.07(30415.96-44854.87)	89013.53(73929.53-103507.37)	196.16(154.04-244.76)
Iran (Islamic Republic of)	Female	5583.76(4874.17-6391.19)	20482.81(18487.06-22695.19)	150.38(133.86-167.78)	240761.83(218947.22-263638.2)	696556.97(643342.17-748797.85)	94.5(85.45-103.63)	632.59(481.57-821)	1845.63(1454.57-2205.46)	156.28(88.61-205.78)	16882.02(13268.8-20564.54)	41973.46(34463.66-49359.08)	196.1(138.26-256.34)
Iran (Islamic Republic of)	Male	5891.15(5108.18-6718.81)	20006.76(18071.5-22102.24)	137.78(122.6-155.28)	242508.34(222026.47-264612.18)	675104.79(623221.55-725936.86)	89.31(82.1-97.43)	776.48(612.1-946.03)	2045.72(1643.29-2469.17)	147.11(106.84-197.13)	21087.04(16755.05-25372.38)	47040.07(39067.39-55244.13)	195.95(150.3-250.47)
Iraq	Both	3152.63(2769.03-3563.03)	15498.69(13642.45-17454.27)	111.64(100.27-122.84)	144651.44(129337.2-161897.31)	496302.62(449958.67-547121.84)	42.87(36.47-49.74)	1061.37(791.61-1443.39)	2613.79(1902.15-3467.13)	63.03(32.22-94.58)	25105.42(18998.71-32801.13)	65573.95(48920.27-86995.53)	98.96(57.21-144.74)
Iraq	Female	1691.44(1474.31-1921.63)	7475.94(6558.49-8435.73)	89.75(77.13-103.62)	77418.41(68680.03-87272.36)	254384.75(227522.49-282524.89)	37.01(29.82-44.87)	531.21(374.42-798.25)	1163.89(816.82-1593.48)	49.5(8.52-89.76)	12837.84(9189.08-18512)	29894.09(21356.22-40298.92)	72.32(25.53-128.03)
Iraq	Male	1461.19(1268.5-1657.41)	8022.75(7027.35-9153.39)	137.01(120.58-155)	67233.04(59413.25-75816.91)	241917.87(217694.47-270566.16)	49.63(41.53-57.83)	530.17(376.63-752.07)	1449.9(1011.44-2027.49)	77.22(40.4-122.69)	12267.58(8881.68-16372.4)	35679.85(25603.02-48236.78)	127.12(73.61-191.56)
Ireland	Both	1489.16(1328.15-1628.92)	2543.23(2425.29-2661.77)	27.66(16.98-41.88)	41298.89(37309.98-45322.99)	72150.04(67569.31-77416.68)	26.75(19.17-36.07)	87.31(62.31-120.96)	142.66(98.6-203)	55.41(28.18-87.48)	1854.58(1397.63-2404.25)	2654.81(2039.64-3422.41)	25.56(9.73-42.22)
Ireland	Female	811.82(720.94-882.12)	1247.16(1173.42-1325.75)	13.18(4.97-25.97)	22075.28(19676.97-24310.76)	35461.27(32814.9-38306.36)	16.08(8.67-26.92)	46.51(32.97-64.21)	72.71(48.66-105.82)	42.99(13.49-77.89)	971.99(740.49-1249.19)	1284.06(990.95-1665.75)	9.78(-4.87-27.27)
Ireland	Male	677.34(582.48-776.92)	1296.07(1210.89-1375.85)	45.3(28.11-65.32)	19223.6(17307.94-21261.26)	36688.77(34008.12-39535.12)	39.05(28.86-48.81)	40.8(28.22-58.01)	69.95(48.83-99.36)	68.92(39.87-106.79)	882.59(645.71-1160.21)	1370.76(1037.87-1769.36)	43.45(23.37-66.6)
Israel	Both	1918.2(1666.67-2138.41)	4861.35(4274.32-5429.26)	34.16(23.06-45.22)	50960.98(46199.41-55874.7)	121975.35(112338.61-132210.35)	27.53(22.38-32.88)	250.29(180.68-330.69)	756.83(560.63-1011.2)	83.84(55.31-114.9)	4604.53(3542.4-5803.07)	11776.02(9140.98-14702.2)	62.9(43.84-82.86)
Israel	Female	1030.12(897.22-1160.33)	2526.52(2220.9-2848.79)	30.26(18.61-44.21)	26236.98(23536.65-28876.03)	61889.76(56122.76-67876.93)	26.48(20.54-33.39)	130.52(94.64-172.51)	380.82(275.66-510.71)	68.25(35.88-106.08)	2405.27(1852.52-3034.53)	5638.79(4394.63-7050.31)	46.28(25.47-69.85)
Israel	Male	888.08(759-1003.85)	2334.84(2029.78-2646.9)	38.68(24.23-55.77)	24723.99(22159.19-27291.13)	60085.59(54856.54-65638.48)	28.63(21.97-35.95)	119.77(85.42-160.04)	376.01(281.09-499.59)	100.74(71.03-137.8)	2199.26(1656.89-2819.18)	6137.23(4680.47-7807.68)	81.57(59.29-106.32)
Italy	Both	26276.64(23264.56-29621.94)	42915.13(38487.98-47598.44)	61.21(52.27-70)	812826.24(753212.91-875814.7)	1180175.62(1095663.12-1268441.97)	36.13(32.99-39.28)	1595.78(1172.06-2136.04)	2995.94(2044.64-4212.11)	55.49(25.27-87.9)	34684.17(26798.18-43775.23)	47246.48(36366.99-60633.94)	37.18(22.07-55.14)
Italy	Female	14289.02(12642.77-16004.21)	22638.22(20411.08-25064.17)	59.42(50.74-68.21)	434139.91(401847.39-469089.45)	616961.54(572578.08-664599.24)	33.89(30.43-37.34)	895.43(655.21-1209.42)	1754.94(1183.81-2492.43)	49.56(18.86-83.94)	18607.09(14563.81-23410.57)	25686.3(19438.34-32973.64)	31.31(15.18-49.84)
Italy	Male	11987.61(10464.99-13772.74)	20276.91(18105.36-22671.56)	63.45(52.14-74.15)	378686.33(349901.86-408200.94)	563214.08(519719.7-605501.63)	38.7(35.39-41.98)	700.35(505.38-949.3)	1241(860.09-1754.92)	59.22(29.33-91.79)	16077.08(12038.31-20619.39)	21560.18(16491-27826.36)	42.84(26.49-61.71)
Jamaica	Both	445.15(390.6-508.08)	1008.98(882.53-1144.22)	90.23(77.79-103.06)	26626.23(23898.92-29668.44)	47656.23(43249.69-52197.46)	49.71(42.67-57.07)	122.46(98.07-148.09)	271.42(205.84-350.86)	44.6(27.49-64.35)	2621.33(2137.85-3097.23)	6506.6(4992.26-8370.04)	94.2(71.22-122.96)
Jamaica	Female	216.82(188.69-248.85)	455.81(397.22-519.51)	78.41(60.48-99.05)	13426.01(11976.69-15155.57)	22984.01(20772.97-25368.24)	44.66(36.88-53.77)	47.24(37.89-57.04)	116.45(88.07-149.54)	65.28(44.01-91.79)	1074.59(879.31-1274.77)	2898.93(2221.9-3760.58)	114.58(85.8-146.38)

Jamaica	Male	228.33(197.7-263.4)	553.16(480.86-641.29)	100.97(87.61-115.07)	13200.22(11759.15-14752.77)	24672.22(22306.91-27323.8)	54.67(47.56-62.6)	75.22(59.51-91.53)	154.96(115.09-201.36)	30.91(14.28-50.25)	1546.74(1242.44-1839.81)	3607.67(2738.34-4639.32)	79.55(55.47-109.85)
Japan	Both	64964.1(59003.07-71969.86)	136480.47(122937.66-150493.26)	115.04(103.78-125.93)	2609935.46(2405927.87-2824404.71)	4367245.54(4060915.37-4677846.6)	63.54(57.61-69.8)	7311.9(5935.71-8661.91)	16135.71(11746.77-20340.23)	27.86(5.19-48.39)	157736.84(131523.34-182375.13)	257021.35(208187.22-304581.54)	37.28(22.42-51.62)
Japan	Female	31014.3(27801.43-34445.86)	59674.28(53750.62-66191.82)	97.18(86.57-107.24)	1274117.4(1172235.58-1381557.52)	2058584.49(1905523.2-2210284.76)	56.98(51.55-62.45)	3920.23(3143.55-4716.78)	8687.24(5929.25-11276.94)	19.62(-7.37-44.15)	79577.78(66043.91-92580.57)	121631.65(94584.92-146667.86)	26.18(7.71-43.68)
Japan	Male	33949.81(30783.93-37433.04)	76806.2(68847.99-85150.62)	131.27(115.04-145.77)	1335818.06(1227977.21-1444884.17)	2308661.05(2146826.7-2472284.41)	69.99(63.13-77.24)	3391.68(2777.7-4039.96)	7448.48(5794.45-9176.07)	35.66(17.28-52.24)	78159.06(64841.75-91456)	135389.7(111448.29-160545.16)	48.77(35.55-62.95)
Jordan	Both	543.62(477-613.19)	4693.15(4172.15-5242.18)	180.76(161.66-200.55)	26100.77(22987.47-29870.68)	139793.23(126842.03-153491.55)	72.07(61.09-83.81)	144.6(112.72-180.45)	624.83(475.81-787.58)	100.58(64.49-137.05)	3577.37(2812.63-4454.62)	15674.28(12280.92-19449.75)	110.8(76.23-153.31)
Jordan	Female	309.46(270.13-353.86)	2184.8(1924.03-2475.01)	133.05(113.38-152.45)	13627.74(12011.83-15629.38)	66829.59(60623.53-74123.81)	61.55(50.25-73.33)	81.1(59.46-105.99)	310.83(224.65-406.42)	102.62(51.28-150.65)	1984.21(1497.68-2582.06)	7560.29(5649.67-9792.58)	90.13(43.89-134.3)
Jordan	Male	234.17(201.73-268.53)	2508.34(2195.41-2867.78)	243.45(209.52-279.18)	12473.03(10853.34-14449.47)	72963.64(65976.38-81197.35)	83.58(69.48-98.52)	63.51(48.53-81.52)	314(225.97-428.66)	105.71(65.59-159.77)	1593.16(1220.8-2030.84)	8113.99(6060.4-10667.41)	136.71(84.64-203.29)
Kazakhstan	Both	1553.27(1311.9-1819.87)	3867.78(3296.21-4547.49)	116.01(101.62-131.76)	228610.69(201310.06-260053.22)	313333.12(281580.84-348328.18)	23.18(16.99-29.93)	236.12(177.37-291.06)	576.95(431.97-732.86)	128.03(98.1-157.53)	8084.93(6279.04-10000.26)	16630.56(12877.9-20818.64)	116.64(89.19-146.36)
Kazakhstan	Female	880.51(738.46-1039.45)	1936.13(1648.6-2245.98)	89.03(74.39-106.95)	122952.07(107239.91-139552.73)	163703.02(147555.38-182983.98)	19.23(12.69-25.87)	125.87(96.4-155.94)	273.13(204.16-351.44)	107.43(76.57-141.09)	4200.48(3311.15-5180.61)	7692.41(5992.51-9494.53)	91.64(65.35-120.61)
Kazakhstan	Male	672.77(561.43-802.94)	1931.65(1619.75-2283.24)	151.47(131.08-173.47)	105658.63(91552.52-122244.05)	149630.1(132478.65-168938.59)	27.8(20.26-35.99)	110.26(80.67-138.31)	303.82(224.92-391.73)	151.84(116.47-193.78)	3884.45(2942.26-4899.39)	8938.16(6753.12-11290.4)	143.62(109.37-180.35)
Kenya	Both	940.51(840.6-1053.28)	3150.82(2785.84-3547)	64.54(59.55-69.75)	113328.77(102409.74-125888.33)	311328.56(281971.77-344330.81)	28.03(23.86-32.61)	346.7(255.71-464.27)	1073.19(804.56-1368.11)	103.31(71.55-133.92)	8002.56(5973.51-10460.79)	26204.32(19589.6-33312.03)	156.55(116.18-199.18)
Kenya	Female	410.57(366.21-460.57)	1427.98(1261.11-1614.03)	73.46(68.15-78.71)	55738.81(50244.97-62099.21)	153294.81(138968.94-169001.35)	28.63(22.45-34.69)	151.04(112.66-206.6)	534.26(388.43-717.74)	138.73(87.01-184.56)	3579.99(2690.4-4860.23)	12542.69(9048.24-16877.51)	178.67(119.2-241.53)
Kenya	Male	529.94(473.62-590.02)	1722.85(1525.3-1943.96)	57.23(51.63-62.92)	57589.96(52075.87-63926.1)	158033.75(142883.06-175388.24)	27.43(23.67-31.26)	195.66(135.67-281.58)	538.93(371.05-736.85)	76.79(48.52-110.66)	4422.57(3117.31-6148.12)	13661.63(9533.8-18324.16)	138.59(95.05-189.33)
Kiribati	Both	7.18(6.07-8.47)	17.97(15.19-20.76)	48.94(39-60.04)	974.02(823.23-1132.79)	1754.05(1525.4-1988.44)	13.09(7.24-19.3)	5.31(3.91-6.85)	12.01(8.42-16.61)	83.2(42-124.37)	169.93(126.59-220.68)	386.4(265.7-529)	104.03(59.62-150.16)
Kiribati	Female	3.53(2.92-4.2)	8.62(7.19-10.09)	45.58(33.98-58.48)	532.32(447.3-623.81)	954.88(825.73-1089.83)	11(3.96-18.94)	3.27(2.34-4.33)	6.9(4.64-10.22)	69(31.65-120.03)	102.69(73.77-136.04)	214.22(143.11-318.05)	88.65(45.79-142.89)
Kiribati	Male	3.66(3.06-4.35)	9.35(7.86-10.89)	52.19(38.98-67.31)	441.7(373.18-513.87)	799.17(690.19-918.15)	15.4(9.64-21.82)	2.04(1.48-2.75)	5.11(3.11-7.25)	104.84(12.05-175.96)	67.24(48.86-89.89)	172.19(107.21-243.88)	128.48(34.44-199.93)
Kuwait	Both	258.81(223.43-294.13)	1627.96(1412.13-1867.66)	153.21(124.56-182.01)	14632.08(12583.22-17058.2)	59016.77(52459.15-66045.25)	56.99(46.26-70.35)	40.68(31.95-49.24)	100.46(75.53-128.37)	31.59(13.5-55.91)	1103.19(872.51-1335.32)	2783.31(2203.88-3463.74)	35.04(16.59-59.11)
Kuwait	Female	109.48(94.83-125.16)	673.29(574.18-776.07)	128.34(101.59-156.71)	6105.95(5185.96-7184.11)	26920.36(23634.19-30390)	57.19(45.13-71.3)	19.18(13.93-24.22)	41.52(29.81-56.11)	36.34(11.61-82.42)	494.72(378.42-613.29)	1206.21(921.28-1550.84)	16.08(-5.12-52.92)
Kuwait	Male	149.33(126.37-173.1)	954.66(823.08-1097.1)	175.93(140.69-214.02)	8526.13(7332.61-9955.65)	32096.41(28514.78-35778.87)	57.48(45.39-71.68)	21.51(16.95-26.01)	58.94(43.16-78.12)	34.87(15.79-56.59)	608.47(476.09-742.22)	1577.09(1206.9-2004.19)	50.42(28.94-77.4)
Kyrgyzstan	Both	335.73(282.32-393.87)	784.48(660.34-924.55)	54.48(41.63-68.69)	50863.16(44837.1-57740.27)	81914.07(73364.62-92395.49)	10.7(5.83-15.79)	90.18(65.95-114.67)	118.24(85.41-154.43)	31.03(13.72-51.69)	3069.36(2308.04-3907)	4102.79(3093.79-5342.65)	47.08(25.68-72.33)
Kyrgyzstan	Female	181.39(151.3-216.76)	384.75(324.93-455.89)	40.31(24.5-57.83)	26918.46(23634.82-30681.19)	41287.47(36578.22-46619.77)	6.24(0.66-12.22)	44.43(32.71-56.43)	57.13(42.04-73.91)	35.52(16.28-59.43)	1500.06(1129.06-1906.51)	1921.13(1445.33-2478.38)	41.21(20.61-68.43)
Kyrgyzstan	Male	154.35(128.38-183.63)	399.74(333.93-471.59)	71.13(54.8-90.12)	23944.7(20839.93-27652.76)	40626.6(35731.07-46619.77)	15.67(9.45-22.28)	45.74(32.87-59.73)	61.12(43.93-80.68)	27.66(8.34-49.85)	1569.3(1152.71-2039.49)	2181.66(1615.31-2868.03)	52.74(28.33-80.19)
Lao People's Democratic Republic	Both	447.85(388.76-511.12)	1331.91(1165.03-1515.45)	89.37(78.88-101.48)	50179.74(43969.14-57362.36)	111742.92(98291.92-125933.73)	30.56(25.52-35.98)	366.36(261.36-481.75)	632.88(463.96-831.36)	129.3(88.1-181.23)	10623.63(7583.51-14032.84)	17713.55(12868.37-23880.72)	175.97(123.2-247.99)
Lao People's Democratic Republic	Female	235.15(204.12-271.88)	693.39(602.03-801.12)	93.14(79.5-108.19)	25607.55(22268.9-29445.88)	54384.94(47773.47-61236.71)	26.95(20.37-34.27)	190.57(135.25-256.7)	311.29(225.44-416.17)	126.19(78.13-194.49)	5629.61(3939.25-7476.17)	8746.07(6344.28-11769.45)	163.34(107.55-244.37)
Lao People's Democratic Republic	Male	212.7(183.35-244.68)	638.52(550.99-730.71)	85.92(72.42-101.03)	24572.19(21205.21-28250.31)	57357.99(49734.34-65197.96)	34.16(28.62-40.42)	175.79(119.37-254.3)	321.59(226.12-444.8)	134.53(76.21-205.99)	4994.02(3374.29-7045.18)	8967.47(6157.11-12537.39)	191.2(115.9-283.63)
Latvia	Both	446.06(385.85-515.35)	702.95(615.31-804.44)	125.59(108.05-145.35)	57504.85(51712.35-64277.75)	55957.89(51242.67-60927.09)	34.23(27.34-41.24)	8.88(5.92-12.8)	33.31(21.81-49.15)	356.27(296.84-426.98)	397.26(297.21-526.36)	885.51(654.84-1167.09)	233.86(185.78-287.06)
Latvia	Female	286.85(243.33-335.27)	387.03(333.1-453.69)	90.34(72.26-108.57)	32101.12(28612.61-35848.65)	30940.07(28044.2-33976.44)	31.85(24.23-39.56)	5.17(3.43-7.46)	22.33(14.14-33.41)	385.33(315.89-471.8)	237.29(176.78-315.27)	529.54(381.7-715.32)	209.44(157.52-269.44)
Latvia	Male	159.21(137.35-186.28)	315.91(274.22-362.9)	188.99(159.6-224.79)	25403.73(22370.21-28863.66)	25017.81(22500.53-27623.69)	37.21(29.49-45.46)	3.71(2.48-5.35)	10.97(7.02-16.45)	294.34(237.75-360.03)	159.97(115.47-211.81)	355.97(255.91-483.52)	258.21(199.32-320.02)
Lebanon	Both	847.36(741.76-961.11)	3477.57(3130.01-3872.54)	154.41(134.81-175.86)	34825.48(31453.75-38468.17)	98679.87(90709.58-107262.29)	78.5(69.8-88.28)	194.88(147.2-250.05)	357.92(246.33-498.19)	22.09(-14.24-58.58)	4579.16(3505.86-5881.96)	7995.11(5870.79-10743.2)	55.28(16.47-94.27)
Lebanon	Female	460.46(400.43-530.01)	1801.25(1607.79-2012.21)	135.15(115.9-157.44)	18916(16925.41-21120.05)	53481.64(48855.71-58384.83)	73.37(63.13-83.4)	99.16(72.01-135.84)	193(122.93-258.42)	21.79(-16.3-56.63)	2348.38(1746.73-3159.25)	4269.25(3028.57-5560.05)	47.08(5.52-85.37)
Lebanon	Male	386.91(334.28-449.19)	1676.32(1490.64-1896.4)	177.34(151.92-204.39)	15909.48(14346.1-17657.02)	45198.23(41043.17-49572.43)	84.12(73.56-95.71)	95.72(70.74-126.26)	164.92(94.72-277.72)	19.91(-28.63-85.22)	2230.78(1646.82-2903.3)	3725.86(2450.27-5766.27)	61.38(8.22-135.31)
Lesotho	Both	190.75(167.77-216.32)	327.84(286.61-374.37)	47.94(39.9-57.19)	13163.84(11660.27-14878.51)	19378.32(17292.08-21763.06)	30.03(25.74-34.59)	47.35(31.95-65.94)	134.19(89.35-189.75)	54.94(21.58-93.95)	1205.31(828.56-1684.21)	3483.89(2312.28-5037.64)	86.26(44.27-138.69)
Lesotho	Female	82.54(72.32-94.63)	154.8(135.34-178.46)	63.48(49.82-78.15)	6782.86(5959.03-7750.46)	9965.78(8877.62-11191.24)	30.16(24.54-36.39)	17.08(11.47-24.63)	59.2(36.24-89.09)	78.31(21.69-149.59)	408.25(283.85-584.78)	1421.53(865.92-2149.02)	116.58(48.59-201.65)
Lesotho	Male	108.22(94.25-124.8)	173.05(149.34-200.78)	35.91(25.88-48.26)	6380.98(5581.36-7218.06)	9412.54(8236.33-10669.16)	29.9(24.27-35.44)	30.27(19.64-43.31)	74.99(47.08-107.11)	42.23(3.56-82.32)	797.07(526.25-1147.4)	2062.36(1285.09-3011.07)	71.7(20.79-124.71)

Liberia	Both	188.2(164.14-212.77)	442.12(387.3-505.57)	0.5(-6.17-8.51)	13583.58(12104.72-15308.05)	32660.77(28921.84-37076.48)	-0.9(-4.82-3.01)	103.82(74.8-137.93)	149.16(101.21-212.18)	86.19(43.31-141.37)	2358.31(1686.51-3114.85)	3677.04(2467.34-5281.74)	119.43(58.55-196.58)
Liberia	Female	88.33(76.26-102.1)	228.08(196.84-263.58)	12.55(3.25-23.1)	6628.04(5830.37-7530.14)	15690(13785.74-17766.28)	-0.37(-4.95-4.59)	47.46(34.1-63.33)	74.15(49.5-104.93)	93.94(39.4-158.34)	1125.1(804.53-1491.46)	1868.14(1248.79-2674.39)	123.28(56.01-207.23)
Liberia	Male	99.88(86.48-112.83)	214.03(185.41-245.98)	-10.09(-18.65--1.04)	6955.54(6207.61-7857.04)	16970.77(14873.26-19524.31)	-1.54(-6.58-3.23)	56.36(37.39-82.29)	75.01(46.86-121.6)	78.9(27.74-150.73)	1233.21(818.69-1784.45)	1808.89(1123.53-2934.3)	114.99(47.84-205.3)
Libya	Both	686.63(607.24-780.7)	3064.75(2715.88-3428.14)	181.44(163.93-199.3)	32447.87(28983.65-36496.74)	100544.28(91458.19-110262.58)	90.83(81.48-101.46)	151.88(104.34-209.56)	424.67(267.46-598.45)	74.32(37.36-115.17)	3600.99(2569.85-4726.34)	10521.07(7112.54-14248.76)	130.76(82.56-184.84)
Libya	Female	346.91(303.6-394.41)	1479.57(1304.45-1661.91)	165.89(146.34-189.71)	15884.54(14116.01-17997.78)	50826.24(46179.09-55945.4)	95.5(84.64-108.69)	77.34(50.69-110.7)	230.59(132.9-333.47)	84.95(34.63-132.03)	1866.73(1279.81-2583.03)	5745.33(3623.78-8064.81)	136.19(73.92-199.17)
Libya	Male	339.72(297.55-393.32)	1585.18(1395-1790.38)	197.52(172.32-222.14)	16563.33(14657.93-18803.59)	49718.04(44916.84-55146.45)	86.27(76.55-98.31)	74.54(50.82-106.63)	194.08(121.39-315.32)	62.96(20.72-125.65)	1734.26(1212.72-2398.26)	4775.74(3190.39-7376.79)	122.97(68.65-201.93)
Lithuania	Both	614.83(530.8-711.54)	899.29(789.27-1021.48)	92.3(73.7-114.34)	75254.05(66785.06-83770.49)	77077.25(69980.28-84815.05)	33.62(25.91-40.9)	13.96(9.42-19.89)	33.53(22.02-49.27)	141.6(111.26-177.71)	573.2(428.64-743.84)	1002.4(757.69-1295.28)	122.91(96.42-151.4)
Lithuania	Female	376.71(321.12-441.63)	479.77(416.54-564.59)	60.63(40.29-82.26)	40834.98(36166.56-46103.48)	41816.92(37720.9-45999.92)	30.7(22.17-39.09)	7.4(4.87-10.53)	20.32(13.53-30.24)	146.54(110.01-189.12)	321.56(243.14-416.91)	557.6(416.12-723.76)	103.83(75.46-136.54)
Lithuania	Male	238.12(204.15-276.61)	419.52(362.02-476.7)	143.31(117.5-177.77)	34419.07(30405.71-38945.85)	35260.33(31653.03-39096.79)	37.13(28.97-45.07)	6.56(4.54-9.33)	13.21(8.58-19.63)	127.35(96.11-164.94)	251.65(186.54-328.28)	444.79(328.86-595.99)	141.84(107.35-179.3)
Luxembourg	Both	175.69(155.56-196.78)	352.27(316.19-388.61)	24.36(15.66-34.7)	5168.72(4701.12-5615.73)	9650.48(8832.94-10495.3)	14.98(10.46-19.6)	11.19(8.06-15.48)	24.85(17.08-36.24)	105.05(69.95-148.75)	228.4(175.33-293.42)	423.09(324.22-561.46)	56.07(38.43-77.8)
Luxembourg	Female	108.6(94.54-123.38)	186.27(164.66-208.14)	8.41(-1.83-20.29)	2917.91(2646.07-3187.2)	4979.07(4537.44-5450.82)	7.94(2.58-13.65)	6.93(4.98-9.79)	15.03(9.98-22.16)	93.28(55.01-140.41)	134.28(103.46-171.85)	235.12(174.45-312.6)	40.06(21.71-62.69)
Luxembourg	Male	67.09(58.78-76.83)	166(146.43-186.5)	50.33(35.31-68.48)	2250.82(2010.72-2468.17)	4671.41(4238.33-5093.17)	24.22(18.02-30.33)	4.26(2.96-6.05)	9.82(6.8-14.3)	120.45(78.17-169.58)	94.12(70.47-123.05)	187.98(142.13-248.59)	76.85(51.93-106.43)
Madagascar	Both	534.14(472.02-609.93)	1396.52(1225.98-1579.57)	23.4(15.56-31.16)	66408.09(58042.09-76411.64)	155206.24(136115.01-177710.29)	6.98(2.39-11.71)	244.37(168.21-337.67)	431.07(295.43-607.49)	61.24(28.05-99.74)	5978.78(4265.3-8043.28)	11671.73(8312.02-16532.53)	94.2(53.69-141.77)
Madagascar	Female	225.64(196.5-259.25)	645.05(557.8-743.32)	36.01(26.08-46.61)	32312.1(27830.61-37598.95)	75824.05(65491.61-87640.82)	6.95(0.64-13.44)	96.27(65.27-146.9)	204.58(132.8-317.47)	87.55(45.59-137.54)	2539.84(1734.13-3765.27)	5644.17(3811.61-8465.83)	116.29(67.94-179.9)
Madagascar	Male	308.5(270.81-352.27)	751.46(655.83-866.25)	14.06(4.39-24.46)	34095.99(29597.68-39535.15)	79382.19(68715.98-92230.67)	7.03(2.05-12.58)	148.1(96.08-209)	226.49(149.39-323.75)	44.12(8.03-87.63)	3438.94(2297.16-4727.03)	6027.57(4128.74-8453.59)	77.83(34-128.85)
Malawi	Both	479.15(420.17-542.85)	1125.17(994.15-1267.13)	34.08(24.91-44.41)	88258.39(64204.45-121660.39)	176591.3(130431.9-244418.59)	5.83(0.48-11.89)	215.61(159.65-276.26)	417.02(307.4-543.16)	165.4(114.02-222.42)	5348.21(3968.66-6786.04)	9947.73(7374.88-12973.54)	193.24(135.09-258.85)
Malawi	Female	223.35(191.91-256.71)	550.82(477.38-631.28)	41.86(29.82-54.35)	44353.8(32421.83-60607.5)	88395.08(65598.71-121356.49)	3.89(-2.66-11.9)	101.29(71.14-134.13)	203.49(149.1-267.68)	194.47(120.05-291.01)	2561.62(1799.33-3386.06)	4566.63(3386.34-5980.92)	193.32(123.39-285.67)
Malawi	Male	255.79(224.18-292.87)	574.35(505.63-648.64)	27.19(15.59-39.97)	43904.6(31934.99-60157.32)	88196.22(64102.28-125491.6)	7.92(0.64-15.03)	114.33(80.52-153.94)	213.52(150.85-288.11)	142.16(83.56-216.21)	2786.59(1995.54-3644.52)	5381.1(3831.83-7108.84)	193.05(125.27-275.92)
Malaysia	Both	2152.54(1895.83-2433.15)	9844.04(8814.72-11076.38)	158.81(140.26-178.64)	228758.79(199687.68-263488.14)	607283.47(545079.46-675780.92)	52.86(44.67-62.43)	815.31(662.72-961.53)	2444.04(1841.86-3138.35)	40.3(20.13-59.31)	22240.35(18066.31-26356.54)	63695.2(49720.43-80374.42)	64.16(42.07-86.98)
Malaysia	Female	1182.77(1028.45-1363.82)	5469.31(4814.34-6223.53)	170.62(142.43-200.05)	114240.91(98139.9-132724.78)	293581.39(262624.87-328324.89)	51.37(41.23-63.38)	379.72(304.15-459.59)	1280.12(963.93-1678.87)	63.25(34.53-94.98)	10458.89(8464.64-12609.61)	32785.52(25460.26-41792.86)	86.31(56.79-121.63)
Malaysia	Male	969.76(849.72-1101.88)	4374.73(3842.47-5015.42)	147.38(123.13-170.11)	114517.88(98680.27-132636.45)	313702.08(278554.82-355491.37)	54.26(45.97-63.55)	435.59(349.67-527)	1163.92(848.29-1528.11)	21.98(-1.75-49.62)	11781.46(9358.27-14277.45)	30909.67(23281.53-40057.94)	46.12(19.7-75.31)
Maldives	Both	24.24(20.56-28.07)	123.56(108.29-140.16)	145.48(121.14-175.23)	2392.65(2091.82-2759.65)	9462.13(8324.49-10897.38)	78.34(69.51-87.78)	15.87(12.59-20.11)	33.06(25.73-41.7)	99.53(55.02-144.76)	443(344.53-562.38)	784.17(622.88-974.04)	93.33(48.95-141.27)
Maldives	Female	10.83(9.05-12.66)	58.56(51.16-66.18)	220.33(185.33-264.81)	1069.66(922.07-1260.42)	3611.04(3216.13-4114.36)	86.17(73.21-100.28)	7.81(5.54-11.06)	16.51(12.66-21.44)	129.12(52.26-226.48)	232.1(164.12-330.35)	381.33(299.33-478.77)	111.64(40.35-199.54)
Maldives	Male	13.41(11.34-15.62)	65(56.23-75.02)	98.71(76.01-125.96)	1322.99(1151.52-1518.27)	5851.08(5066.12-6874.39)	69.6(59.98-80.53)	8.06(6.31-9.86)	16.55(12.75-20.91)	79.85(43.67-127.46)	210.9(164.5-259.12)	402.84(313.98-504.96)	83.26(43.4-133.47)
Mali	Both	648.15(529.43-809.52)	1784.83(1459.28-2246.78)	18.24(10.01-26.71)	50237.05(44301.91-57235.51)	118586.07(105195.19-134564.29)	-5.01(-7.99--1.7)	317.07(221.77-427.26)	589.46(418.87-788.79)	49.13(22.4-83.53)	7902.38(5553.05-10719.65)	14402.1(10209.38-19253.71)	47.96(22.43-79.72)
Mali	Female	343.59(273.16-431.61)	926.92(745.24-1175.04)	18.57(8.4-29.84)	26102.13(22919.42-30007.69)	58562.94(51797.65-66524.54)	-9.15(-12.84--5.22)	159.46(101.63-240.83)	272.17(185.87-376.85)	43.95(9.86-90.89)	4146.18(2664.61-6155.27)	6951.32(4648.47-9671.94)	40.88(8.63-83.32)
Mali	Male	304.56(251.54-384.83)	857.92(698.63-1077.44)	18.03(7.13-29.18)	24134.92(21165.49-27341.31)	60023.13(52772.85-68512.88)	-0.56(-4.45-3.46)	157.61(108.78-217.37)	317.29(210.73-440.68)	54.27(18.63-99.1)	3756.2(2622.22-5120.05)	7450.78(4948.89-10565.55)	56.02(21.28-99.48)
Malta	Both	147.46(129.61-166.06)	350.09(305.72-391.3)	106.29(90.91-124.58)	4387.63(4010.53-4797.77)	8327.54(7637.03-8986.92)	56.8(49.04-64.71)	11.67(8.47-16.03)	22.44(15.8-31.5)	37.45(16.69-62.26)	238.75(181.6-308.16)	423.93(320.26-549.87)	42.91(26.64-60.99)
Malta	Female	82.53(71.69-93.7)	183.12(159.71-208.15)	94.42(75.38-117.22)	2304.91(2080.69-2541.69)	4277.44(3907.1-4655.17)	56.23(47.08-66)	6.27(4.48-8.62)	12.35(8.6-17.8)	37.93(14.5-66.74)	126.03(97.5-161)	222.88(169.07-291.9)	42.84(24.74-64.74)
Malta	Male	64.93(55.81-74.28)	166.96(141.33-190.71)	121.44(99.72-150.21)	2082.72(1875.08-2309.07)	4050.1(3688.09-4415.25)	57.46(48.05-67.96)	5.4(3.81-7.65)	10.09(7.07-14.16)	36.08(12.71-64.12)	112.71(82.97-149.35)	201.04(148.6-264.61)	43.05(22.28-64.81)
Marshall Islands	Both	3.66(3.17-4.3)	10.88(9.39-12.58)	134.68(118.42-151.46)	457.21(388.08-539.31)	887.44(779.62-1009.66)	55.7(46.73-66.59)	2.01(1.49-2.73)	5.34(3.65-7.54)	85.08(55.65-115.09)	60.13(45-79.56)	171.66(117.05-244.54)	122.35(86.6-158.11)
Marshall Islands	Female	1.61(1.35-1.92)	4.67(3.91-5.48)	132.86(112.27-153.2)	232.51(195.28-275.6)	439.04(380.88-501.79)	51.32(39.95-63.9)	0.94(0.66-1.34)	2.67(1.73-4.17)	85.57(46.88-130.72)	28.42(20.37-39.83)	87.9(57.69-134.76)	126.01(79.77-173.64)
Marshall Islands	Male	2.06(1.77-2.41)	6.2(5.35-7.15)	135.57(116.93-156.85)	224.7(189.28-263.73)	448.4(390.91-514.94)	60.22(50.84-70.61)	1.07(0.78-1.52)	2.67(1.83-3.75)	81.86(45.82-122.32)	31.71(23.45-44.13)	83.76(58.05-117.61)	116(74.49-160.72)
Mauritania	Both	185.49(163.5-211.32)	519.57(453.43-588.47)	38.85(25.27-48.73)	12679.21(11384.44-14184.57)	28312.07(25477.18-31307.88)	15.99(11.92-20.02)	111.61(80.62-146.33)	154.14(107.11-207.85)	50.61(17.09-86.01)	2589.38(1861.98-3417.9)	3415.98(2339.8-4623.04)	57.35(22.28-94.93)
Mauritania	Female	106.38(92.38-121.88)	288.27(249.37-330.26)	35.21(20.31-47.61)	6760.16(6021.3-7619.54)	14733.25(13192.94-16404.54)	12(6.93-17.15)	63.58(43.3-86.5)	85.2(54.42-120.48)	47.18(11.01-92.7)	1485.6(1010.45-2040.58)	1951.47(1291.36-2766.15)	52.34(17.41-95.64)
Mauritania	Male	79.12(69.12-90.15)	231.3(201.18-264.63)	43.93(28.15-58.12)	5919.05(5225.48-6728.41)	13578.83(12167.85-15116.67)	20.5(15.11-26.21)	48.02(33.66-63.57)	68.94(45.94-98.27)	55.45(13.85-102.74)	1103.79(763.59-1478.73)	1464.51(948.2-2099.89)	62.54(18.55-115.41)

Mauritius	Both	259.37(223.08-299.33)	947.53(816.51-1072.82)	219.15(179.54-262.27)	20140.62(17671.05-23036.66)	43577.32(39847.11-47836.77)	85.96(70.61-100.72)	120.92(100.94-142.44)	530.27(417.07-674.09)	180.92(151.27-214.15)	3273.68(2683.3-3855.83)	13103.59(10203.67-16731.07)	209.42(172.71-250.9)
Mauritius	Female	138.71(120.07-159.17)	488.78(430.74-557.5)	207.25(171.9-249.21)	10246.16(8917.2-11796.74)	22078.04(20103.78-24405.48)	83.22(67.65-98.37)	59.6(49.18-70.69)	252.15(196.45-318.92)	148.9(114.87-188.47)	1569.94(1286.03-1853.03)	5996.87(4682.61-7655.46)	182.06(144.6-227.34)
Mauritius	Male	120.66(100.47-141.5)	458.75(351.63-543.23)	232.75(156.98-306.35)	9894.47(8616.44-11300.28)	21499.29(19143.13-23990.89)	88.84(70.01-106.9)	61.32(50.31-72.26)	278.12(214.75-353.24)	210.5(175.28-251.89)	1703.74(1371.43-2014.08)	7106.72(5429.58-9167.72)	234.52(192.59-285.58)
Mexico	Both	18223.53(16229.44-20697.23)	73563.27(67530.56-79960.3)	205.88(183.73-229.62)	1085083.31(985111.67-1201855.4)	2887167.01(2673928.4-3102187.31)	83.72(74.11-93.81)	2965.12(2435.36-3506.35)	19069.72(15027.03-23517.28)	283.56(260.71-309.1)	80035.81(65579.5-94198.87)	487385.36(383517.33-604960.7)	391.78(341.46-444.55)
Mexico	Female	10219.83(9079.2-11541.03)	39166.39(35875.34-42419.61)	185.01(161.06-208.58)	566622.15(511760.98-630216.83)	1486151.46(1377741.94-1596634.28)	79.26(68.37-90.37)	1582.05(1296.38-1865.13)	9474.77(7133.42-12125.14)	250.52(220.96-279.25)	42895.67(35178.78-50577.4)	239386.5(183214.32-304460.51)	342.48(287.08-397.7)
Mexico	Male	8003.7(7118.93-9074.91)	34396.88(31276.67-37686.28)	232.15(209.79-255.76)	518461.16(470904.24-571477.77)	1401015.55(1291260.58-1514359.53)	88.64(79.77-97.74)	1383.07(1137.82-1640.19)	9594.95(7087.2-12425.08)	319.4(287.83-357.64)	37140.14(30484.46-43657.04)	247998.86(185882.22-317154.33)	446.78(388.56-515.53)
Micronesia (Federated States of)	Both	11.78(10.09-13.62)	27.41(23.49-31.66)	132.17(115.76-150.88)	1198.57(1043.7-1392.16)	1767.75(1573.29-1991.34)	52.19(43.5-61.47)	7.07(5.15-9.41)	16.57(11.47-22.92)	108.65(53.65-168.09)	212.15(155.93-283.83)	502.29(345.07-702.27)	154.91(83.58-228.66)
Micronesia (Federated States of)	Female	5.23(4.36-6.16)	12.53(10.55-14.62)	140(120.82-164.58)	610.38(525.81-721.92)	904.8(802.73-1020.29)	50.71(40.12-61.86)	3.76(2.62-5.28)	8.4(5.85-11.8)	92.38(36.26-170.33)	111.64(78.62-153.89)	249.7(170.3-352.21)	138.21(67.12-236.34)
Micronesia (Federated States of)	Male	6.55(5.62-7.55)	14.88(12.7-17.37)	125.84(106.6-148.38)	588.19(506.72-683.73)	862.95(761.94-976.38)	53.59(44.37-63.16)	3.31(2.42-4.48)	8.17(4.87-12.07)	125.22(42.19-204.09)	100.51(73.04-135.94)	252.59(149.75-372.12)	172.81(74.35-268.69)
Monaco	Both	19.66(17.28-22.2)	29.3(25.78-33.18)	20.99(12.29-31.08)	537.57(492.85-584.01)	738.71(677.11-798.08)	11.67(7.74-15.82)	0.72(0.48-1.07)	1.58(1.06-2.22)	83.87(49.85-121.81)	16.44(12.32-21.39)	28.28(21-36.84)	45.68(27.64-64.52)
Monaco	Female	11.33(9.87-12.93)	15.77(13.74-17.81)	15.7(4.9-27.25)	293.87(267-322.28)	380.98(347.87-414.06)	7.85(2.3-13.54)	0.37(0.23-0.57)	0.84(0.55-1.24)	93.17(50.85-149.24)	8.57(6.3-11.17)	14.66(10.71-19.29)	45.29(23.18-71.03)
Monaco	Male	8.33(7.22-9.48)	13.53(11.75-15.68)	28.36(16.4-42.31)	243.7(220.72-266.01)	357.73(323.23-391.37)	16.21(10.93-21.82)	0.35(0.23-0.52)	0.74(0.49-1.07)	74.51(35.06-124.63)	7.87(5.8-10.53)	13.62(10.01-18.12)	46.15(22-73.71)
Mongolia	Both	180.79(154.4-212.5)	534.35(461.24-627.23)	90.25(74.13-109.26)	19900.75(17431.26-22788.24)	45976.55(41000.06-51591.98)	48.54(38.95-57.79)	87.86(63.25-114.59)	98.2(67.9-135.64)	-7.21(-20.84-8.45)	2430.71(1771.72-3186.23)	3220.09(2252.66-4406.7)	35.43(13.01-61.73)
Mongolia	Female	93.49(78.79-111.06)	262.94(223.95-309.3)	78.49(62.08-98.37)	9996.32(8705.53-11468.79)	22997.24(20277.31-25909.94)	45.38(35.1-55.72)	47.41(33.7-63.51)	45.88(31.78-63.91)	-8.48(-23.83-10.6)	1323.72(954.85-1792.94)	1476.57(1042.16-2036.27)	26.64(4-57.62)
Mongolia	Male	87.31(74.58-102.6)	271.42(231.21-321.15)	103.05(81.09-127.83)	9904.43(8657.86-11522.08)	22979.31(20272.28-26089.87)	51.88(42.08-61.53)	40.45(28.08-54.64)	52.32(35.02-73.3)	-2.31(-21.81-21.31)	1106.99(778.1-1485.75)	1743.52(1191.74-2460.74)	48.89(19.06-87.23)
Montenegro	Both	156.86(135.67-178.86)	356.89(313.98-405.59)	141.67(122.34-161.76)	8797.45(7898.02-9718.65)	13219.63(12118-14406.93)	49.98(42.12-57.91)	16.37(11.97-21.54)	31.33(22.11-42.4)	21.25(-0.37-43.11)	418.83(317.95-523.84)	767.21(561.38-997.02)	49.87(27.65-73.37)
Montenegro	Female	76.69(65.78-88.56)	171.47(146.73-196.67)	132.72(107.76-160.31)	4530.06(4035.15-5055.88)	6805.79(6200.32-7456.98)	48.86(39.76-58.04)	7.54(5.38-10.04)	14.87(10.62-20.12)	21.69(-5.38-48.51)	190.73(142.67-244.34)	355.56(265.39-457.38)	48.49(22.27-75.81)
Montenegro	Male	80.17(68.66-92.45)	185.42(160.76-214.38)	151(126.69-176.29)	4267.39(3822.04-4755.93)	6413.83(5860.96-7009.08)	51.17(42.51-60.38)	8.83(6.31-11.86)	16.46(11.16-23.14)	20.73(-7.19-53.62)	228.09(168.07-293.87)	411.66(296.45-550.13)	50.98(21.94-84.27)
Morocco	Both	3862.02(3365.89-4458.75)	19008.13(16750.11-21608.06)	252.42(231.58-273.23)	207349.93(185397.34-234404.04)	557989.83(508945.89-607409.95)	89.32(78.14-101.44)	987.63(702.44-1392.34)	2848.04(1999.11-3862.21)	124.81(76.89-176.91)	23987.09(17783.99-31803.17)	69257.06(50004.1-90704.29)	220.52(154.23-294.7)
Morocco	Female	2076.94(1785.89-2424.88)	9035.21(7913.96-10385.65)	213.81(190.13-237.5)	111752.4(99173.63-126576.27)	287013.99(260512.45-316002.64)	81.9(70.29-95.06)	476.48(329.7-715.87)	1465.99(1016.22-2056.73)	143.93(70.41-219.36)	12006.14(8671.45-16768.03)	36429.13(26073.99-49590.52)	230.4(134.64-334.3)
Morocco	Male	1785.08(1553.38-2064.04)	9972.92(8668.79-11438.35)	297.01(266.03-331.85)	95597.52(84410.16-108817.62)	270975.84(244802.27-297028.48)	98.04(84.57-112.25)	511.15(349.92-761.75)	1382.05(896.13-2052.62)	107.84(61.53-172.09)	11980.95(8460.08-16711.39)	32827.93(21939.38-46255.9)	209.96(140.73-300.33)
Mozambique	Both	641.51(569.6-719.43)	1584.87(1391.43-1793.09)	18.42(12.22-25.62)	78702.2(68924.29-90036.22)	161825.78(141822.59-184535.62)	-7.15(-10.15--3.87)	272.12(186.75-373.2)	582.23(408.72-791.03)	70.41(21.28-117.65)	6714.52(4698.43-9054.74)	14664.56(10436.92-19987.6)	97.78(42.67-156.48)
Mozambique	Female	286.86(252.35-326.38)	777.73(673-895.15)	32.42(22.29-43.02)	40716.14(35249.58-47411.31)	83210.4(72275.02-95263.85)	-7.55(-11.93--3.06)	121.29(84.15-166.76)	277.3(190.29-389.31)	89.1(39.05-156.47)	3012.86(2138.84-3994.52)	6553.23(4557.4-9075.38)	101.63(47.72-172.96)
Mozambique	Male	354.65(308.83-405.37)	807.14(694.2-924.9)	6.86(-1.04-15.63)	37986.06(33090.58-43240.6)	78615.38(67987.63-90224.17)	-6.72(-10.81--2.13)	150.83(95.94-226.38)	304.93(209.4-419.58)	56.07(-3.47-123.53)	3701.67(2451.27-5500.86)	8111.32(5597.08-11120.72)	94.47(24.96-173.95)
Myanmar	Both	4480.57(3944.56-5085.72)	13211.79(11584.45-14972.94)	140.06(125.74-155.95)	548546.13(480038.67-627881.68)	1032654.3(926683.82-1149110.26)	43.18(35.42-50.88)	2280.86(1631.85-2992.05)	4050.75(3219.17-5221.21)	118.31(79.31-169.96)	71720.65(50934.67-94921.52)	115695.98(88670.8-151033.44)	166.52(117.77-230.81)
Myanmar	Female	2565.25(2226.1-2960.52)	7903.91(6842.49-9047.69)	145.76(128.34-165.95)	281023.54(242767.95-323695.21)	549642.6(490206.31-616380.91)	43.89(33.85-53.54)	1208.19(850.37-1655.14)	2160.31(1688.2-2810.76)	112.41(67.97-170.81)	39330.44(27279.72-54288.87)	61541.37(47176.74-80807.46)	151.26(96.59-222.63)
Myanmar	Male	1915.32(1654.59-2182.78)	5307.88(4568.36-6051.65)	130.33(109.73-153.36)	267522.59(232960.89-307731.35)	483011.69(428704.69-548515.3)	42.26(34.78-50.27)	1072.67(730.67-1494.26)	1890.44(1466.77-2418.92)	122.75(67.86-199.75)	32390.21(22107.68-45460.87)	54154.61(40522.67-70699.07)	182.94(114.55-275.57)
Namibia	Both	144.28(125.86-163.54)	375.95(329.95-428.55)	62.6(49.98-80.96)	10034.8(8815.95-11368.77)	20949.15(18735.8-23483.06)	24.49(19.75-29.8)	37.87(24.12-57.44)	75.24(49.62-113.16)	28.39(-1.26-62.81)	935.45(606.3-1403.08)	1812.78(1203.92-2631.88)	40.38(9.42-76.94)
Namibia	Female	59.82(51.64-69.06)	171.69(149.3-196.95)	78.55(61.22-99.6)	5080.76(4443.22-5821.77)	10798.2(9642.16-12126.36)	24.9(18.41-31.36)	13.06(8.01-22.27)	30.23(17.72-56.84)	45.01(7.14-91.45)	323.97(208.03-532.34)	684.35(422.7-1183.63)	52.1(15.78-97.27)
Namibia	Male	84.46(73.14-96.47)	204.26(178.1-233.35)	51.39(37.45-69.62)	4954.04(4339.52-5629.68)	10150.94(8993.64-11521.95)	24.13(18.66-29.68)	24.81(15.02-38.73)	45(28.66-67.98)	20.13(-17.42-63.2)	611.48(381.27-950.33)	1128.43(728.49-1639.93)	34.39(-4.86-79.28)
Nauru	Both	0.97(0.82-1.15)	1.49(1.24-1.78)	42.37(33.49-51.74)	124.2(105.42-146.76)	141.29(122.27-163.42)	10.25(5.25-15.78)	0.54(0.38-0.73)	0.78(0.52-1.08)	56.29(25.68-86.52)	18.47(12.93-24.87)	28.22(18.57-38.87)	67.93(37.47-100.07)
Nauru	Female	0.4(0.33-0.49)	0.7(0.57-0.84)	61.32(47.82-74.65)	61.34(51.55-74.13)	73.51(63.6-85.61)	13.81(6.75-21.42)	0.26(0.18-0.37)	0.37(0.23-0.58)	47.77(8.24-92.78)	8.72(5.93-12.26)	13.4(8.49-20.34)	63.11(23.26-112.39)
Nauru	Male	0.57(0.48-0.67)	0.79(0.66-0.93)	29.17(18.57-40.09)	62.86(53.3-73.65)	67.78(57.79-79.23)	6.62(1.53-11.6)	0.29(0.18-0.41)	0.41(0.22-0.63)	61.68(17.1-98.65)	9.76(5.98-14.14)	14.81(8.09-22.85)	70.97(26.25-113.44)
Nepal	Both	1556.89(1348.21-1834.96)	6168.27(5411.17-7192.81)	181.38(165.01-200.26)	139904.26(120880.46-161193.33)	332579.88(299485.38-371910.5)	56.13(48.2-64.84)	366.53(228.15-528.06)	1403.25(904.09-1991.48)	339.62(240.51-454.32)	10716.5(7040.48-15054.17)	36904.04(24906.6-50799.71)	474.41(342.36-622.54)
Nepal	Female	664.89(561.41-780.83)	2922.38(2522.16-3412.11)	202.33(178.07-230.52)	62874.98(53548.2-73281.58)	160401.28(143239.07-179507.08)	58.33(48.05-69.23)	167.05(94.87-243.23)	710.09(401.78-1007.05)	411.8(279.93-583.08)	4979.9(2990.31-7231.53)	18601.19(11269.08-26151.34)	521.98(363.28-722.3)

Nepal	Male	892(764.56-1054.26)	3245.88(2814.7-3767.77)	167.74(147.4-190.96)	77029.27(66600.06-89035.79)	172178.6(153415.39-192704.9)	55.88(48.12-65.08)	199.48(118.26-320.95)	693.16(420.91-1127.26)	282.88(179.7-409.51)	5736.6(3631.28-8731.89)	18302.85(11746.84-27902.69)	433.11(294.73-602.95)
Netherlands	Both	5943.59(5415.65-6551.12)	11258.07(9989.58-12535.09)	65.26(50.85-81.16)	196645.57(180796.45-213891.62)	307403.63(281790.55-335283.21)	34.56(27.43-41.28)	284.23(205.02-402.06)	639.79(441.05-903.5)	82.13(56.71-111.58)	5805.1(4399.62-7488.76)	11057.1(8444.31-14448.51)	68.02(51.74-86.9)
Netherlands	Female	3430.58(3067.55-3854.56)	5754.43(5042.95-6555.85)	47.45(30.33-67.23)	102876.84(93624.32-112593.2)	151829.26(137936.23-167033.64)	28.23(20.61-36.32)	177.47(123.47-256.44)	378.01(255.58-546.5)	63.91(36.66-99.46)	3350.2(2553.19-4344.86)	5921.18(4454.09-7710.56)	48.58(31.45-70.57)
Netherlands	Male	2513(2208.02-2807.98)	5503.64(4759.89-6293.89)	89.5(64.03-117.9)	93768.72(85244.12-103395.9)	155574.37(141839.74-170593.76)	41.38(31.88-50.72)	106.76(76.59-151.66)	261.77(184.11-375.96)	108.33(73.84-147.2)	2454.91(1808.34-3184)	5135.92(3832.75-6774.98)	93.58(69.82-122.62)
New Zealand	Both	1339.51(1187.84-1504.75)	2969.04(2664.69-3344.01)	71.68(58.33-89.2)	35945.72(32850.95-39413.73)	73474.62(67465.57-79881.4)	56.1(48.42-64.54)	35.85(25-50.52)	120.21(85.77-167.7)	155.26(118.95-197.79)	1015.33(769.35-1318.5)	2681.56(2028.01-3442.87)	126.24(101.82-152.79)
New Zealand	Female	735.09(652.13-836.17)	1507.52(1318.23-1697.14)	57.6(39.11-74.58)	20312.82(18383.1-22341.59)	39454.82(35990.78-43066.69)	45.41(36.96-54.83)	20.3(14.48-28.22)	59.12(41.44-82.74)	114.96(80.94-157.26)	564.53(434.67-719.37)	1309.85(1003.33-1670.4)	88.93(65.62-114.1)
New Zealand	Male	604.42(517.15-704.88)	1461.52(1270.99-1680.78)	88.91(66.16-121.93)	15632.91(14149.5-17335.92)	34019.8(30906.48-37336.21)	69.75(58.54-82.93)	15.54(10.39-22.44)	61.09(43.33-83.71)	207.71(160.52-265.78)	450.8(334.88-600.83)	1371.7(1036.1-1785.31)	173.45(136.75-212.05)
Nicaragua	Both	573.17(493.6-662.72)	2598.41(2257.06-2940.68)	205.37(183.88-227.6)	37943.81(33451.79-43127.52)	114334.08(103355.13-126083.54)	83.41(73.15-94.92)	146.33(114.62-181.19)	991.49(736.1-1271.54)	329.81(278.15-379.85)	4014.58(3135.02-4913.39)	24805.22(18306.5-31940.25)	512.01(424.19-606.88)
Nicaragua	Female	220.89(192.06-256.86)	1043.99(918.68-1194.63)	218.61(191.34-250.43)	17993.65(15497.84-20922.34)	50573.49(45071.87-56369.24)	70.27(58.2-84.69)	49.18(37.75-61.98)	380.03(290.03-483.48)	375.03(296.58-453.59)	1370.25(1068.73-1715.1)	8915.05(6700.43-11414.13)	530.08(424.86-652.77)
Nicaragua	Male	352.28(297.21-411.75)	1554.42(1320.28-1793.16)	196.99(171.16-225.36)	19950.16(17588.65-22481.37)	63760.59(57376.16-70379.56)	95.53(84.38-107.13)	97.15(75.52-122.14)	611.47(440.78-796.19)	308.78(252.56-367.53)	2644.34(2060.67-3297.86)	15890.17(11580.84-20744.69)	507(412.89-605.74)
Niger	Both	402.82(354.54-457.22)	1528.04(1338.54-1756.93)	33.86(24.7-44.01)	36258.77(32050.86-41351.81)	110111.45(97815.11-124420.87)	5.85(1.7-10.51)	192.05(132.46-263.77)	455.86(318.65-628.81)	90.7(52.34-139.41)	4965.16(3431.23-6838.87)	11680.7(8126.63-16080.92)	97.2(58.92-145.38)
Niger	Female	191.64(165.69-219.76)	767.28(661.57-879.38)	42.51(30.84-55.39)	17956.21(15596.16-20858.09)	56220.14(49547.04-63688.62)	8.81(3.44-14.91)	80.62(53.32-123.9)	195.37(132.31-272.23)	98.75(47.42-172.56)	2163.98(1427.68-3262.9)	5250.18(3597.64-7276.52)	106.94(57.83-177.64)
Niger	Male	211.18(184.27-240.76)	760.76(663-875.8)	25.97(15.7-37.8)	18302.56(16112.59-20925.75)	53891.31(47581.02-61685.8)	2.94(-2.05-8.62)	111.44(73.28-164.91)	260.5(172.73-386.18)	84.37(39.64-150.19)	2801.18(1847.47-4108.82)	6430.52(4239.69-9482.41)	89.36(46.68-153.94)
Nigeria	Both	7225.1(6471.98-8015.68)	18458.11(16514.94-20531.2)	15.29(11.63-19.59)	543693.22(497268.16-594826.08)	1232801.46(1124206.7-1346377.76)	-3.74(-6.65--0.9)	2359.35(1623.18-3261.42)	4113.74(2910.38-5495.72)	46.61(14.91-84.15)	53923.28(37424.95-74687.99)	97878.17(70338.95-131347.72)	54.14(16.37-98.39)
Nigeria	Female	3875.79(3450.66-4321.6)	10380.53(9247.3-11567.88)	17.92(12.12-24.08)	257943.18(236701.99-280917.57)	644552.87(589733.74-702571.94)	1.75(-2.72-5.84)	1164.22(778.1-1672.88)	1883.92(1243.38-2605.12)	34.11(-3.63-76.03)	25694.03(17614.89-36846.16)	47017.7(31452.4-66996.45)	49.93(5.28-106.22)
Nigeria	Male	3349.31(2998.65-3713.31)	8077.57(7207.68-8958.44)	11.79(7.6-16.19)	285750.05(259920.97-314052.17)	588248.59(535082.34-645166.22)	-8.72(-10.96--6.14)	1195.14(753.51-1780.2)	2229.82(1464.46-3325.59)	58.66(15.89-114.36)	28229.25(17822.1-41872.91)	50860.47(33275.27-76748.11)	57.81(8.04-131.11)
Niue	Both	0.52(0.46-0.59)	0.81(0.71-0.93)	97.13(85.7-109.83)	43.51(39.09-48.93)	45.55(41.2-50.09)	44.37(38.08-50.9)	0.29(0.22-0.37)	0.36(0.26-0.46)	62.32(33.44-85.74)	6.52(5.01-8.36)	8.64(6.28-11.19)	73.01(47.26-96.5)
Niue	Female	0.25(0.21-0.28)	0.37(0.32-0.43)	95.13(80.33-112.8)	23.79(21.17-26.97)	23.87(21.57-26.38)	38.27(31.5-45.83)	0.16(0.12-0.22)	0.17(0.11-0.25)	52.07(15.98-85.6)	3.44(2.52-4.64)	4.09(2.78-5.71)	56.37(28.13-87.77)
Niue	Male	0.28(0.24-0.32)	0.44(0.38-0.51)	98.54(83.75-116.48)	19.72(17.66-22.14)	21.68(19.53-23.97)	51.73(44.39-59.86)	0.13(0.09-0.17)	0.19(0.12-0.25)	75.7(38.17-112.97)	3.08(2.14-4.12)	4.55(3.04-6)	91.68(54.35-129.39)
North Macedonia	Both	375.93(329.57-423.96)	1194.58(1062.69-1348.05)	215.55(186.22-244.74)	25531.67(22879.73-28623.53)	44741.89(41020.17-48749.31)	62.59(53.3-71.94)	35.47(24.31-47.94)	77.33(50.6-109.75)	50.84(26.42-78.57)	1070.33(781.75-1379.26)	2230.93(1630.99-2962.2)	85.89(60.8-113.51)
North Macedonia	Female	174.82(150.38-204.7)	545.07(475.18-622.65)	208.96(167.56-252.06)	12718.43(11220.53-14373.43)	22079.82(20050.05-24282.14)	62.29(51.16-74.22)	15.85(10.81-21.27)	37.64(24.95-53.68)	61.18(28.41-102.74)	492.24(354.75-636.5)	1067.57(771.11-1418.6)	93.49(60.72-132.24)
North Macedonia	Male	201.1(174.71-230.84)	649.51(566.22-750.77)	221.42(179.6-264.09)	12813.25(11465-14360.25)	22662.08(20596.94-24795.92)	62.86(52.1-73.56)	19.62(13.29-27.21)	39.68(25.08-58.25)	42.31(13.83-73.18)	578.09(419.52-776.24)	1163.37(829.68-1574.68)	79.43(49.8-112.17)
Northern Mariana Islands	Both	6.44(5.43-7.65)	23.91(20.27-28.2)	250.04(202.33-306.35)	707.31(590.56-839.87)	1184.15(1065.02-1313.36)	75.5(53.36-99.58)	2.2(1.66-2.89)	8.58(6.58-10.85)	105.14(71.74-141.12)	76.04(55.87-100.81)	246.08(186.89-308.3)	148.97(105.06-196.84)
Northern Mariana Islands	Female	2.33(1.72-2.98)	9.28(7.59-11.38)	282.54(201.31-400.41)	316.19(258.38-380.73)	563.79(500.19-632.64)	84.06(57.62-114.54)	1.05(0.76-1.4)	4.09(3.05-5.44)	94.18(56.82-137.42)	36.04(25.93-48.57)	117.85(87.97-155.47)	146.29(93.2-209.55)
Northern Mariana Islands	Male	4.11(3.46-4.88)	14.63(12.44-17.24)	230.12(190.79-276.93)	391.12(329.66-465.88)	620.37(551.39-689.07)	68.71(48.53-90.32)	1.14(0.85-1.51)	4.49(3.44-5.66)	112.26(70.25-165.79)	40.01(28.93-53.86)	128.23(97.43-161.75)	150.19(99.47-208.61)
Norway	Both	1551.28(1342.47-1756.96)	2620.42(2332.29-2926.96)	35.29(26.43-44.75)	49990.09(46227.32-53659.59)	75263.59(69735.42-80659.63)	18.9(15.21-22.44)	58.5(42.04-82.23)	144.82(101.59-198.51)	170.26(131.91-218.76)	1515.98(1158.5-1962.49)	2515.55(1944.01-3195.73)	69.63(52.68-89.17)
Norway	Female	832.31(724.14-950.55)	1299.86(1160.84-1450.87)	26.91(18.87-36.03)	24699.97(22799.76-26566.09)	35377.37(32846.75-37939.02)	15.82(11.78-19.57)	29.07(20.42-40.25)	76.29(52.54-105.2)	170.48(122.32-229.93)	755.27(581.21-976)	1248.42(966.79-1588.71)	59.52(40.9-81.19)
Norway	Male	718.97(621.18-817.85)	1320.56(1170.67-1485.07)	44.86(33.86-56.75)	25290.12(23372.77-27225.35)	39886.22(36916.63-42870.56)	21.51(17.78-24.95)	29.43(20.55-41.55)	68.53(48.23-94.79)	168.93(129.38-218.4)	760.71(565.18-988.53)	1267.13(961.85-1634.59)	79.92(60.88-101.57)
Oman	Both	204.01(178.05-232.58)	1113.34(974.02-1270.01)	131.5(113.78-149.88)	13023.05(11363.25-14935.95)	46042.69(40788.88-52270.12)	51.3(44.26-58.96)	26.19(18.43-36.55)	66.29(50.67-82.33)	100.42(53.16-156.2)	724.07(535.62-967.74)	2057.55(1657.63-2497.33)	107.26(66.44-154.03)
Oman	Female	97.45(84.5-112.55)	439.64(380.33-504.11)	120.45(101.94-141.24)	5466.68(4771.25-6263.23)	17004.53(15098.68-19150.85)	55.41(46.64-65.48)	12.68(8.85-17.55)	31.77(23.3-41.1)	114.27(55.06-182.52)	342.78(252.45-454.93)	937.11(730.3-1152.39)	120.58(71.66-177.93)
Oman	Male	106.56(91.22-123.37)	673.7(577.9-776.22)	143.4(119.18-168.52)	7556.37(6508.51-8816.18)	29038.16(25253.67-33460.43)	49(40.88-57.73)	13.51(9.07-20.49)	34.52(25.6-45.35)	93.03(33.04-168.74)	381.29(268.02-550.19)	1120.44(858.94-1438.47)	101.31(53.3-158.61)
Pakistan	Both	10526.88(9332.12-11939.68)	26899.35(23796.38-30525.06)	38.46(32.43-45.22)	1030676.84(917813.78-1154464.9)	2209218.94(1974033.04-2476589.04)	10.79(7.47-14.37)	3288.73(2168.6-4814.8)	9678.04(6669.19-13202.44)	134.13(79.78-191.18)	85311.75(58015.63-120486.23)	274446.29(192010.52-372463.44)	170.12(105.61-243.7)
Pakistan	Female	4643.7(4095.24-5228.97)	12471.58(11032.12-14170.32)	44.39(37.02-52.18)	489945.04(435290.53-554844.15)	1090318.34(971697.82-1228881.14)	11.65(7.32-16.19)	1314.29(679.2-1904.24)	4355.07(2475.75-6245.76)	159.15(96.17-262.79)	35308.28(20494.32-50397.38)	123418.3(74418.96-175459.01)	191.39(119.36-304.66)
Pakistan	Male	5883.17(5205.14-6729.15)	14427.77(12717.99-16429.6)	33.91(26.53-41.69)	540731.79(477905.11-607694.69)	1118900.59(992008.92-1261912.5)	9.99(6.19-13.92)	1974.44(1215.27-3341.93)	5322.97(3549.04-7899.11)	116.91(57.76-191.75)	50003.47(31736.9-79857.66)	151027.99(102215.87-222484.96)	154.44(82.69-249.98)

Palau	Both	2.89(2.42-3.42)	9.13(7.84-10.62)	143.49(123.9-167.96)	258.39(227.13-296.21)	511.15(463.35-565.42)	66.35(54.19-78.94)	1.68(1.28-2.22)	4.44(3.31-5.85)	63.93(36.33-91.58)	47.2(35.57-62.71)	129.42(95.48-171.99)	97.57(63.65-129.42)
Palau	Female	1.27(1.02-1.54)	4.24(3.59-4.98)	180.35(145.77-225.85)	130.53(113.11-150.79)	245.9(220.72-272.25)	69.9(55.08-85.58)	0.65(0.47-0.89)	1.64(1.18-2.18)	59.42(26.98-96.82)	18.51(13.46-25.33)	47.91(34.8-62.92)	97.19(58.74-138.04)
Palau	Male	1.62(1.36-1.89)	4.89(4.13-5.8)	115.92(97.75-135.38)	127.86(111.76-146.59)	265.26(237.44-297.58)	63.69(52.24-75.27)	1.03(0.77-1.36)	2.8(2.05-3.73)	66.55(29.83-106.45)	28.7(21.18-38.62)	81.51(58.27-109.2)	97.15(55.38-142.66)
Palestine	Both	360.21(314.31-406.5)	1577.81(1384.43-1787.71)	87.85(76.71-100.21)	15098.56(13500.9-16833.56)	48373.89(43641.85-53223.85)	34.24(28.23-41.2)	112.3(81.84-150.79)	205.14(161.12-256.67)	34.64(12.59-59.16)	2580.99(1895.49-3400.94)	5256.96(4154.85-6471.9)	56.59(23.65-94.13)
Palestine	Female	201.53(173.46-231.18)	744.49(653.71-848.5)	58.64(46.19-73.51)	8390.34(7451.31-9398.39)	24736.05(22242.8-27298.35)	24.53(18.31-31.13)	57.91(41.16-77.67)	102.66(79.17-130.09)	30.42(3.26-62.41)	1360.68(979.69-1803.46)	2548.27(2025.54-3174.13)	44.16(11.9-85.97)
Palestine	Male	158.67(137.57-181.69)	833.31(718.16-954.86)	124.92(102.46-148.24)	6708.22(5972.56-7507.83)	23637.84(21262.78-26353.43)	46.44(38.29-56.28)	54.39(38.67-74.38)	102.48(78.12-130.01)	39.09(9.38-71.66)	1220.31(876.04-1650.79)	2708.7(2083.21-3382.8)	70.46(31.39-121.31)
Panama	Both	490.29(424.4-563.94)	1920.79(1721.59-2154.1)	133.8(108.95-161.65)	31436.9(27734.9-35573.42)	83284.83(75648.64-91235.45)	54.7(44.41-65.79)	57.18(45.18-69.88)	333.09(240.28-447.27)	193.22(163.52-224.39)	1607.02(1288.92-1918.96)	7806.83(5882.42-10123.68)	195.87(159.5-229.55)
Panama	Female	218.03(189-250.4)	850.82(747.89-965.93)	130.37(102.16-161.97)	14702.24(12748.36-16879.35)	38113.66(34175.03-42276.14)	49.48(38.59-61.39)	23.8(18.65-29.04)	143.57(103.96-192.53)	194.26(152.51-238.78)	677.03(543.58-809.22)	3300.67(2509.56-4257.35)	193.93(153.14-237.98)
Panama	Male	272.27(233.46-316.78)	1069.97(936.32-1216.39)	137.06(106.34-170.78)	16734.66(14770.99-18871.46)	45171.17(40886.57-49715.02)	59.66(48.4-71.72)	33.38(26.03-40.95)	189.52(136.45-257.97)	192.55(155.39-232.08)	929.99(742.62-1128.2)	4506.17(3361.89-5908.13)	197.75(158.81-238.62)
Papua New Guinea	Both	212.22(179.35-247.54)	730.28(620.67-847.1)	40.69(30.21-52.67)	45136.99(38495.6-52721.72)	117574.13(102312.01-135398.26)	8.92(4.07-14.22)	67.65(49.46-88.51)	205.42(147.63-283.56)	41.44(15.89-72.21)	2440.67(1800.78-3150.46)	7556.08(5479.34-10318.16)	56.03(31.04-86.57)
Papua New Guinea	Female	82.4(68.16-99.04)	278.02(231.85-328.34)	38.72(25.04-54.9)	22286.4(18734.27-26414.38)	56757.66(48118.97-66004.57)	5.6(-0.96-12.25)	23.5(15.75-32.3)	69.17(48.01-96.3)	36.02(-1.12-88.3)	903.56(643.89-1220.32)	2740.01(2017.92-3699.27)	51.06(15.94-99)
Papua New Guinea	Male	129.82(109.38-152.91)	452.26(377.7-528.43)	41.71(28.84-55.77)	22850.59(19477.77-26825.84)	60816.47(52546.13-70271.69)	12.16(6.87-17.81)	44.15(30.76-60.22)	136.25(93.75-187.8)	44.75(13.19-80.85)	1537.12(1104.6-2087.96)	4816.07(3418.51-6656.28)	59.56(27.7-97.9)
Paraguay	Both	550.65(484.24-633.68)	2208.49(1941.81-2469.84)	137.92(119.32-159.05)	30826.28(27291.15-34510.53)	82649.91(75289.19-90153.24)	56.55(48.35-65.61)	88.26(69.34-108.07)	550.09(402.2-726.18)	205.08(169.86-240.62)	2195.23(1737.51-2645.41)	12815.45(9334.59-16979.24)	273.87(220.15-326.43)
Paraguay	Female	266.07(232.14-308.69)	1037.99(910.96-1164.28)	130.96(108.83-157.6)	14798.4(13150.33-16711.12)	38835.19(35237.64-42589.73)	53.5(42.38-65.55)	41.08(31.59-52.13)	256.3(190.74-339.32)	230.57(180.37-283.09)	1027.8(797.7-1258.6)	5856.11(4420.84-7713.8)	283.31(219.18-353.24)
Paraguay	Male	284.57(246.73-328.88)	1170.5(1007.6-1333.28)	144.48(122.15-169.69)	16027.88(14073.62-18009.46)	43814.73(39298.47-48285.34)	59.32(50.81-68.13)	47.18(36.62-58.38)	293.78(204.34-397.64)	186.11(140.4-230.4)	1167.42(909.36-1415.37)	6959.34(4927.68-9419.35)	265.84(200.25-332.01)
Peru	Both	2145.02(1898.96-2410.35)	11168.75(9931.71-12565.53)	230.17(205.36-258.91)	212446.83(159969.31-278137.6)	601350.51(470486.81-766290.58)	81.42(68.37-94.13)	643.15(490.47-816.86)	2106.65(1459.46-2919.19)	201.91(154.91-250.83)	14818.48(11224.15-18719.23)	42777.82(30999.16-57904.21)	256.35(192.06-326.91)
Peru	Female	1143.4(998.64-1291.97)	6101(5339.33-6925.72)	240.37(205.41-274.68)	95027.23(72311.53-124824.33)	280897.65(223969.26-355605.96)	90.57(72.8-108.12)	312.97(237.77-395.46)	1149.67(785.31-1586.39)	224.51(160.9-293.65)	7203.07(5420.35-9090.21)	22919.44(16593.38-30779.47)	282.1(202.5-368.09)
Peru	Male	1001.62(875.53-1152.26)	5067.75(4405.75-5765.64)	218.87(190.08-249.17)	117419.61(87221.53-153912.3)	320452.86(243457.59-413439.77)	73.87(60.43-87.88)	330.18(249.08-425.9)	956.98(651.52-1370.59)	176.75(129.92-230.12)	7615.4(5669.34-9800.46)	19858.38(13758.77-27065.56)	230.19(170.05-303.1)
Philippines	Both	7221.97(6514.3-7960.21)	27385.46(24697.68-30236.56)	129.89(123.8-136.28)	804124.53(719169.07-898220.76)	1920397.16(1748102.08-2101100.27)	36.55(30.86-43.29)	3254.78(2628.14-3942.68)	11287.42(8642.19-14187.53)	100.98(74.17-132.59)	89876.17(70868.1-109380)	319626.44(242429.79-404655.08)	169.98(128.02-220.46)
Philippines	Female	3714.24(3346.14-4102.98)	14509.18(13022.5-15998.71)	138.19(131.13-145.43)	398545.98(357319.66-446458.48)	956260.4(872322.19-1041390.1)	37.81(30.47-45.89)	1579.65(1262.34-1925.15)	5403.45(3961.58-7118.73)	89.49(60.84-120.32)	42742.76(33917.53-52357.3)	147479.88(108690.62-198320.03)	154.84(107.26-208.8)
Philippines	Male	3507.74(3172.89-3868.9)	12876.28(11587.41-14269.72)	121.35(114.54-128.88)	405578.56(362643.42-452698.51)	964136.76(875272.36-1063126.76)	35.33(30.46-41.09)	1675.13(1303.4-2095.93)	5883.97(4229.78-7977.12)	110.01(73.42-159.6)	47133.41(35935.32-59429)	172146.56(122533-234912.81)	182.39(124.59-257.57)
Poland	Both	9139.74(7894.83-10618.04)	19117.6(16667.57-21953.17)	125.91(113.28-138.88)	564627.65(516675.64-616369.54)	799673.13(738585.84-865805.79)	39.9(35.12-44.73)	992.2(753.45-1221.59)	1130.27(833.98-1481.54)	7.94(-6.08-24.2)	26287.74(20313.78-32297.41)	26989.62(20772.64-33792.69)	12.79(1.43-26.57)
Poland	Female	4864.54(4192.37-5661.56)	10587.22(9193.74-12156.44)	131.96(117.22-147.71)	314092.79(287933.58-343158.14)	450325.85(415295.24-487574.55)	40.77(35.52-46.23)	533.39(410.29-653.68)	649.02(456.26-873.46)	9.94(-7.38-32.73)	13571.82(10546.03-16430.98)	14472.93(10948.91-18519.81)	12.69(-1.94-30.42)
Poland	Male	4275.2(3709.14-4980.71)	8530.37(7420.85-9838.15)	118.67(106.76-131.58)	250534.86(228743.66-273955.84)	349347.28(321517.68-379199.75)	38.65(34.07-43.37)	458.81(339.58-586.21)	481.25(334.36-660.45)	3.73(-8.31-17.88)	12715.92(9574.67-15951.35)	12516.69(9192.36-16505.4)	11.49(-0.01-24.25)
Portugal	Both	4017.88(3468.6-4603.24)	7622.25(6678.19-8588.45)	86.79(73.2-101.49)	124501.87(113135.52-137065.27)	197474.74(181323.05-214745.76)	48.62(42.33-56)	331.32(236.9-446.1)	861.45(596.45-1224.71)	121.9(74.7-177.55)	7214.92(5374.71-9287.31)	13020.84(9852.61-16794.51)	87.57(59.78-121.33)
Portugal	Female	2304.3(1996.19-2654.98)	4378.16(3841.3-4906.63)	83.26(67.65-101.64)	66662.83(60184.26-74022.05)	107795.59(98336.65-118400.25)	50.13(42.86-58.91)	169.01(121.53-225.71)	502.56(340.4-726.68)	141.9(85.14-209.97)	3628.52(2733.35-4628.93)	7207.03(5452.39-9449)	93.74(62.16-132.09)
Portugal	Male	1713.57(1446.69-1989.5)	3244.09(2739.43-3790.59)	90.86(74.27-111.39)	57839.05(52025.63-63860.12)	89679.14(81462.92-98699.44)	46.81(39.02-54.73)	162.32(111.73-225.91)	358.89(248.31-510.18)	97.54(58.46-146.82)	3586.4(2583.63-4666.78)	5813.8(4294.69-7546.73)	78.95(51.03-112.97)
Puerto Rico	Both	1074.91(947.67-1214.93)	2831.39(2486.77-3197.84)	151.45(129.95-179.5)	55386.78(50236.3-61429.82)	103809.45(94905.36-113291.19)	89.46(77.57-102.29)	293.58(236.65-348.23)	639.31(461.03-831.03)	68.86(49.93-88.79)	6659.49(5429.58-7748.68)	13110.2(10085.4-16768.24)	93.61(73.86-114.51)
Puerto Rico	Female	530.86(462.94-610.16)	1410.09(1211.83-1622.2)	148.3(120.18-183.82)	27988.69(25078.34-31351.44)	52344.96(47316.18-57432.1)	85.58(71.86-100.76)	135.46(107.95-163.93)	314.72(226.13-412.65)	53.74(33.14-75.67)	2970.77(2432.05-3488.85)	6107.5(4769.12-7709.16)	79.76(59.55-102.91)
Puerto Rico	Male	544.05(473.24-621.24)	1421.31(1238.13-1632.27)	155.36(127.34-191.21)	27398.09(24735.06-30352.83)	51464.49(47130.82-56077.94)	93.81(80.76-109.18)	158.13(126.26-188.38)	324.59(230.93-426.78)	80.55(60.32-102.69)	3688.72(2989.97-4318.61)	7002.7(5349.7-9107.82)	105.56(84.63-128.93)
Qatar	Both	52.09(44.84-60.03)	868.14(728.36-1027.04)	173.81(152.45-197.19)	3772.8(3148.39-4544.76)	31960.45(27468.14-36725.83)	30.39(21.59-39.66)	9.45(6.96-14.9)	45.8(32.71-62.25)	44.52(-1.23-78.82)	254.8(193.38-370)	1528.03(1161.24-1980.6)	37.09(-0.64-71.54)
Qatar	Female	19.36(16.68-22.32)	199.48(167.27-232.43)	120.13(101.6-139.34)	1176.84(987.37-1406.42)	7840.86(6759.03-9024.01)	34(23.86-44.84)	4.61(3.2-8.68)	18.12(12.99-24.78)	59.02(-8.93-115.01)	112.16(81.44-185.52)	520.86(393.32-679.96)	36.66(-16.26-83.33)
Qatar	Male	32.73(27.6-38.51)	668.67(553.98-794.76)	196.88(169.53-227.59)	2595.96(2129.16-3144.21)	24119.58(20611.47-27918.11)	27.92(19.28-38.04)	4.84(3.4-7.17)	27.69(18.97-38.87)	50.75(16.03-91.73)	142.64(104.56-201.01)	1007.18(751.72-1342.14)	44.53(9.29-84.93)
Republic of Korea	Both	8014.35(6976.67-9230.57)	29455.01(26593.66-32189.85)	219.69(184.56-257.47)	435588.37(385700.28-490440.16)	1047141.41(974139.9-1127293.57)	98.19(84-114.05)	1209.13(1038.69-1377.05)	3833.41(3194.01-4476.31)	136.05(102.51-171.83)	32847.46(27693.16-37322.64)	74259.31(64329.5-84422.72)	112.92(87.81-142.02)

Republic of Korea	Female	4217.1(3664.52-4878.61)	14884.95(13167.97-16578.83)	208.83(174.08-255.05)	236103.7(206379.78-269707.77)	536947.82(494304.02-583514.49)	88.73(73.57-105.42)	585.8(502.29-674.79)	1981.44(1564.09-2398.46)	133.36(88.73-179.49)	15347.92(13083.17-17598.17)	34851.55(29071.93-40603.78)	100.14(69.95-133.44)
Republic of Korea	Male	3797.25(3253.9-4412.28)	14570.06(13029.74-16175.38)	231.77(184.92-280.74)	199484.66(175533.27-224335.36)	510193.59(468941.69-552992.81)	109.44(93.93-127.59)	623.33(522.16-720.39)	1851.98(1557.25-2169.54)	135.28(101.99-176.44)	17499.54(14485.41-20190.08)	39407.76(33671.1-45534.58)	124(96.83-159.87)
Republic of Moldova	Both	418.69(360.32-486.32)	797.18(689.44-928.65)	131.12(112.85-152.26)	78385.59(69160.85-88828.83)	90499.64(81503.12-99899.48)	37.61(30.27-45.2)	13.03(8.91-18.12)	32.98(22.7-46.38)	163.58(125.99-206.93)	520.61(395.38-670.15)	1119.03(824.26-1458.71)	176.41(144.5-211.51)
Republic of Moldova	Female	251.31(212.61-296.93)	423.88(359.61-500.92)	102.16(82.98-125.5)	41352.16(35980.94-47290.97)	46910.16(41853.55-52381.59)	35.21(26.73-44.14)	6.6(4.56-9.3)	18.39(12.44-25.87)	194.47(149.07-249.42)	278.73(209.64-359.26)	603.97(439.95-796.11)	182.3(141.04-227.2)
Republic of Moldova	Male	167.38(141.5-196.85)	373.3(317.89-433.3)	174.65(146.42-207.93)	37033.43(32504.5-42334.63)	43589.48(38819.16-48534.5)	40.3(32.84-48.4)	6.43(4.48-9.02)	14.59(9.95-20.78)	132.99(95.71-177.9)	241.88(180.34-313.72)	515.06(371.5-676.57)	170.76(135.14-207.61)
Romania	Both	4488.54(3938.36-5103.09)	9736.17(8689.74-10883.17)	178.75(155.25-205.73)	316670.36(283833.15-352105.3)	404079.23(370138.3-439860.94)	55.34(46.81-63.91)	263.72(155.96-366.11)	470.84(311.86-683.69)	66.78(33.27-160.11)	8118.69(5469.75-10593.9)	14652.3(10800.05-19072.35)	119.68(86.31-204.53)
Romania	Female	2326.79(2018.52-2694.23)	4771.44(4195.52-5395.49)	157.86(130.66-189.07)	167744.16(147921.44-189370.23)	215532.78(194716.08-237178.08)	54.48(44.92-64.34)	153.9(87.07-213.06)	260.76(170.96-379.98)	51(19.29-145.28)	4452.45(2911.82-5795.1)	7600.49(5660.71-9982.14)	103.04(70.02-188.17)
Romania	Male	2161.74(1860.19-2483.81)	4964.74(4353.85-5637.34)	202.23(170.11-234.32)	148926.2(132475.17-166036.66)	188546.45(171515.44-206777.38)	56.19(47.47-64.58)	109.82(67.41-153.32)	210.08(136.44-304.21)	86.43(47.82-178.44)	3666.25(2505.94-4896.4)	7051.82(5189.44-9317.38)	138.66(97.84-221.74)
Russian Federation	Both	26268.79(23160.51-29550.89)	49417.33(43755.07-56370.51)	94.83(87.99-101.91)	3151492.04(2891945.81-3441295.37)	3841137.49(3535626.37-4154865.89)	25.49(21.33-29.82)	956.02(654.49-1314.73)	1599.14(1118.05-2211.19)	51.67(32.64-75.55)	39637.5(29945.84-51487.28)	55779.9(42329.04-72031.9)	45.23(32.58-60.12)
Russian Federation	Female	17139.1(15181.22-19197.2)	26349(23438.52-29779.43)	55.71(50.87-60.55)	1771731.75(1623562.83-1926628.1)	2084480.25(1920320.08-2244646.92)	20.36(16.24-24.5)	598.76(413.52-811.98)	1002.66(697.68-1417.49)	53.29(31.99-79.69)	23770.31(17856.44-30584.74)	31786.83(23900.17-41519.05)	36.81(24.29-50.13)
Russian Federation	Male	9129.69(7905.09-10492.38)	23068.33(20166.89-26669.79)	168.61(154.83-183.46)	1379760.29(1253914.71-1516082.06)	1756657.24(1609520.34-1908429.33)	32.07(27.23-36.93)	357.26(236.41-508.93)	596.48(396.91-830.59)	49.87(28.97-75.53)	15867.19(11599.16-21058.64)	23993.07(17959.9-31202.26)	57.3(40.62-78.55)
Rwanda	Both	344.02(302.65-393.82)	929.46(816.87-1060.37)	69.95(58.18-82.78)	39432.76(34054.91-45890.15)	81083.47(71136.73-92697.52)	18.84(12.94-25.1)	210.46(152.93-272.67)	312.05(220.33-417.94)	140.88(90.32-202.28)	5303.98(3885.9-6912.38)	7572.59(5474.5-10062.17)	153.26(102.31-219.85)
Rwanda	Female	158.08(138.32-182.67)	491.35(424.71-565.78)	98.12(80.69-117.4)	20445.8(17531.45-23686.54)	42662.71(37345.67-48580.03)	19.56(11.91-27.42)	97.37(67.34-129.59)	171.9(114.15-236.83)	179.3(95.16-275.61)	2533.11(1772.19-3356.35)	4045.96(2793.21-5540.84)	178.54(102.43-269.47)
Rwanda	Male	185.94(162.48-211.62)	438.11(380.46-506.23)	46.09(34.03-59.33)	18986.97(16190.1-22194.14)	38420.76(33347.12-44503.46)	18.04(12.41-24.36)	113.09(77.72-154.04)	140.15(96.35-194.28)	106.31(57.18-178.76)	2770.87(1905.49-3790.37)	3526.63(2479.46-4827.86)	129.38(73.72-212.31)
Saint Kitts and Nevis	Both	12.24(9.91-15.31)	34.68(27.69-43.61)	96.63(67.94-129.09)	583.47(528.84-646.92)	1203.6(1093.48-1312.32)	41.47(33.8-49.76)	4.95(3.84-6.06)	9.07(6.83-11.57)	78.31(50.63-111.27)	111.13(88.58-134.76)	237.25(178.95-305.32)	81.32(49.91-116.95)
Saint Kitts and Nevis	Female	5.85(4.63-7.6)	12.58(10.3-15.68)	53.22(28.21-84.99)	293.98(262.24-331.31)	539.61(484.74-597.57)	28.71(20.84-37.21)	2.5(1.92-3.11)	4.03(3.01-5.14)	95.98(64.75-134.92)	55.13(43.73-67.6)	97.79(74.02-125.8)	81.49(50.19-121.18)
Saint Kitts and Nevis	Male	6.39(5.17-7.93)	22.1(17.3-28.35)	133.59(93.59-176.36)	289.5(262.28-320.11)	663.99(600.73-729.02)	53.71(44.62-63.59)	2.45(1.9-3.01)	5.04(3.77-6.46)	65.29(36.44-96.84)	56(43.84-68.04)	139.46(103.61-180.91)	80.16(45.54-114.44)
Saint Lucia	Both	22.9(20.08-26.05)	84.48(74.8-96.08)	179.71(160.23-200.71)	1449.13(1285.33-1631.72)	3521.98(3220.13-3837.74)	88.62(76.78-100.77)	7.36(5.91-8.82)	19.9(15.38-24.8)	74.28(52.09-102.15)	178.96(143.66-213.12)	488.06(380.53-605.73)	105.62(80.25-134.88)
Saint Lucia	Female	11.19(9.76-12.92)	39.4(34.34-44.85)	172.73(147.43-200.77)	746.03(661.23-844.81)	1701.25(1542.21-1864.23)	80.22(66.85-93.7)	3.4(2.74-4.16)	8.97(6.66-11.5)	87.69(49.99-124.62)	82.32(65.57-99.15)	214.28(164.41-271.65)	108.5(72.74-147.87)
Saint Lucia	Male	11.71(10.1-13.5)	45.08(39.39-51.74)	185.34(159.43-215.38)	703.1(616.91-792.26)	1820.72(1650.39-1997.04)	97.2(84.73-111.13)	3.95(3.14-4.78)	10.93(8.31-13.8)	63.6(43.24-88.88)	96.63(77.26-115.18)	273.78(208.74-341.51)	102.66(78.69-133.09)
Saint Vincent and the Grenadines	Both	17.33(15.08-19.62)	47.77(42.11-53.36)	157.65(139.51-177.05)	1139.74(1014.04-1283.56)	2135.16(1942.02-2329.66)	82.26(70.93-93.48)	5.03(4.04-6.06)	13.46(10.54-16.42)	90.43(68.41-117.41)	122.03(97.97-146.64)	332.14(264.48-406.92)	139.85(111.52-172.47)
Saint Vincent and the Grenadines	Female	8.81(7.59-10.16)	21.45(18.82-24.25)	134.74(112.1-159.67)	582.69(515.15-658.88)	975.41(876.69-1069.11)	66.99(56.01-79.06)	2.67(2.12-3.27)	6.22(4.84-7.74)	94.73(68.83-128.06)	62.71(50.38-75.66)	150.59(119.04-186.33)	131.68(101.24-169.24)
Saint Vincent and the Grenadines	Male	8.52(7.31-9.68)	26.32(22.86-30.1)	179.84(155.42-207.64)	557.05(490.7-633.51)	1159.74(1051.43-1271.67)	97.57(83.94-112.37)	2.37(1.86-2.91)	7.24(5.64-8.95)	89.02(62.86-119.45)	59.32(46.8-72)	181.55(142.88-222.87)	149.41(114.77-188.02)
Samoa	Both	22.19(19.2-25.4)	50.03(44.06-56.46)	59.77(49.43-69.9)	2116.2(1855.58-2453.81)	3447.12(3095.51-3866.11)	25.82(19.6-32.88)	10.57(7.78-14.21)	21.06(16.11-26.96)	52.53(25.92-87.57)	287.51(211.6-386.95)	569.41(427.41-736.14)	69.91(41.41-106.21)
Samoa	Female	10.13(8.6-11.79)	22.46(19.53-25.66)	55.58(42.3-69.61)	1079.08(932.46-1254.91)	1729.13(1534.82-1960.28)	21.21(14.2-28.85)	5.35(3.61-7.57)	10.57(7.71-14.19)	35.17(3.11-94.46)	145.39(102.59-202.83)	290.09(213.29-384.94)	56.58(19.75-112.95)
Samoa	Male	12.06(10.43-13.89)	27.58(24.12-31.79)	63.47(50.08-77.32)	1037.12(894.41-1204.46)	1717.99(1531.36-1954.06)	30.51(23.62-38.43)	5.22(3.76-7.04)	10.48(7.91-13.84)	68.61(24.57-111.91)	142.12(102.92-192.6)	279.31(206.19-372.42)	81.24(38.15-122.87)
San Marino	Both	8.3(7.25-9.44)	17.35(15.27-19.48)	50.04(40.28-59.99)	267.68(245.05-292.26)	495.74(453.85-537.72)	30.46(26.09-35.33)	0.27(0.19-0.39)	0.87(0.52-1.39)	75.58(42.73-117.8)	6.84(5.21-9)	15.86(11.3-22.14)	51.1(32.78-74.36)
San Marino	Female	4.66(4.03-5.37)	9.3(8.14-10.5)	39.39(27-53.13)	136.75(124-149.65)	255.71(231.04-278.46)	28.41(22.65-34.73)	0.15(0.1-0.21)	0.51(0.29-0.83)	75.62(33.74-130.5)	3.64(2.71-4.81)	8.77(6.16-12.55)	48.74(25.89-77.34)
San Marino	Male	3.63(3.11-4.19)	8.05(6.91-9.32)	63.64(49.5-80.77)	130.93(118.99-144.8)	240.02(218.72-262.82)	32.81(26.95-39.02)	0.12(0.08-0.18)	0.36(0.21-0.56)	70.98(31.51-126.4)	3.2(2.36-4.3)	7.09(4.95-9.86)	52.17(28.21-81.38)
Sao Tome and Principe	Both	13.5(11.88-15.33)	30.35(26.85-34.68)	39.82(29.23-54.67)	814.08(726.3-915.67)	1678.32(1494.54-1890.51)	24.48(19.8-29.45)	5.88(4.29-7.72)	11.45(8.19-15.11)	111.65(73.42-159.6)	133.99(97-175.62)	272.08(196.43-361.18)	168.46(118.6-239.69)
Sao Tome and Principe	Female	7.34(6.39-8.44)	17.07(14.87-19.56)	47.14(34.55-64.62)	433.15(383.04-488.25)	856.23(764.51-963.35)	20.17(14.62-25.77)	3.16(2.3-4.16)	6.39(4.43-8.5)	125.66(77.02-185.06)	72.67(52.32-94.83)	152.58(105.7-206.95)	176.78(114.88-256.75)
Sao Tome and Principe	Male	6.16(5.41-7.05)	13.28(11.61-15.26)	31.58(18.47-48.96)	380.92(333.9-432.64)	822.09(719.27-932.16)	29.4(23.24-35.85)	2.72(1.9-3.71)	5.06(3.51-6.81)	97.54(45.44-159.23)	61.32(42-82.79)	119.5(81.41-162.06)	158.44(95.2-251.76)
Saudi Arabia	Both	2504.49(2193.36-2849.9)	15220.11(13266.71-17445.39)	187.23(165.28-211.49)	117579.05(103496.61-133779.15)	460863.59(416129.02-511467.2)	74.18(64.85-84.63)	736.26(530.97-1000.5)	2070.91(1498.98-2800.85)	81.14(36.61-133.64)	18627.84(13098.43-25196.73)	60772.15(44191.1-81136.88)	111.64(58.05-175.56)
Saudi Arabia	Female	1105.83(959.75-1271.74)	5688.86(4868.52-6690.59)	156.61(129.46-188.28)	51287.77(44933.59-58480.27)	190793.92(169759.77-212371.84)	74.24(62.05-86.56)	290.22(211.28-401.91)	879.81(610.37-1225.83)	140.29(72.69-212.1)	7486.57(5394.78-10034.89)	26353.44(18788.99-36338.19)	157.73(77.7-256.02)
Saudi Arabia	Male	1398.66(1206.66-1602.63)	9531.25(8279.16-10911.78)	208.43(182.05-237.09)	66291.28(57673.7-75389.38)	270069.67(241842.72-301903.1)	73.9(63.61-85.61)	446.04(302.9-624.4)	1191.1(840.28-1629.55)	52.08(4.49-119.83)	11141.27(7548.2-15618.42)	34418.71(24720-46591.12)	84.58(28.34-162.29)
Senegal	Both	558.44(490.66-631.51)	1613.74(1426.28-1813.46)	48.5(39.83-58.46)	45464.68(39718.77-52135.06)	106387.7(93902.5-120182.39)	19.05(14.31-23.74)	297.45(210.02-407.97)	617.99(440.07-851.36)	99.57(55.81-153.21)	6894.08(4856.57-9327.32)	14175.01(9959.06-19680.88)	131.39(75.02-203.48)

Senegal	Female	271.28(234.21-310.64)	798.17(695.83-915.81)	56.93(45.19-69.93)	22905.47(19844.77-26185.69)	53343.36(46838.93-60424.23)	21.44(14.84-27.71)	135.94(92.66-213.68)	284.5(195-429.43)	96.32(51.78-157.36)	3249.24(2174.45-4963.42)	6556.36(4464.81-9756.71)	122.88(65.19-202.21)
Senegal	Male	287.16(250.54-325.93)	815.58(715.65-922.58)	40.45(29.65-52.27)	22559.21(19568.02-25980.1)	53044.34(46547.99-60715.91)	16.62(11.62-21.68)	161.52(110.83-231.96)	333.49(230.89-470.54)	102.43(50.31-169.27)	3644.84(2527.31-5179.57)	7618.65(5145.28-10941.58)	139.32(72.97-233.82)
Serbia	Both	2276.51(1991.29-2591.52)	5380.23(4671.7-6121.61)	163.64(139.27-190.49)	147581.55(133434.68-162340.1)	201669.46(185104.18-218667.9)	46.68(38.9-55.68)	319.74(223.97-424.92)	587.32(394.44-827.58)	51.43(17.98-85.7)	8253.96(5934.82-10827.76)	12769.71(9042.34-17150.55)	64.23(32.79-96.81)
Serbia	Female	1086.07(924.48-1241.27)	2584.43(2217.24-2981.34)	167.88(138.47-203.22)	71762.98(64005.77-80062.68)	100759.12(91202.02-111071.18)	51.83(42.33-62.88)	147.34(102.46-200.8)	304.73(203.39-434.68)	62.54(24.03-107.6)	3802.52(2734.41-5018.13)	6354.26(4574.69-8480.08)	68.59(34.47-108.17)
Serbia	Male	1190.44(1027.97-1369.74)	2795.8(2405.15-3211.06)	159.48(128.25-193.25)	75818.57(68003.7-84104.65)	100910.34(92538.97-109692.8)	41.76(32.6-51.5)	172.41(115.66-249.28)	282.59(180.58-409.91)	41.3(4.42-86.84)	4451.44(3117.94-6192.74)	6415.45(4383.65-8726.39)	59.87(22.25-99.82)
Seychelles	Both	16.2(14.09-18.43)	48.88(42.85-54.99)	115.25(96.24-136.32)	1268.7(1126.17-1438.68)	2616.91(2366.15-2893.69)	47.37(39.09-55.65)	6.39(5.2-7.62)	17.15(13.8-20.43)	85.59(63.85-110.12)	149.25(121.61-176.93)	412.33(335.47-492.37)	92.68(70.43-116.06)
Seychelles	Female	8.71(7.54-9.97)	24.55(21.38-27.8)	113.59(90.74-139.32)	645.72(570.79-732.46)	1233.04(1115.65-1376.43)	44.56(34.92-54.09)	3.26(2.62-3.98)	9.16(7.08-11.49)	94.98(65.89-128.8)	71.23(58.42-85.92)	199.24(158.3-244.76)	103.24(74.96-135.4)
Seychelles	Male	7.48(6.44-8.59)	24.33(20.89-27.61)	119.16(95.66-144.82)	622.98(545.59-713.75)	1383.87(1232.81-1542.79)	50.01(41.6-58.86)	3.13(2.41-3.82)	7.99(6.19-9.88)	76.05(47.12-111.37)	78.03(60.8-95.19)	213.09(163.88-264.16)	84.86(53.97-122)
Sierra Leone	Both	287.15(252.15-324.08)	713.2(624.87-809.43)	13.33(5.76-21.73)	22091.55(19779.33-24664.18)	51148.26(45457.65-57618.73)	3.38(0-7.04)	136.42(96.86-180.91)	223.69(161.15-307.15)	49.96(22.35-83.52)	3005.84(2107.74-3920.49)	5466.5(3881.89-7506.03)	75.81(41.06-118.85)
Sierra Leone	Female	138.33(120.82-158.75)	363.07(317.28-416.85)	22.04(11.69-33.82)	10986.06(9685.49-12520.94)	25366.08(22376.87-28657.51)	4.05(-0.42-8.61)	57(39.54-81.42)	106.24(72.35-147.72)	64.93(24.07-113.5)	1293.96(901.5-1798.53)	2641.19(1816.86-3716.55)	95.65(48.96-156.35)
Sierra Leone	Male	148.81(129.3-169.93)	350.13(302.23-402.15)	5.24(-3.52-14.53)	11105.5(9842.12-12505.7)	25782.18(22604.62-29251.16)	2.66(-1.52-7.49)	79.42(55.01-109.36)	117.45(80-165.42)	39.12(7.87-80.46)	1711.88(1177.77-2335.88)	2825.31(1926.1-3934.3)	60.77(25.26-108.78)
Singapore	Both	753.46(694.54-817.88)	3162.23(2837.18-3523.49)	133.55(114.9-155.35)	49587.42(44009.76-55421.14)	144489.39(132031.46-158621.11)	55.22(47.14-64.28)	130.23(110.84-150.52)	328.87(265.22-391.89)	53(32.9-74.92)	3318.03(2791.5-3825.07)	7652.02(6380.8-8985.56)	45.43(29.29-62.99)
Singapore	Female	343.59(313.63-376.26)	1464.1(1295.46-1638.46)	140.1(118.21-165.01)	25258.07(22093.18-28582.58)	68900.16(62307.16-76254.53)	47.98(39.21-58.33)	74.07(62.66-86.19)	190.79(149.01-229.48)	47.98(24.83-72.21)	1807.86(1540.97-2085.6)	4062.54(3359.25-4752.58)	37.51(20.96-56)
Singapore	Male	409.87(374.57-450.52)	1698.13(1506.93-1918.01)	127.66(105.36-153.07)	24329.36(21583.8-27287.43)	75589.24(68279.79-83149.93)	62.5(52.68-72.42)	56.16(46.59-65.89)	138.09(112.37-165.18)	55.66(34.96-81.55)	1510.16(1235.16-1783.86)	3589.48(2926.02-4243.4)	54(36.1-76.27)
Slovakia	Both	1227.76(1081.26-1400.97)	2796.07(2400.12-3206.49)	135.91(118.35-156.93)	75586.39(68458.55-83897.51)	112796.9(103395.55-122813.46)	43.68(36.52-51.06)	180.47(129.51-230.18)	196.36(132.67-273.65)	9.22(-7.53-27.59)	4636.2(3423.07-5827.52)	4942.8(3643.06-6561.38)	14.65(0.1-30.61)
Slovakia	Female	643.38(554.55-754.78)	1422.86(1227.96-1663.43)	125.84(103.78-149.48)	40228.07(35871.55-44835.96)	59992.04(54402.15-65583.56)	43.52(35.5-52.37)	96.23(69.16-122.73)	116.86(78.24-163.95)	9.94(-11.84-34.8)	2356.94(1753.13-2957.11)	2671.31(1967.42-3544.47)	12.52(-7.14-33.71)
Slovakia	Male	584.37(507.18-678.49)	1373.21(1170.53-1583.53)	147.08(121.68-176.3)	35358.33(31720.47-39516.91)	52804.86(47971.54-58028.27)	43.87(36.09-52.19)	84.24(59.33-111.42)	79.5(52.06-112.44)	3.86(-14.46-26.28)	2279.26(1633.03-2925.93)	2271.49(1637.47-3059.82)	14.35(-3.41-36.08)
Slovenia	Both	453.19(396.26-515.33)	1195.89(1046.66-1334.58)	163.94(140.92-190.29)	29265.19(26469.88-32402.12)	46613.58(42845.08-50417.76)	50.8(42.12-59.24)	28.11(19.88-38.71)	47.79(31.11-69.92)	51.6(16.18-95.17)	730.15(564.31-937.11)	1153.2(860.74-1545.86)	63.18(37.03-93.85)
Slovenia	Female	255.3(220.48-291.77)	572.75(498.19-650.07)	126.05(98.3-154.09)	16008.59(14395.67-17786.74)	24146.55(22007.53-26444.29)	45.85(37.28-54.87)	16.68(11.72-23.43)	28.42(18.35-42.38)	52.06(12.23-104.14)	408.12(311.66-529.47)	607.49(445.2-810.16)	53.01(24.82-85.87)
Slovenia	Male	197.9(173.53-227.1)	623.14(537.67-707.9)	212.47(177.21-250.05)	13256.6(11807.42-14884.78)	22467.03(20396.27-24573.71)	56.89(46.02-66.97)	11.43(8-15.82)	19.37(12.58-28.49)	50.98(15.7-91.95)	322.04(244.23-411.95)	545.71(396.12-737.38)	75.87(44.57-110.5)
Solomon Islands	Both	27.2(22.67-32.78)	68.65(57.95-81)	30.27(20.85-42.37)	3535.38(3026.42-4126.92)	8080.35(6985.08-9283.19)	19.64(14.03-25.66)	18.75(12.5-26.19)	25.32(17.93-33.35)	-22.83(-41.16--2.31)	678.98(457.4-953.45)	990.78(700.47-1323.32)	-6.83(-27.7-17.02)
Solomon Islands	Female	11(8.82-13.83)	28.79(24.11-34.53)	32.71(19.94-48.82)	1727.48(1447.46-2043.71)	4059.96(3511.11-4687.99)	20.69(13.51-28.89)	9.46(5.69-13.82)	12.7(9.05-17.31)	-28.56(-49.08-9.87)	354.76(219.9-514.47)	506.58(361.35-675.97)	-11.93(-35.1-26.9)
Solomon Islands	Male	16.2(13.56-19.12)	39.86(33.61-46.86)	29.13(17.52-42.12)	1807.9(1545.56-2106)	4020.39(3458.17-4691.11)	18.55(12.53-24.51)	9.28(6.01-14.24)	12.61(8.86-16.89)	-18.23(-46.33-10.6)	324.22(216.22-488.57)	484.2(341.83-656.83)	-2(-32.57-29.72)
Somalia	Both	288.01(254.27-328.69)	893.39(782.15-1015.08)	12.24(6.02-19.31)	37411.73(32553.86-43466.2)	100977.69(87846.92-117630.28)	-3.9(-7.54-0.29)	164.08(113.47-230.66)	402.43(269.75-567.43)	55.83(27.91-88.87)	4438.6(3043.63-6246.99)	10941.83(7576.59-15273.9)	61.1(31.13-98.19)
Somalia	Female	132.4(114.55-151.53)	456.93(397.41-523.38)	24.55(14.77-35.4)	18721.31(16153.7-21981.79)	50454.02(43911.47-58680.99)	-5.54(-10.43--0.52)	70.92(42.47-113.53)	205.29(130.77-311.2)	80.97(38.72-141.07)	1979.21(1228.88-3070.5)	5465.75(3608.15-8107.07)	80.58(39.05-136.78)
Somalia	Male	155.6(136.02-177.58)	436.46(378.9-502.03)	1.74(-5.64-9.5)	18690.42(16107.38-21826.85)	50523.67(43345.32-59806.12)	-2.29(-7.71-2.84)	93.16(63.29-132.55)	197.14(131.76-277.65)	36.29(6.4-74.45)	2459.4(1672.22-3488.89)	5476.09(3688.33-7800.24)	45.47(14.36-87.89)
South Africa	Both	4500.65(4002.49-5042.48)	13882.61(12514.04-15494.24)	111.47(103.54-120.42)	319073.72(289608.18-351352.54)	689689.57(632202.7-749008.99)	44.65(39.65-49.73)	827.52(616.8-1065.07)	2846.57(2184.61-3552.81)	93.78(67.76-121.35)	21771.05(16448.93-27695.89)	67447.84(51748.68-83850.26)	114.66(87.39-146.99)
South Africa	Female	2126.75(1879.35-2415.32)	6727.19(5996.66-7586.41)	119.57(110.85-129.77)	170402.4(155077.26-187637.68)	364579.43(334062.51-395821.71)	44.6(38.79-50.65)	382.96(285.26-509.33)	1433.66(1089.02-1805.75)	98.03(62.28-133.12)	9969.36(7668.4-12600.98)	31600.96(24675.14-39349.07)	110.62(75.51-148.19)
South Africa	Male	2373.91(2130.48-2653.48)	7155.42(6460.65-7918.77)	103.86(94.45-115.24)	148671.32(134581.09-163893.6)	325110.14(296463.41-354635.41)	44.8(39.86-50.11)	444.56(327.6-581.05)	1412.91(1070.68-1782.95)	88.88(57.31-121.32)	11801.68(8696.1-15151.72)	35846.89(26713.44-45405.74)	118.26(86.09-156.13)
South Sudan	Both	297.28(261.44-332.81)	551.24(485.62-621.05)	19.25(12.26-26.91)	32461.43(28023.41-37994.83)	52768.52(46436.41-60192.53)	3.74(-1.88-9.67)	148.48(99.3-216.95)	220.56(145.71-310.5)	60.67(20.75-111.56)	3410.63(2309.42-4886.87)	5205.54(3433.3-7392.25)	70.56(25.08-135)
South Sudan	Female	112.21(98-127.33)	232.36(202.17-265.86)	28.48(19.05-38.24)	14262.56(12096.42-16990.95)	25468.09(21749.07-29447.89)	7.39(0.47-14.51)	54.9(35.88-85.25)	91.39(60.49-132.82)	79.94(26.55-151.22)	1240.43(831.3-1882.3)	2216.07(1496.73-3161.7)	95.33(33.84-179.65)
South Sudan	Male	185.07(162.07-208.37)	318.88(278.6-360.79)	14.75(5.98-23.94)	18198.86(15592.44-21211.47)	27300.43(23762.99-31101.12)	1.3(-4.18-7.56)	93.58(59.22-139.39)	129.17(79.47-193.97)	49.4(9.84-100.16)	2170.2(1386.54-3189.46)	2989.46(1864.67-4515.51)	56.79(13.02-121.63)
Spain	Both	18008.11(15974.47-20034.57)	29756.98(26466.23-33032.33)	38.78(29.04-50.95)	463241.12(424814.92-504104.38)	718565.83(659713.18-776936.5)	29.37(23.18-36.16)	1393.28(976.71-1910.7)	2363.86(1556.14-3526.13)	29.45(-0.37-65.75)	27203.81(20246.88-34915.96)	33540.86(25024.09-44642.83)	11.29(-6.22-31.24)
Spain	Female	10471.57(9194.83-11815.71)	16396.2(14505.71-18498.22)	29.95(18.57-42.81)	250617.15(228358.19-273626.13)	382476.31(345968.23-417919.5)	27.49(20.2-35.6)	804.75(562.32-1121.08)	1444.47(912.86-2194.33)	27.67(-3.47-65.39)	15117.62(11321.01-19738.44)	19046.33(13829.5-25540.54)	4.88(-13.39-27.62)

Spain	Male	7536.54(6611.56-8448.52)	13360.78(11749.15-15056.05)	50.84(35.13-69.06)	212623.98(194595.25-232654.84)	336089.52(308064.74-366927.99)	31.61(24.17-39.34)	588.53(415.52-806.94)	919.39(620.39-1353.05)	27.31(-2.1-60.76)	12086.19(8853.54-15630.83)	14494.52(10926.51-19734.76)	16.81(-2.12-39.02)
Sri Lanka	Both	2772.3(2433.06-3138.63)	10448.85(9104.86-11702.81)	206.03(182.29-231.24)	271585.2(237347.18-310046.19)	559767.19(509117.17-616165.66)	64.09(52.9-76.21)	878.35(706.68-1055.98)	2240.86(1645.07-3074.18)	97.94(66.82-137.03)	23983.37(19648.64-28946.56)	56366.13(42318.83-75014.69)	128.35(91.65-171.59)
Sri Lanka	Female	1282.12(1105.33-1472.77)	5633.55(4823.41-6455.88)	243.8(207.56-287.31)	129994(112395.41-150142.7)	287242.49(260050.77-317851.33)	69.05(55.35-83.91)	321.75(241.97-407.85)	1060.13(764.88-1471.16)	105.25(61.67-174.76)	9013.85(7098.98-11167.3)	25717.73(19309.25-34585.94)	144.2(94.21-212.96)
Sri Lanka	Male	1490.18(1292.85-1701.3)	4815.31(4173.81-5516.42)	172.09(143.93-202.54)	141591.2(123156.42-162805.24)	272524.7(244847.24-304757.37)	59.74(49.26-70.72)	556.6(446.07-680.49)	1180.73(830.64-1654.31)	91.61(52.86-134.55)	14969.53(11888.52-18333.04)	30648.4(22193.27-41846.95)	119.28(76.2-168.68)
Sudan	Both	2613.86(2291.2-2991.78)	9390.06(8213.41-10628.94)	79.47(69.49-89.85)	143806.13(127570.43-163608.01)	349695.37(314574.01-388712.41)	21.52(16.37-27.17)	619.46(407.43-917.69)	1261.41(794.42-1951.8)	156.1(75.21-273.15)	15492.65(10739.98-22010.02)	32035.57(21409.74-47846.96)	198.76(108.1-325.49)
Sudan	Female	1318.97(1141.54-1525.99)	4113.02(3566.79-4632.49)	56.08(45.45-67.35)	75109.69(65570.21-86954.85)	171802.94(152094.56-193680.9)	14.06(8.09-20.31)	277.91(173.72-433.41)	558.89(343.92-817.97)	166.89(57.88-273.04)	7278.59(4840.57-10863.03)	14758.27(9737.53-21055.28)	193.67(78.55-312.36)
Sudan	Male	1294.89(1125.69-1497.22)	5277.04(4564.93-6100.78)	103.19(87.78-118)	68696.44(61057.1-77825.76)	177892.43(159210.98-198063.76)	29.69(23.46-36.41)	341.55(222.16-546.67)	702.52(402.74-1256.75)	148.37(69.11-321.89)	8214.06(5551.83-12417.92)	17277.3(10509.19-29046.07)	203.33(110.41-398.13)
Suriname	Both	54.56(45.07-67.69)	238.01(198.01-296.05)	186.63(156.82-216.66)	4180.42(3735.7-4709.88)	9742.11(8907.02-10594.42)	56.39(46.75-65.86)	18.94(15.08-23.08)	73.6(56.29-92.18)	139.85(111.26-172.79)	491.89(386.51-598.39)	1823.07(1401.6-2284.49)	173.93(143.65-214.61)
Suriname	Female	23.84(19.66-29.55)	109.85(89.74-136.02)	197.66(164.54-237.76)	2024(1788.04-2292.17)	4781.86(4327.59-5239.63)	55.47(44.82-66.13)	8.61(6.81-10.7)	35.04(26.72-44.34)	153.45(112.89-200.07)	221.63(174.01-271.87)	846.5(654.7-1071.27)	177.58(135.76-225.87)
Suriname	Male	30.71(24.85-38.83)	128.16(105.79-162.53)	178.96(141.22-224.81)	2156.42(1913.91-2429.29)	4960.25(4506.69-5431.02)	57.48(47.49-68.2)	10.33(8.11-12.7)	38.56(28.84-49.23)	128.7(96.56-169.02)	270.26(209.26-333.89)	976.57(731.73-1255.3)	171.01(134.28-221.23)
Sweden	Both	3567.26(3095.92-4082.73)	5046.57(4400.37-5793.74)	16.66(9.08-23.69)	145029.73(132118.27-158292.87)	196631.28(180380.84-213787.66)	13.34(9.89-16.46)	164.07(126.32-211.78)	519.85(382.76-672.04)	215.1(170.82-262.97)	3725.05(2977.65-4581.85)	7665.34(6086.1-9355.29)	106.75(82.34-133.98)
Sweden	Female	1987.71(1711.07-2286.55)	2441.95(2127.29-2779.64)	3.45(-3.8-10.8)	59948.98(54366.37-65682.33)	73953.05(67535.34-80555.71)	5.2(1.63-9.03)	77.21(59.33-97.14)	268.11(193.43-344.54)	234.22(182.37-293.57)	1800.04(1435.53-2219.36)	3660.25(2923.04-4424.25)	97.46(69.44-127.38)
Sweden	Male	1579.55(1363.15-1844.67)	2604.62(2209.74-3060.08)	32.96(18.36-47.36)	85080.75(76658.75-94094.29)	122678.22(110680.3-135145.43)	18.03(13.3-22.9)	86.87(64.86-113.87)	251.74(184.33-335.03)	197.53(151.66-246.54)	1925.01(1491.41-2416.93)	4005.1(3135.19-5041.8)	116.15(89.36-145.04)
Switzerland	Both	3244.9(2860.25-3628.02)	5856.24(5198.96-6506.38)	42.92(33.31-54.26)	99229.44(91074.53-107875.66)	153080.63(141049.55-165952.03)	20.07(15.77-24.72)	159.14(116.05-213.57)	529.27(357.97-739.82)	194.36(141.13-259.87)	3344.45(2620.49-4185.99)	7296.31(5588.47-9366.61)	106.99(78.49-137.6)
Switzerland	Female	1969.73(1722.41-2224)	3195.94(2803.04-3587.58)	28.8(17.9-41.74)	54968.61(49784.2-60345.2)	80018.88(73052.99-86991.99)	14.8(9.55-20.46)	82.77(59.4-112.8)	308.12(202.62-436.39)	211.2(147.2-290.87)	1757.68(1382.3-2196.15)	3963.9(3000.43-5151.53)	100.64(68.86-139.08)
Switzerland	Male	1275.16(1115.56-1442.3)	2660.31(2308.97-3013.88)	64.78(49.84-82.61)	44260.83(40010.96-48607.34)	73061.74(66396.84-79868.59)	26.69(20.82-33)	76.37(55.46-103.38)	221.15(154.3-311.95)	171.46(119.75-233.09)	1586.77(1215.49-2012.03)	3332.41(2542.57-4310.85)	112.3(82.03-147.68)
Syrian Arab Republic	Both	1925.19(1677.39-2206.52)	7753.17(6908.92-8735.13)	271.37(240.02-303.48)	90458.85(81078.88-102944.84)	215843.36(198537.3-236564.94)	109.24(93.05-124.32)	469.94(348.68-618.59)	784.86(548.92-1086.49)	45.27(12.67-72.48)	11780.47(8792.68-15198.74)	20562.22(14788.69-27887.24)	112.42(67.57-160.61)
Syrian Arab Republic	Female	1018.84(878.76-1179.17)	3787.36(3307.81-4305.24)	227.36(199.28-259.2)	46850.71(41438.81-53701.72)	110519.53(100861.09-122455.7)	97.08(81.32-112.65)	218.25(159.76-290.78)	387.79(264.67-540.95)	58.05(21.97-93.5)	5582.92(4117.87-7374.99)	10177.46(7237.52-13909.7)	112.17(65.24-167.96)
Syrian Arab Republic	Male	906.35(778.76-1048.6)	3965.81(3481.53-4554.81)	323.59(275-377.17)	43608.14(38981.8-49707.52)	105323.83(95921.37-115987.28)	122.81(102.88-141.79)	251.7(177.89-344.15)	397.08(272.86-550.09)	34.96(-5.29-71.87)	6197.55(4396.31-8349.41)	10384.76(7313.94-14121.53)	112.04(55.8-176.76)
Taiwan (Province of China)	Both	5993.45(5399.1-6615.37)	16915.36(15649.61-18294.23)	136.42(118.21-156.36)	351148.75(311998.66-387961.53)	712371.23(659162.74-773469.19)	72.43(60.03-84.68)	1249.39(1058.2-1441.97)	3097.95(2348.98-4028.17)	38.59(20.28-60.41)	31801.65(26546.3-36560.49)	76646.56(60534.74-95461.04)	79.94(61.23-102.81)
Taiwan (Province of China)	Female	2285.7(1982.72-2611.36)	8169.77(7426.67-8961.17)	190.3(157.89-225.31)	179297.97(156616.82-207609.11)	388794.97(355587.55-428055.09)	77.04(61.77-92.25)	633.42(529.85-744.63)	1647.78(1222.73-2141.08)	34.78(13.77-59.64)	15515.3(13091.56-17915.19)	38726.87(30694.27-47760.33)	75.37(53.55-100.23)
Taiwan (Province of China)	Male	3707.75(3332.16-4072.54)	8745.59(7963.2-9612.97)	103.74(87.78-123.87)	171850.78(153338.03-192615.17)	323576.26(296933.55-352867.48)	66.67(55.66-77.98)	615.97(516.53-722.04)	1450.17(1097.89-1900.92)	38.43(19.61-62.05)	16286.34(13451.45-18899.15)	37919.69(29556.85-47992.95)	82.22(61.43-104.99)
Tajikistan	Both	264.84(220.64-317.16)	888.32(737.29-1065.9)	93.46(75.78-137.66)	50741.46(43732.14-58692.83)	107234.86(93660.9-122169.4)	21.04(15-27.65)	22.68(17.38-28.46)	102.66(74.79-134.24)	281.96(235.07-339.49)	827.53(656.56-1019.4)	3467.63(2617.61-4401.78)	311.21(254.85-379.16)
Tajikistan	Female	143.04(118.59-170.05)	414.54(338.2-505.44)	67.66(49.05-104.12)	25528.58(21732.6-29784.9)	50892.62(44017.78-58187.33)	15.54(8.22-23.21)	11.15(8.47-14.05)	48.25(35.35-63.93)	285.65(227.81-349.84)	443.81(349.12-549.64)	1669.25(1239.55-2165.54)	278.48(216.57-352.27)
Tajikistan	Male	121.8(100.89-146.78)	473.78(391.27-573.61)	123.69(103.27-175.69)	25212.88(21593.54-29252.95)	56342.24(49010.44-64802.3)	26.44(19.56-33.97)	11.53(8.79-14.93)	54.41(38.44-72.98)	280.75(218.77-357.74)	383.72(302.41-478.62)	1798.38(1327.07-2342.99)	350.34(277.49-438.23)
Thailand	Both	9763.18(8530.42-11121.35)	42947.87(38488.14-47553.99)	271.35(238.23-309.3)	985604.51(867346.97-1132418.56)	2237295.76(2040178.94-2463077.22)	84.6(69.66-99.16)	3339.49(2714.66-3987.23)	10471.58(7593.64-13914.68)	91.36(60.94-124.83)	94340.65(74648.44-115586.91)	241434.23(179082.46-316908.06)	111.75(71.49-156.34)
Thailand	Female	5074.87(4402.34-5793.79)	21890.96(19517.85-24651.9)	264.12(226.43-308.83)	491626.06(430200.66-568882.46)	1112174.26(1006679.24-1231487.27)	80.77(64.17-96.85)	1468.52(1155.88-1790.55)	5843.87(4212.44-7813.52)	131.15(86.23-179.24)	40020.9(31455.97-48977.28)	127866.58(95604.3-166481.11)	156.21(105.32-214.47)
Thailand	Male	4688.32(3994.53-5479.76)	21056.91(18259.74-23841.57)	279.18(232.27-335.16)	493978.45(428131.1-564316.76)	1125121.5(1015859.54-1240403.86)	88.66(75.02-103.77)	1870.97(1455.81-2352.69)	4627.71(3250.89-6300.42)	56.53(24.83-97.08)	54319.75(41178.76-68975.04)	113567.65(82488.85-154009.65)	77.24(37.36-127.86)
Timor-Leste	Both	53.17(45.54-61.37)	223.11(193.37-253.3)	182.1(150.91-247.41)	8537.22(7318.39-9998.88)	18126.84(16185.79-20349.63)	26.96(18.4-37.08)	29.01(20.56-41.5)	87.4(63.35-115.29)	202.97(135.39-281.79)	891.26(612.78-1251.02)	2274.09(1630.5-3022.83)	280.55(183.57-399.19)
Timor-Leste	Female	27.7(23.62-32.09)	119.07(102.4-136.96)	188.21(153.58-257.65)	4092.28(3443.55-4861.06)	8702.6(7739.24-9805.6)	24.03(13.93-35.1)	16.12(11.51-21.73)	42.93(31.44-57.59)	190.22(117.76-285.77)	499.27(350.4-673.01)	1139.19(826.87-1526.77)	253.9(157.15-385.68)
Timor-Leste	Male	25.47(21.64-29.55)	104.04(88.95-120.12)	175.31(143-236.04)	4444.94(3794.51-5221.78)	9424.24(8324.14-10702.39)	29.87(21.18-39.78)	12.89(8.26-22.03)	44.47(29.68-65.95)	224.19(127-349.82)	391.99(249.52-665.14)	1134.9(743.64-1652.35)	317.42(183.2-502.54)
Togo	Both	209.49(182.65-237.72)	792.5(694.65-898.13)	81.8(70.79-94.04)	17790.39(15418.09-20486.75)	52549.71(46504.81-59242.18)	37.9(31.37-44.43)	86.96(62.08-116.5)	229.32(162.7-313.33)	93.1(53.69-141.69)	2181.33(1552.28-2904.76)	6038.79(4243.2-8320.97)	139.31(83.16-205.43)
Togo	Female	108.65(92.83-125.38)	438.08(380.62-498.87)	94.95(79.3-112.46)	9333.28(8034.49-10813.74)	28025.41(24706.14-31745.06)	40.15(31.57-48.4)	42.41(28.29-63.13)	112.31(77.37-161.71)	96.13(49.95-154.85)	1096.64(737.96-1613.43)	2888.78(1996.15-4076.31)	129.73(72.78-200.17)
Togo	Male	100.84(87.38-115.91)	354.42(307.35-406.08)	67.8(53.15-84.31)	8457.11(7277.73-9833.64)	24524.3(21267.21-27992.45)	35.4(28.78-42.63)	44.55(30.83-61.15)	117.01(81.1-161.38)	90.44(43.95-151.63)	1084.69(759.38-1498.06)	3150.01(2165.44-4443.89)	149.08(83.53-233.19)

Tokelau	Both	0.27(0.23-0.31)	0.43(0.38-0.5)	83.83(71.53-96.89)	25.25(22.21-28.42)	27.86(25.06-30.96)	32.13(26.43-38.18)	0.15(0.11-0.2)	0.18(0.13-0.25)	69.37(38.49-99)	3.76(2.8-5.08)	4.58(3.37-6.3)	82.99(52.94-115.69)
Tokelau	Female	0.13(0.11-0.15)	0.19(0.16-0.22)	69.85(54.75-85.44)	13.92(12.24-15.86)	14.02(12.52-15.7)	25.46(19.01-31.99)	0.1(0.07-0.14)	0.1(0.07-0.15)	54.49(21.31-94.25)	2.47(1.77-3.5)	2.66(1.88-3.9)	66.07(30.99-111.75)
Tokelau	Male	0.14(0.12-0.16)	0.24(0.21-0.28)	95.58(79.41-114.29)	11.32(9.98-12.73)	13.85(12.38-15.47)	40.51(34.09-47.77)	0.06(0.04-0.08)	0.08(0.06-0.11)	95.16(47.72-140.35)	1.29(0.95-1.75)	1.92(1.37-2.58)	116.83(68.82-166.77)
Tonga	Both	13.56(11.78-15.51)	25.61(22.63-28.72)	66.77(57.58-77.47)	1311.45(1156.42-1480.71)	1795.61(1616.96-1987.58)	30.26(24.8-36.74)	5.15(3.94-7.02)	11.54(8.76-15.8)	73.71(46.17-108)	144.13(109.47-195.85)	283.88(216.16-383.27)	89.64(61.87-123.79)
Tonga	Female	6.45(5.58-7.42)	11.57(10.1-13.04)	58.3(46.45-70.63)	701.47(610.82-800.17)	946.18(845.06-1053.43)	26.27(19.82-33.61)	2.76(1.97-4.08)	5.29(3.86-7.67)	44.23(17.97-83.52)	79.95(57.59-114.61)	130.49(95.44-181.74)	57.57(30.07-95.99)
Tonga	Male	7.11(6.11-8.25)	14.05(12.31-15.96)	74.48(61.57-90.34)	609.97(535.27-691.51)	849.42(759.92-950.14)	34.7(27.97-41.98)	2.39(1.82-3.19)	6.25(4.5-8.57)	107.36(59.05-155.77)	64.18(47.73-87.18)	153.39(111.36-210.85)	129.64(81.91-179.81)
Trinidad and Tobago	Both	201.43(175.83-227.86)	708.17(621.94-796.61)	197.76(169.13-230.23)	13594.67(12013.6-15278.78)	29017.2(26392.65-31666.24)	83.44(70.7-97.94)	44.63(35.21-53.89)	167.78(116.45-230.3)	165.44(134.41-199.14)	1173.17(936.12-1409.41)	4228.14(3039.26-5670.29)	193.69(154.87-230.35)
Trinidad and Tobago	Female	89.4(77.34-102.55)	312.32(270.57-357.55)	198.23(161.8-237.62)	6609.77(5790.67-7529.05)	13643.66(12363.66-15004.23)	77.8(64.34-93.62)	16.88(13.29-20.43)	70.7(49.06-97.49)	202.14(159.16-253.43)	473.07(374.73-570.24)	1803.74(1309.59-2420.03)	216.27(169.04-266.81)
Trinidad and Tobago	Male	112.02(96.37-127.24)	395.85(339.93-447.97)	196.81(165.09-235.59)	6984.89(6113.62-7925.51)	15373.54(13944.25-16926.78)	88.73(75.06-104.11)	27.75(21.73-33.72)	97.08(66.96-134.44)	142.8(113.88-176.05)	700.1(549.2-847.22)	2424.4(1710.5-3309.12)	178.14(141.45-213.92)
Tunisia	Both	1834.11(1600-2091.85)	8146.44(7190.68-9162.84)	224.62(202.61-246)	78813.47(70403.91-87856.85)	222151.83(202709.46-242331.81)	103.1(91.07-115.65)	267.92(195.45-357.15)	730.48(494.75-1038.1)	73.66(32.37-114.23)	6546.82(4908.72-8428.58)	17395.95(12579.43-23466.84)	147.6(95.44-203.16)
Tunisia	Female	940.34(814.02-1086.96)	3962.4(3453.2-4495.98)	202.08(178.12-230.17)	40753.07(35965.85-45647.99)	114888.95(104081.01-125926.79)	99.82(86.32-113.9)	125.7(90.35-179.21)	375.35(239.75-546.37)	83.14(33.2-137.66)	3167.12(2396.58-4298.16)	8741.54(6090.95-11935.55)	150.29(90.04-217.1)
Tunisia	Male	893.77(771.64-1031.43)	4184.05(3659.37-4791.72)	249.08(221.01-281.86)	38060.41(33744.03-42625.55)	107262.87(96953.44-117382.11)	106.53(93.74-120.71)	142.22(97.15-195.96)	355.12(228.81-535.59)	63.53(12.64-122.76)	3379.69(2455.12-4495.3)	8654.41(6044.82-12187.69)	144.44(77.88-218.33)
Turkey	Both	10737.42(9676.29-11988.49)	53090.27(47509.11-59413.18)	262.27(227.57-299.71)	536331.55(482840.9-600413.17)	1485243.07(1366010.18-1619651.59)	102.43(87.97-117.61)	3554.54(2646.99-5149.62)	6232.1(4736.43-8076.63)	50.71(3.66-90.5)	82136.09(62027.04-112711.85)	138083.02(107162.95-174807.67)	101.69(45.38-155.16)
Turkey	Female	5485.25(4892.15-6248.58)	25989.7(23045.1-29384.42)	245.12(209.92-283.44)	284974.75(254992.55-322653.36)	768234.29(701987.21-843158.37)	96.2(81.52-112.45)	1641.48(1209.14-2563.19)	3258.62(2389.89-4273.14)	60.81(0.19-107.39)	37691.53(28288.35-55498.96)	68866.91(53424.8-87516.22)	107.43(39.72-169.17)
Turkey	Male	5252.17(4677.05-5891.77)	27100.58(23693.7-30740.16)	280.37(237.26-329.04)	251356.8(224033.45-282463.02)	717008.78(651155.81-786123.5)	109.44(92.89-126.72)	1913.06(1319.73-2900.9)	2973.48(2198.69-3917.26)	40.15(-5.82-94.68)	44444.57(31488.48-62478.87)	69216.11(52079.41-88685.66)	96.34(37.83-166.33)
Turkmenistan	Both	265.98(227.63-313.44)	836.09(719.79-966.86)	131.68(111.87-155.69)	38346.44(33065.49-43828.01)	77565.58(68762.4-86708.39)	48.68(40-58.84)	69.36(52.43-85.06)	200.55(141.62-268.24)	135.98(106.28-172.98)	2254.92(1724.34-2776.88)	6465.07(4668.67-8574.4)	203.19(160.14-257.74)
Turkmenistan	Female	141.49(120.2-166.68)	398.45(339.53-464.53)	112.43(90.49-140.06)	19858.62(17139.65-22738.82)	37647.64(33361.33-42182.8)	43.68(34.6-54.44)	36.16(27.59-44.62)	85.37(61.15-114.57)	103.9(73.84-138.1)	1160.55(899.65-1422.39)	2676.45(1940.5-3531.85)	154.91(115.96-201.01)
Turkmenistan	Male	124.49(104.78-147.89)	437.64(374.12-516.97)	153.11(122.98-187.15)	18487.82(15785.83-21454.95)	39917.94(35015.69-45634.88)	53.81(43.34-65.43)	33.2(24.73-41.36)	115.18(80.64-155.51)	170.27(133.21-219.37)	1094.37(823.73-1369.09)	3788.62(2702.45-5090.01)	253.32(201.29-319.98)
Tuvalu	Both	1.36(1.18-1.58)	3.19(2.77-3.65)	77.96(67.19-90.74)	147.74(130.74-168.06)	225.02(202.36-250.59)	21.04(16.02-26.49)	0.83(0.61-1.12)	1.56(1.11-2.22)	89.02(54.46-127.75)	24.09(17.83-32.21)	42.66(30.23-59.39)	118.8(79.42-164.32)
Tuvalu	Female	0.68(0.58-0.8)	1.46(1.25-1.69)	77.09(62.91-92.45)	84.8(74.29-97.29)	114.22(101.51-127.01)	15.55(9.2-22.32)	0.47(0.33-0.67)	0.77(0.5-1.2)	69.91(25.41-124.3)	13.84(9.7-19.62)	21.03(14.24-32.26)	99.9(50.75-156.58)
Tuvalu	Male	0.68(0.58-0.8)	1.72(1.5-2)	77.67(61.25-94.57)	62.94(55.55-72.56)	110.79(99.01-125.35)	29.33(23.75-35.6)	0.36(0.26-0.48)	0.79(0.51-1.11)	114.75(59.51-169.1)	10.25(7.39-13.62)	21.63(14.51-30.32)	146.16(85.96-206.28)
Uganda	Both	716.62(634.16-803.07)	1906.23(1695.44-2153.49)	23.1(15.5-30.98)	91059.87(78683.99-105056.96)	207793.23(181958.23-238247.59)	-2.34(-6.43-1.81)	377.02(264.1-518.9)	808.76(582.42-1111.88)	157.33(102.17-223.17)	8708.63(6192.23-11841.48)	19074.67(13656.92-25939.52)	168.55(109.99-244.99)
Uganda	Female	298.99(260.75-342.62)	906.12(793.9-1031.07)	41.03(30.89-51.76)	45226.58(38337.87-52926.71)	106494.43(92758.22-122932.02)	0.28(-5.66-6.28)	138.01(91.87-214.59)	372.21(264.97-532.34)	237.57(151.59-356.13)	3160.02(2115.32-4847.67)	8355.65(5989.76-11875.96)	236.59(151.41-349.31)
Uganda	Male	417.64(367.36-472.8)	1000.11(882.52-1139.29)	10.17(0.92-20.07)	45833.29(39332.46-53103.99)	101298.8(86661.52-118096.94)	-4.92(-9.41-0.02)	239.02(163.6-329.93)	436.55(297.36-611.09)	111.62(53.54-179.66)	5548.61(3843.54-7433.34)	10719.02(7306.63-15093.86)	129.33(65.61-206.94)
Ukraine	Both	8122.22(6952.79-9523.36)	10343.27(9021.62-11775.39)	51.07(39.54-63.86)	1143240.19(1018659.14-1279135.5)	1129901.52(1022578.98-1246731.91)	17.44(12.85-22.18)	198.49(140.67-276.81)	322.73(223.43-452.93)	44.37(25.07-64.43)	7555.44(5719.06-9816.23)	11226.41(8411.32-14539.73)	48.81(31.74-67.08)
Ukraine	Female	5249.48(4438.98-6195.73)	5611.28(4798.33-6464.04)	25.67(11.1-42.16)	644035.2(570828.95-723499.7)	615171.63(550914.43-683954.6)	12.99(7.62-18.14)	120.16(85.13-167.98)	186.04(123.41-265.85)	48.4(25.94-73.25)	4489.56(3335.42-5839.26)	5868.52(4333.67-7774.6)	43.1(24.43-64.67)
Ukraine	Male	2872.75(2417.11-3358.04)	4731.99(4063.24-5445.21)	97.53(81.91-116.28)	499204.99(437226.24-568432.15)	514729.89(457579.88-576937.35)	23.18(17.39-29.38)	78.32(55.15-107.82)	136.69(93.85-194.02)	43.36(23.43-65.98)	3065.87(2301.99-3999.43)	5357.89(3928.04-7012.77)	62.62(41.88-88.67)
United Arab Emirates	Both	218.66(183.4-254.89)	3360.66(2798.3-3960.05)	224.18(199.18-253.67)	14617.48(12326.97-17309.95)	118153.93(104098.49-133190.23)	60.58(46.8-75.5)	55.74(37.15-74.61)	397.72(238.41-664.7)	41.05(3.11-98.74)	1757.13(1230.89-2351.67)	14788.93(9047.77-23940.73)	70.96(26.41-139.73)
United Arab Emirates	Female	77.24(65.62-89.99)	795.41(666.58-930.79)	175.59(148.65-202.1)	4650.34(3916.76-5503.85)	30349.93(26441.97-34699.56)	67.41(52.69-84.68)	20.14(12.12-28.81)	104.45(57.25-152.6)	77.04(6.85-148.76)	579.52(379.91-813.69)	3646.42(2197.54-5217.04)	83.14(10.86-159.67)
United Arab Emirates	Male	141.42(116.8-167.45)	2565.24(2106.7-3045.52)	238.94(207.1-275.92)	9967.14(8293.27-11850.39)	87804(77080.62-99254.2)	55.71(41.79-71.61)	35.6(22.24-50.67)	293.27(163.74-539.7)	38.11(-1.23-105.85)	1177.61(768.81-1681.1)	11142.5(6464.23-19860.62)	67.44(20.72-148.25)
United Kingdom	Both	25920.09(22848.58-29289.25)	37559.1(33251.96-41943.89)	26.22(23.71-28.61)	721758.7(669096.06-776010.73)	972389.3(901192.15-1043783.88)	15.34(13.54-17.28)	909.63(653.35-1247.82)	1348.66(959.53-1871.88)	52.05(32.36-75.44)	22310.26(17108.26-28458.81)	29494.89(22907.2-37611.86)	33.86(25.31-43.59)
United Kingdom	Female	14709.93(13022.31-16534.9)	18343.96(16174.46-20450.48)	11.12(8.05-14.03)	391702.93(363568.12-420436.61)	484429.33(447955.97-521024.18)	8.07(6.04-10.24)	543.11(392.05-751.5)	787.54(549.95-1108.78)	51.74(30.93-75.8)	12492.07(9682.21-15815.65)	15791.41(12305.27-19850.29)	27.44(18.49-37.95)
United Kingdom	Male	11210.16(9775.44-12660.75)	19215.14(17032.19-21624.86)	45.64(40.52-50.74)	330055.77(304423.1-355716.55)	487959.97(453211.95-524977.29)	23.76(21.34-26.31)	366.53(261.54-505.48)	561.12(397.22-778.92)	53.81(32.6-79.3)	9818.19(7397.37-12614.65)	13703.48(10463.86-17534.76)	41.93(31.75-53.67)
United Republic of Tanzania	Both	1410.03(1239.27-1584.49)	3932.9(3474.7-4387.63)	38.23(28.94-48.14)	136919.5(119874.22-156472.86)	339153.93(298915.04-383250.44)	16.03(11.16-20.88)	525.39(369.82-712.51)	1253.48(970.24-1589.52)	127.8(73.75-199.91)	12646.6(9096.79-17071.52)	29038.43(22553.58-36512.76)	143.09(84.57-216.02)

United Republic of Tanzania	Female	662.1(571.04-756.35)	1959.07(1705.26-2213.04)	47.72(34.37-61.59)	69549.5(60333.14-80244.13)	171607.81(149005.83-195354.06)	14.79(8.35-21.16)	237.56(166.11-336.81)	658.15(488.52-858.25)	167.52(95.59-262.71)	5810.89(4134.28-8151.96)	14854.64(11496.56-18753.86)	172.54(100.3-260.03)
United Republic of Tanzania	Male	747.93(654.62-846.06)	1973.83(1735.48-2238.26)	29.78(18.38-41.67)	67370(58099.68-77941.92)	167546.12(145881.69-191516)	17.35(11.79-23.22)	287.83(193.76-398.98)	595.33(436.5-789.88)	95.5(43.59-170.5)	6835.71(4760.28-9409.68)	14183.8(10596.74-18464.26)	118.25(59.57-196.12)
United States of America	Both	129417.99(115101.54-144637.3)	241349.84(217507.21-267986.55)	47.73(41.2-54.48)	3956111.53(3655349.54-4263369.15)	6881962.38(6367033.41-7384041.12)	34.07(30.66-37.77)	6245.69(4605.84-8256.04)	32584.29(25439.29-40313.07)	277.58(226.67-339.87)	173948.91(135311.24-214805.37)	677948.55(543301.67-807842.8)	193.37(164.16-225.81)
United States of America	Female	69586.26(62124.18-77581.76)	121736.95(110172.68-134527.42)	39.36(32.66-47.15)	2243148.54(2068973.18-2409427.87)	3731333.64(3452343.88-4001535.7)	28.54(24.81-32.59)	3883.83(2887.72-5124.09)	17292.49(13345.86-21855.45)	221.4(172.44-284.6)	100216.03(78852.75-122424.16)	343320.81(274747.27-407803.29)	152.28(125.9-184.38)
United States of America	Male	59831.73(52688.38-68136.15)	119612.89(106893.94-133740.56)	57.35(50.25-64.4)	1712962.99(1577273-1854710.94)	3150628.73(2908019.9-3395946.9)	41.35(37.27-45.36)	2361.85(1676.66-3214.24)	15291.81(11635.25-19278.15)	369.72(296.35-454.13)	73732.88(55831.69-93240.07)	334627.74(261076.74-403036.83)	248.62(207.55-295.56)
United States Virgin Islands	Both	22.16(19.45-24.86)	72.33(63.06-81.66)	209.93(183.81-238.86)	1359.96(1220.43-1525.22)	2662.27(2435.34-2897.23)	97.12(82.01-111.33)	4.48(3.46-5.68)	15.55(11.92-19.24)	75.76(48.83-107.63)	125.91(97.71-158.97)	372.68(290.19-457.85)	122.67(91.42-160.1)
United States Virgin Islands	Female	11.09(9.66-12.59)	37.09(31.85-42.38)	212.13(179.05-247.26)	693.63(609.03-781.23)	1366.78(1241.09-1502.61)	94.79(78.65-110.63)	2.21(1.64-2.92)	7(5.28-8.77)	74.53(37.97-116.65)	60.37(46.41-76.29)	161.68(124.21-202.24)	111.79(72.8-155.29)
United States Virgin Islands	Male	11.08(9.56-12.77)	35.24(30.4-40.43)	208.24(175.05-244.8)	666.33(595.9-750.08)	1295.49(1179.88-1423.49)	99.82(85.68-114.59)	2.27(1.71-2.93)	8.55(6.34-10.81)	79.24(42.34-122.58)	65.55(49.53-83.36)	211.01(159.79-265.2)	133.15(88.74-184.08)
Uruguay	Both	929.41(826.38-1044.91)	1655.99(1498.02-1841.54)	63.51(49.98-78.54)	29760.07(26870.75-33022.56)	51163.79(47040.73-55346.8)	56.47(47.67-66.39)	128.81(113.88-144)	283.7(227.02-351.44)	92.78(59.25-129.08)	2719.07(2416.3-3041.19)	4952.69(4166.37-5815.31)	82.45(57.73-110.28)
Uruguay	Female	473.73(413.47-544.64)	847.98(748.73-957.6)	61.64(43.43-81.11)	15845.31(14071.14-17681.17)	27276.25(24760.38-29927.18)	54.6(44.23-66.54)	55.63(48.63-62.91)	145.19(113.9-182.25)	106.67(68.49-149.92)	1148.44(1015.12-1293.62)	2343.84(1950.92-2772.47)	92.29(64.14-122.61)
Uruguay	Male	455.67(399.59-520.27)	808(722.07-909.52)	65.66(45.86-87.9)	13914.76(12395.28-15535.94)	23887.54(21902.01-25944.21)	58.59(48.45-70.92)	73.18(64.78-82.31)	138.51(110.91-171)	81.44(47.76-118.3)	1570.63(1382.09-1764.42)	2608.84(2175.99-3102.69)	75.72(49.21-103.63)
Uzbekistan	Both	1943.59(1645.1-2280.33)	6081.07(5063.94-7213.55)	92.18(78.95-105.48)	218161.59(189701.39-249360.6)	471472.87(415572.27-533090.23)	35.43(28.58-42.63)	463.58(323.52-707.5)	1094.02(807.59-1408.65)	51.56(3.64-86.67)	14449.27(10578.14-20561.69)	37160.83(28077.24-47367.1)	97.29(45.72-139.17)
Uzbekistan	Female	1106.76(938.67-1300.54)	2982.69(2458.49-3547.78)	65.57(52.19-79.81)	113220.92(98338.3-130369.73)	233180.19(204811.82-262728.68)	29.55(21.54-37.57)	248.59(162.93-454.27)	512.54(380.87-656.82)	38.2(-19.41-84.83)	7354.96(5232.94-11885.99)	17140.06(13030.22-21898.76)	79.84(16.23-130.36)
Uzbekistan	Male	836.84(689.92-1003.25)	3098.38(2548.6-3691.2)	127.37(108.62-146.48)	104940.67(90352.28-121640.01)	238292.68(207185.18-272404.67)	41.73(33.82-49.99)	215(151.97-291.63)	581.48(421.96-753.02)	67.56(33.56-97.53)	7094.31(5183.88-9571.51)	20020.77(14856.27-25618.71)	115.43(72.33-155.71)
Vanuatu	Both	12.12(10.44-14.19)	45.61(39.77-52.7)	110.55(97.64-123.82)	1750.86(1507.82-2068)	4185.42(3694.73-4782.87)	24.32(17.76-31.34)	4.97(3.29-7.25)	22.49(15.59-31.06)	124.15(79.84-192.63)	152.93(103.81-217.6)	659.08(455.76-914.37)	147.57(98.16-220.06)
Vanuatu	Female	5.09(4.29-6.09)	19.34(16.54-22.67)	112.56(95.42-131.4)	868.05(733.34-1052.09)	2075.75(1819.16-2402.84)	21.78(13.62-30.74)	2.42(1.45-3.88)	10.86(7.13-16.18)	113.32(56.98-218.12)	77.03(49.54-117.99)	326.38(214.96-484.34)	138.54(74.43-242.22)
Vanuatu	Male	7.04(6.01-8.17)	26.27(22.65-30.6)	109.16(92.98-126.81)	882.81(759.16-1031.3)	2109.66(1860.74-2412.33)	26.83(20.15-33.56)	2.55(1.71-3.88)	11.63(8.3-16.11)	131.92(70.08-204.21)	75.9(50.65-112.11)	332.7(238.78-461.52)	155.53(93.34-222.13)
Venezuela (Bolivarian Republic of)	Both	3272.36(2840.55-3755.19)	14690.38(13043.37-16407.39)	209.6(176.83-243.86)	229096.37(202032.9-262037.31)	611248.59(560166.55-668784.58)	79.06(66.28-92.55)	415.87(331.79-502.78)	3140.25(2194.46-4296.55)	258.64(216.79-303.19)	12453.16(10022.48-14867.86)	79061.5(56930.8-105495.67)	311.43(254.25-369.01)
Venezuela (Bolivarian Republic of)	Female	1539.1(1330.7-1778.56)	6840.75(6011.62-7694.77)	202.48(163.6-245.83)	112782.86(97744.73-128944.69)	291780.39(262553.92-321109.43)	70.68(56.73-85.49)	189.88(150.4-228.88)	1419.74(991.92-1932.43)	260.63(201.37-318.72)	5639.02(4574.06-6753.19)	34485.17(24987.15-45898.99)	301.41(231.5-368.43)
Venezuela (Bolivarian Republic of)	Male	1733.25(1494.34-2012.34)	7849.64(6755.74-8992.44)	216.59(179.9-257.55)	116313.51(102729.44-133396.56)	319468.2(289543.98-351291.91)	87.67(73.82-103.12)	225.99(179.81-274.27)	1720.51(1198.81-2389.35)	257.51(212.22-309.23)	6814.14(5480.49-8206.08)	44576.34(31832.18-60514.82)	319.78(267.82-379.75)
Viet Nam	Both	6739.78(5889.36-7676.61)	24354.52(21277.45-27892.83)	169.84(149.51-194.55)	871053.22(758535.59-1002467.39)	1983745.78(1760962.64-2217230.05)	63.81(54.61-73.6)	3939.41(3034.45-5035.85)	8087.68(6012.15-10441.32)	40.52(-4.1-72.7)	93396.38(70632-120241.37)	191203.02(142409.93-251491.23)	77.61(27.82-119.89)
Viet Nam	Female	4125.67(3594.58-4765.13)	14313.09(12419.41-16403.97)	166.93(140.78-196.17)	471981.3(406431.23-544722.79)	1025653.8(903568.38-1147507.61)	58.52(48.19-69.31)	2636.65(1958.02-3426.73)	4969.87(3573.24-6515.09)	37.87(-7.06-75.89)	60458.26(45080.26-78768.47)	110127.24(82187.72-144843.3)	65.36(20.26-114.53)
Viet Nam	Male	2614.11(2265.98-3005.63)	10041.43(8574.09-11665.32)	178.33(152.73-208.64)	399071.92(342882.71-463053.37)	958091.98(832194.48-1082694.53)	70.14(59.74-81.78)	1302.76(927.52-1908.68)	3117.8(2222.46-4110.98)	55.38(-9.46-116.77)	32938.12(23396.35-47295.37)	81075.78(56367.13-109706.88)	106.31(25.74-189.21)
Yemen	Both	1322.06(1149.48-1523.51)	6073.69(5244.06-7032.92)	110.43(94.25-132.62)	74916.67(66279.63-84914.42)	231880(207359.28-256984.76)	36(29.51-43.19)	297.58(198.49-447.86)	752.39(515.06-1052.16)	113.55(67.23-171.72)	7994.45(5411.06-11724.23)	20432.61(14405.92-27820.69)	145.94(88.22-219.73)
Yemen	Female	708.29(613.4-828.96)	2984.28(2501.63-3589.54)	92.28(70.11-126.96)	40744.27(35637.65-46689.01)	120620.81(106673.72-135539.01)	28.27(19.92-38.04)	142.59(90.79-241.06)	351.25(238.36-508.35)	123.58(64.18-200.15)	3818.63(2559.36-6196.84)	9907.01(6981.5-13927.6)	154.23(83.92-240.63)
Yemen	Male	613.77(524.84-717.22)	3089.41(2631.76-3572.14)	131.43(110.9-153.96)	34172.41(30159.61-38708.66)	111259.19(99020.23-124779.01)	45.07(37.41-52.76)	154.99(100.54-231.97)	401.13(267.67-574.05)	105.91(55.46-175.75)	4175.82(2769.71-6247.22)	10525.6(7231.83-14651.53)	138.62(77-225.53)
Zambia	Both	389.59(347.12-435.45)	1161.77(1019.28-1295.86)	47.99(37.42-59.07)	40588.45(35262.25-46617.42)	103421.24(90710.44-117429.3)	12.62(7.79-17.54)	197.11(140.33-258.25)	449.82(328.26-607.94)	110.84(57.03-172.26)	4911.4(3520.61-6368.39)	11500.37(8296.24-15501.86)	142.16(79.37-215.1)
Zambia	Female	167.48(145.33-192.68)	555.96(482.3-633.46)	68.57(53.15-85.96)	19927.99(17161.92-23268.41)	50156.13(44058.41-57427.27)	11.65(4.5-18.91)	87.33(61.55-113.76)	211.22(150.18-294.61)	142.42(76.76-223.67)	2321.69(1627.72-3009.91)	5351.59(3865.76-7432.12)	154.16(84.25-248.64)
Zambia	Male	222.11(196.72-248.45)	605.81(528.35-685.11)	32.11(21.17-44.79)	20660.46(17763.62-23819.29)	53265.11(45750.52-61644.72)	13.53(8.33-19.72)	109.78(73.56-152.08)	238.61(165.64-324.89)	87.91(24.32-160.21)	2589.71(1766.46-3588.08)	6148.78(4361.29-8413.72)	132.17(58.19-222.92)
Zimbabwe	Both	872.17(758.51-1013.94)	1723.87(1507.05-1994.59)	34.63(27.71-41.86)	62390.83(54197.53-71236.88)	112269.6(98937.07-126042.93)	27.21(22.04-32.68)	166.79(107.28-266.45)	471.27(305.44-758.61)	75.34(40.19-120.61)	4351.55(2901.85-6710.91)	12361.75(8066.67-19260.88)	101.6(58.54-152.87)
Zimbabwe	Female	360.7(308.5-420.78)	847.94(731.04-985.4)	57.62(46.59-67.78)	31073.67(26572.8-35894.6)	59585.65(52230.22-67098.86)	31.7(24.33-39.81)	66.47(38.26-134.99)	212.3(118.04-433.7)	87.44(44.51-150.67)	1686.35(1038.26-3129.74)	5419.55(3197.29-10510.62)	122.06(72.1-188)
Zimbabwe	Male	511.47(440.47-602.01)	875.93(761.67-1014)	18.61(9.85-27.96)	31317.16(27247.62-35919.68)	52683.96(46172-59904)	22.75(17.52-28.7)	100.32(64.27-163.2)	258.97(167.59-382.05)	67.91(25.61-123.69)	2665.2(1758.2-4287.14)	6942.2(4591.88-10098.21)	89.35(40.49-148.75)

Abbreviation: DALY, disability adjusted life-year; UI, uncertainty interval.

Table S6. The age-standardized rates and their EAPCs of type 2 diabetes related CKD in 204 countries and territories.

Location	Sex	ASIR (95% UI)		EAPC (95% CI)	ASPR (95% UI)		EAPC (95% CI)	ASDR (95% UI)		EAPC (95% CI)	Age-standardized DALYs Rate (95% UI)		EAPC (95% CI)
		1990	2019		1990	2019		1990			1990	2019	
Afghanistan	Both	32.04(28.13-36.52)	51.7(45.52-58.89)	1.76(1.67-1.86)	1365.98(1244.02-1506.55)	1608.28(1474.14-1743.56)	0.56(0.5-0.62)	17.57(12.06-27.88)	16.09(11.2-25.49)	-0.19(-0.25--0.13)	388.72(269.17-566.22)	351.65(249.82-512.93)	-0.25(-0.3--0.2)
Afghanistan	Female	33.79(29.24-39.03)	49.65(43-56.79)	1.57(1.51-1.63)	1454.41(1310-1620.84)	1650.36(1492.58-1812.84)	0.51(0.48-0.54)	16.73(11.21-29.26)	16.07(10.52-27.75)	0.04(-0.07-0.16)	394.38(270.45-613.41)	368.42(250.4-573.59)	-0.08(-0.17-0.01)
Afghanistan	Male	30.37(26.8-34.4)	54.04(47.48-61.22)	1.94(1.74-2.14)	1266.52(1146.39-1400.36)	1571.68(1431.52-1719.26)	0.66(0.55-0.77)	18.26(12.21-28.7)	16.02(10.25-25.95)	-0.38(-0.43--0.33)	379.04(253.53-568.38)	333.81(222.64-504.58)	-0.39(-0.45--0.34)
Albania	Both	16.09(14.08-18.41)	25.77(22.49-29.09)	1.63(1.59-1.68)	1157.16(1042.21-1277.97)	1265.96(1149.84-1383.1)	0.27(0.26-0.29)	1.47(1.02-2.01)	1.09(0.68-1.62)	-1.49(-1.84--1.14)	38.95(28.42-50.57)	33.71(24.75-45.99)	-0.74(-0.97--0.51)
Albania	Female	14.57(12.59-16.86)	22.78(19.78-26.15)	1.53(1.48-1.58)	1127.34(996.22-1258.99)	1217.58(1090.76-1347.77)	0.24(0.23-0.26)	1.28(0.88-1.78)	1.02(0.64-1.55)	-1.57(-1.95--1.18)	35.8(25.82-47.19)	32.17(23.57-43.39)	-0.86(-1.12--0.6)
Albania	Male	17.92(15.46-20.85)	29.11(25.11-33.29)	1.69(1.63-1.75)	1193.32(1072.85-1322.51)	1320.04(1195.92-1451.61)	0.3(0.28-0.31)	1.79(1.22-2.48)	1.2(0.73-1.83)	-1.58(-1.91--1.25)	43.5(31.44-57.19)	35.67(25.38-48.68)	-0.72(-0.94--0.49)
Algeria	Both	36.45(32.3-41.18)	62.55(55.17-70.39)	1.88(1.76-2)	1436.14(1308.13-1575.87)	1778.36(1627.06-1940.79)	0.71(0.68-0.74)	11.52(8.3-16.95)	9.61(6.81-13.22)	-0.4(-0.61--0.19)	218.13(158.06-310.66)	187.54(138.83-254.08)	-0.38(-0.55--0.2)
Algeria	Female	39.4(34.43-45.38)	60.78(53.5-68.34)	1.63(1.42-1.85)	1533.68(1377.18-1705.67)	1813.22(1646.38-1984.1)	0.64(0.57-0.71)	11.99(7.84-20.94)	11.27(7.72-17.12)	0.19(-0.12-0.5)	230.96(158.25-379.8)	211.61(151.38-307.1)	-0.06(-0.28-0.17)
Algeria	Male	33.26(28.95-37.93)	64.21(55.8-73.38)	2.18(2.11-2.24)	1338.12(1209.09-1468.69)	1741.23(1580.73-1909.05)	0.8(0.74-0.86)	11.13(8.17-15.25)	8.36(5.81-11.82)	-0.88(-1.01--0.75)	205.92(150.62-282)	166.88(120.43-232.71)	-0.66(-0.8--0.53)
American Samoa	Both	28.3(24.79-32.2)	40.02(35.36-44.87)	1.11(0.98-1.23)	2133.37(1908.41-2389.98)	2283.35(2066.12-2526.52)	0.15(0.12-0.19)	14.28(11.48-17.48)	20.83(16.65-25.42)	1.38(1.24-1.53)	324.64(261.79-400.63)	478.35(379.02-589.82)	1.41(1.27-1.54)
American Samoa	Female	23.01(19.58-26.9)	34.17(29.7-39.18)	1.26(1.13-1.39)	2153.32(1907.38-2453.75)	2276.05(2044.41-2545.61)	0.13(0.1-0.16)	11(8.65-14.15)	18.06(13.8-23.53)	1.7(1.41-1.99)	260.4(204.77-328.76)	429(331.72-555.93)	1.7(1.43-1.97)
American Samoa	Male	33.88(29.7-38.46)	46.44(40.7-52.39)	1(0.87-1.13)	2120.39(1880.71-2372.39)	2296.22(2066.82-2540.86)	0.17(0.12-0.22)	17.93(13.7-22.64)	24.26(19.56-29.3)	1.19(1.11-1.27)	390.41(304.81-493.17)	534.24(421.88-654.81)	1.21(1.14-1.29)
Andorra	Both	29.93(26.45-33.73)	30.83(27.05-34.65)	0.05(-0.08-0.18)	921(836.4-1010.96)	906.47(822.48-991.08)	-0.13(-0.17--0.09)	2.09(1.39-3.19)	1.84(1.19-2.77)	-0.37(-0.59--0.16)	38.57(28.09-53.18)	34.08(24.74-46.33)	-0.43(-0.56--0.29)
Andorra	Female	30.17(26.24-34.26)	30.98(27.01-34.9)	0.03(-0.09-0.16)	879.66(794.06-976.66)	866.08(779.19-953.25)	-0.11(-0.14--0.08)	1.96(1.23-3.08)	1.87(1.14-2.9)	-0.07(-0.39-0.25)	36.91(26.2-52.83)	35.1(24.69-48.81)	-0.18(-0.39-0.03)
Andorra	Male	29.89(25.94-34.23)	30.89(26.83-35.27)	0.07(-0.07-0.2)	959.85(871.19-1057.95)	944.7(852.4-1040.04)	-0.14(-0.17--0.1)	2.29(1.52-3.47)	1.83(1.2-2.74)	-0.7(-0.81--0.59)	40.64(29.2-56.83)	33.3(24.18-44.63)	-0.68(-0.74--0.62)
Angola	Both	11.12(9.96-12.39)	15.99(14.22-18.07)	1.32(1.2-1.44)	1069.94(958.43-1200.74)	1119.96(1006.44-1238.58)	0.12(0.07-0.16)	6.43(4.31-8.61)	6.42(4.12-8.85)	-0.07(-0.12--0.02)	135.93(91.28-181.22)	128.66(86.42-175.66)	-0.27(-0.31--0.22)
Angola	Female	9.25(8.08-10.52)	14.36(12.55-16.48)	1.6(1.45-1.75)	1040.8(920.5-1178.58)	1074.69(958.24-1199.66)	0.1(0.06-0.15)	4.27(1.57-6.71)	4.84(1.99-7.25)	0.31(0.22-0.4)	92.83(38.6-143.66)	97.28(44.6-144.15)	0.04(-0.04-0.12)
Angola	Male	13.02(11.65-14.52)	17.95(15.67-20.4)	1.17(1.08-1.27)	1099.27(978.01-1236.84)	1173.11(1052.69-1313.3)	0.15(0.11-0.2)	8.86(5.87-12.11)	8.58(6.07-11.82)	-0.14(-0.19--0.1)	181.77(123.17-244.54)	169.08(120.68-225.67)	-0.3(-0.35--0.25)
Antigua	Both	27.45(24.32-30.84)	41.84(36.79-46.66)	1.43(1.3-1.57)	1496.04(1345.5-1660.01)	1783.19(1602.68-1968.97)	0.62(0.58-0.66)	7.47(5.97-9.04)	11.33(8.78-14.12)	1.75(1.52-1.99)	178.32(142.46-215.83)	247.76(194.52-304.71)	1.37(1.19-1.55)
Antigua	Female	24.37(21.19-27.72)	38.1(33.15-42.93)	1.53(1.41-1.66)	1387.31(1232.16-1554.35)	1649.55(1475.51-1840.89)	0.66(0.62-0.7)	5.03(3.96-6.22)	10.27(7.91-12.76)	2.65(2.51-2.79)	126.33(101.03-156.22)	229.84(180.13-285.4)	2.22(2.11-2.33)
Antigua	Male	31.08(27.05-35.36)	45.94(40.39-52.08)	1.31(1.16-1.46)	1629.14(1466.62-1819.4)	1930.08(1731.89-2148.02)	0.55(0.51-0.6)	11.21(8.97-13.74)	12.86(9.83-16.32)	0.91(0.47-1.34)	246.96(195.21-301.08)	270.46(209.83-335.59)	0.61(0.31-0.91)
Argentina	Both	28.76(25.43-32.02)	36.97(33.23-41.07)	0.81(0.71-0.92)	1085.65(983.52-1183.32)	1213.12(1109.67-1320.85)	0.37(0.33-0.4)	6.75(5.27-8.36)	8.35(6.48-10.45)	0.66(0.31-1)	142.57(112.64-171.54)	161.92(126.91-196.85)	0.37(0.08-0.66)
Argentina	Female	26.16(22.88-29.51)	33.92(29.93-38.7)	0.82(0.7-0.94)	1066.95(958.51-1182.12)	1184.26(1070.27-1304.29)	0.33(0.29-0.37)	5.31(4.09-6.65)	6.83(5.23-8.68)	0.82(0.43-1.21)	112.23(88.11-135.56)	131.86(103.07-162.73)	0.53(0.21-0.85)
Argentina	Male	32.18(28.1-36.32)	41.07(36.43-46.77)	0.81(0.71-0.9)	1118.29(1009.76-1223.97)	1256.91(1143.77-1377.26)	0.4(0.37-0.44)	8.77(6.79-10.93)	10.56(8.11-13.33)	0.55(0.24-0.87)	182(142.14-219.85)	201.59(157.67-247.82)	0.26(-0.02-0.54)
Armenia	Both	11.5(9.87-13.41)	21.95(19.12-25.09)	2.42(2.3-2.53)	1565.04(1382.05-1752.25)	1692.03(1512.94-1862.16)	0.22(0.2-0.25)	0.94(0.73-1.2)	3.86(2.95-4.96)	5.01(4.66-5.37)	25.7(20.77-30.89)	86.64(67.88-107.23)	4.26(3.93-4.59)
Armenia	Female	11.41(9.68-13.46)	19.5(16.81-22.41)	1.97(1.82-2.11)	1500.99(1309.99-1695.76)	1581.46(1400.62-1754.8)	0.14(0.11-0.16)	0.72(0.56-0.9)	3.03(2.31-3.88)	4.99(4.47-5.51)	21.4(17.29-26.16)	65.29(51.43-81.04)	3.9(3.49-4.31)
Armenia	Male	11.71(10.01-13.72)	25.25(21.76-29.25)	2.92(2.82-3.02)	1644.35(1448.54-1851.65)	1831.01(1641.63-2019.94)	0.33(0.3-0.35)	1.29(0.98-1.67)	5.05(3.8-6.45)	4.93(4.65-5.21)	32.06(25.85-38.71)	114.82(88.15-143.26)	4.49(4.23-4.75)
Australia	Both	34.2(31.8-36.89)	38.62(34.72-42.61)	0.33(0.26-0.4)	988.65(912.89-1062.18)	1010.27(932.86-1095.53)	0.04(0-0.09)	0.5(0.36-0.7)	0.94(0.64-1.38)	2.94(2.54-3.35)	15.55(12.29-19.62)	23.43(17.6-30.15)	1.71(1.48-1.94)
Australia	Female	34.34(31.58-37.33)	37.26(33.38-41.47)	0.15(0.07-0.22)	1020.86(934.13-1113.47)	1017.01(929.32-1108.57)	-0.03(-0.09-0.02)	0.45(0.32-0.63)	0.81(0.54-1.19)	2.89(2.61-3.17)	15.59(11.95-19.89)	21.27(16.06-27.71)	1.34(1.24-1.45)
Australia	Male	35.27(31.73-39.19)	40.36(35.21-45.64)	0.4(0.31-0.5)	963.36(885.03-1052.65)	1007.54(916.98-1103.74)	0.11(0.08-0.14)	0.62(0.44-0.86)	1.1(0.73-1.61)	2.64(2.04-3.24)	16.18(12.69-20.56)	26.03(19.27-34.1)	1.93(1.55-2.31)
Austria	Both	28.82(24.99-32.72)	36.27(32-40.42)	0.68(0.6-0.75)	916.41(826.62-1003.11)	970.64(889.42-1059.86)	0.1(0.05-0.15)	1.28(0.93-1.72)	3.38(2.39-4.59)	4.6(3.93-5.26)	30.06(23.09-37.99)	54.35(41.88-69.23)	2.9(2.35-3.45)
Austria	Female	28.83(24.98-32.92)	35.35(30.9-39.77)	0.58(0.51-0.66)	877.67(788.11-970.21)	911.06(827.1-1003.32)	0.04(0.01-0.08)	1.2(0.87-1.6)	3.26(2.23-4.47)	4.75(4.12-5.39)	28.25(21.79-35.95)	50.71(39.25-65.17)	2.84(2.37-3.31)
Austria	Male	28.8(24.82-33.11)	37.49(32.65-42.5)	0.81(0.72-0.89)	968.72(876.95-1065.88)	1037.39(944.94-1136.16)	0.13(0.07-0.2)	1.42(1.02-1.92)	3.52(2.51-4.83)	4.17(3.5-4.84)	32.65(24.58-41.22)	58.46(44.14-74.91)	2.83(2.22-3.45)
Azerbaijan	Both	12.69(10.94-14.85)	23.48(20.55-26.73)	2.2(1.97-2.44)	1569.49(1402.05-1759.08)	1730.31(1553.27-1919.37)	0.29(0.24-0.34)	2.61(2-3.31)	5.7(4.27-7.27)	3.47(2.94-4.01)	74.76(58.18-92.26)	131.85(103.62-164.83)	2.44(2.02-2.86)
Azerbaijan	Female	12.63(10.74-14.82)	21.79(18.85-25.16)	1.89(1.65-2.12)	1506.46(1323.16-1705.44)	1635.05(1449.15-1831.15)	0.24(0.19-0.29)	2.16(1.62-2.9)	5.3(3.87-6.9)	3.8(3.29-4.32)	62.38(48.51-78.96)	119.32(90.72-152.06)	2.66(2.27-3.06)
Azerbaijan	Male	12.84(10.95-15.02)	25.71(22.21-29.55)	2.58(2.34-2.81)	1649.19(1454.5-1839.91)	1841.61(1647.91-2040.95)	0.33(0.28-0.38)	3.27(2.4-4.17)	6.2(4.34-8.52)	3.05(2.52-3.59)	91.48(67.97-115.04)	147.12(108.56-192.42)	2.2(1.76-2.64)

Bahamas	Both	24.59(21.6-27.69)	35.19(31.35-39.56)	1.15(0.99-1.31)	1454.35(1315.07-1610.46)	1588.01(1449.04-1734.06)	0.24(0.19-0.3)	6.71(5.33-8.16)	8.29(6.27-10.48)	1.13(0.95-1.31)	169.43(134.72-203.34)	204.93(157.85-256.19)	0.95(0.82-1.07)
Bahamas	Female	22.07(19.28-25.19)	32.22(28.17-36.58)	1.2(1.05-1.36)	1382.96(1243.02-1556.61)	1487.92(1343.89-1638.8)	0.21(0.17-0.26)	5.53(4.32-6.82)	7.26(5.5-9.33)	1.46(1.22-1.71)	143.37(113.11-174.98)	181.27(138.74-227.55)	1.19(1-1.37)
Bahamas	Male	27.66(24.03-31.76)	38.63(33.74-44)	1.08(0.91-1.24)	1543.84(1394.95-1708.88)	1705.27(1548.41-1868.83)	0.26(0.2-0.33)	8.56(6.79-10.51)	9.73(7.24-12.33)	0.69(0.53-0.85)	204.31(161.46-246.74)	234.06(178.34-292.78)	0.67(0.57-0.76)
Bahrain	Both	43.6(39.02-49.09)	74.22(66.4-82.63)	1.92(1.76-2.07)	1538.83(1406.74-1686.81)	1951.23(1784.22-2133.28)	0.85(0.78-0.91)	13.07(10.04-16.86)	11.92(8.88-15.35)	-0.13(-0.71-0.47)	246.93(193.56-311.21)	219.49(171.24-276.69)	-0.38(-0.87-0.12)
Bahrain	Female	46.35(41.05-52.19)	67.93(60.81-75.54)	1.54(1.29-1.78)	1650.47(1498.08-1829.88)	1943.56(1770.74-2121.87)	0.68(0.58-0.79)	13.15(9.59-17.11)	11.83(8.61-15.31)	-0.32(-0.81-0.17)	259.98(195.75-331.12)	219.29(167.01-277.38)	-0.7(-1.1--0.29)
Bahrain	Male	41.74(36.49-47.44)	78.91(69.51-88.54)	2.26(2.15-2.36)	1466.56(1330.91-1610.59)	1986.87(1803.92-2179.55)	1.03(1-1.05)	13.31(9.79-18.59)	12.14(8.84-16.48)	0.01(-0.7-0.71)	240.02(181.66-324.26)	222.47(168.26-286.36)	-0.1(-0.7-0.5)
Bangladesh	Both	12.09(10.5-13.81)	18.42(15.92-21.01)	1.41(1.23-1.59)	1244.99(1110.18-1399.53)	1307.12(1178.2-1441.31)	0.06(0.02-0.11)	3.39(2.37-4.54)	2.94(2.12-4)	-0.3(-0.66-0.06)	79.07(57.55-103.54)	71.09(52.82-93.88)	0.04(-0.22-0.3)
Bangladesh	Female	10.4(9.11-11.73)	16.21(14.09-18.49)	1.48(1.35-1.6)	1089.67(964.72-1235.81)	1134.47(1018.05-1261.63)	0.06(0.01-0.11)	3.05(1.79-4.32)	2.98(2.05-4.14)	0.02(-0.33-0.37)	60.69(38.65-82.84)	60.18(44.8-79.63)	0.28(0.06-0.51)
Bangladesh	Male	13.49(11.38-15.7)	20.53(17.28-23.96)	1.42(1.19-1.65)	1377.06(1222.99-1556.27)	1476.9(1318.35-1648.61)	0.12(0.07-0.17)	3.66(2.43-5)	2.93(2-4.11)	-0.49(-0.9--0.08)	94.22(64.87-126.73)	81.72(58.61-109.5)	-0.02(-0.3-0.27)
Barbados	Both	24.05(21.09-27.21)	36.3(32.27-41.44)	1.34(1.21-1.48)	1451.31(1313.07-1608.12)	1605.56(1467.33-1758.62)	0.29(0.25-0.32)	5.24(4.22-6.29)	7.4(5.73-9.29)	1.18(0.95-1.42)	127.15(103.68-150.63)	174.4(137.81-214.82)	1.07(0.88-1.26)
Barbados	Female	21.99(19.16-25.16)	33.69(29.76-38.28)	1.43(1.29-1.56)	1379.77(1229.15-1544.8)	1509.72(1364.86-1656.13)	0.28(0.25-0.31)	4.27(3.45-5.16)	6.91(5.34-8.63)	2.08(1.73-2.43)	106.48(86.89-127.12)	164.84(129.43-204.92)	1.84(1.56-2.11)
Barbados	Male	26.64(22.97-30.48)	39.3(34.05-45.55)	1.24(1.1-1.38)	1544.38(1391.93-1728.65)	1716.63(1559.57-1905.18)	0.27(0.23-0.31)	6.74(5.42-8.11)	8.1(6.14-10.19)	0.23(-0.02-0.47)	155.66(126.25-185.06)	186.43(145.61-231.08)	0.26(0.06-0.46)
Barbuda	Both	27.45(24.32-30.84)	41.84(36.79-46.66)	1.43(1.3-1.57)	1496.04(1345.5-1660.01)	1783.19(1602.68-1968.97)	0.62(0.58-0.66)	7.47(5.97-9.04)	11.33(8.78-14.12)	1.75(1.52-1.99)	178.32(142.46-215.83)	247.76(194.52-304.71)	1.37(1.19-1.55)
Barbuda	Female	24.37(21.19-27.72)	38.1(33.15-42.93)	1.53(1.41-1.66)	1387.31(1232.16-1554.35)	1649.55(1475.51-1840.89)	0.66(0.62-0.7)	5.03(3.96-6.22)	10.27(7.91-12.76)	2.65(2.51-2.79)	126.33(101.03-156.22)	229.84(180.13-285.4)	2.22(2.11-2.33)
Barbuda	Male	31.08(27.05-35.36)	45.94(40.39-52.08)	1.31(1.16-1.46)	1629.14(1466.62-1819.4)	1930.08(1731.89-2148.02)	0.55(0.51-0.6)	11.21(8.97-13.74)	12.86(9.83-16.32)	0.91(0.47-1.34)	246.96(195.21-301.08)	270.46(209.83-335.59)	0.61(0.31-0.91)
Belarus	Both	12.04(10.46-13.9)	15.82(13.85-17.97)	0.99(0.66-1.31)	1738.53(1538.07-1952.26)	1684.26(1506.47-1854.98)	-0.19(-0.24--0.14)	0.3(0.21-0.43)	0.38(0.25-0.54)	0.92(0.53-1.32)	11.29(8.62-14.58)	14.73(10.91-19.4)	1.13(0.92-1.35)
Belarus	Female	12.16(10.52-14.22)	13.94(12.04-16.04)	0.34(-0.05-0.73)	1654.88(1452.81-1875.52)	1551.8(1370.39-1729.37)	-0.29(-0.34--0.23)	0.25(0.18-0.37)	0.31(0.21-0.45)	0.88(0.54-1.22)	10.45(7.92-13.61)	12.53(9.1-16.57)	0.77(0.64-0.91)
Belarus	Male	12.2(10.52-14.16)	19.32(16.62-22.17)	1.9(1.67-2.14)	1862.89(1627.17-2094.97)	1871.26(1665.9-2074.78)	-0.06(-0.1--0.02)	0.42(0.29-0.59)	0.52(0.35-0.77)	0.91(0.49-1.33)	13.22(9.97-16.88)	18.66(13.88-24.91)	1.48(1.18-1.77)
Belgium	Both	31.28(27.91-34.9)	33.23(29.97-36.91)	0.21(0.14-0.28)	961.79(878.73-1052.05)	959.56(878.16-1042.26)	-0.06(-0.08--0.04)	1.75(1.26-2.42)	1.75(1.22-2.54)	0.11(0-0.21)	38.3(29.17-48.49)	34.64(26.15-44.94)	-0.23(-0.32--0.15)
Belgium	Female	31.69(27.81-35.57)	33.21(28.96-37.61)	0.15(0.09-0.21)	929.82(842.42-1024.14)	919.91(828.13-1010.97)	-0.08(-0.09--0.06)	1.68(1.21-2.33)	1.67(1.13-2.41)	0.01(-0.1-0.12)	37.32(28.53-47.36)	32.72(24.76-42.43)	-0.41(-0.51--0.31)
Belgium	Male	31.07(27.41-35.33)	33.52(29.57-37.52)	0.28(0.2-0.36)	999.57(906.86-1099.03)	1002.96(913.55-1100.79)	-0.04(-0.07--0.02)	1.9(1.37-2.6)	1.87(1.31-2.75)	0.1(0-0.21)	39.99(29.63-51.74)	37(27.82-48.6)	-0.11(-0.2--0.01)
Belize	Both	23.8(20.58-27.27)	36.63(32.37-40.8)	1.43(1.36-1.5)	1412.75(1277.09-1558.37)	1620.15(1482.12-1762.99)	0.4(0.38-0.43)	6.24(5.01-7.62)	11.32(8.99-14.03)	2.3(1.76-2.84)	148.92(119.09-179.24)	275.37(220.29-336.85)	2.3(1.84-2.77)
Belize	Female	22.75(19.67-26.23)	34.88(30.37-39.87)	1.38(1.31-1.45)	1365.28(1224.12-1526.42)	1543.75(1395.57-1693.08)	0.37(0.34-0.39)	6.27(4.92-7.86)	10.45(8.32-12.88)	2.35(1.9-2.8)	150.11(118.94-184.15)	259.38(208.03-318.55)	2.35(1.96-2.74)
Belize	Male	24.82(21.29-28.88)	38.36(33.53-43.57)	1.48(1.4-1.56)	1457.97(1312.47-1618.89)	1694.54(1538.32-1866.98)	0.44(0.41-0.47)	6.2(4.89-7.62)	12.2(9.52-15.21)	2.28(1.61-2.97)	147.68(116.83-179.38)	291.24(231.7-363.19)	2.27(1.71-2.84)
Benin	Both	17.08(15.27-19.11)	22.83(20.11-25.78)	1.01(0.95-1.08)	1077.29(968.5-1197.48)	1174.1(1062.56-1295.12)	0.25(0.22-0.28)	8.99(6.7-11.64)	8.78(6.36-11.86)	0.08(-0.03-0.19)	181.96(133.8-232.62)	177.16(128.55-239.71)	0.05(-0.04-0.15)
Benin	Female	16.37(14.46-18.51)	22.05(19.21-25.15)	1.04(0.98-1.11)	1068(954.52-1186.2)	1156.24(1044.08-1280.74)	0.26(0.23-0.29)	7.57(5.47-9.96)	7.55(4.89-10.35)	0.17(0.04-0.31)	157.66(114.64-207.42)	155.5(104.99-216.58)	0.12(0-0.24)
Benin	Male	17.68(15.54-19.83)	23.78(20.65-27.1)	1.03(0.97-1.1)	1089.35(967.63-1219.85)	1194.46(1066.99-1328.45)	0.24(0.21-0.27)	10.5(7.58-13.99)	10.38(7.12-14.76)	0.13(0.01-0.24)	207.32(149.74-277.74)	203.3(139.52-285.31)	0.07(-0.03-0.18)
Bermuda	Both	23.99(21.12-27.06)	38.25(34.05-42.97)	1.58(1.37-1.78)	1386.66(1246.91-1536.27)	1542.43(1406.18-1685.28)	0.32(0.27-0.37)	4.7(3.69-5.79)	4.02(3.05-5.16)	-0.24(-0.42--0.07)	111.66(89.2-134.19)	101.47(81.14-125.74)	-0.13(-0.26-0)
Bermuda	Female	21.31(18.64-24.4)	33.8(29.7-38.47)	1.55(1.35-1.74)	1304.83(1162.75-1456.79)	1411.64(1271.57-1554.85)	0.24(0.2-0.28)	3.56(2.78-4.42)	2.98(2.19-3.89)	-0.67(-0.82--0.51)	85.39(67.97-103.02)	77.34(61.56-97.61)	-0.45(-0.56--0.34)
Bermuda	Male	27.21(23.74-30.95)	43.09(37.81-49.28)	1.57(1.36-1.79)	1488.63(1344.7-1655.34)	1693.07(1531.76-1851.28)	0.38(0.32-0.44)	6.41(5.06-7.91)	5.44(4.17-7.04)	0.06(-0.2-0.32)	146.01(115.76-176.57)	130.8(104.71-161.79)	0.08(-0.13-0.28)
Bhutan	Both	15.69(13.63-18.01)	24.97(21.9-28.4)	1.76(1.7-1.82)	1396.18(1245.27-1566.3)	1536.16(1377.83-1699.87)	0.31(0.29-0.33)	5.8(3.56-8.65)	8.18(5.53-11.27)	1.33(1.26-1.4)	138(86.35-201.52)	181.01(126.02-244.44)	1.05(0.99-1.12)
Bhutan	Female	14.61(12.52-16.86)	22.94(19.87-26.06)	1.72(1.67-1.78)	1300.53(1152.75-1474.03)	1406.48(1257.32-1565.83)	0.29(0.27-0.31)	5.64(2.87-8.87)	8.1(4.33-11.56)	1.31(1.25-1.37)	136.57(76.68-204.16)	177.87(102.32-254.86)	0.93(0.87-0.99)
Bhutan	Male	16.93(14.68-19.7)	26.91(23.53-31.43)	1.75(1.67-1.82)	1486.69(1314.87-1675.4)	1655.09(1478.17-1842.02)	0.33(0.3-0.36)	6.09(3.49-10.97)	8.26(5.35-12.11)	1.28(1.16-1.41)	140.87(83-240.83)	184.16(121.57-265.69)	1.14(1.02-1.25)
Bolivia	Both	22.16(19.76-24.73)	34.27(30.56-38.13)	1.49(1.44-1.54)	1168.7(1058.44-1293.14)	1301.93(1186.77-1422.19)	0.32(0.29-0.36)	10.82(8.23-14.02)	16.26(11.9-21.72)	1.5(1.43-1.57)	229.27(172.47-298.05)	314.93(228.62-418.68)	1.16(1.09-1.23)
Bolivia	Female	18.1(16.03-20.31)	30.38(26.75-34.37)	1.76(1.69-1.83)	1044.47(931.35-1174.8)	1170.31(1061.59-1290.27)	0.36(0.31-0.4)	9.95(7.41-14.19)	15.88(11.35-21.7)	1.66(1.6-1.72)	213.53(158.35-305.7)	307.37(223.83-422.07)	1.28(1.22-1.34)
Bolivia	Male	26.63(23.31-30.3)	38.48(33.97-43.48)	1.26(1.23-1.29)	1308.79(1177.69-1445.22)	1443.28(1309.14-1588.41)	0.28(0.25-0.31)	11.95(8.57-15.72)	16.72(11.51-23.17)	1.3(1.19-1.4)	248.43(179.3-322.97)	323.62(223.73-443.57)	1.02(0.92-1.13)
Bosnia and Herzegovina	Both	16.65(14.8-18.86)	29.55(26.13-33.13)	2.23(2.08-2.37)	1231.61(1111.23-1372.23)	1387.54(1268.82-1520.7)	0.45(0.41-0.48)	1.61(1.14-2.18)	2.4(1.62-3.38)	1.23(0.5-1.96)	43.37(31.85-55.51)	58.99(43.36-78.3)	0.92(0.31-1.53)
Bosnia and Herzegovina	Female	16.03(13.97-18.34)	26.79(23.37-30.73)	1.98(1.83-2.12)	1215.36(1082.01-1374.16)	1344.93(1216.44-1495.78)	0.39(0.35-0.43)	1.72(1.2-2.32)	2.27(1.54-3.23)	0.4(-0.29-1.09)	45.32(33.17-57.62)	55.1(40.97-73.83)	0.2(-0.36-0.76)
Bosnia and Herzegovina	Male	17.65(15.5-20.11)	33.29(29.14-37.84)	2.47(2.33-2.62)	1259.51(1131.59-1409.63)	1444.45(1310.4-1597.48)	0.51(0.47-0.55)	1.44(0.99-1.98)	2.57(1.71-3.7)	2.37(1.51-3.24)	40.59(29.55-53.26)	63.87(46.04-85.94)	1.81(1.1-2.52)
Botswana	Both	19.95(17.52-22.47)	31.01(27.31-34.89)	1.41(1.24-1.58)	1218.33(1094.8-1356.7)	1385.38(1258.65-1510.79)	0.36(0.31-0.42)	6.08(4.03-8.88)	9.41(6.34-13.81)	1.08(0.66-1.51)	134.17(87.75-192.06)	202.13(136.12-289.66)	0.95(0.5-1.41)

Botswana	Female	14.84(12.77-17)	24.86(21.55-28.45)	1.67(1.49-1.84)	1140.79(1012.73-1293.5)	1290.91(1155.2-1429.21)	0.37(0.32-0.43)	3.92(2.38-7.05)	7.29(4.58-11.89)	2.09(1.89-2.3)	85.3(52.78-145.99)	152.47(98.29-241.19)	2.03(1.84-2.22)
Botswana	Male	26.55(23.32-30.01)	39.23(34.7-44.17)	1.25(1.11-1.38)	1321.09(1184.6-1465.56)	1517.48(1377.23-1662.18)	0.37(0.32-0.43)	9.31(5.97-13.22)	12.85(7.81-18.97)	0.54(-0.08-1.17)	198.95(128.33-280.49)	271.69(176.26-389.67)	0.43(-0.23-1.09)
Brazil	Both	24.64(22.16-27.37)	31.84(28.99-35.17)	0.81(0.74-0.88)	1202.74(1107.89-1299.58)	1252.16(1157.88-1341.85)	0.07(0.04-0.1)	4.6(3.7-5.51)	4.58(3.66-5.54)	0.04(-0.05-0.13)	106.69(86.65-126.43)	103.75(84.78-122.4)	-0.23(-0.36--0.1)
Brazil	Female	23.06(20.72-25.67)	29.1(26.41-32.32)	0.75(0.66-0.83)	1150.41(1055.59-1245.57)	1171.45(1081.36-1258.15)	0.02(0-0.04)	3.89(3.07-4.74)	3.92(3.11-4.79)	0.05(-0.06-0.17)	91.86(74.2-109.13)	89.82(73.87-106.78)	-0.25(-0.41--0.09)
Brazil	Male	26.39(23.82-29.22)	35.04(31.86-38.74)	0.89(0.84-0.95)	1261.24(1162.39-1366.46)	1345.55(1246.34-1450.66)	0.13(0.1-0.17)	5.49(4.46-6.53)	5.47(4.34-6.65)	0.07(-0.01-0.14)	124.17(100.98-147.8)	121.2(98.66-144.3)	-0.17(-0.28--0.07)
Brunei	Both	43.09(38.02-48.31)	45.99(40.09-52.3)	0.18(0.04-0.31)	1813.59(1646.04-2001.02)	1774.01(1609.19-1953.07)	-0.14(-0.19--0.09)	16.69(13.98-19.7)	16.69(13.74-19.76)	0.7(0.44-0.96)	332.17(278.24-390.21)	306.14(255.06-357.56)	0.28(0.01-0.55)
Brunei	Female	37.44(32.98-42.46)	39.76(34.79-45.53)	0.18(0.03-0.32)	1737.18(1564.28-1933.89)	1676.62(1520.77-1859.54)	-0.16(-0.21--0.11)	13.51(10.97-16.58)	14.23(11.54-16.96)	0.62(0.48-0.76)	284.33(230.49-349.8)	276.04(226.28-330.36)	0.34(0.17-0.51)
Brunei	Male	49.61(43.27-56.01)	53.43(46.34-61.08)	0.2(0.08-0.32)	1912.82(1724.99-2118.9)	1899.11(1716.52-2106.34)	-0.11(-0.15--0.06)	23.9(19.42-29.17)	24.1(19-29.6)	0.97(0.34-1.6)	420.96(341.42-507.69)	382.18(304.86-461.94)	0.39(-0.11-0.89)
Bulgaria	Both	18.32(15.97-21.21)	29.65(26.28-33.47)	1.65(1.6-1.71)	1290.82(1157.94-1430.14)	1400.74(1268.65-1530.52)	0.19(0.14-0.23)	1.38(0.99-1.8)	2.88(1.98-4.02)	2.66(2.28-3.05)	41.3(31.05-51.95)	78.19(55.71-107.3)	2.26(1.97-2.55)
Bulgaria	Female	16.47(14.25-19.21)	25.09(21.55-29.13)	1.42(1.35-1.5)	1255.71(1108.98-1405.27)	1340.03(1193-1482.24)	0.13(0.09-0.18)	1.18(0.85-1.54)	2.33(1.6-3.22)	2.33(1.99-2.68)	36.76(27.58-46.82)	65.88(47.16-89.77)	2(1.74-2.26)
Bulgaria	Male	20.54(17.83-23.78)	35.85(31.45-40.46)	1.94(1.91-1.97)	1335.61(1193.76-1482.93)	1488.02(1349-1631.79)	0.28(0.24-0.33)	1.65(1.16-2.18)	3.71(2.47-5.17)	3.08(2.65-3.52)	46.87(34.43-58.81)	94.74(67.07-129.34)	2.57(2.24-2.91)
Burkina Faso	Both	15.25(13.56-16.92)	20.81(18.29-23.26)	1.15(1.05-1.25)	1048.41(944.41-1166.77)	1127.64(1027.86-1236.6)	0.21(0.18-0.24)	8.01(5.9-10.51)	8.36(6.27-11.06)	0.1(-0.01-0.21)	157.41(116.76-202.06)	167.16(125.84-219.5)	0.14(0.01-0.26)
Burkina Faso	Female	15.27(13.36-17.25)	21.02(18.35-23.83)	1.18(1.09-1.27)	1050.4(938.55-1178.78)	1119.5(1006.6-1239.4)	0.21(0.18-0.24)	6.64(4.65-9.11)	7.57(5.55-10.05)	0.38(0.28-0.47)	132.9(94.63-177.39)	153.08(111.42-201.16)	0.39(0.27-0.5)
Burkina Faso	Male	15.2(13.41-17.11)	20.57(18.01-23.19)	1.13(1.03-1.23)	1048.44(937.5-1170.9)	1136.4(1026.03-1257.56)	0.21(0.17-0.24)	9.53(6.93-12.56)	9.32(6.74-12.69)	-0.08(-0.22-0.05)	184.25(134.33-242.17)	184.35(135.11-249.49)	-0.03(-0.19-0.12)
Burundi	Both	11.77(10.54-13.06)	14.49(12.88-16.24)	0.82(0.75-0.9)	1038.46(924.64-1167.28)	1048.08(941.39-1171.09)	0(-0.02-0.02)	8.45(5.96-11.54)	6.66(4.78-8.94)	-1.1(-1.21--0.99)	178.04(128-239.75)	135.97(96.9-178.61)	-1.25(-1.37--1.12)
Burundi	Female	9.75(8.55-10.94)	12.63(11.01-14.31)	0.97(0.89-1.05)	1006.15(879.95-1147.76)	1008.36(898.18-1132.07)	-0.01(-0.03-0.01)	6.51(4.36-10.02)	5.58(3.92-7.96)	-0.85(-0.99--0.71)	140.09(94.66-209.4)	115.3(81.87-162.09)	-1.04(-1.19--0.88)
Burundi	Male	14.19(12.61-15.95)	16.14(14.24-18.32)	0.55(0.47-0.62)	1076.35(958.36-1213.46)	1086.71(971.87-1224.07)	-0.02(-0.05-0.01)	10.84(7.49-15.05)	7.7(5.26-10.81)	-1.44(-1.54--1.33)	224.11(157.34-314.89)	155.35(106.86-217.28)	-1.56(-1.68--1.43)
Cambodia	Both	14.09(12.28-16.16)	22.12(19.43-24.94)	1.56(1.45-1.67)	1778.37(1571.73-1997.91)	1853.87(1662.27-2070)	0.07(0.03-0.11)	8.16(6.26-10.35)	7.31(5.71-9.07)	-0.47(-0.56--0.38)	214.2(163.5-272.87)	181.11(140.56-228.34)	-0.69(-0.79--0.6)
Cambodia	Female	14.58(12.6-16.85)	22.81(19.84-26.1)	1.54(1.44-1.65)	1727.82(1515.34-1959.07)	1782.5(1588.82-1984.04)	0.06(0.02-0.1)	7.98(5.89-10.32)	6.94(5.3-8.75)	-0.66(-0.78--0.54)	216.41(161.85-278.13)	176.09(136.4-223.91)	-0.92(-1.03--0.8)
Cambodia	Male	13.45(11.65-15.45)	21.07(18.26-24.21)	1.56(1.44-1.68)	1844.44(1618.31-2092.36)	1942.59(1722.61-2177.63)	0.08(0.04-0.12)	8.47(6.26-11.27)	7.9(5.9-10.09)	-0.23(-0.3--0.15)	212.04(154.91-281.2)	188.38(139.85-244.57)	-0.4(-0.48--0.32)
Cameroon	Both	22.09(19.72-24.89)	30.95(27.39-35.09)	1.25(1.17-1.34)	1132.4(1027.48-1245.17)	1322.36(1204.86-1437.95)	0.58(0.52-0.63)	13.49(9.55-17.91)	12.9(9.48-17.14)	-0.13(-0.21--0.04)	269.83(194.41-356.43)	261.38(190.13-352.84)	-0.1(-0.23-0.02)
Cameroon	Female	22.52(19.8-25.76)	29.76(25.98-34.04)	1.07(0.99-1.15)	1145.98(1031.47-1264.38)	1305.44(1185.18-1429.92)	0.54(0.48-0.6)	13.86(8.48-19.04)	11.85(8.13-15.98)	-0.55(-0.59--0.51)	284.23(181.93-387.92)	242.68(167.09-330.34)	-0.53(-0.59--0.47)
Cameroon	Male	21.65(19.09-24.56)	32.25(28.18-36.77)	1.45(1.35-1.54)	1119.19(1002.22-1244.42)	1341.66(1213.68-1481.18)	0.61(0.55-0.68)	13.07(9.15-18.54)	14.13(10.19-19.84)	0.33(0.16-0.5)	254.51(176.95-358.24)	281.99(197.84-398.08)	0.35(0.14-0.56)
Canada	Both	29.46(25.51-33.43)	30.51(26.88-34.26)	0.26(0.2-0.32)	775.04(700.44-857.09)	923.25(837.62-1019.6)	0.58(0.43-0.72)	1.87(1.38-2.44)	1.87(1.35-2.55)	-0.42(-0.6--0.23)	40.73(31.44-50.58)	40.65(31.08-51.4)	-0.21(-0.34--0.09)
Canada	Female	26.3(22.59-30.57)	27.75(24.41-31.19)	0.28(0.22-0.34)	719.43(642.55-807.91)	898.57(803.2-999.22)	0.65(0.44-0.86)	1.8(1.34-2.33)	1.9(1.37-2.6)	-0.12(-0.26-0.01)	38.43(30.14-47.58)	39.69(30.63-50.58)	-0.02(-0.13-0.08)
Canada	Male	33.59(29.15-37.84)	33.62(28.97-38.82)	0.16(0.08-0.24)	848.94(764.27-938.65)	954.53(862.16-1059.34)	0.45(0.37-0.54)	1.97(1.43-2.61)	1.8(1.29-2.49)	-0.88(-1.13--0.63)	43.72(32.9-55.63)	41.5(31.04-53.49)	-0.48(-0.64--0.32)
Central African Republic	Both	11.11(9.86-12.45)	13.99(12.34-15.7)	0.85(0.75-0.94)	1075.43(944.23-1207.9)	1087.18(964.6-1209.52)	0(-0.03-0.04)	7.91(5.41-10.62)	8.07(5.44-11.12)	0.11(-0.02-0.25)	173.47(119.53-233.77)	175.5(118.28-244.46)	0.07(-0.06-0.2)
Central African Republic	Female	9.46(8.23-10.89)	12.28(10.6-14.14)	0.95(0.83-1.07)	1049.22(910.19-1184.53)	1048.4(917.74-1173.57)	-0.01(-0.05-0.02)	5.64(2.86-8.28)	5.79(2.59-9.05)	0.14(0.01-0.27)	124.07(66.1-180.92)	124.47(60.03-191.26)	0.06(-0.07-0.19)
Central African Republic	Male	13.02(11.59-14.6)	16.16(14.34-18.1)	0.8(0.74-0.87)	1105.65(978.29-1248.68)	1132.78(1011.67-1273.2)	0.03(0-0.06)	10.86(7.48-14.69)	11.54(8-15.68)	0.24(0.1-0.39)	234.15(161.63-315.71)	245.25(171.53-333.03)	0.18(0.04-0.33)
Chad	Both	15.89(14.17-17.73)	20.24(17.99-22.93)	0.86(0.73-0.98)	1044.71(939.91-1162.69)	1111.05(1007.02-1224.28)	0.16(0.12-0.2)	7.99(5.45-11.89)	8.41(5.86-11.81)	0.28(0.16-0.39)	164.17(112.54-238.43)	173.66(121.88-243.71)	0.31(0.19-0.42)
Chad	Female	15.21(13.16-17.31)	19.07(16.64-21.89)	0.8(0.68-0.92)	1037.21(928.61-1165.06)	1091.02(982.76-1215.42)	0.15(0.12-0.19)	6.93(4.41-11.93)	6.53(4.35-10.59)	-0.15(-0.22--0.09)	146.8(94.81-250.89)	140.04(94.46-222.38)	-0.1(-0.16--0.03)
Chad	Male	16.4(14.55-18.47)	21(18.55-23.85)	0.87(0.76-0.98)	1054.35(942.21-1183.68)	1134.81(1018.21-1258.09)	0.17(0.13-0.22)	9.13(6.04-13.75)	10.04(6.86-14.12)	0.45(0.26-0.65)	181.85(121.9-269.42)	201.58(139.63-285.49)	0.48(0.28-0.69)
Chile	Both	29.14(25.9-32.94)	43.81(39.27-49.05)	1.44(1.33-1.56)	1125.11(1023.99-1226.49)	1298.96(1187.94-1412.4)	0.47(0.42-0.52)	4(3.03-5.03)	5.85(4.48-7.46)	1.64(1.22-2.06)	86.42(66.14-105.06)	111.99(87.71-137.79)	1.08(0.71-1.46)
Chile	Female	28.7(25.23-32.89)	41.81(36.93-47.17)	1.33(1.2-1.46)	1130.76(1020.14-1249.34)	1279.88(1160.95-1403.37)	0.4(0.34-0.46)	3.66(2.74-4.62)	5.4(4.1-7.02)	1.67(1.24-2.1)	77.77(59.29-94.99)	101.04(79.06-124.66)	1.08(0.69-1.47)
Chile	Male	29.74(25.69-33.93)	46.37(40.86-52.81)	1.58(1.47-1.69)	1123.46(1015.07-1238)	1326.92(1211.08-1452.63)	0.54(0.5-0.59)	4.43(3.35-5.57)	6.4(4.92-8.19)	1.58(1.16-2)	97.06(74.94-118.18)	125.2(97.08-154.92)	1.08(0.71-1.44)
China	Both	19.44(17.31-21.78)	21.29(19.23-23.64)	0.53(0.44-0.62)	1678.25(1523.94-1851.42)	1606.88(1457.7-1760.75)	0.02(-0.08-0.12)	3.9(3.2-4.65)	3.57(2.83-4.32)	0.07(-0.09-0.22)	97.41(78.35-116.36)	82.96(66.87-99)	-0.07(-0.24-0.1)
China	Female	17.96(15.96-20.15)	19.78(17.91-21.91)	0.58(0.48-0.68)	1827.52(1649.8-2037.78)	1726.8(1562.23-1891.75)	0.02(-0.09-0.13)	3.71(2.94-4.53)	3.17(2.43-3.95)	-0.27(-0.42--0.12)	96.43(76.25-117.21)	76.62(60.35-93.78)	-0.37(-0.54--0.2)
China	Male	21.38(19.01-23.97)	23.21(20.78-25.91)	0.47(0.39-0.55)	1537.28(1394.88-1683.65)	1487.72(1352.52-1629.46)	0.01(-0.09-0.1)	4.37(3.44-5.45)	4.27(3.29-5.41)	0.44(0.25-0.62)	100.95(79.2-124.17)	91.64(71.98-113.97)	0.22(0.04-0.4)
Colombia	Both	29.78(26.16-33.94)	41.32(36.78-46.76)	1.01(0.93-1.09)	1740.38(1561.76-1923.37)	1831.41(1664.29-2005.04)	0.08(0.05-0.12)	5.75(4.44-7.06)	4.85(3.42-6.7)	-0.66(-0.85--0.46)	130.75(105.14-156.17)	116.51(88.86-151.77)	-0.58(-0.74--0.42)
Colombia	Female	28.02(24.37-32.19)	36.12(31.18-41.45)	0.8(0.72-0.87)	1650.79(1468.01-1852.26)	1665.93(1489.19-1847.37)	-0.04(-0.07-0)	5.53(4.29-6.82)	4.14(2.93-5.65)	-1.09(-1.32--0.86)	123.03(98.84-147.61)	100.49(76.05-130.4)	-0.87(-1.05--0.69)

Colombia	Male	31.61(27.63-36.58)	47.36(41.02-54.46)	1.25(1.16-1.34)	1834.15(1647.08-2031.12)	2026.16(1828.16-2235.02)	0.23(0.19-0.27)	5.97(4.61-7.35)	5.74(4.04-8.06)	-0.18(-0.37-0.02)	138.74(109.73-167.37)	136.02(102.23-179.34)	-0.25(-0.43--0.08)
Comoros	Both	12.39(11.03-13.85)	15.93(14.14-17.86)	0.94(0.87-1.01)	1046.57(941.02-1170.99)	1074.6(971.45-1197.45)	0.03(0-0.06)	8.04(5.35-11.05)	7.32(5.48-9.64)	-0.5(-0.61--0.38)	159.92(99.94-218.81)	144.63(108.1-189.57)	-0.55(-0.69--0.4)
Comoros	Female	11.15(9.82-12.6)	15.35(13.41-17.5)	1.17(1.11-1.23)	1017.72(902.16-1154.41)	1044.15(934.43-1166.66)	0.05(0.02-0.07)	6.74(4.48-9.45)	7.08(5.17-9.42)	0.03(-0.06-0.12)	138.43(86.75-192.38)	142.36(105.55-187.45)	-0.07(-0.18-0.05)
Comoros	Male	13.69(12.07-15.51)	16.71(14.75-19)	0.76(0.69-0.84)	1077.41(966.31-1208.36)	1105.4(988.62-1235.49)	0.01(-0.03-0.04)	9.47(6.03-13.79)	7.63(5.37-10.65)	-0.97(-1.13--0.82)	183.12(110.93-262.08)	148.12(104.01-200.75)	-0.98(-1.17--0.8)
Costa Rica	Both	60.77(56.43-65.37)	60.6(56.57-65.33)	-0.05(-0.06--0.03)	2297.78(2118.98-2491.23)	2269.17(2104.56-2456.65)	-0.1(-0.12--0.08)	4.2(3.27-5.18)	7.45(5.3-10.35)	1.77(1.08-2.46)	122.38(98.58-148.24)	185.26(140.6-239.83)	1.23(0.69-1.77)
Costa Rica	Female	60.24(55.6-65.19)	59.82(55.27-64.41)	-0.07(-0.08--0.05)	2229.54(2025.56-2445.3)	2172.11(1994.02-2367.54)	-0.14(-0.16--0.11)	3.82(2.96-4.78)	6.28(4.48-8.6)	1.54(0.87-2.21)	113.67(91.82-137.53)	158.18(120.33-205.15)	0.98(0.49-1.47)
Costa Rica	Male	61.4(56.28-66.95)	61.49(56.74-67.23)	-0.03(-0.04--0.02)	2369.39(2179.24-2574.52)	2379.29(2187.99-2589.1)	-0.05(-0.07--0.03)	4.6(3.57-5.68)	8.88(6.33-12.42)	2.01(1.3-2.73)	131.62(105.53-159.7)	216.82(161.04-285.48)	1.49(0.91-2.07)
Croatia	Both	22.13(19.28-25.32)	34.52(30.72-38.64)	1.64(1.55-1.72)	1312.63(1183.11-1460.55)	1394.84(1270.21-1525.76)	0.15(0.13-0.17)	1.76(1.29-2.29)	2.26(1.55-3.18)	0.6(0.24-0.97)	46.37(34.98-58.11)	52.98(39.66-68.36)	0.23(-0.07-0.54)
Croatia	Female	20.42(17.64-23.82)	31.36(27.1-35.86)	1.52(1.45-1.6)	1272.33(1127.05-1441.14)	1339.85(1210.96-1479.94)	0.1(0.08-0.13)	1.63(1.19-2.15)	2.15(1.46-3.03)	0.78(0.47-1.09)	42.66(32.2-53.61)	48.85(36.64-63.67)	0.23(-0.03-0.5)
Croatia	Male	24.78(21.24-28.4)	38.93(34.31-44.66)	1.72(1.61-1.82)	1376.43(1235.75-1529.83)	1468.43(1332.48-1613.02)	0.19(0.17-0.21)	2(1.46-2.66)	2.43(1.65-3.44)	0.23(-0.24-0.7)	51.76(38.75-65.51)	58.08(42.47-75.86)	0.12(-0.25-0.5)
Cuba	Both	18.12(15.98-20.56)	31.78(27.96-36)	1.9(1.81-1.99)	1327.12(1191.27-1485.68)	1471.42(1337.91-1616.71)	0.29(0.26-0.32)	2(1.59-2.4)	3.72(2.68-5.02)	2.77(2.5-3.04)	59.41(47.84-71.38)	100.6(76.38-129.34)	2.18(1.97-2.39)
Cuba	Female	17.25(14.78-20.09)	30.26(26.19-34.72)	1.89(1.8-1.98)	1267.51(1127.92-1432.33)	1389.48(1245.03-1532.21)	0.28(0.25-0.31)	1.68(1.33-2.02)	3.55(2.57-4.89)	3.46(2.98-3.94)	53.11(42.63-63.72)	96.92(73.02-127.98)	2.61(2.24-2.99)
Cuba	Male	19.02(16.53-22.06)	33.42(29.21-38.85)	1.92(1.82-2.02)	1387.94(1239.65-1567.23)	1558.88(1410.13-1722.77)	0.31(0.29-0.34)	2.33(1.84-2.83)	3.93(2.85-5.3)	2.17(1.99-2.36)	65.95(52.33-79.02)	104.77(78.91-134.83)	1.78(1.69-1.87)
Cyprus	Both	38.36(33.63-42.93)	39.96(35.2-45.19)	0.14(0-0.27)	1076.81(978.91-1185.04)	1040.49(949.15-1135.52)	-0.16(-0.23--0.1)	8.48(6.05-11.65)	4.77(3.4-6.66)	-2.32(-2.49--2.14)	123.34(91.87-165.18)	71.33(54.38-93.62)	-2.22(-2.33--2.1)
Cyprus	Female	36.53(32.04-41.34)	36.77(31.87-42.16)	0.04(-0.1-0.19)	1010.83(913.84-1123.22)	959.22(864.79-1064.51)	-0.21(-0.27--0.15)	8.99(6.11-12.88)	4.4(3.02-6.25)	-2.64(-2.9--2.38)	120.95(88.11-165.32)	61.71(46.19-81.05)	-2.55(-2.71--2.39)
Cyprus	Male	40.39(35.09-45.58)	43.58(37.93-49.59)	0.24(0.11-0.37)	1150.06(1038.58-1267.72)	1130.99(1024.28-1237.92)	-0.12(-0.19--0.04)	8.07(5.55-11.3)	5.09(3.55-7.11)	-1.88(-2.25--1.5)	127.39(92.07-169)	81.02(60.75-107.8)	-1.88(-2.11--1.65)
Czech Republic	Both	18.91(16.52-21.37)	28.79(25.15-32.39)	1.36(1.24-1.48)	1298.56(1173.77-1435.41)	1362.31(1236.74-1488.43)	0.1(0.08-0.12)	1.88(1.38-2.42)	1.38(0.96-1.89)	-1.04(-1.17--0.92)	50.25(37.84-63.39)	37.41(28.24-47.65)	-0.99(-1.07--0.91)
Czech Republic	Female	17.87(15.53-20.32)	26.02(22.51-29.48)	1.2(1.08-1.33)	1263.96(1128.65-1415.13)	1311.31(1179.05-1452.71)	0.08(0.07-0.1)	1.65(1.24-2.1)	1.27(0.87-1.74)	-1.06(-1.19--0.93)	43.59(33.54-54.59)	33.68(25.55-42.66)	-1.02(-1.1--0.94)
Czech Republic	Male	20.43(17.52-23.31)	32.43(27.81-37.35)	1.52(1.4-1.64)	1349.3(1204.71-1490.98)	1427.51(1290.05-1567.25)	0.11(0.09-0.14)	2.24(1.62-2.95)	1.52(1.06-2.12)	-1.1(-1.29--0.9)	59.06(44.04-74.53)	41.88(31.47-53.95)	-0.99(-1.12--0.87)
Democratic Republic of the Congo	Both	11.8(10.55-13.15)	15.49(13.74-17.25)	0.93(0.83-1.03)	1097.22(980.5-1232.23)	1105.13(997.01-1224.41)	-0.05(-0.09--0.01)	7.93(6.1-10.2)	6.56(4.56-8.81)	-0.75(-0.8--0.71)	158.53(119-203.01)	132.72(94.38-178.38)	-0.71(-0.77--0.66)
Democratic Republic of the Congo	Female	10.89(9.52-12.34)	14.88(13.05-16.85)	1.07(0.97-1.17)	1076.43(952.41-1223.05)	1073.28(965.62-1196.69)	-0.06(-0.09--0.02)	6.32(4.61-8.26)	5.75(3.73-7.99)	-0.43(-0.48--0.37)	129.28(94.18-170.05)	117.15(80.62-162.17)	-0.43(-0.49--0.38)
Democratic Republic of the Congo	Male	12.86(11.45-14.39)	16.17(14.15-18.17)	0.75(0.68-0.83)	1120(995.6-1260.04)	1137.27(1011.53-1275.77)	-0.05(-0.09--0.01)	9.59(6.98-12.62)	7.73(5.35-10.55)	-0.85(-0.92--0.78)	189.93(138.43-252.7)	154.4(108.77-207.36)	-0.84(-0.92--0.76)
Denmark	Both	25.74(22.35-29.18)	31.86(28.18-35.55)	0.8(0.76-0.83)	903.38(818.36-993.1)	952.62(868.91-1040.27)	0.15(0.13-0.16)	0.89(0.64-1.2)	1.84(1.32-2.56)	2.26(1.89-2.62)	25.48(19.29-32.16)	37.28(28.91-47.81)	1(0.71-1.29)
Denmark	Female	24.94(21.6-28.63)	30.27(26.35-34.36)	0.7(0.68-0.72)	863.41(776.88-963.52)	899.48(811.56-988.83)	0.09(0.07-0.11)	0.78(0.56-1.02)	1.53(1.09-2.12)	1.95(1.71-2.19)	23.87(18.27-30.22)	32.12(25.13-40.43)	0.53(0.33-0.72)
Denmark	Male	26.99(23.34-30.85)	33.73(29.27-38.63)	0.86(0.8-0.91)	953.99(854.88-1056.35)	1011.36(914.21-1115.34)	0.18(0.15-0.2)	1.11(0.79-1.52)	2.29(1.61-3.24)	2.36(1.82-2.9)	28.19(21.03-36.53)	43.76(33.26-57.02)	1.32(0.9-1.75)
Djibouti	Both	12.59(11.22-14.09)	17.25(15.33-19.34)	1.21(1.08-1.33)	1036.61(928.46-1152.38)	1119.54(1012.77-1232.82)	0.25(0.21-0.28)	6.28(4.35-8.97)	8.05(5.83-10.75)	0.88(0.78-0.97)	127.53(87.54-178.98)	158.19(113.97-212.8)	0.74(0.64-0.84)
Djibouti	Female	11.22(9.86-12.7)	15.86(13.91-18)	1.32(1.19-1.45)	1006.72(893.79-1137.05)	1081.18(970.55-1208.19)	0.26(0.22-0.3)	5.18(3.5-7.84)	7.18(5.03-10.73)	1.23(1.16-1.31)	106.92(72.94-161.29)	141.38(99.17-205.64)	1.05(0.98-1.12)
Djibouti	Male	13.91(12.25-15.59)	18.32(16.09-20.76)	1.06(0.94-1.18)	1064.38(945.67-1183.88)	1155.05(1038.71-1281.62)	0.24(0.2-0.28)	7.52(4.96-10.76)	8.8(6.27-11.88)	0.5(0.36-0.63)	149.23(97.28-215.94)	172.55(121.24-233.4)	0.44(0.3-0.57)
Dominica	Both	29.21(25.68-32.94)	39.2(34.65-43.99)	0.95(0.78-1.12)	1535.08(1392.32-1701)	1672.39(1528.78-1827.76)	0.24(0.17-0.31)	10.1(8.14-12.33)	13.6(10.4-17.27)	1.35(1.23-1.47)	231.33(186.67-279.95)	302.22(235.01-380.87)	1.15(1.05-1.24)
Dominica	Female	26.7(23.17-30.78)	35.45(30.89-39.87)	0.91(0.75-1.08)	1464.9(1310.41-1641.76)	1555.35(1407.03-1715.25)	0.17(0.1-0.23)	8.64(6.79-10.66)	12.18(9.38-15.82)	1.7(1.49-1.91)	201.48(159.58-248.77)	271.28(208.57-345.55)	1.41(1.24-1.58)
Dominica	Male	32.47(28.31-36.49)	42.86(37.57-48.73)	0.9(0.73-1.06)	1631.82(1475.6-1810.92)	1785.96(1619.74-1965.25)	0.23(0.16-0.3)	12.44(9.81-15.38)	15.37(11.33-19.87)	0.84(0.77-0.9)	276.02(218.89-337.96)	334.9(250.06-433.73)	0.72(0.64-0.8)
Dominican Republic	Both	16.09(14.14-18.42)	28.72(25.47-32.37)	1.74(1.6-1.88)	1239.69(1119.75-1383.2)	1426.61(1299.2-1562)	0.4(0.37-0.43)	3.64(2.79-4.55)	6.25(4.35-8.63)	2.98(2.62-3.34)	88.47(69.46-108.49)	153.78(109.91-211.73)	2.72(2.43-3)
Dominican Republic	Female	14.88(12.95-17.19)	26.46(23.13-30.02)	1.73(1.58-1.87)	1179.77(1053.02-1325.37)	1331.87(1202.19-1468.83)	0.35(0.33-0.38)	3.24(2.46-4.05)	5.66(3.79-7.84)	2.99(2.61-3.38)	78.22(61.63-96.12)	136.24(98.68-184.76)	2.62(2.37-2.87)
Dominican Republic	Male	17.34(15.12-19.9)	31.02(27.1-35.53)	1.75(1.61-1.88)	1300.67(1165.46-1450.96)	1525.47(1377.1-1673.82)	0.44(0.4-0.47)	4.04(2.99-5.13)	6.86(4.52-10.54)	2.97(2.52-3.43)	98.86(74.6-122.9)	172.13(117.29-258.63)	2.79(2.44-3.15)
Ecuador	Both	22.3(19.46-25.42)	45.36(40.59-50.56)	2.4(2.25-2.55)	1191.1(1074.39-1319.19)	1512.36(1387.38-1645.89)	0.77(0.74-0.81)	5.38(4.27-6.55)	14.15(10.54-18.71)	3.73(3.04-4.43)	116.24(92.83-139.1)	272.56(203.76-356.78)	3.23(2.58-3.88)
Ecuador	Female	21.64(18.74-24.79)	43.83(38.84-49.17)	2.37(2.18-2.57)	1106.63(992.38-1229.57)	1415.09(1288.25-1549.96)	0.82(0.76-0.87)	5.57(4.38-6.78)	13.68(10.14-17.76)	3.43(2.75-4.11)	116.65(92.5-140.02)	263.91(197.44-343.72)	3.06(2.44-3.69)
Ecuador	Male	22.95(19.97-26.27)	46.98(41.17-53.09)	2.43(2.32-2.54)	1277.95(1145.72-1427.97)	1614.83(1469.92-1767.8)	0.74(0.71-0.77)	5.27(4.11-6.61)	14.69(10.79-19.68)	4.02(3.31-4.74)	116.36(91.95-141.87)	282.19(205.28-375.62)	3.39(2.72-4.06)
Egypt	Both	36.47(32.24-41.62)	65.61(58.36-73.72)	2(1.86-2.14)	1379.85(1254.46-1509.13)	1829.87(1671.05-1986.64)	0.95(0.91-0.99)	11.94(7.77-16.05)	14.42(8.5-21.48)	0.86(0.75-0.96)	246.45(167.6-322.24)	302.75(184.53-442.24)	0.89(0.81-0.96)
Egypt	Female	41.97(36.69-48.42)	65.14(57.79-73.67)	1.63(1.38-1.88)	1511.03(1366.27-1674.57)	1876.1(1703.76-2052.46)	0.82(0.72-0.92)	13.34(7.03-19.34)	18.39(8.14-27.52)	1.32(1.22-1.42)	278.32(157.8-388.16)	362.63(179.52-526.21)	1.08(0.99-1.17)
Egypt	Male	31(27.2-35.34)	65.61(57.58-75.06)	2.46(2.39-2.54)	1249.24(1127.98-1375.53)	1783.08(1629.23-1946.57)	1.12(1.08-1.17)	10.5(7.38-14.83)	11.91(7.33-19.96)	0.66(0.52-0.79)	214.26(154.14-295.49)	259.32(166.88-419.6)	0.87(0.77-0.96)

El Salvador	Both	29.79(25.76-34.73)	48.71(43.18-54.9)	1.71(1.63-1.79)	1770.01(1595.13-1966.07)	2086.16(1907.68-2284.15)	0.59(0.55-0.62)	5.43(4.27-6.63)	19.43(13.68-26.21)	4.66(3.86-5.47)	149.27(118.06-180.61)	470.92(342.22-632.72)	4.32(3.56-5.07)
El Salvador	Female	26.02(22.48-30.46)	39.87(34.79-44.86)	1.61(1.53-1.68)	1642.39(1456.76-1850.04)	1811.71(1647.64-2017.58)	0.42(0.38-0.45)	4.42(3.42-5.41)	13.03(9.21-17.59)	3.96(3.42-4.51)	121.67(95.97-148.49)	305(223.2-409.91)	3.58(3.04-4.12)
El Salvador	Male	34.05(29.06-39.8)	60.7(51.93-69.8)	1.92(1.81-2.02)	1915.28(1711.23-2135.79)	2455.87(2218.55-2710.66)	0.82(0.78-0.87)	6.6(5.16-8.29)	28.17(19.66-38.52)	5.3(4.33-6.27)	180.74(141.81-223.2)	696.13(490.41-951.18)	4.97(4.08-5.86)
Equatorial Guinea	Both	11.03(9.85-12.29)	20(17.75-22.47)	2.36(2.21-2.5)	1078.11(959.86-1216.8)	1194.82(1076.55-1325.61)	0.36(0.33-0.39)	7.51(5.06-10.42)	9.47(6.38-13.19)	1.03(0.91-1.15)	161.72(109.66-220.08)	173.89(119.95-249.64)	0.43(0.27-0.59)
Equatorial Guinea	Female	9.38(8.27-10.53)	18.98(16.71-21.51)	2.73(2.59-2.88)	1050.32(921.79-1193.74)	1156.2(1029.1-1286.98)	0.36(0.33-0.39)	5.31(2.57-8.16)	8.4(4.62-12.56)	1.99(1.83-2.15)	115.5(59.97-177.26)	153.45(89.6-233.77)	1.35(1.17-1.53)
Equatorial Guinea	Male	13.12(11.63-14.74)	21.48(18.8-24.41)	1.98(1.84-2.12)	1113.85(985.2-1261.82)	1235.52(1108.91-1383.42)	0.33(0.31-0.36)	10.6(6.65-14.92)	11(7.45-15.39)	0.13(0.04-0.22)	222.87(143.59-315.43)	203.22(136.69-283.49)	-0.37(-0.54--0.21)
Eritrea	Both	10.69(9.42-12.07)	14.87(13.06-16.8)	1.08(0.99-1.16)	1005(892.89-1130.18)	1053.41(943.48-1170.66)	0.09(0.06-0.12)	6.6(3.99-10.09)	7.7(5.13-10.84)	0.44(0.27-0.61)	146.96(92.85-213.68)	159.24(107.96-223.44)	0.19(0.04-0.33)
Eritrea	Female	9.19(8-10.39)	13.12(11.4-15.01)	1.17(1.09-1.26)	975.24(856.35-1103.16)	1012.96(901.87-1137.65)	0.08(0.05-0.12)	4.97(2.55-9.36)	6.77(4.3-10.81)	1.11(0.95-1.28)	110.84(61.38-201.23)	138.33(90.17-212.85)	0.84(0.72-0.97)
Eritrea	Male	13.11(11.47-14.83)	17.49(15.21-20.01)	0.92(0.85-1)	1045.21(924.96-1181.48)	1102.37(982.26-1234.9)	0.09(0.05-0.12)	9.56(5.36-14.5)	9.18(4.97-13.45)	-0.34(-0.55--0.14)	205.32(120.82-304.79)	190.55(111.05-277.07)	-0.48(-0.67--0.28)
Estonia	Both	14.78(12.72-17.27)	24.8(21.3-28.6)	1.97(1.8-2.13)	1763.37(1573.48-1979.13)	1850.13(1666.83-2050.47)	0.15(0.13-0.17)	0.45(0.32-0.61)	2.06(1.38-3.04)	5.67(5.12-6.22)	15.51(11.7-19.81)	41.74(30.37-56.05)	3.65(3.24-4.06)
Estonia	Female	15.13(12.94-17.75)	21.57(18.25-25.4)	1.22(1.01-1.43)	1673.64(1480.2-1902.43)	1702.93(1528.76-1905.01)	0.02(-0.01-0.06)	0.38(0.26-0.52)	1.95(1.31-2.92)	5.92(5.44-6.39)	14.18(10.69-18.22)	37.4(27.27-50.27)	3.42(3.1-3.74)
Estonia	Male	14.47(12.41-17.11)	30.02(25.76-34.52)	3.02(2.85-3.19)	1889.69(1677.37-2131.76)	2047.94(1837.08-2272.15)	0.3(0.28-0.33)	0.63(0.44-0.87)	2.21(1.48-3.23)	4.98(4.27-5.7)	18.48(13.9-23.57)	48.09(34.49-64.87)	3.78(3.19-4.38)
Ethiopia	Both	11.89(10.65-13.19)	14.79(13.16-16.53)	0.7(0.61-0.79)	1012.05(921.48-1109.37)	1033.77(941.48-1125.25)	0(-0.04-0.04)	11.01(8.39-13.94)	7.62(5.86-9.54)	-1.31(-1.42--1.2)	232.59(175.89-291.49)	142.92(113.27-175.2)	-1.74(-1.86--1.63)
Ethiopia	Female	10.52(9.35-11.77)	14.1(12.53-15.93)	0.96(0.86-1.05)	1004.24(908.5-1106.7)	1017.29(923.56-1107.11)	-0.01(-0.05-0.02)	9.2(6.16-12.68)	6.64(5.08-8.41)	-1.33(-1.48--1.18)	200.66(138-268.11)	126.45(98.74-156.72)	-1.84(-2.01--1.66)
Ethiopia	Male	13.01(11.65-14.4)	15.42(13.74-17.23)	0.55(0.45-0.64)	1019.13(923.1-1115.99)	1050.44(957.15-1145.27)	0.02(-0.02-0.07)	12.7(9.29-16.64)	8.52(6.31-11.13)	-1.28(-1.37--1.2)	260.89(186.7-347.41)	158.11(117.07-204.21)	-1.64(-1.73--1.55)
Fiji	Both	26(22.77-29.29)	33.66(29.57-37.42)	0.75(0.7-0.79)	2144.74(1920.41-2407.73)	2223.81(2014.57-2464.83)	0.02(-0.01-0.05)	12.6(9.84-16.26)	17.18(13.26-21.54)	0.54(0.14-0.94)	305.2(235.41-393.33)	392.55(308.19-500.62)	0.4(0.04-0.76)
Fiji	Female	20.26(17.48-23.09)	26.91(23.28-30.34)	0.8(0.74-0.86)	2165.86(1924.82-2463.88)	2207.8(1979.58-2463.73)	-0.02(-0.05-0)	10.06(7.13-13.91)	13.27(10.12-17.36)	0.6(0.24-0.96)	262.04(187.42-355.4)	329.48(253.31-424.24)	0.46(0.15-0.77)
Fiji	Male	32.03(28.03-36.2)	42.07(36.94-46.91)	0.8(0.75-0.85)	2128.4(1906.07-2386.34)	2258.62(2036.13-2511.7)	0.08(0.05-0.12)	16.01(12.6-21.15)	24.1(18.68-30.44)	0.84(0.37-1.31)	355.02(274.47-460.97)	483.56(376.53-622.47)	0.51(0.09-0.93)
Finland	Both	20.21(17.59-23.02)	24.6(21.79-27.66)	0.5(0.41-0.58)	824.11(736.5-907.45)	890.37(804.35-970.22)	0.14(0.06-0.23)	0.86(0.64-1.09)	1.27(0.91-1.71)	2.29(2-2.57)	21.14(16.76-25.87)	25.15(19.8-31.27)	1.12(0.9-1.34)
Finland	Female	20.91(17.85-24.08)	26.09(22.51-29.81)	0.61(0.54-0.68)	783.63(698.58-869.98)	868.45(781.88-963.96)	0.29(0.24-0.35)	0.77(0.58-0.99)	1.2(0.84-1.61)	2.29(1.97-2.61)	19.55(15.51-23.98)	24.13(18.95-30.32)	1.06(0.92-1.21)
Finland	Male	19.21(16.51-22.04)	23.13(20.24-26.67)	0.37(0.24-0.5)	871.96(780.12-967.62)	912.07(820.93-1000.64)	-0.03(-0.15-0.1)	1.04(0.77-1.37)	1.38(0.98-1.9)	2.1(1.68-2.53)	23.73(18.49-29.36)	26.49(20.38-33.02)	1.1(0.74-1.46)
France	Both	26.27(23.36-29.6)	29.73(26.3-33.47)	0.3(0.23-0.37)	845.5(767.63-926.84)	833.54(756.81-911.08)	-0.26(-0.36--0.15)	1.41(0.99-1.95)	1.19(0.82-1.68)	-0.54(-0.7--0.38)	28.02(21.46-35.41)	25.05(19.2-32.29)	-0.56(-0.69--0.44)
France	Female	25.39(22.4-28.86)	28.54(24.81-32.5)	0.29(0.23-0.34)	788.81(711.93-874.79)	783.81(707.39-867.95)	-0.21(-0.3--0.11)	1.2(0.83-1.66)	1(0.67-1.41)	-0.67(-0.93--0.4)	24.73(18.54-31.3)	22.35(16.96-28.88)	-0.68(-0.88--0.48)
France	Male	27.7(23.93-31.93)	31.33(27.29-36.26)	0.3(0.21-0.39)	916.32(825.12-1011.81)	892.1(802.7-984.02)	-0.31(-0.42--0.2)	1.89(1.32-2.68)	1.52(1.07-2.16)	-0.63(-0.71--0.56)	33.82(25.31-43.98)	29.01(21.86-37.75)	-0.56(-0.63--0.49)
Gabon	Both	15.07(13.33-16.76)	22.98(20.22-25.7)	1.51(1.45-1.58)	1150.17(1029.82-1295.28)	1235.21(1117.58-1373.21)	0.2(0.17-0.22)	9.86(6.5-13.46)	11.91(6.54-16.8)	0.6(0.37-0.84)	199.61(136.15-272.49)	224.29(135.38-312.57)	0.36(0.12-0.59)
Gabon	Female	13.08(11.41-15)	21.34(18.61-24.58)	1.73(1.67-1.79)	1116.46(991.59-1262.22)	1196.23(1078.9-1331.75)	0.21(0.19-0.24)	7.38(3.44-10.75)	10.53(3.81-16.23)	1.21(0.85-1.57)	147.09(73.07-212.3)	193.3(78.48-295.77)	0.91(0.53-1.3)
Gabon	Male	17.43(15.32-19.59)	24.45(21.35-27.51)	1.2(1.15-1.24)	1188.08(1054.21-1344.97)	1278.67(1148.5-1419.9)	0.19(0.16-0.21)	13.45(8.64-18.07)	13.44(8.95-18.67)	-0.07(-0.21-0.07)	269.02(174.93-362.5)	259.18(178.68-355.21)	-0.2(-0.34--0.06)
Gambia	Both	16.7(14.9-18.63)	22.34(19.92-24.91)	0.99(0.94-1.05)	1064.94(963.27-1174.85)	1161.57(1056.36-1271.8)	0.25(0.22-0.27)	7.99(5.66-10.83)	8.74(6.39-11.71)	0.25(0.14-0.36)	158.65(113.11-214.74)	175.54(128.15-233.27)	0.28(0.14-0.43)
Gambia	Female	16.7(14.65-19.04)	21.87(18.88-24.78)	0.93(0.87-0.98)	1067.4(956.98-1183.94)	1151.89(1039.4-1265.96)	0.24(0.22-0.26)	6.89(4.68-9.72)	7.49(5.34-10.24)	0.16(0.03-0.29)	138.54(96.03-197.49)	152.72(108.75-207.43)	0.19(0.02-0.36)
Gambia	Male	16.87(14.91-19.01)	22.94(20.24-25.86)	1.04(0.98-1.1)	1065.94(950.32-1183.92)	1174.26(1051.52-1304.26)	0.25(0.22-0.28)	9.16(6.37-12.59)	10.2(7.16-14.3)	0.39(0.29-0.49)	177.97(123.25-244.91)	200.6(139.22-281.59)	0.42(0.29-0.55)
Germany	Both	32.39(28.7-36.23)	36.87(33.76-40.04)	0.3(0.24-0.37)	978.04(892.97-1077.07)	979.73(904.12-1058.48)	-0.19(-0.27--0.1)	1.89(1.4-2.46)	3.95(2.85-5.36)	3.46(3.04-3.89)	45.22(35.14-56.58)	61.25(47.37-78.6)	1.39(1.15-1.63)
Germany	Female	32.15(28.22-36.24)	36.1(32.47-39.59)	0.27(0.21-0.34)	937.02(845.94-1038.35)	930.01(850.4-1009.63)	-0.21(-0.29--0.12)	1.8(1.34-2.36)	3.81(2.7-5.21)	3.7(3.29-4.12)	43.2(33.83-53.83)	57.44(44.77-73.26)	1.45(1.27-1.64)
Germany	Male	32.84(28.69-37.2)	37.88(34.51-41.7)	0.31(0.24-0.39)	1033.65(931.73-1145.8)	1034.19(947.17-1125.73)	-0.21(-0.29--0.12)	2.06(1.5-2.75)	4.13(2.98-5.68)	2.99(2.54-3.44)	48.15(36.77-60.48)	65.52(50.03-84.52)	1.22(0.92-1.53)
Ghana	Both	14.74(13.03-16.6)	21.62(19.04-24.17)	1.31(1.24-1.37)	1058.55(947.02-1189.84)	1170.98(1057.63-1301.01)	0.28(0.25-0.3)	7.63(5.41-10.66)	9.56(6.56-12.9)	0.92(0.73-1.1)	156.16(110.91-213.08)	190.15(133.43-260.78)	0.91(0.73-1.09)
Ghana	Female	15.72(13.74-17.81)	22.04(19.1-24.82)	1.13(1.07-1.19)	1076.45(958.88-1206.4)	1166.26(1051.46-1297.7)	0.23(0.22-0.25)	8.27(5.52-12.8)	9.08(6.22-12.11)	0.18(0.06-0.3)	173.99(114.8-264.88)	181.27(125.79-241.49)	0.1(-0.01-0.2)
Ghana	Male	13.75(11.96-15.65)	21.19(18.52-24.04)	1.48(1.41-1.55)	1038.94(915.09-1182.3)	1173.5(1043.92-1317.88)	0.32(0.28-0.35)	6.87(4.81-9.45)	10.16(5.86-14.58)	1.84(1.56-2.13)	136.87(93.84-187.22)	201.54(123.31-291.47)	1.89(1.6-2.18)
Greece	Both	37.96(33.29-42.65)	36.26(31.98-40.37)	-0.14(-0.28-0)	1271.47(1027.22-1588.81)	1206.71(982.26-1482.93)	-0.24(-0.28--0.19)	4.59(3.37-6.21)	3.23(2.34-4.41)	-0.94(-1.35--0.52)	81.64(62.09-104.7)	60.83(46.97-77.57)	-0.55(-0.88--0.22)
Greece	Female	38.63(33.62-43.54)	36.26(31.44-40.87)	-0.2(-0.33--0.07)	1206.11(985.21-1497.05)	1126.58(923.59-1382.94)	-0.3(-0.34--0.25)	4.62(3.37-6.26)	3.05(2.16-4.2)	-1.22(-1.6--0.84)	81.83(61.94-104.58)	55.06(42.25-70.29)	-1.05(-1.36--0.73)
Greece	Male	37.25(32.27-42.38)	36.31(31.74-41.33)	-0.06(-0.22-0.09)	1343.24(1054.83-1701.04)	1293.7(1031.41-1609.95)	-0.18(-0.23--0.13)	4.56(3.33-6.22)	3.43(2.46-4.73)	-0.63(-1.11--0.14)	81.2(61.41-104.99)	67.25(50.94-86.39)	-0.07(-0.43-0.29)
Greenland	Both	23.95(21.08-26.97)	30.26(26.81-33.94)	0.76(0.72-0.79)	808.42(723.24-910.14)	1014.49(923.2-1113.7)	0.75(0.72-0.77)	3.24(2.4-4.32)	2.65(1.85-3.72)	-0.93(-1.14--0.72)	70.35(53.58-88.28)	58.76(43.63-77.44)	-0.78(-0.95--0.62)

Greenland	Female	21.34(18.51-24.32)	27.36(24.09-31.23)	0.77(0.73-0.81)	704.57(608.57-810.98)	980.27(877.67-1088.44)	1.06(1.02-1.1)	3.15(2.23-4.35)	2.48(1.71-3.51)	-1.51(-1.87--1.14)	67.02(50.31-86.34)	54.33(39.93-71.8)	-1.29(-1.58--1.01)
Greenland	Male	27.24(23.69-31.47)	32.84(28.8-37.16)	0.61(0.57-0.65)	908.03(812.41-1014.08)	1046.51(946.34-1151.26)	0.48(0.46-0.5)	3.33(2.36-4.55)	2.8(1.88-3.95)	-0.23(-0.53-0.08)	73.45(54.91-95.54)	62.52(45.19-83.91)	-0.25(-0.5-0)
Grenada	Both	26.8(23.64-30.09)	43.96(38.72-49.18)	1.55(1.4-1.7)	1470.23(1325.52-1620.54)	1730.16(1578.43-1890.5)	0.46(0.4-0.52)	9.71(7.82-11.8)	13.85(11.16-16.68)	1.6(1.43-1.78)	230.3(184.53-277.62)	322.01(259.52-390.56)	1.53(1.39-1.67)
Grenada	Female	22.56(19.5-25.79)	36.13(31.29-41.34)	1.43(1.26-1.6)	1378.39(1219.06-1540.74)	1570.83(1422.15-1735.51)	0.37(0.31-0.43)	7.35(5.86-8.94)	11.53(9.16-14.09)	2.14(1.8-2.48)	179.86(143.84-220.45)	269.66(218.11-333.27)	1.93(1.65-2.21)
Grenada	Male	32.2(28.02-36.75)	51.98(45.58-58.34)	1.53(1.38-1.67)	1590.52(1426.53-1761.23)	1903.63(1729.56-2085.69)	0.51(0.45-0.58)	13.18(10.47-16.2)	17.32(13.9-20.92)	1.26(0.72-1.8)	298.56(236.71-362.76)	384.8(311.03-464.9)	1.18(0.87-1.48)
Grenadines	Both	24.04(20.9-27.11)	34.63(30.59-38.74)	1.22(1.1-1.35)	1460.37(1314.6-1620.46)	1613.12(1468.6-1761.62)	0.29(0.25-0.33)	7.31(5.89-8.8)	10.55(8.35-12.86)	1.58(1.32-1.84)	170.92(137.07-206.08)	245.63(195.68-300.45)	1.44(1.27-1.61)
Grenadines	Female	22.19(19.13-25.52)	32.25(28.32-36.5)	1.24(1.12-1.35)	1401.62(1245.76-1573.71)	1516.48(1358.1-1664.75)	0.24(0.2-0.28)	6.72(5.36-8.22)	9.72(7.58-12.06)	1.76(1.38-2.13)	158.84(127.23-192.75)	229.07(181.61-282.21)	1.54(1.31-1.78)
Grenadines	Male	26.28(22.76-29.77)	36.86(32.11-41.93)	1.13(1-1.26)	1532.29(1373.45-1700.75)	1703.01(1544.75-1871.74)	0.29(0.24-0.34)	8.4(6.79-10.21)	11.54(9.06-14.22)	1.32(1.12-1.51)	187.94(148.89-226.98)	262.4(207.95-320.58)	1.26(1.12-1.41)
Guam	Both	23.27(20.25-26.48)	31.1(27.23-35.45)	0.77(0.6-0.94)	1982.06(1770.14-2206.8)	2119.3(1920.27-2340.19)	0.12(0.07-0.18)	8.76(7.15-10.58)	10.22(8.04-12.71)	0.79(0.46-1.11)	190.9(156.17-228.05)	263.58(205.38-324.98)	1.38(1.17-1.6)
Guam	Female	21.75(18.47-25.14)	27.85(23.9-32)	0.65(0.47-0.83)	2049.65(1818.57-2307.56)	2140.8(1922.99-2390.94)	0.07(0.02-0.12)	9.61(7.57-11.84)	10.03(7.66-12.62)	0.18(-0.26-0.63)	213.63(173.28-258.16)	267.05(207.07-328.9)	0.89(0.63-1.15)
Guam	Male	25.38(21.92-28.99)	35.02(30.65-40.04)	0.87(0.72-1.02)	1927.61(1709.93-2158.08)	2104.91(1879.48-2338.19)	0.17(0.12-0.23)	7.87(6.18-9.67)	10.59(8.15-13.52)	1.53(1.24-1.82)	169.13(136.24-207.44)	261.31(201.72-330.1)	1.94(1.74-2.15)
Guatemala	Both	31.71(27.91-36.24)	49.98(44.31-56.69)	1.5(1.39-1.62)	1747.49(1571.91-1928.97)	2084.5(1898.21-2278.67)	0.57(0.54-0.6)	8.76(6.82-10.82)	17.32(12.98-22.63)	3.02(2.66-3.39)	194.3(154.47-239.21)	381.61(288.78-496.39)	3.13(2.7-3.56)
Guatemala	Female	28.44(24.78-32.56)	44.93(39.39-50.92)	1.5(1.35-1.65)	1633.38(1460.35-1834.91)	1912.66(1734.68-2110.11)	0.52(0.49-0.56)	8.77(6.84-10.9)	15.95(11.77-20.67)	2.76(2.34-3.18)	189.14(150.22-231.5)	360.74(271.17-471.19)	3.1(2.59-3.61)
Guatemala	Male	35.06(30.06-40.47)	56.17(48.79-63.76)	1.57(1.49-1.66)	1862.66(1663.92-2073.32)	2292.21(2074.36-2530.14)	0.66(0.63-0.69)	8.83(6.69-11.29)	19.7(14.66-25.96)	3.49(3.1-3.88)	200.15(155.42-250.54)	411.88(310.66-536.57)	3.26(2.87-3.65)
Guinea	Both	16.51(14.73-18.56)	21.85(19.38-24.8)	0.98(0.91-1.05)	1034.76(928.86-1138.61)	1132.67(1019.92-1240.73)	0.26(0.24-0.29)	8.93(6.2-12.69)	8.9(6.42-11.96)	-0.13(-0.23--0.03)	183.46(126.36-252.84)	184.85(133.24-245.92)	-0.03(-0.13-0.07)
Guinea	Female	16.81(14.83-19.23)	21.5(18.78-24.51)	0.86(0.79-0.94)	1044.71(928.91-1155.37)	1126.97(1009.49-1238)	0.24(0.22-0.26)	9.22(6.14-14.03)	7.9(5.45-11.36)	-0.59(-0.75--0.43)	196.37(131.67-299.14)	168.67(118.35-236.7)	-0.51(-0.68--0.34)
Guinea	Male	16.16(14.3-18.16)	21.99(19.22-25.07)	1.07(1-1.14)	1026.72(912.32-1139.59)	1144.51(1018.84-1263.13)	0.3(0.26-0.33)	8.66(5.89-13.02)	9.89(6.9-13.43)	0.3(0.2-0.39)	170.3(115.32-257.01)	199.89(138.62-271.5)	0.42(0.33-0.51)
Guinea-Bissau	Both	17.78(16-19.78)	21.63(19.13-24.4)	0.72(0.63-0.82)	1093.1(984.17-1211.24)	1180.29(1069.81-1292.14)	0.22(0.2-0.24)	13.42(9.65-17.89)	11.3(8.24-15.1)	-0.58(-0.62--0.53)	291.54(207.39-387.78)	242.39(176.61-325.24)	-0.58(-0.63--0.52)
Guinea-Bissau	Female	16.23(14.34-18.42)	19.99(17.46-22.94)	0.78(0.69-0.88)	1085.88(973.28-1214.88)	1157.94(1041.65-1275.78)	0.21(0.19-0.23)	10.62(7.03-15.28)	9.2(6.32-13.23)	-0.55(-0.61--0.49)	237.16(157.32-340.41)	200.15(137.16-287.03)	-0.59(-0.66--0.52)
Guinea-Bissau	Male	19.17(17.09-21.25)	23.71(20.84-26.84)	0.76(0.68-0.84)	1102.62(979.1-1233.08)	1208.37(1085.36-1338.29)	0.24(0.21-0.27)	16.41(11.71-21.78)	14.17(10.39-18.61)	-0.43(-0.48--0.37)	347.06(240.45-476.33)	295.43(214.71-389.56)	-0.45(-0.52--0.39)
Guyana	Both	22.43(19.9-25.27)	35.86(31.67-40.41)	1.37(1.27-1.47)	1414.68(1271.49-1561.96)	1617.83(1473.06-1770.34)	0.34(0.29-0.39)	8.09(6.41-9.93)	13.36(9.74-17.44)	2.43(2.18-2.67)	196.93(156.85-243.05)	330.42(244.88-434.74)	2.47(2.24-2.71)
Guyana	Female	20.85(18.25-23.7)	32.92(28.84-37.5)	1.31(1.2-1.42)	1352.99(1207.92-1524.06)	1522.65(1373.75-1679.34)	0.31(0.27-0.36)	7.04(5.38-8.86)	12.07(8.74-15.8)	2.58(2.31-2.85)	174.17(135.55-217.46)	302.36(224.71-402.41)	2.59(2.35-2.83)
Guyana	Male	24.1(21-27.31)	39.05(34.18-44.67)	1.43(1.33-1.53)	1480.25(1313.65-1634.88)	1722.96(1557.22-1892.44)	0.37(0.32-0.43)	9.3(7.26-11.58)	14.96(10.73-19.78)	2.31(2.08-2.55)	221.78(173.29-277.35)	362.94(263.08-479.17)	2.39(2.15-2.62)
Haiti	Both	19.53(17.2-22.18)	27.94(24.53-31.81)	1.18(1.15-1.22)	1361.2(1219.23-1519.92)	1459.14(1319.78-1608.16)	0.2(0.18-0.23)	9.1(6.32-14.18)	9.2(6.06-14.47)	0.35(0.21-0.5)	222.73(157.51-330.41)	224.8(152.72-346.33)	0.4(0.24-0.55)
Haiti	Female	18.08(15.6-20.99)	26.39(22.97-30.19)	1.28(1.23-1.33)	1315.04(1164.21-1481.37)	1403.82(1262.49-1562.23)	0.21(0.19-0.24)	7.45(4.91-12.79)	8.16(4.94-14.12)	0.64(0.48-0.79)	194.54(131.79-310.92)	211.07(134.22-347.24)	0.65(0.5-0.8)
Haiti	Male	21.16(18.52-24.27)	29.69(25.96-33.95)	1.1(1.06-1.13)	1408.96(1255.58-1570.65)	1521.09(1367.25-1680.1)	0.2(0.17-0.23)	11.26(7.09-19)	10.38(6.58-17.05)	0.02(-0.18-0.22)	255.8(168.85-405.29)	240.2(156.71-374.41)	0.13(-0.08-0.35)
Honduras	Both	29.81(25.89-34.11)	42.34(37.24-48.05)	1.14(1.1-1.17)	1778.47(1598.55-1982.19)	1939.19(1758.87-2131.79)	0.24(0.22-0.26)	7.12(4.97-11.06)	16.92(12.43-23.02)	3.49(3.17-3.8)	175.44(126.64-248.86)	381.08(282.47-504.96)	3.09(2.83-3.36)
Honduras	Female	28.99(25.23-33.39)	40.56(35.62-46.27)	1.08(1.05-1.11)	1706.53(1514.07-1927.14)	1831.39(1639.08-2023.57)	0.2(0.18-0.22)	6.33(4.49-9.45)	17.2(12.31-23.67)	3.8(3.26-4.33)	165.97(122.68-225.36)	394.2(289.06-533.25)	3.31(2.9-3.73)
Honduras	Male	30.67(26.5-35.41)	44.35(38.44-50.6)	1.21(1.16-1.25)	1852.78(1653.98-2073.75)	2060.06(1862.39-2285.88)	0.29(0.26-0.31)	7.97(5.02-14.21)	16.61(11.98-23.45)	3.16(2.92-3.4)	185.56(126.79-301.75)	366.36(268.06-509.95)	2.85(2.66-3.04)
Hungary	Both	15.92(13.95-18.12)	29.2(25.77-33.09)	1.99(1.86-2.12)	1260.78(1132.44-1404.81)	1367.46(1243.93-1507.07)	0.2(0.17-0.23)	1.34(0.99-1.68)	2.09(1.5-2.86)	3.04(2.15-3.93)	35.88(27.81-43.95)	47.75(36.72-61.53)	1.97(1.29-2.65)
Hungary	Female	15.26(13.2-17.5)	27.37(23.88-31.44)	1.87(1.73-2.01)	1236.53(1089.63-1406.74)	1329.65(1201.12-1484.42)	0.18(0.15-0.21)	1.31(0.97-1.66)	2.11(1.5-2.88)	3.21(2.25-4.17)	34.33(26.44-41.78)	45.8(35.36-59.22)	2.02(1.29-2.75)
Hungary	Male	16.92(14.64-19.38)	32.15(27.84-36.94)	2.18(2.06-2.29)	1298.97(1162.3-1454.78)	1422.7(1286.9-1573.59)	0.24(0.21-0.26)	1.38(1.02-1.77)	1.98(1.4-2.76)	2.58(1.83-3.33)	37.86(28.59-47.28)	49.5(37.47-63.63)	1.84(1.24-2.44)
Iceland	Both	24.57(21.97-27.64)	26.24(23.18-29.35)	-0.02(-0.17-0.14)	869.16(786.65-950.68)	881.09(802.86-962.32)	-0.05(-0.09--0.01)	0.61(0.43-0.86)	0.72(0.49-1.05)	0.57(-0.26-1.4)	17.04(13.21-21.78)	18.13(13.81-23.8)	0(-0.45-0.46)
Iceland	Female	24.47(21.6-28.07)	25.31(22.14-28.9)	-0.16(-0.34-0.03)	850.81(761.15-946.81)	858.74(774.47-948.78)	-0.07(-0.13--0.02)	0.53(0.37-0.75)	0.57(0.38-0.84)	0.01(-0.86-0.88)	15.42(11.87-19.85)	15.84(12.14-21.01)	-0.28(-0.71-0.16)
Iceland	Male	24.74(21.94-27.92)	27.38(23.94-31.07)	0.13(0.01-0.26)	890.92(811.19-973.55)	906.32(825.12-993.58)	-0.03(-0.06-0)	0.72(0.5-1.03)	0.94(0.65-1.37)	1.04(0.29-1.8)	19.03(14.5-24.96)	20.93(15.51-27.67)	0.25(-0.22-0.73)
India	Both	20(17.92-22.37)	24.06(21.54-26.69)	0.43(0.31-0.55)	1773.61(1622.46-1939.24)	1781.26(1631.28-1936.93)	-0.27(-0.4--0.14)	5.45(4.04-6.93)	5.82(4.37-7.34)	0.08(-0.22-0.38)	130.69(99.63-163.89)	142.09(107.11-180.96)	0.33(0.06-0.59)
India	Female	16.47(14.69-18.5)	21.03(18.71-23.51)	0.65(0.55-0.74)	1632.24(1491.55-1788.96)	1625.37(1487.75-1757.7)	-0.3(-0.44--0.16)	4.19(2.85-5.52)	4.69(3.32-6.18)	0.17(-0.07-0.41)	102.55(73.77-133.28)	116.08(84.89-149.15)	0.38(0.21-0.55)
India	Male	23.33(20.97-25.91)	27.3(24.55-30.22)	0.35(0.21-0.48)	1903.51(1742.41-2074.96)	1936.85(1773.93-2113.93)	-0.22(-0.34--0.1)	6.65(4.87-8.78)	7.07(5.25-9.24)	0.12(-0.24-0.48)	157.13(117.62-202.15)	169.54(125.5-224.52)	0.37(0.01-0.72)
Indonesia	Both	15.07(13.47-16.87)	21.46(19.33-24)	1.08(0.99-1.17)	1964.2(1779.09-2168.58)	2044.29(1860.96-2227.02)	0.04(-0.02-0.1)	6.78(5.51-8.18)	7.16(5.68-8.7)	0.21(0.07-0.34)	178.25(141.51-214.97)	183.2(144.46-225.82)	0.11(0.01-0.2)
Indonesia	Female	15.35(13.67-17.22)	21.79(19.46-24.34)	1.09(0.98-1.19)	1879.29(1701.35-2075.66)	1936.75(1764.37-2102.48)	0.04(-0.03-0.1)	6.03(4.72-7.57)	5.76(4.31-7.29)	-0.19(-0.45-0.07)	165.83(128.95-207.15)	155.28(118.39-198.06)	-0.28(-0.46--0.09)

Indonesia	Male	14.76(13.16-16.54)	21.05(18.95-23.53)	1.06(0.97-1.15)	2056.07(1867.72-2265.08)	2156.23(1964.26-2364.39)	0.03(-0.03-0.1)	7.67(6.16-9.47)	8.84(6.75-11.13)	0.55(0.5-0.6)	192.23(149.81-237.97)	214.18(163.71-276.17)	0.44(0.41-0.47)
Iran	Both	41.75(37.27-46.62)	55.56(50.11-61.29)	1.01(0.91-1.12)	1601.46(1480.07-1724.47)	1763.47(1638.32-1890.19)	0.29(0.25-0.33)	7.32(5.88-9.15)	6.19(5.04-7.41)	-0.77(-0.88--0.66)	152.72(124.83-180.36)	126.85(105.48-147.59)	-0.81(-0.93--0.7)
Iran	Female	42.93(38.18-48.54)	55.61(50.23-61.33)	1.03(0.84-1.21)	1645.1(1519.17-1776.06)	1779.09(1650.47-1907.4)	0.29(0.22-0.35)	6.78(5.19-9.46)	5.97(4.72-7.15)	-0.78(-0.97--0.59)	142.52(112.67-179.68)	120.34(98.91-141.17)	-0.89(-1.09--0.7)
Iran	Male	40.89(36.4-45.88)	55.35(49.71-60.98)	0.94(0.88-0.99)	1561.53(1442.92-1681.13)	1748.76(1624.17-1876.96)	0.28(0.24-0.32)	7.89(6.29-9.59)	6.41(5.17-7.76)	-0.78(-0.84--0.72)	162.51(131.95-193.5)	133.37(110.84-157.01)	-0.73(-0.79--0.66)
Iraq	Both	40.64(35.78-45.92)	66.41(59.41-74.19)	1.86(1.8-1.92)	1587.64(1445.81-1742.32)	1933.82(1778.38-2100.71)	0.74(0.72-0.76)	15.36(11.44-21.56)	14.21(10.3-19.09)	-0.35(-0.42--0.29)	327.39(249.41-430.37)	297.51(225.49-383.86)	-0.43(-0.48--0.39)
Iraq	Female	42.64(37.36-48.32)	62.98(55.95-70.54)	1.64(1.48-1.79)	1684.73(1522.63-1862.39)	1942.88(1768.42-2125.93)	0.63(0.57-0.68)	14.6(10.27-21.87)	11.8(8.46-15.97)	-0.74(-0.85--0.64)	324.42(231.53-469.26)	259.88(188.78-344.38)	-0.79(-0.85--0.72)
Iraq	Male	38.62(33.61-43.56)	70.14(61.49-79.42)	2.12(2.04-2.2)	1492.49(1351.45-1645.06)	1933.89(1764.26-2134.66)	0.87(0.8-0.93)	16.34(11.72-23.72)	17.19(11.93-24.73)	0.02(-0.04-0.09)	332.49(240.4-450.17)	340.82(248.41-457.38)	-0.09(-0.14--0.04)
Ireland	Both	34.27(30.64-37.41)	33.49(31.95-35.1)	-0.06(-0.09--0.03)	1039.67(937.85-1139.96)	1032.06(959.87-1115.31)	-0.13(-0.17--0.1)	2.24(1.61-3.06)	1.8(1.25-2.56)	-0.79(-0.87--0.72)	44.87(34.45-57.51)	34.78(26.91-44.46)	-1.32(-1.53--1.11)
Ireland	Female	34.17(30.04-37.51)	31.67(29.78-33.65)	-0.15(-0.22--0.08)	1024.15(912.1-1131.34)	971.53(889.34-1059.58)	-0.19(-0.24--0.15)	1.98(1.42-2.69)	1.56(1.05-2.26)	-0.9(-0.99--0.82)	41.55(32.01-53.2)	30.83(23.87-40.09)	-1.47(-1.69--1.25)
Ireland	Male	35.01(30.61-39.72)	35.61(33.33-37.8)	-0.01(-0.03-0.01)	1060.69(955.04-1172.03)	1098.03(1012.53-1187.95)	-0.09(-0.14--0.03)	2.67(1.86-3.71)	2.17(1.5-3.07)	-0.72(-0.82--0.62)	49.89(36.95-64.83)	39.74(30.24-50.86)	-1.22(-1.43--1.01)
Israel	Both	37.77(33.08-41.99)	41.74(36.94-46.45)	0.32(0.18-0.46)	1056.27(959.04-1157.78)	1115.01(1023.63-1212.15)	0.11(0.05-0.16)	5.59(4.17-7.34)	5.89(4.41-7.8)	0.77(-0.01-1.56)	95.7(74.04-119.95)	98.15(75.85-122.92)	0.47(-0.33-1.28)
Israel	Female	37.04(32.63-41.52)	40.17(35.53-45.46)	0.28(0.15-0.41)	1013.22(908.23-1117.24)	1056.12(956.51-1165.87)	0.1(0.06-0.15)	5.31(3.91-6.94)	5.07(3.75-6.73)	0.8(0.1-1.51)	91.47(71.14-114)	83.7(65.69-103.58)	0.48(-0.23-1.18)
Israel	Male	38.65(33.28-43.5)	43.7(38.22-49.27)	0.37(0.22-0.52)	1107.13(997.07-1218.58)	1181.85(1076.73-1296.03)	0.1(0.03-0.18)	5.94(4.29-7.91)	6.96(5.23-9.26)	0.75(-0.13-1.65)	100.83(76.86-128)	115.52(88.39-146.61)	0.47(-0.45-1.39)
Italy	Both	28.22(24.98-31.73)	29(26.08-32.2)	0.03(-0.03-0.08)	992.32(913.88-1070.04)	967.71(891.07-1041.86)	-0.44(-0.64--0.25)	1.81(1.34-2.42)	1.55(1.08-2.13)	-0.55(-1.13-0.03)	38.52(29.78-47.92)	29.7(23.05-37.5)	-1.07(-1.4--0.73)
Italy	Female	26.32(23.35-29.43)	27.55(24.72-30.68)	0.03(-0.02-0.09)	945.82(867.26-1021.87)	916.19(842.02-993.98)	-0.53(-0.82--0.24)	1.62(1.2-2.15)	1.41(0.99-1.94)	-0.56(-1.23-0.13)	35.03(27.5-43.62)	27.32(21.08-34.59)	-1.14(-1.54--0.74)
Italy	Male	30.84(27.2-35.03)	30.86(27.65-34.44)	-0.01(-0.07-0.05)	1054.87(972.98-1134.62)	1027.15(945.49-1101.37)	-0.38(-0.5--0.26)	2.16(1.59-2.93)	1.78(1.26-2.5)	-0.57(-1.02--0.11)	43.86(33.5-55.22)	32.97(25.33-42.07)	-1.03(-1.3--0.76)
Ivory Coast	Both	19.47(17.4-21.64)	24.58(21.94-27.49)	0.86(0.78-0.94)	1138.99(1028.78-1263.22)	1216.32(1105.44-1339.69)	0.18(0.15-0.2)	9.85(7.13-12.77)	8.96(6.51-11.88)	-0.5(-0.66--0.34)	201.25(145.65-263.86)	183.3(131.14-242.56)	-0.51(-0.7--0.31)
Ivory Coast	Female	18.75(16.5-21.33)	23.81(20.83-26.91)	0.89(0.81-0.97)	1134.07(1019.72-1261.26)	1206.02(1087.63-1328.34)	0.19(0.17-0.21)	7.87(5.53-10.6)	7.26(5-10.05)	-0.29(-0.42--0.16)	163.14(116.92-220.28)	151.7(107.21-206.08)	-0.23(-0.42--0.05)
Ivory Coast	Male	20.08(17.86-22.62)	25.35(22.44-28.6)	0.87(0.78-0.96)	1144.65(1024.16-1278.2)	1227.51(1104.4-1372.14)	0.16(0.13-0.19)	11.68(8.34-15.66)	10.72(7.49-14.55)	-0.55(-0.72--0.37)	234.61(168.08-314.07)	213.58(148.52-292.14)	-0.61(-0.82--0.4)
Jamaica	Both	25.6(22.41-29.12)	34.2(29.89-38.84)	1.02(0.82-1.22)	1441.17(1301.82-1594.57)	1594.88(1449.88-1747.64)	0.33(0.26-0.4)	6.71(5.38-8.08)	8.72(6.54-11.25)	0.29(-0.25-0.83)	147.8(120.21-174.93)	217.29(166.61-278.39)	0.88(0.4-1.36)
Jamaica	Female	23.41(20.41-26.94)	30.17(26.21-34.47)	0.91(0.73-1.09)	1372.54(1229.87-1541.08)	1490.33(1346.09-1643.81)	0.28(0.22-0.34)	4.7(3.78-5.67)	6.92(5.19-9.01)	0.32(-0.34-0.99)	114.31(92.67-136.88)	187.27(143.02-243.43)	1.04(0.51-1.57)
Jamaica	Male	27.98(24.29-32.18)	38.33(33.4-44.2)	1.1(0.88-1.31)	1517.6(1363.01-1681.24)	1704.44(1545.38-1879.57)	0.36(0.28-0.44)	9.28(7.39-11.24)	10.9(8.05-14.12)	0.24(-0.29-0.78)	188.21(151.62-222.93)	250.95(191.1-322.4)	0.71(0.21-1.22)
Japan	Both	37.53(34.2-41.45)	40.12(36.2-44.31)	0.04(-0.01-0.1)	1579.35(1454.08-1707.16)	1565.65(1444.96-1686.76)	-0.12(-0.16--0.09)	4.73(3.84-5.63)	3.2(2.46-3.94)	-1.39(-1.56--1.23)	94.63(79.33-109.41)	69.71(56.93-83.23)	-0.88(-1.11--0.64)
Japan	Female	31.45(28.35-34.68)	30.81(27.76-34.05)	-0.1(-0.15--0.05)	1410.62(1294.44-1529.38)	1370.42(1258.71-1483.25)	-0.15(-0.2--0.1)	4.15(3.33-4.97)	2.56(1.85-3.23)	-1.75(-1.9--1.6)	82.81(68.76-96.22)	55.03(44.34-65.87)	-1.15(-1.34--0.96)
Japan	Male	45.11(41.06-49.88)	50.26(45.43-55.63)	0.05(-0.02-0.13)	1791.36(1657.23-1929.75)	1786.16(1651.88-1921.69)	-0.15(-0.18--0.11)	5.64(4.59-6.75)	4.04(3.2-4.93)	-1.15(-1.35--0.96)	110.94(92.28-129.39)	86.91(71.18-103.13)	-0.72(-0.98--0.45)
Jordan	Both	40.46(35.83-45.29)	69.27(62.34-76.89)	2(1.9-2.1)	1534.79(1402.47-1682.57)	1913.51(1753.73-2076.25)	0.82(0.79-0.84)	14.26(11.17-17.86)	12.64(9.73-15.83)	-0.33(-0.51--0.15)	284.26(225.17-351.51)	255.54(200.25-316.67)	-0.31(-0.49--0.12)
Jordan	Female	46.93(41.48-52.8)	67.51(60.25-75.43)	1.47(1.28-1.67)	1672.11(1524.3-1844.6)	1932.46(1765.66-2115.4)	0.63(0.55-0.7)	15.94(11.85-20.83)	13.43(9.89-17.47)	-0.72(-1.17--0.28)	320.81(241.83-414.85)	263.63(198.16-336.1)	-0.81(-1.23--0.39)
Jordan	Male	34.3(29.73-38.81)	70.88(62.72-79.91)	2.6(2.58-2.63)	1402.68(1270.53-1555.69)	1900.79(1739.46-2086.39)	1.04(1-1.07)	12.57(9.69-16.39)	11.93(8.6-16.11)	0.17(-0.05-0.39)	248.56(192.41-316.41)	247.88(185.46-326.65)	0.28(0.13-0.43)
Kazakhstan	Both	11.82(10.09-13.68)	21.04(18.2-24.28)	2.3(2.03-2.58)	1600.25(1428.86-1796.73)	1688.01(1526.69-1867.2)	0.16(0.1-0.21)	1.9(1.45-2.33)	3.62(2.76-4.59)	1.93(1.61-2.24)	59.71(46.86-72.99)	93.39(72.59-115.43)	1.08(0.79-1.38)
Kazakhstan	Female	11.28(9.48-13.17)	18.37(15.74-21.23)	1.91(1.62-2.2)	1536.28(1346.72-1740.12)	1587.03(1433.04-1772.14)	0.11(0.05-0.16)	1.65(1.26-2.04)	2.82(2.15-3.6)	1.42(1.11-1.73)	53.74(42.62-66.05)	74.59(58.39-91.62)	0.55(0.25-0.86)
Kazakhstan	Male	12.91(11.08-15.05)	25.17(21.57-29.24)	2.74(2.48-3.01)	1692.75(1500.15-1911.3)	1827.72(1640.29-2034.92)	0.23(0.17-0.29)	2.39(1.8-2.96)	5.03(3.83-6.46)	2.39(2.06-2.72)	69.86(53.35-86.11)	122.53(93.32-152.77)	1.62(1.32-1.91)
Kenya	Both	11.77(10.53-13.09)	14.8(13.2-16.6)	0.69(0.59-0.79)	1020(934.72-1111.39)	1058.92(971.4-1149.77)	0.04(0.01-0.07)	5.26(3.89-7.06)	6.56(4.91-8.37)	0.84(0.77-0.91)	102.13(77.04-133.15)	126.82(96.39-160.25)	0.84(0.73-0.94)
Kenya	Female	10.05(8.95-11.24)	12.76(11.32-14.37)	0.69(0.59-0.79)	988.17(903.74-1080.5)	1021.08(936.91-1112.84)	0.04(0.01-0.08)	4.51(3.34-6.13)	5.95(4.38-7.93)	1.14(0.98-1.29)	88.97(68.18-119.21)	114.04(84.51-151.75)	1.01(0.89-1.13)
Kenya	Male	13.57(12.17-15.13)	17.03(15.21-19.08)	0.7(0.63-0.78)	1052.88(968.14-1144.7)	1100.9(1012.42-1197.17)	0.04(0.01-0.07)	6.04(4.22-8.53)	7.22(5.01-9.84)	0.65(0.4-0.9)	115.75(82.42-161.79)	141.1(100.9-187.59)	0.74(0.46-1.01)
Kiribati	Both	17.96(15.65-20.65)	24.63(21.53-28.01)	0.98(0.91-1.05)	1965.14(1731.63-2217.69)	2021.78(1801.76-2246.37)	0(-0.03-0.03)	15.61(11.98-19.59)	20.13(14.83-27.31)	0.59(0.15-1.02)	409.07(310.95-523.01)	502.12(360.62-677.42)	0.42(0.03-0.82)
Kiribati	Female	15.65(13.32-18.18)	20.48(17.47-23.55)	0.82(0.75-0.89)	2017.54(1760-2301.34)	2025.67(1781.94-2270.04)	-0.06(-0.08--0.03)	17.07(12.54-22.21)	20.1(13.91-29.76)	0.27(-0.18-0.72)	455.77(333.15-593.89)	505.59(352.69-729.5)	0.08(-0.33-0.49)
Kiribati	Male	21.47(18.63-24.68)	31.2(27.37-35.48)	1.19(1.11-1.27)	1913.9(1682.26-2155.42)	2030.27(1797.96-2264.36)	0.08(0.05-0.12)	13.81(10.68-18.07)	20.36(12.72-27.88)	1.06(0.65-1.48)	354.95(268.22-468.97)	499.13(320.43-684.99)	0.89(0.51-1.28)
Kuwait	Both	41.56(36.93-46.87)	63.32(56.02-71.32)	1.54(1.48-1.6)	1534.48(1401.98-1679.28)	1797.03(1635.84-1958.99)	0.52(0.49-0.56)	8.89(6.97-10.85)	5.23(3.91-6.85)	-2.16(-2.97--1.34)	186.92(149.12-223.33)	116.07(90.5-144.05)	-1.89(-2.6--1.18)
Kuwait	Female	48.01(42.32-54.82)	62.19(54.66-69.69)	1.08(0.9-1.27)	1693.76(1537.15-1874.33)	1843.61(1681.3-2022.94)	0.39(0.29-0.48)	10.16(7.37-12.86)	5.3(3.69-7.17)	-2.09(-2.79--1.39)	220.5(162.95-274.46)	119.59(89.96-154.35)	-1.93(-2.57--1.28)
Kuwait	Male	38.17(33.05-43.52)	63.88(56.25-73.1)	1.77(1.72-1.81)	1441.3(1307.94-1590.02)	1759.64(1602.12-1927.34)	0.57(0.53-0.61)	8.11(6.35-9.96)	5.18(3.78-6.96)	-2.24(-3.18--1.29)	167.42(133.24-200.48)	113.44(86.92-144.4)	-1.92(-2.72--1.12)

Kyrgyzstan	Both	10.67(9.07-12.37)	15.67(13.46-18.22)	1.48(1.24-1.72)	1501.57(1342.2-1678.12)	1517.87(1375.52-1686.26)	-0.02(-0.07-0.03)	2.91(2.14-3.68)	2.54(1.88-3.24)	-1.03(-1.48--0.58)	95.35(71.36-120.6)	78.35(59.56-100.3)	-1.28(-1.69--0.87)
Kyrgyzstan	Female	10.06(8.43-11.94)	13.81(11.78-16.2)	1.16(0.9-1.43)	1434.9(1268.49-1618.24)	1425.74(1277.62-1593.7)	-0.07(-0.12--0.01)	2.46(1.8-3.12)	2.2(1.63-2.82)	-0.84(-1.25--0.42)	83.32(62.64-105.86)	67.71(51.81-86.98)	-1.23(-1.59--0.88)
Kyrgyzstan	Male	11.72(9.97-13.64)	18.27(15.54-21.25)	1.79(1.56-2.02)	1590(1414.74-1792.63)	1632.17(1468.06-1826.21)	0.03(-0.03-0.08)	3.68(2.68-4.71)	2.99(2.19-3.84)	-1.36(-1.83--0.9)	112.65(83.52-143.43)	91.44(68.97-116.9)	-1.39(-1.84--0.94)
Laos	Both	20.71(18.26-23.34)	29.37(25.87-33.1)	1.19(1.09-1.29)	1955.7(1745.48-2176.67)	2062.32(1859.84-2274.37)	0.13(0.1-0.16)	18.9(13.88-24.78)	16.26(12.38-20.89)	-0.7(-0.78--0.63)	471.49(340.12-613.83)	380.41(283.82-499.12)	-0.91(-0.98--0.84)
Laos	Female	20.79(18.23-23.71)	29.83(26.24-34.12)	1.24(1.14-1.34)	1903.52(1690.06-2140.79)	1984.74(1783.57-2191.83)	0.12(0.09-0.15)	18.3(13.07-24.49)	15.19(11.28-20.13)	-0.89(-0.98--0.8)	472.05(338.16-628.19)	364.4(268.96-484.06)	-1.13(-1.22--1.04)
Laos	Male	20.65(18-23.44)	28.89(25.17-32.75)	1.13(1.03-1.24)	2013.75(1781.5-2261.6)	2142.29(1919.73-2393.1)	0.12(0.09-0.16)	19.99(13.87-29.15)	17.58(12.78-23.92)	-0.56(-0.63--0.5)	474.42(329.02-670.5)	398.7(281.37-544.94)	-0.7(-0.76--0.64)
Latvia	Both	12.54(10.84-14.45)	19.41(16.87-22.26)	1.46(1.25-1.67)	1772.13(1579.89-1997.78)	1802.09(1624.59-1997.38)	0(-0.05-0.05)	0.26(0.17-0.36)	0.73(0.48-1.06)	4.95(4.37-5.54)	11.28(8.4-14.99)	22.67(16.59-29.92)	3.12(2.79-3.45)
Latvia	Female	12.89(10.93-15.22)	17.97(15.33-21)	0.87(0.57-1.16)	1693.66(1496.6-1916.13)	1680.63(1499.13-1881.2)	-0.09(-0.15--0.03)	0.21(0.14-0.3)	0.7(0.45-1.03)	5.67(5-6.35)	10.64(7.91-14.17)	21.15(15.29-28.12)	3.05(2.66-3.44)
Latvia	Male	12.28(10.69-14.23)	22.23(19.32-25.43)	2.4(2.26-2.55)	1888.5(1666.22-2138.98)	1967.36(1762.19-2195.33)	0.11(0.07-0.14)	0.36(0.25-0.53)	0.8(0.52-1.2)	3.72(3.17-4.27)	12.83(9.4-16.77)	25.28(18.26-34.36)	3.07(2.71-3.43)
Lebanon	Both	36.47(32.16-41.08)	67.11(60.1-75.06)	2.24(2.05-2.43)	1460.41(1333.9-1601.64)	1861.65(1712.82-2023.93)	0.92(0.88-0.97)	10.11(7.7-13.02)	7.02(4.84-9.69)	-1.22(-1.38--1.05)	207.17(159.42-263.14)	153.95(113.18-206.14)	-0.95(-1.13--0.77)
Lebanon	Female	39.41(34.37-44.81)	63.65(56.73-71.17)	1.86(1.58-2.14)	1551.39(1397.8-1710.49)	1855.88(1695.54-2023.27)	0.77(0.68-0.87)	9.9(7.16-13.67)	6.83(4.38-9.13)	-1.48(-1.63--1.32)	208.71(156.06-279.28)	149.84(106.46-195.12)	-1.27(-1.43--1.12)
Lebanon	Male	33.65(29.36-38.44)	71.32(63.31-80.72)	2.62(2.54-2.71)	1363.66(1235.31-1502.18)	1869.72(1703.25-2054.98)	1.09(1.06-1.11)	10.4(7.82-13.55)	7.25(4.17-12.17)	-0.96(-1.18--0.73)	206.16(155.57-268.59)	159.02(104.7-247.18)	-0.61(-0.86--0.37)
Lesotho	Both	19.2(17.06-21.52)	25.42(22.62-28.7)	0.8(0.56-1.04)	1169.95(1052.35-1297.22)	1289.95(1168.71-1422.56)	0.26(0.19-0.32)	5.46(3.78-7.49)	12.73(8.59-17.52)	3.55(3.19-3.92)	122.89(86.04-169.38)	275.77(187.35-387.53)	3.43(3.07-3.78)
Lesotho	Female	14.79(13.01-16.89)	20.56(18.05-23.44)	0.98(0.74-1.22)	1108.21(985.39-1247.82)	1210.13(1090.62-1343.86)	0.25(0.19-0.3)	3.37(2.26-4.85)	9.44(5.78-13.97)	4.75(4.23-5.27)	74.36(51.77-104.62)	195.52(122.08-290.56)	4.54(4.04-5.05)
Lesotho	Male	25.24(22.38-28.59)	32.74(28.9-37.23)	0.75(0.54-0.96)	1256.84(1122.5-1389.9)	1405.8(1260.26-1552.84)	0.29(0.21-0.36)	9.12(6.15-12.92)	18.53(12.13-25.29)	2.73(2.38-3.08)	192.04(130.37-268.84)	397.69(258.72-554.62)	2.85(2.49-3.22)
Liberia	Both	16.43(14.52-18.36)	22.31(19.66-25.25)	1.14(1.02-1.26)	1099.59(989.96-1222.64)	1187.64(1078.11-1306.75)	0.27(0.23-0.3)	10.85(7.84-14.45)	9.08(6.28-12.79)	-0.38(-0.67--0.1)	216.52(156.14-284.63)	180.9(124.79-252.37)	-0.4(-0.7--0.1)
Liberia	Female	16.72(14.6-19.17)	22.83(19.9-26.27)	1.16(1.03-1.29)	1107.52(992.43-1235.23)	1187.9(1072.79-1309.15)	0.27(0.23-0.31)	10.77(7.92-14.4)	9(6.06-12.35)	-0.35(-0.71-0.01)	222.11(163.44-295.42)	184.43(125.14-258.45)	-0.4(-0.76--0.03)
Liberia	Male	16.08(14.08-18.06)	21.98(19.11-24.91)	1.17(1.05-1.29)	1095.65(981.04-1231.46)	1189.78(1071.29-1325.46)	0.26(0.22-0.29)	10.92(7.52-15.96)	9.18(5.8-14.8)	-0.38(-0.61--0.16)	211.13(144.54-301.5)	178.09(114.13-287.15)	-0.38(-0.63--0.13)
Libya	Both	37.16(33.05-42.06)	61.98(55.25-69.59)	1.9(1.73-2.06)	1477.54(1345.22-1624.65)	1805.03(1655.4-1960.43)	0.74(0.67-0.81)	9.26(6.43-12.72)	9.61(6.08-13.78)	0.34(0.17-0.52)	197.77(141.5-260.39)	212.41(143.31-287.49)	0.41(0.26-0.55)
Libya	Female	39.96(35.22-45.27)	60.37(53.41-67.66)	1.64(1.37-1.91)	1577.5(1434.17-1747.43)	1843.74(1681.91-2014.67)	0.65(0.54-0.77)	9.68(6.38-13.98)	10.44(5.93-15.21)	0.66(0.36-0.95)	216.11(148.4-301.16)	233.99(148.16-326.27)	0.6(0.36-0.84)
Libya	Male	34.81(30.61-39.75)	63.48(55.89-71.76)	2.12(2.05-2.2)	1398.12(1267.54-1551.32)	1770.31(1611.89-1945.26)	0.8(0.77-0.83)	8.93(6.02-12.78)	8.81(5.51-14.3)	-0.02(-0.18-0.13)	182.65(128.03-255.14)	191.56(128.54-296.17)	0.14(0-0.28)
Lithuania	Both	13.68(11.77-15.82)	17.14(15.06-19.62)	0.81(0.63-0.99)	1772.53(1567.11-1995.09)	1718.5(1542.75-1911.38)	-0.14(-0.17--0.11)	0.32(0.22-0.44)	0.52(0.35-0.76)	1.44(0.92-1.96)	12.8(9.55-16.67)	18.06(13.47-23.7)	1.03(0.73-1.32)
Lithuania	Female	13.89(11.83-16.2)	15.38(13.24-18.33)	0.22(-0.03-0.47)	1685.35(1470.55-1919.58)	1580.26(1402.31-1779.9)	-0.24(-0.27--0.2)	0.26(0.17-0.36)	0.47(0.31-0.68)	1.68(1.15-2.22)	11.75(8.85-15.29)	16(11.92-20.88)	0.85(0.6-1.1)
Lithuania	Male	13.58(11.64-15.67)	20.23(17.52-23.03)	1.66(1.54-1.78)	1894.74(1669.44-2143.19)	1903.7(1700.91-2130)	-0.02(-0.04-0)	0.43(0.3-0.61)	0.65(0.43-0.96)	1.22(0.72-1.73)	14.71(10.98-19.08)	21.56(15.98-28.93)	1.24(0.89-1.58)
Luxembourg	Both	30.77(27.27-34.43)	35.62(31.78-39.68)	0.51(0.39-0.62)	990.81(898.67-1078.87)	1031.27(937.99-1120.31)	0.14(0.1-0.18)	2.13(1.54-2.93)	2.14(1.49-3.05)	0.25(0.02-0.48)	41.62(32.01-53.21)	40.4(30.75-53.01)	0.08(-0.14-0.3)
Luxembourg	Female	31.45(27.46-35.69)	35.65(31.26-40.19)	0.45(0.34-0.55)	978.96(887.17-1073.87)	1004.65(909.79-1104.71)	0.11(0.08-0.15)	1.99(1.46-2.77)	2.07(1.41-3)	0.36(0.13-0.59)	39.72(30.86-50.55)	39.75(29.94-52.06)	0.21(-0.01-0.42)
Luxembourg	Male	30.09(26.49-34.24)	35.96(31.7-40.45)	0.61(0.46-0.75)	1007.36(900.75-1102.63)	1059.93(960.17-1158.67)	0.16(0.11-0.21)	2.4(1.67-3.35)	2.27(1.55-3.32)	0.09(-0.16-0.35)	44.94(33.85-58.61)	41.54(31.43-54.93)	-0.08(-0.32-0.15)
Macedonia	Both	19.6(17.22-21.95)	35.21(31.5-39.58)	1.96(1.85-2.08)	1299.36(1171.19-1446.17)	1472.69(1345.29-1605.92)	0.39(0.36-0.42)	2.01(1.41-2.71)	2.54(1.71-3.58)	0.56(0.23-0.89)	55.82(41.46-71.88)	68.6(50.5-90.72)	0.58(0.33-0.83)
Macedonia	Female	17.33(14.98-20.11)	31.03(27.28-35.26)	1.91(1.8-2.03)	1262.62(1118.12-1425.8)	1419.33(1281.16-1572.56)	0.36(0.34-0.39)	1.69(1.18-2.25)	2.28(1.55-3.22)	0.75(0.34-1.16)	48.9(35.52-62.9)	62.64(45.94-83.35)	0.65(0.36-0.93)
Macedonia	Male	22.11(19.26-25.28)	40.21(35.29-46.01)	2.04(1.9-2.18)	1341.97(1210.13-1489.82)	1537.28(1400.96-1681.29)	0.43(0.39-0.47)	2.39(1.64-3.33)	2.88(1.88-4.2)	0.48(0.17-0.78)	63.52(46.94-84.28)	75.73(55.31-101.02)	0.54(0.27-0.81)
Madagascar	Both	10.63(9.44-11.95)	13.34(11.79-15.02)	0.72(0.65-0.8)	1009.11(905.38-1124.89)	1037.62(934.18-1148.65)	0.03(0-0.06)	5.74(4.02-7.96)	5.39(3.73-7.64)	-0.33(-0.43--0.22)	118.65(83.78-158.9)	112.12(79.98-154.3)	-0.3(-0.39--0.21)
Madagascar	Female	9.11(7.99-10.33)	11.92(10.41-13.59)	0.88(0.8-0.96)	981.84(868.54-1108.33)	1003.58(891.71-1120.79)	0.04(0.01-0.07)	4.58(3.15-6.88)	4.84(3.19-7.56)	0.05(-0.06-0.16)	99.8(69.4-150.09)	103.14(71.08-154.44)	-0.02(-0.11-0.08)
Madagascar	Male	12.02(10.58-13.66)	14.85(13.09-16.89)	0.66(0.6-0.73)	1035.72(923.32-1160.89)	1073.65(959.4-1197.34)	0.04(0-0.07)	6.8(4.49-9.61)	6.02(4.04-8.61)	-0.49(-0.6--0.37)	135.86(91.39-188.37)	122.28(83.49-170.28)	-0.44(-0.55--0.33)
Malawi	Both	12.66(11.26-14.17)	16(14.2-17.96)	0.89(0.82-0.95)	1806.95(1329.14-2388.05)	1875.7(1397.94-2492.72)	0.05(0.02-0.08)	7.17(5.42-9.2)	7.23(5.39-9.4)	-0.11(-0.27-0.05)	144.84(109.59-183.66)	144.2(108.24-184.09)	-0.18(-0.35-0)
Malawi	Female	11.14(9.72-12.59)	14.16(12.41-16.22)	0.9(0.83-0.97)	1742.88(1284.44-2339.09)	1779.38(1332.46-2349.44)	0.01(-0.01-0.04)	6.31(4.57-8.31)	6.1(4.46-8.03)	-0.21(-0.32--0.11)	129.4(94.81-168.66)	119.48(89.81-154.55)	-0.42(-0.53--0.3)
Malawi	Male	14.44(12.86-16.31)	18.25(16.23-20.54)	0.89(0.83-0.95)	1877.45(1382.57-2490.91)	1984.78(1465.16-2667.3)	0.1(0.07-0.13)	8.2(5.87-11.07)	8.76(6.33-11.8)	0.08(-0.2-0.36)	163.32(118.12-213.86)	176.02(127.42-231.92)	0.09(-0.2-0.38)
Malaysia	Both	23.04(20.47-25.94)	35.16(31.67-39.36)	1.51(1.42-1.59)	1894.91(1697.22-2115.34)	2056.63(1864.31-2256.89)	0.26(0.25-0.27)	9.76(8.07-11.44)	10.26(7.76-13.12)	-0.44(-0.72--0.15)	231.54(189.39-270.8)	235.26(186.27-293.81)	-0.38(-0.58--0.18)
Malaysia	Female	24.78(21.62-28.27)	39.11(34.74-44.12)	1.6(1.52-1.69)	1870.98(1659.41-2110.28)	2037.62(1840.31-2262.34)	0.29(0.28-0.3)	8.84(7.19-10.66)	10.9(8.16-14.06)	0.07(-0.28-0.42)	214.34(174.01-257.66)	245.65(190.98-311.31)	-0.07(-0.33-0.19)
Malaysia	Male	21.17(18.64-23.92)	31.16(27.44-35.57)	1.42(1.33-1.51)	1919.94(1701.76-2170.39)	2072.9(1856.73-2313.67)	0.22(0.2-0.23)	10.72(8.62-12.97)	9.61(7.1-12.49)	-0.98(-1.23--0.72)	249.36(200.8-299.84)	224.41(169.08-288.26)	-0.69(-0.86--0.53)
Maldives	Both	26.11(22.58-29.71)	40.9(36.04-45.89)	1.7(1.59-1.81)	2044.67(1839.49-2291.56)	2211.58(2011.78-2436.61)	0.25(0.24-0.26)	22.1(17.74-27.59)	13.09(10.17-16.61)	-2.23(-2.5--1.97)	474.13(381.23-593.36)	259.69(206.86-321.16)	-2.49(-2.79--2.19)

Maldives	Female	27.34(23.49-31.28)	42.72(37.05-47.89)	1.66(1.56-1.75)	1991.47(1774.15-2255.19)	2142.91(1949.49-2378.74)	0.26(0.24-0.27)	25.09(18.94-34.44)	14.23(10.82-18.5)	-2.48(-2.81--2.15)	567.15(420.37-785.62)	286.25(226.27-358.99)	-2.82(-3.16--2.46)
Maldives	Male	25.15(21.78-28.83)	39.39(34.34-45.02)	1.73(1.61-1.86)	2081.74(1859.39-2340.43)	2259.41(2047.73-2506.74)	0.25(0.23-0.26)	19.89(15.76-24.58)	12.05(9.18-15.3)	-2.09(-2.32--1.85)	405.03(320.52-491.02)	236.03(184.23-294.82)	-2.25(-2.52--1.97)
Mali	Both	15.3(12.73-18.85)	20.52(17.05-25.82)	1.05(0.95-1.15)	1028(924.48-1142.32)	1116.57(1018.73-1234.46)	0.24(0.21-0.27)	9.29(6.62-12.52)	8.32(6.08-10.98)	-0.2(-0.36--0.03)	193.29(138.41-256.56)	170.07(122.67-223.97)	-0.28(-0.46--0.11)
Mali	Female	15.66(12.71-19.6)	21.28(17.34-27.35)	1.06(0.95-1.17)	1040.48(925.35-1169.86)	1120(1011.11-1242.59)	0.23(0.19-0.26)	9.04(5.96-13.64)	7.86(5.39-10.75)	-0.39(-0.53--0.24)	196.8(130.53-290.61)	166.51(115.41-227.74)	-0.51(-0.67--0.35)
Mali	Male	14.88(12.44-18.4)	19.73(16.32-24.55)	1.05(0.94-1.16)	1016.39(909.15-1133.42)	1116.86(1003.77-1242.27)	0.25(0.22-0.29)	9.6(6.85-13.06)	8.75(6.02-11.96)	-0.05(-0.24-0.14)	189.84(133.12-257.97)	173.27(117.75-238.63)	-0.06(-0.27-0.14)
Malta	Both	33.73(29.85-37.82)	35.89(31.71-39.91)	0.18(0.09-0.27)	1041.46(951.67-1137.31)	1019.95(934.18-1108.28)	-0.13(-0.16--0.1)	3(2.18-4.08)	2.16(1.54-3.02)	-1.12(-1.35--0.88)	56.94(43.68-72.72)	44.16(33.64-56.8)	-0.85(-1.06--0.65)
Malta	Female	33.58(29.35-38.11)	35.41(31.01-39.88)	0.16(0.08-0.24)	1001.92(905.75-1104.73)	982.31(893.91-1076.07)	-0.14(-0.16--0.11)	2.68(1.92-3.67)	2(1.41-2.84)	-1.17(-1.42--0.93)	52.39(40.53-66.76)	42.08(32.32-54.46)	-0.88(-1.07--0.68)
Malta	Male	34.18(29.73-38.92)	36.77(31.81-41.57)	0.22(0.11-0.33)	1090.38(986.06-1205.39)	1062.21(964.6-1161.52)	-0.14(-0.19--0.1)	3.56(2.55-4.94)	2.45(1.73-3.49)	-1.1(-1.37--0.83)	63.84(47.73-84.08)	47.26(35.7-61.42)	-0.86(-1.1--0.62)
Marshall Islands	Both	21.07(18.56-23.97)	29.94(26.64-34.19)	1.14(1.11-1.18)	1937.25(1729.36-2192.68)	2031.87(1824.91-2252.37)	0.08(0.05-0.11)	13.84(10.36-18.83)	18.47(13.15-25.31)	1.01(0.76-1.26)	338.34(255.45-450.29)	449.18(318.16-619.34)	0.96(0.71-1.21)
Marshall Islands	Female	17.86(15.34-20.77)	24.9(21.58-28.55)	1.04(1-1.08)	1986.84(1741.42-2261.51)	2041.5(1810.29-2277.45)	0.03(0.01-0.05)	12.28(8.76-17.56)	18.14(12.21-27.93)	1.18(0.8-1.56)	316.74(229.11-445.49)	461.04(312.94-693.72)	1.11(0.73-1.49)
Marshall Islands	Male	24.95(21.9-28.47)	34.51(30.35-39.4)	1.05(1-1.1)	1897.57(1671.12-2147.57)	2020.24(1799.24-2266.52)	0.11(0.08-0.15)	16.22(12.08-22.62)	18.69(13.2-25.64)	0.68(0.48-0.87)	368.62(277.02-503.89)	436.54(308.38-594.43)	0.73(0.56-0.9)
Mauritania	Both	18.17(16.18-20.47)	25(21.84-28.25)	1.08(1.04-1.12)	1079.95(983.61-1185.71)	1181.35(1079.13-1284.55)	0.23(0.21-0.26)	12.77(9.42-16.6)	8.69(6.16-11.66)	-1.32(-1.41--1.23)	260.84(190.41-338.2)	168.77(118.37-224.59)	-1.43(-1.54--1.32)
Mauritania	Female	19.85(17.32-22.58)	27.43(23.85-31.35)	1.11(1.07-1.15)	1112.66(997.86-1232.23)	1226.98(1107.19-1352.61)	0.3(0.28-0.32)	13.45(9.4-18.13)	9.7(6.17-13.59)	-1.2(-1.28--1.12)	283.2(195.44-385.49)	192.32(128.25-268.24)	-1.34(-1.43--1.25)
Mauritania	Male	16.48(14.48-18.57)	22.48(19.56-25.69)	1.04(0.98-1.1)	1044.09(942.65-1161.97)	1138.04(1029.28-1254.23)	0.18(0.14-0.23)	11.99(8.67-15.89)	7.7(5.16-10.96)	-1.38(-1.54--1.23)	236.92(168.34-313.13)	145.25(96.56-204.27)	-1.49(-1.67--1.32)
Mauritius	Both	33.49(28.9-38.01)	50.84(44.04-57.09)	1.33(1.21-1.44)	2257.09(2027.6-2520.58)	2580(2349.81-2840.65)	0.4(0.36-0.44)	17.82(15.16-20.87)	31.07(24.41-39.32)	2.23(1.85-2.62)	427.98(352.92-498.37)	733.03(578.1-932.24)	2.2(1.81-2.58)
Mauritius	Female	33.54(29.16-38.15)	49.49(43.94-56.33)	1.21(1.1-1.31)	2197.37(1945.9-2475.63)	2480.68(2241.33-2761.8)	0.38(0.34-0.41)	15.79(13.12-18.71)	26.66(20.77-33.53)	2.01(1.62-2.41)	381.57(315.3-447.01)	628.81(495.97-802)	1.89(1.51-2.28)
Mauritius	Male	33.44(28.42-38.63)	52.35(40.67-61.01)	1.46(1.33-1.59)	2326.4(2083.24-2618)	2687.84(2398.69-2991.25)	0.43(0.38-0.47)	20.38(17.12-23.83)	36.52(28.55-45.77)	2.4(2.02-2.79)	482.98(394.92-565.7)	850.91(661.98-1081.06)	2.43(2.03-2.83)
Mexico	Both	41.25(36.78-46.69)	60.72(55.88-65.94)	1.21(1.11-1.3)	2077.01(1905.67-2259.67)	2375.01(2200.93-2542.51)	0.37(0.34-0.4)	8.26(6.83-9.74)	17.03(13.44-21.03)	2.8(2.36-3.25)	186.6(155.67-217.85)	410.66(324.66-508.17)	3.01(2.54-3.48)
Mexico	Female	44.86(39.83-50.76)	60.97(56.09-66.02)	0.92(0.82-1.02)	2094.07(1921.25-2288.84)	2308.8(2144.66-2475.92)	0.24(0.21-0.28)	8.7(7.14-10.29)	15.75(11.87-20.2)	2.36(1.88-2.83)	193.85(161.23-227.34)	379.02(291.72-481.07)	2.57(2.06-3.09)
Mexico	Male	37.34(33.25-42.4)	60.41(55.16-65.94)	1.55(1.46-1.65)	2057.66(1884.91-2234.66)	2447.93(2268.72-2641.64)	0.51(0.47-0.54)	7.87(6.45-9.31)	18.51(13.83-23.89)	3.28(2.87-3.68)	179.34(148.52-210.39)	446.68(335.59-570.97)	3.48(3.05-3.9)
Micronesia	Both	23.92(20.83-27.11)	36.36(32.15-41.12)	1.35(1.29-1.4)	1982.92(1774.42-2230.3)	2169.3(1962.8-2403.79)	0.24(0.21-0.27)	17.21(12.88-22.78)	27.45(20.22-36.33)	1.51(1.09-1.93)	425.29(317.65-562.28)	657.71(471.95-897.48)	1.41(1.03-1.78)
Micronesia	Female	20.71(17.63-24.08)	31.28(27.08-36.01)	1.32(1.24-1.4)	2038.47(1807.12-2336.75)	2178.09(1957.08-2431.95)	0.18(0.15-0.21)	17.55(12.39-24.84)	25.74(18.59-34.98)	1.11(0.58-1.64)	447.72(320.37-617.53)	631.24(449.3-871.23)	0.99(0.52-1.46)
Micronesia	Male	27.67(24.32-31.18)	42.93(37.79-48.34)	1.45(1.41-1.49)	1936.13(1724.75-2169.73)	2172.6(1956.82-2406.53)	0.3(0.27-0.34)	17.17(12.79-23.01)	29.96(18.86-42.09)	1.96(1.69-2.23)	405.92(298.41-545.41)	691.75(436.58-993.1)	1.87(1.62-2.13)
Moldova	Both	9.54(8.28-11.05)	13.91(12.04-16.16)	1.04(0.81-1.26)	1730.33(1528.65-1959.57)	1730.98(1552.1-1931.06)	-0.06(-0.08--0.03)	0.39(0.27-0.54)	0.56(0.39-0.78)	1.63(1.28-1.98)	12.3(9.49-15.67)	19.37(14.4-24.98)	1.67(1.4-1.95)
Moldova	Female	9.64(8.21-11.3)	12.9(10.95-15.3)	0.45(0.13-0.78)	1640.64(1426.27-1876.05)	1610.17(1431.27-1813.27)	-0.14(-0.17--0.1)	0.31(0.22-0.44)	0.5(0.34-0.7)	2.22(1.82-2.62)	11.01(8.47-14.11)	17.73(13.11-23.28)	1.76(1.44-2.09)
Moldova	Male	9.56(8.2-11.1)	15.71(13.53-18.16)	1.86(1.75-1.97)	1852.57(1635.87-2104.05)	1885.69(1681.61-2104.93)	0.03(0-0.07)	0.54(0.39-0.75)	0.69(0.47-0.97)	0.93(0.6-1.27)	14.52(11.03-18.46)	21.94(16.18-28.47)	1.51(1.27-1.75)
Mongolia	Both	16.42(14.18-19.12)	20.8(18.19-24.15)	1.02(0.89-1.15)	1521.25(1366.18-1690.69)	1575.84(1435.96-1729.43)	0.14(0.11-0.17)	8.98(6.52-11.71)	4.78(3.45-6.43)	-3.37(-3.87--2.88)	219.9(161.83-288.75)	121.95(88.03-162.2)	-3.18(-3.64--2.72)
Mongolia	Female	15.85(13.44-18.77)	18.31(15.81-21.4)	0.64(0.52-0.75)	1464.32(1302.39-1640.26)	1477.41(1325.6-1634.81)	0.03(0.01-0.05)	8.65(6.23-11.45)	4.01(2.83-5.5)	-3.97(-4.53--3.4)	223.49(160.38-301.12)	102.36(73.91-136.74)	-4.02(-4.57--3.48)
Mongolia	Male	17.33(14.94-20.19)	24.27(21.05-28.48)	1.45(1.3-1.61)	1584.84(1420.42-1777.81)	1699.33(1537.85-1883.1)	0.28(0.23-0.33)	9.82(6.9-13.25)	5.88(4.07-8.05)	-2.89(-3.32--2.45)	222.86(155.79-294.52)	147.74(104.38-199.41)	-2.39(-2.77--2.01)
Montenegro	Both	25.03(21.79-28.34)	35.37(31.12-40.16)	1.31(1.27-1.36)	1386.43(1246.25-1530.44)	1467.89(1336.92-1604.26)	0.18(0.16-0.2)	2.78(2.04-3.66)	3.24(2.33-4.34)	0.3(0.15-0.45)	67.2(51.22-83.95)	77.7(57.71-100.76)	0.29(0.14-0.44)
Montenegro	Female	21.97(18.84-25.38)	31.37(27.11-35.81)	1.3(1.27-1.33)	1333.56(1186.26-1490.28)	1409.32(1275.82-1559.96)	0.17(0.15-0.19)	2.21(1.59-2.95)	2.68(1.93-3.56)	0.37(0.12-0.61)	54.85(41.12-70.33)	65.45(49.4-83.82)	0.32(0.1-0.54)
Montenegro	Male	29.14(25.41-33.54)	40.57(35.5-46.89)	1.31(1.25-1.37)	1461.58(1321.58-1616.53)	1544.23(1406.14-1692.05)	0.17(0.16-0.19)	3.63(2.57-4.86)	4.02(2.75-5.67)	0.15(0.03-0.28)	83.66(62.1-107.12)	93.17(68.34-123.88)	0.2(0.09-0.32)
Morocco	Both	28.19(24.79-32.25)	59.83(53.32-67.35)	2.72(2.64-2.81)	1303.95(1183.31-1437.96)	1745.3(1604.31-1898.38)	1.04(1.01-1.06)	8.47(6.08-12.2)	10.76(7.65-14.67)	1.12(0.96-1.29)	179.3(133.16-243.07)	228.48(166.7-298.92)	1.17(1.04-1.31)
Morocco	Female	29.99(25.94-34.75)	56.34(49.56-64.24)	2.45(2.24-2.65)	1381.41(1248.75-1538.1)	1755.06(1600.55-1923.94)	0.95(0.9-1)	7.84(5.34-12.19)	10.62(7.29-14.84)	1.67(1.42-1.92)	174.9(125.23-249.76)	233.25(168.48-316.43)	1.6(1.36-1.83)
Morocco	Male	26.4(23.26-30.15)	63.49(55.7-72.43)	3.02(2.93-3.11)	1223.06(1095.28-1352.24)	1738.32(1577.62-1899.56)	1.14(1.06-1.22)	9.29(6.38-14.55)	10.93(7.3-16.42)	0.52(0.18-0.86)	184.95(131.78-264.26)	224.45(154.05-315.36)	0.72(0.5-0.93)
Mozambique	Both	11.07(9.96-12.25)	15.03(13.3-16.94)	1.01(0.97-1.06)	1047.33(936.5-1170.29)	1108.08(999.59-1223.31)	0.13(0.1-0.16)	5.95(4.23-8.07)	7(5.04-9.48)	0.58(0.44-0.71)	118.26(83.72-157.83)	140.26(101.71-186.62)	0.62(0.49-0.76)
Mozambique	Female	9.45(8.42-10.6)	13.32(11.63-15.22)	1.14(1.1-1.19)	1011.94(899.93-1148.69)	1060.66(952.06-1182.97)	0.12(0.09-0.15)	4.99(3.54-6.85)	5.83(4.07-8.17)	0.51(0.43-0.59)	100.1(72.58-133.53)	113.91(80.86-155.85)	0.41(0.33-0.49)
Mozambique	Male	12.87(11.42-14.42)	17.14(14.98-19.33)	0.95(0.91-0.99)	1088.55(967.09-1216.43)	1166.09(1045.18-1293.37)	0.15(0.12-0.18)	7.11(4.69-10.48)	8.66(6-11.93)	0.74(0.53-0.96)	139.2(93.46-201.19)	174.62(124.23-235.61)	0.88(0.67-1.09)
Myanmar	Both	18.66(16.58-20.92)	27.78(24.53-31.19)	1.35(1.23-1.47)	1924.11(1720.5-2153.19)	2029.15(1836.11-2243.11)	0.13(0.09-0.18)	10.44(7.73-13.49)	9.7(7.78-12.26)	-0.3(-0.41--0.19)	276.43(201.77-364.2)	239.14(188.72-306.73)	-0.58(-0.69--0.47)
Myanmar	Female	20.08(17.65-22.81)	29.61(25.83-33.65)	1.32(1.22-1.42)	1892.97(1671.92-2149.49)	1975.86(1766.49-2193.31)	0.12(0.08-0.17)	10.12(7.27-13.9)	9.01(7.11-11.79)	-0.52(-0.68--0.35)	283.09(199.88-385.71)	228.8(178.66-296.98)	-0.9(-1.07--0.73)

Myanmar	Male	17.05(15-19.17)	25.39(22.13-28.68)	1.35(1.21-1.49)	1959.82(1750.44-2208.36)	2091.05(1875.46-2343.11)	0.15(0.1-0.19)	10.97(7.65-15.15)	10.7(8.6-13.33)	-0.05(-0.11-0.01)	271.39(189.4-372.79)	252.83(197.09-323.72)	-0.23(-0.29--0.17)
Namibia	Both	19.47(17.32-21.79)	26.92(23.81-30.4)	1.14(0.92-1.36)	1186.68(1069.16-1316.38)	1272.76(1157.84-1395.98)	0.18(0.11-0.24)	6.09(4.02-9)	6.2(4.12-9.43)	-0.35(-0.8-0.09)	131.48(86.66-196.16)	131.79(88.76-188.25)	-0.4(-0.82-0.02)
Namibia	Female	14.99(13.06-17.09)	21.47(18.69-24.55)	1.29(1.05-1.53)	1126.69(1003.55-1262.72)	1185.25(1067.03-1314.03)	0.13(0.07-0.18)	3.86(2.42-6.52)	4.2(2.5-7.84)	-0.16(-0.57-0.26)	84.2(54.88-135.72)	86.89(54.22-149.29)	-0.32(-0.69-0.05)
Namibia	Male	24.92(22-28.02)	34.38(30.15-39.19)	1.11(0.91-1.31)	1260.83(1131.04-1400.67)	1393.51(1261.79-1534)	0.26(0.2-0.33)	9.19(5.81-14.05)	9.37(6.14-13.95)	-0.32(-0.77-0.15)	190.75(120.54-294.46)	195.11(127.95-283.74)	-0.3(-0.76-0.16)
Nepal	Both	15.96(13.97-18.28)	26.5(23.31-30.57)	1.48(1.4-1.57)	1191.45(1065.79-1328.26)	1365.85(1243.55-1510.44)	0.21(0.1-0.32)	4.38(2.8-6.34)	7(4.66-9.93)	1.81(1.48-2.13)	109.35(72.95-152.14)	164(111.37-224.08)	1.48(1.17-1.79)
Nepal	Female	13.94(11.9-16.12)	23.87(20.7-27.57)	1.59(1.5-1.68)	1077.29(949.49-1213.63)	1239.96(1120.51-1370.75)	0.25(0.15-0.35)	4(2.27-5.86)	6.77(3.86-9.52)	2.01(1.57-2.46)	102.68(61.67-146.57)	157.68(95.56-221.19)	1.56(1.14-1.97)
Nepal	Male	17.88(15.57-20.79)	29.37(25.63-33.88)	1.44(1.35-1.53)	1301.6(1152.52-1465.97)	1508.52(1357.79-1675.42)	0.22(0.1-0.33)	4.79(2.95-7.75)	7.25(4.47-11.67)	1.63(1.4-1.85)	115.97(74.82-176.86)	170.85(111.59-260.92)	1.43(1.21-1.64)
Netherlands	Both	28.39(25.97-31.19)	31.56(28-34.97)	0.41(0.36-0.46)	1031.18(944.65-1124.17)	1035.93(949.54-1133.24)	-0.09(-0.15--0.02)	1.4(1.01-1.97)	1.65(1.14-2.32)	0.99(0.59-1.39)	28.65(21.71-36.48)	30.83(23.54-39.97)	0.47(0.27-0.68)
Netherlands	Female	27.36(24.54-30.58)	30.39(26.42-34.69)	0.42(0.37-0.46)	964.78(868.99-1067.49)	963.8(869.74-1068.64)	-0.07(-0.14-0)	1.33(0.94-1.88)	1.57(1.08-2.25)	1.02(0.51-1.53)	27.2(20.8-34.64)	29.17(22.38-37.42)	0.4(0.15-0.66)
Netherlands	Male	29.72(26.31-33.11)	33.01(28.68-37.45)	0.4(0.34-0.45)	1112.79(1012.06-1225.32)	1113.53(1010.67-1224.49)	-0.13(-0.2--0.07)	1.55(1.1-2.17)	1.76(1.23-2.54)	0.76(0.52-1.01)	30.92(23.21-40.09)	32.85(24.72-42.74)	0.44(0.3-0.59)
New Zealand	Both	32.77(29.25-36.55)	37.23(33.58-41.91)	0.38(0.33-0.43)	927.02(848.22-1018.14)	1023.37(937.1-1115.38)	0.23(0.14-0.31)	0.96(0.67-1.34)	1.42(1.02-1.96)	1.57(1.11-2.03)	26.36(20.11-33.98)	34.5(26.3-44.19)	0.96(0.6-1.32)
New Zealand	Female	32.44(28.91-36.67)	36.11(31.76-40.65)	0.29(0.21-0.37)	953.22(859.89-1056.59)	1043.28(951.5-1144.36)	0.2(0.1-0.3)	0.92(0.66-1.26)	1.23(0.87-1.73)	1.57(1.16-1.99)	26.43(20.33-33.66)	31.45(23.86-40.08)	0.82(0.54-1.09)
New Zealand	Male	34.01(29.46-39.07)	38.65(33.8-44.1)	0.4(0.34-0.45)	907.18(823.28-1004.18)	1003.75(911.5-1105.16)	0.23(0.16-0.3)	1.03(0.71-1.48)	1.65(1.18-2.27)	1.46(0.9-2.01)	26.79(20.25-35.31)	38.12(28.93-49.26)	1.03(0.56-1.5)
Nicaragua	Both	35.05(30.43-40.21)	53.9(47.49-60.56)	1.45(1.41-1.49)	1935.15(1751.99-2130.9)	2276.7(2088.22-2477.49)	0.51(0.49-0.53)	10.35(8.06-12.84)	24.57(18.83-31.07)	3.34(2.96-3.71)	254.42(201.29-308.96)	552.02(412.19-706.28)	3.05(2.75-3.36)
Nicaragua	Female	26.77(23.39-30.98)	41.4(36.47-47.26)	1.55(1.52-1.57)	1697.08(1512.78-1908.87)	1864.92(1681.93-2061.96)	0.32(0.3-0.34)	6.32(4.87-8.04)	17.19(13.32-21.62)	3.61(3.01-4.21)	162.33(126.44-201.7)	367.41(279.88-464.39)	3.07(2.69-3.45)
Nicaragua	Male	43.96(37.55-50.96)	68.23(58.89-77.67)	1.45(1.4-1.5)	2200.01(1987.7-2421.98)	2771.14(2510.34-3023.58)	0.71(0.67-0.74)	15.69(12.23-19.5)	33.98(25.16-43.96)	3.14(2.87-3.41)	362.14(286.14-447.11)	777.11(570.31-1005.46)	3.1(2.81-3.38)
Niger	Both	14.88(13.16-16.58)	19.82(17.48-22.45)	1.08(1-1.16)	994.19(899.33-1104.02)	1123.37(1013.55-1241.03)	0.37(0.31-0.43)	8.76(6.23-12.21)	7.51(5.39-10.19)	-0.41(-0.49--0.33)	180.99(127.49-246.63)	153.47(110.13-207.24)	-0.49(-0.59--0.39)
Niger	Female	14.46(12.62-16.42)	19.06(16.51-21.79)	1.07(0.98-1.16)	1010.54(908.44-1132.21)	1126.95(1009.92-1253.67)	0.35(0.29-0.41)	7.52(5.03-11.42)	6.23(4.38-8.7)	-0.71(-0.78--0.64)	160.6(109.66-241.53)	132.23(93.26-179.12)	-0.78(-0.88--0.68)
Niger	Male	15.24(13.48-17.04)	20.54(18.07-23.38)	1.12(1.04-1.2)	981.18(879.7-1091.58)	1119.89(1002.8-1243.93)	0.39(0.32-0.46)	10.05(6.87-14.88)	8.89(5.99-12.93)	-0.15(-0.27--0.03)	200.07(135.06-291.17)	175.65(118.78-253.95)	-0.2(-0.33--0.07)
Nigeria	Both	16.61(14.89-18.37)	22.14(19.77-24.65)	1.16(1.03-1.3)	1043.8(962.92-1128.62)	1134.88(1049.64-1222.69)	0.29(0.25-0.32)	6.65(4.63-9.14)	6.14(4.46-8.1)	-0.56(-0.73--0.38)	128.71(90.55-176.02)	118.66(86.37-156.84)	-0.51(-0.69--0.33)
Nigeria	Female	18.21(16.35-20.25)	22.74(20.4-25.35)	0.89(0.75-1.02)	1061.79(981.07-1149.06)	1138.81(1053.57-1230.8)	0.25(0.22-0.28)	6.26(4.23-8.99)	5.44(3.75-7.33)	-0.85(-1.01--0.69)	124.8(85.57-177.92)	107.73(74.01-146.39)	-0.79(-0.95--0.63)
Nigeria	Male	15.62(13.98-17.22)	21.32(18.92-23.82)	1.24(1.12-1.37)	1025.79(946.09-1111.96)	1129.99(1041.28-1218.25)	0.31(0.28-0.34)	7.14(4.7-10.49)	6.9(4.55-10.06)	-0.35(-0.52--0.17)	134.28(86.91-196.33)	130.76(87.28-193.24)	-0.29(-0.48--0.09)
North Korea	Both	19.11(16.89-21.61)	23.41(20.65-26.51)	0.72(0.53-0.91)	1692.65(1508.13-1910.96)	1647.24(1476.16-1831.59)	-0.16(-0.21--0.12)	5.11(3.9-6.54)	4.79(3.7-6.07)	-0.18(-0.32--0.04)	140.84(107.43-178.4)	134.34(105.06-169.19)	-0.13(-0.22--0.05)
North Korea	Female	17.46(15.25-20.07)	22.39(19.34-25.48)	0.88(0.72-1.05)	1819.52(1591.38-2080.15)	1771.11(1560.81-1982.7)	-0.14(-0.18--0.1)	5.14(3.8-6.71)	4.58(3.42-6.15)	-0.36(-0.5--0.22)	143.91(107.77-182.87)	132.6(99.65-172.66)	-0.26(-0.34--0.18)
North Korea	Male	22.62(19.71-25.59)	25.58(22.23-29.52)	0.43(0.21-0.64)	1545.14(1378.93-1735.2)	1513.79(1354.99-1686.33)	-0.16(-0.22--0.1)	5.25(3.84-7.08)	5.36(3.94-7.2)	0.15(0-0.31)	138.48(102.49-182.97)	139.81(107.48-177.89)	0.09(-0.01-0.18)
Northern Mariana Islands	Both	32.73(28.21-37.76)	40.99(35.94-46.73)	0.6(0.36-0.85)	2146.47(1912.52-2406.6)	2263.18(2044.13-2493.84)	0.09(0-0.17)	16.08(12.95-20.16)	20.04(15.98-24.24)	0.98(0.86-1.1)	367.36(290.7-459.73)	449(354.95-553.99)	0.86(0.76-0.96)
Northern Mariana Islands	Female	27.43(20.68-33.97)	31.8(26.69-37.34)	0.39(-0.02-0.79)	2191.45(1915.58-2491.54)	2208.56(1968.95-2472.37)	-0.03(-0.14-0.08)	17.81(13.58-23.1)	18.68(14.23-24.39)	0.22(0.12-0.32)	425.54(321.07-551.47)	433.87(330.89-562.82)	0.12(0.02-0.21)
Northern Mariana Islands	Male	37.21(32.4-42.49)	50.11(43.86-57.11)	0.83(0.69-0.98)	2121.82(1884.45-2388.36)	2324.78(2093.01-2577.83)	0.18(0.11-0.25)	15(11.81-18.77)	21.72(17.54-26.3)	1.65(1.47-1.82)	330.55(260.42-413.11)	467.24(369.6-573.08)	1.5(1.35-1.64)
Norway	Both	21.11(18.62-23.68)	26.62(23.81-29.77)	0.72(0.64-0.79)	822.17(760.46-885.35)	890.54(823.91-957)	0.09(0.03-0.14)	0.77(0.55-1.06)	1.23(0.88-1.66)	2.21(1.91-2.51)	21.58(16.59-27.61)	24.72(19.27-31.24)	0.67(0.49-0.84)
Norway	Female	19.42(17.17-21.92)	24.94(22.27-27.84)	0.72(0.61-0.83)	732.11(675.56-787.9)	795.44(736.28-857.39)	0.04(-0.06-0.13)	0.6(0.43-0.83)	1.05(0.75-1.42)	2.68(2.31-3.05)	18.62(14.4-23.75)	22.08(17.09-27.9)	0.78(0.6-0.96)
Norway	Male	23.45(20.66-26.32)	28.62(25.56-31.97)	0.65(0.59-0.71)	929.77(858.07-998.48)	989.51(914.33-1062.84)	0.07(0.02-0.11)	1.05(0.75-1.48)	1.53(1.07-2.13)	1.61(1.39-1.83)	26(19.77-33.7)	28.29(21.55-36.36)	0.45(0.29-0.62)
Oman	Both	31.33(27.56-35.32)	63.31(56.62-70.92)	2.35(2.14-2.57)	1352.03(1231.53-1474.92)	1790.76(1645.53-1934.31)	0.91(0.84-0.98)	5.53(3.99-7.61)	6.75(5.18-8.51)	1.08(0.89-1.27)	120.16(89.96-159.98)	142.42(115.43-171.52)	0.86(0.72-1)
Oman	Female	33.08(28.88-37.83)	59.6(52.82-67.18)	2.08(1.76-2.4)	1448.44(1301.32-1602.32)	1814.78(1652.68-1984.11)	0.8(0.7-0.9)	5.25(3.73-7.31)	6.51(4.75-8.48)	1.02(0.85-1.19)	121.34(89.83-161.13)	145.45(115.15-179.28)	0.86(0.71-1)
Oman	Male	30.16(26.21-34.51)	66.69(58.75-75.16)	2.57(2.42-2.71)	1299.28(1180.68-1428.33)	1805.34(1655.07-1962.61)	1.01(0.96-1.06)	6.27(4.39-9.21)	7.36(5.51-9.75)	1(0.79-1.2)	123.96(89.35-177.72)	144.29(113.64-177.04)	0.83(0.69-0.97)
Pakistan	Both	18.11(16.16-20.63)	23.78(21.23-26.77)	1.02(0.97-1.07)	1523.23(1376.18-1685.13)	1596.25(1449.02-1752.67)	0.15(0.12-0.18)	6.31(4.27-9.28)	10.06(7.09-13.52)	1.63(1.36-1.91)	147.9(101.14-209.16)	235.83(169.06-317.3)	1.61(1.33-1.89)
Pakistan	Female	17.66(15.6-19.93)	22.75(20.19-25.67)	0.91(0.88-0.94)	1554.81(1403.19-1734.45)	1612.27(1458.87-1772.68)	0.13(0.11-0.15)	5.59(2.92-8.04)	9.24(5.44-13.13)	1.74(1.46-2.02)	133.87(77.33-189.99)	218.18(131.67-305.06)	1.64(1.35-1.94)
Pakistan	Male	18.5(16.35-21.14)	24.77(22.01-27.98)	1.13(1.06-1.2)	1494.14(1340.27-1657.02)	1578.25(1426.6-1737.3)	0.16(0.11-0.21)	6.89(4.27-11.72)	10.87(7.38-15.91)	1.61(1.34-1.89)	159.03(102.05-256.3)	252.55(174.24-364.73)	1.62(1.35-1.9)
Palestine	Both	41.06(36.04-45.91)	65.89(58.62-73.69)	1.56(1.48-1.64)	1514.63(1379.48-1661.51)	1827.35(1671.08-1987.6)	0.58(0.55-0.61)	14.52(10.61-19.5)	10.93(8.63-13.66)	-0.67(-0.96--0.38)	303.12(224.58-402.17)	233.18(186.39-286.11)	-0.62(-0.89--0.34)
Palestine	Female	41.77(36.32-47.41)	61.21(54.19-69.23)	1.4(1.24-1.57)	1574.79(1426.93-1736.92)	1807.87(1647.63-1979.77)	0.49(0.44-0.54)	13.43(9.57-18.04)	10.02(7.72-12.65)	-0.75(-0.99--0.51)	289.69(208.19-384.21)	217.66(172.93-269.98)	-0.72(-0.95--0.5)
Palestine	Male	40.15(35.08-45.6)	71.21(62.45-80.55)	1.78(1.69-1.86)	1444.2(1312.21-1583.38)	1863.37(1695.15-2031.98)	0.72(0.65-0.79)	16.09(11.64-21.99)	12.63(9.62-15.91)	-0.46(-0.9--0.02)	321.12(233.14-429.8)	255.29(200.83-313.8)	-0.47(-0.88--0.07)

Panama	Both	32.49(28.31-37.42)	46.5(41.64-51.99)	1.07(0.99-1.16)	1822.64(1635.11-2022.98)	2003.09(1821.12-2194.85)	0.22(0.18-0.26)	4.04(3.17-4.91)	7.94(5.73-10.65)	2.4(2.01-2.78)	106.44(85.64-126.67)	188.22(141.42-244.04)	1.99(1.66-2.31)
Panama	Female	29.69(25.77-34.13)	40.55(35.66-46.14)	0.94(0.87-1.02)	1703.62(1510.06-1910.12)	1800.28(1616.71-1993.86)	0.12(0.09-0.15)	3.35(2.6-4.1)	6.46(4.65-8.68)	2.36(1.87-2.85)	90.36(72.48-107.92)	154.92(117.13-199.93)	1.91(1.5-2.31)
Panama	Male	35.21(30.36-40.93)	52.56(46.06-59.63)	1.19(1.1-1.29)	1937.49(1741.37-2147.58)	2212.49(2007.7-2436.4)	0.32(0.28-0.37)	4.74(3.71-5.84)	9.63(6.93-13.15)	2.49(2.15-2.83)	122.21(97.08-147.94)	223.75(167.59-293.85)	2.09(1.8-2.38)
Papua New Guinea	Both	10.85(9.42-12.49)	14.81(12.73-17)	0.89(0.72-1.06)	1728.35(1521.46-1953.7)	1756.53(1567.01-1961.05)	-0.04(-0.07-0)	3.95(2.98-5.04)	4.72(3.47-6.28)	0.54(0.39-0.69)	112.19(85.51-141.95)	133.46(100.65-175.9)	0.52(0.4-0.65)
Papua New Guinea	Female	8.43(7.16-10.02)	11.56(9.78-13.49)	0.89(0.72-1.06)	1753.63(1511.81-2010.91)	1743.45(1522.23-1966.43)	-0.09(-0.12--0.06)	2.78(1.93-3.78)	3.31(2.34-4.51)	0.55(0.36-0.73)	85.92(62.32-112.22)	101.62(76.53-134.68)	0.53(0.39-0.66)
Papua New Guinea	Male	13.22(11.46-15.19)	17.79(15.16-20.48)	0.84(0.67-1.01)	1706.32(1500.98-1945.84)	1769.48(1566.14-1994.69)	0.01(-0.03-0.05)	5.17(3.79-6.82)	6.12(4.43-8)	0.5(0.35-0.64)	137.52(100.46-182)	163.03(119.83-216.63)	0.49(0.37-0.61)
Paraguay	Both	24.6(21.63-28.14)	39.26(34.57-43.83)	1.47(1.41-1.53)	1201.79(1085.5-1321.44)	1404.2(1288.43-1519.83)	0.46(0.41-0.5)	4.31(3.36-5.29)	10.37(7.55-13.64)	3.76(3.5-4.02)	99.13(79.01-119.21)	230.5(169.86-302.18)	3.52(3.28-3.75)
Paraguay	Female	23.01(20.06-26.64)	35.94(31.57-40.32)	1.42(1.37-1.48)	1129.65(1021.62-1254.48)	1292.4(1176.19-1412.7)	0.42(0.38-0.46)	3.7(2.85-4.7)	8.95(6.63-11.79)	3.75(3.43-4.07)	88.26(68.53-108.14)	202.25(152.86-263.95)	3.45(3.15-3.75)
Paraguay	Male	26.31(22.83-30.32)	42.76(37.08-48.58)	1.5(1.43-1.57)	1277.14(1142.32-1406.92)	1520.04(1371.26-1664.26)	0.48(0.43-0.53)	5.07(3.95-6.29)	12.11(8.47-16.31)	3.78(3.56-4)	111.85(87.49-135.39)	262.46(187.99-351.2)	3.57(3.38-3.76)
Peru	Both	18.3(16.2-20.54)	35.47(31.43-39.8)	2.33(2.2-2.45)	1559.66(1187.69-2011.83)	1832.88(1434.15-2320.3)	0.5(0.48-0.53)	5.93(4.53-7.55)	6.54(4.51-9.11)	0.44(0.21-0.67)	124.96(94.88-156.74)	133.95(97.28-180.91)	0.33(0.14-0.52)
Peru	Female	19.18(16.75-21.66)	37.53(32.88-42.71)	2.36(2.19-2.53)	1371.05(1056.04-1763.14)	1662.45(1327.57-2084.08)	0.61(0.58-0.64)	5.62(4.24-7.1)	6.73(4.59-9.37)	0.38(0.1-0.66)	118.4(89.56-148.57)	137.8(99.49-185.45)	0.31(0.08-0.55)
Peru	Male	17.33(15.18-19.81)	33.28(28.91-37.91)	2.3(2.22-2.37)	1753.73(1307.4-2296.34)	2012.53(1531.52-2579)	0.42(0.39-0.46)	6.27(4.72-8.09)	6.31(4.3-9.05)	0.5(0.26-0.73)	131.91(98.59-168.03)	129.65(90.03-177.29)	0.34(0.14-0.54)
Philippines	Both	23.81(21.55-26.12)	33.68(30.46-37.06)	1.07(1.04-1.11)	2028.91(1852.49-2231.3)	2142.08(1964.22-2324.95)	0.09(0.05-0.12)	13.2(10.81-15.68)	15.74(12.37-19.58)	0.96(0.69-1.23)	285.51(232.67-341.66)	382.99(294.27-478.45)	1.41(1.13-1.69)
Philippines	Female	23.79(21.5-26.14)	33.87(30.56-37.25)	1.09(1.04-1.13)	1969.33(1793.94-2167.77)	2074.7(1902.89-2249.33)	0.11(0.08-0.14)	12.79(10.5-15.59)	14.18(10.62-18.42)	0.72(0.42-1.01)	269.08(218.1-326.14)	340.22(253.82-452.47)	1.19(0.92-1.47)
Philippines	Male	23.81(21.58-26.16)	33.17(30.1-36.49)	1.03(1-1.06)	2093.44(1907.83-2289.13)	2212.52(2031.62-2412.86)	0.06(0.03-0.1)	13.66(10.93-16.87)	17.58(13.11-23.5)	1.24(0.98-1.49)	302.82(236.76-375.63)	429.93(315.83-578.41)	1.63(1.35-1.91)
Poland	Both	20.63(17.93-23.89)	27.29(23.93-31.3)	0.28(0.04-0.52)	1323.35(1214.59-1438.84)	1312.59(1207.07-1418.5)	-0.22(-0.29--0.15)	2.33(1.77-2.86)	1.53(1.13-1.99)	-1.23(-1.86--0.6)	60.07(46.77-73.14)	38.91(30.09-48.52)	-1.26(-1.69--0.83)
Poland	Female	18.9(16.37-21.86)	26.33(23.02-30.33)	0.32(0.03-0.61)	1329.07(1214.4-1453.96)	1332.54(1222.99-1440.37)	-0.17(-0.24--0.1)	2.04(1.59-2.49)	1.38(0.98-1.85)	-1.17(-1.84--0.5)	53.29(41.54-64.38)	35.5(26.95-45.43)	-1.22(-1.67--0.78)
Poland	Male	23.26(20.22-26.91)	28.74(25.34-32.84)	0.24(0.06-0.42)	1331.34(1220.55-1447.61)	1294.1(1189.18-1401.68)	-0.29(-0.36--0.22)	2.82(2.15-3.57)	1.77(1.26-2.4)	-1.39(-1.97--0.81)	70.02(53.43-86.25)	43.5(32.31-56.78)	-1.36(-1.77--0.93)
Portugal	Both	27.45(24.03-31.26)	30.97(26.85-35.07)	0.35(0.26-0.43)	965.16(871.75-1063.56)	955.28(872.26-1048.04)	-0.09(-0.13--0.04)	2.54(1.84-3.4)	2.85(2.03-3.98)	0.55(0.04-1.07)	51.95(39.09-65.78)	49.51(37.64-63.09)	0.11(-0.45-0.66)
Portugal	Female	27.25(23.7-31.23)	31.57(27.55-35.72)	0.44(0.36-0.52)	926.41(831.2-1042.18)	921.73(837.69-1019.6)	-0.04(-0.09-0.01)	2.11(1.53-2.78)	2.62(1.83-3.69)	0.95(0.4-1.5)	44.91(34.38-56.65)	45.99(35.21-58.83)	0.39(-0.22-1)
Portugal	Male	27.88(23.86-31.87)	30.36(25.74-35.29)	0.23(0.14-0.33)	1013.92(911.79-1120.89)	993.66(891.26-1099.44)	-0.15(-0.19--0.11)	3.32(2.34-4.57)	3.21(2.27-4.5)	-0.02(-0.49-0.45)	62.63(45.39-80.55)	54.39(40.67-69.97)	-0.27(-0.75-0.22)
Puerto Rico	Both	29.49(26.13-33.29)	43.23(37.78-48.6)	1.35(1.13-1.57)	1525.24(1383.85-1691.63)	1727.22(1569.92-1889.9)	0.44(0.35-0.53)	8.39(6.79-9.97)	8.04(5.77-10.58)	0.45(0.12-0.77)	184.77(151.29-215.58)	192.57(146.94-246.5)	0.63(0.41-0.84)
Puerto Rico	Female	27.11(23.61-31.19)	38.47(33-43.94)	1.22(1.01-1.44)	1441.35(1291.78-1615.02)	1583.64(1423.89-1749.1)	0.34(0.26-0.42)	7.08(5.73-8.53)	6.68(4.81-8.84)	0.34(-0.01-0.69)	152.49(125.15-179.34)	158.15(122.37-200.38)	0.57(0.35-0.78)
Puerto Rico	Male	32.22(27.97-36.74)	48.77(42.36-55.83)	1.47(1.24-1.7)	1621.96(1464.67-1795.16)	1898.18(1725.21-2089.55)	0.54(0.44-0.64)	9.96(8.05-11.82)	9.79(7.02-12.97)	0.59(0.28-0.9)	222.3(180.55-259.86)	234.35(176.69-303.73)	0.7(0.49-0.92)
Qatar	Both	46.62(41.4-52.74)	77.18(68.98-86.16)	1.79(1.6-1.99)	1604.71(1463.9-1758.85)	1995.38(1831.88-2172.61)	0.76(0.67-0.85)	16.92(12.4-29.04)	15.81(11.98-20.87)	0.09(-0.21-0.39)	303.37(228.43-485.64)	256.95(199.91-333.83)	-0.34(-0.66--0.01)
Qatar	Female	54.11(47.16-61.38)	71.69(63.71-80.03)	1.08(0.79-1.37)	1794.45(1625.77-1978.56)	2026.37(1843.73-2208.05)	0.51(0.36-0.66)	19.35(13.24-37.84)	24.18(17.59-32.04)	1.29(0.91-1.67)	367.71(261.1-674.29)	380.86(289.39-495.68)	0.5(0.12-0.88)
Qatar	Male	44.09(38.62-50.84)	79.43(70.33-89.31)	2.1(1.93-2.28)	1517.51(1370.89-1674.09)	1989.69(1816.17-2176.62)	0.94(0.87-1.01)	15.63(10.85-24.77)	13.05(9.49-17.69)	-0.42(-0.8--0.04)	270.01(194.45-396.69)	215.01(162.17-281.47)	-0.67(-0.99--0.36)
Republic of Congo	Both	12.67(11.17-14.23)	17.84(15.68-20.1)	1.34(1.22-1.46)	1139.56(1011.95-1273.41)	1167.95(1047.99-1284.38)	0.05(0.02-0.08)	9.93(6.46-13.47)	8.72(5.54-12.15)	-0.61(-0.72--0.49)	206.17(135.2-286.49)	171.39(113.14-236.88)	-0.83(-0.95--0.7)
Republic of Congo	Female	11.14(9.69-12.79)	16.7(14.4-19.12)	1.54(1.41-1.67)	1116.69(978.38-1266.79)	1140.01(1012.67-1266.28)	0.06(0.03-0.09)	8.17(3.63-12.09)	8.05(3.53-12.29)	-0.09(-0.19-0.01)	170.8(79.35-254.54)	160.82(76.32-246.35)	-0.26(-0.39--0.14)
Republic of Congo	Male	14.68(12.85-16.57)	19.13(16.71-21.65)	1.07(0.96-1.17)	1166.98(1034.68-1312.24)	1198(1071.6-1335.5)	0.03(0-0.07)	12.29(8.87-16.65)	9.52(6.7-13.07)	-1.19(-1.35--1.03)	253.01(181.38-346.97)	184.65(131.51-252.77)	-1.43(-1.61--1.25)
Romania	Both	15.71(13.94-17.88)	26.63(23.86-29.58)	1.73(1.58-1.88)	1192.12(1067.78-1329.6)	1262.18(1148-1391.67)	0.1(0.05-0.15)	1.04(0.64-1.41)	1.2(0.79-1.71)	1.26(0.66-1.86)	29.06(19.74-37.29)	40.45(30.04-52.57)	1.7(1.31-2.1)
Romania	Female	14.76(12.84-16.97)	23.27(20.59-26.2)	1.41(1.24-1.58)	1186.75(1042.26-1350.83)	1224.93(1092.33-1374.18)	0(-0.05-0.05)	1.07(0.63-1.46)	1.1(0.72-1.58)	0.57(-0.12-1.27)	28.92(18.98-37.44)	36.88(27.47-47.97)	1.19(0.75-1.63)
Romania	Male	16.97(14.78-19.35)	31.22(27.52-35.26)	2.1(1.97-2.23)	1204.92(1073.04-1342.57)	1317.38(1189.98-1445.73)	0.23(0.18-0.27)	0.99(0.63-1.36)	1.33(0.87-1.91)	2.08(1.59-2.57)	29.17(20.1-38.29)	44.99(32.97-59.16)	2.26(1.91-2.61)
Russia	Both	14.55(13-16.27)	21.46(19.17-24.17)	1.46(1.28-1.63)	1805.92(1656.87-1966.1)	1810.83(1666.2-1960.12)	-0.04(-0.08-0)	0.57(0.41-0.77)	0.67(0.47-0.93)	0.48(0.24-0.73)	22.04(16.76-28.21)	23.85(18.18-30.47)	0.09(-0.09-0.28)
Russia	Female	15.13(13.55-16.9)	19.39(17.33-21.67)	0.84(0.63-1.05)	1722.92(1578.6-1875.49)	1671.96(1536.92-1810.93)	-0.14(-0.18--0.11)	0.53(0.37-0.71)	0.63(0.44-0.89)	0.56(0.34-0.77)	21.05(15.95-26.86)	21.91(16.43-28.29)	-0.04(-0.21-0.12)
Russia	Male	14.11(12.54-15.92)	25.32(22.42-28.98)	2.38(2.23-2.52)	1929.45(1767.05-2105.8)	2004.23(1842.91-2168.74)	0.1(0.04-0.15)	0.73(0.52-0.99)	0.77(0.53-1.07)	0.08(-0.2-0.37)	24.51(18.63-31.54)	27.23(20.73-35.27)	0.21(0-0.42)
Rwanda	Both	12.03(10.69-13.51)	15.89(14.12-17.91)	1.16(1.01-1.3)	1035.27(921.68-1157.34)	1052.83(946.77-1162.71)	0(-0.04-0.05)	8.8(6.52-11.43)	6.87(4.9-9.22)	-1.56(-1.88--1.25)	185.72(138.66-239.49)	134.63(99.3-175.77)	-1.92(-2.27--1.57)
Rwanda	Female	10.07(8.88-11.44)	14.6(12.76-16.64)	1.5(1.36-1.64)	1005.05(883.36-1132.31)	1018.45(907.83-1141.79)	0.01(-0.03-0.05)	7.31(5.08-9.81)	6.35(4.21-8.79)	-1.02(-1.31--0.73)	158.36(112.28-207.18)	124.19(88.18-166.09)	-1.47(-1.8--1.15)
Rwanda	Male	14.39(12.73-16.24)	17.67(15.43-20.11)	0.87(0.75-0.99)	1071.25(951.77-1204.1)	1094.55(980.83-1223.82)	0(-0.04-0.05)	10.7(7.57-14.55)	7.65(5.28-10.5)	-2(-2.34--1.66)	219.99(156.82-296.55)	149.84(107.33-201.68)	-2.28(-2.66--1.9)
Saint Lucia	Both	25.68(22.62-29.06)	38.74(34.28-44.1)	1.33(1.18-1.48)	1497.15(1352.87-1659.44)	1653.06(1509.05-1804.8)	0.29(0.24-0.33)	9.05(7.37-10.85)	9.61(7.43-11.93)	0.22(-0.04-0.48)	206.07(165.87-244.39)	225.56(176.78-280.08)	0.41(0.21-0.61)

Saint Lucia	Female	22.77(19.83-26.09)	34.9(30.29-39.87)	1.35(1.21-1.49)	1420.23(1269.43-1592.65)	1540.11(1391.18-1687.84)	0.24(0.2-0.28)	7.2(5.82-8.72)	7.83(5.82-10.02)	0.2(-0.09-0.5)	169.98(135.7-205.22)	188.7(144.96-238.41)	0.35(0.12-0.58)
Saint Lucia	Male	29.29(25.47-33.56)	42.85(37.54-49.04)	1.25(1.09-1.4)	1593.33(1426.39-1763.45)	1774.29(1604.86-1946.73)	0.3(0.25-0.35)	12.11(9.73-14.6)	11.98(9.29-15.02)	0.02(-0.23-0.27)	256.67(205.96-304.01)	268.28(208.62-333.32)	0.33(0.14-0.52)
Saint Vincent	Both	24.04(20.9-27.11)	34.63(30.59-38.74)	1.22(1.1-1.35)	1460.37(1314.6-1620.46)	1613.12(1468.6-1761.62)	0.29(0.25-0.33)	7.31(5.89-8.8)	10.55(8.35-12.86)	1.58(1.32-1.84)	170.92(137.07-206.08)	245.63(195.68-300.45)	1.44(1.27-1.61)
Saint Vincent	Female	22.19(19.13-25.52)	32.25(28.32-36.5)	1.24(1.12-1.35)	1401.62(1245.76-1573.71)	1516.48(1358.1-1664.75)	0.24(0.2-0.28)	6.72(5.36-8.22)	9.72(7.58-12.06)	1.76(1.38-2.13)	158.84(127.23-192.75)	229.07(181.61-282.21)	1.54(1.31-1.78)
Saint Vincent	Male	26.28(22.76-29.77)	36.86(32.11-41.93)	1.13(1-1.26)	1532.29(1373.45-1700.75)	1703.01(1544.75-1871.74)	0.29(0.24-0.34)	8.4(6.79-10.21)	11.54(9.06-14.22)	1.32(1.12-1.51)	187.94(148.89-226.98)	262.4(207.95-320.58)	1.26(1.12-1.41)
Samoa	Both	24.53(21.49-27.8)	33.82(30.02-37.99)	0.93(0.82-1.04)	2034.72(1815.62-2288.19)	2142.66(1939.97-2376.67)	0.08(0.03-0.12)	13.48(10.06-17.82)	15.75(12.24-20.03)	0.45(0.24-0.66)	319.3(238.58-427.82)	377.18(290.94-480.31)	0.49(0.28-0.69)
Samoa	Female	21.97(18.88-25.41)	29.79(25.9-33.93)	0.86(0.73-0.98)	2092.05(1841.96-2370.43)	2164.77(1927.59-2429.65)	0.03(-0.01-0.08)	12.77(8.72-17.94)	14.99(11.08-20.16)	0.25(-0.07-0.57)	316.57(223.49-441.73)	379.92(281.72-500.41)	0.37(0.07-0.67)
Samoa	Male	27.72(24.33-31.47)	38.51(34.01-43.87)	0.99(0.88-1.09)	1985.85(1770.88-2243.29)	2128.44(1919.03-2394.02)	0.13(0.08-0.17)	14.75(11.02-19.63)	16.86(12.92-21.82)	0.61(0.48-0.74)	327.01(244.95-435.59)	378.72(289.23-490.81)	0.61(0.49-0.74)
Sao Tome and Principe	Both	20.27(17.99-22.75)	29.24(26-32.86)	1.33(1.27-1.39)	1158.71(1043.14-1288.68)	1304.31(1185.73-1430.8)	0.39(0.36-0.41)	10.51(7.84-13.69)	13.14(9.47-17.24)	0.72(0.61-0.83)	211.81(156.34-274.79)	261.51(189.33-341.62)	0.6(0.43-0.76)
Sao Tome and Principe	Female	21.13(18.55-24.09)	30.81(27.01-35.01)	1.36(1.3-1.41)	1183.78(1061.36-1315.33)	1334.19(1212.39-1470.35)	0.41(0.39-0.43)	10.37(7.69-13.58)	13.72(9.54-18.13)	0.77(0.59-0.95)	217.73(159.41-282.63)	278.22(196.91-366.29)	0.57(0.34-0.81)
Sao Tome and Principe	Male	19.4(17.22-21.98)	27.85(24.37-31.93)	1.33(1.26-1.41)	1132.21(1003.89-1262.45)	1270.81(1140.87-1403.12)	0.35(0.32-0.38)	10.95(7.97-14.93)	12.47(8.52-16.86)	0.61(0.52-0.7)	207.44(146.6-274.92)	244.25(170.48-326.91)	0.64(0.55-0.72)
Saudi Arabia	Both	42.25(37.43-47.77)	78.37(70.88-86.26)	2.09(1.96-2.21)	1514.01(1384.98-1660.65)	2068.44(1912.38-2240.22)	1.06(1.03-1.09)	15.15(11.02-20.41)	16.56(12.09-21.68)	0.55(0.29-0.82)	324.27(236.09-439.53)	349.64(261.69-456.77)	0.44(0.18-0.7)
Saudi Arabia	Female	45.48(39.96-51.84)	71.26(63.65-80.1)	1.61(1.39-1.84)	1633.73(1479.55-1806.55)	2064.06(1887.9-2261.1)	0.88(0.8-0.97)	13.5(9.88-18.42)	16.55(11.59-22.9)	0.69(0.43-0.94)	306.78(224.06-413.62)	360.61(263.96-487.93)	0.54(0.26-0.81)
Saudi Arabia	Male	40.2(35.25-45.61)	82.99(73.82-92.32)	2.43(2.37-2.49)	1436.93(1303.16-1575.51)	2075.1(1907.77-2259.6)	1.19(1.14-1.24)	16.78(11.75-23.05)	16.68(11.96-22.76)	0.42(0.12-0.72)	341.54(240.62-469.69)	343.29(251.89-451.65)	0.35(0.08-0.63)
Senegal	Both	17.26(15.31-19.37)	21.73(19.35-24.18)	0.82(0.75-0.89)	1126.67(1011.61-1246.54)	1176.05(1062.01-1293.95)	0.1(0.07-0.13)	11.01(7.84-15.01)	9.82(7.11-13.4)	-0.33(-0.46--0.21)	217.91(155.56-295.21)	193.03(138.6-264.76)	-0.32(-0.48--0.16)
Senegal	Female	16.77(14.58-19.02)	20.52(17.93-23.22)	0.72(0.66-0.78)	1120.82(997.62-1245.87)	1150.08(1027.97-1271.57)	0.07(0.04-0.1)	10.11(6.92-15.89)	8.6(6.01-12.93)	-0.66(-0.8--0.52)	204.63(139.16-316.67)	170.99(119.24-254.14)	-0.69(-0.86--0.51)
Senegal	Male	17.61(15.47-19.8)	22.98(20.23-25.85)	0.96(0.88-1.04)	1135.01(1007.53-1273.45)	1204.35(1082-1342.43)	0.13(0.1-0.17)	11.88(8.3-16.92)	11.18(7.93-15.41)	0(-0.13-0.14)	229.94(160.78-324.64)	216.85(151.82-304.24)	0.04(-0.13-0.21)
Serbia	Both	19.16(16.92-21.55)	32.71(28.73-36.82)	1.92(1.85-1.98)	1348.19(1212.05-1483.45)	1441.7(1318.43-1575.33)	0.19(0.17-0.2)	3.09(2.2-4.15)	3.6(2.5-5.04)	0.15(-0.05-0.34)	72.68(53.49-94.46)	78.32(56.45-103.68)	-0.16(-0.33-0.01)
Serbia	Female	17.02(14.7-19.44)	29.43(25.34-33.72)	1.93(1.88-1.99)	1257.17(1115.8-1411.59)	1356.71(1218.64-1499.77)	0.24(0.21-0.26)	2.62(1.85-3.61)	3.29(2.25-4.55)	0.23(0.02-0.45)	62.74(45.61-82.72)	70.69(51.42-93.6)	-0.19(-0.4-0.01)
Serbia	Male	21.74(18.94-24.72)	36.82(32.03-41.84)	1.94(1.85-2.02)	1455.1(1300.49-1612.72)	1543.4(1411.91-1692.14)	0.15(0.14-0.17)	3.7(2.55-5.37)	4.01(2.66-5.83)	0.07(-0.13-0.27)	84.78(60.61-116.54)	87.6(61.56-118)	-0.12(-0.28-0.04)
Seychelles	Both	28.56(24.9-32.4)	43.11(38.08-48.01)	1.42(1.3-1.54)	2109.12(1890.99-2366.05)	2279.25(2069.36-2512.56)	0.22(0.2-0.23)	11.42(9.25-13.59)	17.33(13.96-20.68)	0.96(0.55-1.38)	264.21(215.62-313.75)	372.98(305.03-442.58)	0.73(0.36-1.09)
Seychelles	Female	27.67(23.84-31.74)	42.83(37.26-48.49)	1.51(1.39-1.62)	2029.45(1801.66-2295.27)	2179.47(1966.45-2423.39)	0.22(0.21-0.23)	9.75(7.89-11.87)	16.66(12.91-20.78)	1.46(1.01-1.92)	224.43(183-271.48)	350.46(278.8-429.28)	1.23(0.85-1.62)
Seychelles	Male	29.48(25.64-33.71)	43.23(37.65-48.51)	1.3(1.18-1.43)	2204.91(1965.63-2469.9)	2373.94(2130.62-2619.65)	0.18(0.16-0.2)	13.64(10.6-16.64)	17.65(13.84-21.8)	0.3(-0.09-0.69)	312.17(244.59-379.49)	390.48(305.18-477.09)	0.16(-0.2-0.52)
Sierra Leone	Both	14.97(13.24-16.8)	20.29(17.78-22.79)	1.09(1-1.17)	998.16(908.34-1100.78)	1124.46(1021.39-1231.37)	0.38(0.35-0.41)	7.99(5.8-10.52)	7.43(5.41-10.02)	-0.06(-0.16-0.03)	159.22(113.57-207.13)	152.78(111.06-206.6)	0.06(-0.04-0.17)
Sierra Leone	Female	14.85(12.99-16.92)	20.39(17.89-23.3)	1.16(1.08-1.25)	998.5(893.64-1112.39)	1128.67(1018.85-1245.47)	0.42(0.39-0.45)	6.95(4.83-9.83)	6.87(4.77-9.48)	0.11(0.01-0.2)	140.81(99.25-195.96)	145.63(100.41-202.48)	0.29(0.18-0.39)
Sierra Leone	Male	14.99(13.09-17.06)	20.28(17.44-23.05)	1.07(0.98-1.16)	999.6(897.16-1116.66)	1122.05(1010.66-1241.77)	0.34(0.31-0.37)	8.96(6.29-12.16)	8.04(5.59-11.17)	-0.14(-0.24--0.03)	176.07(122.15-238.06)	160.41(111.66-221.84)	-0.08(-0.19-0.03)
Singapore	Both	34.82(32.18-37.53)	39.98(36.04-44.29)	0.51(0.45-0.56)	1872.31(1703.77-2052.45)	1833.96(1669.19-2002.01)	-0.1(-0.15--0.06)	6.78(5.78-7.84)	4.52(3.6-5.38)	0.07(-0.4-0.55)	150.4(128.31-172.54)	98.49(82.28-115.58)	-0.09(-0.53-0.35)
Singapore	Female	29.51(27.01-32.2)	36.41(32.24-40.75)	0.66(0.54-0.78)	1795.76(1609.96-2004.58)	1754.53(1581.45-1942.41)	-0.11(-0.17--0.04)	6.85(5.83-7.95)	4.74(3.7-5.72)	0.22(-0.29-0.74)	151.93(129.58-175.25)	101.17(83.35-118.45)	-0.03(-0.48-0.42)
Singapore	Male	40.99(37.78-44.75)	43.7(39.02-48.89)	0.33(0.29-0.38)	1972.1(1782.61-2166.38)	1916.38(1731.45-2098.73)	-0.13(-0.16--0.1)	6.87(5.76-8.05)	4.27(3.44-5.16)	-0.18(-0.63-0.27)	150.18(125.85-175.55)	95.44(78.96-112.83)	-0.2(-0.63-0.23)
Slovakia	Both	20.35(17.96-23.09)	29.77(25.79-33.93)	1.27(1.22-1.32)	1300.88(1174.84-1445.95)	1346.93(1228.31-1473.38)	0.06(0.04-0.08)	3.03(2.21-3.84)	2.11(1.44-2.96)	0.03(-0.5-0.57)	77.34(57.93-96.09)	53.58(40.18-70.47)	-0.32(-0.76-0.14)
Slovakia	Female	18.74(16.14-21.81)	26.97(23.37-31.16)	1.18(1.12-1.24)	1263.15(1120.68-1421.86)	1299.16(1166.58-1429.67)	0.05(0.03-0.07)	2.72(2-3.44)	2.03(1.37-2.83)	0.11(-0.29-0.51)	68.32(50.99-85.82)	49.93(37.15-65.59)	-0.3(-0.6-0.01)
Slovakia	Male	22.55(19.71-25.9)	33.8(29.02-38.65)	1.39(1.34-1.44)	1355.61(1217.37-1508.41)	1415.23(1285.04-1558.95)	0.09(0.07-0.11)	3.48(2.48-4.58)	2.21(1.48-3.12)	-0.11(-0.81-0.6)	88.82(64.01-113.43)	57.92(42.69-77.52)	-0.35(-0.95-0.26)
Slovenia	Both	18.52(16.15-20.95)	28.45(24.96-31.77)	1.58(1.53-1.62)	1244.65(1122.22-1386.57)	1290.44(1179.5-1413.91)	0.07(0.05-0.09)	1.19(0.85-1.62)	0.93(0.61-1.36)	-0.35(-0.59--0.12)	29.99(23.11-38.33)	26.58(19.7-35.54)	-0.23(-0.4--0.05)
Slovenia	Female	17.28(14.97-19.75)	25.36(22-28.82)	1.37(1.32-1.43)	1216.92(1079.85-1366.1)	1245.99(1121.59-1382.64)	0.03(0.01-0.06)	1.1(0.78-1.52)	0.85(0.56-1.23)	-0.47(-0.72--0.21)	27.47(21.05-35.41)	24.52(18.12-32.54)	-0.28(-0.44--0.12)
Slovenia	Male	20.56(17.88-23.37)	32.53(28.18-36.83)	1.73(1.66-1.8)	1293.76(1159.53-1447.49)	1349.62(1223.78-1485.63)	0.09(0.07-0.11)	1.35(0.95-1.86)	1.09(0.72-1.6)	-0.1(-0.39-0.19)	33.94(25.98-43.48)	29.62(21.82-39.78)	-0.19(-0.41-0.04)
Solomon Islands	Both	18.2(15.79-20.94)	21.21(18.37-24.3)	0.38(0.28-0.49)	1900.38(1684.05-2143.86)	1925.26(1720.05-2147.36)	-0.05(-0.08--0.01)	13.41(9.44-18.53)	8.2(6.25-10.4)	-1.9(-2.32--1.46)	395.87(273.36-541.96)	252.19(187.96-323.89)	-1.76(-2.15--1.36)
Solomon Islands	Female	14.7(12.49-17.39)	16.96(14.51-19.85)	0.37(0.27-0.48)	1950.49(1700.07-2226.24)	1928.84(1711.89-2173.18)	-0.11(-0.13--0.08)	14.57(8.95-21.36)	8.21(6.11-10.84)	-2.24(-2.74--1.74)	441.01(277.08-631.57)	258.99(193.21-336.96)	-2.09(-2.54--1.64)
Solomon Islands	Male	20.73(17.89-23.68)	25.24(21.81-29.01)	0.53(0.42-0.64)	1851.86(1626.26-2103.97)	1920.71(1702.4-2163.46)	0(-0.04-0.05)	12.48(8.39-19.25)	8.15(6.22-10.49)	-1.6(-1.98--1.22)	357.26(238.55-538.06)	245.33(181.39-319.61)	-1.44(-1.79--1.08)
Somalia	Both	12.11(10.84-13.58)	14.08(12.6-15.72)	0.58(0.49-0.67)	1027.12(917.3-1152.95)	1054.77(948.32-1177.78)	0.05(0.03-0.08)	8.49(5.95-11.86)	8.18(5.75-11.16)	0.04(-0.01-0.09)	180.61(127.7-249.75)	171.59(120.84-235.94)	-0.04(-0.08-0)
Somalia	Female	10.36(9.12-11.68)	12.64(11.17-14.28)	0.74(0.65-0.84)	998.07(885.29-1135.59)	1019.61(906.84-1148.79)	0.05(0.03-0.08)	6.77(4.05-10.71)	7.19(4.79-10.68)	0.54(0.42-0.65)	147.57(91.31-230.51)	151.15(101.64-223.91)	0.36(0.27-0.45)

Somalia	Male	14.16(12.58-15.96)	16.14(14.26-18.12)	0.5(0.44-0.57)	1059.31(944.43-1192.5)	1094.89(980.4-1228.27)	0.06(0.03-0.08)	10.56(7.23-14.8)	9.76(6.81-13.43)	-0.25(-0.29--0.22)	219.38(153.92-304.92)	202.39(142.74-277.92)	-0.27(-0.31--0.23)
South Africa	Both	21.72(19.39-24.3)	30.89(27.89-34.32)	1.13(0.95-1.32)	1280.38(1177.2-1389.71)	1401.84(1290.69-1509.61)	0.25(0.19-0.31)	4.47(3.37-5.8)	7.55(5.86-9.44)	2.53(2.16-2.9)	101.51(77.65-127.19)	154.13(119.37-188.18)	2.13(1.79-2.46)
South Africa	Female	17.85(15.81-20.1)	25.99(23.2-29.19)	1.23(1.05-1.41)	1242.11(1137.6-1354.29)	1339.95(1230.39-1449.68)	0.22(0.17-0.27)	3.47(2.6-4.64)	6.22(4.74-7.82)	2.75(2.4-3.11)	81.08(62.45-101.97)	124.87(97.78-153.98)	2.21(1.92-2.51)
South Africa	Male	27.06(24.27-30.13)	37.7(34.07-41.66)	1.04(0.88-1.2)	1336.84(1230.38-1448.4)	1490.74(1376.45-1607.72)	0.29(0.22-0.35)	6.01(4.45-7.96)	9.75(7.49-12.34)	2.36(1.94-2.79)	130.28(97.88-167.33)	196.08(150.22-243.54)	2.07(1.66-2.48)
South Korea	Both	28.61(25.21-32.59)	32.36(29.26-35.37)	0.39(0.3-0.48)	1273.05(1146.93-1403.66)	1241.2(1150.53-1339.9)	-0.16(-0.24--0.07)	5(4.28-5.75)	4.5(3.73-5.26)	-0.17(-0.36-0.02)	109.79(94.08-123.8)	83.44(72.36-94.89)	-0.79(-0.92--0.65)
South Korea	Female	25.63(22.43-29.49)	29.67(26.14-33.11)	0.46(0.38-0.54)	1252.17(1108.22-1402.7)	1202.21(1100.63-1319.76)	-0.22(-0.31--0.13)	4.03(3.44-4.67)	3.81(3.02-4.59)	-0.28(-0.58-0.03)	89.8(76.9-102.33)	69.55(58.33-80.48)	-0.93(-1.16--0.71)
South Korea	Male	33.25(28.92-38.31)	35.55(31.94-39.48)	0.2(0.1-0.3)	1321.27(1192.92-1452.43)	1286.75(1183.9-1393.29)	-0.16(-0.24--0.08)	7.02(5.93-8.25)	5.6(4.61-6.62)	-0.23(-0.41--0.05)	143.15(121.73-164.04)	101.34(86.65-116.39)	-0.79(-0.9--0.68)
South Sudan	Both	12.74(11.37-14.13)	15.05(13.35-16.78)	0.59(0.54-0.64)	1027.81(916.99-1155.61)	1062.56(953.81-1182.86)	0.07(0.04-0.09)	7.43(5.02-10.68)	7.53(5.16-10.41)	0.06(0.04-0.08)	148.71(101.01-212.15)	146.15(98.14-201.98)	-0.07(-0.1--0.04)
South Sudan	Female	10.96(9.65-12.41)	13.45(11.8-15.38)	0.72(0.65-0.78)	996.12(877.82-1135.97)	1026.25(900-1154.38)	0.08(0.06-0.1)	5.98(3.9-9.26)	6.67(4.48-9.72)	0.47(0.41-0.54)	120.82(82.01-182.52)	130.19(90.63-184.46)	0.33(0.25-0.41)
South Sudan	Male	14.08(12.49-15.75)	16.42(14.48-18.46)	0.56(0.51-0.61)	1054.17(931.29-1184.5)	1100.44(976.97-1232.01)	0.08(0.05-0.1)	8.74(5.72-12.95)	8.24(5.21-12.21)	-0.23(-0.27--0.19)	171.71(112.19-248.77)	159.62(101.03-235.49)	-0.3(-0.34--0.26)
Spain	Both	31.46(28.04-34.82)	30.62(27.11-34.09)	-0.05(-0.16-0.06)	906.3(831.14-990.49)	839.62(769.5-916.69)	-0.23(-0.26--0.2)	2.61(1.84-3.6)	1.8(1.22-2.62)	-1.36(-1.5--1.21)	49.37(37.36-62.9)	30.68(23.28-40.45)	-1.68(-1.82--1.54)
Spain	Female	31.52(27.77-35.49)	30.41(26.41-34.54)	-0.05(-0.16-0.06)	869.88(787.35-959.63)	802.75(726.52-884.79)	-0.18(-0.22--0.14)	2.41(1.69-3.32)	1.7(1.12-2.47)	-1.41(-1.64--1.18)	46.4(35.25-59.65)	28.97(22.04-38.14)	-1.81(-2--1.63)
Spain	Male	31.49(27.82-35.17)	31.05(27.21-34.96)	-0.04(-0.15-0.08)	948.02(866.34-1037.19)	879.9(802.56-966.1)	-0.28(-0.32--0.25)	2.95(2.07-4.03)	1.96(1.35-2.85)	-1.33(-1.42--1.24)	53.65(39.98-69.19)	32.86(24.73-43.73)	-1.57(-1.69--1.45)
Sri Lanka	Both	25.15(22.27-28.14)	38.62(33.86-42.91)	1.51(1.42-1.61)	2030.69(1820.48-2262.8)	2227.14(2031.9-2453.3)	0.29(0.28-0.31)	9.36(7.54-11.19)	9.57(7.03-12.97)	0.16(-0.28-0.61)	213.19(175.66-255.14)	221.34(166.69-292.99)	0.28(-0.04-0.6)
Sri Lanka	Female	23.6(20.55-26.92)	37.76(32.57-43.01)	1.64(1.56-1.72)	1933.01(1715.14-2178.32)	2112.6(1904.39-2341.5)	0.3(0.29-0.32)	7.04(5.22-8.88)	8.04(5.8-11.02)	0.51(-0.06-1.07)	162.37(126.95-199.63)	183.38(139.36-243.54)	0.52(0.13-0.91)
Sri Lanka	Male	26.68(23.35-30.25)	39.46(34.54-44.7)	1.4(1.29-1.52)	2127.29(1893.25-2384.07)	2360.32(2118.66-2634.08)	0.32(0.29-0.34)	11.68(9.46-14.1)	11.5(8.17-15.76)	0.05(-0.33-0.42)	263.67(213.48-320.63)	265.89(194.69-362.24)	0.21(-0.08-0.5)
Sudan	Both	28.2(24.88-31.86)	52.03(45.91-58.78)	2.12(2.07-2.18)	1306.97(1182.52-1437.71)	1618.84(1482.38-1761.39)	0.7(0.66-0.75)	7.54(5.05-11.23)	8.05(5.09-12.62)	-0.08(-0.32-0.17)	167.34(115.87-236.78)	178.04(120.44-262.15)	-0.07(-0.3-0.17)
Sudan	Female	30.19(26.21-34.76)	49.9(43.54-56.27)	1.92(1.81-2.02)	1382.22(1236.44-1539.95)	1634.19(1480.77-1791.15)	0.64(0.61-0.66)	6.97(4.4-11.14)	7.69(4.78-11.53)	0.11(-0.15-0.37)	163.22(106.61-244.55)	175.48(116.86-250.01)	0.06(-0.17-0.3)
Sudan	Male	26.48(23.22-30.28)	53.78(46.7-61.97)	2.33(2.19-2.47)	1232.15(1102.41-1365.52)	1596.65(1446.65-1754.2)	0.78(0.7-0.86)	8.15(5.39-13.15)	8.38(4.82-15.33)	-0.26(-0.5--0.02)	171.45(116.73-262.37)	179.98(110.11-299.11)	-0.19(-0.43-0.06)
Suriname	Both	20.37(16.85-25.18)	38.28(32.05-47.24)	2.26(2.1-2.42)	1423.53(1290.23-1568.61)	1607(1470.44-1746.22)	0.38(0.36-0.39)	7.68(6.13-9.33)	12.87(9.96-16.17)	1.91(1.59-2.23)	185.13(147.04-223.53)	299.3(231.49-372.41)	1.66(1.38-1.94)
Suriname	Female	17.43(14.37-21.6)	33.47(27.41-41.5)	2.38(2.21-2.55)	1345.54(1203.83-1502.4)	1493.46(1350.37-1636.18)	0.34(0.33-0.35)	6.48(5.13-8.09)	11.04(8.4-13.97)	1.99(1.67-2.32)	160.91(127.25-196.75)	260.19(201.55-328.32)	1.67(1.39-1.95)
Suriname	Male	23.5(19.23-29.41)	43.52(36.15-54.07)	2.18(2.01-2.34)	1505.73(1357.1-1666.31)	1735.27(1576.86-1899.22)	0.43(0.41-0.45)	9.26(7.34-11.42)	15.23(11.43-19.37)	1.81(1.49-2.13)	213.29(166.01-260.88)	345.91(260.28-436.03)	1.67(1.39-1.95)
Sweden	Both	21.94(19.24-24.79)	23.11(20.22-26.39)	0.16(0.15-0.18)	1120.13(1013.18-1238.36)	1147.47(1043.52-1264.23)	0.04(-0.03-0.11)	0.97(0.75-1.23)	1.92(1.43-2.48)	2.46(2.12-2.79)	23.69(18.91-28.99)	33(26.21-40.06)	1.23(1.01-1.44)
Sweden	Female	21.52(18.58-24.56)	21.26(18.57-24.01)	-0.02(-0.04-0)	823.88(743.8-909.48)	805.91(727.62-887.66)	-0.09(-0.14--0.04)	0.74(0.58-0.92)	1.64(1.21-2.08)	3.01(2.69-3.33)	20.12(16.03-24.66)	28.2(22.63-34)	1.33(1.14-1.53)
Sweden	Male	22.72(19.84-26.17)	25.12(21.54-29.16)	0.29(0.26-0.31)	1448.49(1296.08-1613.58)	1497.61(1347.93-1660.52)	0.05(-0.03-0.12)	1.37(1.04-1.79)	2.32(1.71-3.06)	1.69(1.35-2.02)	29.12(22.63-36.5)	38.83(30.5-48.45)	0.97(0.75-1.2)
Switzerland	Both	30.07(26.59-33.39)	33.15(29.41-36.73)	0.27(0.19-0.34)	1017.2(930.8-1106.3)	990.45(906.99-1081.35)	-0.16(-0.19--0.12)	1.4(1.04-1.86)	2.3(1.58-3.16)	2.24(2.03-2.44)	31.22(24.5-38.97)	37.38(29.22-47.43)	0.9(0.77-1.02)
Switzerland	Female	31.21(27.39-35.3)	33.84(29.62-38.03)	0.27(0.19-0.35)	993.89(895.66-1097.44)	960.6(873.27-1057.75)	-0.14(-0.17--0.1)	1.13(0.83-1.5)	2.11(1.42-2.93)	2.6(2.37-2.82)	27.58(21.73-34.61)	34.92(27.29-44.23)	0.96(0.86-1.06)
Switzerland	Male	28.88(25.44-32.43)	32.66(28.47-36.89)	0.3(0.22-0.38)	1043.1(940.18-1146.34)	1021.55(926.73-1124.04)	-0.18(-0.22--0.14)	1.93(1.41-2.6)	2.59(1.83-3.63)	1.65(1.44-1.86)	37.35(28.91-46.87)	40.67(31.28-52.42)	0.7(0.54-0.87)
Syria	Both	36.82(32.42-42.04)	60.5(54.46-67.77)	1.89(1.69-2.1)	1455.19(1334.47-1597.1)	1736.28(1605.78-1896.43)	0.68(0.6-0.76)	10.32(7.6-13.72)	7.97(5.73-10.68)	-1.54(-1.85--1.22)	223.42(166.98-291.97)	171.97(126.92-227.45)	-1.49(-1.79--1.18)
Syria	Female	41.1(35.84-46.92)	59.85(53.06-67.14)	1.54(1.25-1.83)	1560.48(1415.63-1741.37)	1763.66(1614.6-1940.07)	0.56(0.43-0.69)	10.17(7.41-13.75)	8.78(6.24-11.83)	-1.17(-1.52--0.81)	221.2(164.17-290.05)	179.06(129.87-237.81)	-1.29(-1.6--0.97)
Syria	Male	33.03(28.69-37.98)	60.92(54-69.53)	2.22(2.11-2.33)	1356.74(1236.75-1503.76)	1703.33(1562.51-1871.45)	0.79(0.76-0.83)	10.46(7.44-14.63)	7.54(5.25-10.37)	-1.74(-2.02--1.45)	225.23(161.73-303.34)	167.75(120.72-226.02)	-1.62(-1.92--1.32)
Taiwan	Both	36.98(33.58-40.39)	42.27(39.15-45.54)	0.4(0.31-0.5)	1930.07(1739.41-2149.56)	1967.51(1807.96-2144.78)	0.02(-0.01-0.05)	9.89(8.44-11.45)	7.64(5.76-9.93)	-1.04(-1.21--0.87)	208.12(177.61-238.78)	192.9(151.81-239.45)	-0.25(-0.39--0.11)
Taiwan	Female	30.66(26.79-34.84)	38.51(35.14-42.27)	0.75(0.59-0.9)	2033.26(1814.46-2301.63)	2055.65(1866.18-2279.74)	0.02(-0.02-0.06)	10.21(8.6-12.05)	7.38(5.47-9.6)	-1.33(-1.47--1.19)	214.93(184.43-247.36)	181.67(144.16-223.56)	-0.6(-0.71--0.49)
Taiwan	Male	42.52(38.63-46.38)	46.57(42.51-50.78)	0.25(0.2-0.31)	1836.66(1660.34-2034.35)	1877.09(1711.78-2053.27)	0.01(-0.02-0.03)	9.65(8.19-11.22)	7.91(5.96-10.29)	-0.74(-0.96--0.52)	202.84(171.69-233.9)	204.59(159.47-257.21)	0.08(-0.11-0.27)
Tajikistan	Both	9.22(7.75-10.81)	16.14(13.69-18.88)	2.12(1.82-2.42)	1489.91(1306.16-1682.3)	1570.51(1410.09-1743.75)	0.14(0.08-0.2)	0.81(0.62-1.02)	2.73(2.01-3.65)	4.83(4.43-5.24)	27.44(21.77-33.62)	65.82(50.48-82.7)	3.38(3.02-3.74)
Tajikistan	Female	9.04(7.51-10.67)	14.18(11.81-16.69)	1.6(1.3-1.91)	1420.79(1238.01-1620.49)	1467.4(1297.35-1641.09)	0.08(0.02-0.14)	0.69(0.53-0.88)	2.31(1.7-3.04)	4.53(4.14-4.92)	26.79(21.13-33.2)	58.47(44.84-73.54)	2.82(2.47-3.17)
Tajikistan	Male	9.59(8.04-11.3)	18.37(15.49-21.54)	2.55(2.24-2.86)	1569.18(1373.92-1771.24)	1680.69(1503.97-1878.26)	0.19(0.12-0.25)	1(0.76-1.32)	3.3(2.38-4.48)	4.9(4.46-5.34)	29.34(23.11-36.12)	74.82(55.91-95.79)	3.79(3.4-4.18)
Tanzania	Both	13.08(11.61-14.56)	16.7(14.87-18.56)	0.83(0.78-0.88)	1004.94(898.18-1118.62)	1087.94(977.73-1198.65)	0.24(0.22-0.26)	5.96(4.31-8.09)	6.43(4.9-8.24)	0.28(0.2-0.35)	120.74(88.23-160.93)	126.84(99.46-157.47)	0.17(0.1-0.23)
Tanzania	Female	12.07(10.55-13.75)	15.95(13.89-18.03)	0.93(0.87-0.99)	982.11(870.51-1097.69)	1061.17(940.05-1175.08)	0.26(0.24-0.29)	5.26(3.71-7.45)	6.26(4.61-8.23)	0.74(0.6-0.88)	107.91(78.75-151.19)	123.33(95.31-156.24)	0.58(0.44-0.72)
Tanzania	Male	14.06(12.41-15.8)	17.42(15.4-19.59)	0.74(0.7-0.79)	1030.07(913.37-1157.26)	1117.97(1001.63-1247.16)	0.22(0.2-0.24)	6.71(4.63-9.57)	6.55(4.83-8.74)	-0.17(-0.24--0.1)	134.19(94.19-184.86)	130.15(98.09-169.7)	-0.22(-0.29--0.15)

Thailand	Both	26.81(23.62-30.21)	41.01(36.8-45.31)	1.4(1.28-1.53)	2198.72(1973.67-2446.96)	2297.92(2094.54-2532.88)	0.06(0.03-0.09)	10.8(8.89-12.85)	10.5(7.62-13.92)	0.07(-0.06-0.21)	247.68(200.65-294.93)	237.01(176.44-311.43)	0.01(-0.13-0.15)
Thailand	Female	26.35(23.07-29.95)	38.84(34.65-43.73)	1.27(1.1-1.43)	2097.38(1873.7-2366.18)	2149.75(1938.71-2388.55)	0.01(-0.01-0.04)	8.74(6.89-10.71)	10.46(7.53-14)	1.07(0.75-1.39)	202.84(161.4-246.39)	230.85(172.4-300.06)	0.9(0.58-1.22)
Thailand	Male	27.33(23.63-31.55)	43.45(37.69-49.12)	1.54(1.44-1.65)	2313.11(2050.15-2583.49)	2466.3(2224.57-2734.03)	0.11(0.07-0.14)	13.65(10.66-17.17)	10.45(7.46-14.2)	-1.07(-1.35--0.78)	300.87(235.79-374.89)	242.26(177.94-326.43)	-0.87(-1.13--0.62)
Timor-Leste	Both	18.13(15.98-20.48)	26.38(23.2-29.52)	1.38(1.29-1.48)	1893.83(1695.95-2130.35)	2001.06(1809.85-2222)	0.15(0.12-0.19)	11.67(8.68-16.65)	11.99(8.92-15.52)	0.09(-0.11-0.28)	277.43(201.41-389.03)	274.4(199.2-358.71)	-0.07(-0.3-0.16)
Timor-Leste	Female	19.26(16.92-21.83)	27.95(24.38-31.99)	1.38(1.3-1.46)	1838.82(1629.1-2091.53)	1925.16(1730.15-2147.42)	0.16(0.12-0.2)	12.82(9.45-17.26)	11.57(8.66-15.36)	-0.49(-0.69--0.29)	314.68(230.83-419.88)	273.17(202.17-365.95)	-0.65(-0.88--0.42)
Timor-Leste	Male	17.05(14.76-19.46)	24.81(21.45-28.23)	1.36(1.26-1.47)	1946(1722.7-2187.83)	2075.85(1861.24-2330.24)	0.16(0.12-0.19)	10.65(7.07-18.26)	12.42(8.61-17.9)	0.65(0.44-0.86)	242.64(159.63-406.03)	275.78(184.63-398.03)	0.55(0.31-0.79)
Tobago	Both	23.46(20.55-26.47)	37.07(32.76-41.55)	1.58(1.48-1.68)	1423.4(1284.9-1580.92)	1616.17(1465.82-1765.15)	0.41(0.39-0.43)	5.82(4.66-7.07)	9.17(6.37-12.53)	2.02(1.77-2.27)	139.04(111.21-167.2)	225.11(162.65-303.6)	1.96(1.7-2.21)
Tobago	Female	20.14(17.42-23.03)	31.88(27.71-36.47)	1.58(1.51-1.66)	1339.85(1193.52-1504.75)	1486.72(1345.21-1635.23)	0.35(0.34-0.37)	4.05(3.22-4.93)	7.21(5.01-9.98)	2.46(2.11-2.8)	106.68(84.8-128.04)	186.22(136.15-250.72)	2.21(1.9-2.52)
Tobago	Male	27.05(23.47-30.6)	42.47(36.74-47.95)	1.56(1.45-1.68)	1515.07(1360.87-1691.54)	1753.49(1587.47-1937.28)	0.46(0.42-0.49)	8.26(6.63-10.06)	11.61(8.03-15.99)	1.6(1.4-1.81)	178.16(141.64-213.38)	268.9(192.09-362.42)	1.72(1.5-1.95)
Togo	Both	17.16(15.16-19.19)	22.02(19.55-24.57)	0.91(0.81-1.01)	1052.96(949.83-1160.01)	1140.8(1040.45-1251.04)	0.21(0.18-0.25)	8.7(6.33-11.78)	8.04(5.88-10.92)	-0.28(-0.34--0.23)	177.16(128.04-233.88)	166.77(122.01-223.72)	-0.2(-0.25--0.14)
Togo	Female	16.52(14.26-18.85)	20.97(18.31-23.69)	0.89(0.79-1)	1044.79(936.54-1167.32)	1115.4(1004.67-1237.69)	0.18(0.15-0.22)	7.89(5.39-12.09)	6.73(4.74-9.54)	-0.55(-0.68--0.43)	164.73(113.56-239.3)	140.3(99.35-198.77)	-0.55(-0.7--0.4)
Togo	Male	17.79(15.44-20.14)	23.92(21.09-26.89)	1.09(0.99-1.18)	1063.86(947.26-1184.65)	1173.21(1052.53-1303.04)	0.25(0.21-0.29)	9.61(6.85-13.02)	10.15(7.29-13.9)	0.22(0.07-0.37)	190.52(135.24-256.07)	205.56(146.73-277.74)	0.31(0.15-0.47)
Tonga	Both	23.53(20.65-26.76)	32.44(28.62-36.41)	0.97(0.9-1.04)	2066.19(1849.76-2311.02)	2164.08(1958.34-2387.61)	0.08(0.06-0.1)	10.35(8.06-14.1)	14.96(11.42-20.43)	1.24(0.85-1.64)	251.24(194.65-337.9)	355.13(269.87-477.49)	1.15(0.81-1.49)
Tonga	Female	21.23(18.51-24.34)	27.71(24.21-31.37)	0.78(0.7-0.87)	2129.67(1880.51-2402.12)	2163.81(1938.66-2400.3)	0(-0.02-0.02)	10.32(7.46-15.04)	12.26(8.88-17.73)	0.25(-0.19-0.69)	265.88(195.32-380.32)	306.99(223.84-425.99)	0.13(-0.25-0.51)
Tonga	Male	26.07(22.59-29.81)	38.25(33.61-43.41)	1.2(1.14-1.26)	2000.01(1782.45-2244.91)	2172.58(1955.99-2418.39)	0.18(0.15-0.21)	10.68(8.36-13.75)	18.78(13.83-25.35)	2.25(1.85-2.66)	238.08(183.81-312.95)	415.08(304.79-566.8)	2.24(1.88-2.59)
Trinidad	Both	23.46(20.55-26.47)	37.07(32.76-41.55)	1.58(1.48-1.68)	1423.4(1284.9-1580.92)	1616.17(1465.82-1765.15)	0.41(0.39-0.43)	5.82(4.66-7.07)	9.17(6.37-12.53)	2.02(1.77-2.27)	139.04(111.21-167.2)	225.11(162.65-303.6)	1.96(1.7-2.21)
Trinidad	Female	20.14(17.42-23.03)	31.88(27.71-36.47)	1.58(1.51-1.66)	1339.85(1193.52-1504.75)	1486.72(1345.21-1635.23)	0.35(0.34-0.37)	4.05(3.22-4.93)	7.21(5.01-9.98)	2.46(2.11-2.8)	106.68(84.8-128.04)	186.22(136.15-250.72)	2.21(1.9-2.52)
Trinidad	Male	27.05(23.47-30.6)	42.47(36.74-47.95)	1.56(1.45-1.68)	1515.07(1360.87-1691.54)	1753.49(1587.47-1937.28)	0.46(0.42-0.49)	8.26(6.63-10.06)	11.61(8.03-15.99)	1.6(1.4-1.81)	178.16(141.64-213.38)	268.9(192.09-362.42)	1.72(1.5-1.95)
Tunisia	Both	35.59(31.37-40.36)	63.23(56.12-70.76)	2.08(1.92-2.23)	1408.12(1277.14-1538.34)	1762.11(1605.48-1918.45)	0.8(0.76-0.84)	6.39(4.69-8.51)	6.42(4.34-9.16)	0.08(-0.03-0.19)	133.79(101.8-172.1)	140.75(101.83-189.11)	0.23(0.14-0.32)
Tunisia	Female	37.88(32.81-43.5)	60.35(52.89-68.05)	1.79(1.54-2.03)	1486.65(1335.55-1642.71)	1767.01(1604.36-1931.42)	0.68(0.6-0.76)	6.14(4.45-8.71)	6.23(3.96-9.09)	0.15(0.02-0.28)	132.96(100.73-179.46)	136.87(96.19-185.96)	0.22(0.13-0.31)
Tunisia	Male	33.51(29.26-38.24)	66.37(58.76-74.92)	2.38(2.32-2.44)	1330.02(1194.17-1468.65)	1760.23(1594.75-1920.99)	0.92(0.89-0.95)	6.72(4.74-9.2)	6.67(4.26-10.05)	0.04(-0.09-0.16)	135.11(98.88-177.52)	145.35(102.34-205.3)	0.25(0.16-0.35)
Turkey	Both	30.82(27.76-34.37)	59.83(53.44-66.76)	2.58(2.37-2.79)	1330.91(1221.62-1460.57)	1670.52(1539.13-1817.37)	0.95(0.87-1.04)	11.36(8.41-16.8)	7.56(5.72-9.77)	-0.77(-1.07--0.47)	234.68(176.43-324.6)	159.32(124.36-201.07)	-0.74(-0.97--0.51)
Turkey	Female	30.03(26.79-33.92)	55.43(49.23-62.64)	2.49(2.17-2.81)	1369.36(1242.18-1521.83)	1644.73(1501.9-1804.6)	0.88(0.75-1)	9.71(7.08-15.53)	7.19(5.27-9.47)	-0.45(-0.91-0.02)	204.97(155.15-306.66)	148.8(115.65-188.68)	-0.57(-0.85--0.3)
Turkey	Male	31.74(28.47-35.36)	64.86(57.04-73.35)	2.67(2.56-2.79)	1297.38(1179.2-1426.81)	1710.45(1560.44-1872.95)	1.04(0.99-1.09)	13.4(9.23-20.78)	8(5.93-10.51)	-1.09(-1.33--0.85)	268.98(189.76-389.09)	171.1(130.72-218.51)	-0.9(-1.11--0.69)
Turkmenistan	Both	12.91(11.23-14.82)	19.51(17.06-22.08)	1.54(1.35-1.73)	1588.93(1410.09-1776.17)	1717.58(1539.23-1893.79)	0.24(0.2-0.28)	3.62(2.76-4.42)	5.19(3.74-6.85)	0.64(0.26-1.01)	106.16(81.64-130.33)	148.58(109.24-194.47)	0.65(0.33-0.97)
Turkmenistan	Female	12.22(10.53-14.2)	17.02(14.66-19.62)	1.18(0.95-1.4)	1520.14(1341.77-1697.38)	1606.82(1437.47-1784.48)	0.17(0.13-0.21)	3.25(2.47-4)	4.04(2.92-5.41)	0.11(-0.22-0.44)	97.75(75.14-119.55)	116.11(85.22-151.8)	0.05(-0.22-0.32)
Turkmenistan	Male	14.02(12.12-16.27)	22.77(19.84-26.37)	1.88(1.71-2.05)	1676.97(1483.56-1889.03)	1846.07(1647.18-2071.3)	0.31(0.27-0.34)	4.23(3.17-5.26)	6.73(4.79-8.82)	1.03(0.6-1.46)	118.5(91-145.86)	187.83(136.38-246.09)	1.11(0.72-1.5)
Uganda	Both	11.38(10.16-12.68)	14.3(12.73-16.06)	0.85(0.76-0.94)	1053.73(938.26-1179.01)	1066.79(961.35-1179.09)	0(-0.02-0.02)	7.17(5.11-9.91)	7.42(5.45-10.17)	-0.13(-0.25-0)	141.11(100.58-192.57)	144.21(105.48-193.75)	-0.17(-0.32--0.03)
Uganda	Female	9.31(8.19-10.53)	12.18(10.67-13.72)	0.96(0.86-1.07)	1018.65(902.95-1160.46)	1020.02(912.83-1145.2)	-0.02(-0.04-0.01)	5.17(3.49-8.1)	5.95(4.21-8.51)	0.33(0.2-0.46)	100.3(68.51-151.88)	113.23(82.67-160.09)	0.27(0.13-0.42)
Uganda	Male	13.53(11.96-15.16)	17.01(15.03-19.18)	0.86(0.79-0.94)	1091.15(963.96-1224.61)	1124.13(1000.73-1253.79)	0.03(0.01-0.06)	9.39(6.56-12.89)	9.48(6.46-13.17)	-0.27(-0.42--0.11)	184.88(130.58-248.8)	185.18(130.46-256.96)	-0.31(-0.48--0.13)
UK	Both	26.91(23.85-30.09)	29.07(25.82-32.48)	0.02(-0.12-0.16)	877.43(811.98-944.68)	880.45(816.39-946.1)	-0.14(-0.19--0.09)	0.96(0.7-1.31)	0.9(0.66-1.23)	0.27(-0.25-0.79)	24.41(18.8-30.81)	22.93(17.68-29.29)	0.02(-0.19-0.23)
UK	Female	25.99(23.18-29.07)	26.59(23.55-29.63)	-0.09(-0.21-0.03)	839.19(775.86-903.22)	830.18(768.56-890.56)	-0.11(-0.15--0.08)	0.88(0.65-1.2)	0.88(0.63-1.19)	0.56(-0.09-1.22)	23.07(17.91-29.14)	22.34(17.35-28.48)	0.17(-0.1-0.44)
UK	Male	28.4(25.16-31.77)	31.98(28.53-35.81)	0.07(-0.1-0.23)	927.74(856.03-997.91)	937.92(868.26-1011.57)	-0.19(-0.27--0.12)	1.14(0.82-1.56)	0.94(0.67-1.31)	-0.35(-0.7-0)	26.84(20.52-33.83)	23.7(18.15-30.43)	-0.28(-0.42--0.14)
Ukraine	Both	11.42(9.82-13.32)	13.88(12.1-15.76)	0.69(0.46-0.91)	1778.54(1572.59-2019.94)	1719.68(1540.97-1919.73)	-0.19(-0.22--0.15)	0.32(0.23-0.44)	0.43(0.3-0.59)	1.15(0.98-1.33)	10.99(8.31-14.1)	15.26(11.48-19.7)	1.37(1.18-1.57)
Ukraine	Female	11.62(9.89-13.7)	12.32(10.59-14.18)	0.04(-0.25-0.33)	1715.21(1500.51-1961.69)	1601.1(1414.82-1808.59)	-0.29(-0.33--0.25)	0.27(0.19-0.37)	0.36(0.24-0.51)	1.15(0.99-1.3)	10.11(7.5-13.15)	12.72(9.31-16.57)	0.98(0.78-1.18)
Ukraine	Male	11.47(9.77-13.33)	16.74(14.46-19.19)	1.59(1.44-1.74)	1877.88(1644.09-2145.92)	1886.98(1675.33-2120.73)	-0.06(-0.09--0.03)	0.46(0.33-0.63)	0.57(0.39-0.8)	0.81(0.63-0.99)	13.08(10.04-17.02)	19.41(14.39-25.21)	1.64(1.44-1.83)
United Arab Emirates	Both	49.02(43.27-54.9)	75.43(67.46-83.38)	1.43(1.29-1.57)	1632.22(1483.82-1784.15)	1966.03(1805.68-2145.45)	0.58(0.5-0.66)	20.41(12.32-27.03)	15.89(9.34-25.51)	-0.83(-1.47--0.18)	412.04(272.38-528.87)	337.67(215.62-525.05)	-0.65(-1.17--0.13)
United Arab Emirates	Female	52.74(46.19-59.83)	72.61(64.93-80.78)	1.11(0.86-1.36)	1778.11(1606.52-1964.12)	2012.57(1849.4-2196.82)	0.44(0.31-0.57)	20.87(11.58-29.88)	17.12(8.55-24.47)	-0.14(-1.3-1.04)	427.74(259.39-603.83)	360.14(204.11-505.45)	-0.12(-1.05-0.83)
United Arab Emirates	Male	46.95(41.08-53.11)	77.29(68.59-86.47)	1.63(1.51-1.74)	1567.74(1429.3-1721.43)	1962.44(1782.51-2156.4)	0.68(0.62-0.74)	20.24(11.88-28.06)	15.38(8.87-28.04)	-1.25(-1.64--0.86)	405.17(253.09-559.78)	329.32(207.16-570.54)	-0.99(-1.31--0.67)
Uruguay	Both	22.9(20.49-25.62)	30.07(27.28-33.49)	0.92(0.87-0.96)	783.18(701.12-879.41)	1032.15(938.71-1127.49)	0.96(0.89-1.03)	3.27(2.88-3.65)	4.6(3.75-5.64)	1.69(1.42-1.98)	68.96(61.29-76.9)	90.09(76.15-105.04)	1.35(1.11-1.59)

Uruguay	Female	20.46(17.93-23.34)	26.3(23.28-29.78)	0.81(0.75-0.88)	749(657.8-849.55)	970.49(871.4-1074.04)	0.87(0.82-0.92)	2.38(2.09-2.68)	3.69(2.95-4.52)	2.07(1.78-2.37)	51.29(45.25-58.03)	71.63(60.03-83.91)	1.63(1.38-1.88)
Uruguay	Male	26.18(23.1-29.77)	35.19(31.45-39.51)	1.04(1.01-1.07)	832.6(743.08-929.6)	1117.8(1022.07-1222.39)	1.06(0.97-1.14)	4.59(4.02-5.19)	5.98(4.78-7.39)	1.41(1.13-1.68)	92.68(81.68-104.11)	115.5(96.58-136.41)	1.16(0.91-1.4)
USA	Both	39.81(35.66-44.36)	42.95(38.79-47.47)	0.08(-0.01-0.18)	1282.62(1181.8-1382.62)	1367.81(1265.31-1468.93)	0.16(0.1-0.21)	1.88(1.4-2.46)	5.45(4.24-6.67)	3.86(3.4-4.32)	55.23(42.86-68.25)	122.51(98.96-145.8)	2.91(2.53-3.29)
USA	Female	37.56(33.84-41.63)	40.52(36.78-44.64)	0.08(-0.01-0.18)	1302.73(1199.56-1401.86)	1404.24(1294.12-1511.65)	0.17(0.13-0.22)	1.93(1.43-2.5)	5(3.89-6.22)	3.41(2.92-3.91)	55.42(43.52-67.44)	113.42(92.05-135.88)	2.54(2.12-2.96)
USA	Male	43.15(38.44-48.57)	45.99(41.43-50.94)	0.03(-0.08-0.14)	1276.38(1178.3-1379.34)	1336.89(1236.52-1439.68)	0.12(0.04-0.19)	1.81(1.3-2.45)	6.02(4.61-7.59)	4.44(4.01-4.87)	54.87(41.85-69.15)	133.22(104.83-160.42)	3.32(2.98-3.67)
Uzbekistan	Both	16.31(13.98-18.93)	24.81(21.24-28.74)	1.48(1.32-1.63)	1592.91(1421.6-1779.57)	1763.2(1585.22-1947.81)	0.33(0.29-0.37)	4.12(2.86-6.37)	6.13(4.59-7.71)	1.1(0.2-2)	119.05(86.31-169.65)	157.38(121.21-196.84)	0.57(-0.21-1.35)
Uzbekistan	Female	16.59(14.22-19.3)	22.18(18.82-25.96)	0.93(0.78-1.08)	1534.94(1353.42-1734.08)	1657.01(1486.3-1839.47)	0.26(0.21-0.3)	3.84(2.5-7.05)	5.33(4.06-6.69)	0.89(0.07-1.71)	108.89(76.61-177.09)	135.99(104.96-169.36)	0.38(-0.33-1.1)
Uzbekistan	Male	15.98(13.51-18.78)	28.36(24.25-33.26)	2.13(1.98-2.28)	1660.18(1475.77-1864.47)	1891.92(1702.82-2105.9)	0.43(0.38-0.47)	4.41(3.14-5.83)	7.18(5.33-9.18)	1.43(0.45-2.42)	130.08(94.95-172.05)	184.15(140.35-231.95)	0.82(-0.02-1.67)
Vanuatu	Both	17.81(15.56-20.37)	25.8(22.57-29.43)	1.08(0.97-1.19)	1934.64(1720.5-2192.74)	2020.03(1818.57-2258.55)	0.07(0.03-0.1)	8.8(6.05-12.6)	14.8(10.72-19.88)	1.98(1.85-2.12)	216.56(150.23-304.65)	361.28(255.66-495.04)	1.87(1.74-1.99)
Vanuatu	Female	15.65(13.54-18.39)	22.29(19.3-25.71)	1.02(0.92-1.12)	1998.71(1764.11-2303.23)	2044.49(1823.23-2314.07)	0.01(-0.01-0.04)	9.3(5.71-14.45)	14.59(10-21.41)	1.51(1.37-1.66)	237.66(152.49-361.03)	369.53(251.5-539.91)	1.41(1.25-1.58)
Vanuatu	Male	19.4(16.83-22.05)	28.93(25.17-33.17)	1.18(1.07-1.3)	1875.19(1661.42-2128.64)	1997.23(1785.3-2252.66)	0.11(0.08-0.15)	8.42(5.74-12.64)	15.02(10.96-20.92)	2.41(2.22-2.59)	199.56(137.89-291.07)	354.12(258.92-484.07)	2.3(2.14-2.46)
Venezuela	Both	32.7(28.69-37.43)	48.81(43.67-54.23)	1.35(1.24-1.46)	1885.51(1700.87-2087.17)	2064.83(1891.31-2252.71)	0.27(0.24-0.29)	4.73(3.77-5.72)	11.16(7.89-15.17)	2.58(2.06-3.1)	125.88(102.03-149.2)	267.84(194.88-354.99)	2.19(1.7-2.68)
Venezuela	Female	29.89(25.97-34.43)	43.33(38.11-48.61)	1.26(1.15-1.37)	1771.18(1575.93-1974.78)	1875.83(1688.14-2064.05)	0.17(0.16-0.19)	4.03(3.21-4.86)	9.26(6.5-12.54)	2.52(2.04-3.01)	107.9(87.71-128.53)	220.84(161.59-292.82)	2.05(1.59-2.52)
Venezuela	Male	35.7(31.05-41.32)	54.65(47.38-62.35)	1.43(1.32-1.55)	2010.89(1817.58-2227.82)	2275.13(2065.89-2491.59)	0.36(0.32-0.39)	5.55(4.44-6.75)	13.46(9.51-18.48)	2.62(2.01-3.24)	145.97(118.04-174.11)	321.09(233.57-430.67)	2.3(1.75-2.85)
Vietnam	Both	16.64(14.7-18.76)	26.28(23.16-29.55)	1.71(1.64-1.78)	1851.83(1637.95-2079.98)	1947.26(1741.1-2150.09)	0.15(0.12-0.19)	10.77(8.34-13.76)	10.09(7.58-12.82)	-0.33(-0.83-0.17)	229.91(174.92-296.81)	208.42(158.71-269.88)	-0.37(-0.83-0.09)
Vietnam	Female	17.65(15.35-20.26)	27.45(23.91-31.28)	1.68(1.59-1.76)	1811.54(1583.12-2048.52)	1878.45(1665.54-2099.2)	0.13(0.1-0.16)	11.76(8.78-15.31)	10.17(7.33-13.32)	-0.74(-1.37--0.11)	256.48(191.83-335.74)	211.58(158.86-274.68)	-0.81(-1.36--0.26)
Vietnam	Male	15.29(13.38-17.39)	24.65(21.28-28.16)	1.75(1.68-1.81)	1905.41(1676.79-2149.55)	2024.37(1788.19-2260.23)	0.16(0.11-0.2)	9.09(6.53-13.22)	9.71(7.03-12.69)	0.34(0.07-0.61)	193.19(140.22-273.14)	202.44(146.65-264.01)	0.29(-0.01-0.6)
Virgin Islands	Both	41.99(37.14-47.74)	44.75(40.25-50)	0.04(-0.06-0.13)	1343.1(1226.13-1469.22)	1465.86(1343.62-1586.5)	0.22(0.16-0.29)	2.27(1.64-3)	6.22(4.5-8.04)	3.4(2.84-3.96)	63.17(48.2-78.08)	134.85(105.12-167.66)	2.59(2.13-3.05)
Virgin Islands	Female	39.66(34.96-45.61)	42.06(37.09-47.74)	0.02(-0.07-0.12)	1349.74(1221.31-1483.85)	1493.63(1358.5-1641.59)	0.24(0.19-0.29)	2.34(1.7-3.09)	5.72(4.09-7.69)	2.94(2.37-3.52)	63.91(48.7-79.68)	124.7(94.74-158.79)	2.17(1.69-2.66)
Virgin Islands	Male	45.6(39.32-52.04)	48.11(42.56-54.23)	-0.02(-0.12-0.08)	1354.84(1225.19-1483.26)	1444.99(1309.67-1573.19)	0.17(0.08-0.26)	2.16(1.52-2.92)	6.87(4.91-9.46)	3.99(3.43-4.56)	62.08(46.89-78.67)	146.95(108.35-193.31)	3.05(2.6-3.5)
Yemen	Both	27.27(23.95-31.09)	46.96(41.11-54.21)	2.05(2.01-2.09)	1242.7(1127.79-1364.45)	1483.49(1351.26-1623.29)	0.62(0.58-0.67)	7.2(4.86-10.68)	6.88(4.82-9.42)	-0.2(-0.28--0.11)	163.57(113.17-239.37)	157.22(113.51-211.36)	-0.18(-0.28--0.09)
Yemen	Female	28.32(24.64-32.89)	45.4(38.46-54.09)	1.91(1.8-2.01)	1304.8(1173.46-1452.95)	1505.39(1356.45-1671.53)	0.56(0.54-0.59)	6.35(4.08-10.73)	6.16(4.2-8.99)	-0.13(-0.29-0.04)	150.22(102.11-240.45)	147.39(106.19-206.18)	-0.08(-0.23-0.08)
Yemen	Male	26.29(22.74-30.21)	48.61(41.78-56.2)	2.2(2.1-2.3)	1182.89(1073.13-1301.47)	1462.47(1327.7-1606.67)	0.69(0.6-0.77)	8.69(5.7-13.39)	7.69(5.15-11.09)	-0.49(-0.53--0.44)	183.22(124.47-268.2)	167.83(116.68-231.35)	-0.38(-0.45--0.31)
Zambia	Both	13.98(12.55-15.47)	18.12(16.12-20.13)	0.93(0.83-1.04)	1043.73(938.55-1151.47)	1106.92(1001.81-1211.45)	0.15(0.12-0.18)	8.68(6.22-11.48)	8.73(6.41-11.79)	-0.24(-0.45--0.02)	177.18(128.79-227.48)	178.05(131.64-238.22)	-0.27(-0.5--0.04)
Zambia	Female	12.52(11.05-14.13)	16.55(14.45-18.65)	1.01(0.9-1.12)	1024.96(912.01-1151.33)	1068.91(962.23-1180.59)	0.11(0.08-0.14)	8.09(5.76-10.66)	7.76(5.54-10.68)	-0.46(-0.59--0.32)	170.16(123.09-217.79)	158.44(115.26-216.95)	-0.59(-0.74--0.44)
Zambia	Male	15.13(13.47-16.9)	19.92(17.63-22.3)	1.01(0.91-1.12)	1061.2(941.94-1184.04)	1147.67(1028.21-1274.39)	0.2(0.16-0.23)	9.09(6.21-12.8)	9.86(6.97-13.55)	0.11(-0.21-0.43)	181.33(124.21-247.58)	200.62(143.45-268.42)	0.13(-0.21-0.48)
Zimbabwe	Both	21.15(18.63-24.11)	24.73(21.96-28.15)	0.3(-0.01-0.61)	1197.5(1070.2-1337.28)	1286.99(1155.37-1415.35)	0.11(0.03-0.2)	5.13(3.41-8.36)	8.44(5.57-13.72)	1.95(1.54-2.36)	109.71(74.24-168.41)	179.97(119.98-279.91)	1.95(1.57-2.32)
Zimbabwe	Female	17.15(14.86-19.74)	21(18.32-24.09)	0.45(0.14-0.77)	1144.27(1011.94-1286.91)	1228.39(1094.75-1363.41)	0.15(0.07-0.23)	3.87(2.27-7.79)	6.55(3.76-13.33)	2.69(2.21-3.18)	82.32(51.04-153.17)	137.77(82.92-268.34)	2.75(2.27-3.23)
Zimbabwe	Male	25.5(22.36-29.38)	30.1(26.63-34.28)	0.36(0.07-0.65)	1259.15(1118.7-1410.61)	1368.47(1229.07-1518.36)	0.12(0.03-0.21)	6.81(4.55-10.32)	11.41(7.73-16.49)	1.67(1.13-2.21)	140.97(95.17-215.39)	241(162.58-344.68)	1.73(1.2-2.27)

Abbreviation: ASIR, age-standardized incidence rate; ASPR, age-standardized prevalence rate; ASDR, age-standardized death rate; CI, confidence interval; DALY, disability adjusted life-year; EAPC, estimated annual percentage change; UI, uncertainty interval

Table S7. The YLDs and their ASRs of impairment caused by diabetes mellitus related chronic kidney disease.

Location	Impairment	Sex	Chronic kidney disease caused by diabetes mellitus type 1					Chronic kidney disease caused by diabetes mellitus type 2				
			YLDs (95% UI)		ASR (95% UI)		EAPC (95% CI)	YLDs (No. ×1000) (95% UI)		ASR (95% UI)		EAPC (95% CI)
			1990	2019	1990	2019		1990	2019	1990	2019	
Global	Anemia	Both	11470.32(16760.35-7439.05)	17964.13(26338.69-11600.09)	0.21(0.13-0.3)	0.24(0.15-0.35)	0.88(0.74-1.02)	99136.83(147999.72-64178.14)	201132.29(303662.62-127973.98)	2.55(1.65-3.78)	2.48(1.58-3.74)	-0.06(-0.1--0.03)
		Female	6225.64(9147.69-4005.86)	10452.23(15353.92-6791.17)	0.23(0.15-0.33)	0.27(0.18-0.4)	1.06(0.9-1.21)	59968.08(88371.32-38797.28)	121482.64(181340.85-78554.82)	2.79(1.79-4.08)	2.79(1.8-4.16)	0.07(0.04-0.1)
		Male	5244.68(7797.26-3358.05)	7511.9(11118.68-4829.12)	0.18(0.12-0.27)	0.2(0.13-0.3)	0.65(0.53-0.78)	39168.74(59127.96-25005.77)	79649.64(121591.93-49780.79)	2.36(1.52-3.53)	2.19(1.37-3.32)	-0.28(-0.33--0.23)
	Mild anemia	Both	665.26(1475.83-232.46)	1368.46(3004.87-473.39)	0.01(0-0.03)	0.02(0.01-0.04)	1.62(1.46-1.79)	7093.14(15545.26-2468.99)	18784.19(41002.28-6546.09)	0.18(0.06-0.4)	0.23(0.08-0.51)	0.94(0.9-0.98)
		Female	332.2(744.15-116.61)	698.37(1530.14-243.47)	0.01(0-0.03)	0.02(0.01-0.04)	1.74(1.6-1.88)	2942.69(6474-1023.42)	7464.77(16124-2638.76)	0.14(0.05-0.3)	0.17(0.06-0.37)	0.98(0.92-1.04)
		Male	333.05(738.35-114.67)	670.09(1467.08-230.22)	0.01(0-0.03)	0.02(0.01-0.04)	1.51(1.32-1.71)	4150.45(9072.33-1440.47)	11319.41(24877.23-3942.62)	0.25(0.09-0.55)	0.31(0.11-0.68)	0.82(0.79-0.85)
	Moderate anemia	Both	8375.76(12354.11-5334.29)	13565.78(19864.65-8731.55)	0.15(0.09-0.22)	0.18(0.11-0.26)	1(0.87-1.14)	68400.78(101709.22-43411.44)	143178.08(213495.44-90374.5)	1.76(1.12-2.6)	1.77(1.12-2.63)	0.07(0.04-0.1)
		Female	4620.82(6833.6-2937.4)	8094.35(12036.15-5178.5)	0.17(0.11-0.25)	0.21(0.14-0.31)	1.2(1.05-1.35)	43092.21(64193.26-27559.37)	90896.31(134728.03-57848.62)	2(1.28-2.97)	2.09(1.33-3.1)	0.22(0.19-0.25)
		Male	3754.94(5612.51-2346.86)	5471.43(8130.4-3473.94)	0.13(0.08-0.19)	0.15(0.09-0.22)	0.73(0.61-0.85)	25308.56(37832.89-15992.87)	52281.77(77721.3-32950.49)	1.52(0.97-2.24)	1.44(0.91-2.14)	-0.18(-0.22--0.13)
	Severe anemia	Both	2429.31(3533.42-1588.46)	3029.9(4391.12-1979.24)	0.04(0.03-0.06)	0.04(0.03-0.06)	0.17(0.03-0.31)	23642.91(34225.29-15885.39)	39170.02(56017.65-26285.94)	0.61(0.41-0.88)	0.48(0.32-0.69)	-0.83(-0.91--0.74)
		Female	1272.62(1838.81-827.04)	1659.51(2423.14-1077.94)	0.05(0.03-0.07)	0.04(0.03-0.06)	0.24(0.07-0.41)	13933.18(20028.24-9529.46)	23121.56(32961.13-15483.35)	0.65(0.44-0.93)	0.53(0.35-0.75)	-0.66(-0.72--0.59)
		Male	1156.69(1703.81-756.01)	1370.38(2002.98-891.99)	0.04(0.03-0.06)	0.04(0.02-0.05)	0.08(-0.05-0.21)	9709.73(14127.4-6525.67)	16048.47(23028.89-10780.36)	0.59(0.4-0.86)	0.44(0.3-0.63)	-1.11(-1.22--1)
High SDI	Anemia	Both	864.65(1371.99-521.03)	1043.21(1637.95-628.67)	0.1(0.06-0.16)	0.1(0.06-0.15)	0.08(-0.04-0.2)	14576.15(22348.75-8945.28)	25194.39(39747.3-15400.47)	1.37(0.84-2.1)	1.25(0.76-1.97)	-0.21(-0.31--0.11)
		Female	606.14(955.94-365.62)	720.17(1134.53-448.17)	0.14(0.08-0.22)	0.13(0.08-0.21)	0.16(0.03-0.28)	9597.2(14504.24-5923.12)	15011.46(23499.62-9220.22)	1.53(0.94-2.33)	1.38(0.83-2.15)	-0.23(-0.34--0.12)
		Male	258.52(413.22-149.81)	323.04(534.79-179)	0.07(0.04-0.11)	0.06(0.03-0.1)	0.01(-0.25-0.27)	4978.95(7965.16-2994.74)	10182.93(16499.99-5880.41)	1.21(0.73-1.93)	1.14(0.66-1.86)	-0.15(-0.24--0.06)
	Mild anemia	Both	144.34(317.39-49.89)	209.37(453.27-72.09)	0.02(0.01-0.04)	0.02(0.01-0.04)	0.65(0.53-0.78)	2302.33(5026.93-808.25)	4678.81(10367.75-1634.34)	0.22(0.08-0.47)	0.24(0.08-0.53)	0.36(0.32-0.41)
		Female	81.03(181.52-28.73)	108.1(244.31-37.92)	0.02(0.01-0.04)	0.02(0.01-0.05)	0.57(0.41-0.73)	1005.83(2274-353.19)	1818.21(3982.89-640.37)	0.16(0.06-0.37)	0.17(0.06-0.38)	0.31(0.25-0.36)
		Male	63.31(141.28-21.95)	101.26(222.33-35.43)	0.02(0.01-0.03)	0.02(0.01-0.04)	0.78(0.56-0.99)	1296.5(2891.51-462.57)	2860.6(6369.34-993.49)	0.3(0.11-0.68)	0.32(0.11-0.72)	0.18(0.14-0.21)
	Moderate anemia	Both	654.93(989.91-409.6)	763.66(1151.87-484.63)	0.08(0.05-0.12)	0.07(0.04-0.11)	-0.01(-0.13-0.1)	10855.17(16107.38-6813.62)	18425.54(27792.24-11708.98)	1.02(0.64-1.51)	0.91(0.58-1.37)	-0.28(-0.39--0.17)
		Female	479.37(731.51-300.94)	563.32(853.2-356.92)	0.11(0.07-0.17)	0.1(0.07-0.16)	0.11(-0.01-0.23)	7703.74(11500.12-4780.63)	12062.72(18301.36-7628.83)	1.23(0.76-1.83)	1.1(0.69-1.68)	-0.24(-0.35--0.13)
		Male	175.56(263.91-107.53)	200.33(303.07-123.11)	0.05(0.03-0.07)	0.04(0.02-0.06)	-0.19(-0.46-0.08)	3151.43(4708.26-1987.81)	6362.81(9721.83-3932.53)	0.78(0.49-1.16)	0.71(0.44-1.08)	-0.21(-0.32--0.1)
	Severe anemia	Both	65.39(96.2-41.91)	70.19(101.64-46.16)	0.01(0-0.01)	0.01(0-0.01)	-0.4(-0.55--0.24)	1418.66(2038.8-943.47)	2090.04(3053.99-1372.32)	0.13(0.09-0.19)	0.1(0.07-0.15)	-0.78(-0.96--0.6)
		Female	45.74(67.79-29.45)	48.75(71.66-32.47)	0.01(0.01-0.02)	0.01(0.01-0.01)	-0.2(-0.33--0.07)	887.64(1288.94-577.89)	1130.53(1668.02-728.85)	0.14(0.09-0.2)	0.1(0.07-0.15)	-0.9(-1.09--0.71)
		Male	19.65(29.55-12.46)	21.44(31.64-13.58)	0(0-0.01)	0(0-0)	-0.77(-1.07--0.47)	531.02(769.97-349.25)	959.51(1430.81-612.97)	0.13(0.09-0.19)	0.11(0.07-0.16)	-0.64(-0.8--0.48)
High-middle SDI	Anemia	Both	1715.94(2647.93-1075.4)	2103.12(3289.39-1292.86)	0.15(0.09-0.23)	0.15(0.09-0.23)	0.3(0.18-0.41)	19610.22(29476.02-12518.13)	31700.48(48289.77-19829.97)	1.88(1.21-2.82)	1.56(0.98-2.37)	-0.61(-0.65--0.56)
		Female	995.99(1520.49-628.48)	1402.17(2177.02-871.2)	0.17(0.11-0.26)	0.19(0.12-0.3)	0.81(0.69-0.94)	12457.71(18592.68-7999.4)	20380.1(30355.76-12830.44)	2.08(1.34-3.1)	1.83(1.15-2.74)	-0.36(-0.41--0.32)
		Male	719.95(1133.55-439.86)	700.95(1124.92-409.5)	0.13(0.08-0.21)	0.11(0.06-0.17)	-0.43(-0.54--0.33)	7152.51(10941.89-4415.59)	11320.39(17799.87-6923.54)	1.72(1.06-2.58)	1.3(0.8-2.02)	-1.01(-1.09--0.92)
	Mild anemia	Both	149.17(345.15-51.99)	266.11(591.82-92.07)	0.01(0-0.03)	0.02(0.01-0.04)	1.59(1.41-1.78)	1745.95(3849.98-606.27)	4086.21(9098.99-1404.37)	0.17(0.06-0.36)	0.2(0.07-0.45)	0.89(0.84-0.95)
		Female	71.86(163.02-25.14)	137.11(314.17-48.08)	0.01(0-0.03)	0.02(0.01-0.04)	2.14(1.96-2.32)	737.34(1616.36-257.51)	1691.99(3776.34-595.43)	0.12(0.04-0.27)	0.15(0.05-0.34)	1.11(1.02-1.19)
		Male	77.31(177.9-26.66)	129(290.83-43.99)	0.01(0-0.03)	0.02(0.01-0.04)	1.09(0.89-1.29)	1008.61(2221.43-351.46)	2394.21(5322.69-817)	0.23(0.08-0.52)	0.27(0.09-0.6)	0.63(0.59-0.67)
	Moderate anemia	Both	1325.61(2040.09-831.22)	1614.88(2415.15-1013.62)	0.12(0.07-0.18)	0.12(0.07-0.18)	0.32(0.21-0.42)	14517.23(21682.41-9265.97)	23664.94(35870.66-14953.71)	1.39(0.89-2.07)	1.17(0.74-1.76)	-0.57(-0.62--0.53)
		Female	781.88(1194.35-486.65)	1119.85(1700.28-700.16)	0.13(0.08-0.2)	0.16(0.1-0.24)	0.88(0.76-1.01)	9615.19(14395.41-6151.83)	16245.37(24377.42-10280.91)	1.61(1.03-2.39)	1.46(0.93-2.19)	-0.25(-0.3--0.2)
		Male	543.73(845.07-332.9)	495.03(758.12-294.08)	0.1(0.06-0.15)	0.08(0.05-0.12)	-0.53(-0.62--0.44)	4902.04(7302.7-3060.69)	7419.57(11262.01-4730.8)	1.18(0.74-1.77)	0.85(0.54-1.28)	-1.17(-1.26--1.07)
	Severe anemia	Both	241.16(361.22-151.9)	222.13(333.16-141.82)	0.02(0.01-0.03)	0.02(0.01-0.02)	-0.88(-0.97--0.79)	3347.04(4840.18-2242.03)	3949.33(5667.84-2573.53)	0.33(0.22-0.47)	0.19(0.13-0.28)	-1.84(-1.94--1.74)
		Female	142.25(215.53-89.39)	145.21(221.12-93.11)	0.02(0.02-0.04)	0.02(0.01-0.03)	-0.51(-0.61--0.41)	2105.18(3084-1379.72)	2442.73(3477.72-1601.1)	0.35(0.23-0.51)	0.22(0.14-0.31)	-1.69(-1.79--1.6)

		Male	98.91(148.87-61.43)	76.92(117.21-47.37)	0.02(0.01-0.03)	0.01(0.01-0.02)	-1.39(-1.5--1.28)	1241.86(1778.34-831.7)	1506.6(2197.97-985.29)	0.31(0.21-0.44)	0.17(0.11-0.25)	-2.06(-2.18--1.95)
Low SDI	Anemia	Both	1857.91(2789.06-1192.03)	4660.31(7007.32-2946)	0.32(0.21-0.48)	0.38(0.24-0.56)	0.97(0.79-1.15)	7969.21(11448.39-5275.34)	19363.88(27951.99-12624.63)	3.44(2.26-4.98)	3.93(2.59-5.66)	0.5(0.46-0.54)
		Female	924.31(1397.12-601.46)	2466.51(3688.87-1547.61)	0.35(0.23-0.52)	0.42(0.27-0.63)	1.1(0.9-1.31)	4607.11(6600.95-3029.14)	11462.5(16548.97-7611.64)	3.79(2.48-5.42)	4.33(2.86-6.21)	0.49(0.46-0.51)
		Male	933.6(1420.41-584.3)	2193.8(3311.32-1376.69)	0.3(0.19-0.45)	0.33(0.21-0.5)	0.81(0.65-0.96)	3362.1(4901.22-2213.75)	7901.38(11667.07-5183.56)	3.09(2.05-4.46)	3.53(2.32-5.2)	0.51(0.44-0.57)
	Mild anemia	Both	50.14(113.01-17.31)	176.97(392.57-60.72)	0.01(0-0.02)	0.02(0.01-0.04)	2.19(1.94-2.45)	241.64(533.64-84.51)	784.74(1703.1-272.72)	0.1(0.03-0.21)	0.15(0.05-0.32)	1.58(1.49-1.67)
		Female	22.99(52.51-8.04)	88.49(197.33-31.28)	0.01(0-0.02)	0.02(0.01-0.04)	2.34(2.1-2.58)	97.9(218.4-34.17)	311.03(679.88-109.05)	0.07(0.03-0.16)	0.11(0.04-0.23)	1.38(1.3-1.47)
		Male	27.16(61.15-9.58)	88.48(201.15-30.64)	0.01(0-0.03)	0.02(0.01-0.04)	2.09(1.82-2.36)	143.74(314.38-50.44)	473.71(1024.75-165.45)	0.12(0.04-0.26)	0.19(0.07-0.41)	1.78(1.68-1.87)
	Moderate anemia	Both	1235.62(1886.69-767.03)	3376.29(5106.43-2079.94)	0.22(0.14-0.33)	0.27(0.17-0.41)	1.25(1.06-1.43)	4717.39(7087.22-3030.66)	12615.28(18745.69-7937.16)	2(1.29-2.97)	2.52(1.61-3.75)	0.89(0.86-0.92)
		Female	623.21(956.56-395.22)	1819.04(2771.26-1121.02)	0.24(0.15-0.36)	0.31(0.2-0.47)	1.4(1.2-1.61)	2813.78(4174.45-1835.15)	7692.71(11415.21-4944.37)	2.28(1.48-3.39)	2.86(1.83-4.26)	0.84(0.81-0.88)
		Male	612.41(951.89-373.22)	1557.25(2393.27-936.87)	0.19(0.12-0.3)	0.23(0.14-0.35)	1.04(0.88-1.21)	1903.61(2866.76-1219.67)	4922.58(7305.9-3113.52)	1.71(1.09-2.56)	2.17(1.37-3.18)	0.94(0.9-0.99)
	Severe anemia	Both	572.14(857.25-362.02)	1107.05(1650.21-696.76)	0.1(0.06-0.15)	0.09(0.06-0.13)	0.11(-0.05-0.27)	3010.18(4374.37-1992.59)	5963.86(8658.57-3973.93)	1.34(0.9-1.95)	1.26(0.84-1.82)	-0.24(-0.36--0.12)
		Female	278.11(415.87-174.62)	558.98(831.64-353.13)	0.1(0.07-0.16)	0.1(0.06-0.14)	0.17(-0.02-0.37)	1695.44(2452.9-1130.54)	3458.76(5030.09-2294.59)	1.44(0.97-2.07)	1.36(0.9-1.97)	-0.19(-0.27--0.11)
		Male	294.04(444.28-184.02)	548.07(821.76-345.65)	0.09(0.06-0.13)	0.08(0.05-0.12)	0.05(-0.09-0.19)	1314.74(1927.52-868.18)	2505.1(3638.65-1681.12)	1.26(0.84-1.84)	1.17(0.79-1.71)	-0.29(-0.45--0.13)
Low-middle SDI	Anemia	Both	3527.19(5245.07-2172.57)	5578.75(8234.86-3500.21)	0.29(0.18-0.43)	0.31(0.19-0.46)	0.65(0.49-0.81)	24761.63(35548.67-16123.1)	56078.81(82099.69-36356.49)	4.23(2.77-6.08)	4.27(2.78-6.23)	-0.11(-0.24-0.01)
		Female	1759.82(2637.45-1109.74)	3039.62(4547.45-1893.51)	0.3(0.19-0.45)	0.33(0.21-0.5)	0.75(0.58-0.91)	13483.45(19288.98-8903.02)	31698.57(45900.73-20845.16)	4.41(2.91-6.31)	4.51(2.96-6.52)	-0.06(-0.16-0.04)
		Male	1767.37(2636.14-1079.96)	2539.13(3834.79-1575.12)	0.28(0.18-0.41)	0.28(0.18-0.43)	0.54(0.39-0.7)	11278.18(16349.49-7344.22)	24380.25(36166.61-15686.29)	4.08(2.67-5.88)	4.06(2.63-6.01)	-0.18(-0.32--0.03)
	Mild anemia	Both	116(253.7-40.09)	297.77(654.69-103.14)	0.01(0-0.02)	0.02(0.01-0.04)	2.08(1.89-2.26)	819.33(1775.99-289.26)	2722.49(5958.83-953.14)	0.13(0.05-0.28)	0.2(0.07-0.44)	1.48(1.44-1.53)
		Female	52.19(115.18-17.75)	142.47(311.85-49.43)	0.01(0-0.02)	0.02(0.01-0.03)	2.15(2.01-2.29)	300.99(655.14-105.01)	999.12(2147.66-352.28)	0.09(0.03-0.2)	0.14(0.05-0.3)	1.44(1.42-1.47)
		Male	63.81(144.38-22.27)	155.3(345.62-53.78)	0.01(0-0.03)	0.02(0.01-0.04)	2.07(1.84-2.29)	518.33(1126.88-183.68)	1723.37(3819.6-592.29)	0.17(0.06-0.37)	0.27(0.09-0.6)	1.62(1.56-1.68)
	Moderate anemia	Both	2455.71(3707.88-1490.98)	4255.57(6310.14-2648.01)	0.2(0.12-0.3)	0.23(0.15-0.35)	0.97(0.82-1.12)	15344.66(22845.44-9837.93)	38269.14(57170.19-24005.83)	2.58(1.64-3.82)	2.9(1.81-4.3)	0.3(0.2-0.4)
		Female	1228.01(1880.35-748.56)	2349.38(3554.25-1458.53)	0.21(0.13-0.32)	0.26(0.16-0.39)	1.11(0.96-1.27)	8462.37(12573.74-5483.14)	22051.85(32740.75-14002.1)	2.73(1.75-4.06)	3.12(1.99-4.61)	0.34(0.26-0.42)
		Male	1227.7(1858.31-731.39)	1906.19(2894.72-1161.05)	0.19(0.12-0.29)	0.21(0.13-0.32)	0.81(0.66-0.96)	6882.29(10403.96-4386.35)	16217.3(24191.58-10234.12)	2.43(1.54-3.64)	2.68(1.69-3.99)	0.24(0.11-0.37)
	Severe anemia	Both	955.48(1449.4-599.46)	1025.41(1516.34-642.92)	0.08(0.05-0.12)	0.06(0.04-0.09)	-0.61(-0.79--0.42)	8597.64(12302.46-5804.56)	15087.18(21562.83-10254.08)	1.52(1.04-2.19)	1.17(0.8-1.67)	-1.1(-1.28--0.91)
		Female	479.62(737.49-301.87)	547.77(816.29-348.56)	0.09(0.05-0.13)	0.06(0.04-0.09)	-0.61(-0.81--0.41)	4720.08(6714.91-3165.3)	8647.6(12313.08-5783.5)	1.59(1.07-2.24)	1.25(0.84-1.79)	-0.97(-1.13--0.8)
		Male	475.86(719.35-296.73)	477.64(746.61-293.49)	0.08(0.05-0.11)	0.05(0.03-0.08)	-0.59(-0.77--0.41)	3877.55(5543.17-2588.34)	6439.58(9245.83-4314.86)	1.48(1-2.12)	1.1(0.74-1.58)	-1.26(-1.48--1.05)
Middle SDI	Anemia	Both	3498.75(5229.29-2204.42)	4568.44(6959.06-2868.33)	0.19(0.12-0.28)	0.2(0.12-0.3)	0.58(0.43-0.74)	32169.14(47628.84-20591.39)	68669.1(103825.27-42975.49)	3.29(2.1-4.82)	2.89(1.84-4.35)	-0.34(-0.38--0.3)
		Female	1936.12(2916.56-1234.57)	2817.61(4281.2-1766.29)	0.21(0.13-0.32)	0.24(0.15-0.36)	0.89(0.71-1.08)	19789.44(29060.28-12653.21)	42847.66(64248.44-26991.04)	3.72(2.38-5.41)	3.36(2.12-5.04)	-0.22(-0.27--0.16)
		Male	1562.63(2339.79-966.77)	1750.83(2668.01-1085.56)	0.17(0.1-0.25)	0.16(0.1-0.24)	0.14(0.02-0.26)	12379.7(18631.59-7861.28)	25821.43(39939.65-16102.87)	2.87(1.86-4.27)	2.42(1.51-3.71)	-0.55(-0.62--0.48)
	Mild anemia	Both	205.26(462.08-70.03)	417.39(921.87-141.69)	0.01(0-0.03)	0.02(0.01-0.04)	1.89(1.72-2.06)	1979.15(4339.75-691.87)	6496.8(13983.34-2279.22)	0.19(0.07-0.42)	0.27(0.09-0.58)	1.35(1.27-1.44)
		Female	103.97(234.65-35.72)	221.76(510.99-74.84)	0.01(0-0.02)	0.02(0.01-0.04)	2.21(2.05-2.36)	798.73(1745.23-276.7)	2638.61(5737.04-923.49)	0.14(0.05-0.3)	0.2(0.07-0.45)	1.57(1.45-1.7)
		Male	101.29(224.83-34.66)	195.63(441.54-66.67)	0.01(0-0.03)	0.02(0.01-0.04)	1.6(1.41-1.79)	1180.41(2582.46-412.43)	3858.19(8279.14-1362.33)	0.25(0.09-0.54)	0.34(0.12-0.73)	1.22(1.16-1.27)
	Moderate anemia	Both	2699.24(4075.3-1680.46)	3547.21(5330.49-2226.11)	0.14(0.09-0.22)	0.15(0.1-0.23)	0.62(0.47-0.76)	22928.57(34182.36-14527.07)	50108.15(75201.2-31391.69)	2.32(1.47-3.41)	2.11(1.33-3.14)	-0.23(-0.27--0.19)
		Female	1505.76(2307.75-939.61)	2237.8(3414.51-1404.08)	0.16(0.1-0.25)	0.19(0.12-0.29)	0.96(0.78-1.14)	14471.42(21666.71-9223.55)	32777.79(48729.71-20507.55)	2.7(1.7-4)	2.57(1.61-3.81)	-0.04(-0.1-0.02)
		Male	1193.48(1811.22-718.45)	1309.41(1961.58-799.23)	0.13(0.08-0.19)	0.12(0.07-0.18)	0.1(-0.01-0.2)	8457.16(12624.81-5339.17)	17330.36(25881.55-10968.32)	1.94(1.24-2.87)	1.63(1.04-2.42)	-0.56(-0.63--0.49)
	Severe anemia	Both	594.25(866.5-389)	603.84(878.94-402.37)	0.03(0.02-0.05)	0.03(0.02-0.04)	-0.2(-0.4--0.01)	7261.42(10581.23-4826.75)	12064.14(17251.9-8143.31)	0.78(0.52-1.14)	0.52(0.35-0.74)	-1.32(-1.39--1.26)
		Female	326.4(475.8-215.15)	358.05(518.81-238.62)	0.04(0.02-0.05)	0.03(0.02-0.04)	-0.04(-0.28-0.19)	4519.29(6522.87-2996.67)	7431.26(10631.46-5034.28)	0.88(0.59-1.28)	0.59(0.4-0.85)	-1.25(-1.3--1.2)
		Male	267.85(395.92-170.69)	245.79(367.82-157.3)	0.03(0.02-0.04)	0.02(0.01-0.03)	-0.43(-0.59--0.28)	2742.13(4028.8-1841.5)	4632.89(6751.53-3076.11)	0.68(0.46-0.99)	0.45(0.3-0.65)	-1.43(-1.53--1.34)
Andean Latin America	Anemia	Both	103.35(211.09-47.75)	161.09(310.37-75.76)	0.25(0.12-0.48)	0.25(0.12-0.47)	0.25(0.12-0.39)	437.13(661.21-283.51)	1274.63(1955.32-786.17)	2.1(1.36-3.15)	2.31(1.43-3.53)	0.29(0.26-0.33)
		Female	62.14(131.97-28.63)	102.93(196.97-50.42)	0.31(0.15-0.63)	0.31(0.16-0.6)	0.36(0.19-0.53)	271.98(408.46-176.73)	741.73(1115.41-457.92)	2.4(1.54-3.63)	2.54(1.57-3.81)	0.18(0.14-0.21)
		Male	41.21(82.7-18.6)	58.17(117.78-23.41)	0.18(0.09-0.35)	0.18(0.07-0.36)	0.1(0.01-0.2)	165.16(249.47-103.32)	532.9(817.89-324.73)	1.79(1.12-2.67)	2.07(1.26-3.17)	0.45(0.41-0.48)

	Mild anemia	Both	5.17(12.77-1.55)	16.14(39.99-5.04)	0.01(0-0.03)	0.02(0.01-0.06)	2.61(2.39-2.83)	27.06(60.74-9.56)	135.01(289.76-47.41)	0.13(0.05-0.29)	0.24(0.09-0.52)	2.15(1.97-2.34)
		Female	2.86(7.35-0.82)	10.53(27.09-3.09)	0.01(0-0.04)	0.03(0.01-0.08)	3.27(3.03-3.52)	11.37(25.3-4.07)	55.4(120.32-19.22)	0.1(0.03-0.22)	0.19(0.07-0.41)	2.17(1.94-2.41)
		Male	2.31(5.55-0.71)	5.61(13.64-1.86)	0.01(0-0.03)	0.02(0.01-0.04)	1.67(1.49-1.86)	15.69(35.23-5.54)	79.61(174.04-27.6)	0.16(0.06-0.36)	0.3(0.11-0.66)	2.17(2.03-2.31)
	Moderate anemia	Both	79.93(162.85-36.22)	128.57(247.17-60.55)	0.19(0.09-0.37)	0.2(0.09-0.38)	0.36(0.22-0.5)	308.21(468.06-194.94)	924.58(1396.49-571.28)	1.47(0.93-2.23)	1.67(1.03-2.52)	0.39(0.33-0.45)
		Female	47.4(100.46-21.35)	81.87(158.92-39.44)	0.24(0.11-0.48)	0.25(0.12-0.49)	0.49(0.32-0.65)	199.5(302.66-125.93)	573.11(868.81-349.13)	1.75(1.12-2.68)	1.96(1.19-2.97)	0.34(0.27-0.41)
		Male	32.53(67.35-13.95)	46.69(98.4-17.62)	0.14(0.07-0.27)	0.15(0.06-0.3)	0.19(0.07-0.3)	108.71(163.02-65.92)	351.47(536.9-212.6)	1.17(0.71-1.76)	1.37(0.83-2.09)	0.45(0.4-0.5)
	Severe anemia	Both	18.25(35.52-9.11)	16.39(29.62-8.69)	0.05(0.02-0.08)	0.03(0.01-0.05)	-1.67(-1.91--1.44)	101.86(150.62-66.18)	215.05(309.97-139.67)	0.5(0.32-0.75)	0.39(0.26-0.57)	-0.79(-0.89--0.7)
		Female	11.89(24.49-5.75)	10.53(18.95-5.62)	0.06(0.03-0.12)	0.03(0.02-0.06)	-1.79(-2.12--1.46)	61.11(91.39-39.8)	113.23(171.47-71.68)	0.55(0.35-0.82)	0.39(0.25-0.59)	-1.09(-1.22--0.95)
		Male	6.36(12.67-2.9)	5.86(11.16-2.86)	0.03(0.01-0.06)	0.02(0.01-0.03)	-1.39(-1.49--1.29)	40.76(60.41-24.94)	101.82(154.4-63.35)	0.45(0.28-0.68)	0.4(0.25-0.6)	-0.47(-0.53--0.41)
Australasia	Anemia	Both	22.89(43.88-11.83)	36.28(67.15-18.75)	0.11(0.05-0.21)	0.12(0.06-0.23)	0.57(0.36-0.77)	262.16(409.58-155.37)	456.91(737.73-258.74)	1.12(0.67-1.74)	0.86(0.49-1.37)	-0.98(-1.13--0.83)
		Female	17.6(33.45-8.88)	28.39(52.58-14.29)	0.16(0.08-0.31)	0.18(0.09-0.35)	0.73(0.49-0.97)	184.23(283.89-103.83)	284.06(450.2-156.33)	1.35(0.78-2.07)	1.01(0.57-1.6)	-1.07(-1.23--0.91)
		Male	5.29(11.07-2.48)	7.89(16.33-3.64)	0.06(0.03-0.12)	0.05(0.02-0.11)	-0.07(-0.21-0.08)	77.93(135.97-40.79)	172.85(312.51-90.64)	0.88(0.48-1.48)	0.71(0.38-1.28)	-0.79(-0.91--0.67)
	Mild anemia	Both	4.02(10.63-1.33)	7.52(19.37-2.42)	0.02(0.01-0.05)	0.02(0.01-0.06)	1.14(0.88-1.39)	42.99(95.69-15.07)	93.01(206.36-33.37)	0.18(0.06-0.4)	0.18(0.06-0.39)	-0.14(-0.21--0.07)
		Female	2.52(7.01-0.81)	4.78(12.74-1.47)	0.02(0.01-0.07)	0.03(0.01-0.09)	1.33(1.03-1.62)	21.1(47.47-7.57)	39.33(90.18-13.46)	0.15(0.06-0.35)	0.14(0.05-0.33)	-0.38(-0.46--0.3)
		Male	1.5(3.72-0.47)	2.74(6.87-0.81)	0.01(0-0.04)	0.02(0-0.04)	0.74(0.5-0.98)	21.89(50.15-7.39)	53.67(124.77-19.17)	0.23(0.08-0.51)	0.22(0.08-0.51)	-0.18(-0.25--0.11)
	Moderate anemia	Both	17.34(31.64-8.81)	26.59(47.86-14.3)	0.08(0.04-0.16)	0.09(0.04-0.16)	0.46(0.26-0.66)	194.08(297.58-117.23)	324.51(511.62-188.03)	0.84(0.51-1.27)	0.61(0.36-0.96)	-1.14(-1.3--0.98)
		Female	13.81(25.08-7.06)	21.86(39.93-11.33)	0.13(0.06-0.23)	0.14(0.07-0.26)	0.66(0.43-0.89)	146.61(227.55-83.99)	222.64(353.72-124.13)	1.07(0.62-1.65)	0.79(0.45-1.25)	-1.13(-1.3--0.97)
		Male	3.52(7.43-1.59)	4.74(9.15-2.16)	0.04(0.02-0.08)	0.03(0.01-0.07)	-0.38(-0.49--0.27)	47.47(80.81-25.03)	101.87(182.58-53.81)	0.55(0.3-0.91)	0.42(0.22-0.75)	-1(-1.14--0.86)
	Severe anemia	Both	1.53(2.67-0.86)	2.17(3.75-1.19)	0.01(0-0.01)	0.01(0-0.01)	0.12(-0.09-0.32)	25.09(39.12-15.05)	39.39(60.27-22.31)	0.11(0.07-0.17)	0.07(0.04-0.11)	-1.4(-1.61--1.19)
		Female	1.27(2.25-0.68)	1.75(3.22-0.91)	0.01(0.01-0.02)	0.01(0.01-0.02)	0.16(-0.05-0.37)	16.52(28.19-8.87)	22.09(36.3-11.27)	0.12(0.07-0.2)	0.08(0.04-0.12)	-1.57(-1.81--1.34)
		Male	0.26(0.47-0.14)	0.42(0.8-0.21)	0(0-0)	0(0-0)	-0.28(-0.54--0.03)	8.57(15.47-4.29)	17.3(31.26-8.51)	0.1(0.05-0.18)	0.07(0.03-0.13)	-1.28(-1.44--1.13)
Caribbean	Anemia	Both	71.54(123.36-37.5)	122.17(223.92-63.98)	0.19(0.1-0.32)	0.27(0.14-0.5)	1.32(1.27-1.37)	722.03(1114.14-450.61)	1751.44(2686.61-1094)	2.75(1.71-4.19)	3.39(2.12-5.19)	0.61(0.54-0.67)
		Female	39.89(71.17-20.88)	73.2(132.98-37.57)	0.21(0.11-0.36)	0.32(0.16-0.59)	1.56(1.49-1.63)	441.72(662.3-274.76)	1076.69(1638.15-668.99)	3.18(1.96-4.74)	3.95(2.45-5.99)	0.62(0.55-0.69)
		Male	31.65(55.23-16.32)	48.97(90.25-24.7)	0.17(0.09-0.29)	0.22(0.11-0.41)	1.02(0.97-1.07)	280.31(443.4-170.07)	674.75(1076.08-407.79)	2.29(1.39-3.61)	2.81(1.69-4.49)	0.59(0.54-0.65)
	Mild anemia	Both	4.75(11.14-1.55)	9.91(22.75-3.37)	0.01(0-0.03)	0.02(0.01-0.05)	1.84(1.69-2)	76.33(171-26.62)	232.33(508.11-82.89)	0.29(0.1-0.65)	0.45(0.16-0.98)	1.45(1.33-1.57)
		Female	2.14(4.98-0.65)	4.36(10.18-1.42)	0.01(0-0.03)	0.02(0.01-0.04)	2(1.83-2.16)	28.95(63.96-10.02)	85.59(192.09-29.56)	0.21(0.07-0.46)	0.31(0.11-0.7)	1.36(1.25-1.47)
		Male	2.6(5.75-0.87)	5.55(12.22-1.86)	0.02(0.01-0.03)	0.02(0.01-0.05)	1.75(1.61-1.9)	47.38(104.79-16.44)	146.74(322.35-52.19)	0.38(0.13-0.84)	0.61(0.22-1.34)	1.54(1.42-1.67)
	Moderate anemia	Both	57.53(100.99-29.98)	98.78(184.1-51.58)	0.15(0.08-0.26)	0.22(0.11-0.42)	1.38(1.32-1.43)	550.12(831.92-341.23)	1326.48(2002.88-832.24)	2.09(1.3-3.15)	2.57(1.61-3.87)	0.58(0.51-0.64)
		Female	31.87(57.1-16.5)	60.2(109.67-30.83)	0.17(0.09-0.29)	0.26(0.13-0.49)	1.7(1.63-1.77)	353.18(532.29-218.36)	870.32(1290.48-538.41)	2.55(1.57-3.83)	3.19(1.97-4.72)	0.65(0.58-0.72)
		Male	25.66(45.31-13.14)	38.59(73.4-19.38)	0.14(0.07-0.24)	0.18(0.09-0.34)	0.96(0.92-1.01)	196.95(300.58-122.37)	456.16(703.28-285.07)	1.61(1.01-2.47)	1.9(1.19-2.92)	0.44(0.38-0.49)
	Severe anemia	Both	9.26(17.27-5)	13.48(25.91-7.03)	0.02(0.01-0.04)	0.03(0.02-0.06)	0.61(0.52-0.71)	95.58(139.33-61.84)	192.63(279.38-124.68)	0.36(0.23-0.53)	0.37(0.24-0.54)	-0.03(-0.09-0.03)
		Female	5.87(11.52-2.97)	8.65(17.33-4.31)	0.03(0.02-0.06)	0.04(0.02-0.08)	0.57(0.43-0.72)	59.6(88.02-37.9)	120.78(176.76-77.75)	0.43(0.27-0.63)	0.45(0.29-0.65)	-0.01(-0.09-0.07)
		Male	3.38(6.32-1.82)	4.84(9.74-2.49)	0.02(0.01-0.03)	0.02(0.01-0.04)	0.72(0.66-0.79)	35.98(54.08-23.15)	71.85(105.73-44.88)	0.29(0.19-0.44)	0.3(0.19-0.44)	-0.05(-0.09-0)
Central Asia	Anemia	Both	253.41(444.7-137.07)	392.51(684.5-217.36)	0.35(0.2-0.6)	0.41(0.23-0.71)	0.7(0.45-0.95)	1274.93(1890.71-819.36)	2611.03(3887.55-1682.2)	2.57(1.66-3.78)	3.42(2.23-5.08)	1.12(1.02-1.23)
		Female	166.67(309.54-86.27)	261.18(460.69-137.4)	0.46(0.24-0.84)	0.53(0.28-0.96)	0.65(0.33-0.96)	908.46(1353.15-576.67)	1720.83(2577.27-1126.99)	3.11(1.99-4.62)	3.86(2.53-5.74)	0.84(0.72-0.96)
		Male	86.75(146.44-47.86)	131.32(220.01-72.83)	0.25(0.15-0.41)	0.29(0.16-0.47)	0.86(0.67-1.04)	366.47(547.07-234.16)	890.21(1362.42-555.85)	2.07(1.33-3.05)	3.1(1.97-4.66)	1.56(1.47-1.65)
	Mild anemia	Both	13.3(33.35-4.42)	29.03(69.9-9.48)	0.02(0.01-0.05)	0.03(0.01-0.07)	2.14(1.81-2.47)	67.09(149.19-23.16)	185.45(408.5-64.42)	0.14(0.05-0.3)	0.25(0.09-0.54)	2.34(2.11-2.57)
		Female	7.53(19.61-2.38)	16.03(40.66-5.05)	0.02(0.01-0.05)	0.03(0.01-0.08)	2.18(1.87-2.49)	34.22(74.13-11.85)	80.25(177.23-27.79)	0.12(0.04-0.25)	0.18(0.06-0.4)	1.87(1.6-2.14)
		Male	5.77(13.9-1.95)	13(30.76-4.37)	0.02(0.01-0.05)	0.03(0.01-0.07)	2.13(1.76-2.5)	32.87(72.74-11.47)	105.2(239.69-36.23)	0.17(0.06-0.38)	0.34(0.12-0.75)	2.64(2.41-2.86)
	Moderate anemia	Both	199.8(358.44-105.74)	311.36(542.8-168.42)	0.28(0.15-0.48)	0.32(0.18-0.56)	0.73(0.47-0.99)	956.49(1434.3-611.42)	2015.34(3023.7-1286.76)	1.92(1.23-2.88)	2.64(1.68-3.93)	1.25(1.13-1.38)

		Female	134.24(252.4-68.04)	213.65(384.11-111.89)	0.37(0.19-0.68)	0.44(0.23-0.79)	0.69(0.35-1.02)	703.7(1069.52-442.91)	1392.72(2104.05-898.22)	2.41(1.52-3.65)	3.12(2.01-4.67)	1.03(0.89-1.17)
		Male	65.56(112.72-35.25)	97.71(166.47-53.91)	0.19(0.11-0.31)	0.21(0.12-0.35)	0.87(0.7-1.05)	252.79(379.19-160.4)	622.62(957.83-390.44)	1.44(0.92-2.14)	2.2(1.4-3.28)	1.66(1.56-1.75)
	Severe anemia	Both	40.32(66.67-23.24)	52.12(84.64-31.19)	0.06(0.03-0.09)	0.05(0.03-0.09)	-0.04(-0.21-0.14)	251.36(364.25-165.96)	410.24(582.79-267.12)	0.51(0.34-0.74)	0.54(0.36-0.76)	0.19(0.16-0.22)
		Female	24.9(42.98-13.85)	31.51(52.4-18.01)	0.07(0.04-0.12)	0.06(0.04-0.11)	-0.16(-0.37-0.05)	170.55(253.37-108.47)	247.85(364.75-153.92)	0.59(0.37-0.87)	0.56(0.35-0.8)	-0.27(-0.31--0.23)
		Male	15.42(25.3-8.77)	20.61(31.82-12.54)	0.05(0.03-0.07)	0.05(0.03-0.07)	0.2(0.05-0.35)	80.81(116.83-53.34)	162.39(232.55-105.39)	0.46(0.3-0.66)	0.56(0.37-0.8)	0.75(0.72-0.78)
Central Europe	Anemia	Both	144.16(222.56-85.42)	141.86(221.68-83.79)	0.12(0.07-0.19)	0.13(0.08-0.21)	0.63(0.52-0.73)	1141.73(1770.7-714.17)	1488.28(2361.31-907.1)	0.79(0.49-1.21)	0.78(0.48-1.23)	-0.11(-0.17--0.06)
		Female	79.5(124.2-46.98)	95.26(148.36-56.48)	0.13(0.08-0.21)	0.18(0.1-0.28)	1.43(1.3-1.56)	637.21(989.49-393.64)	821(1288.36-506.77)	0.82(0.51-1.27)	0.87(0.54-1.37)	0.26(0.18-0.33)
		Male	64.67(101.69-37.64)	46.6(75.63-26.21)	0.11(0.06-0.18)	0.09(0.05-0.14)	-0.53(-0.62--0.44)	504.52(789.82-303.79)	667.28(1074.63-390.42)	0.84(0.52-1.3)	0.78(0.45-1.25)	-0.49(-0.58--0.4)
	Mild anemia	Both	16.1(36.24-5.43)	23.11(51.61-7.93)	0.01(0-0.03)	0.02(0.01-0.04)	2.02(1.84-2.2)	155.5(341.62-54.11)	266.51(591.79-92.51)	0.11(0.04-0.24)	0.14(0.05-0.3)	0.78(0.72-0.83)
		Female	7.09(16.03-2.34)	11.24(25.17-3.82)	0.01(0-0.03)	0.02(0.01-0.05)	2.55(2.39-2.71)	50.52(115.02-17.72)	83.94(183.94-28.93)	0.07(0.02-0.15)	0.09(0.03-0.19)	1.04(1-1.07)
		Male	9.01(20.81-3.11)	11.87(26.68-4.13)	0.01(0.01-0.03)	0.02(0.01-0.04)	1.51(1.3-1.73)	104.98(231.77-36.53)	182.57(401.98-63.32)	0.17(0.06-0.37)	0.21(0.07-0.46)	0.67(0.58-0.76)
	Moderate anemia	Both	112.63(172.15-67.34)	106.92(163.1-64.9)	0.1(0.06-0.15)	0.1(0.06-0.16)	0.52(0.42-0.62)	829.23(1291.16-523.95)	1063.51(1643.01-661.75)	0.57(0.36-0.88)	0.56(0.35-0.86)	-0.13(-0.18--0.08)
		Female	63.77(98.47-37.57)	76.02(118.07-45.86)	0.11(0.06-0.17)	0.14(0.08-0.22)	1.4(1.27-1.53)	504.56(776.46-314.32)	659.73(1029.15-410.05)	0.65(0.4-1)	0.7(0.44-1.1)	0.32(0.25-0.39)
		Male	48.85(75.9-29.09)	30.9(47.7-18.75)	0.09(0.05-0.14)	0.06(0.04-0.1)	-0.86(-0.94--0.78)	324.67(496.06-200.73)	403.78(618.62-248.09)	0.54(0.34-0.83)	0.47(0.29-0.73)	-0.71(-0.81--0.62)
	Severe anemia	Both	15.43(23.58-9.49)	11.82(17.84-7.61)	0.01(0.01-0.02)	0.01(0.01-0.02)	-0.44(-0.57--0.32)	157(230.86-101.71)	158.25(229.46-102.5)	0.11(0.07-0.16)	0.08(0.05-0.12)	-1.16(-1.25--1.08)
		Female	8.63(13.42-5.26)	8(12.49-5.08)	0.01(0.01-0.02)	0.01(0.01-0.02)	0.35(0.2-0.51)	82.13(125.03-51.19)	77.33(113.86-49.09)	0.11(0.07-0.16)	0.08(0.05-0.12)	-0.84(-0.97--0.71)
		Male	6.8(10.64-4.08)	3.82(5.77-2.4)	0.01(0.01-0.02)	0.01(0-0.01)	-1.62(-1.69--1.54)	74.86(111.72-48.27)	80.93(118.92-51.47)	0.13(0.08-0.19)	0.09(0.06-0.14)	-1.41(-1.52--1.31)
Central Latin America	Anemia	Both	272.69(430.15-162.6)	372.47(582.92-227.47)	0.14(0.09-0.22)	0.15(0.09-0.24)	0.38(0.33-0.42)	2782.42(4224.88-1794.01)	7608.5(11579.87-4725.03)	3.4(2.19-5.16)	3.3(2.04-5.03)	-0.09(-0.11--0.07)
		Female	154.86(245.88-91.02)	232.14(371.35-140.52)	0.16(0.1-0.25)	0.18(0.11-0.29)	0.6(0.53-0.67)	1780.97(2667.67-1147.95)	4581.91(6883.22-2890.79)	4.01(2.56-5.99)	3.64(2.3-5.47)	-0.32(-0.35--0.29)
		Male	117.82(188.8-67.19)	140.33(221.29-84.2)	0.12(0.07-0.19)	0.12(0.07-0.19)	0.08(0.02-0.15)	1001.46(1527.23-634.77)	3026.59(4627.59-1865.85)	2.74(1.77-4.15)	2.93(1.81-4.47)	0.24(0.22-0.27)
	Mild anemia	Both	21.79(53.62-7.48)	43.22(103.86-15.03)	0.01(0-0.03)	0.02(0.01-0.04)	1.33(1.21-1.45)	250.08(546.33-86.65)	921.72(2031.59-325.73)	0.29(0.1-0.63)	0.39(0.14-0.86)	0.96(0.86-1.05)
		Female	12.98(32.56-4.45)	26.65(64.55-9.26)	0.01(0-0.03)	0.02(0.01-0.05)	1.53(1.38-1.67)	113.67(253.49-39.83)	388.84(858.31-137.36)	0.24(0.08-0.52)	0.31(0.11-0.68)	0.75(0.65-0.85)
		Male	8.81(21.26-2.95)	16.57(38.61-5.76)	0.01(0-0.02)	0.01(0-0.03)	1.12(1.04-1.21)	136.41(301.29-46.73)	532.88(1180.65-186.67)	0.34(0.12-0.75)	0.5(0.17-1.11)	1.17(1.09-1.26)
	Moderate anemia	Both	217.15(339.87-129.35)	293.72(450.92-179.79)	0.11(0.07-0.17)	0.12(0.07-0.18)	0.38(0.33-0.42)	2008.69(2990.2-1284.25)	5483.11(8126.51-3415.07)	2.43(1.56-3.64)	2.38(1.48-3.52)	-0.09(-0.11--0.06)
		Female	121.99(194.66-71.71)	183.02(285.46-111.04)	0.13(0.08-0.2)	0.14(0.09-0.22)	0.61(0.55-0.68)	1359.33(2033.1-867.85)	3558.9(5242.66-2214.26)	3.05(1.94-4.61)	2.83(1.76-4.17)	-0.27(-0.29--0.25)
		Male	95.15(154.76-54.14)	110.71(175.59-66.02)	0.09(0.05-0.15)	0.09(0.06-0.15)	0.06(-0.01-0.13)	649.36(978.15-410.45)	1924.21(2872.6-1200.19)	1.77(1.13-2.66)	1.87(1.17-2.79)	0.19(0.16-0.22)
	Severe anemia	Both	33.75(51.04-20.89)	35.52(52.89-22.55)	0.02(0.01-0.03)	0.01(0.01-0.02)	-0.5(-0.62--0.37)	523.65(757.59-350.9)	1203.67(1714-795.58)	0.67(0.45-0.98)	0.53(0.35-0.76)	-0.72(-0.81--0.62)
		Female	19.89(30.12-12.3)	22.47(34.2-14.13)	0.02(0.01-0.03)	0.02(0.01-0.03)	-0.3(-0.43--0.17)	307.96(445.55-204.87)	634.17(905.29-421.68)	0.72(0.48-1.05)	0.51(0.34-0.73)	-1.09(-1.22--0.96)
		Male	13.86(21.87-8.34)	13.05(19.24-8.01)	0.01(0.01-0.02)	0.01(0.01-0.02)	-0.74(-0.89--0.59)	215.69(308.89-141.83)	569.51(810.85-378.21)	0.62(0.41-0.9)	0.56(0.37-0.81)	-0.25(-0.3--0.2)
Central Sub-Saharan Africa	Anemia	Both	150.59(332.16-66.62)	389.06(811.08-178.85)	0.24(0.12-0.49)	0.26(0.13-0.51)	0.52(0.36-0.69)	486.74(721.17-309.2)	1209.9(1807-763.56)	2.12(1.37-3.12)	2.44(1.53-3.6)	0.43(0.37-0.48)
		Female	78.24(164.32-34.84)	207.74(446.48-98.57)	0.27(0.13-0.54)	0.29(0.15-0.59)	0.6(0.39-0.82)	311.88(470.34-196.5)	793.34(1186.08-508.42)	2.38(1.54-3.49)	2.72(1.75-4.02)	0.39(0.31-0.47)
		Male	72.35(165.61-31.04)	181.32(385-79.4)	0.21(0.1-0.44)	0.22(0.1-0.44)	0.44(0.33-0.54)	174.86(259.15-109.19)	416.56(631.48-254.81)	1.82(1.15-2.68)	2.15(1.34-3.24)	0.51(0.47-0.56)
	Mild anemia	Both	3.8(9.82-1.25)	16.41(41.06-5.41)	0.01(0-0.02)	0.01(0-0.03)	2.33(2.05-2.61)	17.81(39.78-6.16)	69.56(155.8-24.16)	0.07(0.03-0.16)	0.12(0.04-0.28)	1.86(1.63-2.09)
		Female	1.88(5.04-0.59)	9.12(25.35-2.64)	0.01(0-0.02)	0.01(0-0.04)	2.59(2.29-2.88)	7.98(17.61-2.77)	32.19(73.41-11.38)	0.06(0.02-0.13)	0.1(0.03-0.22)	1.82(1.62-2.01)
		Male	1.91(5.01-0.62)	7.29(18.57-2.42)	0.01(0-0.02)	0.01(0-0.03)	2.05(1.78-2.32)	9.83(22-3.4)	37.37(83.69-12.9)	0.09(0.03-0.2)	0.16(0.05-0.35)	2.03(1.82-2.25)
	Moderate anemia	Both	105.39(228.74-47.6)	303.65(639.96-139.82)	0.17(0.08-0.34)	0.2(0.1-0.4)	0.9(0.74-1.07)	342.48(506.94-208.3)	912.8(1369.38-584.63)	1.49(0.92-2.21)	1.83(1.16-2.7)	0.67(0.6-0.74)
		Female	54.94(117.35-25.41)	163.55(354.8-78.38)	0.19(0.09-0.38)	0.23(0.12-0.47)	1.04(0.82-1.26)	224.12(335.35-137.54)	617.99(928.99-390.52)	1.73(1.07-2.56)	2.11(1.35-3.17)	0.65(0.56-0.75)
		Male	50.45(113.28-21.67)	140.1(296.6-61)	0.14(0.07-0.3)	0.17(0.08-0.33)	0.74(0.64-0.84)	118.36(179.62-69.72)	294.81(450.16-176.63)	1.22(0.75-1.85)	1.52(0.93-2.28)	0.72(0.67-0.76)
	Severe anemia	Both	41.4(90.65-17.06)	69(146-29.44)	0.06(0.03-0.13)	0.04(0.02-0.09)	-1.03(-1.23--0.84)	126.44(188.83-80.26)	227.53(350.46-141.25)	0.55(0.36-0.81)	0.49(0.31-0.74)	-0.56(-0.64--0.48)
		Female	21.42(47.05-8.85)	35.06(75.64-15.58)	0.07(0.03-0.15)	0.05(0.02-0.1)	-1.19(-1.37--1)	79.77(122.1-47.82)	143.16(220.18-86.82)	0.6(0.36-0.91)	0.51(0.31-0.79)	-0.64(-0.7--0.59)

		Male	19.99(47.21-7.37)	33.94(76.88-13.08)	0.05(0.02-0.12)	0.04(0.02-0.09)	-0.82(-1.05--0.59)	46.67(73.68-28.17)	84.38(134.55-51.37)	0.5(0.31-0.79)	0.47(0.28-0.76)	-0.36(-0.54--0.19)
East Asia	Anemia	Both	1114.8(1686.79-703.41)	570.56(902.53-339.27)	0.09(0.06-0.14)	0.04(0.02-0.06)	-2.56(-2.67--2.44)	19730.82(29574.34-12290.59)	21804.83(33595.94-12976.89)	2.45(1.56-3.64)	1.11(0.67-1.69)	-2.54(-2.69--2.39)
		Female	570.12(867.49-352.84)	329.47(509.37-192.45)	0.09(0.06-0.13)	0.04(0.03-0.07)	-2.21(-2.37--2.04)	11601.07(17293.43-7318.87)	13408.63(20289.04-8060.82)	2.61(1.66-3.88)	1.26(0.76-1.91)	-2.19(-2.33--2.05)
		Male	544.67(852.11-334.6)	241.09(398.06-141.32)	0.09(0.05-0.14)	0.04(0.02-0.06)	-2.96(-3.04--2.88)	8129.75(12415.83-5028.4)	8396.21(13773.52-4901.45)	2.37(1.52-3.56)	0.98(0.59-1.6)	-3.03(-3.26--2.8)
	Mild anemia	Both	78.62(179.62-26.4)	88.42(197.53-30.25)	0.01(0-0.01)	0.01(0-0.01)	0.19(0.01-0.38)	1371.97(2996.16-481.05)	3381.79(7351.93-1191.74)	0.16(0.05-0.34)	0.17(0.06-0.36)	0.71(0.56-0.87)
		Female	34.13(78.85-11.45)	37.73(84.57-13.24)	0.01(0-0.01)	0(0-0.01)	0.38(0.2-0.57)	527.52(1149.34-183.78)	1411.59(3107.36-497.18)	0.11(0.04-0.24)	0.13(0.05-0.29)	1.35(1.1-1.59)
		Male	44.49(102.29-14.84)	50.69(115.75-17.48)	0.01(0-0.02)	0.01(0-0.01)	0.06(-0.13-0.26)	844.45(1860.22-302.52)	1970.19(4301.94-690.61)	0.21(0.07-0.47)	0.21(0.08-0.46)	0.33(0.23-0.43)
	Moderate anemia	Both	867.77(1319.52-535.68)	420.99(634.79-255.28)	0.07(0.04-0.11)	0.03(0.02-0.05)	-2.7(-2.79--2.6)	14412.46(21532.31-9072.29)	15982.15(23857.04-9887.76)	1.78(1.12-2.63)	0.82(0.5-1.22)	-2.5(-2.64--2.37)
		Female	443.05(681.14-274.39)	256.72(389.34-153.28)	0.07(0.04-0.11)	0.03(0.02-0.05)	-2.21(-2.36--2.06)	8752.48(13101.67-5516.14)	10662.63(15920.48-6473.49)	1.96(1.24-2.91)	1(0.61-1.5)	-2(-2.14--1.87)
		Male	424.72(653.18-257.41)	164.28(257.35-100.15)	0.07(0.04-0.11)	0.03(0.02-0.04)	-3.26(-3.32--3.2)	5659.98(8510.63-3554.07)	5319.53(8098.58-3252.8)	1.64(1.04-2.45)	0.64(0.39-0.96)	-3.3(-3.53--3.07)
	Severe anemia	Both	168.41(258.12-107.96)	61.14(91.69-37.85)	0.01(0.01-0.02)	0(0-0.01)	-3.91(-4.08--3.74)	3946.39(5769.26-2593.43)	2440.89(3570.79-1554.03)	0.51(0.34-0.75)	0.13(0.08-0.18)	-4.84(-5.12--4.56)
		Female	92.94(141.74-55.83)	35.02(54.05-20.78)	0.01(0.01-0.02)	0(0-0.01)	-3.82(-4.06--3.58)	2321.07(3458.76-1492.38)	1334.4(2045.03-826.71)	0.54(0.34-0.79)	0.13(0.08-0.19)	-4.88(-5.14--4.63)
		Male	75.47(117.75-46.91)	26.12(39.05-16.23)	0.01(0.01-0.02)	0(0-0.01)	-4.05(-4.17--3.93)	1625.32(2374.87-1069.2)	1106.49(1649.97-694.64)	0.52(0.34-0.76)	0.13(0.09-0.2)	-4.77(-5.08--4.46)
Eastern Europe	Anemia	Both	463.44(721.42-287.98)	631.51(988.17-378.47)	0.2(0.12-0.31)	0.27(0.16-0.43)	1.62(1.36-1.88)	4208.08(6401.52-2637.19)	6435.78(9951.73-3965.23)	1.55(0.97-2.33)	1.85(1.13-2.85)	0.47(0.35-0.59)
		Female	275.36(426.93-172.98)	406.43(642.37-244.43)	0.23(0.14-0.36)	0.36(0.21-0.57)	2.26(1.86-2.66)	2789.49(4234.74-1756.43)	3877.19(6037.07-2408.34)	1.65(1.04-2.5)	1.84(1.13-2.88)	0.2(0.06-0.35)
		Male	188.09(299.75-112.12)	225.07(369.93-123.37)	0.17(0.1-0.27)	0.19(0.11-0.32)	0.72(0.51-0.94)	1418.59(2228.24-852.67)	2558.58(4135.08-1553.77)	1.57(0.96-2.38)	2.06(1.25-3.24)	0.92(0.85-0.99)
	Mild anemia	Both	52.56(120.47-18.41)	94.02(212.53-32.18)	0.02(0.01-0.05)	0.04(0.01-0.09)	2.96(2.57-3.35)	398.41(924.38-137.65)	781.76(1779.51-273.83)	0.14(0.05-0.33)	0.23(0.08-0.52)	1.9(1.77-2.02)
		Female	26.58(62.39-9.44)	47.33(107.93-16.6)	0.02(0.01-0.05)	0.05(0.02-0.1)	3.39(2.82-3.96)	208.2(480.26-74.24)	331.27(738.14-118.51)	0.12(0.04-0.28)	0.16(0.06-0.36)	1.2(1.07-1.33)
		Male	25.98(57.79-8.88)	46.69(106.03-15.73)	0.02(0.01-0.05)	0.04(0.01-0.09)	2.57(2.27-2.88)	190.21(429.28-65.14)	450.48(1045.66-154.25)	0.19(0.07-0.44)	0.35(0.12-0.79)	2.45(2.3-2.6)
	Moderate anemia	Both	356.33(538.91-225.07)	476.17(736.71-292.92)	0.15(0.1-0.23)	0.21(0.13-0.32)	1.54(1.28-1.79)	3230.13(4861.32-2038.99)	4906.59(7465.56-3024.55)	1.19(0.75-1.78)	1.41(0.88-2.14)	0.44(0.32-0.57)
		Female	219.86(333.98-139.71)	323.48(498.71-198.17)	0.18(0.12-0.28)	0.28(0.17-0.43)	2.22(1.83-2.61)	2239.31(3359.37-1413.39)	3138.26(4839.27-1972.91)	1.32(0.84-1.98)	1.49(0.94-2.27)	0.25(0.11-0.39)
		Male	136.47(213.94-81.59)	152.7(242.43-85.89)	0.13(0.08-0.2)	0.13(0.07-0.22)	0.47(0.27-0.67)	990.82(1541.11-601.2)	1768.33(2751.74-1071.54)	1.1(0.68-1.68)	1.43(0.88-2.23)	0.88(0.8-0.95)
	Severe anemia	Both	54.56(82-34.89)	61.32(93.81-36.72)	0.02(0.01-0.03)	0.02(0.01-0.04)	0.59(0.41-0.77)	579.54(863.97-367.36)	747.43(1094.57-462.42)	0.21(0.14-0.32)	0.21(0.13-0.31)	-0.48(-0.72--0.24)
		Female	28.92(45.69-17.91)	35.63(57.5-20.26)	0.02(0.01-0.04)	0.03(0.02-0.05)	1.28(1.01-1.55)	341.98(528.85-205.11)	407.66(627.11-238.98)	0.2(0.12-0.31)	0.19(0.11-0.29)	-0.75(-1.03--0.47)
		Male	25.63(39.89-15.33)	25.69(39.76-14.94)	0.02(0.01-0.04)	0.02(0.01-0.03)	-0.16(-0.43-0.12)	237.56(363.01-145.53)	339.77(521.7-209.96)	0.27(0.17-0.42)	0.28(0.17-0.43)	-0.2(-0.35--0.04)
Eastern Sub-Saharan Africa	Anemia	Both	541.98(854.76-330.62)	1279.34(2022.75-787.13)	0.27(0.17-0.41)	0.3(0.19-0.46)	0.72(0.55-0.9)	1564.28(2288.85-1008.9)	3720.8(5580.6-2375.53)	2.15(1.39-3.15)	2.46(1.57-3.64)	0.45(0.4-0.49)
		Female	260.36(409.74-158.41)	659.59(1029.43-402.35)	0.28(0.18-0.43)	0.33(0.2-0.51)	0.93(0.72-1.14)	980.16(1422.21-632.22)	2373.23(3488.9-1505.3)	2.5(1.63-3.65)	2.85(1.81-4.19)	0.43(0.37-0.49)
		Male	281.62(448.74-172.05)	619.75(985.76-367.83)	0.25(0.15-0.38)	0.26(0.16-0.41)	0.48(0.34-0.61)	584.12(874.12-370.8)	1347.57(2071.71-862)	1.78(1.16-2.64)	2.02(1.28-3.07)	0.42(0.38-0.45)
	Mild anemia	Both	16.34(36.92-5.81)	62.26(142.83-21.42)	0.01(0-0.02)	0.02(0.01-0.04)	2.46(2.17-2.75)	66.03(145.16-23.18)	219.54(476.18-75.44)	0.08(0.03-0.18)	0.13(0.05-0.29)	1.79(1.64-1.94)
		Female	7.25(16.53-2.57)	30.92(71.52-10.51)	0.01(0-0.02)	0.02(0.01-0.04)	2.87(2.57-3.17)	25.55(56.52-8.85)	86.69(189.55-29.64)	0.06(0.02-0.12)	0.09(0.03-0.2)	1.83(1.66-2)
		Male	9.09(20.8-3.25)	31.34(71.42-10.96)	0.01(0-0.03)	0.02(0.01-0.04)	2.15(1.86-2.44)	40.47(89.71-14.27)	132.85(288.49-46.65)	0.11(0.04-0.25)	0.18(0.06-0.39)	1.88(1.74-2.02)
	Moderate anemia	Both	371.49(591.53-223.65)	976.02(1555.28-586.84)	0.19(0.11-0.29)	0.23(0.14-0.35)	1.08(0.89-1.27)	1096.47(1640.42-696.47)	2769.56(4147.86-1755.97)	1.49(0.95-2.23)	1.83(1.17-2.73)	0.75(0.68-0.82)
		Female	183.09(294.59-108.86)	508.03(794.71-304.02)	0.2(0.12-0.31)	0.25(0.15-0.39)	1.27(1.05-1.5)	693.85(1035.34-441.66)	1813.81(2714.11-1147.39)	1.76(1.13-2.65)	2.18(1.39-3.24)	0.79(0.71-0.87)
		Male	188.4(306.83-112.67)	467.99(754.29-276.29)	0.17(0.1-0.26)	0.2(0.12-0.31)	0.84(0.69-0.99)	402.62(603.37-251.11)	955.75(1445.84-607.86)	1.21(0.77-1.8)	1.44(0.91-2.16)	0.62(0.57-0.67)
	Severe anemia	Both	154.15(242.93-92.8)	241.06(369.11-148.63)	0.07(0.04-0.11)	0.06(0.04-0.08)	-0.72(-0.83--0.61)	401.78(588.44-267.93)	731.7(1048.33-490.54)	0.57(0.38-0.84)	0.49(0.33-0.71)	-0.7(-0.76--0.63)
		Female	70.02(108.61-41.89)	120.64(182.97-75.03)	0.07(0.05-0.11)	0.06(0.04-0.09)	-0.47(-0.63--0.31)	260.75(379.63-173)	472.74(679.03-314.87)	0.68(0.46-1)	0.58(0.38-0.83)	-0.78(-0.84--0.71)
		Male	84.13(134.48-49.44)	120.42(191.72-70.73)	0.07(0.04-0.1)	0.05(0.03-0.08)	-1(-1.08--0.92)	141.02(204.77-93.07)	258.97(376.48-172.3)	0.46(0.3-0.66)	0.4(0.27-0.59)	-0.63(-0.69--0.56)
High-income Asia Pacific	Anemia	Both	247.49(399.56-142.73)	216.31(345.8-123.59)	0.14(0.08-0.22)	0.1(0.05-0.15)	-0.6(-0.81--0.38)	5307.37(8093.57-3250.12)	9552.41(15444.65-5712.02)	2.75(1.69-4.18)	1.85(1.1-3.01)	-1.5(-1.59--1.41)
		Female	167.49(272.67-96.97)	142.85(229.59-82.59)	0.18(0.1-0.3)	0.14(0.08-0.22)	-0.35(-0.57--0.13)	3326.19(5061.01-2025.64)	5435.84(8357.29-3397.38)	2.92(1.78-4.46)	1.88(1.19-2.9)	-1.6(-1.69--1.52)
		Male	80(137.19-43.5)	73.46(128.17-38.04)	0.09(0.05-0.16)	0.06(0.03-0.1)	-1.05(-1.3--0.8)	1981.18(3221.05-1152.78)	4116.57(6962.57-2268.28)	2.6(1.54-4.2)	1.87(1.04-3.16)	-1.33(-1.43--1.23)

		Both	30.04(68.62-10.13)	40.25(93.28-13.78)	0.02(0.01-0.04)	0.02(0.01-0.04)	0.74(0.51-0.98)	657.08(1434.28-232.23)	1626.74(3646.78-579.24)	0.33(0.12-0.72)	0.33(0.12-0.75)	-0.13(-0.18--0.07)
	Mild anemia	Female	14.17(32.59-4.63)	18.11(42.13-6.16)	0.02(0-0.04)	0.02(0.01-0.04)	1.12(0.82-1.42)	246.51(547.19-86.35)	581.19(1261.9-206.45)	0.22(0.08-0.48)	0.21(0.08-0.47)	-0.04(-0.11-0.03)
		Male	15.87(36.21-5.5)	22.15(51.59-7.22)	0.02(0.01-0.04)	0.02(0.01-0.04)	0.35(0.15-0.55)	410.58(892.76-146.06)	1045.55(2388.82-372.98)	0.5(0.18-1.09)	0.49(0.17-1.12)	-0.36(-0.44--0.29)
		Both	190.69(302.28-111.82)	160.34(250.07-94.19)	0.11(0.06-0.17)	0.07(0.04-0.11)	-0.7(-0.91--0.49)	4115.11(6202.42-2540.52)	7206.35(11048.81-4445.16)	2.14(1.32-3.21)	1.38(0.86-2.13)	-1.65(-1.75--1.56)
	Moderate anemia	Female	135.56(217.01-79.05)	114.81(179.18-67.75)	0.15(0.08-0.23)	0.11(0.06-0.17)	-0.4(-0.62--0.18)	2766.61(4221.3-1673.95)	4505.08(6882.68-2879.77)	2.43(1.48-3.67)	1.55(0.99-2.38)	-1.66(-1.74--1.58)
		Male	55.13(93.08-31.05)	45.54(73.64-25.04)	0.07(0.04-0.11)	0.04(0.02-0.06)	-1.37(-1.63--1.11)	1348.5(2083.69-815.68)	2701.27(4346.52-1543.2)	1.8(1.11-2.74)	1.21(0.68-1.96)	-1.53(-1.66--1.41)
		Both	26.76(44.7-15.67)	15.72(24.46-9.38)	0.01(0.01-0.02)	0.01(0-0.01)	-2.01(-2.34--1.69)	535.18(774.41-340.39)	719.32(1087.79-457.52)	0.28(0.18-0.41)	0.14(0.09-0.2)	-2.58(-2.82--2.33)
	Severe anemia	Female	17.77(29.03-10.17)	9.94(15.95-5.96)	0.02(0.01-0.03)	0.01(0.01-0.02)	-1.81(-2.11--1.52)	313.07(468.5-191.3)	349.58(563.83-205.61)	0.28(0.17-0.41)	0.12(0.07-0.19)	-2.96(-3.21--2.71)
		Male	8.99(16.19-4.86)	5.78(9.35-3.31)	0.01(0.01-0.02)	0(0-0.01)	-2.36(-2.77--1.95)	222.1(331.84-138.34)	369.74(574.13-221.82)	0.3(0.19-0.46)	0.17(0.1-0.25)	-2.19(-2.39--1.98)
High-income North America	Anemia	Both	373.75(614.41-216.54)	468.95(768.83-279.56)	0.13(0.07-0.21)	0.12(0.07-0.19)	0.06(-0.07-0.19)	4702.29(7345.14-2821)	9491.78(14952.27-5700.03)	1.28(0.77-2.01)	1.47(0.88-2.32)	1.2(0.9-1.5)
		Female	280.72(457.97-164.55)	329.6(532.05-195.66)	0.19(0.11-0.3)	0.17(0.1-0.27)	-0.14(-0.23--0.06)	3100.65(4743.55-1837.31)	5684.17(9248.63-3353.64)	1.45(0.86-2.25)	1.63(0.96-2.65)	1.14(0.82-1.45)
		Male	93.02(155.58-51.27)	139.35(232.35-76.38)	0.07(0.04-0.12)	0.07(0.04-0.12)	0.57(0.16-1)	1601.63(2573.03-964.03)	3807.61(6031.67-2143.3)	1.13(0.68-1.79)	1.33(0.75-2.12)	1.25(0.97-1.54)
	Mild anemia	Both	71.72(161.92-24.68)	92.67(202.47-32.18)	0.02(0.01-0.05)	0.02(0.01-0.05)	0.08(-0.03-0.2)	871.43(1909.43-302.69)	1706.92(3850.82-588.2)	0.24(0.08-0.52)	0.27(0.09-0.6)	0.79(0.59-0.98)
		Female	44.28(100.39-15.39)	49.02(109.35-17.2)	0.03(0.01-0.07)	0.03(0.01-0.06)	-0.38(-0.48--0.27)	403.87(931.93-143)	707.3(1590.85-253.45)	0.19(0.07-0.45)	0.21(0.07-0.47)	0.59(0.39-0.79)
		Male	27.44(63.07-9.45)	43.64(96.16-14.54)	0.02(0.01-0.04)	0.02(0.01-0.04)	0.73(0.37-1.09)	467.56(1045.06-165.9)	999.62(2199.23-340.06)	0.32(0.11-0.71)	0.35(0.12-0.77)	0.72(0.54-0.9)
	Moderate anemia	Both	280.69(430.84-171.32)	343.79(531.95-211.88)	0.1(0.06-0.15)	0.09(0.05-0.14)	0(-0.14-0.13)	3412.38(5094.67-2114.42)	6903.09(10522.57-4254.53)	0.93(0.58-1.39)	1.07(0.66-1.63)	1.27(0.94-1.6)
		Female	220.05(338.87-136.41)	257.29(408.21-158.46)	0.15(0.09-0.22)	0.13(0.08-0.2)	-0.16(-0.24--0.08)	2451.54(3786.7-1482.29)	4509.15(7192.8-2631.84)	1.15(0.7-1.74)	1.29(0.76-2.04)	1.19(0.86-1.53)
		Male	60.64(96.22-35.86)	86.49(135.74-50.34)	0.05(0.03-0.08)	0.05(0.03-0.08)	0.49(0.03-0.94)	960.84(1446.42-594.84)	2393.94(3705.96-1435.3)	0.69(0.43-1.04)	0.84(0.5-1.31)	1.5(1.17-1.83)
	Severe anemia	Both	21.34(31.18-13.82)	32.5(49.07-20.65)	0.01(0-0.01)	0.01(0-0.01)	0.75(0.59-0.91)	418.48(628.3-267.11)	881.77(1326.97-549.43)	0.11(0.07-0.17)	0.14(0.08-0.2)	1.52(1.18-1.86)
		Female	16.39(24.81-10.47)	23.29(35.45-14.37)	0.01(0.01-0.02)	0.01(0.01-0.02)	0.69(0.51-0.86)	245.25(376.13-150.23)	467.72(739.69-266.71)	0.11(0.07-0.17)	0.13(0.08-0.21)	1.54(1.18-1.91)
		Male	4.94(7.28-3.08)	9.22(14.19-5.7)	0(0-0.01)	0(0-0.01)	0.87(0.55-1.18)	173.23(264.87-108.18)	414.05(667.63-247.4)	0.13(0.08-0.19)	0.15(0.09-0.23)	1.23(0.91-1.55)
North Africa and Middle East	Anemia	Both	1023.09(1639.51-614.34)	1981.75(3273.67-1186.24)	0.27(0.17-0.43)	0.32(0.19-0.53)	0.72(0.66-0.79)	6580.78(9663.71-4287.49)	16203.57(24490.13-10090.32)	4.06(2.64-5.97)	4.03(2.53-6.12)	0.08(-0.03-0.19)
		Female	629.35(1024.52-375.71)	1343.64(2167.54-789.2)	0.35(0.22-0.56)	0.44(0.26-0.71)	0.92(0.84-1)	4950.02(7255.54-3273.81)	12237.13(18188.13-7806.68)	6.02(4.01-8.78)	6.11(3.88-8.96)	0.21(0.06-0.36)
		Male	393.74(629.27-230.55)	638.11(1064.74-362.79)	0.19(0.11-0.3)	0.21(0.12-0.35)	0.42(0.35-0.49)	1630.76(2519.13-1000.04)	3966.44(6570.48-2278.73)	2.09(1.28-3.22)	2(1.16-3.35)	-0.23(-0.28--0.18)
	Mild anemia	Both	64.11(148.91-21.42)	210.99(483.75-70.9)	0.02(0.01-0.05)	0.03(0.01-0.08)	2.15(2.1-2.2)	528.64(1158.6-181.8)	2165.52(4752.04-758.42)	0.31(0.11-0.69)	0.53(0.18-1.17)	1.82(1.69-1.94)
		Female	34.8(83.69-11.24)	122.12(286.34-39.64)	0.02(0.01-0.05)	0.04(0.01-0.09)	2.55(2.48-2.62)	206.51(459.78-70.85)	878.89(1912.33-304.47)	0.22(0.08-0.5)	0.41(0.14-0.9)	2.27(2.04-2.51)
		Male	29.31(65.46-9.9)	88.87(207.83-30.25)	0.02(0.01-0.04)	0.03(0.01-0.07)	1.74(1.67-1.82)	322.14(709.97-110.45)	1286.63(2831.24-451.85)	0.41(0.14-0.9)	0.64(0.23-1.41)	1.5(1.43-1.56)
	Moderate anemia	Both	820.2(1304.72-486.51)	1549.34(2517.02-920.06)	0.21(0.13-0.34)	0.25(0.15-0.4)	0.66(0.61-0.72)	4860.12(7251.9-3109.59)	12132.89(18097.14-7654.35)	2.95(1.88-4.38)	3.01(1.93-4.49)	0.19(0.06-0.33)
		Female	508.51(817.13-300.24)	1071.11(1703.94-619.3)	0.28(0.17-0.45)	0.35(0.21-0.56)	0.89(0.82-0.97)	3746.65(5568.29-2412.06)	9785.13(14547.82-6161.37)	4.47(2.87-6.61)	4.88(3.08-7.2)	0.46(0.29-0.64)
		Male	311.69(497.26-182.99)	478.23(795.95-268.53)	0.15(0.09-0.23)	0.16(0.09-0.26)	0.28(0.2-0.36)	1113.47(1647.47-700.78)	2347.76(3498.29-1459.15)	1.43(0.91-2.11)	1.19(0.75-1.78)	-0.73(-0.79--0.67)
	Severe anemia	Both	138.78(213.24-83.94)	221.41(343.55-131.04)	0.04(0.02-0.06)	0.04(0.02-0.06)	0.07(-0.06-0.2)	1192.02(1721.68-798.67)	1905.16(2737.49-1254.1)	0.8(0.54-1.15)	0.49(0.32-0.7)	-1.55(-1.62--1.48)
		Female	86.04(135.25-51.46)	150.41(243.41-88.83)	0.05(0.03-0.08)	0.05(0.03-0.08)	0.16(0.01-0.31)	996.87(1448.06-669.34)	1573.1(2245.81-1030.96)	1.33(0.89-1.91)	0.82(0.54-1.18)	-1.51(-1.6--1.43)
		Male	52.74(84.57-30.86)	71(115-40)	0.03(0.02-0.04)	0.02(0.01-0.04)	-0.03(-0.14-0.09)	195.15(281.13-125.03)	332.05(481.2-214.43)	0.25(0.16-0.36)	0.17(0.11-0.24)	-1.38(-1.42--1.33)
Oceania	Anemia	Both	13.66(28.1-6.02)	28.72(62.12-12.45)	0.17(0.08-0.33)	0.18(0.08-0.39)	0.41(0.33-0.5)	90.71(134.93-57.69)	259.43(380.17-165.22)	2.9(1.89-4.36)	3.67(2.36-5.38)	0.76(0.73-0.79)
		Female	6.81(14.39-2.91)	13.87(31.16-5.82)	0.18(0.08-0.37)	0.19(0.08-0.42)	0.26(0.13-0.38)	64.08(94.92-40.99)	179.04(262.24-114.69)	3.9(2.53-5.78)	4.94(3.19-7.23)	0.73(0.69-0.76)
		Male	6.85(14.05-3.08)	14.84(33.23-6)	0.15(0.07-0.31)	0.18(0.07-0.39)	0.59(0.53-0.65)	26.63(41.35-16.33)	80.39(125.7-46.86)	1.97(1.21-3.1)	2.48(1.46-3.85)	0.82(0.77-0.87)
	Mild anemia	Both	0.47(1.22-0.14)	1.22(3.37-0.36)	0.01(0-0.02)	0.01(0-0.02)	1.06(1.01-1.11)	6.99(15.37-2.44)	22.41(50.41-7.83)	0.23(0.08-0.51)	0.33(0.12-0.74)	1.05(0.99-1.1)
		Female	0.22(0.56-0.06)	0.59(1.73-0.15)	0.01(0-0.01)	0.01(0-0.02)	1.07(1.02-1.11)	2(4.47-0.69)	5.78(13.1-2.02)	0.11(0.04-0.24)	0.14(0.05-0.32)	0.72(0.65-0.79)
		Male	0.25(0.62-0.07)	0.63(1.62-0.19)	0.01(0-0.02)	0.01(0-0.02)	1.05(0.97-1.13)	4.99(10.92-1.74)	16.64(36.72-5.85)	0.36(0.12-0.8)	0.52(0.18-1.15)	1.11(1.05-1.17)
	Moderate anemia	Both	10.45(21.4-4.54)	22.51(49.4-9.71)	0.13(0.06-0.25)	0.14(0.06-0.31)	0.48(0.4-0.55)	64.37(97.09-40.71)	188.62(279.15-119.95)	2.06(1.33-3.09)	2.65(1.7-3.9)	0.81(0.78-0.85)

		Female	5.32(11.37-2.17)	10.99(25.34-4.35)	0.14(0.06-0.29)	0.15(0.06-0.34)	0.3(0.2-0.4)	45.71(69.55-28.63)	132.5(197.45-84.75)	2.76(1.76-4.14)	3.61(2.3-5.47)	0.81(0.76-0.85)
		Male	5.13(10.76-2.3)	11.52(26.3-4.74)	0.11(0.05-0.23)	0.14(0.06-0.3)	0.67(0.62-0.73)	18.66(28.56-11.78)	56.11(84.65-34.21)	1.43(0.89-2.17)	1.77(1.08-2.64)	0.82(0.75-0.9)
	Severe anemia	Both	2.73(5.91-1.17)	4.98(11.34-2.04)	0.03(0.02-0.07)	0.03(0.01-0.07)	0.02(-0.13-0.16)	19.35(29.15-12.2)	48.4(71.79-31.23)	0.61(0.39-0.9)	0.69(0.45-1)	0.45(0.42-0.48)
		Female	1.27(2.8-0.54)	2.29(5.33-0.97)	0.03(0.02-0.07)	0.03(0.01-0.07)	-0.09(-0.34-0.15)	16.37(24.91-10.22)	40.76(60.54-25.9)	1.04(0.67-1.55)	1.19(0.76-1.76)	0.5(0.48-0.53)
		Male	1.46(3.34-0.58)	2.69(6.64-0.97)	0.03(0.01-0.07)	0.03(0.01-0.08)	0.13(0.05-0.21)	2.98(4.48-1.84)	7.64(11.65-4.82)	0.18(0.12-0.27)	0.19(0.12-0.29)	0.15(0.11-0.19)
South Asia	Anemia	Both	3824.27(5619.92-2388.43)	5879.36(8676.62-3677.27)	0.33(0.21-0.49)	0.32(0.2-0.48)	0.52(0.29-0.74)	29899.59(42711.22-19668.13)	74198.41(107816.97-48290.46)	5.49(3.62-7.82)	5.53(3.61-8.06)	-0.1(-0.26-0.07)
		Female	1794.76(2678.04-1124.67)	2929.73(4405.65-1803.33)	0.33(0.21-0.49)	0.32(0.2-0.48)	0.48(0.23-0.74)	15188.85(21793.42-9959.51)	39878.32(57609.79-26202.32)	5.46(3.6-7.8)	5.67(3.74-8.24)	0.01(-0.13-0.14)
		Male	2029.51(2964.43-1241.67)	2949.63(4380.62-1810.13)	0.33(0.21-0.48)	0.33(0.2-0.48)	0.56(0.36-0.77)	14710.74(21205.81-9598.66)	34320.09(50249.99-22190.57)	5.52(3.64-7.97)	5.42(3.54-7.9)	-0.17(-0.37-0.03)
	Mild anemia	Both	103.46(233.35-36.16)	249.91(553.22-87.39)	0.01(0-0.02)	0.01(0-0.03)	1.95(1.65-2.26)	697.4(1501.72-247.22)	2280.87(4970-807.01)	0.11(0.04-0.24)	0.16(0.06-0.35)	1.42(1.32-1.52)
		Female	38.69(88.44-13.55)	93.96(208.94-33.18)	0.01(0-0.02)	0.01(0-0.02)	1.68(1.43-1.92)	208.34(447.27-73.15)	687.26(1473.68-243.63)	0.07(0.02-0.14)	0.09(0.03-0.2)	1.3(1.24-1.36)
		Male	64.77(143.35-22.43)	155.95(349.5-54.07)	0.01(0-0.03)	0.02(0.01-0.04)	2.17(1.83-2.52)	489.06(1063.07-172.19)	1593.62(3485.5-562.78)	0.15(0.05-0.33)	0.23(0.08-0.5)	1.63(1.51-1.75)
	Moderate anemia	Both	2551.08(3851.62-1566.61)	4350.23(6518.42-2636.7)	0.22(0.14-0.33)	0.24(0.15-0.35)	0.88(0.66-1.1)	17195.76(25842.16-11000.78)	48207.49(71438.7-30695)	3.05(1.93-4.54)	3.56(2.26-5.28)	0.49(0.34-0.63)
		Female	1184.75(1807.01-724.17)	2178.38(3317.54-1323.28)	0.22(0.13-0.33)	0.24(0.14-0.36)	0.9(0.66-1.15)	8518.5(12666.63-5491.69)	25619.78(37974.1-16317.05)	2.96(1.88-4.41)	3.61(2.3-5.34)	0.61(0.5-0.73)
		Male	1366.32(2042.12-818.23)	2171.85(3238.68-1300.24)	0.22(0.13-0.33)	0.24(0.14-0.36)	0.87(0.67-1.07)	8677.26(13109.62-5556.24)	22587.71(33575.42-14440.13)	3.13(1.99-4.68)	3.54(2.26-5.25)	0.41(0.23-0.59)
	Severe anemia	Both	1169.73(1780.3-753.17)	1279.22(1913.5-814.13)	0.1(0.07-0.16)	0.07(0.05-0.11)	-0.6(-0.84--0.36)	12006.43(17309.55-8039)	23710.05(33993.93-16007.77)	2.33(1.57-3.37)	1.81(1.22-2.6)	-1.09(-1.32--0.87)
		Female	571.32(880.09-363.36)	657.38(970.15-423.72)	0.11(0.07-0.17)	0.07(0.05-0.11)	-0.66(-0.94--0.38)	6462.01(9242.58-4356.84)	13571.29(19424.44-9206.33)	2.43(1.66-3.48)	1.97(1.34-2.82)	-0.91(-1.09--0.72)
		Male	598.41(924.5-376.65)	621.83(956.82-386.94)	0.1(0.06-0.15)	0.07(0.04-0.11)	-0.53(-0.74--0.33)	5544.42(7987.62-3665.23)	10138.76(14657.35-6907.18)	2.24(1.51-3.24)	1.66(1.11-2.39)	-1.3(-1.56--1.04)
Southeast Asia	Anemia	Both	1090.22(1690.84-637.13)	1161.54(1748.65-683.09)	0.2(0.12-0.31)	0.18(0.1-0.27)	-0.23(-0.45--0.01)	8808.14(13015.31-5623.58)	22834.84(34214.51-14475.78)	3.56(2.28-5.23)	4.04(2.56-6.08)	0.35(0.32-0.37)
		Female	608.24(938.51-357.57)	718.65(1103.11-434.85)	0.23(0.14-0.35)	0.21(0.13-0.33)	0.18(-0.13-0.49)	5903.08(8746.31-3723.96)	15244.18(22815.64-9590.51)	4.3(2.79-6.33)	4.85(3.07-7.19)	0.35(0.33-0.37)
		Male	481.98(754.38-275.18)	442.89(717.6-248.46)	0.18(0.1-0.27)	0.14(0.08-0.23)	-0.76(-0.85--0.66)	2905.06(4410.97-1830.41)	7590.66(11772.02-4642.5)	2.73(1.75-4.09)	3.08(1.9-4.75)	0.28(0.22-0.34)
	Mild anemia	Both	59.44(137.54-20.36)	109.32(238.22-37.52)	0.01(0-0.03)	0.02(0.01-0.03)	1.6(1.27-1.93)	501.65(1097.21-175.31)	2093.54(4576.72-735.18)	0.19(0.06-0.4)	0.35(0.12-0.77)	2.25(2.19-2.31)
		Female	32.84(80.01-11.26)	59.99(132.93-21.02)	0.01(0-0.03)	0.02(0.01-0.04)	1.69(1.36-2.03)	228.25(502.43-80.08)	927.72(2023.7-327.08)	0.15(0.05-0.33)	0.29(0.1-0.62)	2.33(2.26-2.41)
		Male	26.59(59.95-9.03)	49.32(110.36-16.79)	0.01(0-0.03)	0.01(0-0.03)	1.54(1.2-1.87)	273.41(600.56-95.45)	1165.81(2586.74-415.93)	0.23(0.08-0.5)	0.44(0.16-0.98)	2.25(2.19-2.3)
	Moderate anemia	Both	880.19(1358.86-510.19)	940.33(1418.35-560.31)	0.16(0.1-0.25)	0.14(0.08-0.22)	-0.2(-0.42-0.02)	6627.5(9897.42-4229.62)	17855.78(26940.15-11063.29)	2.67(1.69-3.99)	3.16(2-4.76)	0.5(0.46-0.53)
		Female	491.41(775.63-284.59)	590.46(901.3-356.35)	0.19(0.11-0.29)	0.18(0.11-0.27)	0.26(-0.06-0.58)	4547.26(6817.06-2851.18)	12459.74(18771.71-7838.12)	3.29(2.07-4.88)	3.96(2.5-5.91)	0.57(0.53-0.61)
		Male	388.78(621.18-220.28)	349.87(559.8-195.79)	0.14(0.08-0.22)	0.11(0.06-0.18)	-0.83(-0.91--0.75)	2080.24(3106.47-1305.34)	5396.04(8185.76-3286.11)	1.96(1.23-2.94)	2.22(1.36-3.33)	0.26(0.2-0.33)
	Severe anemia	Both	150.59(232.4-89.93)	111.89(167.59-69.4)	0.03(0.02-0.04)	0.02(0.01-0.02)	-1.56(-1.76--1.37)	1678.98(2428.83-1126.81)	2885.52(4145.35-1905.85)	0.71(0.47-1.03)	0.52(0.34-0.75)	-1.16(-1.23--1.1)
		Female	83.98(131.23-49.6)	68.19(104.16-42.57)	0.03(0.02-0.05)	0.02(0.01-0.03)	-1.27(-1.52--1.03)	1127.57(1630.23-734.1)	1856.72(2726.28-1213.07)	0.86(0.56-1.24)	0.6(0.39-0.88)	-1.31(-1.36--1.25)
		Male	66.61(107.73-38.08)	43.7(66.57-26.06)	0.02(0.01-0.04)	0.01(0.01-0.02)	-1.91(-2.03--1.78)	551.41(815.42-359.33)	1028.81(1517.91-664.01)	0.53(0.35-0.78)	0.42(0.27-0.61)	-0.97(-1.04--0.89)
Southern Latin America	Anemia	Both	80.97(156.8-39.96)	83.78(162.27-41.58)	0.16(0.08-0.31)	0.13(0.06-0.25)	-0.51(-0.67--0.34)	742.93(1131.13-472.26)	1251.37(1932.09-772.59)	1.69(1.07-2.54)	1.47(0.9-2.27)	-0.51(-0.53--0.5)
		Female	45.15(90.16-21.82)	52.62(102.7-25.51)	0.18(0.09-0.36)	0.15(0.07-0.31)	-0.19(-0.36--0.01)	356.42(554.75-223.8)	554.48(889.75-328.8)	1.41(0.88-2.19)	1.12(0.66-1.78)	-0.79(-0.82--0.77)
		Male	35.83(71.3-16.96)	31.16(60.97-15.21)	0.15(0.07-0.29)	0.1(0.05-0.2)	-0.94(-1.12--0.76)	386.5(595.44-229.79)	696.89(1114.08-424.07)	2.1(1.27-3.2)	1.99(1.21-3.17)	-0.26(-0.31--0.21)
	Mild anemia	Both	9.56(24.04-3.13)	13.75(33.51-4.42)	0.02(0.01-0.05)	0.02(0.01-0.05)	0.5(0.25-0.76)	86.31(191.86-30.72)	193.47(405.49-67.6)	0.19(0.07-0.42)	0.23(0.08-0.48)	0.62(0.53-0.7)
		Female	5.01(13.67-1.5)	8(20.45-2.44)	0.02(0.01-0.05)	0.02(0.01-0.06)	0.81(0.53-1.09)	35.48(81.58-12.47)	67.69(149.47-24.19)	0.14(0.05-0.32)	0.14(0.05-0.31)	0.02(-0.05-0.1)
		Male	4.55(10.96-1.52)	5.75(13.36-1.83)	0.02(0.01-0.05)	0.02(0.01-0.04)	0.14(-0.12-0.41)	50.84(110.62-17.92)	125.78(271.54-43.73)	0.26(0.09-0.56)	0.35(0.12-0.76)	1(0.89-1.11)
	Moderate anemia	Both	63.59(124.31-31.13)	63.76(122.55-31.62)	0.13(0.06-0.25)	0.1(0.05-0.19)	-0.59(-0.75--0.44)	549.61(818.3-346.99)	902.14(1359.32-566.44)	1.25(0.79-1.86)	1.06(0.66-1.58)	-0.61(-0.63--0.59)
		Female	36.56(72.51-17.98)	41.48(79.36-20.41)	0.14(0.07-0.29)	0.12(0.06-0.24)	-0.27(-0.45--0.1)	282.12(442.03-177.02)	432.76(669.05-255.71)	1.12(0.7-1.73)	0.87(0.51-1.36)	-0.84(-0.87--0.81)
		Male	27.03(54.47-12.5)	22.28(45.27-10.51)	0.11(0.05-0.21)	0.07(0.03-0.15)	-1.07(-1.23--0.9)	267.49(409.57-157.44)	469.38(736.12-285.27)	1.46(0.88-2.22)	1.35(0.82-2.07)	-0.36(-0.42--0.31)
	Severe anemia	Both	7.82(13.7-4.17)	6.26(11.01-3.49)	0.02(0.01-0.03)	0.01(0-0.02)	-1.42(-1.61--1.22)	107(159.73-69)	155.76(239.08-94.76)	0.25(0.16-0.37)	0.18(0.11-0.28)	-1.11(-1.15--1.08)
		Female	3.58(6.78-1.86)	3.14(6.22-1.61)	0.01(0.01-0.03)	0.01(0-0.02)	-1.13(-1.28--0.97)	38.82(63.26-23.19)	54.03(87.18-31.54)	0.16(0.1-0.26)	0.1(0.06-0.17)	-1.37(-1.43--1.3)

		Male	4.25(7.68-2.21)	3.12(5.49-1.68)	0.02(0.01-0.03)	0.01(0-0.02)	-1.65(-1.88--1.41)	68.18(105.33-41.39)	101.73(160.09-58.59)	0.38(0.24-0.58)	0.29(0.17-0.46)	-0.96(-1--0.93)
Southern Sub-Saharan Africa	Anemia	Both	147(248.19-85.56)	238.75(410.27-139.2)	0.27(0.16-0.44)	0.29(0.17-0.5)	0.5(0.33-0.66)	683.09(1002.96-439.41)	1572.94(2328.94-1012.08)	2.46(1.6-3.62)	2.91(1.87-4.31)	0.51(0.46-0.57)
		Female	92.34(156.61-53.5)	159.14(272.07-91.07)	0.33(0.19-0.54)	0.38(0.22-0.65)	0.68(0.44-0.93)	446.56(657.73-284.84)	1011.38(1492.33-660.94)	2.69(1.73-3.96)	3.11(2.02-4.63)	0.41(0.3-0.51)
		Male	54.66(89.98-29.79)	79.62(137.36-44.9)	0.2(0.11-0.32)	0.21(0.12-0.34)	0.31(0.24-0.39)	236.53(357.74-144.43)	561.56(857.84-343.87)	2.26(1.42-3.4)	2.78(1.7-4.24)	0.66(0.62-0.71)
	Mild anemia	Both	9.08(21.15-3.03)	18.45(42.97-6.09)	0.02(0.01-0.04)	0.02(0.01-0.05)	1.14(0.83-1.45)	49.32(109.14-16.93)	136.29(297.7-47.64)	0.18(0.06-0.39)	0.25(0.09-0.55)	1.05(0.75-1.35)
		Female	4.52(10.94-1.54)	9.45(22.25-3.05)	0.02(0.01-0.04)	0.02(0.01-0.05)	1.37(0.99-1.75)	19.98(43.9-6.87)	53.82(118.14-18.95)	0.12(0.04-0.26)	0.16(0.06-0.36)	0.99(0.68-1.3)
		Male	4.56(10.54-1.54)	9.01(20.93-2.97)	0.02(0.01-0.05)	0.02(0.01-0.06)	1.02(0.78-1.27)	29.34(64.28-10.01)	82.48(179.55-28.38)	0.27(0.09-0.58)	0.39(0.13-0.86)	1.23(0.97-1.49)
	Moderate anemia	Both	113.21(197.25-64.63)	184.37(322.87-105.12)	0.2(0.12-0.33)	0.23(0.13-0.39)	0.54(0.36-0.72)	495.04(734.85-317.27)	1150.8(1698.48-740.84)	1.78(1.14-2.64)	2.13(1.36-3.16)	0.54(0.46-0.62)
		Female	70.48(123.2-39.78)	123.18(209.55-69.57)	0.25(0.14-0.41)	0.29(0.17-0.5)	0.76(0.5-1.02)	330.57(500.87-205.38)	765.71(1151.08-490.22)	1.99(1.24-2.97)	2.35(1.51-3.51)	0.47(0.33-0.62)
		Male	42.73(71.07-22.73)	61.19(105.44-32.81)	0.15(0.08-0.24)	0.16(0.09-0.27)	0.29(0.22-0.36)	164.47(252.99-100.05)	385.1(576.8-238.25)	1.59(0.98-2.42)	1.94(1.2-2.89)	0.63(0.58-0.67)
	Severe anemia	Both	24.71(39.51-14.86)	35.93(56.12-21.89)	0.05(0.03-0.07)	0.04(0.03-0.07)	0.03(-0.04-0.1)	138.73(201.61-91.65)	285.84(410.11-187.51)	0.49(0.33-0.71)	0.53(0.35-0.76)	0.2(0.05-0.34)
		Female	17.34(28.84-10.15)	26.51(41.96-16.18)	0.06(0.04-0.1)	0.06(0.04-0.1)	0.18(0.05-0.3)	96.01(141-60.99)	191.85(282.69-125.03)	0.58(0.37-0.83)	0.59(0.38-0.88)	0.03(-0.07-0.13)
		Male	7.37(11.82-4.24)	9.42(15.08-5.69)	0.03(0.02-0.05)	0.03(0.02-0.04)	-0.12(-0.3-0.05)	42.72(64-27)	93.99(143.36-59.13)	0.41(0.26-0.61)	0.46(0.29-0.68)	0.41(0.19-0.64)
Tropical Latin America	Anemia	Both	416.72(679.46-245.87)	777.7(1235.52-468.69)	0.26(0.16-0.42)	0.34(0.2-0.54)	0.82(0.68-0.97)	2843.87(4310.64-1808.24)	6514.16(10016.15-4055.11)	3.13(1.99-4.7)	2.71(1.69-4.15)	-0.65(-0.72--0.59)
		Female	276.06(443.04-165.01)	617.22(977.03-373.22)	0.34(0.21-0.53)	0.52(0.31-0.83)	1.41(1.27-1.54)	1815.1(2741.78-1146.9)	4286.9(6578.77-2692.01)	3.62(2.27-5.44)	3.22(2.02-4.93)	-0.52(-0.58--0.46)
		Male	140.66(244.35-77.52)	160.48(278.21-88.13)	0.17(0.1-0.29)	0.16(0.09-0.28)	-0.61(-0.85--0.37)	1028.77(1620.01-609.95)	2227.26(3635.28-1311.43)	2.62(1.59-4.02)	2.16(1.29-3.49)	-0.87(-0.95--0.8)
	Mild anemia	Both	23.55(52.46-8.06)	64.53(144.25-21.8)	0.02(0.01-0.04)	0.03(0.01-0.06)	1.65(1.33-1.96)	210.28(473.61-72.46)	632.67(1413.37-220.98)	0.23(0.08-0.51)	0.26(0.09-0.59)	0.3(0.22-0.38)
		Female	12.06(27.45-4.12)	40.74(92.05-14.13)	0.01(0.01-0.03)	0.03(0.01-0.08)	2.77(2.55-2.99)	73.26(165.94-25.95)	241.83(524.75-84.54)	0.14(0.05-0.33)	0.18(0.06-0.4)	0.71(0.63-0.79)
		Male	11.49(27.09-3.87)	23.79(53.91-7.91)	0.02(0.01-0.04)	0.02(0.01-0.05)	0.36(-0.13-0.85)	137.02(314-47.15)	390.84(886.27-134.98)	0.32(0.11-0.74)	0.36(0.13-0.83)	0.16(0.07-0.24)
	Moderate anemia	Both	318.31(519.34-185.14)	611.63(957.8-368.41)	0.2(0.12-0.32)	0.27(0.16-0.42)	0.97(0.82-1.11)	2081.69(3174.55-1296.31)	4860.05(7418.5-2990.62)	2.28(1.42-3.44)	2.02(1.24-3.06)	-0.59(-0.65--0.52)
		Female	208.74(335.7-124.03)	492.11(776.03-294.8)	0.26(0.15-0.41)	0.41(0.25-0.66)	1.59(1.44-1.73)	1362.48(2061.07-818.29)	3378.54(5177.88-2112.41)	2.71(1.64-4.09)	2.54(1.59-3.9)	-0.35(-0.42--0.29)
		Male	109.57(188.98-59.21)	119.52(199.43-65.8)	0.13(0.07-0.22)	0.12(0.07-0.2)	-0.61(-0.82--0.39)	719.21(1127.56-428.09)	1481.51(2388.65-878.16)	1.83(1.1-2.84)	1.44(0.85-2.32)	-1.04(-1.13--0.96)
	Severe anemia	Both	74.85(124.63-42.87)	101.54(166.03-62.2)	0.05(0.03-0.08)	0.04(0.03-0.07)	-0.29(-0.42--0.17)	551.9(824.09-349.07)	1021.44(1554.87-640.03)	0.62(0.39-0.93)	0.43(0.27-0.66)	-1.38(-1.44--1.32)
		Female	55.25(95.89-30.52)	84.37(140.69-49.29)	0.07(0.04-0.11)	0.07(0.04-0.12)	0.09(-0.04-0.22)	379.36(595.98-235.24)	666.54(1036.32-399.96)	0.76(0.48-1.2)	0.5(0.3-0.78)	-1.56(-1.63--1.49)
		Male	19.6(35.33-10.55)	17.17(28.15-9.94)	0.02(0.01-0.04)	0.02(0.01-0.03)	-1.56(-1.8--1.32)	172.54(278.48-98.59)	354.9(554.27-211.17)	0.46(0.27-0.74)	0.35(0.21-0.55)	-1.06(-1.12--1.01)
Western Europe	Anemia	Both	208.15(344.84-123.09)	228.08(376-133.32)	0.05(0.03-0.09)	0.05(0.03-0.08)	0.13(-0.11-0.37)	4708.05(7417.91-2954.62)	5042.24(8093.25-2992.32)	0.78(0.5-1.22)	0.49(0.3-0.78)	-1.71(-1.83--1.59)
		Female	140.81(230.33-84.88)	162.42(264.59-94.88)	0.07(0.04-0.11)	0.07(0.04-0.11)	0.56(0.27-0.85)	3459.78(5395.7-2151.33)	3388.07(5275.91-2048.23)	0.94(0.59-1.46)	0.59(0.36-0.92)	-1.7(-1.84--1.56)
		Male	67.34(115.07-35.7)	65.66(115.21-34.77)	0.04(0.02-0.07)	0.03(0.02-0.05)	-0.74(-0.92--0.57)	1248.27(2083.92-717.64)	1654.17(2909-901.73)	0.56(0.32-0.93)	0.38(0.2-0.66)	-1.51(-1.58--1.44)
	Mild anemia	Both	44.98(100.27-14.99)	57.93(130.15-20.12)	0.01(0-0.02)	0.01(0-0.03)	0.74(0.45-1.03)	874.58(1978.21-308.39)	1200.77(2657.4-424.21)	0.15(0.05-0.33)	0.12(0.04-0.27)	-0.78(-0.84--0.72)
		Female	23.78(54.23-8.02)	31.87(73.47-11.25)	0.01(0-0.03)	0.01(0-0.03)	1.13(0.79-1.47)	417.79(937.66-145.59)	499.69(1117.36-176.4)	0.12(0.04-0.26)	0.09(0.03-0.21)	-0.91(-0.98--0.85)
		Male	21.2(48.89-7.1)	26.06(57.36-8.73)	0.01(0-0.02)	0.01(0-0.02)	0.2(-0.07-0.47)	456.79(1007.94-162.77)	701.08(1585.4-241.64)	0.2(0.07-0.45)	0.16(0.06-0.36)	-0.9(-0.95--0.85)
	Moderate anemia	Both	152.3(229.42-96.33)	159.71(242.31-98.75)	0.04(0.03-0.06)	0.03(0.02-0.05)	-0.02(-0.25-0.2)	3403.96(5110.68-2141.95)	3470.02(5288.26-2198.2)	0.56(0.36-0.85)	0.34(0.22-0.51)	-1.9(-2.04--1.77)
		Female	109.04(167.01-67.65)	122.71(188.88-74.5)	0.05(0.03-0.08)	0.05(0.03-0.08)	0.46(0.18-0.74)	2708.3(4110.6-1690.38)	2616.79(3973.68-1623.12)	0.73(0.47-1.11)	0.46(0.29-0.69)	-1.77(-1.92--1.62)
		Male	43.26(67.71-25.53)	36.99(57.31-22.3)	0.03(0.02-0.04)	0.02(0.01-0.03)	-1.14(-1.28--1.01)	695.66(1067.71-432.54)	853.23(1309.91-521.62)	0.32(0.2-0.48)	0.19(0.12-0.29)	-1.85(-1.93--1.76)
	Severe anemia	Both	10.87(15.95-7.05)	10.44(15.38-6.65)	0(0-0)	0(0-0)	-0.36(-0.62--0.1)	429.51(634.49-279.93)	371.44(543.57-241.31)	0.07(0.05-0.1)	0.03(0.02-0.05)	-2.56(-2.73--2.39)
		Female	7.99(11.86-5.22)	7.83(11.71-5)	0(0-0.01)	0(0-0)	-0.07(-0.34-0.2)	333.69(493.6-213.04)	271.59(400.63-171.93)	0.09(0.06-0.13)	0.04(0.03-0.06)	-2.47(-2.68--2.27)
		Male	2.88(4.26-1.81)	2.6(3.85-1.62)	0(0-0)	0(0-0)	-1.18(-1.42--0.94)	95.82(141.33-61.34)	99.86(151.03-61.72)	0.04(0.03-0.06)	0.02(0.01-0.03)	-2.39(-2.49--2.29)
Western Sub-Saharan Africa	Anemia	Both	906.14(1355.99-568.36)	2802.35(4162.94-1777.75)	0.42(0.26-0.61)	0.55(0.36-0.82)	1.33(1.2-1.47)	2159.69(3185.12-1398.85)	5849.03(8712.65-3806.99)	2.51(1.64-3.75)	3.17(2.04-4.72)	0.91(0.84-0.98)
		Female	479.17(724.14-297.17)	1586.17(2394.01-983.7)	0.48(0.3-0.71)	0.65(0.41-0.98)	1.53(1.38-1.69)	1450.2(2135.59-932.11)	3904.51(5851.2-2467.97)	3.2(2.06-4.67)	3.77(2.4-5.62)	0.65(0.59-0.71)
		Male	426.97(628.39-264.74)	1216.19(1809.4-771.22)	0.36(0.23-0.53)	0.44(0.28-0.64)	0.95(0.84-1.06)	709.5(1070.14-456.96)	1944.52(2915.37-1247.56)	1.86(1.21-2.78)	2.51(1.62-3.77)	1.14(1.04-1.24)

	Both	32.41(73.94-11.46)	119.39(266.75-41.75)	0.02(0.01-0.04)	0.03(0.01-0.06)	1.91(1.72-2.09)	136.17(302.75-47.53)	438.3(970.31-152.81)	0.15(0.05-0.34)	0.24(0.08-0.52)	1.66(1.53-1.79)
Mild anemia	Female	16.87(38.28-5.81)	65.83(153.78-23.14)	0.02(0.01-0.04)	0.03(0.01-0.07)	1.92(1.74-2.1)	71.64(162.38-25.08)	218.52(486.7-76.7)	0.15(0.05-0.35)	0.22(0.07-0.48)	1.23(1.12-1.33)
	Male	15.53(35.12-5.5)	53.56(121.37-18.63)	0.02(0.01-0.04)	0.03(0.01-0.06)	1.93(1.74-2.13)	64.54(142.11-22.1)	219.79(482.72-76)	0.15(0.05-0.34)	0.26(0.09-0.57)	2.02(1.86-2.18)
	Both	609.68(917.64-377.54)	2036.99(3052.39-1264.13)	0.29(0.18-0.43)	0.41(0.26-0.61)	1.58(1.44-1.72)	1666.88(2494.24-1069.68)	4592.21(6835.34-2952.86)	1.94(1.25-2.91)	2.49(1.6-3.68)	0.97(0.9-1.05)
Moderate anemia	Female	336.37(514.27-206.12)	1203.43(1836.23-739.53)	0.35(0.21-0.52)	0.5(0.31-0.76)	1.77(1.6-1.94)	1155.85(1723.4-739.49)	3181.01(4816.62-2036.03)	2.57(1.62-3.78)	3.09(1.96-4.62)	0.74(0.67-0.8)
	Male	273.32(412.17-165.46)	833.56(1278.43-497.33)	0.24(0.15-0.36)	0.3(0.19-0.45)	1.15(1.03-1.27)	511.03(768.77-325.8)	1411.2(2105.53-907.74)	1.34(0.85-2.01)	1.83(1.18-2.74)	1.2(1.1-1.3)
	Both	264.06(401.21-168.98)	645.97(979.88-403.19)	0.11(0.07-0.16)	0.11(0.07-0.17)	0.48(0.34-0.62)	356.64(513.47-237.38)	818.52(1167.99-534.36)	0.41(0.27-0.6)	0.44(0.29-0.62)	0.27(0.17-0.37)
Severe anemia	Female	125.94(193.59-79.28)	316.9(483.66-199.06)	0.11(0.07-0.17)	0.12(0.08-0.18)	0.61(0.47-0.75)	222.72(325.37-144.53)	504.98(743.1-325.43)	0.48(0.31-0.69)	0.46(0.29-0.68)	-0.07(-0.15-0)
	Male	138.12(213.72-85.02)	329.07(520.12-198.95)	0.11(0.07-0.16)	0.11(0.07-0.17)	0.27(0.1-0.44)	133.93(193.52-86.68)	313.54(453.85-203.6)	0.37(0.24-0.53)	0.42(0.27-0.61)	0.49(0.34-0.63)

Abbreviation: ASR, age-standardized rate; CI, confidence interval; EAPC, estimated annual percentage change; UI, uncertainty interval; YLD, years lived with disability

Figure S1. Ranking the prevalent cases, deaths and DALYs of non-communicable diseases per 100,000 worldwide.

Figure legends: (A) prevalent cases per 100,000 among both sexes; (B) deaths per 100,000 among both sexes; (C) DALYs per 100,000 among both sexes. DALY, disability adjusted life-year.

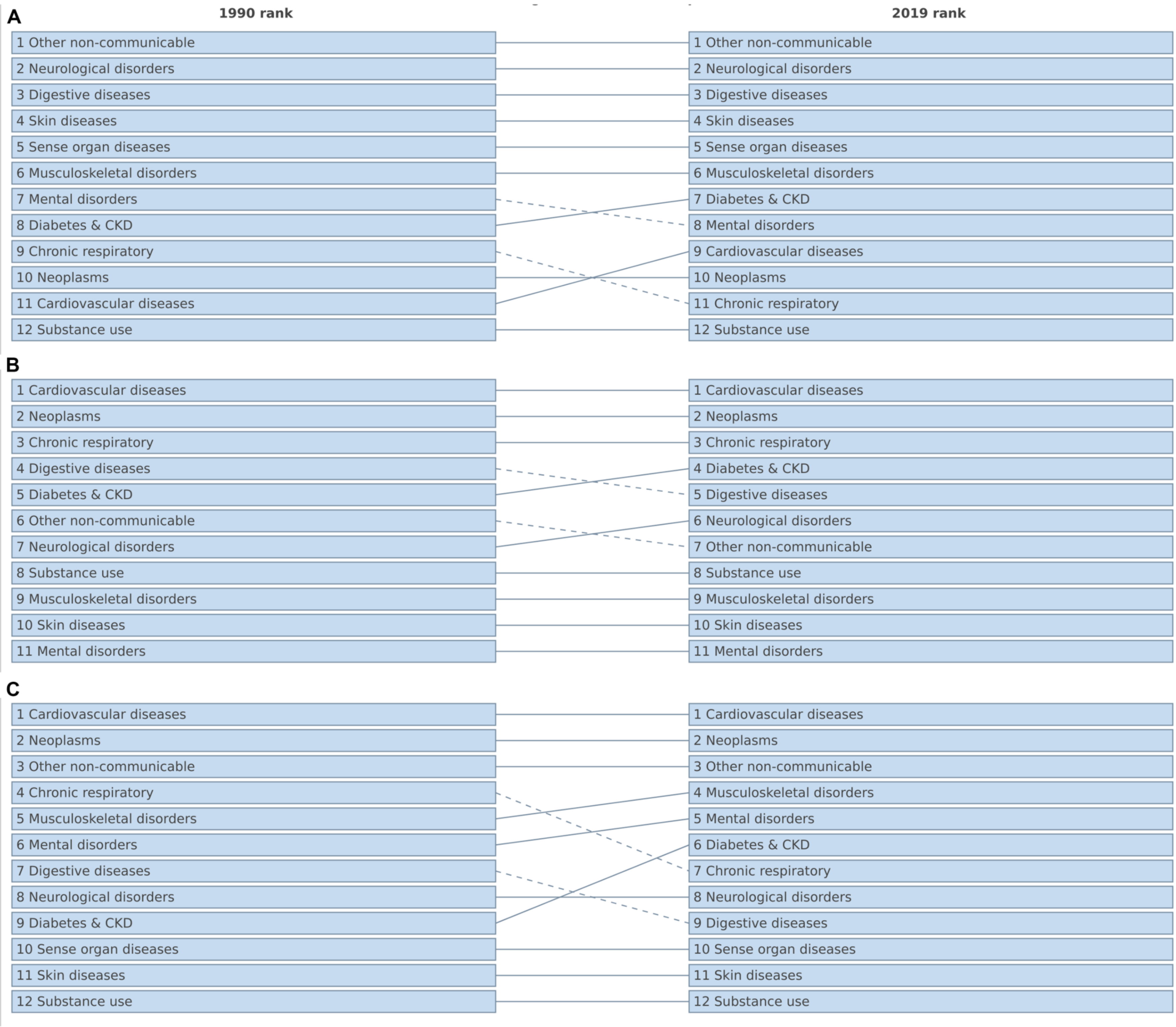

Figure S2. Ranking the prevalent cases, deaths and DALYs of chronic kidney disease per 100,000 worldwide.

Figure legends: (A) new cases per 100,000 among both sexes; (B) prevalent cases per 100,000 among both sexes; (C) deaths per 100,000 among both sexes; (D) DALYs per 100,000 among both sexes.

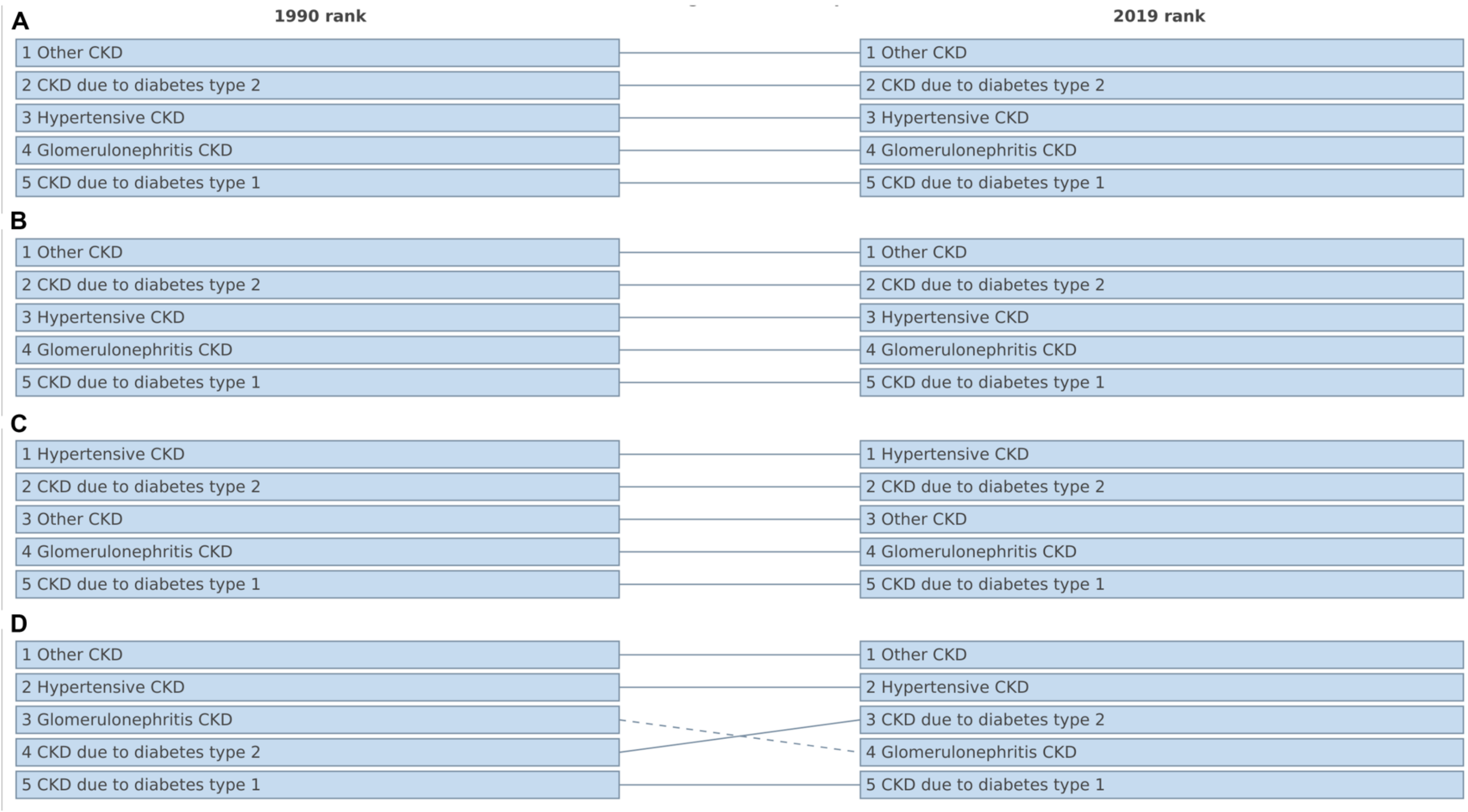

Figure S3. The number of patients with type 2 diabetes related CKD over 30 years.

Figure legends: CKD, chronic kidney disease

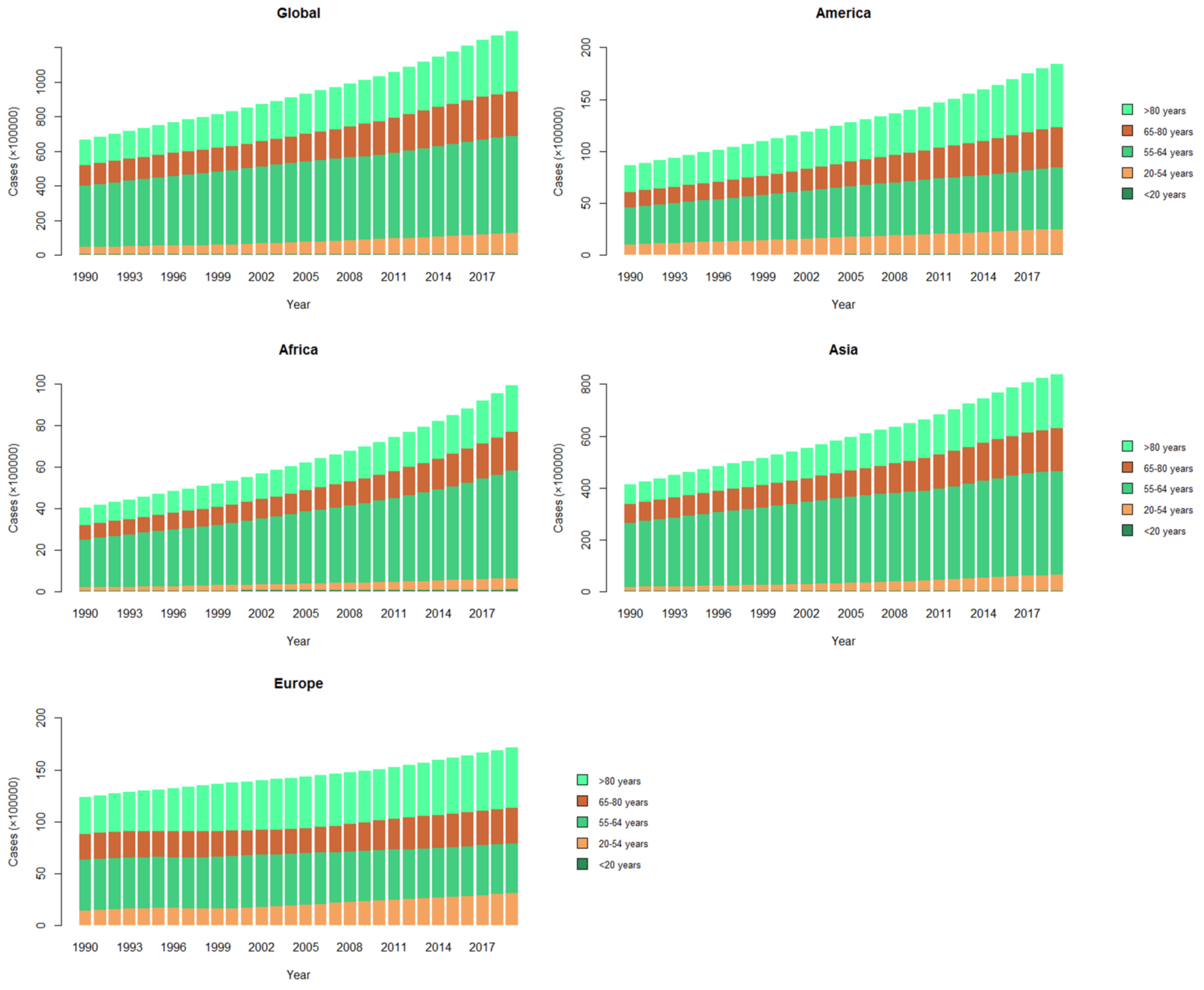

Figure S4. The number of type 2 diabetes related CKD deaths over 30 years.

Figure legends: The vertical axis is the deaths of type 2 diabetes related CKD in four world regions (America, Asia, Africa, and Europe). The horizontal axis represents 30 years (1990-2019). Each column is the total number of deaths among five age-groups (>80years, 65-80 years, 55-64 years, 20-54 years, and <20 years) that year. CKD, chronic kidney disease. CKD, chronic kidney disease

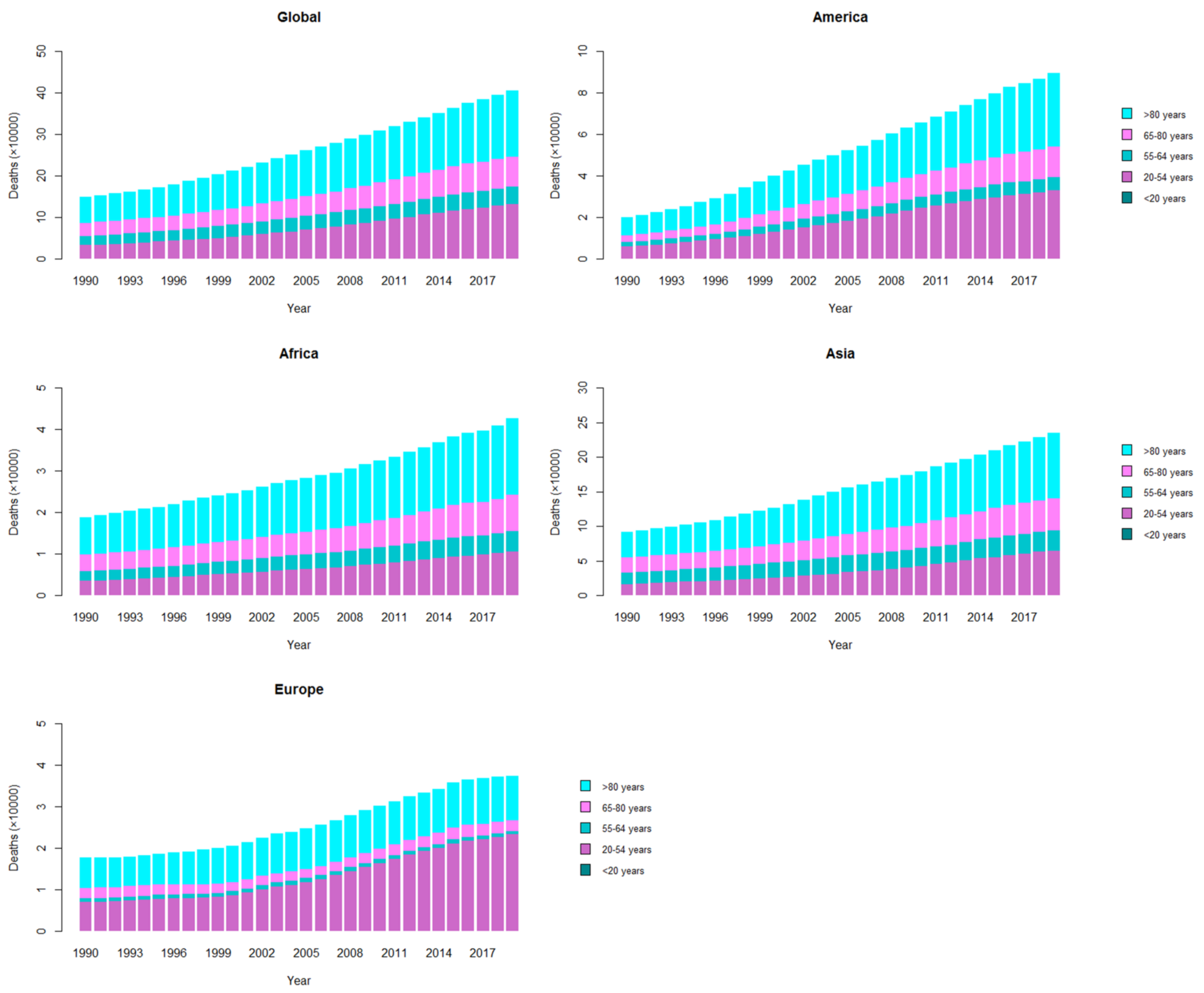

Figure S5. The number of type 2 diabetes related CKD DALYs over 30 years.

Figure legends: The vertical axis is the DALYs of type 2 diabetes related CKD in four world regions (America, Asia, Africa, and Europe). The horizontal axis represents 30 years (1990-2019). Each column is the total number of DALYs among five age-groups (>80years, 65-80 years, 55-64 years, 20-54 years, and <20 years) that year. CKD, chronic kidney disease. CKD, chronic kidney disease；DALY: disability adjusted life-year.

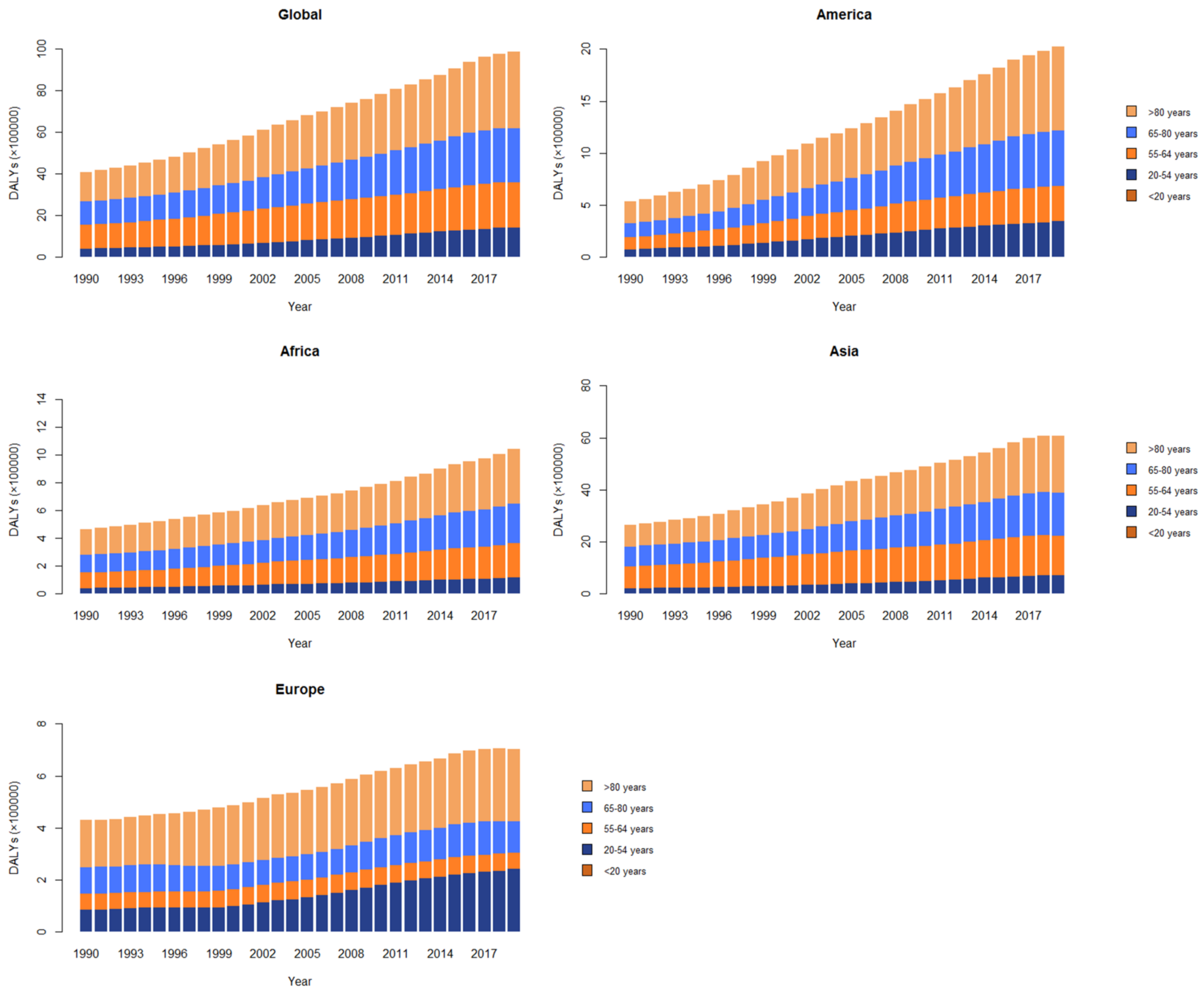

Supplement: Supplementary file 1 [file DataSheet_1.pdf]
